# Targeted RNA-Seq Reveals the *M. tuberculosis* Transcriptome from an In Vivo Infection Model

**DOI:** 10.3390/biology10090848

**Published:** 2021-08-31

**Authors:** Fernanda Cornejo-Granados, Gamaliel López-Leal, Dulce A. Mata-Espinosa, Jorge Barrios-Payán, Brenda Marquina-Castillo, Edgar Equihua-Medina, Zyanya L. Zatarain-Barrón, Camilo Molina-Romero, Rogelio Hernández-Pando, Adrian Ochoa-Leyva

**Affiliations:** 1 Departamento de Microbiología Molecular, Instituto de Biotecnología, Universidad Nacional Autonóma de México, Cuernavaca 62210, Mexico; fernanda.cornejo@ibt.unam.mx (F.C.-G.); gamlopez@ccg.unam.mx (G.L.-L.); edgar.equihua@uaem.edu.mx (E.E.-M.); 2 Sección de Patología Experimental, Departamento de Patología, Instituto Nacional de Ciencias Médicas y Nutrición “Salvador Zubirán”, Vasco de Quiroga 15, Tlalpan, Sección XVI, Ciudad de México 14000, Mexico; dulmat@comunidad.unam.mx (D.A.M.-E.); jorge.barriosp@incmnsz.mx (J.B.-P.); brenda.marquina@comunidad.unam.mx (B.M.-C.); zyanyal@comunidad.unam.mx (Z.L.Z.-B.); camilomol@comunidad.unam.mx (C.M.-R.)

**Keywords:** transcriptome, tuberculosis, host-pathogen, RNA-seq, in vivo infection

## Abstract

**Simple Summary:**

High-throughput sequencing techniques such as RNA-seq allow a more detailed characterization of the gene expression profile during in vivo infections. However, using this strategy for intracellular pathogens such as *Mycobacterium tuberculosis* (Mtb) entails technical limitations. Some authors have resorted to flow cytometers to separate infected cells or significantly increase sequencing depth to obtain pathogens’ gene expression. However, these options carry additional expenses in specialized equipment. We propose an experimental protocol based on differential cell lysis and a probe-based ribosomal depletion to determine the gene expression of Mtb and its host during in vivo infection. This method allowed us to increase the number of observed expressed genes from 13 using a traditional RNA-seq approach to 702. In addition, we observed the expression of genes essential for establishing the infection, codifying proteins such as PE-PGRS, lipoproteins lppN and LpqH, and three ncRNAs (small RNA MTS2823, transfer-messenger RNA RF00023, and ribozyme RF00010). We believe our method represents a valuable alternative to current RNA-seq approaches to study host–pathogen interactions and will help explore host–pathogen mechanisms in tuberculosis and other similar models of intracellular infections.

**Abstract:**

The study of host-pathogen interactions using in vivo models with intracellular pathogens like Mycobacterium tuberculosis (Mtb) entails technical limitations, such as: (i) Selecting an efficient differential lysis system to enrich the pathogen cells; (ii) obtaining sufficient high-quality RNA; and (iii) achieving an efficient rRNA depletion. Thus, some authors had used flow cytometers to separate infected cells or significantly increase the sequencing depth of host–pathogen RNA libraries to observe the pathogens’ gene expression. However, these options carry additional expenses in specialized equipment typically not available for all laboratories. Here, we propose an experimental protocol involving differential cell lysis and a probe-based ribosomal depletion to determine the gene expression of Mtb and its host during in vivo infection. This method increased the number of observed pathogen-expressed genes from 13 using the traditional RNA-seq approach to 702. After eliminating rRNA reads, we observed that 61.59% of Mtb sequences represented 702 genes, while 38.41% represented intergenic regions. Some of the most expressed genes codified for IS1081 (Rv2512c) transposase and eight PE-PGRS members, such as PGRS49 and PGRS50. As expected, a critical percent of the expressed genes codified for secreted proteins essential for infection, such as PE68, lppN, and LpqH. Moreover, three Mtb ncRNAs were highly expressed (small RNA MTS2823, transfer-messenger RNA RF00023, and ribozyme RF00010). Many of the host-expressed genes were related to the inflammation process and the expression of surfactant proteins such as the Sftpa and Sftpc, known to bind Mtb to alveolar macrophages and mi638, a microRNA with no previous associations with pulmonary diseases. The main objective of this study is to present the method, and a general catalog of the Mtb expressed genes at one point of the in vivo infection. We believe our method represents a different approach to the existing ones to study host–pathogen interactions in tuberculosis and other similar intracellular infections, without the necessity of specialized equipment.

## 1. Introduction

RNA-seq approaches have helped to define the finely regulated host–pathogen interactions. However, its application for in vivo models of intracellular bacteria such as *M. tuberculosis* (Mtb) entails several limitations: (i) Selecting an efficient cell lysis system [1]; (ii) obtaining sufficient high-quality RNA [1,2]; and (iii) achieving an efficient rRNA depletion [1,2]. Hence, some studies have used infected cell cultures [3,4], labeled bacteria to separate infected from non-infected cells [1,5], increased the number of infecting bacilli to obtain enough RNA [6], or selectively amplified bacterial RNA/cDNA [7]. However, these strategies simplify the myriad factors involved in an in vivo model or modify the gene expression profile by altering the typical course of infection.

Currently, two studies describe the Mtb transcriptome during in vivo murine infection. The first [6] used DNA microarrays to compare the in vivo gene expression in BALB/c vs. BALB/c ^SCID/SCID^ vs. bacterial cultures at different time points of infection. The second [5] compared the bacteria transcriptome of Mtb Erdman marked with fluorescent reporter mCherry between alveolar and interstitial macrophages isolated directly from infected mouse lungs at day 14 post-infection using RNA-seq. 

Undoubtedly, the use of RNA-seq offers a more sensitive and quantitative approach to determine the expression of genes and intergenic regions. However, the use of specialized equipment to separate infected from non-infected cells or significantly increasing the sequencing depth to enrich the information on the pathogens’ gene expression entails additional expenses not available for all research laboratories worldwide. Overall, the purpose of this study is to present the efficiency and reproducibility of our experimental method to increase the number of observed pathogen expressed genes during tuberculosis in vivo infection. This method could be used in the future to explore the pathogen gene expression profile at different time points of the infection and use different Mtb-infecting strains.

Here, we used a well-characterized murine model for pulmonary tuberculosis [8,9] to obtain the Mtb and mouse transcriptome from lungs 21-days post-infection using an experimental approach involving differential cell lysis and a probe-based ribosomal depletion. 

This murine model is based on the intratracheal instillation of live Mtb bacilli into male BALB/c mice and shows the evolution of the disease in two clear phases, an acute phase that spans from day 1–28 post-infection, characterized by inflammatory infiltrate and formation of granulomas, and an advance phase, from day 28 onwards, characterized by pneumonia, focal necrosis, and fibrosis [9]. Furthermore, we used male mice because of the essential differences in their immune response compared to female mice [10]. Female mice show a hyper-inflammatory response and better protection against tuberculosis, while male mice show anti-inflammatory responses probably favored by testosterone, and are more susceptible to infection. 

## 2. Materials and Methods

### 2.1. Bacteria Cultures

The *M. tuberculosis* (Mtb) H37Rv strain was cultured in Middlebrook 7H9 broth (Millipore, Burlington, MA, USA, Cat. M0178) enriched with ADC Growth Supplement (Millipore, Burlington, MA, USA, Cat. M0553) at 37 °C. The optical density (OD) was monitored weekly, and the purity was assessed with a Zihel-Neelsen stain. As soon as the culture reached the mid-logarithmic phase (OD = 0.6), bacilli were harvested and aliquoted, adjusting to 2.5 × 10^5^ colony-forming units (CFU) in 100 µL of phosphate-buffered saline (PBS) and kept at −80 °C until use. Before inoculation, the frozen stock was thawed, diluted, and sonicated to disperse clumps. 

### 2.2. Mice Infection

The experimental model of pulmonary tuberculosis used in this study has been previously described [8,9,11]. Briefly, pathogen-free male BALB/c mice of 6–8 weeks of age were anesthetized with sevoflurane (Sevorane^®^) (Abbvie, IL, EUA) and inoculated intratracheally using a feeding needle with 2.5 × 10^5^ CFU of the H37Rv Mtb strain. 

Twenty-one days post-infection, mice were euthanized, and both lungs were extracted aseptically, snap-frozen in liquid nitrogen, and kept at −80 °C until use. All the infection and lung extraction procedures were performed in a Class 3 biological safety cabinet.

### 2.3. M. tuberculosis Transcriptome

To analyze the gene expression for the pathogen and the host, we used both lungs of each mouse to obtain the bacteria and mouse RNA. We performed three strategies to enrich the mycobacterial RNA in the infected lungs and obtain the Mtb transcriptome. Two strategies were implemented during the RNA extraction, and the third, after the sequencing library construction.

### 2.4. I. M. tuberculosis Transcriptome

RNA extraction. The infected left side lungs were assigned randomly to each strategy, one lung to Strategy 1, and three lungs to Strategies 2 and 3.

(i) RNA extracted without differential lysis and centrifugation (Strategy 1): We pulverized one infected left lung using sterile mortars and pestles frozen with liquid nitrogen and placed the homogenate in a microfuge tube. Then, total RNA was extracted directly using the Quick RNA miniprep Kit (Zymo Research, Irvine, CA, USA; Cat.R1055) following the manufacturer’s recommendations, including the digestion with DNAseI to remove the contaminating DNA. Finally, we quantified and assessed the quality of the resulting RNA by Qubit Fluorometer (Invitrogen, Waltham, MA, USA, Cat. Q32851) and Agilent 2100 Bioanalyzer (Agilent Technologies, Santa Clara, CA, USA, Cat. 5067-4626), respectively.
(ii) RNA extracted with differential lysis and centrifugation (Strategy 2): We pulverized six infected left lungs independently using sterile mortars and pestles frozen with liquid nitrogen and placed each homogenate in microfuge tubes. Then, we added RLT buffer (Qiagen, Hilden, Germany, Cat.79216) with β mercaptoethanol as the initial lysis buffer to each tube and centrifuged at 14,000 rpm/4 °C for 5 min. We discarded the supernatant and kept the cream-colored pellet on ice. This procedure allowed the enrichment of bacterial cells in the pellet due to the mild-lysis produced by the RLT, which breaks most mouse cells keeping the bacterial cells intact due to its thicker wall membrane. Immediately after centrifugation, we continued the RNA extraction from the pellet using the Quick RNA miniprep Kit (Zymo Research, Irvine, CA, USA; Cat.R1055) following the manufacturer’s recommendations, including the digestion with DNAseI to remove the contaminating DNA. Finally, we quantified and assessed the quality of the resulting RNA by Qubit Fluorometer and Agilent 2100 Bioanalyzer, respectively.

Construction of sequencing libraries. The resulting RNA extracted from each lung with Strategies 1 and 2 was treated independently with the Ribo-Zero rRNA Epidemiology Removal Kit (Illumina, San Diego, CA, USA; Cat.MRZE706) following the manufacturers’ recommendations. Next, 700 µg of depleted RNA was used as input for the NEBNext Ultra RNA Library Prep Kit for Illumina (New England BioLabs, Ipswich, MA, USA; Cat.E7530S), adjusting the Size Select conditions for insert sizes between 400 and 600 bp and the enrichment PCR to 15 cycles. The quantity and quality of all libraries were assessed by Qubit Fluorometer and Agilent 2100 bioanalyzer, respectively. Finally, the library from Strategy 1 and three libraries from strategy 2 were sequenced directly in a NextSeq 500 Mid Output cell in a 150-cycle paired-end format at the National Institute of Genomic Medicine (INMEGEN) in Mexico City, Mexico. The remaining three libraries from Strategy 2 were further treated with the in-house ribosomal subtractive hybridization.

In House Ribosomal Substractive hybridization (Strategy 3).Three independent libraries constructed with RNA from Strategy 2 were hybridized with in-house ribosomal probes to reduce Mtb rRNA sequences. Briefly, we amplified the rRNA from the Mtb genome and sheared the amplicons on a Covaris instrument to an average size of 100–300 bp. The resulting fragments were processed with the NEBNext Fast DNA Library Prep Set for Ion Torrent (New England BioLabs, Ipswich, MA, USA; Cat.E6270S), further amplified with biotinylated primers and purified with AMPure XP beads (Beckman-Coulter, Pasadena, CA, USA; Cat.A63880). 

Next, each library was hybridized with the in-house ribosomal probes for 72 hrs using a temperature ramp from 95 °C to 65 °C. After hybridization, we used magnetic streptavidin-coated beads (Dyabeads MyOne Streptavidin C1; Invitrogen, Waltham, CA, USA; Cat.65001) and washing buffers at a range temperature of 65–99 °C to gradually pull down the biotinylated probes and separate the captured rRNA. Finally, the non-captured fraction of the libraries was quantified by Qubit Fluorometer, and the quality was assessed by Agilent 2100 bioanalyzer. The non-captured fraction of the libraries were sequenced in a NextSeq 500 Mid Output cell in a 150-cycle paired-end format at the INMEGEN in Mexico City, Mexico.

### 2.5. Mouse Transcriptome

For the host transcriptome, we used the right lung of the same three mice used in Strategy 3 for the Mtb transcriptome.

RNA extraction. We pulverized each of the three lungs using a sterile mortar and pestle frozen with liquid nitrogen. Following the manufacturer’s recommendations, the resulting homogenate was processed with the Quick RNA miniprep kit (Zymo Research, Irvine, CA, USA; Cat.R1055), including digestion with DNAseI to remove the contaminating DNA. After the extraction, we quantified and assessed the RNA quality by Qubit Fluorometer and Agilent 2100 bioanalyzer, respectively.

Construction of RNA sequencing libraries.Total RNA from each lung was treated with the NEBNext Poly(A) mRNA Magnetic Isolation Module (New England BioLabs, Ipswich, MA, USA; Cat.E7490) following the manufacturer’s recommendations. Then, 700 µg of the isolated mRNA was used as input for the NEBNext Ultra RNA Library Prep Kit for Illumina (New England BioLabs, Ipswich, MA, USA; Cat.E7530S), adjusting the Size Select conditions for insert sizes between 400 and 600 bp and the enrichment PCR to 15 cycles. After the procedure, we quantified and assessed each library’s quality by Qubit Fluorometer and Agilent 2100 bioanalyzer, respectively. The final libraries were sequenced in a NextSeq500 Mid Output cell in a 300-cycle paired-end format at the INMEGEN in Mexico City, Mexico. 

### 2.6. Sequencing and Assembly of the M. tuberculosis Infectious Strain

We extracted the genomic DNA from a previously harvested aliquot of the infecting H37Rv Mtb strain using the Quick-DNA Fecal/Soil Microbe Miniprep Kit (Zymo Research, Irvine, CA, USA; Cat.D6010) following the manufacturer’s recommendations. The quantity and quality of the resulting DNA were determined using agarose gel electrophoresis and Qubit fluorometer, respectively. 

The sequencing library was constructed with the Nextera XT DNA Library Preparation Kit (Illumina, San Diego, CA, USA; Cat.FC-131-1024) following the manufacturer’s recommendations and selecting an insert size of 400–600 bp. The final library was quantified with the Qubit fluorometer, and the size distribution was analyzed with Agilent 2100 bioanalyzer. This library was sequenced with the MiSeq Output cell in a 500 cycle paired-end format at the INMEGEN in Mexico City, Mexico. 

**Figure 1:**
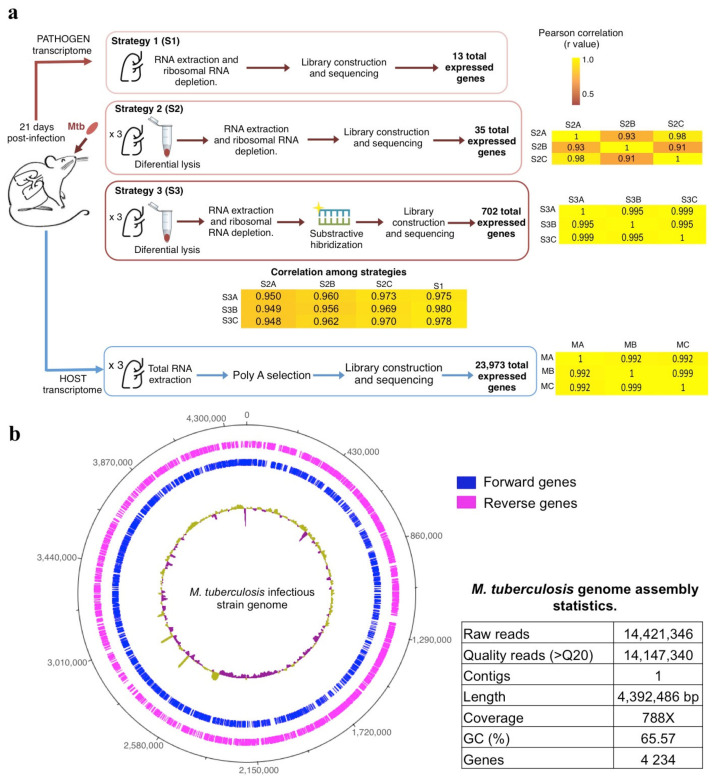
(**a**) Experimental strategies to obtain the Mtb (S1, S2, and S3) and mouse (M) transcriptome profiles. Pearson correlations between three biological replicates (A, B, and C) and strategies (S) are shown in yellow heat maps. (**b**) Assembled genome of the *M. tuberculosis* infectious strain. Regions with depth coverage lower and above average (788×) are shown as purple and yellow inner circles, respectively. In addition, the statistic parameters of the assembly were reported in the table.

The sequencing run produced 14,421,346 total paired reads, 98.1% of which remained after quality filters (≥Q20) and adaptor removal with FASTX-toolkit (v0.0.13) (http://hannonlab.cshl.edu/fastx_toolkit/index.html, accessed on 28 June 2021), and Trimmomatic (v0.36) (http://www.usadellab.org/cms/index.php?page=trimmomatic released 2014, accessed on 28 June 2021). Then, we used SPADES (v.3.13.9) (https://github.com/ablab/spades, accessed on 28 June 2021) and MeDuSa (http://combo.dbe.unifi.it/medusa, accessed on 28 June 2021) to build the de novo assembly. This resulted in 1 contig with 4,392,486 bp and average sequence depth coverage of 788× (Figure 1b).

We used Glimmer (v3.02) [12] within Blast2Go (v5.2) [13] with the Prokaryotic Gene Finding feature to locate 4234 genes and determined a homolog for 99.24% of them analyzing against the non-redundant (nr) database with BLASTX, setting the E-value cut-off at 1.0 × 10^−3^. The transcripts identified as members of the PPE-PGRS family by blast were further searched in the Mycobrowser database [14] to have a more specific description of the PPE-PGRS name. Additionally, all genes were associated with protein families through InterProScan, and functionally mapped to GO terms setting the following parameters: E-value-hi-filer: 1.0 × 10^−3^; annotation cut-off: 55 and GO weight: 5. Finally, the non-coding RNA regions present in the assembled Mtb genome were determined with Infernal (v1.1.3) [15] comparing against the Rfam 14.1 database. The resulting coordinates of the identified regions were added to the gff file with all the annotations.

### 2.7. Analysis of Host and Pathogen RNA-Seq Data 

#### 2.7.1. Analysis of *M. tuberculosis* Transcriptome

Pair-end reads were checked for quality > 20 Q with FASTX-toolkit (v0.0.13), (http://hannonlab.cshl.edu/fastx_toolkit/index.html, accessed on 28 June 2021) and adaptors were trimmed using Trimmomatic (v0.36) (http://www.usadellab.org/cms/index.php?page=trimmomatic, accessed on 28 June 2021). Next, as Avraham et al. 2016 [1] suggested, we built a composed database to analyze the mixed host–pathogen reads and minimize spurious read alignments. This database contained the mouse and Mtb rRNA sequences, mouse reference genome GRCm38.p6 (GenBank GCA_000001635.8), and the de novo assembly of the Mtb infectious genome. All read mappings were performed using SMALT (v0.7.6) (https://github.com/rcallahan/smalt, accessed on 28 June 2021), adjusting strict parameters to avoid cross-mapping reads that will affect transcript quantification. We only considered as positive reads that mapped with a minimum coverage of ≥80%.

The reads mapped to rRNA and mouse sequences were counted and separated using the Samtools suite (v1.3.1) (https://sourceforge.net/projects/samtools/files/samtools/, accessed on 28 June 2021). Finally, to determine the number of reads mapped to Mtb genes and non-coding regions, we intersected the aligned bam file with the corresponding gff file containing all the annotations using Bedtools (v2.26.0) (https://github.com/arq5x/bedtools2/releases, accessed on 28 June 2021). The gene count of each library was normalized by reads per kilobase per million mapped reads (RPKM), and Pearson rank correlations between replicates and between control and experimental libraries were calculated with the GraphPad Prism7 (GraphPad software, San Diego, CA, USA).

Further, we used Blast2Go (BioBam Bioinformatics, Valencia, Spain) to perform an InterPro and gene ontology (GO) enrichment analysis using Fisher’s exact test, considering a significant *p*-value of <0.05 and the complete Mtb assembled genome as a reference. We used the KEGG database with a bi-directional best-hit method (BBH) and *Mycobacterium tuberculosis* as the reference gene set, then determined the percentage of each pathway represented by our set of expressed genes. 

To analyze the presence of expressed genes corresponding to secreted proteins, we aligned the sequences of the 529 most expressed genes against the published secretome of *M. tuberculosis* [16], considering as positive an E-value < 0.001 and coverage > 70%. Additionally, we constructed 100 groups of 529 genes randomly selected from the 4234 total Mtb genes. Then, we compared each group with the Mtb secretome and determined the number of groups resulting in more than 85 proteins aligned with an E-value < 0.001 and >70% coverage.

Finally, to analyze if the distribution of the 12 genes from the Top 20 list in the 1138 kb region was a random event, we first divided our Mtb assembled genome into four sections, each accounting for ~25% of the total length. Next, we created a hundred groups of 20 random genes selected from the 702 observed expressed genes and observed their location. Lastly, for each group, we counted the number of genes located within the same region of the genome and considered a positive event if there were 12 or more genes located together.

#### 2.7.2. Analysis of Mouse Transcriptome

Pair-end reads were checked for quality > 20 Q with FASTX-toolkit (v0.0.13) (http://hannonlab.cshl.edu/fastx_toolkit/index.html, accessed on 28 June 2021), and adaptors were trimmed using Trim_Galore (v0.4.2) (https://github.com/FelixKrueger/TrimGalore, accessed on 28 June 2021). Next, filtered reads were aligned against the *Mus musculus* NCBI reference genome GRCm38.p6 (GenBank GCA_000001635.8) using Bowtie2 (v2.3.5) (http://bowtie-bio.sourceforge.net/bowtie2/index.shtml, accessed on 28 June 2021) with default parameters. Next, the raw count expression profiles were obtained using HTSeq (version 0.6.1) (https://htseq.readthedocs.io/en/master/history.html#version-0-6-1, accessed on 28 June 2021), then, gene count was normalized by RPKM. 

We used the gene ontology (GO) database to analyze enriched GO terms in the Biological Process, Molecular Function, and Cellular Component categories using a Fisher’s exact test, a significant *p*-Value of <0.05, and *Mus musculus* as the reference genome. Additionally, we used the KEGG database to analyze the most abundant KEGG pathways with a bi-directional best-hit method (BBH) and *Mus musculus* as the reference gene set.

## 3. Results and Discussion

### 3.1. The Differential Cell Lysis Protocol Concentrated the Number of Mycobacterial Cells and Increased the Extracted Bacterial RNA

As a first approach, we sequenced the total RNA extracted from one infected lung (Figure 1a) to explore the expression obtained following the typical RNA-seq strategy (Strategy 1, S1). Next, we analyzed the sequencing data mapping against a multifasta file containing the mouse reference genome, Mtb rRNA sequences, and the Mtb strain genome used for the infection (see materials and methods) (Figure 1b). The analysis showed that only 1.70% of sequences belonged to Mtb, representing 13 expressed genes (Table S1). 

**Figure 2:**
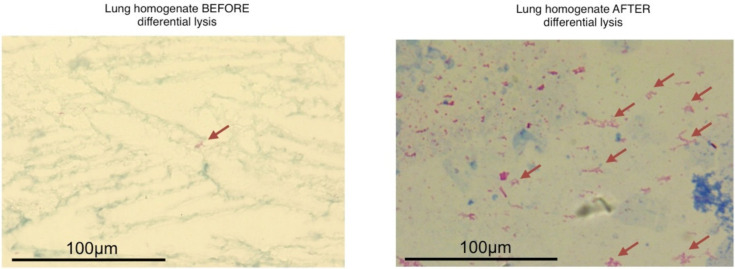
Zihel-Neelsen staining of the infected lung homogenate before and after differential cellular lysis. The red arrow shows the bacilli per field.

Thus, we standardized a differential cell lysis protocol to concentrate the number of mycobacterial cells before the RNA extraction (Strategy 2, S2) (Figure 1a). In this case, the Zihel-Neelsen staining showed a bacterial cell concentration (Figure 2), and the sequences from three biological replicates confirmed a ~4% increase in the proportion of Mtb sequences compared to S1 (Table S1). 

However, despite using a ribosomal depletion kit, ~97% of the Mtb sequences still belonged to ribosomal transcripts, and the remaining sequences represented the expression of 35 genes. Some studies have reported that commercial kits remove 70–85% of ribosomal RNA in pure mycobacterial cultures [17]. However, our samples derived from infected tissue, and the mix of host and mycobacteria cells might have reduced the depletion efficiency. 

**Figure 3:**
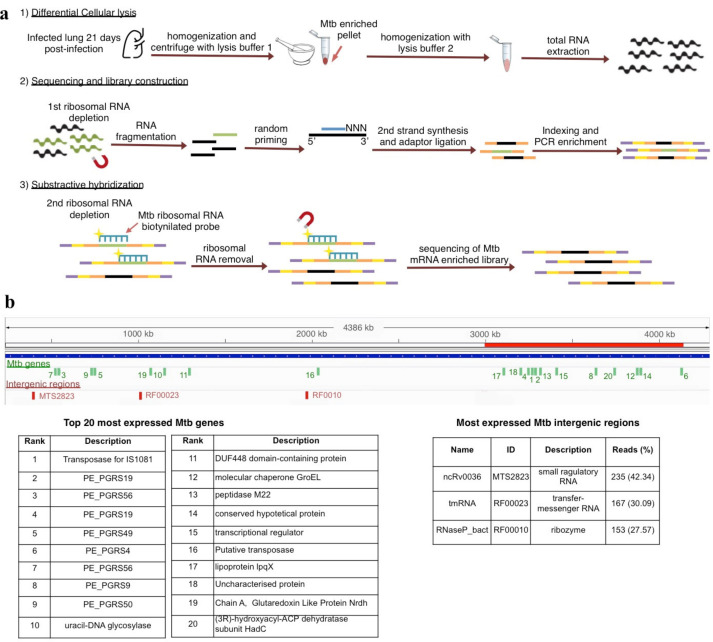
Final strategy to obtain Mtb gene expression profile and genome location of the twenty most expressed genes. (**a**) Detailed experimental procedure used to obtain the transcriptome profile in Strategy 3. (**b**) Genomic location of the Top 20 most expressed Mtb genes (green vertical lines) and the three most expressed intergenic regions (red vertical lines) across the Mtb-sequenced genome. The red horizontal line indicates the 1138 kb region of the Mtb genome (from 3,005,716 bp to 4,144,624 bp) that concentrated twelve of the top 20 most expressed genes. The tables indicate each expressed gene ranked from the most to the least expressed and the percentage of reads mapped to each intergenic region.

To reduce the amount of ribosomal RNA, we designed biotinylated probes to selectively subtract the ribosomal transcripts from the total RNA of three independent infected lungs (Strategy 3, S3) (Figure 1a and Figure 3a) (see materials and methods). This method successfully decreased 24.56% of Mtb ribosomal transcripts, allowing the observation of 702 expressed genes (Table S2), a ~50 fold enrichment in the gene number compared to S1. Additionally, Pearson correlations of the gene expression abundance between strategies showed no bias due to the differential lysis and probe hybridization methods (r > 0.9) as well as good reproducibility among samples (Figure 1a).

### 3.2. The MTb Gene Expression Profile Showed a High Abundance of Genes Codifiyng PE-PGRS Members and the Insertion Sequence IS1081

We used the data obtained with Strategy 3 (S3) to describe the gene expression profile of Mtb during the in vivo infection. Interestingly, the data showed that 61.59% of the non-ribosomal Mtb sequences represented 702 genes (Table S2), while 38.41% represented 30 intergenic regions. To avoid analyzing transitory transcripts, we only considered 529 genes with ≥2 reads in at least one sample and an average RPKM ≥ 1 for further analysis (Table S2).

First, we determined the InterPro and Gene Ontology (GO) annotations significantly (*p* < 0.05) enriched in the 529 most expressed genes as compared to the complete Mtb genome. The InterPro analysis showed a significant (*p* < 0.05) enrichment of eight protein domains related to fatty acid metabolism and two protein families representing type VII secretion system subunits (Table 1 and Table S3). 

As expected, the GO analysis showed a significant (*p* < 0.05) enrichment of terms related to lymphocyte and T-cell co-stimulation, T-cell receptors, nitrate reductase, zymogen binding, and scavenger response (Table 2 and Table S4). Accordingly, the KEGG pathway analyses of the 529 most expressed genes showed fatty acid metabolism, secretion, T-cell and lymphocyte co-stimulation, glyoxylate and dicarboxylate metabolism, fatty acid synthesis, secretion systems, and quorum sensing as some of the pathways covered ≥20% by the expressed genes (Table 3 and Table S5).

The InterPro, GO, and KEGG analysis showed an enrichment of terms related to energy production and macromolecules synthesis. This profile was expected and is consistent with the exponential growth phase of the bacteria during this infection stage [8,9]. Moreover, the abundant presence of pathways for glyoxylate metabolism indicated that the mycobacteria are adapting to a stressful environment [4] using lipids as a carbon source [18]. Additionally, the expression of genes related to T-cell stimulation and cell recruitment is consistent with the day post-infection we are analyzing (day 21), where the granuloma formation occurs [8,9]. 

The most over-expressed Mtb gene was the transposase for IS1081 (Rv2512c) (Table 4), an insertion sequence used as a molecular marker for *M. bovis* and Mtb but with no previous reports of expression during Mtb in vivo infections. In contrast, the insertion sequence IS6110, besides being considered the standard molecular marker for Mtb strains [19], has also been proposed as a promoter that can affect mycobacterial fitness given its location within the genome of some strains [19]. Some authors suggest it can affect the bacterial gene expression by causing a frameshift that affects the transcription or producing an RNA pseudoknot, which interferes with translation [20]. Additionally, increased transposase activity for IS6110 was observed in mice infected with Mtb H37Rv and in bacterial liquid cultures with nutrient deficiency [20]. These results suggest that the transposition of IS6110 may respond to stress conditions such as the one produced during an in vivo infection. However, the IS6110 transposase showed a low expression ranking under 300 of the 529 expressed genes in our data. 

Notably, we observed that the genome of Mtb H37Rv has 33 copies for the IS6110 transposase while the IS1081 transposase has only 6, suggesting that the high expression of the transposase for IS1081 we observed was not due to a higher gene copy number. Thus, suggesting the importance of considering the role of IS1081 during the development of Mtb in different environments.

The following eight most expressed genes belonged to the PE-PGRS family (Table 4), considered important Mtb antigens that are in close contact with the host immune system [21]. Particularly, PGRS49 and PGRS50 have been proposed as strong vaccine candidates [22] due to their strong antigenicity. On the other hand, PE-PGRS9 and PE-PGRS53 had been previously related to chronic Mtb infections [21,23]. Interestingly, only one previous in vivo study [6] reported the expression of PE-PGRS members, but not as the primarily expressed genes. Thus, to the best of our knowledge, this is the first RNA-seq study to highlight the role of some PE-PGRS transcripts during the early stages of tuberculosis in vivo infection. In line with the high expression of PE-PGRS, we also observed an increased expression of secretion system subunits necessary to export them, especially ESX-1, ESX-3, and ESX-5 [24]. 

Interestingly, 12 of the 20 most expressed genes were located within a 1138 kb region, representing 25.95% of the genome (Figure 3b and Table S2). To test if this observation was coincidental, we formed 100 groups of 20 random genes selected from the complete set of 702 expressed genes observed and determined their location. Notably, neither group showed more than nine genes within the same region (*p* = 0.00). 

In a previous study, Talaat [6] defined a 34.1 kb region concentrating 20 significantly expressed genes during in vivo infection, and interestingly, our 1138 kb region contained five of those 20 genes. These results suggest that the transcription of this section of the genome is essential for the host–pathogen interaction. 

### 3.3. An Increased Proportion of Expressed Genes Belong to the MTb Secretome

We compared how many of the 529 expressed genes corresponded to the Mtb secretome previously reported [16]. According to the previous publication [16], we expected that only ~12% (63 genes) belonged to the secretome, but we observed that 16.07% (85 genes) codified for secreted proteins (Table S6). These suggested a slight overabundance of the genes for secreted proteins in the transcriptome. To test if this observation was coincidental, we formed 100 groups of 529 random genes selected from 4234 total Mtb genes and compared their presence in the secretome. As a result, 41 from the 100 groups had more than 85 genes aligned with the secretome (E-value < 0.001 and >70% coverage). These results suggest that the over-abundance of genes for secreted proteins had a low probability of being a random event. 

Among the expressed transcripts for secreted proteins, we observed seven lipoproteins, such as lppN (Rv2270) and LpqH (Rv3763), which are considered important for the host–pathogen interaction (Table S6). Some studies suggest they help internalize the bacteria into the macrophages [25]. Particularly LpqH has shown some effects that favor the host, such as increasing IL-12, while other effects favor the pathogen, such as inhibiting INF γ, and decreasing the expression of MCH-II and antigen processing [26,27,28]. In the study by Pisu [5], they detect some lipoproteins responsible for the degradation of triglycerides and cholesterol overexpressed in infected alveolar macrophages, such as Lpl and LipA. However, they did not detect overexpression of LpqH or lppN, probably because these proteins respond to different stimuli in other cell types.

On the other hand, 28.24% (24 genes) of the 85 expressed transcripts for secreted proteins were members of the PE-PGRS family (Table S6). Four of them were some of the most expressed genes (PGRS-49, PGRS56, PGRS9 y PGRS50) (Table 4). We also observed PE68, a gene codified in the region of difference 1 (RD1), which is absent in non-virulent Mtb strains such as BCG [29]. PE68 is considered an efficient immunomodulator that stimulates the host immune cellular response, even promoting tissue damage. Interestingly, although PE68 is not considered essential for the survival of Mtb, some studies suggest that alterations in its sequence affect strain virulence [29]. This transcript ranked in the 25th place of the 529 expressed genes. 

### 3.4. A High Expression of Three ncRNAs during Mtb In Vivo Infection

From the whole Mtb sequences, 38.41% mapped to 30 intergenic regions. Previous reports showed that the expression of intergenic regions in liquid cultures during exponential growth is around 28%, while during the stationary phase, their expression increased to 58% [30]. These results suggest that the expression of intergenic regions varies during the development of Mtb in different environments. 

Among the intergenic regions, we identified three ncRNAs, namely small regulatory RNA MTS2823 (ncRv0036) (42.34% of sequences), the transfer-messenger RNA RF00023 (30.09%), and the ribozyme RF0010 (27.57%) (Figure 3b). 

There is scarce information about the role of ncRNAs during the Mtb infection. Mainly, MTS2823 is considered necessary for Mtb growth under different environments, and its expression is especially accumulated in stationary phase cultures [30], mouse lungs with chronic infections [30], and even in dormant mycobacteria [31]. Furthermore, previous reports suggest that MTS2823 may mediate the down-regulation of genes expressed in the exponential growth phase [30]. Although the mechanisms remain to be clarified, more in vivo studies are needed to determine if MTS2823 down-regulates genes during different stages of infection. 

Our results suggest that MTS2823 also plays an essential role during the early stages of in vivo infection. Probably, this expression increases in later stages, favoring the maintenance of the chronic infection. Thus, the exciting idea is to favor its expression earlier to control the spread of infection. Contrary, sRNAs induced in the stationary phase such as MRS0997 and MTS1338 were not detected in our dataset [30].

### 3.5. The Host Gene Expression Showed an Inflammatory Profile Consistent with the Early Stage of Infection

Finally, we sequenced the host RNA from the same infected mice, and after assessing good biological reproducibility (Pearson r = 0.9946) (Figure 1a), we selected 15,677 genes with ≥10 reads in two samples for further analysis (Table S7). 

The GO analysis considering the top 5% most expressed genes (Table S8) showed inflammation, antigen processing and presentation, cellular response to lipopolysaccharides, and bacterial evasion activities as some of the most significantly (*p* < 0.05) enriched terms. Accordingly, the KEGG pathway analysis showed antigen processing and presentation, phagolysosome, IL-17, and TNF-α signaling, and Toll receptors activities as some of the pathways represented in ≥10% (Table S9). 

In the top 20 most expressed genes, we observed transcripts for two surfactant-associated proteins (Sftpc and Sftpa), three histocompatibility genes, and microRNA 638. Notably, Sftpa is known to act as an opsonin to enhance the ingestion of Mtb and other pulmonary pathogens by alveolar macrophages [32,33,34]. However, interestingly, this internalization pathway has also been shown to decrease reactive nitrogen intermediates levels, suggesting binding of Sftpa may be one mechanism by which Mtb reduced the macrophage cytotoxic response [34,35]. 

The dominant immune cellular response in the early infection has been previously described for this murine model (8), consistent with previous studies [36]. In contrast, the expression of genes related to T-cell and lymphocyte co-stimulation and antigen processing is typically associated with chronic infections [37] but maybe involved with granuloma formation and maintenance in early stages [38]. 

Interestingly, we observed microRNA mi6381 as one of the twenty most expressed genes. This microRNA has no previous reports associating it to tuberculosis or any other pulmonary disease. On the other hand, several in vitro studies suggest that micro RNAs such as mir155, mir135b, or mir146a play essential roles in mycobacterial infections [39,40,41,42,43]. Mainly mir155 was found overexpressed in mononuclear cells of tuberculosis patients than of healthy individuals [44]. However, we did not observe this miRNA in our list of expressed genes, suggesting that different miRNAs may be active in different infected tissues.

### 3.6. Comparisson of Mtb Gene Expression Using Other RNA-Seq Methods during In Vivo Infections

**Figure 4:**
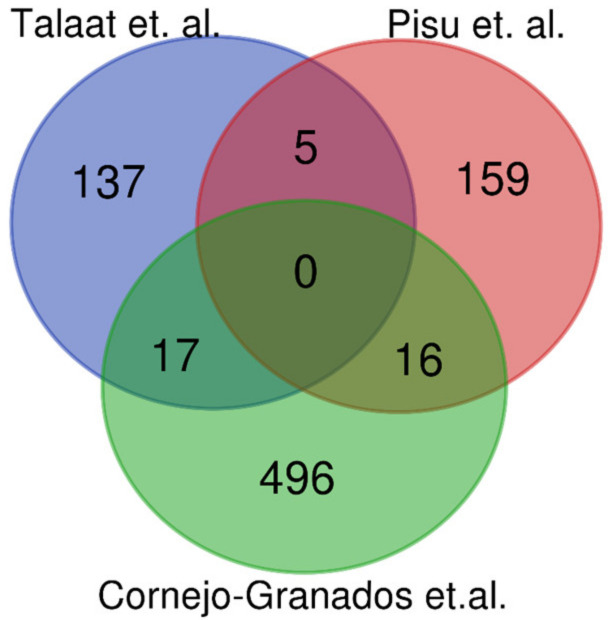
Venn diagram comparing the expressed genes between Talaat et al. [6], Pisu et al. [5], and Cornejo-Granados in vivo studies.

As mentioned before, to the best of our knowledge, two studies aim to describe the Mtb gene expression during an in vivo infection. Both studies have essential methodological similarities and differences with the present study, which we highlight in Table 5. Thus, it is essential to note that discrepancies in the observed expressed genes among studies could arise from the methodological and biological differences such as the murine model, the mycobacterial strain, or the criteria established by each group to consider genes as expressed for the analysis. In this sense, the study by Pisu et al. can be considered the most related to our work; however, it carries critical methodological differences (Table 5), which make the gene expression results not directly comparable between methods. Despite that, we compared our results with Pisu [5], who used RNA-seq to establish an “in vivo signature” of 180 genes expressed in macrophages, and Talaat [6], who used microarrays to determined 159 genes significantly expressed in in vivo conditions. We observed that only 16 and 17 of our expressed genes were observed in Pisu’s and Talaat’s studies, respectively (Figure 4), while five genes were shared by Talaat vs. Pisu. Thus, this suggested that the observed Mtb gene expression among the three studies depends on the different experimental methods and biological variants, such as the murine model and the bacterial strain used.

## 4. Conclusions

The experimental method using the differential cell lysis and a probe-based ribosomal depletion presented here allowed the observation of the most expressed genes and intergenic regions of Mtb directly from an in vivo infection, increasing the number of expressed genes observed, from 13 with the traditional approach to 702 using our experimental method.

We acknowledge that the number of genes analyzed could be broadened, increasing the sequencing depth. However, the main objective at this point was to demonstrate the technical efficacy and reproducibility of our experimental method. Additionally, it is important to mention that our analysis only assessed gene expression levels, from which we can only draw limited conclusions. We are conducting studies using this method and using different Mtb strains, allowing us to understand biological questions using the key value of RNA-seq in a comparative analysis.

The present study revealed the presence of highly expressed MTS2823, a non-coding RNA. Additionally, we observed mi638, a microRNA with no previous reports associating it to tuberculosis or any other pulmonary disease. The analysis we present is a general catalog of the gene expression at one point during the in vivo tuberculosis infection, and it does not intend to draw critical conclusions regarding the pathogen’s physiology. In order to better understand the relevance of the expressed genes we observed, it is necessary to analyze and compare additional time points of the disease. In this regard, it is probable that our catalog of expressed genes only reflects the typical cross-talk during the evolution of the host–pathogen interaction. In this sense, our research group has ongoing experiments using this experimental approach to analyze and compare the gene expression during earlier and later stages of the infection and with Mtb strains from different genotypes. 

Additionally, it is essential to note that this protocol is not the only experimental approach available for using RNA-seq in an in vivo infection model. As mentioned before, the study by Pisu et al. also uses RNA-seq to analyze the gene expression profile of infected macrophages isolated directly from a murine lung. Definitively, individual cell analysis seams an optimal approach for studying any infectious disease. However, this methodology entails additional expenses in specialized reagents and equipment not typically available for all laboratories. The advantage of our method is that it does not require specialized equipment to conduct a comparative analysis directly from an in vivo infection. Thus, we believe this approach is an efficient and reproducible method that is an alternative for the general scientific community to explore the in vivo gene expression of diverse Tb strains and clinical isolates with prevalence worldwide.

## Figures and Tables

**Table 1:** InterPro domains and families significantly overrepresented in the 529 most expressed *M. tuberculosis* genes.

InterPro ID	Category	Description	*p*-Value
IPR014043	domain	Acyl transferase	6.267 × 10^−7^
IPR009081	domain	Phosphopantetheine binding ACP domain	1.438 × 10^−6^
IPR014030	domain	Beta-ketoacyl synthase, N-terminal	2.747 × 10^−5^
IPR014031	domain	Beta-ketoacyl synthase, C-terminal	4.885 × 10^−5^
IPR013968	domain	Polyketide synthase, ketoreductase domain	1.624 × 10^−4^
IPR023836	family	EccCa-like, Actinobacteria	1.087 × 10^−3^
IPR023837	family	EccCb-like, Actinobacteria	1.087 × 10^−3^
IPR032821	domain	Ketoacyl-synthetase, C-terminal extension	1.190 × 10^−3^
IPR006091	domain	Acyl-CoA oxidase/dehydrogenase, central domain	1.363 × 10^−3^
IPR020807	domain	Polyketide synthase, dehydratase domain	1.757 × 10^−3^
IPR009075	domain	Acyl-CoA dehydrogenase/oxidase C-terminal	1.995 × 10^−3^
IPR002543	domain	FtsK domain	6.180 × 10^−3^
IPR003029	domain	S1 domain	7.039 × 10^−3^
IPR003495	domain	CobW/HypB/UreG, nucleotide-binding domain	7.039 × 10^−3^
IPR023753	domain	FAD/NAD(P)	1.360 × 10^-2^
IPR000788	domain	Ribonucleotide reductase large subunit, C-terminal	1.558 × 10^−2^
IPR001030	domain	Aconitase/3-isopropylmalate dehydratase large subunit, alpha/beta/alpha domain	1.558 × 10^−2^
IPR002300	domain	Aminoacyl-tRNA synthetase, class Ia	1.558 × 10^−2^
IPR003714	domain	PhoH-like protein	1.558 × 10^−2^
IPR004100	domain	ATPase, F1/V1/A1 complex, alpha/beta subunit, N-terminal domain	1.558 × 10^−2^

**Table 2:** Gene Ontology terms significantly enriched by category related to the 529 most expressed *M. tuberculosis* gene separated by category.

GO ID	GO Name	*p*−Value
**A. Biological Process**		
GO:0031295	T-cell co-stimulation	1.765 × 10^−5^
GO:0031294	Lymphocyte co-stimulation	1.765 × 10^−5^
GO:0009081	Branched-chain amino acid metabolic process	4.007 × 10^−5^
GO:0009083	Branched-chain amino acid catabolic process	1.102 × 10^−4^
GO:0006549	Isoleucine metabolic process	1.197 × 10^−4^
GO:0000288	Nuclear-transcribed mRNA catabolic process, Deadenylation-dependent decay	2.663 × 10^−4^
GO:0006573	Valine metabolic process	3.513 × 10^−4^
GO:0060184	Cell cycle switching	4.878 × 10^−4^
GO:0051728	Cell cycle switching, mitotic to meiotic cell cycle	4.878 × 10^−4^
GO:0006574	Valine catabolic process	5.108 × 10^−4^
**B. Cellular Component**		
GO:0005643	Nuclear pore	4.20 × 10^−3^
GO:0000932	P-body	5.39 × 10^−3^
GO:0009986	Cell surface	6.00 × 10^−3^
GO:0098978	Glutamatergic synapse	6.18 × 10^−3^
GO:1990527	Tec1p-Ste12p-Dig1p complex	7.04 × 10^−3^
GO:0110165	Cellular anatomical entity	1.23 × 10^−2^
GO:1990526	Ste12p-Dig1p-Dig2p complex	1.60 × 10^−2^
GO:0030496	Midbody	1.81 × 10^−2^
GO:0009325	Nitrate reductase complex	1.83 × 10^−2^
GO:0016020	Membrane	2.56 × 10^−2^
**C. Molecular Function**		
GO:0004085	Butyryl-CoA dehydrogenase activity	1.62 × 10^−4^
GO:0005488	Binding	6.96 × 10^−4^
GO:0052890	Oxidoreductase activity, acting on the CH-CH group of donors, with a flavin as acceptor	7.83 × 10^−4^
GO:0043168	Anion binding	1.16 × 10^−3^
GO:0003955	NAD(P)H dehydrogenase (quinone) activity	1.94 × 10^−3^
GO:0017056	Structural constituent of nuclear pore	2.87 × 10^−3^
GO:0004962	Endothelin receptor activity	2.94 × 10^−3^
GO:0036094	Small molecule binding	3.15 × 10^−3^
GO:1901363	Heterocyclic compound binding	4.09 × 10^−3^
GO:0097159	Organic cyclic compound binding	4.10 × 10^−3^

**Table 3:** KEGG pathways overrepresented by the 529 most expressed *M. tuberculosis* genes.

KEGG Id	Pathway	% of the Pathway Represented by the Expressed Genes
03020	RNA polymerase	75.00
00630	Glyoxylate and dicarboxylate metabolism	34.15
03018	RNA degradation	33.33
00562	Inositol phosphate metabolism	33.33
00910	Nitrogen metabolism	31.82
05152	Tuberculosis	30.77
00680	Methane metabolism	28.57
00290	Valine, leucine and isoleucine biosynthesis	28.57
00430	Taurine and hypotaurine metabolism	28.57
00020	Citrate cycle (TCA cycle)	28.13
00730	Thiamine metabolism	27.27
00983	Drug metabolism—other enzymes	27.27
00061	Fatty acid biosynthesis	26.67
02024	Quorum sensing	25.00
00010	Glycolysis/Gluconeogenesis	24.24
00270	Cysteine and methionine metabolism	24.24
00260	Glycine, serine, and threonine metabolism	24.00
03060	Protein export	23.53
01053	Biosynthesis of siderophore group nonribosomal peptides	22.22
03070	Bacterial secretion system	21.43

**Table 4:** Top 20 most expressed *M. tuberculosis* genes at day 21 post-infection.

Expression Ranking	Gen ID	Gene Description	Mean RPKM
1	Rv2512c	Transposase for insertion sequence element IS1081	6.84 × 10^5^
2	Rv1067c	PE_PGRS19 PE-PGR	3.45 × 10^5^
3	Rv3512	PE_PGRS56 PE-PGRS	1.91 × 10^5^
4	Rv1067c	PE_PGRS19 PE-PGR	1.65 × 10^5^
5	Rv3344c	PE_PGRS49 PE-PGR	1.63E × 10^5^
6	Rv0279c	PE_PGRS4 PE-PGRS	1.10E × 10^5^
7	Rv3512	PE_PGRS56 PE-PGRS	5.01 × 10^4^
8	Rv0746	PE_PGRS9 PE-PGRS	4.35 × 10^4^
9	Rv3345c	PE_PGRS50 PE-PGR	1.94 × 10^4^
10	Rv0105c	uracil-DNA glycosylase	7.61 × 10^3^
11	Rv2840c	DUF448 domain-containing protein	6.94 × 10^3^
12	Rv0440	molecular chaperone GroEL	5.71 × 10^3^
13	Rv3620c	peptidase M22	4.88 × 10^3^
14	Rv0454	conserved hypotetical protein	4.58 × 10^3^
15	Rv0967	transcriptional regulator	4.12 × 10^3^
16	Rv2424c	Putative transposase	4.05 × 10^3^
17	Rv1228	lipoprotein lpqX	3.66 × 10^3^
18	Rv2424c	Uncharacterized protein	3.11 × 10^3^
19	Rv3053c	Chain A, Glutaredoxin Like Protein Nrdh	3.09 × 10^3^
20	Rv0637	(3R)-hydroxyacyl-ACP dehydratase subunit HadC	2.96 × 10^3^

**Table 5:** Main characteristics of previous studies describing *M. tuberculosis* in vivo gene expression.

	Talaat et al., 2004 [6]	Pisu et al., 2020 [5]	Cornejo-Granados et al., 2021
Mtb strain	H37Rv	Erdman ATCC 35801 mCherry	H37Rv
Day post-infection analyzed	7, 14, 21 and 28	14	21
Murine model used	BALB/c and BALB/c ^SCID/SCID^	C57BL/6J WT	BALB/c
Route of infection	Intranasal	Intranasal	Intratracheal
Infected tissue analyzed	Complete infected lungs	Alveolar and interstitial macrophages isolated from infected lungs	Complete infected lungs
RNA extraction method	Total RNA extraction with Tri Reagent (Trizol) from groups of 50 mice	Isolated cells were treated with Trizol and centrifuged to pellet mycobacterial cells. ~80% of the Trizol (containing host RNA) was removed. Finally, total RNA was extracted with fresh Trizol, and a proportion of the host RNA was added back.	Used a differential centrifugation of individual lungs with a mild-lysis buffer to pellet mycobacterial cells. Plus, removing the supernatant containing host RNA followed by total RNA extraction from the mycobacterial cells with a commercial kit.
Ribosomal RNA elimination	-	Ribo-Zero Gold rRNA Removal Kit (Epidemiology)	Ribo-Zero Gold rRNA Removal Kit (Epidemiology) and In-house Mtb ribosomal probes
Methodology for gene expression analysis	DNA Microarrays with oligonucleotides representing Mtb coding sequences.	RNA-seq	RNA-seq
Key insights	-The expression profile of Mtb in SCID mice is most similar to the profile when grown in broth. -Around 49 genes were only expressed in vivo, and 20 of these are contiguous in a delimited area of the Mtb genome.	-In vivo signature of 180 genes upregulated in macrophages. -Transcriptional signatures varied with macrophage ontology. -Alveolar macrophages showed a distinct up regulation of genes for the acquisition and use of fatty acids. -Interstitial macrophages showed an up regulation of genes for iron sequestration.	-About 62.59% of non-ribosomal sequences represented 702 genes, while 38.41% represented intergenic regions. -The transposase for IS1081 was the most expressed gene. -Eight genes for PE-PGRS members are among the most expressed genes.-Three highly expressed ncRNAs (MTS2823, RF00023, and RF00010)

## Data Availability

The RNA-seq data and the *M. tuberculosis* genome used in this study have been deposited in NCBI under BioProject number PRJNA669742. All additional results are available in the Supplementary Materials.

## Supplementary Materials

The following are available online at https://www.mdpi.com/article/10.3390/biology10090848/s1. Table S1: Read count of the three strategies for the *M. tuberculosis* transcriptome. Table S2: *M. tuberculosis* genes expressed 21 days post-infection observed with Strategy 3. Table S3: InterPro domains and families significantly overrepresented in the 529 most expressed *M. tuberculosis* genes. Table S4: Gene Ontology terms significantly enriched by category related to the 529 most expressed *M. tuberculosis* gene. Table S5: KEGG pathways overrepresented by the 529 most expressed *M. tuberculosis* genes. Table S6: *M. tuberculosis* expressed genes belonging to the predicted secretome. Table S7: 15,677 most expressed mouse genes at day 21 post-infection with *M. tuberculosis*. Table S8: Gene Ontology terms significantly enriched by category related to the most expressed mouse genes. Table S9: KEGG pathways overrepresented by the most expressed mouse genes.
