# Supplementary material for: Targeted RNA-Seq Reveals the M. tuberculosis Transcriptome from an In Vivo Infection Model
Source: Biology (Basel). 2021 Aug 31;10(9):848. doi: 10.3390/biology10090848 (PMC8467220; doi:10.3390/biology10090848)
Supplement: Supplementary file 1 [file biology-10-00848-s001.zip › TableS1_r1.pdf]

Table S1. Read count of the three strategies for the M. tuberculosis transcriptome.

		Raw reads	Clean reads	Reads mapped to the mouse genome	Reads mapped to the Mtb genome	Reads mapped to Mtb ribosomal transcripts
STRATEGY 1	Direct RNA extraction	7,672,944	7,585,764	4,599,634	12,185,398	124,446
STRATEGY 2	Differential cellular lysis	14,339,158	14,222,581	6,696,869	20,919,450	835,125
		17,385,954	17,281,608	7,894,465	25,176,073	1,026,435
		11,610,750	11,525,470	5,556,047	17,081,517	594,224
STRATEGY 3	Differential cellular lysis + ribosomal probe hybridization	63,536,942	62,791,012	55,673,290	118,464,302	58,099
		40,357,826	39,855,911	34,996,425	74,852,336	55,234
		46,740,872	46,156,549	41,138,380	87,294,929	21,996
