# Supplementary material for: Targeted RNA-Seq Reveals the M. tuberculosis Transcriptome from an In Vivo Infection Model
Source: Biology (Basel). 2021 Aug 31;10(9):848. doi: 10.3390/biology10090848 (PMC8467220; doi:10.3390/biology10090848)
Supplement: Supplementary file 1 [file biology-10-00848-s001.zip › TableS2_r1.pdf]

Table S2. M. tuberculosis genes expressed 21 days post-infection observed with Strategy 3
***Genes within the 1,138kb region in the M. tuberculosis genome.

Expression Ranking	Expression Ranking in the list of 529 genes	Gene name	Gen ID	Gene description	Mean RPKM
1	1***	Scaffold_1_orf04709	Rv2512c	Transposase for insertion sequence element IS1081	6.84E+05
2	2***	Scaffold_1_orf04711	Rv1067c	PE_PGRS19 PE-PGR	3.45E+05
3	3	Scaffold_1_orf00755	Rv3512	PE_PGRS56 PE-PGRS	1.91E+05
4	4***	Scaffold_1_orf04705	Rv1067c	PE_PGRS19 PE-PGR	1.65E+05
5	5	Scaffold_1_orf01047	Rv3344c	PE_PGRS49 PE-PGR	1.63E+05
6	6***	Scaffold_1_orf05988	Rv0279c	PE_PGRS4 PE-PGRS	1.10E+05
7	7	Scaffold_1_orf00760	Rv3512	PE_PGRS56 PE-PGRS	5.01E+04
8	8***	Scaffold_1_orf05233	Rv0746	PE_PGRS9 PE-PGRS	4.35E+04
9	9	Scaffold_1_orf01045	Rv3345c	PE_PGRS50 PE-PGR	1.94E+04
10	10	Scaffold_1_orf01629	Rv0105c	uracil-DNA glycosylase	7.61E+03
11	11	Scaffold_1_orf01792	Rv2840c	DUF448 domain-containing protein	6.94E+03
12	12***	Scaffold_1_orf05667	Rv0440	molecular chaperone GroEL	5.71E+03
13	13***	Scaffold_1_orf04766	Rv3620c	peptidase M22	4.88E+03
14	14***	Scaffold_1_orf05644	Rv0454	conserved hypotetical protein	4.58E+03
15	15***	Scaffold_1_orf04905	Rv0967	transcriptional regulator	4.12E+03
16	16	Scaffold_1_orf02899	Rv2424c	Putative transposase	4.05E+03
17	17***	Scaffold_1_orf04432	Rv1228	lipoprotein lpqX	3.66E+03
18	-	Scaffold_1_orf02074	Rv2706c	Uncharacterised protein	3.17E+03
19	-	Scaffold_1_orf02463	Rv2451	hypothetical protein	3.17E+03
20	18***	Scaffold_1_orf04567	Rv2424c	Uncharacterised protein	3.11E+03
21	19	Scaffold_1_orf01508	Rv3053c	Chain A, Glutaredoxin Like Protein Nrdh	3.09E+03
22	20***	Scaffold_1_orf05411	Rv0637	(3R)-hydroxyacyl-ACP dehydratase subunit HadC	2.96E+03
23	21	Scaffold_1_orf03271	Rv1962A	antitoxin VapB35	2.72E+03
24	22	Scaffold_1_orf05692	Rv0420c	membrane protein	2.27E+03
25	23	Scaffold_1_orf00204	Rv3873	PPE68 PPE FAMILY	2.02E+03
26	24	Scaffold_1_orf04443	Rv1221	RNA polymerase sigma factor SigE	1.97E+03
27	25	Scaffold_1_orf00877	Rv3437	conserved transmembrane protein	1.84E+03
28	26	Scaffold_1_orf02274	Rv2576c	membrane protein	1.83E+03
29	27	Scaffold_1_orf00919	Rv3412	Uncharacterised protein	1.80E+03
30	28	Scaffold_1_orf03455	Rv1846c	transcriptional regulator	1.78E+03
31	29	Scaffold_1_orf03175	Rv2011c	MarR family transcriptional regulator	1.72E+03
32	-	Scaffold_1_orf03493	Rv1819c	Uncharacterised protein	1.65E+03
33	30	Scaffold_1_orf06032	Rv0251c	Hsp20/alpha crystallin family protein	1.63E+03
34	31	Scaffold_1_orf00031	Rv0056	50S ribosomal protein L9	1.62E+03
35	32	Scaffold_1_orf04289	Rv1322A	methylmalonyl-CoA epimerase	1.62E+03
36	33	Scaffold_1_orf03409	Rv1876	bacterioferritin	1.61E+03
37	34	Scaffold_1_orf00594	Rv3615c	type VII secretion system ESX-1 filament-forming target	1.57E+03
38	35	Scaffold_1_orf05795	Rv0351	nucleotide exchange factor GrpE	1.57E+03
39	-	Scaffold_1_orf04765	Rv3619c	esat-6 like protein esxO	1.56E+03
40	36	Scaffold_1_orf00907	Rv3420c	ribosomal-protein-alanine acetyltransferase rimI	1.55E+03
41	37	Scaffold_1_orf05257	Rv0733	Chain A,Adenylate Kinase Complexed With Two Molecu	1.50E+03
42	38	Scaffold_1_orf02771	Rv2270	lipoprotein lppN	1.46E+03
43	39	Scaffold_1_orf01943	Rv2784c	lipoprotein LppU	1.44E+03
44	-	Scaffold_1_orf04759	Rv1197	esat-6 like protein esxM	1.44E+03
45	-	Scaffold_1_orf02480	Rv2441c	50S ribosomal protein L27	1.42E+03
46	40	Scaffold_1_orf00102	Rv0009	peptidyl-prolyl cis-trans isomerase	1.40E+03
47	41	Scaffold_1_orf05889	Rv0287	type VII secretion protein EsxS	1.37E+03
48	42	Scaffold_1_orf02479	Rv2442c	50S ribosomal protein L21	1.30E+03
49	43	Scaffold_1_orf00595	Rv3614c	ESX-1 secretion system protein	1.28E+03
50	44	Scaffold_1_orf05473	Rv0605	Uncharacterised protein	1.27E+03
51	45	Scaffold_1_orf05794	Rv0352	molecular chaperone DnaJ	1.25E+03
52	46	Scaffold_1_orf03959	Rv1735c	Uncharacterized membrane protein MT1776	1.25E+03
53	47	Scaffold_1_orf04032	Rv1691	TPR-repeat-containing protein	1.23E+03
54	48	Scaffold_1_orf05766	Rv0369c	putative oxidoreductase	1.23E+03
55	49	Scaffold_1_orf05735	Rv0391	O-succinylhomoserine sulfhydrylase	1.21E+03
56	50	Scaffold_1_orf02444	Rv2466c	conserved protein	1.19E+03
57	51	Scaffold_1_orf05691	Rv0421c	alpha/beta hydrolase	1.18E+03
58	52	Scaffold_1_orf06146	Rv0175	MCE-associated membrane protein	1.16E+03
59	53	Scaffold_1_orf01617	Rv2986c	hydrolase MUTT1	1.15E+03
60	54	Scaffold_1_orf04832	Rv1015c	50S ribosomal protein L25	1.14E+03
61	55	Scaffold_1_orf05306	Rv0701	50S ribosomal protein L3 rplC	1.13E+03
62	57	Scaffold_1_orf05303	Rv0702	50S ribosomal protein L4	1.10E+03
63	56	Scaffold_1_orf02205	Rv2621c	putative transcriptional regulatory protein	1.10E+03
64	58	Scaffold_1_orf02109	Rv2680	PF11452 family protein	1.08E+03
65	59	Scaffold_1_orf02927	Rv2159c	carboxymuconolactone decarboxylase family protein	1.07E+03
66	60	Scaffold_1_orf05797	Rv0350	fusion protein	1.04E+03
67	61	Scaffold_1_orf02048	Rv2725c	GTPase HflX	1.04E+03
68	62	Scaffold_1_orf06038	Rv0247c	succinate dehydrogenase/fumarate reductase iron-sulfur	1.02E+03
69	63	Scaffold_1_orf04687	Rv1078	RDD family protein	1.00E+03
70	64	Scaffold_1_orf05456	Rv0614	galactose-1-phosphate uridylyltransferase	9.89E+02
71	65	Scaffold_1_orf05253	Rv0736	Conserved membrane protein of uncharacterised functio	9.85E+02
72	66	Scaffold_1_orf00330	Rv3791	decaprenylphosphoryl-D-2-keto erythropentose reductas	9.69E+02
73	67	Scaffold_1_orf04038	Rv1687c	ABC transporter ATP-binding protein	9.66E+02
74	68	Scaffold_1_orf06281	Rv0091	5'-methylthioadenosine/S-adenosylhomocysteine nucleo	9.66E+02
75	-	Scaffold_1_orf02567	Rv2389c	Resuscitation-promoting factor RpfD	9.43E+02
76	69	Scaffold_1_orf00509	Rv3674c	ultraviolet N-glycosylase/AP lyase	9.40E+02
77	70	Scaffold_1_orf02081	Rv2702	polyphosphate glucokinase	9.29E+02
78	71	Scaffold_1_orf06109	Rv0199	Conserved membrane protein of uncharacterised functio	9.22E+02
79	-	Scaffold_1_orf03272	Rv1962c	PIN domain-containing protein	9.09E+02
80	72	Scaffold_1_orf00620	Rv3594	Phage endolysin	8.96E+02
81	73	Scaffold_1_orf05689	Rv0422c	phosphomethylpyrimidine kinase thiD	8.96E+02
82	74	Scaffold_1_orf02083	Rv2701c	extragenic suppressor protein SUHB	8.89E+02
83	75	Scaffold_1_orf03136	Rv2033c	DUF3097 domain-containing protein	8.80E+02
84	76	Scaffold_1_orf01606	Rv2993c	2-hydroxyhepta-22C4-diene-1-2C7-dioate isomerase	8.77E+02
85	77	Scaffold_1_orf03767	Rv1472	enoyl-CoA hydratase	8.64E+02
86	78	Scaffold_1_orf01773	Rv2890c	30S ribosomal protein S2	8.58E+02
87	-	Scaffold_1_orf02913	Rv2166c	division/cell wall cluster transcriptional repressor MraZ	8.58E+02
88	79	Scaffold_1_orf00646	Rv3577	putative Zn-dependent hydrolases	8.55E+02
89	80	Scaffold_1_orf05956	Rv0534c	1,4-dihydroxy-2-naphthoate polyprenyltransferase	8.44E+02
90	81	Scaffold_1_orf06090	Rv0211	Conserved membrane protein of uncharacterised functio	8.43E+02
91	82	Scaffold_1_orf05123	Rv0830	SAM-dependent methyltransferase	8.19E+02
92	83	Scaffold_1_orf05901	Rv0281	SAM-dependent methyltransferase	8.16E+02

93	84	Scaffold_1_orf01539	Rv3035	PQQ enzyme repeat-containing protein	8.15E+02
94	85	Scaffold_1_orf00596	Rv3610c	ATP-dependent zinc metalloprotease FtsH	8.12E+02
95	86	Scaffold_1_orf00474	Rv3695	RDD family protein	8.09E+02
96	87	Scaffold_1_orf02347	Rv2528c	restriction system protein mrr	8.05E+02
97	88	Scaffold_1_orf04026	Rv1695	NAD(+) kinase	8.03E+02
98	89	Scaffold_1_orf04535	Rv1161	nitrate reductase subunit alpha	8.02E+02
99	90	Scaffold_1_orf01590	Rv3003c	acetolactate synthase large subunit IlvB1	7.99E+02
100	91	Scaffold_1_orf05484	Rv0600c	sensor histidine kinase	7.96E+02
101	92	Scaffold_1_orf03393	Rv1886c	esterase, putative, antigen 85-B	7.95E+02
102	93	Scaffold_1_orf01251	Rv3218	diacylglycerol kinase	7.75E+02
103	94	Scaffold_1_orf05563	Rv0502	phospholipid/glycerol acyltransferase	7.72E+02
104	96	Scaffold_1_orf06037	Rv0248c	fumarate reductase/succinate dehydrogenase flavoprote	7.64E+02
105	95	Scaffold_1_orf01124	Rv3299c	arylsulfatase AtsB	7.64E+02
106	97	Scaffold_1_orf00592	Rv3616c	type VII secretion system ESX-1 target EspA	7.63E+02
107	98	Scaffold_1_orf01924	Rv2795c	putative transposase	7.61E+02
108	99	Scaffold_1_orf02073	Rv2707	YihY/virulence factor BrkB family protein	7.61E+02
109	100	Scaffold_1_orf04623	Rv1110	lytB-related protein lytB2	7.36E+02
110	101	Scaffold_1_orf05148	Rv0799c	conserved protein	7.36E+02
111	102	Scaffold_1_orf05059	Rv0867c	molybdenum cofactor biosynthesis protein E2	7.30E+02
112	103	Scaffold_1_orf03749	Rv1485	ferrochelatase	7.16E+02
113	104	Scaffold_1_orf02656	Rv2338c	molybdopterin biosynthesis protein moeW	7.14E+02
114	-	Scaffold_1_orf03369	Rv1899c	lipoprotein LppD	7.10E+02
115	105	Scaffold_1_orf03421	Rv1868	UDP-glucose 4-epimerase GalE4	7.06E+02
116	106	Scaffold_1_orf03307	Rv1940	riboflavin biosynthesis protein RibA	6.98E+02
117	107	Scaffold_1_orf02065	Rv2712c	Uncharacterised protein	6.91E+02
118	108	Scaffold_1_orf06086	Rv0215c	acyl-CoA dehydrogenase	6.90E+02
119	109	Scaffold_1_orf04333	Rv1295	threonine synthase	6.85E+02
120	110	Scaffold_1_orf05622	Rv0467	isocitrate lyase	6.79E+02
121	111	Scaffold_1_orf04667	Rv1087	PE_PGRS21 PE-PGRS	6.74E+02
122	112	Scaffold_1_orf00047	Rv0046c	myo-inositol-1-phosphate synthase	6.72E+02
123	-	Scaffold_1_orf03681	Rv1531	carboxymuconolactone decarboxylase family protein	6.72E+02
124	-	Scaffold_1_orf04381	Rv1265	hypothetical protein	6.68E+02
125	113	Scaffold_1_orf05218	Rv0752c	acyl-CoA dehydrogenase FadE9	6.66E+02
126	114	Scaffold_1_orf02334	Rv2535c	aminopeptidase P family protein	6.63E+02
127	-	Scaffold_1_orf00134	Rv3911	RNA polymerase sigma factor SigM	6.61E+02
128	115	Scaffold_1_orf02139	Rv2659c	integrase	6.57E+02
129	-	Scaffold_1_orf00597	Rv3609c	GTP cyclohydrolase I FolE	6.54E+02
130	116	Scaffold_1_orf02854	Rv2212	adenylate cyclase	6.52E+02
131	117	Scaffold_1_orf02677	Rv2328	PE23 PE family	6.45E+02
132	118	Scaffold_1_orf04429	Rv1229c	sodium:proton antiporter	6.45E+02
133	119	Scaffold_1_orf02362	Rv2518c	lipoprotein LppS	6.40E+02
134	121	Scaffold_1_orf02394	Rv2495c	branched-chain alpha-ketoacid dehydrogenase complex	6.38E+02
135	122	Scaffold_1_orf04588	Rv1131	Chain A, Crystal Structure Of Methylcitrate Synthase Fro	6.38E+02
136	120	Scaffold_1_orf03416	Rv1872c	L-lactate dehydrogenase	6.38E+02
137	123	Scaffold_1_orf04457	Rv1212c	glycogen synthase	6.37E+02
138	124	Scaffold_1_orf05503	Rv0589	virulence factor Mce family protein	6.34E+02
139	125	Scaffold_1_orf03548	Rv1785c	cytochrome P450	6.27E+02
140	126	Scaffold_1_orf05586	Rv0490	two-component sensor histidine kinase	6.25E+02
141	127	Scaffold_1_orf06258	Rv0106	Probable cobalamin synthesis protein	6.20E+02
142	128	Scaffold_1_orf04134	Rv1627c	lipid-transfer protein	6.13E+02
143	129	Scaffold_1_orf00129	Rv3915	putative N-acetylmuramoyl-L-alanine amidase	6.07E+02
144	130	Scaffold_1_orf00040	Rv0050	penicillin-binding protein	6.02E+02
145	131	Scaffold_1_orf00894	Rv3428c	IS21 family transposase	6.01E+02
146	132	Scaffold_1_orf02801	Rv2246	3-oxoacyl-[acyl-carrier-protein] synthase 2 KasB	5.91E+02
147	134	Scaffold_1_orf01140	Rv3290c	L-lysine 6-transaminase	5.79E+02
148	133	Scaffold_1_orf05886	Rv0290	type VII secretion system ESX-3 subunit EccD3	5.79E+02
149	135	Scaffold_1_orf04846	Rv1005c	para-aminobenzoate synthase component I pabD	5.76E+02
150	136	Scaffold_1_orf01798	Rv2836c	MATE family efflux transporter	5.66E+02
151	137	Scaffold_1_orf00190	Rv3881c	type VII secretion system ESX-1 target EspB	5.65E+02
152	138	Scaffold_1_orf03628	Rv1568	adenosylmethionine-8-amino-7-oxononanoate aminotrar	5.64E+02
153	139	Scaffold_1_orf05530	Rv0576	transcriptional regulator	5.64E+02
154	140	Scaffold_1_orf00487	Rv3685c	cytochrome P450	5.44E+02
155	-	Scaffold_1_orf01628	Rv2976c	uracil-DNA glycosylase	5.42E+02
156	141	Scaffold_1_orf04156	Rv1614	prolipoprotein diacylglyceryl transferase lgt	5.40E+02
157	142	Scaffold_1_orf00189	Rv3882c	type VII secretion system ESX-1 subunit EccE1	5.34E+02
158	143	Scaffold_1_orf03551	Rv1782	type VII secretion system ESX-5 subunit EccB5	5.33E+02
159	144	Scaffold_1_orf03997	Rv1713	ribosome biogenesis GTPase Der	5.33E+02
160	145	Scaffold_1_orf05554	Rv0509	glutamyl-tRNA reductase	5.27E+02
161	146	Scaffold_1_orf00858	Rv3450c	conserved membrane protein	5.25E+02
162	147	Scaffold_1_orf02064	Rv2713	soluble pyridine nucleotide transhydrogenase	5.24E+02
163	148	Scaffold_1_orf04589	Rv1130	MmgE/PrpD family protein	5.23E+02
164	149	Scaffold_1_orf01615	Rv2988c	3-isopropylmalate dehydratase large subunit	5.21E+02
165	150	Scaffold_1_orf03531	Rv1795	type VII secretion system ESX-5 subunit EccD5	5.17E+02
166	151	Scaffold_1_orf05497	Rv0591	MCE-family protein MCE2C	5.13E+02
167	-	Scaffold_1_orf03429	Rv1863c	CPBP family intramembrane metalloprotease	5.13E+02
168	152	Scaffold_1_orf02930	Rv2157c	UDP-N-acetylmuramoyl-tripeptide--D-alanyl-D-alanine lig	5.10E+02
169	154	Scaffold_1_orf01118	Rv3303c	NAD(P)H dehydrogenase (quinone)	5.00E+02
170	153	Scaffold_1_orf05620	Rv0468	3-hydroxybutyryl-CoA dehydrogenase	5.00E+02
171	-	Scaffold_1_orf05472	Rv0606	IS1536, transposase, truncated	4.98E+02
172	-	Scaffold_1_orf02749	Rv2286c	DSBA-like thioredoxin domain protein	4.92E+02
173	155	Scaffold_1_orf00912	Rv3417c	chaperonin GroEL	4.82E+02
174	156	Scaffold_1_orf04356	Rv1280c	periplasmic oligopeptide-binding lipoprotein oppA	4.77E+02
175	157	Scaffold_1_orf03967	Rv1731	succinic semialdehyde dehydrogenase	4.76E+02
176	158	Scaffold_1_orf02453	Rv2459	integral membrane transport protein	4.72E+02
177	-	Scaffold_1_orf03145	Rv2028c	universal stress protein	4.65E+02
178	159	Scaffold_1_orf06237	Rv0119	acyl-CoA synthetase	4.55E+02
179	160	Scaffold_1_orf01529	Rv3043c	cytochrome ubiquinol oxidase subunit I	4.54E+02
180	161	Scaffold_1_orf03491	Rv1820	acetolactate synthase	4.51E+02
181	162	Scaffold_1_orf04439	Rv1223	serine protease htrA	4.49E+02
182	163	Scaffold_1_orf04410	Rv1243c	PE_PGRS23 PE-PGRS	4.43E+02
183	164	Scaffold_1_orf04533	Rv1162	nitrate reductase subunit beta	4.42E+02
184	165	Scaffold_1_orf04144	Rv1620c	thiol reductant ABC exporter subunit CydC	4.33E+02
185	166	Scaffold_1_orf01868	Rv2853	PE_PGRS48 PE-PGRS	4.23E+02
186	167	Scaffold_1_orf02998	Rv2115c	ATPase AAA	4.23E+02
187	168	Scaffold_1_orf00136	Rv3910	transmembrane protein	4.20E+02
188	-	Scaffold_1_orf00918	Rv3413c	Conserved protein of uncharacterised function 2C alanir	4.12E+02
189	169	Scaffold_1_orf03774	Rv1467c	acyl-CoA dehydrogenase FadE15	4.05E+02
190	170	Scaffold_1_orf01736	Rv2913c	D-amino acid aminohydrolase	4.04E+02
191	172	Scaffold_1_orf04368	Rv1272c	ABC transporter ATP-binding protein	4.02E+02
192	171	Scaffold_1_orf05898	Rv0282	type VII secretion system ESX-3 AAA family ATPase Ecc	4.02E+02
193	173	Scaffold_1_orf03330	Rv1925	nitrate ABC transporter substrate-binding protein	3.98E+02
194	174	Scaffold_1_orf02107	Rv2682c	1-deoxy-D-xylulose-5-phosphate synthase	3.87E+02
195	175	Scaffold_1_orf04380	Rv1266c	putative transmembrane serine/threonine-protein kinase	3.87E+02
196	176	Scaffold_1_orf05524	Rv0578c	PE_PGRS7 PE-PGRS	3.78E+02
197	-	Scaffold_1_orf02869	Rv2200c	COX2/NosJ periplasmic domain protein	3.76E+02
198	-	Scaffold_1_orf00475	Rv3694c	transmembrane protein	3.73E+02
199	177	Scaffold_1_orf04960	Rv0931c	serine/threonine protein kinase	3.72E+02
200	178	Scaffold_1_orf05895	Rv0284	type VII secretion system ESX-3 FtsK/SpoIIIE family ATI	3.71E+02
201	179	Scaffold_1_orf04959	Rv0932c	phosphate-binding protein	3.69E+02
202	-	Scaffold_1_orf00497	Rv3679	anion transporter ATPase	3.69E+02
203	180	Scaffold_1_orf00711	Rv3534c	4-hydroxy-2-oxovalerate aldolase	3.69E+02
204	181	Scaffold_1_orf03356	Rv1908c	catalase/peroxidase HPI	3.68E+02
205	182	Scaffold_1_orf03142	Rv2030c	erythromycin esterase	3.66E+02
206	183	Scaffold_1_orf03880	Rv1398c	antitoxin	3.59E+02
207	-	Scaffold_1_orf01949	Rv2781c	zinc protease	3.58E+02
208	-	Scaffold_1_orf02915	Rv2165c	16S rRNA (cytosine(1402)-N(4))-methyltransferase Rsm	3.58E+02
209	-	Scaffold_1_orf03430	Rv1862	zinc-binding dehydrogenase	3.55E+02
210	184	Scaffold_1_orf01677	Rv2948c	p-hydroxybenzoic acid--AMP ligase FadD22	3.50E+02
211	-	Scaffold_1_orf04030	Rv1692	phosphatase	3.49E+02
212	-	Scaffold_1_orf02960	Rv2139	Dihydroorotate dehydrogenase	3.31E+02
213	-	Scaffold_1_orf04686	Rv1079	cystathionine gamma-synthase	3.18E+02
214	185	Scaffold_1_orf03490	Rv1821	preprotein translocase subunit SecA	3.17E+02
215	-	Scaffold_1_orf02568	Rv2388c	coproporphyrinogen III oxidase	3.16E+02
216	-	Scaffold_1_orf02199	Rv2625c	zinc metalloprotease	3.14E+02
217	186	Scaffold_1_orf04901	Rv0969	metal cation transporter P-type ATPase CtpV	3.12E+02

218	187	Scaffold_1_orf04586	Rv1133c	5-methyltetrahydropteroyltriglutamate-homocysteine me	3.11E+02
219	188	Scaffold_1_orf00139	Rv3909	Conserved protein	3.08E+02
220	-	Scaffold_1_orf03842	Rv1426c	alpha/beta hydrolase	3.05E+02
221	-	Scaffold_1_orf05596	Rv0483	lipoprotein LprQ	2.99E+02
222	-	Scaffold_1_orf00617	Rv3595c	PE_PGRS59 PE-PGR	2.96E+02
223	-	Scaffold_1_orf03568	Rv1771	L-gulono-1,4-lactone dehydrogenase	2.88E+02
224	-	Scaffold_1_orf02645	Rv2344c	deoxyguanosine triphosphate triphosphohydrolase	2.86E+02
225	189	Scaffold_1_orf04504	Rv1183	RND transporter MmpL10	2.80E+02
226	190	Scaffold_1_orf01496	Rv3060c	transcriptional regulator, GntR family	2.79E+02
227	-	Scaffold_1_orf00802	Rv3496c	Mce family protein Mce4D	2.73E+02
228	191	Scaffold_1_orf01296	Rv3193c	Conserved membrane protein of uncharacterised functio	2.64E+02
229	192	Scaffold_1_orf03477	Rv1832	glycine dehydrogenase	2.62E+02
230	-	Scaffold_1_orf03495	Rv1818c	PE_PGRS33 PE-PGR	2.61E+02
231	193	Scaffold_1_orf05718	Rv0402c	RND transporter MmpL1	2.58E+02
232	-	Scaffold_1_orf06290	Rv0086	hydrogenase HycQ	2.57E+02
233	194	Scaffold_1_orf05557	Rv0507	RND transporter MmpL2	2.55E+02
234	-	Scaffold_1_orf05891	Rv0286	PPE4 PPE FAMILY P	2.53E+02
235	195	Scaffold_1_orf03451	Rv1849	urease subunit beta	2.53E+02
236	196	Scaffold_1_orf04074	Rv1664	polyketide synthase	2.49E+02
237	-	Scaffold_1_orf02675	Rv2329c	NarK/NasA family nitrate transporter	2.39E+02
238	197	Scaffold_1_orf00191	Rv3880c	ESX-1 secretion-associated protein EspL	2.33E+02
239	198	Scaffold_1_orf00824	Rv3479	DUF3376 domain-containing protein	2.30E+02
240	-	Scaffold_1_orf00184	Rv3885c	type VII secretion system ESX-2 subunit EccE2	2.30E+02
241	-	Scaffold_1_orf02747	Rv2287	Na+/H+ antiporter	2.28E+02
242	199	Scaffold_1_orf05427	Rv0630c	exonuclease subunit V beta recB	2.26E+02
243	200	Scaffold_1_orf05362	Rv0667	DNA-directed RNA polymerase subunit beta	2.25E+02
244	-	Scaffold_1_orf02564	Rv2391	sulfite reductase	2.22E+02
245	201	Scaffold_1_orf04308	Rv1311	F0F1 ATP synthase subunit epsilon	2.18E+02
246	-	Scaffold_1_orf00714	Rv3533c	PPE62 PPE FAMILY	2.12E+02
247	-	Scaffold_1_orf01675	Rv2950c	long-chain-fatty-acid--AMP ligase FadD29	2.10E+02
248	202	Scaffold_1_orf02968	Rv2134c	DUF3090 domain-containing protein	2.03E+02
249	203	Scaffold_1_orf03550	Rv1784	FtsK/SpoIIIE family protein	1.97E+02
250	204	Scaffold_1_orf05487	Rv0598c	PIN domain-containing protein	1.93E+02
251	205	Scaffold_1_orf03344	Rv1917c	PPE34 PPE FAMILY	1.91E+02
252	206	Scaffold_1_orf01701	Rv2935	phthiocerol type I polyketide synthase PpsE	1.84E+02
253	209	Scaffold_1_orf00730	Rv3527	Uncharacterised protein	1.77E+02
254	208	Scaffold_1_orf00171	Rv3894c	type VII secretion system ESX-2 FtsK/SpoIIIE family ATI	1.77E+02
255	207	Scaffold_1_orf04714	Rv1065	cysteine dioxygenase	1.77E+02
256	210	Scaffold_1_orf00692	Rv3547	deazaflavin-dependent nitroreductase	1.75E+02
257	211	Scaffold_1_orf00910	Rv3418c	molecular chaperone GroES	1.74E+02
258	212	Scaffold_1_orf01540	Rv3034c	acetyltransferase	1.68E+02
259	213	Scaffold_1_orf02354	Rv2524c	DUF1729 domain-containing protein	1.65E+02
260	-	Scaffold_1_orf00210	Rv3870	type VII secretion system ESX-1 FtsK/SpoIIIE family ATI	1.65E+02
261	214	Scaffold_1_orf00416	Rv3733c	NUDIX domain-containing protein	1.59E+02
262	215	Scaffold_1_orf04516	Rv1174c	hemophore-related protein	1.58E+02
263	216	Scaffold_1_orf02401	Rv2490c	Uncharacterised protein	1.55E+02
264	-	Scaffold_1_orf05229	Rv0747	Uncharacterised protein	1.54E+02
265	218	Scaffold_1_orf01681	Rv2946c	polyketide synthase	1.52E+02
266	217	Scaffold_1_orf01860	Rv3728	Truncated hydrogenase nickle incorporation protein	1.52E+02
267	-	Scaffold_1_orf01676	Rv2949c	Uncharacterised protein	1.48E+02
268	219	Scaffold_1_orf04214	Rv2168c	transposase	1.44E+02
269	220	Scaffold_1_orf02085	Rv2699c	DUF4193 domain-containing protein	1.28E+02
270	221	Scaffold_1_orf01827	Rv2876	DUF2631 domain-containing protein	1.27E+02
271	222	Scaffold_1_orf03687	Rv1527c	polyketide synthase	1.20E+02
272	223	Scaffold_1_orf01861	Rv3410c	Truncated hydrogenase nickle incorporation protein	1.20E+02
273	224	Scaffold_1_orf02171	Rv2639c	integral membrane protein	1.20E+02
274	-	Scaffold_1_orf01901	Rv2810c	putative transposase	1.19E+02
275	225	Scaffold_1_orf04507	Rv1181	polyketide synthase	1.18E+02
276	226	Scaffold_1_orf00613	Rv3597c	nucleoid-associated protein	1.18E+02
277	227	Scaffold_1_orf01671	Rv2952	Phthiotriol/phenolphthiotriol dimycocerosates methyltran	1.10E+02
278	228	Scaffold_1_orf04310	Rv1310	F0F1 ATP synthase subunit beta	1.09E+02
279	229	Scaffold_1_orf03079	Rv2067c	Uncharacterised protein	1.08E+02
280	230	Scaffold_1_orf01849	Rv2863	ribonuclease VapC23	1.05E+02
281	231	Scaffold_1_orf04183	Rv1594	quinolinate synthetase	1.03E+02
282	232	Scaffold_1_orf03608	Rv1581c	phage protein	1.01E+02
283	233	Scaffold_1_orf05088	Rv0847	lipoprotein lpqS	9.77E+01
284	234	Scaffold_1_orf01470	Rv3079c	conserved oxidoreductase	9.63E+01
285	235	Scaffold_1_orf01777	Rv2887	MarR family transcriptional regulator	9.49E+01
286	236	Scaffold_1_orf02367	Rv2513	Uncharacterised protein	9.42E+01
287	-	Scaffold_1_orf00022	Rv0062	Uncharacterised protein	9.36E+01
288	237	Scaffold_1_orf05285	Rv0713	transmembrane protein	9.26E+01
289	238	Scaffold_1_orf02485	Rv2437	Isoprenylcysteine carboxyl methyltransferase (ICMT) far	9.10E+01
290	239	Scaffold_1_orf06162	Rv0164	cyclase	9.04E+01
291	240	Scaffold_1_orf00943	Rv3397c	phytoene synthase	8.65E+01
292	241	Scaffold_1_orf02727	Rv2303c	antibiotic resistance protein	8.63E+01
293	242	Scaffold_1_orf05615	Rv0471c	1,4-dihydroxy-2-naphtoate prenyltransferase	8.15E+01
294	243	Scaffold_1_orf03428	Rv1864c	MOSC domain protein	7.91E+01
295	-	Scaffold_1_orf01250	Rv3219	WhiB family transcriptional regulator	7.81E+01
296	-	Scaffold_1_orf01630	Rv2975c	dak phosphatase	7.81E+01
297	244	Scaffold_1_orf05167	Rv0784	putative deacetylase	7.68E+01
298	245	Scaffold_1_orf04366	Rv1274	lipoprotein lprB	7.59E+01
299	246	Scaffold_1_orf01026	Rv3350c	PPE56 PPE FAMILY	7.56E+01
300	247	Scaffold_1_orf02674	Rv2330c	lipoprotein LppP	7.55E+01
301	249	Scaffold_1_orf05202	Rv0762c	nuclear transport factor 2 family protein	7.30E+01
302	-	Scaffold_1_orf03609	Rv1580c	phage protein	7.30E+01
303	248	Scaffold_1_orf00927	Rv3407	type II toxin-antitoxin system Phd/YefM family antitoxin	7.30E+01
304	250	Scaffold_1_orf01852	Rv2861c	type I methionyl aminopeptidase	7.22E+01
305	251	Scaffold_1_orf06164	Rv0163	acyl-CoA thioesterase	7.20E+01
306	252	Scaffold_1_orf02566	Rv2390c	membrane protein	7.14E+01
307	253	Scaffold_1_orf03076	Rv2069	RNA polymerase sigma factor SigC	7.14E+01
308	-	Scaffold_1_orf02961	Rv3870	dihydroorotate dehydrogenase	7.07E+01
309	254	Scaffold_1_orf00669	Rv3562	acyl-CoA dehydrogenase	7.03E+01
310	255	Scaffold_1_orf04791	Rv0818	DNA-binding response regulator	6.87E+01
311	256	Scaffold_1_orf03912	Rv1379	bifunctional pyr operon transcriptional regulator/uracil ph	6.85E+01
312	-	Scaffold_1_orf01703	Rv2934	phthiocerol type I polyketide synthase PpsD	6.76E+01
313	257	Scaffold_1_orf04215	Rv3326	Putative transposase for insertion sequence element IS6	6.76E+01
314	258	Scaffold_1_orf06284	Rv0089	methyltransferase	6.71E+01
315	259	Scaffold_1_orf05607	Rv0475	heparin-binding hemagglutinin	6.64E+01
316	260	Scaffold_1_orf02451	Rv2461c	ATP-dependent Clp protease proteolytic subunit	6.61E+01
317	261	Scaffold_1_orf02541	Rv2405	growth inhibitor PemK	6.61E+01
318	262	Scaffold_1_orf04713	Rv1066	rhodanese domain-containing protein	6.52E+01
319	263	Scaffold_1_orf02556	Rv3555c	Uncharacterised protein	6.52E+01
320	264	Scaffold_1_orf01260	Rv3214	Chain A, Structure Solution To 2 Angstrom And Function	6.51E+01
321	265	Scaffold_1_orf04863	Rv0995	Putative ribosomal-protein-alanine acetyltransferase	6.51E+01
322	266	Scaffold_1_orf00481	Rv3689	hypothetical protein, partial	6.46E+01
323	267	Scaffold_1_orf04171	Rv1602	imidazole glycerol phosphate synthase subunit HisH	6.42E+01
324	268	Scaffold_1_orf00628	Rv3588c	carbonic anhydrase	6.39E+01
325	-	Scaffold_1_orf03610	Rv1579c	phage protein	6.33E+01
326	269	Scaffold_1_orf04695	Rv1072	transmembrane protein	6.30E+01
327	270	Scaffold_1_orf01476	Rv3074	HNH endonuclease	6.25E+01
328	-	Scaffold_1_orf03141	Rv2031c	alpha-crystallin	6.07E+01
329	271	Scaffold_1_orf02319	Rv2544	lipoprotein lppB	6.01E+01
330	272	Scaffold_1_orf01122	Rv3301c	phosphate-specific transport system accessory protein F	5.98E+01
331	273	Scaffold_1_orf03562	Rv1774	FAD-binding oxidoreductase	5.94E+01
332	274	Scaffold_1_orf01048	Rv3344c	PE_PGRS49 PE-PGR	5.88E+01
333	275	Scaffold_1_orf01538	Rv3036c	DUF3298 domain-containing protein	5.83E+01
334	276	Scaffold_1_orf00845	Rv3462c	translation initiation factor IF-1	5.82E+01
335	277	Scaffold_1_orf04881	Rv0981	Response regulator MprA	5.80E+01
336	278	Scaffold_1_orf01782	Rv2846c	integral membrane efflux protein EfpAt	5.64E+01
337	279	Scaffold_1_orf05421	Rv0634c	MBL fold metallo-hydrolase	5.58E+01
338	281	Scaffold_1_orf02863	Rv2205c	glycerate kinase	5.55E+01
339	280	Scaffold_1_orf00370	Rv3764c	two component system sensor kinase	5.55E+01
340	282	Scaffold_1_orf03673	Rv1535	Uncharacterised protein	5.45E+01
341	283	Scaffold_1_orf06096	Rv0207c	NYN domain protein	5.42E+01
342	284	Scaffold_1_orf04531	Rv1164	respiratory nitrate reductase subunit gamma	5.38E+01

343	285	Scaffold_1_orf02893	Rv2182c	1-acyl-sn-glycerol-3-phosphate acyltransferase	5.36E+01
344	286	Scaffold_1_orf02885	Rv2190c	NlpC/P60 family protein	5.34E+01
345	-	Scaffold_1_orf05334	Rv0682	30S ribosomal protein S12	5.31E+01
346	287	Scaffold_1_orf03215	Rv1991A	antitoxin MazE	5.19E+01
347	288	Scaffold_1_orf03263	Rv1965	ABC transporter permease	5.13E+01
348	289	Scaffold_1_orf01378	Rv3134c	universal stress protein	5.11E+01
349	290	Scaffold_1_orf00981	Rv3351c	Putative oxidoreductase	5.01E+01
350	291	Scaffold_1_orf05624	Rv0466	Acyl-ACP thioesterase	5.01E+01
351	292	Scaffold_1_orf03124	Rv2042c	nuclear transport factor 2 (NTF2) domain protein - 798 n	4.99E+01
352	293	Scaffold_1_orf05328	Rv0686	membrane protein	4.99E+01
353	-	Scaffold_1_orf02643	Rv2346c	ESAT-6 like protein EsxO	4.99E+01
354	-	Scaffold_1_orf04760	Rv1042c	IS like-2 transposase	4.99E+01
355	294	Scaffold_1_orf00522	Rv3664c	ABC transporter permease	4.98E+01
356	295	Scaffold_1_orf03994	Rv1714	oxidoreductase	4.90E+01
357	296	Scaffold_1_orf02970	Rv2133c	Phosphatidylinositol 3-and 4-kinase	4.88E+01
358	297	Scaffold_1_orf03993	Rv1716	Kynurenine formamidase	4.80E+01
359	298	Scaffold_1_orf02259	Rv2585c	protein translocase subunit SecF	4.76E+01
360	300	Scaffold_1_orf05165	Rv0785	FAD-binding dehydrogenase	4.74E+01
361	-	Scaffold_1_orf04996	Rv0912	transmembrane protein	4.74E+01
362	299	Scaffold_1_orf03772	Rv1468c	PE_PGRS29 PE-PGR	4.74E+01
363	301	Scaffold_1_orf00197	Rv3878	secretion protein EspJ	4.73E+01
364	302	Scaffold_1_orf01636	Rv2971	Chain A, A Structural Characterization Of The Isoniazid M	4.69E+01
365	303	Scaffold_1_orf01800	Rv2835c	sugar ABC transporter permease	4.60E+01
366	304	Scaffold_1_orf00042	Rv0048c	DUF1707 domain-containing protein	4.58E+01
367	305	Scaffold_1_orf03922	Rv3326	Putative transposase for insertion sequence element IS6	4.50E+01
368	306	Scaffold_1_orf00790	Rv3502c	3-oxoacyl-ACP reductase	4.47E+01
369	307	Scaffold_1_orf01329	Rv3171c	alpha/beta fold family hydrolase	4.43E+01
370	308	Scaffold_1_orf03533	Rv1794	Chain C, Crystal Structure Of Pe8-ppe15 In Complex Wi	4.41E+01
371	309	Scaffold_1_orf04953	Rv0936	phosphate ABC transporter permease	4.40E+01
372	310	Scaffold_1_orf00308	Rv3806c	decaprenyl-phosphate phosphoribosyltransferase	4.38E+01
373	311	Scaffold_1_orf05322	Rv0691c	mycofactocin system transcriptional regulator	4.30E+01
374	312	Scaffold_1_orf00126	Rv3917c	ParB family protein	4.30E+01
375	313	Scaffold_1_orf00200	Rv3876	ESX-1 secretion associated protein EspL	4.30E+01
376	314	Scaffold_1_orf04518	Rv1172c	PE12 PE FAMILY P	4.30E+01
377	315	Scaffold_1_orf03791	Rv1456c	heme A synthase	4.27E+01
378	316	Scaffold_1_orf04478	Rv1047	mutator family transposase	4.24E+01
379	317	Scaffold_1_orf05347	Rv0673	enoyl-CoA hydratase	4.24E+01
380	318	Scaffold_1_orf04840	Rv1010	dimethyladenosine transferase ksgA	4.18E+01
381	319	Scaffold_1_orf02916	Rv2164c	hypothetical protein	4.14E+01
382	320	Scaffold_1_orf04962	Rv0929	phosphate ABC transporter permease subunit PstC	4.09E+01
383	321	Scaffold_1_orf05033	Rv0885	diiron oxygenase	4.05E+01
384	322	Scaffold_1_orf03704	Rv1516c	sugar transferase	4.04E+01
385	323	Scaffold_1_orf04415	Rv1240	Chain A, Structure Of Malate Dehydrogenase	4.03E+01
386	324	Scaffold_1_orf02393	Rv2496c	alpha-ketoacid dehydrogenase subunit beta	4.01E+01
387	325	Scaffold_1_orf04782	Rv0824c	tRNA dihydrouridine synthase DusB	3.92E+01
388	326	Scaffold_1_orf01889	Rv2817c	CRISPR-associated protein cas1	3.88E+01
389	327	Scaffold_1_orf02962	Rv2138	lipoprotein LppL	3.88E+01
390	-	Scaffold_1_orf03541	Rv1792	esat-6 like protein	3.88E+01
391	328	Scaffold_1_orf01511	Rv3051c	class 1b ribonucleoside-diphosphate reductase subunit a	3.83E+01
392	329	Scaffold_1_orf00489	Rv3684	cysteine synthase/cystathionine beta-synthase	3.83E+01
393	330	Scaffold_1_orf00850	Rv3457c	DNA-directed RNA polymerase subunit alpha	3.82E+01
394	331	Scaffold_1_orf02898	Rv2178c	3-deoxy-7-phosphoheptulonate synthase class II	3.80E+01
395	-	Scaffold_1_orf03596	Rv1758	serine esterase, cutinase family	3.80E+01
396	332	Scaffold_1_orf02923	Rv2161c	F420-dependent oxidoreductase	3.79E+01
397	-	Scaffold_1_orf00430	Rv0086	Uncharacterised protein	3.78E+01
398	333	Scaffold_1_orf04690	Rv1076	lipase lipU	3.70E+01
399	334	Scaffold_1_orf06100	Rv0204c	TIGR00374 family protein	3.70E+01
400	335	Scaffold_1_orf00261	Rv3843c	transmembrane protein	3.68E+01
401	336	Scaffold_1_orf03290	Rv1948c	Uncharacterised protein	3.68E+01
402	337	Scaffold_1_orf03348	Rv1915	isocitrate lyase	3.61E+01
403	338	Scaffold_1_orf05132	Rv0811c	folate-binding protein	3.60E+01
404	339	Scaffold_1_orf04654	Rv1945	HNH endonuclease	3.55E+01
405	340	Scaffold_1_orf04247	Rv1346	acyl-[acyl-carrier-protein] dehydrogenase MbtN	3.43E+01
406	-	Scaffold_1_orf01851	Rv2862c	DUF1707 domain-containing protein	3.41E+01
407	-	Scaffold_1_orf03394	Rv1885c	chorismate mutase	3.32E+01
408	341	Scaffold_1_orf05769	Rv0367c	DUF3423 domain-containing protein	3.31E+01
409	342	Scaffold_1_orf01369	Rv3140	acyl-CoA dehydrogenase - 1206 nt	3.30E+01
410	343	Scaffold_1_orf03663	Rv1542c	group 1 truncated hemoglobin	3.29E+01
411	344	Scaffold_1_orf03917	Rv1375	YcaO-like family protein	3.26E+01
412	345	Scaffold_1_orf06179	Rv0154c	acyl-CoA dehydrogenase, putative	3.26E+01
413	346	Scaffold_1_orf02040	Rv2731	DUF349 domain-containing protein	3.25E+01
414	347	Scaffold_1_orf05856	Rv0311	Uncharacterised protein	3.24E+01
415	348	Scaffold_1_orf03574	Rv1768	PE_PGRS31 PE-PGRS	3.22E+01
416	349	Scaffold_1_orf00271	Rv3834c	serine--tRNA ligase	3.16E+01
417	350	Scaffold_1_orf03093	Rv2059	zinc/manganese transporter substrate-binding protein	3.15E+01
418	351	Scaffold_1_orf04492	Rv1194c	conserved protein	3.15E+01
419	352	Scaffold_1_orf01725	Rv2921c	signal recognition particle-docking protein FtsY	3.14E+01
420	353	Scaffold_1_orf03364	Rv1902c	putative sialic acid transporter	3.14E+01
421	354	Scaffold_1_orf02266	Rv2580c	histidine--tRNA ligase	3.13E+01
422	355	Scaffold_1_orf00041	Rv0049	Uncharacterised protein	3.12E+01
423	356	Scaffold_1_orf01099	Rv3314c	thymidine phosphorylase	3.10E+01
424	357	Scaffold_1_orf03640	Rv1559	threonine ammonia-lyase	3.09E+01
425	358	Scaffold_1_orf06039	Rv0246	integral membrane protein	3.09E+01
426	-	Scaffold_1_orf02452	Rv2460c	ATP-dependent Clp protease proteolytic subunit	3.09E+01
427	359	Scaffold_1_orf05903	Rv0280	PPE3 PPE FAMILY P	3.05E+01
428	360	Scaffold_1_orf03255	Rv1971	MCE family protein	3.03E+01
429	-	Scaffold_1_orf03729	Rv1498A	Hypotetical protein	3.03E+01
430	361	Scaffold_1_orf03182	Rv2008c	putative ATPase	3.01E+01
431	-	Scaffold_1_orf04671	Rv1087	PE_PGRS21 PE-PGRS	2.98E+01
432	362	Scaffold_1_orf03737	Rv1493	methylmalonyl-CoA mutase	2.94E+01
433	364	Scaffold_1_orf03027	Rv2097c	proteasome component	2.93E+01
434	363	Scaffold_1_orf04118	Rv1636	universal stress protein	2.93E+01
435	365	Scaffold_1_orf04590	Rv1129c	XRE family transcriptional regulator	2.93E+01
436	366	Scaffold_1_orf04018	Rv1702c	HNH endonuclease	2.92E+01
437	367	Scaffold_1_orf05734	Rv0392c	NAD(P)/FAD-dependent oxidoreductase	2.92E+01
438	368	Scaffold_1_orf01740	Rv2910c	nuclear transport factor 2 family protein	2.91E+01
439	-	Scaffold_1_orf03896	Rv2027c	dosT two component sensor histidine kinase	2.91E+01
440	369	Scaffold_1_orf01422	Rv3106	NADPH:adrenodoxin oxidoreductase fprA (NADPH-ferre	2.91E+01
441	371	Scaffold_1_orf03924	Rv3327	IS110 family transposase	2.89E+01
442	370	Scaffold_1_orf01931	Rv2791c	transposase	2.89E+01
443	372	Scaffold_1_orf01385	Rv3130c	diacylglycerol O-acyltransferase	2.86E+01
444	-	Scaffold_1_orf00449	Rv3713	cobyric acid synthase cobQ2	2.86E+01
445	373	Scaffold_1_orf02271	Rv2577	purple acid phosphatase-related protein	2.85E+01
446	374	Scaffold_1_orf03670	Rv1537	DNA-damage-inducible protein P, putative	2.83E+01
447	375	Scaffold_1_orf02800	Rv2247	acyl-CoA carboxylase subunit beta	2.80E+01
448	-	Scaffold_1_orf04837	Rv1011	4-diphosphocytidyl-2-C-methyl-D-erythritol kinase ispE	2.76E+01
449	376	Scaffold_1_orf04128	Rv1630	30S ribosomal protein S1	2.76E+01
450	377	Scaffold_1_orf06074	Rv0223c	aldehyde dehydrogenase	2.72E+01
451	378	Scaffold_1_orf00371	Rv3763	lipoprotein LpqH	2.69E+01
452	379	Scaffold_1_orf00823	Rv3480c	wax ester/triacylglycerol synthase family O-acyltransfera	2.67E+01
453	380	Scaffold_1_orf05696	Rv0418	lipoprotein aminopeptidase LpqL	2.65E+01
454	381	Scaffold_1_orf03134	Rv2035	activator of Hsp90 ATPase 1 family protein	2.64E+01
455	-	Scaffold_1_orf03443	Rv1857	molybdate ABC transporter substrate-binding protein	2.64E+01
456	-	Scaffold_1_orf04230	Rv1356c	Uncharacterised protein	2.64E+01
457	-	Scaffold_1_orf02878	Rv2194	ubiquinol-cytochrome c reductase cytochrome c subunit	2.63E+01
458	382	Scaffold_1_orf00119	Rv0001	chromosomal replication initiator protein DnaA	2.62E+01

459	-	Scaffold_1_orf04997	Rv0911	VOC family protein	2.57E+01
460	383	Scaffold_1_orf03733	Rv1496	methylmalonyl Co-A mutase-associated GTPase MeaB	2.57E+01
461	384	Scaffold_1_orf02838	Rv2223c	alpha/beta hydrolase	2.55E+01
462	-	Scaffold_1_orf00769	Rv3508	PE_PGRS54 PE-PGRS	2.55E+01
463	385	Scaffold_1_orf01602	Rv2996c	D-3-phosphoglycerate dehydrogenase	2.54E+01
464	386	Scaffold_1_orf00922	Rv3411c	inosine-5-monophosphate dehydrogenase guaB2 - 1578	2.53E+01
465	-	Scaffold_1_orf01349	Rv3154	NADH:ubiquinone oxidoreductase subunit J	2.53E+01
466	387	Scaffold_1_orf03614	Rv1577c	phage prohead protease, HK97 family	2.52E+01
467	388	Scaffold_1_orf05136	Rv0808	amidophosphoribosyltransferase PurF	2.52E+01
468	-	Scaffold_1_orf01322	Rv3177	alpha/beta hydrolase	2.51E+01
469	389	Scaffold_1_orf05664	Rv0443	DinB family protein	2.50E+01
470	390	Scaffold_1_orf02077	Rv2703	RNA polymerase sigma factor sigA	2.50E+01
471	392	Scaffold_1_orf04500	Rv1186c	Conserved protein	2.46E+01
472	391	Scaffold_1_orf01710	Rv2930	fatty-acid-CoA ligase fadD26	2.46E+01
473	393	Scaffold_1_orf01640	Rv2970c	lipase/esterase LIPN	2.44E+01
474	-	Scaffold_1_orf02788	Rv2257c	putative Beta-lactamase	2.43E+01
475	395	Scaffold_1_orf01154	Rv3280	propionyl-CoA carboxylase beta chain	2.42E+01
476	396	Scaffold_1_orf02528	Rv2411c	circularly permuted type 2 ATP-grasp protein	2.42E+01
477	394	Scaffold_1_orf02499	Rv2429	alkyl hydroperoxide reductase	2.42E+01
478	397	Scaffold_1_orf04938	Rv0946c	glucose-6-phosphate isomerase	2.40E+01
479	-	Scaffold_1_orf06180	Rv0153c	phosphotyrosine protein phosphatase PtpB	2.40E+01
480	400	Scaffold_1_orf03904	Rv1384	carbamoyl phosphate synthase large subunit	2.38E+01
481	398	Scaffold_1_orf00044	Rv0047c	PadR family transcriptional regulator	2.38E+01
482	399	Scaffold_1_orf04364	Rv1275	DUF3558 domain-containing protein	2.38E+01
483	-	Scaffold_1_orf01840	Rv2867c	DUF4081 domain-containing protein	2.33E+01
484	401	Scaffold_1_orf01086	Rv3324c	Uncharacterised protein	2.33E+01
485	402	Scaffold_1_orf04806	Rv1029	potassium-transporting ATPase subunit A	2.32E+01
486	403	Scaffold_1_orf06125	Rv0189c	dihydroxy-acid dehydratase	2.31E+01
487	-	Scaffold_1_orf04489	Rv1196	PPE18 PPE FAMILY	2.30E+01
488	405	Scaffold_1_orf03555	Rv1780	conserved protein	2.29E+01
489	404	Scaffold_1_orf02842	Rv2220	glutamine synthetase 1	2.29E+01
490	406	Scaffold_1_orf00624	Rv3590c	PE_PGRS58 PE-PGR	2.27E+01
491	-	Scaffold_1_orf04194	Rv1451	protoheme IX farnesyltransferase	2.27E+01
492	-	Scaffold_1_orf00929	Rv3406	TauD/TfdA family dioxygenase	2.24E+01
493	-	Scaffold_1_orf03188	Rv2005c	universal stress protein	2.24E+01
494	-	Scaffold_1_orf00448	Rv3714c	conserved hypothetical protein	2.24E+01
495	-	Scaffold_1_orf03539	Rv1790	PPE27 PPE FAMILY	2.24E+01
496	407	Scaffold_1_orf06042	Rv0244c	acyl-CoA dehydrogenase	2.17E+01
497	-	Scaffold_1_orf00478	Rv3692	MoxR family ATPase	2.16E+01
498	408	Scaffold_1_orf00350	Rv3778c	Selenocysteine lyase	2.14E+01
499	409	Scaffold_1_orf00186	Rv3884c	stage V sporulation protein K-like protein	2.13E+01
500	410	Scaffold_1_orf00878	Rv3436c	glucosamine-fructose-6-phosphate aminotransferase glr	2.13E+01
501	411	Scaffold_1_orf02590	Rv2379c	non-ribosomal peptide synthetase	2.11E+01
502	412	Scaffold_1_orf03730	Rv1498c	class I SAM-dependent methyltransferase	2.09E+01
503	413	Scaffold_1_orf03964	Rv1733c	transmembrane protein	2.04E+01
504	414	Scaffold_1_orf04899	Rv0970	integral membrane protein	2.04E+01
505	415	Scaffold_1_orf03957	Rv1736c	nitrate reductase molybdenum cofactor assembly chape	2.03E+01
506	416	Scaffold_1_orf04207	Rv1364c	sigma factor regulatory protein	2.03E+01
507	-	Scaffold_1_orf00351	Rv3777	putative oxidoreductase	2.02E+01
508	-	Scaffold_1_orf02174	Rv2638	anti-anti-sigma factor	1.97E+01
509	417	Scaffold_1_orf04515	Rv1175c	NADPH-dependent 2,4-dienoyl-CoA reductase	1.97E+01
510	418	Scaffold_1_orf03231	Rv1984c	cutinase cfp21	1.96E+01
511	419	Scaffold_1_orf02060	Rv2715	alpha/beta hydrolase	1.94E+01
512	420	Scaffold_1_orf03444	Rv1856c	short-chain dehydrogenase	1.92E+01
513	421	Scaffold_1_orf00503	Rv3676	cAMP receptor protein	1.91E+01
514	423	Scaffold_1_orf05332	Rv0684	elongation factor G	1.89E+01
515	422	Scaffold_1_orf05775	Rv0364	membrane protein	1.89E+01
516	-	Scaffold_1_orf04718	Rv1063c	patatin family protein	1.88E+01
517	424	Scaffold_1_orf03497	Rv1816	TetR/AcrR family transcriptional regulator	1.88E+01
518	-	Scaffold_1_orf02787	Rv2258c	class I SAM-dependent methyltransferase	1.88E+01
519	425	Scaffold_1_orf01280	Rv3202c	ATP-dependent DNA helicase	1.87E+01
520	-	Scaffold_1_orf03129	Rv2038c	ABC transporter ATP-binding protein	1.86E+01
521	-	Scaffold_1_orf01390	Rv3128c	Conserved protein of uncharacterised function	1.84E+01
522	-	Scaffold_1_orf01839	Rv2868c	flavodoxin-dependent (E)-4-hydroxy-3-methylbut-2-enyl-	1.82E+01
523	426	Scaffold_1_orf05865	Rv0305c	PPE6 PPE FAMILY	1.82E+01
524	427	Scaffold_1_orf02967	Rv2135c	Uncharacterised protein	1.82E+01
525	-	Scaffold_1_orf01100	Rv3313c	adenosine deaminase family protein	1.81E+01
526	-	Scaffold_1_orf03728	Rv1499	glycosyltransferase	1.81E+01
527	428	Scaffold_1_orf00013	Rv0066c	isocitrate dehydrogenase (NADP(+)	1.78E+01
528	429	Scaffold_1_orf03319	Rv1933c	acyl-CoA dehydrogenase	1.77E+01
529	-	Scaffold_1_orf00459	Rv3707c	putative secreted protein	1.77E+01
530	-	Scaffold_1_orf00923	Rv3410c	GuaB3 family IMP dehydrogenase-related protein	1.77E+01
531	431	Scaffold_1_orf01946	Rv2783c	polynucleotide phosphorylase	1.76E+01
532	430	Scaffold_1_orf05005	Rv0905	enoyl-CoA hydratase	1.76E+01
533	432	Scaffold_1_orf05142	Rv0803	Phosphoribosylformylglycinamidine synthase subunit Pu	1.76E+01
534	-	Scaffold_1_orf01673	Rv2951c	LLM class flavin-dependent oxidoreductase	1.74E+01
535	433	Scaffold_1_orf02378	Rv2504c	succinyl-CoA:3-ketoacid-CoA transferase	1.73E+01
536	-	Scaffold_1_orf05336	Rv0680c	DUF3060 domain-containing protein	1.72E+01
537	434	Scaffold_1_orf00547	Rv3649	DEAD/DEAH box helicase	1.72E+01
538	435	Scaffold_1_orf05994	Rv0278c	PE_PGRS3 PE-PGRS	1.72E+01
539	436	Scaffold_1_orf02845	Rv2219	DUF4191 domain-containing protein	1.71E+01
540	-	Scaffold_1_orf02026	Rv2739c	glycosyl transferase - 1167 nt	1.71E+01
541	437	Scaffold_1_orf01758	Rv2900c	formate dehydrogenase H FdhF	1.70E+01
542	438	Scaffold_1_orf02398	Rv2492	thymidylate synthase-like protein	1.69E+01
543	439	Scaffold_1_orf05290	Rv0711	arylsulfatase AtsA	1.69E+01
544	440	Scaffold_1_orf03073	Rv2071c	precorrin-4 C11-methyltransferase	1.67E+01
545	441	Scaffold_1_orf00317	Rv3800c	polyketide synthase	1.66E+01
546	442	Scaffold_1_orf00050	Rv0044c	LLM class F420-dependent oxidoreductase	1.65E+01
547	-	Scaffold_1_orf03355	Rv1909c	transcriptional repressor	1.64E+01
548	443	Scaffold_1_orf06158	Rv0167	ABC transporter permease	1.62E+01
549	-	Scaffold_1_orf03933	Rv1751	hypothetical protein	1.61E+01
550	445	Scaffold_1_orf04897	Rv0971c	enoyl-CoA hydratase	1.59E+01
551	-	Scaffold_1_orf03482	Rv1826	Chain A, Crystal Structure Of Glycine Cleavage System	1.59E+01
552	444	Scaffold_1_orf02010	Rv2744c	35kd antigen	1.59E+01
553	-	Scaffold_1_orf02457	Rv2456c	MFS transporter	1.59E+01
554	-	Scaffold_1_orf00926	Rv3408	ribonuclease VapC47	1.57E+01
555	446	Scaffold_1_orf05501	Rv0590	virulence factor mce family protein	1.56E+01
556	-	Scaffold_1_orf00092	Rv0015c	serine/threonine protein kinase	1.56E+01
557	-	Scaffold_1_orf02455	Rv2457c	ATP-dependent Clp protease ATP-binding subunit ClpX	1.56E+01
558	447	Scaffold_1_orf00304	Rv3810	exported repetitive protein pirG	1.51E+01
559	448	Scaffold_1_orf02254	Rv2587c	protein translocase subunit SecD	1.50E+01
560	-	Scaffold_1_orf02158	Rv2645	Hypothetical protein	1.49E+01
561	-	Scaffold_1_orf03368	Rv1900c	putative alpha/beta hydrolase family protein	1.49E+01
562	449	Scaffold_1_orf00679	Rv3555c	PF13338 domain protein	1.48E+01
563	-	Scaffold_1_orf02249	Rv2589	aspartate aminotransferase family protein	1.48E+01
564	452	Scaffold_1_orf05401	Rv0643c	methoxy mycolic acid synthase MmaA3	1.46E+01
565	450	Scaffold_1_orf05120	Rv0833	PE_PGRS13 PE-PGRS	1.46E+01
566	451	Scaffold_1_orf01753	Rv2903c	signal peptidase I	1.46E+01
567	453	Scaffold_1_orf00626	Rv3589	A/G-specific adenine glycosylase	1.45E+01
568	454	Scaffold_1_orf00049	Rv0045c	myo-inositol-1-phosphate synthase	1.44E+01
569	455	Scaffold_1_orf01927	Rv2793c	tRNA pseudouridine(55) synthase TruB	1.44E+01
570	456	Scaffold_1_orf03848	Rv1421	RNase adaptor protein RapZ	1.43E+01
571	457	Scaffold_1_orf03762	Rv1475c	aconitate hydratase	1.41E+01
572	-	Scaffold_1_orf01921	Rv2796c	lipoprotein LppV	1.39E+01
573	-	Scaffold_1_orf00090	Rv0016c	penicillin-binding protein A	1.38E+01
574	458	Scaffold_1_orf01750	Rv2905	lipoprotein LppW	1.37E+01
575	459	Scaffold_1_orf03701	Rv1518	glycosyl transferase family protein	1.35E+01
576	-	Scaffold_1_orf01200	Rv3248c	adenosylhomocysteinase	1.34E+01
577	461	Scaffold_1_orf00569	Rv3634c	NAD-dependent epimerase/dehydratase family protein	1.33E+01
578	460	Scaffold_1_orf05214	Rv0755c	PPE12 PPE FAMILY	1.33E+01
579	-	Scaffold_1_orf00671	Rv3561	fatty acid--CoA ligase	1.31E+01
580	-	Scaffold_1_orf05822	Rv0336	Conserved protein of uncharacterised function	1.30E+01
581	-	Scaffold_1_orf05987	Rv0515	Conserved protein of uncharacterised function	1.30E+01
582	-	Scaffold_1_orf03321	Rv1932	2-Cys peroxiredoxin	1.30E+01

583	462	Scaffold_1_orf00349	Rv3779	transmembrane protein alanine and leucine rich	1.29E+01
584	464	Scaffold_1_orf03672	Rv1536	isoleucyl-tRNA synthetase ileS	1.27E+01
585	463	Scaffold_1_orf06228	Rv0125	serine protease	1.27E+01
586	-	Scaffold_1_orf01204	Rv3245c	two component system sensor histidine kinase mtrB	1.26E+01
587	465	Scaffold_1_orf06222	Rv0129c	esterase, putative, antigen 85-C	1.26E+01
588	466	Scaffold_1_orf01862	Rv2856	HoxN/HupN/NixA family nickel/cobalt transporter	1.25E+01
589	-	Scaffold_1_orf04317	Rv1306	F0F1 ATP synthase subunit B	1.25E+01
590	-	Scaffold_1_orf02920	Rv2162c	PE_PGRS38 PE-PGR	1.25E+01
591	467	Scaffold_1_orf02608	Rv2368c	PhoH family protein	1.22E+01
592	-	Scaffold_1_orf04835	Rv1013	long-chain-fatty-acid--CoA ligase	1.22E+01
593	469	Scaffold_1_orf00326	Rv3794	integral membrane indolylacetylinositol arabinosyltransfe	1.21E+01
594	468	Scaffold_1_orf00737	Rv3522	lipid-transfer protein	1.21E+01
595	-	Scaffold_1_orf06201	Rv0144	transcriptional regulator, tetR-family	1.18E+01
596	470	Scaffold_1_orf05205	Rv0761c	alcohol dehydrogenase	1.14E+01
597	471	Scaffold_1_orf05921	Rv0557	glycosyl transferase family protein	1.14E+01
598	473	Scaffold_1_orf03657	Rv1547	DNA polymerase III subunit alpha	1.13E+01
599	472	Scaffold_1_orf01375	Rv3136	PPE51 PPE FAMILY	1.13E+01
600	474	Scaffold_1_orf05431	Rv0628c	FIST N domain protein	1.12E+01
601	476	Scaffold_1_orf03089	Rv2062c	cobaltochelatase subunit CobN	1.11E+01
602	475	Scaffold_1_orf02521	Rv2416c	enhanced intracellular survival protein eis	1.11E+01
603	-	Scaffold_1_orf05054	Rv0872c	PE_PGRS15 PE-PGR	1.09E+01
604	477	Scaffold_1_orf01300	Rv3191c	transposase for insertion sequence element IS1086 fam	1.09E+01
605	478	Scaffold_1_orf00862	Rv3447c	type VII secretion system ESX-4 FtsK/SpoIIIE family ATI	1.07E+01
606	479	Scaffold_1_orf05392	Rv0648	alpha-mannosidase	1.07E+01
607	480	Scaffold_1_orf03889	Rv1392	S-adenosylmethionine synthetase	1.06E+01
608	-	Scaffold_1_orf04037	Rv1688	DNA-3-methyladenine glycosylase	1.06E+01
609	481	Scaffold_1_orf01531	Rv3042c	phosphoserine phosphatase SerB	1.05E+01
610	482	Scaffold_1_orf03317	Rv1934c	acyl-CoA dehydrogenase	1.05E+01
611	483	Scaffold_1_orf05724	Rv0399c	D-alanyl-D-alanine carboxypeptidase	1.05E+01
612	484	Scaffold_1_orf02894	Rv2181	Probable conserved integral membrane protein	1.04E+01
613	485	Scaffold_1_orf05490	Rv0597c	ATPase AAA+ superfamily protein	1.04E+01
614	-	Scaffold_1_orf00775	Rv3508	PE_PGRS54 PE-PGRS	1.01E+01
615	486	Scaffold_1_orf05360	Rv0668	DNA-directed RNA polymerase subunit beta	1.01E+01
616	487	Scaffold_1_orf00573	Rv3630	integral membrane protein	9.96E+00
617	488	Scaffold_1_orf04647	Rv1095	ATP-binding protein	9.92E+00
618	489	Scaffold_1_orf01359	Rv3148	NADH dehydrogenase (quinone) subunit D	9.76E+00
619	490	Scaffold_1_orf06044	Rv0243	acetyl-CoA C-acyltransferase	9.76E+00
620	-	Scaffold_1_orf03407	Rv1877	MFS transporter	9.65E+00
621	491	Scaffold_1_orf03446	Rv1854c	NAD(P)/FAD-dependent oxidoreductase	9.63E+00
622	492	Scaffold_1_orf02476	Rv2444c	Putative ribonuclease E Rne	9.40E+00
623	493	Scaffold_1_orf03165	Rv2048c	beta-ketoacyl synthase	9.40E+00
624	494	Scaffold_1_orf02621	Rv2357c	glycyl-tRNA synthetase	9.36E+00
625	495	Scaffold_1_orf02758	Rv2280	FAD-binding oxidoreductase	9.36E+00
626	-	Scaffold_1_orf02067	Rv2711	metal-dependent transcriptional regulator	9.32E+00
627	496	Scaffold_1_orf01194	Rv3254	putative secreted protein	9.30E+00
628	497	Scaffold_1_orf06011	Rv0267	NarK family nitrate/nitrite MFS transporter	9.28E+00
629	-	Scaffold_1_orf01266	Rv3210c	conserved protein	9.28E+00
630	-	Scaffold_1_orf01166	Rv3270	copper-translocating P-type ATPase	9.24E+00
631	498	Scaffold_1_orf03590	Rv1760	diacylglycerol O-acyltransferase	9.18E+00
632	499	Scaffold_1_orf03833	Rv1432	NAD(P)/FAD-dependent oxidoreductase	9.08E+00
633	500	Scaffold_1_orf01215	Rv3240c	Putative preprotein translocase SecA1 1 subunit	9.06E+00
634	-	Scaffold_1_orf02484	Rv2438c	glutamine-dependent NAD(+) synthetase nadE	8.99E+00
635	501	Scaffold_1_orf04249	Rv1345	long-chain-fatty-acid--ACP ligase	8.42E+00
636	502	Scaffold_1_orf02638	Rv2351c	Phospholipase C	8.39E+00
637	503	Scaffold_1_orf06147	Rv0174	Mce family protein Mce1F	8.34E+00
638	504	Scaffold_1_orf03111	Rv2048c	beta-ketoacyl synthase	8.31E+00
639	505	Scaffold_1_orf02234	Rv2601	spermidine synthaset	8.21E+00
640	506	Scaffold_1_orf02427	Rv2476c	NAD-dependent glutamate dehydrogenase	8.18E+00
641	-	Scaffold_1_orf04517	Rv1173	7,8-didemethyl-8-hydroxy-5-deazariboflavin synthase	7.98E+00
642	-	Scaffold_1_orf04157	Rv1613	Chain A, Structure Of Mycobacterium Tuberculosis Trypt	7.94E+00
643	507	Scaffold_1_orf04314	Rv1308	F0F1 ATP synthase subunit alpha	7.83E+00
644	-	Scaffold_1_orf00960	Rv3388	PE_PGRS52 PE-PGRS	7.71E+00
645	508	Scaffold_1_orf02425	Rv2477c	energy-dependent translational throttle protein EttA	7.70E+00
646	-	Scaffold_1_orf00745	Rv3517	cullin-2C a subunit of E3 ubiquitin ligase	7.69E+00
647	-	Scaffold_1_orf02965	Rv2136c	Undecaprenyl-diphosphatase	7.61E+00
648	-	Scaffold_1_orf01612	Rv2990c	class I SAM-dependent methyltransferase	7.55E+00
649	509	Scaffold_1_orf06069	Rv0226c	conserved membrane protein	7.46E+00
650	510	Scaffold_1_orf02780	Rv2264c	Conserved protein of uncharacterised function	7.38E+00
651	511	Scaffold_1_orf00568	Rv3635	transmembrane protein	7.33E+00
652	512	Scaffold_1_orf03834	Rv1431	membrane protein	7.30E+00
653	513	Scaffold_1_orf01344	Rv3159c	PPE53 PPE FAMILY	7.28E+00
654	-	Scaffold_1_orf04961	Rv0930	phosphate-transport membrane ABC transporter pstA1	7.15E+00
655	514	Scaffold_1_orf06226	Rv0126	treS product	7.11E+00
656	-	Scaffold_1_orf05750	Rv0381c	Conserved protein of uncharacterised function	7.10E+00
657	-	Scaffold_1_orf02062	Rv2714	conserved alanine and leucine rich protein	7.03E+00
658	-	Scaffold_1_orf03594	Rv1759c	Uncharacterised protein	6.98E+00
659	-	Scaffold_1_orf00434	Rv3725	hypothetical oxidoreductase	6.94E+00
660	-	Scaffold_1_orf06197	Rv0145	class I SAM-dependent methyltransferase	6.77E+00
661	-	Scaffold_1_orf06189	Rv0149	quinone oxidoreductase	6.66E+00
662	515	Scaffold_1_orf05051	Rv0873	acyl-CoA dehydrogenase	6.61E+00
663	516	Scaffold_1_orf00517	Rv3667	acetyl-coenzyme A synthetase	6.60E+00
664	517	Scaffold_1_orf05542	Rv0570	ribonucleoside-diphosphate reductase subunit alpha	6.53E+00
665	-	Scaffold_1_orf01622	Rv2982c	NAD(P)H-dependent glycerol-3-phosphate dehydrogena	6.39E+00
666	518	Scaffold_1_orf01695	Rv2940c	polyketide synthase	6.29E+00
667	519	Scaffold_1_orf05711	Rv0408	putative phosphate acetyltransferase	6.23E+00
668	-	Scaffold_1_orf00021	Rv0062	putative endoglucanase A	6.13E+00
669	520	Scaffold_1_orf01704	Rv2933	phthiocerol type I polyketide synthase PpsC	6.07E+00
670	-	Scaffold_1_orf01623	Rv2981c	D-alanine--D-alanine ligase	5.75E+00
671	-	Scaffold_1_orf05774	Rv0365c	fructose-bisphosphate aldolase	5.71E+00
672	-	Scaffold_1_orf02248	Rv2590	fatty-acid-CoA ligase	5.68E+00
673	521	Scaffold_1_orf03635	Rv1563c	4-alpha-D-glucan 1-alpha-D-glucosylmutase	5.62E+00
674	-	Scaffold_1_orf00743	Rv3518c	Putative cytochrome P450 monooxygenase 142 cyp142	5.48E+00
675	-	Scaffold_1_orf00656	Rv3570c	flavin-dependent monooxygenase oxygenase subunit Hs	5.45E+00
676	-	Scaffold_1_orf03784	Rv1462	Fe-S cluster assembly protein SufD	5.41E+00
677	-	Scaffold_1_orf04822	Rv1020	transcription-repair coupling factor	5.38E+00
678	-	Scaffold_1_orf04036	Rv1689	tyrosyl-tRNA synthase	5.10E+00
679	522	Scaffold_1_orf03786	Rv1461	Fe-S cluster assembly protein SufB	5.08E+00
680	-	Scaffold_1_orf05091	Rv0845	two component system sensor kinase	5.05E+00
681	524	Scaffold_1_orf04360	Rv1278	DNA double-strand break repair Rad50 ATPase	4.91E+00
682	523	Scaffold_1_orf02469	Rv2448c	Valine--tRNA ligase	4.91E+00
683	-	Scaffold_1_orf04149	Rv1617	pyruvate kinase	4.84E+00
684	-	Scaffold_1_orf04121	Rv1634	MFS transporter	4.56E+00
685	-	Scaffold_1_orf01454	Rv3088	wax ester/triacylglycerol synthase family O-acyltransfera	4.53E+00
686	525	Scaffold_1_orf05714	Rv0405	membrane bound polyketide synthase pks6	4.43E+00
687	526	Scaffold_1_orf00018	Rv0064	membrane protein	4.39E+00
688	527	Scaffold_1_orf02584	Rv2381c	polyketide synthase, putative	4.26E+00
689	-	Scaffold_1_orf05634	Rv0458	aldehyde dehydrogenase family protein	4.24E+00
690	-	Scaffold_1_orf03898	Rv1387	PPE20 PPE FAMILY	3.99E+00
691	-	Scaffold_1_orf05959	Rv0532	acyl-[acyl-carrier protein] desaturaseCoA synthetase	3.62E+00
692	-	Scaffold_1_orf00315	Rv3801c	fatty-acid--CoA ligase	3.42E+00
693	528	Scaffold_1_orf05790	Rv0355c	PPE8 PPE FAMILY	3.32E+00
694	-	Scaffold_1_orf03471	Rv1836c	von Willebrand factor-2C type A	3.17E+00
695	-	Scaffold_1_orf05707	Rv0410c	serine/threonine protein kinase	3.00E+00
696	-	Scaffold_1_orf03469	Rv1837c	malate synthase G	2.92E+00
697	-	Scaffold_1_orf00423	Rv3729	S-adenosylmethionine-dependent methyltransferase, cla	2.56E+00
698	-	Scaffold_1_orf05114	Rv0834c	PE_PGRS14 PE-PGR	2.44E+00
699	529	Scaffold_1_orf06268	Rv0101	non-ribosomal peptide synthetase	1.72E+00
700	-	Scaffold_1_orf03800	Rv1450c	PE_PGRS27 PE-PGR	1.54E+00
701	-	Scaffold_1_orf02587	Rv2380c	non-ribosomal peptide synthetase	1.24E+00
702	-	Scaffold_1_orf01708	Rv2931	phthiocerol type I polyketide synthase PpsA	1.15E+00
