# Supplementary material for: Targeted RNA-Seq Reveals the M. tuberculosis Transcriptome from an In Vivo Infection Model
Source: Biology (Basel). 2021 Aug 31;10(9):848. doi: 10.3390/biology10090848 (PMC8467220; doi:10.3390/biology10090848)
Supplement: Supplementary file 1 [file biology-10-00848-s001.zip › TableS3_r1.pdf]

Table S3. InterPro domains and families significantly overrepresented in the 529 most expressed M. tuberculosis genes.

InterPro ID	Category	Description	P-Value
IPR014043	domain	Acyl transferase	6.267E-07
IPR009081	domain	Phosphopantetheine binding ACP domain	1.438E-06
IPR014030	domain	Beta-ketoacyl synthase, N-terminal	2.747E-05
IPR014031	domain	Beta-ketoacyl synthase, C-terminal	4.885E-05
IPR013968	domain	Polyketide synthase, ketoreductase domain	1.624E-04
IPR023836	family	EccCa-like, Actinobacteria	1.087E-03
IPR023837	family	EccCb-like, Actinobacteria	1.087E-03
IPR032821	domain	Ketoacyl-synthetase, C-terminal extension	1.190E-03
IPR006091	domain	Acyl-CoA oxidase/dehydrogenase, central doma	1.363E-03
IPR020807	domain	Polyketide synthase, dehydratase domain	1.757E-03
IPR009075	domain	Acyl-CoA dehydrogenase/oxidase C-terminal	1.995E-03
IPR002543	domain	FtsK domain	6.180E-03
IPR003029	domain	S1 domain	7.039E-03
IPR003495	domain	CobW/HypB/UreG, nucleotide-binding domain	7.039E-03
IPR023753	domain	FAD/NAD(P	1.360E-02
IPR000788	domain	Ribonucleotide reductase large subunit, C-termi	1.558E-02
IPR001030	domain	Aconitase/3-isopropylmalate dehydratase large s	1.558E-02
IPR002300	domain	Aminoacyl-tRNA synthetase, class Ia	1.558E-02
IPR003714	domain	PhoH-like protein	1.558E-02
IPR004100	domain	ATPase, F1/V1/A1 complex, alpha/beta subunit,	1.558E-02
IPR011115	domain	SecA DEAD-like, N-terminal	1.558E-02
IPR011116	domain	SecA Wing/Scaffold	1.558E-02
IPR011130	domain	SecA, preprotein cross-linking domain	1.558E-02
IPR013509	domain	Ribonucleotide reductase large subunit, N-termi	1.558E-02
IPR014018	domain	SecA motor DEAD	1.558E-02
IPR023234	domain	NarG-like domain	1.558E-02
IPR025878	domain	Acetyl-CoA dehydrogenase-like C-terminal doma	1.558E-02
IPR000185	family	Protein translocase subunit SecA	1.558E-02
IPR001844	family	Chaperonin Cpn60	1.558E-02
IPR002423	family	Chaperonin Cpn60/TCP-1 family	1.558E-02
IPR003816	family	Nitrate reductase, gamma subunit	1.558E-02
IPR004392	family	Hydrogenase maturation factor HypB	1.558E-02
IPR005372	family	Uncharacterised protein family UPF0182	1.558E-02
IPR013538	family	Activator of Hsp90 ATPase homologue 1-like	1.558E-02
IPR013786	domain	Acyl-CoA dehydrogenase/oxidase, N-terminal	1.792E-02
IPR001584	domain	Integrase, catalytic core	1.811E-02
IPR013154	domain	Alcohol dehydrogenase, N-terminal	1.857E-02
IPR006656	domain	Molybdopterin oxidoreductase	2.900E-02
IPR003959	domain	ATPase, AAA-type, core	3.870E-02
IPR000194	domain	ATPase, F1/V1/A1 complex, alpha/beta subunit,	4.287E-02
IPR004675	domain	Alkylhydroperoxidase AhpD core	4.287E-02
IPR004843	domain	Calcineurin-like phosphoesterase domain, ApaH	4.287E-02
IPR013155	domain	Methionyl/Valyl/Leucyl/Isoleucyl-tRNA synthetas	4.287E-02
IPR025948	domain	HTH-like domain	4.287E-02
IPR000537	family	UbiA prenyltransferase family	4.287E-02
IPR001714	family	Peptidase M24, methionine aminopeptidase	4.287E-02
IPR006254	family	Isocitrate lyase	4.287E-02
IPR013126	family	Heat shock protein 70 family	4.287E-02
IPR024520	family	Protein of unknown function DUF3558	4.287E-02
IPR038965	family	Transposase InsF-like	4.287E-02
IPR004707	family	Membrane transport protein MmpL family	4.611E-02
