# Supplementary material for: Targeted RNA-Seq Reveals the M. tuberculosis Transcriptome from an In Vivo Infection Model
Source: Biology (Basel). 2021 Aug 31;10(9):848. doi: 10.3390/biology10090848 (PMC8467220; doi:10.3390/biology10090848)
Supplement: Supplementary file 1 [file biology-10-00848-s001.zip › TableS4_r1.pdf]

Tabla S4. Gene Ontology terms significantly enriched by category related to the 529 most expressed M. tuberculosis gene.

GO ID	Nombre GO	P-Value
**A. Biological Process**		
GO:0031295	T cell costimulation	1.765E-05
GO:0031294	lymphocyte costimulation	1.765E-05
GO:0009081	branched-chain amino acid metabolic process	4.007E-05
GO:0009083	branched-chain amino acid catabolic process	1.102E-04
GO:0006549	isoleucine metabolic process	1.197E-04
GO:0000288	nuclear-transcribed mRNA catabolic process, deadenylation-dependent decay	2.663E-04
GO:0006573	valine metabolic process	3.513E-04
GO:0060184	cell cycle switching	4.878E-04
GO:0051728	cell cycle switching, mitotic to meiotic cell cycle	4.878E-04
GO:0006574	valine catabolic process	5.108E-04
GO:0006552	leucine catabolic process	5.108E-04
GO:0006550	isoleucine catabolic process	5.108E-04
GO:0006551	leucine metabolic process	6.270E-04
GO:0006939	smooth muscle contraction	6.963E-04
GO:0140013	meiotic nuclear division	7.369E-04
GO:1901565	organonitrogen compound catabolic process	1.030E-03
GO:0006631	fatty acid metabolic process	1.040E-03
GO:0031340	positive regulation of vesicle fusion	1.211E-03
GO:0031338	regulation of vesicle fusion	1.211E-03
GO:0044281	small molecule metabolic process	1.287E-03
**B. Cellular Component**		
GO:0005643	nuclear pore	4.20E-03
GO:0000932	P-body	5.39E-03
GO:0009986	cell surface	6.00E-03
GO:0098978	glutamatergic synapse	6.18E-03
GO:1990527	Tec1p-Ste12p-Dig1p complex	7.04E-03
GO:0110165	cellular anatomical entity	1.23E-02
GO:1990526	Ste12p-Dig1p-Dig2p complex	1.60E-02
GO:0030496	midbody	1.81E-02
GO:0009325	nitrate reductase complex	1.83E-02
GO:0016020	membrane	2.56E-02
GO:0031300	intrinsic component of organelle membrane	2.57E-02
GO:0031301	integral component of organelle membrane	2.57E-02
GO:0098982	GABA-ergic synapse	2.90E-02
GO:0062071	Pi Mi complex	2.90E-02
GO:0005863	striated muscle myosin thick filament	2.90E-02
GO:0030015	CCR4-NOT core complex	3.08E-02
GO:0030014	CCR4-NOT complex	3.08E-02
GO:0089713	Cbf1-Met4-Met28 complex	3.87E-02
GO:0042101	T cell receptor complex	4.29E-02
GO:0042105	alpha-beta T cell receptor complex	4.29E-02
**C. Molecular Function**		
GO:0004085	butyryl-CoA dehydrogenase activity	1.62E-04
GO:0005488	binding	6.96E-04
GO:0052890	oxidoreductase activity, acting on the CH-CH group of donors, with a flavin as ac	7.83E-04
GO:0043168	anion binding	1.16E-03
GO:0003955	NAD(P)H dehydrogenase (quinone) activity	1.94E-03
GO:0017056	structural constituent of nuclear pore	2.87E-03
GO:0004962	endothelin receptor activity	2.94E-03
GO:0036094	small molecule binding	3.15E-03
GO:1901363	heterocyclic compound binding	4.09E-03
GO:0097159	organic cyclic compound binding	4.10E-03
GO:0035375	zymogen binding	6.18E-03
GO:0097367	carbohydrate derivative binding	6.87E-03
GO:0030527	structural constituent of chromatin	7.04E-03
GO:0004590	orotidine-5'-phosphate decarboxylase activity	7.09E-03
GO:0005044	scavenger receptor activity	7.23E-03
GO:0038024	cargo receptor activity	7.23E-03
GO:1901265	nucleoside phosphate binding	7.82E-03
GO:0000166	nucleotide binding	7.82E-03
GO:0004032	alditol:NADP+ 1-oxidoreductase activity	1.11E-02
GO:0043167	ion binding	1.20E-02
