# Supplementary material for: Targeted RNA-Seq Reveals the M. tuberculosis Transcriptome from an In Vivo Infection Model
Source: Biology (Basel). 2021 Aug 31;10(9):848. doi: 10.3390/biology10090848 (PMC8467220; doi:10.3390/biology10090848)
Supplement: Supplementary file 1 [file biology-10-00848-s001.zip › TableS5_r1.pdf]

Table S5. KEGG pathways overrepresented by the 529 most expressed M. tuberculosis genes.

KEGG ID	Pathway	%
03020	RNA polymerase	75.00
00630	Glyoxylate and dicarboxylate metabolism	34.15
03018	RNA degradation	33.33
00562	Inositol phosphate metabolism	33.33
00910	Nitrogen metabolism	31.82
05152	Tuberculosis	30.77
00680	Methane metabolism	28.57
00290	Valine, leucine and isoleucine biosynthesis	28.57
00430	Taurine and hypotaurine metabolism	28.57
00020	Citrate cycle (TCA cycle)	28.13
00730	Thiamine metabolism	27.27
00983	Drug metabolism - other enzymes	27.27
00061	Fatty acid biosynthesis	26.67
02024	Quorum sensing	25.00
00010	Glycolysis / Gluconeogenesis	24.24
00270	Cysteine and methionine metabolism	24.24
00260	Glycine, serine and threonine metabolism	24.00
03060	Protein export	23.53
01053	Biosynthesis of siderophore group nonribosomal peptides	22.22
03070	Bacterial secretion system	21.43
00572	Arabinogalactan biosynthesis - Mycobacterium	21.43
00760	Nicotinate and nicotinamide metabolism	21.05
00620	Pyruvate metabolism	20.83
02020	Two-component system	20.34
00660	C5-Branched dibasic acid metabolism	20.00
00350	Tyrosine metabolism	20.00
00190	Oxidative phosphorylation	18.75
00640	Propanoate metabolism	18.37
00072	Synthesis and degradation of ketone bodies	18.18
00521	Streptomycin biosynthesis	18.18
00625	Chloroalkane and chloroalkene degradation	18.18
00561	Glycerolipid metabolism	17.24
00860	Porphyrin and chlorophyll metabolism	17.14
00500	Starch and sucrose metabolism	16.67
00780	Biotin metabolism	16.67
00280	Valine, leucine and isoleucine degradation	15.79
00520	Amino sugar and nucleotide sugar metabolism	15.38
00240	Pyrimidine metabolism	15.38
00220	Arginine biosynthesis	15.00
00480	Glutathione metabolism	14.29
00650	Butanoate metabolism	14.04
03010	Ribosome	13.11
03410	Base excision repair	12.50
00071	Fatty acid degradation	12.00
00900	Terpenoid backbone biosynthesis	11.54
00230	Purine metabolism	11.11
00790	Folate biosynthesis	11.11
00770	Pantothenate and CoA biosynthesis	11.11
02010	ABC transporters	10.81
00360	Phenylalanine metabolism	10.81
00362	Benzoate degradation	10.00
00340	Histidine metabolism	10.00
00720	Carbon fixation pathways in prokaryotes	9.71
00920	Sulfur metabolism	9.52
00330	Arginine and proline metabolism	9.52
00310	Lysine degradation	9.30
03440	Homologous recombination	9.09
00380	Tryptophan metabolism	8.89
00970	Aminoacyl-tRNA biosynthesis	7.25
00903	Limonene and pinene degradation	7.14
00564	Glycerophospholipid metabolism	6.90
00410	beta-Alanine metabolism	6.45
04112	Cell cycle - Caulobacter	6.45
04212	Longevity regulating pathway - worm	5.36
00195	Photosynthesis	4.76
04931	Insulin resistance	4.05
04217	Necroptosis	2.08
