# Supplementary material for: Targeted RNA-Seq Reveals the M. tuberculosis Transcriptome from an In Vivo Infection Model
Source: Biology (Basel). 2021 Aug 31;10(9):848. doi: 10.3390/biology10090848 (PMC8467220; doi:10.3390/biology10090848)
Supplement: Supplementary file 1 [file biology-10-00848-s001.zip › TableS6_r1.pdf]

Table S6. M. tuberculosis expressed genes belonging to the predicted secretome.

Gen ID	Gene description
Rv3344c	PE_PGRS49 PE-PGR
Rv3512	PE_PGRS56 PE-PGRS
Rv0746	PE_PGRS9 PE-PGRS
Rv3345c	PE_PGRS50 PE-PGR
Rv0105c	uracil-DNA glycosylase
Rv2424c	Uncharacterised protein
Rv3053c	Chain A, Glutaredoxin Like Protein Nrdh
Rv3873	PPE68 PPE FAMILY
Rv0351	nucleotide exchange factor GrpE
Rv2270	lipoprotein lppN
Rv2784c	lipoprotein LppU
Rv0009	peptidyl-prolyl cis-trans isomerase
Rv0287	type VII secretion protein EsxS
Rv2442c	50S ribosomal protein L21
Rv3791	decaprenylphosphoryl-D-2-keto erythropentose reductase
Rv0830	SAM-dependent methyltransferase
Rv0281	SAM-dependent methyltransferase
Rv3035	PQQ enzyme repeat-containing protein
Rv0502	phospholipid/glycerol acyltransferase
Rv1087	PE_PGRS21 PE-PGRS
Rv2659c	integrase
Rv2518c	lipoprotein LppS
Rv3881c	type VII secretion system ESX-1 target EspB
Rv3685c	cytochrome P450
Rv0119	acyl-CoA synthetase
Rv1243c	PE_PGRS23 PE-PGRS
Rv2853	PE_PGRS48 PE-PGRS
Rv1266c	putative transmembrane serine/threonine-protein kinase H pknH
Rv0578c	PE_PGRS7 PE-PGRS
Rv0931c	serine/threonine protein kinase
Rv0932c	phosphate-binding protein
Rv3909	Conserved protein
Rv1664	polyketide synthase
Rv0598c	PIN domain-containing protein
Rv1917c	PPE34 PPE FAMILY
Rv3547	deazaflavin-dependent nitroreductase
Rv3418c	molecular chaperone GroES
Rv3034c	acetyltransferase
Rv1174c	hemophore-related protein
Rv0164	cyclase
Rv1274	lipoprotein lprB
Rv3350c	PPE56 PPE FAMILY
Rv2861c	type I methionyl aminopeptidase
Rv2544	lipoprotein lppB
Rv3344c	PE_PGRS49 PE-PGR
Rv3036c	DUF3298 domain-containing protein
Rv1714	oxidoreductase
Rv2585c	protein translocase subunit SecF
Rv1468c	PE_PGRS29 PE-PGR
Rv3878	secretion protein EspJ
Rv3502c	3-oxoacyl-ACP reductase
Rv1172c	PE12 PE FAMILY P
Rv1076	lipase lipU
Rv1768	PE_PGRS31 PE-PGRS
Rv0280	PPE3 PPE FAMILY P
Rv1971	MCE family protein
Rv1493	methylmalonyl-CoA mutase
Rv1702c	HNH endonuclease
Rv3106	NADPH:adrenodoxin oxidoreductase fprA (NADPH-ferredoxin reductase)
Rv2791c	transposase
Rv2577	purple acid phosphatase-related protein
Rv3763	lipoprotein LpqH
Rv2930	fatty-acid-CoA ligase fadD26
Rv2970c	lipase/esterase LIPN
Rv1275	DUF3558 domain-containing protein
Rv2220	glutamine synthetase 1
Rv3590c	PE_PGRS58 PE-PGR
Rv1984c	cutinase cfp21
Rv3202c	ATP-dependent DNA helicase
Rv0305c	PPE6 PPE FAMILY
Rv0278c	PE_PGRS3 PE-PGRS
Rv2587c	protein translocase subunit SecD
Rv0833	PE_PGRS13 PE-PGRS
Rv2905	lipoprotein LppW
Rv3634c	NAD-dependent epimerase/dehydratase family protein
Rv0755c	PPE12 PPE FAMILY
Rv3136	PPE51 PPE FAMILY
Rv0399c	D-alanyl-D-alanine carboxypeptidase
Rv2280	FAD-binding oxidoreductase
Rv2351c	Phospholipase C
Rv2264c	Conserved protein of uncharacterised function
Rv3159c	PPE53 PPE FAMILY
Rv3667	acetyl-coenzyme A synthetase
Rv0355c	PPE8 PPE FAMILY
Rv0101	non-ribosomal peptide synthetase
