# Supplementary material for: Targeted RNA-Seq Reveals the M. tuberculosis Transcriptome from an In Vivo Infection Model
Source: Biology (Basel). 2021 Aug 31;10(9):848. doi: 10.3390/biology10090848 (PMC8467220; doi:10.3390/biology10090848)
Supplement: Supplementary file 1 [file biology-10-00848-s001.zip › TableS7_r1.pdf]

Table S7. 15,677 most expressed mouse genes at day 21-post infection with M. tuberculosis.

Gene	GI	Chromosome	Gene name	Mean RPKM
gene48456	17708	NC_005089.1	COX1	4.24E+03
gene35175	20389	NC_000080.6	Sftpc	4.15E+03
gene26194	17105	NC_000076.6	Lyz2	4.02E+03
gene48475	17711	NC_005089.1	CYTB	2.32E+03
gene42193	22287	NC_000085.6	Scgb1a1	2.04E+03
gene48446	17716	NC_005089.1	ND1	1.92E+03
gene42207	14319	NC_000085.6	Fth1	1.80E+03
gene39665	14964	NC_000083.6	H2-D1	1.55E+03
gene15344	1E+08	NC_000072.6	Tpt1-ps3	1.30E+03
gene48450	17717	NC_005089.1	ND2	1.20E+03
gene34174	20387	NC_000080.6	Sftpa1	1.20E+03
gene41470	16149	NC_000084.6	Cd74	1.15E+03
gene23644	13627	NC_000075.6	Eef1a1	1.07E+03
gene4915	12010	NC_000068.7	B2m	1.07E+03
gene38332	1E+08	NC_000082.6	Gm9843	1.04E+03
gene39548	14972	NC_000083.6	H2-K1	1.01E+03
gene8228	1E+08	NC_000069.6	Mir6381	9.64E+02
gene39574	14960	NC_000083.6	H2-Aa	8.28E+02
gene48472	17721	NC_005089.1	ND5	8.00E+02
gene18971	1E+08	NC_000073.6	Hbb-bs	7.88E+02
gene48473	17722	NC_005089.1	ND6	7.71E+02
gene48468	17719	NC_005089.1	ND4	7.34E+02
gene39573	14961	NC_000083.6	H2-Ab1	7.27E+02
gene13369	11461	NC_000071.6	Actb	7.17E+02
gene14651	1E+08	NC_000072.6	Gm9794	7.00E+02
gene16913	11816	NC_000073.6	Apoe	6.90E+02
gene17937	14325	NC_000073.6	Ftl1	6.75E+02
gene5959	629957	NC_000068.7	Gm14303	6.72E+02
gene13049	1E+08	NC_000071.6	Rps16-ps2	6.42E+02
gene3340	16819	NC_000068.7	Lcn2	5.88E+02
gene3540	666531	NC_000068.7	Gm13456	5.74E+02
gene490	22167	NC_000067.6	Txn-ps1	5.70E+02
gene39614	14962	NC_000083.6	Cfb	5.34E+02
gene19892	546015	NC_000073.6	Gm5905	4.94E+02
gene7930	1E+08	NC_000069.6	Gm4332	4.89E+02
gene21722	1E+08	NC_000074.6	Gm10073	4.71E+02
gene36379	110454	NC_000081.6	Ly6a	4.56E+02
gene19882	66141	NC_000073.6	Ifitm3	4.52E+02
gene39721	15040	NC_000083.6	H2-T23	4.50E+02
gene1289	1E+08	NC_000067.6	Gm15427	4.41E+02
gene27188	621823	NC_000077.6	Psme2b	4.21E+02
gene39577	1E+08	NC_000083.6	H2-Ea-ps	4.19E+02
gene42329	17476	NC_000085.6	Mpeg1	4.16E+02
gene29386	12409	NC_000077.6	Cbr2	4.08E+02
gene48442	17724	NC_005089.1	Rnr1	4.04E+02

gene27004	15122	NC_000077.6	Hba-a1	4.03E+02
gene17926	1E+08	NC_000073.6	Rpl14-ps1	4.01E+02
gene48444	17725	NC_005089.1	Rnr2	3.96E+02
gene17996	20210	NC_000073.6	Saa3	3.96E+02
gene39592	11596	NC_000083.6	Ager	3.90E+02
gene26458	67945	NC_000076.6	Rpl41	3.81E+02
gene25670	13629	NC_000076.6	Eef2	3.67E+02
gene24123	14775	NC_000075.6	Gpx1	3.64E+02
gene11099	1E+08	NC_000071.6	Gm15772	3.53E+02
gene39575	14969	NC_000083.6	H2-Eb1	3.49E+02
gene16085	664868	NC_000072.6	Rpl38-ps2	3.46E+02
gene21592	667618	NC_000074.6	Gm8730	3.44E+02
gene764	1E+08	NC_000067.6	Rpl10a-ps1	3.41E+02
gene39672	15015	NC_000083.6	H2-Q4	3.27E+02
gene48477	17739	NC_005089.1	TrnP	3.21E+02
gene39875	110183	NC_000083.6	Rn18s-rs5	3.18E+02
gene24308	668829	NC_000075.6	Gm9385	3.05E+02
gene20289	1E+08	NC_000074.6	Rpl19-ps11	3.03E+02
gene36486	26961	NC_000081.6	Rpl8	3.02E+02
gene12185	20309	NC_000071.6	Cxcl15	2.95E+02
gene19957	13033	NC_000073.6	Ctsd	2.92E+02
gene23328	56040	NC_000075.6	Rplp1	2.90E+02
gene27006	110257	NC_000077.6	Hba-a2	2.87E+02
gene1936	1E+08	NC_000067.6	Gm2000	2.86E+02
gene36374	17069	NC_000081.6	Ly6e	2.84E+02
gene24290	1E+08	NC_000075.6	Rps27rt	2.81E+02
gene8937	1E+08	NC_000070.6	Gm12481	2.80E+02
gene1658	12654	NC_000067.6	Chil1	2.76E+02
gene20700	620772	NC_000074.6	Gm6180	2.74E+02
gene19314	1E+08	NC_000073.6	Gm15500	2.74E+02
gene5351	13010	NC_000068.7	Cst3	2.72E+02
gene21317	12317	NC_000074.6	Calr	2.69E+02
gene39946	15516	NC_000083.6	Hsp90ab1	2.68E+02
gene27874	18643	NC_000077.6	Pfn1	2.65E+02
gene25045	664781	NC_000076.6	Gm7338	2.64E+02
gene37238	1E+08	NC_000081.6	Rpl39-ps	2.60E+02
gene5428	1E+08	NC_000068.7	Rps15a-ps7	2.60E+02
gene6723	621155	NC_000069.6	Gm6204	2.57E+02
gene10494	12259	NC_000070.6	C1qa	2.56E+02
gene37670	666274	NC_000082.6	Rps10-ps2	2.50E+02
gene39677	110557	NC_000083.6	H2-Q6	2.49E+02
gene22212	1E+08	NC_000074.6	Gm21399	2.43E+02
gene37918	ene=LOC1	NC_000082.6	LOC102638850	2.40E+02
gene30702	628277	NC_000078.6	Gm6863	2.39E+02
gene3402	14828	NC_000068.7	Hspa5	2.36E+02
gene2924	22352	NC_000068.7	Vim	2.33E+02
gene15101	1E+08	NC_000072.6	Gm10443	2.33E+02
gene6411	665189	NC_000069.6	Gm7536	2.29E+02

gene23225	1E+08	NC_000075.6	Gm6166	2.28E+02
gene14660	667310	NC_000072.6	Gm8566	2.26E+02
gene39569	16913	NC_000083.6	Psmb8	2.24E+02
gene42250	107350	NC_000085.6	AW112010	2.21E+02
gene35541	621054	NC_000080.6	Tpm3-rs7	2.20E+02
gene37601	12741	NC_000082.6	Cldn5	2.19E+02
gene25185	19156	NC_000076.6	Psap	2.17E+02
gene27595	76293	NC_000077.6	Mfap4	2.15E+02
gene1775	545369	NC_000067.6	Gm5835	2.13E+02
gene10408	23833	NC_000070.6	Cd52	2.10E+02
gene32963	634386	NC_000079.6	Ftl1-ps1	2.06E+02
gene25613	20054	NC_000076.6	Rps15	2.06E+02
gene11136	727711	NC_000071.6	Gm15459	2.05E+02
gene39537	1E+08	NC_000083.6	Gm19412	2.01E+02
gene9879	ene=LOC1	NC_000070.6	LOC102637129	2.01E+02
gene32852	674597	NC_000079.6	Gm9625	1.97E+02
gene41090	12475	NC_000084.6	Cd14	1.96E+02
gene24460	20282	NC_000075.6	Scp2-ps2	1.95E+02
gene21775	15439	NC_000074.6	Hp	1.93E+02
gene17901	27207	NC_000073.6	Rps11	1.93E+02
gene25224	432466	NC_000076.6	Gm5424	1.89E+02
gene14812	384419	NC_000072.6	Igkv8-30	1.87E+02
gene25582	ene=LOC1	NC_000076.6	LOC102632476	1.86E+02
gene42088	54683	NC_000085.6	Prdx5	1.85E+02
gene37725	666489	NC_000082.6	Gm8130	1.81E+02
gene15515	666634	NC_000072.6	Gm8203	1.80E+02
gene8313	1E+08	NC_000069.6	Gm10288	1.79E+02
gene19342	1E+08	NC_000073.6	Gm4366	1.78E+02
gene7556	56338	NC_000069.6	Txnip	1.77E+02
gene40226	12266	NC_000083.6	C3	1.76E+02
gene3905	ene;gene=L	NC_000068.7	LOC105244208	1.76E+02
gene14013	1E+08	NC_000072.6	Atp6v0c-ps2	1.76E+02
gene14904	20388	NC_000072.6	Sftpb	1.73E+02
gene7339	1E+08	NC_000069.6	Gm4202	1.73E+02
gene508	433297	NC_000067.6	Gm5526	1.71E+02
gene18802	22228	NC_000073.6	Ucp2	1.71E+02
gene8337	1E+08	NC_000069.6	Rpsa-ps10	1.68E+02
gene35693	1E+08	NC_000081.6	Gm10250	1.64E+02
gene26195	17110	NC_000076.6	Lyz1	1.64E+02
gene27791	12514	NC_000077.6	Cd68	1.62E+02
gene30838	20715	NC_000078.6	Serpina3g	1.60E+02
gene23161	19350	NC_000075.6	Rab7-ps1	1.60E+02
gene10493	12262	NC_000070.6	C1qc	1.59E+02
gene27157	620678	NC_000077.6	Gm12174	1.58E+02
gene10670	433776	NC_000070.6	Gm13050	1.57E+02
gene27619	22187	NC_000077.6	Ubb	1.57E+02
gene19878	80876	NC_000073.6	Ifitm2	1.56E+02
gene14558	633683	NC_000072.6	Rps15-ps2	1.55E+02

gene16153	1E+08	NC_000072.6	Rps25-ps1	1.55E+02
gene29368	192662	NC_000077.6	Arhgdia	1.54E+02
gene40838	665509	NC_000084.6	Gm7665	1.53E+02
gene697	13674	NC_000067.6	Eif4a-ps4	1.52E+02
gene27171	14694	NC_000077.6	Rack1	1.52E+02
gene12222	17329	NC_000071.6	Cxcl9	1.51E+02
gene21021	16478	NC_000074.6	Jund	1.51E+02
gene16274	76846	NC_000073.6	Rps9	1.50E+02
gene11327	625174	NC_000071.6	Gm6560	1.49E+02
gene15112	23983	NC_000072.6	Pcbp1	1.49E+02
gene20978	665649	NC_000074.6	Gm7730	1.49E+02
gene39006	433073	NC_000083.6	Gm5492	1.48E+02
gene31440	674228	NC_000079.6	Gm9616	1.48E+02
gene5853	12608	NC_000068.7	Cebpb	1.47E+02
gene6583	1E+08	NC_000069.6	Rps23-ps1	1.44E+02
gene42881	20249	NC_000085.6	Scd1	1.41E+02
gene7108	1E+08	NC_000069.6	Gm3788	1.41E+02
gene7475	17210	NC_000069.6	Mcl1	1.40E+02
gene41259	117158	NC_000084.6	Scgb3a2	1.39E+02
gene11301	14190	NC_000071.6	Fgl2	1.33E+02
gene7285	20200	NC_000069.6	S100a6	1.33E+02
gene16046	17313	NC_000072.6	Mgp	1.32E+02
gene3464	624088	NC_000068.7	Gm13436	1.31E+02
gene5119	12616	NC_000068.7	Cenpb	1.30E+02
gene16014	1E+08	NC_000072.6	Dynlt1-ps1	1.30E+02
gene18762	27050	NC_000073.6	Rps3	1.30E+02
gene21074	69550	NC_000074.6	Bst2	1.29E+02
gene39700	22154	NC_000083.6	Tubb5	1.28E+02
gene9674	1E+08	NC_000070.6	Gm12715	1.27E+02
gene20110	669429	NC_000074.6	Gm1840	1.27E+02
gene28742	1E+08	NC_000077.6	Gm11560	1.26E+02
gene22943	1E+08	NC_000075.6	Gm10080	1.25E+02
gene23380	67891	NC_000075.6	Rpl4	1.24E+02
gene15720	69202	NC_000072.6	Ptms	1.24E+02
gene10410	73723	NC_000070.6	Sh3bgrl3	1.24E+02
gene41766	545267	NC_000084.6	Gm5823	1.23E+02
gene16647	20103	NC_000073.6	Rps5	1.21E+02
gene41933	19045	NC_000085.6	Ppp1ca	1.21E+02
gene42012	12631	NC_000085.6	Cfl1	1.20E+02
gene34177	20390	NC_000080.6	Sftpd	1.20E+02
gene39798	24108	NC_000083.6	Ubd	1.20E+02
gene31136	12925	NC_000078.6	Crip1	1.20E+02
gene21332	16477	NC_000074.6	Junb	1.19E+02
gene30742	12313	NC_000078.6	Calm1	1.18E+02
gene15818	652986	NC_000072.6	Rpl18-ps2	1.18E+02
gene1091	620213	NC_000067.6	Gm6136	1.18E+02
gene14834	108022	NC_000072.6	Igkv6-15	1.17E+02
gene34784	667952	NC_000080.6	Gm8894	1.17E+02

gene20075	664922	NC_000074.6	Gm7407	1.17E+02
gene39280	666609	NC_000083.6	Gm8186	1.16E+02
gene29509	11852	NC_000078.6	Rhob	1.14E+02
gene40162	76905	NC_000083.6	Lrg1	1.14E+02
gene17155	1E+08	NC_000073.6	Gm9844	1.14E+02
gene31694	1E+08	NC_000079.6	Gm11273	1.14E+02
gene36675	Gene;gene	NC_000081.6	LOC102634709	1.14E+02
gene5582	19018	NC_000068.7	Scand1	1.14E+02
gene8011	1E+08	NC_000069.6	Gm4617	1.13E+02
gene25522	13014	NC_000076.6	Cstb	1.13E+02
gene27435	16145	NC_000077.6	Igtp	1.12E+02
gene27385	667832	NC_000077.6	Gm12231	1.10E+02
gene3800	1E+08	NC_000068.7	Rpl9-ps7	1.10E+02
gene39538	21356	NC_000083.6	Tapbp	1.09E+02
gene28284	20305	NC_000077.6	Ccl6	1.09E+02
gene36908	667682	NC_000081.6	Rpl31-ps8	1.08E+02
gene28881	14824	NC_000077.6	Grn	1.08E+02
gene29205	15081	NC_000077.6	H3f3b	1.07E+02
gene34793	19186	NC_000080.6	Psme1	1.07E+02
gene28234	20307	NC_000077.6	Ccl8	1.07E+02
gene1686	1E+08	NC_000067.6	Gm4204	1.07E+02
gene34314	218963	NC_000080.6	Gm1821	1.07E+02
gene18970	1E+08	NC_000073.6	Hbb-bt	1.06E+02
gene21700	19171	NC_000074.6	Psmb10	1.04E+02
gene30102	ene=LOC1	NC_000078.6	LOC102640946	1.04E+02
gene27378	14778	NC_000077.6	Gpx3	1.03E+02
gene8460	666586	NC_000070.6	Gm11808	1.03E+02
gene36376	57248	NC_000081.6	Ly6i	1.03E+02
gene11373	1E+08	NC_000071.6	Gm10221	1.02E+02
gene13129	12739	NC_000071.6	Cldn3	1.01E+02
gene35003	13030	NC_000080.6	Ctsb	1.01E+02
gene30503	432676	NC_000078.6	Gm5436	1.00E+02
gene7473	668457	NC_000069.6	Rps10-ps1	9.99E+01
gene7468	13040	NC_000069.6	Ctss	9.97E+01
gene26425	11947	NC_000076.6	Atp5b	9.92E+01
gene2270	21346	NC_000067.6	Tagln2	9.82E+01
gene2108	1E+08	NC_000067.6	Gm16418	9.81E+01
gene12181	20311	NC_000071.6	Cxcl5	9.74E+01
gene18637	1E+08	NC_000073.6	Rps13-ps2	9.72E+01
gene29763	15902	NC_000078.6	Id2	9.71E+01
gene19588	791073	NC_000073.6	Mir762	9.70E+01
gene36438	66168	NC_000081.6	Grina	9.69E+01
gene18532	666170	NC_000073.6	Gm7964	9.69E+01
gene12224	15945	NC_000071.6	Cxcl10	9.68E+01
gene6010	64138	NC_000068.7	Ctsz	9.51E+01
gene17903	22121	NC_000073.6	Rpl13a	9.44E+01
gene15505	19951	NC_000072.6	Rpl32	9.39E+01
gene21751	1E+08	NC_000074.6	Rps26-ps1	9.36E+01

gene36380	17067	NC_000081.6	Ly6c1	9.29E+01
gene27185	15944	NC_000077.6	Irgm1	9.16E+01
gene14356	66058	NC_000072.6	Tmem176a	9.15E+01
gene7288	20201	NC_000069.6	S100a8	8.92E+01
gene39712	1E+08	NC_000083.6	Gm9840	8.85E+01
gene22636	1E+08	NC_000075.6	Gm10698	8.85E+01
gene15867	56644	NC_000072.6	Clec7a	8.84E+01
gene14868	619547	NC_000072.6	Rpl34-ps1	8.80E+01
gene41457	60440	NC_000084.6	Iigp1	8.79E+01
gene4914	67578	NC_000068.7	Patl2	8.79E+01
gene27827	56486	NC_000077.6	Gabarap	8.77E+01
gene41311	628438	NC_000084.6	Hspe1-rs1	8.72E+01
gene21028	65972	NC_000074.6	Ifi30	8.72E+01
gene13667	1E+08	NC_000072.6	Gm9835	8.69E+01
gene35375	1E+08	NC_000080.6	Rps3a2	8.66E+01
gene11660	67568	NC_000071.6	Mrfap1	8.61E+01
gene729	55949	NC_000067.6	Eef1b2	8.54E+01
gene29289	19039	NC_000077.6	Lgals3bp	8.53E+01
gene24194	1E+08	NC_000075.6	Mir6236	8.51E+01
gene35671	20363	NC_000081.6	Sepp1	8.42E+01
gene30518	67963	NC_000078.6	Npc2	8.30E+01
gene27347	16362	NC_000077.6	Irf1	8.27E+01
gene12448	17472	NC_000071.6	Gbp4	8.24E+01
gene39722	15039	NC_000083.6	H2-T22	8.19E+01
gene39060	gene=LOC1	NC_000083.6	LOC102633627	8.19E+01
gene42002	66556	NC_000085.6	Drap1	8.18E+01
gene6795	28146	NC_000069.6	Serp1	8.16E+01
gene16032	1E+08	NC_000072.6	Rpl36a-ps3	8.16E+01
gene37402	12703	NC_000082.6	Socs1	8.14E+01
gene42114	12868	NC_000085.6	Cox8a	8.14E+01
gene40970	626327	NC_000084.6	Gm6665	8.12E+01
gene1846	ene=LOC1	NC_000067.6	LOC102640575	8.12E+01
gene32024	1E+08	NC_000079.6	Gm11361	8.11E+01
gene48474	17729	NC_005089.1	TrnE	8.10E+01
gene33229	ene=LOC1	NC_000079.6	LOC101055909	8.08E+01
gene23877	19034	NC_000075.6	Gm10123	8.01E+01
gene3074	227613	NC_000068.7	Tubb4b	7.98E+01
gene48448	17740	NC_005089.1	TrnQ	7.97E+01
gene6134	66481	NC_000068.7	Rps21	7.97E+01
gene27227	68662	NC_000077.6	Scgb3a1	7.96E+01
gene135	19989	NC_000067.6	Rpl7	7.87E+01
gene27859	66102	NC_000077.6	Cxcl16	7.86E+01
gene5749	67701	NC_000068.7	Wfdc2	7.82E+01
gene35221	16432	NC_000080.6	Itm2b	7.75E+01
gene7429	19172	NC_000069.6	Psmb4	7.72E+01
gene24592	66125	NC_000076.6	Sf3b5	7.72E+01
gene32562	77134	NC_000079.6	Hnrnpa0	7.72E+01
gene21181	1E+08	NC_000074.6	Gm2225	7.68E+01

gene6780	623114	NC_000069.6	Gm6394	7.66E+01
gene14355	65963	NC_000072.6	Tmem176b	7.66E+01
gene14498	21743	NC_000072.6	Inmt	7.64E+01
gene12150	16069	NC_000071.6	Jchain	7.62E+01
gene39567	16912	NC_000083.6	Psmb9	7.58E+01
gene41468	20044	NC_000084.6	Rps14	7.57E+01
gene24878	215900	NC_000076.6	Fam26f	7.55E+01
gene1531	1E+08	NC_000067.6	Rpl28-ps1	7.53E+01
gene39655	16994	NC_000083.6	Ltb	7.50E+01
gene18004	668838	NC_000073.6	Gm9392	7.48E+01
gene27820	276770	NC_000077.6	Eif5a	7.44E+01
gene22452	15894	NC_000075.6	Icam1	7.37E+01
gene39545	630499	NC_000083.6	H2-K2	7.33E+01
gene37073	22142	NC_000081.6	Tuba1a	7.28E+01
gene34353	606520	NC_000080.6	Gm6055	7.27E+01
gene23699	619900	NC_000075.6	Rps27a-ps2	7.26E+01
gene22014	13057	NC_000074.6	Cyba	7.20E+01
gene3423	654354	NC_000068.7	Gm13443	7.13E+01
gene39568	21354	NC_000083.6	Tap1	7.08E+01
gene16239	1E+08	NC_000073.6	Gm3375	7.05E+01
gene27394	20692	NC_000077.6	Sparc	7.05E+01
gene17164	14470	NC_000073.6	Rabac1	7.03E+01
gene26616	380683	NC_000077.6	Sec14l3	7.03E+01
gene27436	54396	NC_000077.6	Irgm2	7.02E+01
gene25565	12215	NC_000076.6	Bsg	7.01E+01
gene25776	432482	NC_000076.6	Gm5425	7.01E+01
gene20202	16783	NC_000074.6	Lamp1	6.98E+01
gene7187	545536	NC_000069.6	Gm10704	6.98E+01
gene37097	110213	NC_000081.6	Tmbim6	6.94E+01
gene10492	12260	NC_000070.6	C1qb	6.92E+01
gene9959	54325	NC_000070.6	Elovl1	6.91E+01
gene19520	12721	NC_000073.6	Coro1a	6.82E+01
gene37146	23994	NC_000081.6	Dazap2	6.82E+01
gene29367	18453	NC_000077.6	P4hb	6.81E+01
gene17592	12606	NC_000073.6	Cebpa	6.80E+01
gene28280	20304	NC_000077.6	Ccl5	6.77E+01
gene12167	665733	NC_000071.6	Eif5al3-ps	6.76E+01
gene9558	16476	NC_000070.6	Jun	6.75E+01
gene17244	13107	NC_000073.6	Cyp2f2	6.74E+01
gene27434	631323	NC_000077.6	Gm12250	6.64E+01
gene5774	19025	NC_000068.7	Ctsa	6.62E+01
gene18851	16068	NC_000073.6	Il18bp	6.62E+01
gene14925	19240	NC_000072.6	Tmsb10	6.59E+01
gene36570	14958	NC_000081.6	H1f0	6.58E+01
gene19899	54123	NC_000073.6	Irf7	6.55E+01
gene15710	21991	NC_000072.6	Tpi1	6.52E+01
gene25827	22027	NC_000076.6	Hsp90b1	6.51E+01
gene23371	1E+08	NC_000075.6	Gm18541	6.51E+01

gene19835	622335	NC_000073.6	Gm6314	6.50E+01
gene9526	620016	NC_000070.6	Gm12669	6.50E+01
gene27169	432554	NC_000077.6	Gm12183	6.48E+01
gene35262	22070	NC_000080.6	Tpt1	6.45E+01
gene4033	12258	NC_000068.7	Serping1	6.42E+01
gene30215	11845	NC_000078.6	Arf6	6.42E+01
gene40410	668773	NC_000083.6	Gm9349	6.40E+01
gene39674	15016	NC_000083.6	H2-Q5	6.35E+01
gene28770	1E+08	NC_000077.6	Gm10039	6.33E+01
gene25977	12226	NC_000076.6	Btg1	6.33E+01
gene36564	16852	NC_000081.6	Lgals1	6.32E+01
gene39680	15007	NC_000083.6	H2-Q10	6.31E+01
gene30090	18035	NC_000078.6	Nfkbia	6.30E+01
gene6855	666967	NC_000069.6	Gm8388	6.29E+01
gene5737	20568	NC_000068.7	Slpi	6.25E+01
gene41748	11946	NC_000084.6	Atp5a1	6.23E+01
gene17775	72310	NC_000073.6	Nkg7	6.21E+01
gene7555	1E+08	NC_000069.6	Gm15441	6.19E+01
gene1324	633295	NC_000067.6	Tdpx-ps1	6.18E+01
gene12199	665864	NC_000071.6	Gm7832	6.13E+01
gene28817	52469	NC_000077.6	Coa3	6.12E+01
gene29536	238074	NC_000078.6	Gm4928	6.09E+01
gene7217	17829	NC_000069.6	Muc1	6.07E+01
gene4004	545429	NC_000068.7	Gm13680	6.06E+01
gene19266	13690	NC_000073.6	Eif4g2	6.03E+01
gene8227	14469	NC_000069.6	Gbp2	6.03E+01
gene31134	68337	NC_000078.6	Crip2	6.01E+01
gene10440	27981	NC_000070.6	Rsrp1	5.98E+01
gene9314	1E+08	NC_000070.6	Gm11223	5.97E+01
gene39629	110956	NC_000083.6	D17H6S56E-5	5.96E+01
gene3871	228033	NC_000068.7	Atp5g3	5.95E+01
gene21562	1E+08	NC_000074.6	Sap18b	5.95E+01
gene32626	13024	NC_000079.6	Ctla2a	5.93E+01
gene2213	14127	NC_000067.6	Fcer1g	5.92E+01
gene3538	1E+08	NC_000068.7	Gm13453	5.92E+01
gene31941	50708	NC_000079.6	Hist1h1c	5.88E+01
gene7896	1E+08	NC_000069.6	Gm9761	5.86E+01
gene42626	1E+08	NC_000085.6	Rpl9-ps6	5.84E+01
gene25796	666974	NC_000076.6	Gm8394	5.81E+01
gene39733	69717	NC_000083.6	Gm10499	5.79E+01
gene9937	433749	NC_000070.6	Gm12844	5.78E+01
gene29752	629383	NC_000078.6	Gm6969	5.76E+01
gene38096	637273	NC_000082.6	Gm7204	5.74E+01
gene39698	15937	NC_000083.6	Ier3	5.72E+01
gene32980	1E+08	NC_000079.6	Gm4149	5.71E+01
gene10254	17357	NC_000070.6	Marcksl1	5.65E+01
gene12756	11837	NC_000071.6	Rplp0	5.65E+01
gene11047	1E+08	NC_000070.6	Isg15	5.63E+01

gene35483	621103	NC_000080.6	Gm6201	5.59E+01
gene14918	22134	NC_000072.6	Tgoln1	5.53E+01
gene12779	433943	NC_000071.6	Gstm2-ps1	5.52E+01
gene5992	1E+08	NC_000068.7	Mir6340	5.51E+01
gene9649	665362	NC_000070.6	Gm12791	5.49E+01
gene40673	383374	NC_000084.6	Gm10557	5.49E+01
gene21961	12857	NC_000074.6	Cox4i1	5.46E+01
gene5713	68386	NC_000068.7	0610039K10Rik	5.44E+01
gene3019	ene=LOC1	NC_000068.7	LOC102637566	5.41E+01
gene17416	22177	NC_000073.6	Tyrobp	5.40E+01
gene15662	56619	NC_000072.6	Clec4e	5.39E+01
gene32470	67044	NC_000079.6	Higd2a	5.38E+01
gene21117	16598	NC_000074.6	Klf2	5.37E+01
gene10476	15903	NC_000070.6	Id3	5.36E+01
gene27845	52898	NC_000077.6	Rnasek	5.35E+01
gene14336	71660	NC_000072.6	Rarres2	5.34E+01
gene19481	244214	NC_000073.6	Gm4973	5.33E+01
gene33338	667518	NC_000079.6	Gm8680	5.30E+01
gene13222	14693	NC_000071.6	Gnb2	5.29E+01
gene19527	11674	NC_000073.6	Aldoa	5.29E+01
gene22902	545339	NC_000075.6	Gm5831	5.26E+01
gene35491	1E+08	NC_000080.6	Gm10076	5.25E+01
gene1540	620659	NC_000067.6	Gm6170	5.21E+01
gene17726	668579	NC_000073.6	Cd9-ps	5.20E+01
gene25592	12798	NC_000076.6	Cnn2	5.19E+01
gene36628	11911	NC_000081.6	Atf4	5.16E+01
gene41927	23789	NC_000085.6	Coro1b	5.14E+01
gene11813	20531	NC_000071.6	Slc34a2	5.12E+01
gene41873	16343	NC_000084.6	Eif3s6-ps2	5.12E+01
gene37501	12609	NC_000082.6	Cebpd	5.09E+01
gene17388	1E+08	NC_000073.6	Gm10169	5.09E+01
gene27285	15384	NC_000077.6	Hnrnpab	5.07E+01
gene6013	67126	NC_000068.7	Atp5e	5.05E+01
gene42490	433229	NC_000085.6	Gm5514	5.02E+01
gene37106	11830	NC_000081.6	Aqp5	5.02E+01
gene16739	666407	NC_000073.6	Mrip-ps	5.02E+01
gene28814	54409	NC_000077.6	Ramp2	4.99E+01
gene24226	54199	NC_000075.6	Ccrl2	4.98E+01
gene7722	624251	NC_000069.6	Gm6485	4.97E+01
gene24769	14219	NC_000076.6	Ctgf	4.96E+01
gene18479	623286	NC_000073.6	Gm6415	4.95E+01
gene31061	21928	NC_000078.6	Tnfaip2	4.94E+01
gene29273	20973	NC_000077.6	Syngr2	4.94E+01
gene40870	1E+08	NC_000084.6	Gm10269	4.94E+01
gene14910	22320	NC_000072.6	Vamp8	4.91E+01
gene38394	622000	NC_000082.6	Gm6278	4.89E+01
gene13866	11844	NC_000072.6	Arf5	4.89E+01
gene27864	19175	NC_000077.6	Psmb6	4.89E+01

gene9583	620883	NC_000070.6	Gm12696	4.87E+01
gene26464	27370	NC_000076.6	Rps26	4.87E+01
gene20742	11739	NC_000074.6	Slc25a4	4.85E+01
gene11745	545747	NC_000071.6	Gm5865	4.85E+01
gene27241	18412	NC_000077.6	Sqstm1	4.84E+01
gene12746	12861	NC_000071.6	Cox6a1	4.84E+01
gene8026	ene=LOC1	NC_000069.6	LOC108168897	4.84E+01
gene18110	620782	NC_000073.6	Gm6181	4.83E+01
gene39728	667803	NC_000083.6	H2-T-ps	4.82E+01
gene28767	20918	NC_000077.6	Eif1	4.82E+01
gene20795	1E+08	NC_000074.6	Gm9892	4.82E+01
gene5998	1E+08	NC_000068.7	Atp5k-ps2	4.77E+01
gene34031	69019	NC_000080.6	Spcs1	4.77E+01
gene5543	66734	NC_000068.7	Map1lc3a	4.77E+01
gene19918	12476	NC_000073.6	Cd151	4.76E+01
gene25703	14797	NC_000076.6	Aes	4.75E+01
gene40351	668724	NC_000083.6	Gm9320	4.74E+01
gene11798	20657	NC_000071.6	Sod3	4.72E+01
gene8226	14468	NC_000069.6	Gbp2b	4.72E+01
gene31604	22336	NC_000079.6	Vdac3-ps1	4.68E+01
gene37702	1E+08	NC_000082.6	Gm15776	4.68E+01
gene10673	621961	NC_000070.6	Anp32b-ps1	4.66E+01
gene22515	625409	NC_000075.6	Gm6581	4.65E+01
gene16041	232440	NC_000072.6	H2afj	4.65E+01
gene4899	67693	NC_000068.7	Hypk	4.64E+01
gene9897	58810	NC_000070.6	Akr1a1	4.64E+01
gene14667	56753	NC_000072.6	Tacstd2	4.62E+01
gene15702	14790	NC_000072.6	Grcc10	4.62E+01
gene30839	546546	NC_000078.6	Serpina3h	4.61E+01
gene8224	55932	NC_000069.6	Gbp3	4.58E+01
gene10969	67808	NC_000070.6	Tprgl	4.57E+01
gene7753	1E+08	NC_000069.6	Gm4540	4.57E+01
gene40357	67268	NC_000083.6	Myl12a	4.56E+01
gene3100	69454	NC_000068.7	Clic3	4.52E+01
gene4814	ene=LOC1	NC_000068.7	LOC102636129	4.51E+01
gene25415	17319	NC_000076.6	Mif	4.49E+01
gene9946	114143	NC_000070.6	Atp6v0b	4.49E+01
gene5335	21824	NC_000068.7	Thbd	4.48E+01
gene28052	654359	NC_000077.6	Gm12338	4.44E+01
gene19522	26417	NC_000073.6	Mapk3	4.43E+01
gene27447	624814	NC_000077.6	Gm12251	4.41E+01
gene14776	667683	NC_000072.6	Igkv4-59	4.40E+01
gene3881	1E+08	NC_000068.7	Mrpl23-ps1	4.40E+01
gene8225	1.1E+08	NC_000069.6	Gm40158	4.37E+01
gene3331	1E+08	NC_000068.7	Gm13611	4.36E+01
gene23433	19035	NC_000075.6	Ppib	4.36E+01
gene21669	67971	NC_000074.6	Tppp3	4.35E+01
gene4895	14827	NC_000068.7	Pdia3	4.34E+01

gene38105	57262	NC_000082.6	Retnla	4.30E+01
gene29519	17775	NC_000078.6	Laptm4a	4.30E+01
gene23203	102462	NC_000075.6	Imp3	4.29E+01
gene9440	68441	NC_000070.6	Rraga	4.29E+01
gene42568	627788	NC_000085.6	Gm6788	4.29E+01
gene12956	80885	NC_000071.6	Hcar2	4.29E+01
gene8454	67427	NC_000070.6	Rps20	4.28E+01
gene28990	67803	NC_000077.6	Limd2	4.28E+01
gene8747	665367	NC_000070.6	Gm12350	4.28E+01
gene12074	433912	NC_000071.6	Rpl7-ps7	4.28E+01
gene11166	1E+08	NC_000071.6	Gm15610	4.27E+01
gene8728	108767	NC_000070.6	Pnrc1	4.24E+01
gene36909	56645	NC_000081.6	Tcea1-ps1	4.24E+01
gene37072	22143	NC_000081.6	Tuba1b	4.23E+01
gene5062	668896	NC_000068.7	Spcs2-ps	4.22E+01
gene15	619596	NC_000067.6	Gm6085	4.22E+01
gene30206	217664	NC_000078.6	Mgat2	4.22E+01
gene29094	667003	NC_000077.6	Gm11675	4.21E+01
gene15723	30853	NC_000072.6	Mlf2	4.20E+01
gene26666	22433	NC_000077.6	Xbp1	4.20E+01
gene19519	66162	NC_000073.6	Bola2	4.20E+01
gene9945	=Gene;ger	NC_000070.6	LOC108168974	4.18E+01
gene5712	26943	NC_000068.7	Serinc3	4.18E+01
gene39566	14999	NC_000083.6	H2-DMb1	4.18E+01
gene23434	382083	NC_000075.6	Snx22	4.17E+01
gene26140	382423	NC_000076.6	Atxn7l3b	4.17E+01
gene31484	23849	NC_000079.6	Klf6	4.14E+01
gene39570	21355	NC_000083.6	Tap2	4.13E+01
gene20023	1E+08	NC_000073.6	Bc1	4.13E+01
gene6818	666756	NC_000069.6	Gm8276	4.11E+01
gene18634	13032	NC_000073.6	Ctsc	4.11E+01
gene42131	433217	NC_000085.6	Gm16437	4.11E+01
gene25757	216185	NC_000076.6	Gm4799	4.11E+01
gene34736	65107	NC_000080.6	Lrp10	4.10E+01
gene7633	17114	NC_000069.6	M6pr-ps	4.09E+01
gene2003	11758	NC_000067.6	Prdx6	4.08E+01
gene15003	11993	NC_000072.6	Aup1	4.08E+01
gene37534	64136	NC_000082.6	Sdf2l1	4.07E+01
gene1196	665766	NC_000067.6	Gm7776	4.05E+01
gene15692	50908	NC_000072.6	C1s1	4.04E+01
gene7061	24088	NC_000069.6	Tlr2	4.04E+01
gene41921	52004	NC_000085.6	Cdk2ap2	4.03E+01
gene1794	639456	NC_000067.6	Gm7266	4.03E+01
gene12293	55985	NC_000071.6	Cxcl13	4.02E+01
gene42027	17826	NC_000085.6	Fam89b	4.01E+01
gene42508	625193	NC_000085.6	Gm6563	4.00E+01
gene29247	20382	NC_000077.6	Srsf2	3.99E+01
gene31028	629595	NC_000078.6	Gm6988	3.98E+01

gene20524	1E+08	NC_000074.6	Nudc-ps1	3.96E+01
gene29280	12702	NC_000077.6	Socs3	3.95E+01
gene40547	12193	NC_000083.6	Zfp36l2	3.95E+01
gene18759	12406	NC_000073.6	Serpinh1	3.94E+01
gene30130	66864	NC_000078.6	Clec14a	3.94E+01
gene37229	66151	NC_000081.6	Prr13	3.93E+01
gene27809	69186	NC_000077.6	Tmem256	3.93E+01
gene20017	677315	NC_000073.6	Gm9711	3.93E+01
gene1911	15939	NC_000067.6	Ier5	3.93E+01
gene12466	1E+08	NC_000071.6	Rps15a-ps5	3.92E+01
gene39634	114584	NC_000083.6	Clic1	3.92E+01
gene1863	67771	NC_000067.6	Arpc5	3.91E+01
gene28762	666128	NC_000077.6	Gm11598	3.91E+01
gene21151	666211	NC_000074.6	Gm7984	3.91E+01
gene31043	1E+08	NC_000078.6	Mpc1-ps	3.91E+01
gene40451	625054	NC_000083.6	Gm6548	3.91E+01
gene37535	623568	NC_000082.6	Gm6440	3.90E+01
gene15152	12785	NC_000072.6	Cnbp	3.90E+01
gene7321	1E+08	NC_000069.6	Gm9774	3.90E+01
gene12069	1E+08	NC_000071.6	Gm2199	3.88E+01
gene42199	18540	NC_000085.6	Pcna-ps2	3.88E+01
gene24065	74840	NC_000075.6	Manf	3.87E+01
gene12328	735265	NC_000071.6	Mir703	3.87E+01
gene7877	13609	NC_000069.6	S1pr1	3.87E+01
gene21308	15936	NC_000074.6	Ier2	3.86E+01
gene41489	667279	NC_000084.6	Rps2-ps10	3.84E+01
gene4964	58521	NC_000068.7	Eid1	3.82E+01
gene25886	18674	NC_000076.6	Slc25a3	3.81E+01
gene30199	665967	NC_000078.6	Gm7868	3.80E+01
gene28830	26949	NC_000077.6	Vat1	3.80E+01
gene4981	1E+08	NC_000068.7	Gm10774	3.78E+01
gene1152	320982	NC_000067.6	Arl4c	3.74E+01
gene17315	22695	NC_000073.6	Zfp36	3.74E+01
gene11507	67695	NC_000071.6	Ost4	3.74E+01
gene18006	16828	NC_000073.6	Ldha	3.73E+01
gene30205	66483	NC_000078.6	Rpl36al	3.73E+01
gene1085	19231	NC_000067.6	Ptma	3.73E+01
gene20579	1E+08	NC_000074.6	Gm10131	3.72E+01
gene28258	20556	NC_000077.6	Slfn2	3.70E+01
gene40444	15181	NC_000083.6	Gm10093	3.69E+01
gene26791	ene=LOC1	NC_000077.6	LOC102633332	3.68E+01
gene16657	68953	NC_000073.6	Chmp2a	3.68E+01
gene41542	627792	NC_000084.6	Gm6789	3.67E+01
gene34861	668118	NC_000080.6	Gm8983	3.67E+01
gene32267	15508	NC_000079.6	Hsp25-ps1	3.67E+01
gene19384	434233	NC_000073.6	Gm5601	3.67E+01
gene39565	15000	NC_000083.6	H2-DMb2	3.66E+01
gene36448	66445	NC_000081.6	Cyc1	3.66E+01

gene5460	15901	NC_000068.7	Id1	3.65E+01
gene27084	12450	NC_000077.6	Ccng1	3.62E+01
gene42197	621699	NC_000085.6	Gm6252	3.62E+01
gene32494	66494	NC_000079.6	Prelid1	3.62E+01
gene7151	246703	NC_000069.6	Apoa1bp	3.61E+01
gene40953	666114	NC_000084.6	Gm7936	3.61E+01
gene5060	16176	NC_000068.7	Il1b	3.60E+01
gene14426	53379	NC_000072.6	Hnrnpa2b1	3.60E+01
gene24092	14678	NC_000075.6	Gnai2	3.58E+01
gene11944	545756	NC_000071.6	Gm5867	3.58E+01
gene27819	56310	NC_000077.6	Gps2	3.57E+01
gene7098	1E+08	NC_000069.6	Gm9790	3.57E+01
gene36749	12257	NC_000081.6	Tspo	3.57E+01
gene10893	ene;gene=l	NC_000070.6	LOC102638183	3.56E+01
gene26482	12512	NC_000076.6	Cd63	3.56E+01
gene25655	30055	NC_000076.6	Timm13	3.56E+01
gene23679	671392	NC_000075.6	Gm9531	3.56E+01
gene4715	66181	NC_000068.7	Nop10	3.55E+01
gene29578	1E+08	NC_000078.6	Gm35638	3.53E+01
gene39719	77810	NC_000083.6	A930015D03Rik	3.52E+01
gene39670	15013	NC_000083.6	H2-Q2	3.52E+01
gene32719	13039	NC_000079.6	Ctsl	3.51E+01
gene27195	1E+08	NC_000077.6	Tgtp2	3.50E+01
gene6124	26444	NC_000068.7	Psma7	3.49E+01
gene39296	19252	NC_000083.6	Dusp1	3.49E+01
gene5584	628707	NC_000068.7	Gm14253	3.48E+01
gene15793	640703	NC_000072.6	Gm7308	3.47E+01
gene11021	66077	NC_000070.6	Aurkaip1	3.47E+01
gene12930	623672	NC_000071.6	Gm6444	3.47E+01
gene5627	1E+08	NC_000068.7	Gm14176	3.47E+01
gene15083	1E+08	NC_000072.6	Gm9769	3.46E+01
gene34110	1E+08	NC_000080.6	Rpl23a-ps3	3.46E+01
gene26117	21664	NC_000076.6	Phlda1	3.45E+01
gene29349	11465	NC_000077.6	Actg1	3.45E+01
gene10460	1E+08	NC_000070.6	Gm12989	3.44E+01
gene39556	14977	NC_000083.6	Slc39a7	3.44E+01
gene32662	665455	NC_000079.6	Gm7640	3.43E+01
gene558	20846	NC_000067.6	Stat1	3.43E+01
gene15630	17113	NC_000072.6	M6pr	3.42E+01
gene18834	630138	NC_000073.6	Gm7027	3.42E+01
gene15791	111223	NC_000072.6	Tpi-rs11	3.42E+01
gene37390	13731	NC_000082.6	Emp2	3.41E+01
gene4638	623031	NC_000068.7	Gm13910	3.41E+01
gene14727	692165	NC_000072.6	Igkv10-96	3.40E+01
gene38832	20656	NC_000083.6	Sod2	3.40E+01
gene16761	20364	NC_000073.6	Sepw1	3.39E+01
gene4208	1E+08	NC_000068.7	Gm13736	3.38E+01
gene33187	667582	NC_000079.6	Gm8712	3.38E+01

gene5921	228916	NC_000068.7	Gm14269	3.37E+01
gene15635	667035	NC_000072.6	Gm8430	3.37E+01
gene29749	629364	NC_000078.6	Gm16372	3.35E+01
gene40027	66119	NC_000083.6	Tomm6	3.35E+01
gene33429	668181	NC_000079.6	Ncf2-rs	3.35E+01
gene7702	99543	NC_000069.6	Olfml3	3.35E+01
gene29637	1E+08	NC_000078.6	Bnip3l-ps	3.34E+01
gene19471	104277	NC_000073.6	Glud-ps	3.34E+01
gene29387	111241	NC_000077.6	Hmga1-rs1	3.33E+01
gene36694	69029	NC_000081.6	Smdt1	3.33E+01
gene26364	67125	NC_000076.6	Tspan31	3.33E+01
gene26006	67603	NC_000076.6	Dusp6	3.33E+01
gene23790	13036	NC_000075.6	Ctsh	3.32E+01
gene17862	1E+08	NC_000073.6	Gm15396	3.32E+01
gene37208	16668	NC_000081.6	Krt18	3.32E+01
gene35970	18458	NC_000081.6	Pabpc1	3.30E+01
gene9920	20116	NC_000070.6	Rps8	3.30E+01
gene3411	1E+08	NC_000068.7	Gm13450	3.29E+01
gene39739	1E+08	NC_000083.6	Gm4246	3.29E+01
gene28655	103742	NC_000077.6	Mien1	3.29E+01
gene34341	16854	NC_000080.6	Lgals3	3.29E+01
gene14011	94283	NC_000072.6	Ybx1-ps2	3.28E+01
gene33232	218501	NC_000079.6	Gm4815	3.28E+01
gene25001	1E+08	NC_000076.6	Gm3699	3.28E+01
gene5546	68728	NC_000068.7	Trp53inp2	3.27E+01
gene27950	66048	NC_000077.6	Emc6	3.27E+01
gene19349	54208	NC_000073.6	Arl6ip1	3.27E+01
gene24157	22040	NC_000075.6	Trex1	3.27E+01
gene7164	56700	NC_000069.6	Glmp	3.27E+01
gene23936	56492	NC_000075.6	Cldn18	3.26E+01
gene8741	1E+08	NC_000070.6	Gm12751	3.26E+01
gene30748	1E+08	NC_000078.6	Gm2614	3.26E+01
gene19514	56312	NC_000073.6	Nupr1	3.24E+01
gene19218	66085	NC_000073.6	Eif3f	3.24E+01
gene14733	692161	NC_000072.6	Igkv19-93	3.23E+01
gene38707	545184	NC_000083.6	Gm5812	3.23E+01
gene24795	15352	NC_000076.6	Gm4739	3.22E+01
gene6197	20672	NC_000068.7	Sox18	3.21E+01
gene13778	12390	NC_000072.6	Cav2	3.21E+01
gene13522	ɔgene;gbke	NC_000071.6	LOC100534385	3.21E+01
gene10529	13200	NC_000070.6	Ddost	3.21E+01
gene3175	27176	NC_000068.7	Rpl7a	3.20E+01
gene34735	17387	NC_000080.6	Mmp14	3.20E+01
gene14501	11826	NC_000072.6	Aqp1	3.19E+01
gene12741	56455	NC_000071.6	Dynll1	3.18E+01
gene10465	52830	NC_000070.6	Pnrc2	3.17E+01
gene1351	1E+08	NC_000067.6	Gm10193	3.16E+01
gene1821	1E+08	NC_000067.6	Gm15453	3.16E+01

gene40724	225134	NC_000084.6	Gm4833	3.16E+01
gene25124	668347	NC_000076.6	Gm9118	3.15E+01
gene1563	18703	NC_000067.6	Pigr	3.15E+01
gene14961	19695	NC_000072.6	Reg3g	3.14E+01
gene42671	15959	NC_000085.6	Ifit3	3.14E+01
gene3631	666876	NC_000068.7	Gm13503	3.14E+01
gene27377	15254	NC_000077.6	Hint1	3.13E+01
gene16802	12315	NC_000073.6	Calm3	3.13E+01
gene21571	546090	NC_000074.6	Gm5913	3.12E+01
gene18680	1E+08	NC_000073.6	Gm15501	3.12E+01
gene31688	1E+08	NC_000079.6	Mir1896	3.12E+01
gene42878	20250	NC_000085.6	Scd2	3.11E+01
gene32514	67511	NC_000079.6	Tmed9	3.11E+01
gene5635	21817	NC_000068.7	Tgm2	3.10E+01
gene3036	664822	NC_000068.7	Gm13331	3.10E+01
gene21921	68196	NC_000074.6	Hsbp1	3.10E+01
gene29177	50773	NC_000077.6	Nt5c	3.09E+01
gene42994	433251	NC_000085.6	Rpl13a-ps1	3.09E+01
gene29012	13207	NC_000077.6	Ddx5	3.09E+01
gene11172	654361	NC_000071.6	Gm7332	3.09E+01
gene7005	1E+08	NC_000069.6	Gm9762	3.09E+01
gene19558	20768	NC_000073.6	Sephs2	3.09E+01
gene11353	1E+08	NC_000071.6	Gm3724	3.08E+01
gene39195	27361	NC_000083.6	Msrb1	3.07E+01
gene7993	67704	NC_000069.6	1810037I17Rik	3.07E+01
gene8812	1E+08	NC_000070.6	Gm12396	3.06E+01
gene13260	26893	NC_000071.6	Cops6	3.06E+01
gene16918	57278	NC_000073.6	Bcam	3.06E+01
gene39735	675325	NC_000083.6	2410017I17Rik	3.05E+01
gene23088	12955	NC_000075.6	Cryab	3.05E+01
gene38267	106143	NC_000082.6	Cggbp1	3.04E+01
gene30803	76933	NC_000078.6	Ifi27l2a	3.04E+01
gene22939	15270	NC_000075.6	H2afx	3.04E+01
gene19097	24075	NC_000073.6	Taf10	3.03E+01
gene24882	22110	NC_000076.6	Tspyl1	3.02E+01
gene14345	107526	NC_000072.6	Gimap4	3.02E+01
gene43113	1E+08	NC_000085.6	Rps12-ps3	3.02E+01
gene10205	14612	NC_000070.6	Gja4	3.01E+01
gene27872	67863	NC_000077.6	Slc25a11	3.00E+01
gene8223	229900	NC_000069.6	Gbp7	3.00E+01
gene24400	12387	NC_000075.6	Ctnnb1	2.99E+01
gene27191	21822	NC_000077.6	Tgtp1	2.99E+01
gene17360	57296	NC_000073.6	Psmd8	2.99E+01
gene24386	67115	NC_000075.6	Rpl14	2.99E+01
gene39590	106512	NC_000083.6	Gpsm3	2.98E+01
gene14456	666487	NC_000072.6	Gm8129	2.97E+01
gene25005	12484	NC_000076.6	Cd24a	2.97E+01
gene21363	1E+08	NC_000074.6	Atp5k-ps1	2.96E+01

gene21974	15227	NC_000074.6	Foxf1	2.96E+01
gene19539	52858	NC_000073.6	Cdipt	2.96E+01
gene21112	67922	NC_000074.6	Fam32a	2.95E+01
gene17468	18301	NC_000073.6	Fxyd5	2.95E+01
gene14739	692154	NC_000072.6	Igkv1-88	2.93E+01
gene32074	20723	NC_000079.6	Serpinb9	2.93E+01
gene26106	432502	NC_000076.6	Rpl6l	2.92E+01
gene28757	16669	NC_000077.6	Krt19	2.92E+01
gene24896	17118	NC_000076.6	Marcks	2.91E+01
gene37862	1E+08	NC_000082.6	Gm20056	2.91E+01
gene19084	109042	NC_000073.6	Prkcdbp	2.90E+01
gene18536	80889	NC_000073.6	Mesdc1	2.89E+01
gene21336	68544	NC_000074.6	2310036O22Rik	2.88E+01
gene13564	433968	NC_000071.6	Gm5566	2.88E+01
gene28572	18023	NC_000077.6	Nfe2l1	2.88E+01
gene40707	665047	NC_000084.6	Gm7464	2.87E+01
gene7277	20193	NC_000069.6	S100a1	2.87E+01
gene7265	30791	NC_000069.6	Slc39a1	2.86E+01
gene39205	79044	NC_000083.6	Mrps34	2.86E+01
gene26452	17904	NC_000076.6	Myl6	2.86E+01
gene36397	68453	NC_000081.6	Gpihbp1	2.86E+01
gene41984	76308	NC_000085.6	Rab1b	2.85E+01
gene28291	1E+08	NC_000077.6	Wfdc17	2.84E+01
gene8222	229898	NC_000069.6	Gbp5	2.84E+01
gene39260	56424	NC_000083.6	Stub1	2.84E+01
gene15698	12034	NC_000072.6	Phb2	2.83E+01
gene17894	20130	NC_000073.6	Rras	2.83E+01
gene30414	12192	NC_000078.6	Zfp36l1	2.82E+01
gene24967	53599	NC_000076.6	Cd164	2.82E+01
gene18483	20068	NC_000073.6	Rps17	2.81E+01
gene10273	622469	NC_000070.6	Gm12966	2.80E+01
gene30804	52668	NC_000078.6	Ifi27	2.80E+01
gene13888	66144	NC_000072.6	Atp6v1f	2.80E+01
gene42898	94067	NC_000085.6	Mrpl43	2.80E+01
gene21056	67023	NC_000074.6	Use1	2.80E+01
gene14234	19667	NC_000072.6	Rbpsuh-rs3	2.79E+01
gene32914	16371	NC_000079.6	Irx1	2.79E+01
gene39121	353502	NC_000083.6	Hcfc1r1	2.79E+01
gene22024	11821	NC_000074.6	Aprt	2.79E+01
gene25689	432479	NC_000076.6	4930404N11Rik	2.79E+01
gene5563	613262	NC_000068.7	BC029722	2.78E+01
gene39406	18712	NC_000083.6	Pim1	2.78E+01
gene36469	66914	NC_000081.6	Vps28	2.77E+01
gene20025	12443	NC_000073.6	Ccnd1	2.77E+01
gene2744	623483	NC_000068.7	Gm13196	2.77E+01
gene28983	11421	NC_000077.6	Ace	2.77E+01
gene39170	67078	NC_000083.6	Pgp	2.77E+01
gene28549	1E+08	NC_000077.6	Gm11539	2.75E+01

gene17760	668668	NC_000073.6	Gm9294	2.75E+01
gene15010	68499	NC_000072.6	Mrpl53	2.75E+01
gene39563	14312	NC_000083.6	Brd2	2.75E+01
gene24850	432448	NC_000076.6	Gm5422	2.75E+01
gene29385	67880	NC_000077.6	Dcxr	2.75E+01
gene7289	20202	NC_000069.6	S100a9	2.75E+01
gene6395	665094	NC_000069.6	Gm7488	2.75E+01
gene12151	65961	NC_000071.6	Utp3	2.74E+01
gene9312	622491	NC_000070.6	Gm11221	2.74E+01
gene4403	19182	NC_000068.7	Psmc3	2.74E+01
gene30934	22375	NC_000078.6	Wars	2.74E+01
gene11560	545743	NC_000071.6	Gm5864	2.73E+01
gene39259	72106	NC_000083.6	Jmjd8	2.72E+01
gene27484	108660	NC_000077.6	Rnf187	2.72E+01
gene11838	666465	NC_000071.6	Gm8121	2.72E+01
gene4843	625328	NC_000068.7	H3f3c	2.71E+01
gene15538	20315	NC_000072.6	Cxcl12	2.70E+01
gene6891	67437	NC_000069.6	Ssr3	2.70E+01
gene12310	433923	NC_000071.6	Gm5560	2.70E+01
gene1700	13007	NC_000067.6	Csrp1	2.69E+01
gene21140	1E+08	NC_000074.6	Gm38435	2.69E+01
gene28177	104522	NC_000077.6	AU040972	2.69E+01
gene36450	68877	NC_000081.6	Maf1	2.69E+01
gene37220	106073	NC_000081.6	Mfsd5	2.68E+01
gene27087	432551	NC_000077.6	Hspd1-ps3	2.67E+01
gene5106	1E+08	NC_000068.7	Gm14048	2.67E+01
gene42667	12642	NC_000085.6	Ch25h	2.66E+01
gene24632	17684	NC_000076.6	Cited2	2.66E+01
gene27915	52700	NC_000077.6	Txndc17	2.65E+01
gene21261	81489	NC_000074.6	Dnajb1	2.65E+01
gene27774	78304	NC_000077.6	Naa38	2.64E+01
gene24481	12774	NC_000075.6	Ccr5	2.64E+01
gene37843	106264	NC_000082.6	0610012G03Rik	2.63E+01
gene42176	225896	NC_000085.6	Ubxn1	2.63E+01
gene736	667644	NC_000067.6	Gm11605	2.62E+01
gene41908	54445	NC_000085.6	Unc93b1	2.61E+01
gene24373	16785	NC_000075.6	Rpsa	2.61E+01
gene42447	54391	NC_000085.6	Rfk	2.60E+01
gene25519	1E+08	NC_000076.6	Gm10146	2.60E+01
gene13433	11867	NC_000071.6	Arpc1b	2.60E+01
gene19739	668750	NC_000073.6	Gm9333	2.59E+01
gene11122	14362	NC_000071.6	Fzd1	2.59E+01
gene39193	68342	NC_000083.6	Ndufb10	2.58E+01
gene1652	12227	NC_000067.6	Btg2	2.58E+01
gene10648	27984	NC_000070.6	Efhd2	2.58E+01
gene21011	14232	NC_000074.6	Fkbp8	2.58E+01
gene28434	67888	NC_000077.6	Tmem100	2.58E+01
gene8419	624684	NC_000069.6	Gm6520	2.58E+01

gene37570	13358	NC_000082.6	Slc25a1	2.57E+01
gene27135	1E+08	NC_000077.6	Gm12166	2.57E+01
gene24352	17874	NC_000075.6	Myd88	2.57E+01
gene5373	545472	NC_000068.7	Gm14121	2.56E+01
gene34427	667210	NC_000080.6	Gm8518	2.55E+01
gene9331	353204	NC_000070.6	Aldoart1	2.55E+01
gene2094	433375	NC_000067.6	Creg1	2.54E+01
gene48455	17747	NC_005089.1	TrnY	2.54E+01
gene24156	66940	NC_000075.6	Shisa5	2.54E+01
gene17400	12336	NC_000073.6	Capns1	2.54E+01
gene7283	20198	NC_000069.6	S100a4	2.54E+01
gene26363	12567	NC_000076.6	Cdk4	2.54E+01
gene5336	17064	NC_000068.7	Cd93	2.53E+01
gene26372	14421	NC_000076.6	B4galnt1	2.52E+01
gene31434	236294	NC_000079.6	Gm4900	2.52E+01
gene6741	545524	NC_000069.6	Gm10254	2.52E+01
gene35448	239217	NC_000080.6	Kctd12	2.52E+01
gene22922	21838	NC_000075.6	Thy1	2.52E+01
gene13106	17969	NC_000071.6	Ncf1	2.51E+01
gene41995	23825	NC_000085.6	Banf1	2.51E+01
gene9464	269553	NC_000070.6	Mrpl48-ps	2.51E+01
gene20493	1E+08	NC_000074.6	Gm29728	2.51E+01
gene18182	664849	NC_000073.6	Gm7367	2.50E+01
gene19915	66853	NC_000073.6	Pnpla2	2.50E+01
gene24716	432438	NC_000076.6	Gm5421	2.50E+01
gene25385	623346	NC_000076.6	Gm6419	2.50E+01
gene37014	67739	NC_000081.6	Slc48a1	2.50E+01
gene21582	1E+08	NC_000074.6	Gm15210	2.49E+01
gene28075	1E+08	NC_000077.6	Rpl36-ps2	2.49E+01
gene21439	1E+08	NC_000074.6	Gm19935	2.49E+01
gene29524	633030	NC_000078.6	Gm7099	2.49E+01
gene36382	1E+08	NC_000081.6	Ly6c2	2.49E+01
gene39056	ene=LOC1	NC_000083.6	LOC102633973	2.48E+01
gene19087	20597	NC_000073.6	Smpd1	2.47E+01
gene1346	1E+08	NC_000067.6	Gm3608	2.47E+01
gene35454	16365	NC_000080.6	Acod1	2.45E+01
gene32931	94066	NC_000079.6	Mrpl36	2.45E+01
gene27330	22272	NC_000077.6	Uqcrq	2.45E+01
gene27384	14667	NC_000077.6	Gm2a	2.44E+01
gene24469	654467	NC_000075.6	Gm10052	2.44E+01
gene4791	20813	NC_000068.7	Srp14	2.44E+01
gene41001	433170	NC_000084.6	Gm5503	2.44E+01
gene14176	22793	NC_000072.6	Zyx	2.44E+01
gene9360	66928	NC_000070.6	Tmem261	2.44E+01
gene16608	1E+08	NC_000073.6	Gm10179	2.44E+01
gene17861	16541	NC_000073.6	Napsa	2.44E+01
gene39066	671007	NC_000083.6	Gm9514	2.43E+01
gene19879	68713	NC_000073.6	Ifitm1	2.43E+01

gene41092	17991	NC_000084.6	Ndufa2	2.43E+01
gene7725	11853	NC_000069.6	Rhoc	2.43E+01
gene36322	1E+08	NC_000081.6	Gm3150	2.43E+01
gene36600	223696	NC_000081.6	Tomm22	2.43E+01
gene18116	497028	NC_000073.6	Gm5776	2.42E+01
gene19358	319622	NC_000073.6	Itpripl2	2.42E+01
gene7458	76893	NC_000069.6	Cers2	2.42E+01
gene32655	1E+08	NC_000079.6	Gm20563	2.42E+01
gene3082	68475	NC_000068.7	Ssna1	2.42E+01
gene5018	67885	NC_000068.7	1500011K16Rik	2.41E+01
gene7372	20195	NC_000069.6	S100a11	2.40E+01
gene14999	13448	NC_000072.6	Dok1	2.40E+01
gene17977	13732	NC_000073.6	Emp3	2.40E+01
gene21065	94065	NC_000074.6	Mrpl34	2.40E+01
gene881	227290	NC_000067.6	Aamp	2.39E+01
gene10303	16792	NC_000070.6	Laptm5	2.39E+01
gene39564	14998	NC_000083.6	H2-DMa	2.39E+01
gene41928	19265	NC_000085.6	Ptprcap	2.38E+01
gene33057	432789	NC_000079.6	Gm5452	2.38E+01
gene6508	1E+08	NC_000069.6	Gm9791	2.38E+01
gene41688	628298	NC_000084.6	Gm14328	2.38E+01
gene3769	1E+08	NC_000068.7	Gm13597	2.38E+01
gene19098	12751	NC_000073.6	Tpp1	2.37E+01
gene15866	=Gene;ger	NC_000072.6	LOC108167382	2.37E+01
gene25470	16414	NC_000076.6	Itgb2	2.37E+01
gene996	433328	NC_000067.6	Gm5530	2.37E+01
gene36375	1E+08	NC_000081.6	Gm28068	2.35E+01
gene30105	21869	NC_000078.6	Nkx2-1	2.35E+01
gene17860	22260	NC_000073.6	Nr1h2	2.35E+01
gene21457	16373	NC_000074.6	Irx3	2.35E+01
gene16589	628898	NC_000073.6	Gm6929	2.35E+01
gene26739	16342	NC_000077.6	Eif3s6-ps1	2.35E+01
gene1058	64294	NC_000067.6	Itm2c	2.35E+01
gene11115	667617	NC_000071.6	Rps4x-ps	2.34E+01
gene23524	28015	NC_000075.6	Polr2m	2.34E+01
gene21013	71780	NC_000074.6	Isyna1	2.33E+01
gene3178	20932	NC_000068.7	Surf4	2.33E+01
gene27499	11840	NC_000077.6	Arf1	2.33E+01
gene29619	71853	NC_000078.6	Pdia6	2.32E+01
gene11532	=Gene;ger	NC_000071.6	LOC108169038	2.32E+01
gene31037	15519	NC_000078.6	Hsp90aa1	2.32E+01
gene26568	1E+08	NC_000076.6	Gm9770	2.32E+01
gene31064	217869	NC_000078.6	Eif5	2.31E+01
gene26636	66152	NC_000077.6	Uqcr10	2.31E+01
gene982	1E+08	NC_000067.6	Gm28071	2.31E+01
gene1893	14645	NC_000067.6	Glul	2.31E+01
gene42466	16952	NC_000085.6	Anxa1	2.31E+01
gene21108	326618	NC_000074.6	Tpm4	2.31E+01

gene35066	12759	NC_000080.6	Clu	2.30E+01
gene1033	1E+08	NC_000067.6	Rpl19-ps1	2.30E+01
gene11707	665839	NC_000071.6	Gm7816	2.30E+01
gene22436	66177	NC_000075.6	Ubl5	2.29E+01
gene12225	56066	NC_000071.6	Cxcl11	2.29E+01
gene26780	111116	NC_000077.6	Akt2-ps	2.29E+01
gene19763	546009	NC_000073.6	Gm5904	2.28E+01
gene42177	107197	NC_000085.6	Uqcc3	2.28E+01
gene26291	75612	NC_000076.6	Gns	2.28E+01
gene32916	1E+08	NC_000079.6	Rpl9-ps4	2.28E+01
gene13983	78412	NC_000072.6	3110062M04Rik	2.28E+01
gene8896	18829	NC_000070.6	Ccl21a	2.27E+01
gene17448	69804	NC_000073.6	Tmem147	2.27E+01
gene7474	56205	NC_000069.6	Ensa	2.27E+01
gene17963	19899	NC_000073.6	Rpl18	2.27E+01
gene9313	433716	NC_000070.6	Gm11222	2.27E+01
gene12734	109154	NC_000071.6	Mlec	2.27E+01
gene34260	13587	NC_000080.6	Ear2	2.27E+01
gene36660	1E+08	NC_000081.6	Gm18284	2.26E+01
gene20226	665536	NC_000074.6	Gm7676	2.26E+01
gene14793	619846	NC_000072.6	Igkv5-48	2.26E+01
gene15691	50909	NC_000072.6	C1ra	2.25E+01
gene35835	64833	NC_000081.6	Acot10	2.25E+01
gene23224	28014	NC_000075.6	D9Wsu149	2.25E+01
gene38255	1E+08	NC_000082.6	Gm9816	2.25E+01
gene23079	30057	NC_000075.6	Timm8b	2.25E+01
gene38825	21454	NC_000083.6	Tcp1	2.25E+01
gene40510	433141	NC_000083.6	Rpsa-ps7	2.24E+01
gene22036	1E+08	NC_000074.6	Gm16378	2.24E+01
gene10868	627985	NC_000070.6	Trmt112-ps2	2.23E+01
gene14829	232065	NC_000072.6	Igkv8-19	2.23E+01
gene33418	ene=LOC1	NC_000079.6	LOC101055980	2.22E+01
gene11777	433885	NC_000071.6	Gm5555	2.22E+01
gene21088	1E+08	NC_000074.6	Gm18860	2.22E+01
gene36242	22089	NC_000081.6	Tsg101-ps	2.22E+01
gene22948	75617	NC_000075.6	Rps25	2.21E+01
gene25657	17873	NC_000076.6	Gadd45b	2.21E+01
gene27314	213673	NC_000077.6	9530068E07Rik	2.21E+01
gene38945	14289	NC_000083.6	Fpr2	2.21E+01
gene40089	654432	NC_000083.6	Gm7334	2.20E+01
gene29392	14104	NC_000077.6	Fasn	2.20E+01
gene11836	625721	NC_000071.6	Gm6615	2.20E+01
gene7116	1E+08	NC_000069.6	Gm10705	2.20E+01
gene14908	66510	NC_000072.6	Rnf181	2.19E+01
gene14332	666005	NC_000072.6	Gm7887	2.19E+01
gene3070	227612	NC_000068.7	Tor4a	2.19E+01
gene16048	11857	NC_000072.6	Arhgdib	2.18E+01
gene12699	330171	NC_000071.6	Kctd10	2.18E+01

gene40190	54217	NC_000083.6	Rpl36	2.17E+01
gene19052	545987	NC_000073.6	Gm5900	2.17E+01
gene28982	1E+08	NC_000077.6	Gm11651	2.17E+01
gene30836	238393	NC_000078.6	Serpina3f	2.16E+01
gene13045	19384	NC_000071.6	Ran	2.16E+01
gene19606	66824	NC_000073.6	Pycard	2.16E+01
gene10007	22608	NC_000070.6	Ybx1	2.15E+01
gene40356	67938	NC_000083.6	Myl12b	2.15E+01
gene39313	67675	NC_000083.6	Cuta	2.15E+01
gene34026	16427	NC_000080.6	Itih4	2.15E+01
gene21694	59016	NC_000074.6	Thap11	2.15E+01
gene2255	18611	NC_000067.6	Pea15a	2.15E+01
gene25616	66374	NC_000076.6	2310011J03Rik	2.15E+01
gene27177	1E+08	NC_000077.6	Gm12184	2.14E+01
gene22651	67398	NC_000075.6	Srpr	2.14E+01
gene26817	14646	NC_000077.6	Glns-ps1	2.14E+01
gene42452	235503	NC_000085.6	Eef1a1-ps1	2.14E+01
gene42434	624438	NC_000085.6	Gm10819	2.13E+01
gene38920	626785	NC_000083.6	Gm6705	2.13E+01
gene42938	18034	NC_000085.6	Nfkb2	2.12E+01
gene8926	12913	NC_000070.6	Creb3	2.11E+01
gene5981	58203	NC_000068.7	Zbp1	2.11E+01
gene20076	631071	NC_000074.6	Gm16589	2.11E+01
gene42122	665419	NC_000085.6	Rab11b-ps2	2.10E+01
gene5614	ene;gene=L	NC_000068.7	LOC105244345	2.10E+01
gene546	20324	NC_000067.6	Sdpr	2.10E+01
gene12427	20750	NC_000071.6	Spp1	2.09E+01
gene37174	110310	NC_000081.6	Krt7	2.09E+01
gene42254	624395	NC_000085.6	Gm6501	2.09E+01
gene8481	53378	NC_000070.6	Sdcbp	2.09E+01
gene15407	20893	NC_000072.6	Bhlhe40	2.09E+01
gene40188	224904	NC_000083.6	2410015M20Rik	2.09E+01
gene36734	109754	NC_000081.6	Cyb5r3	2.08E+01
gene34498	193533	NC_000080.6	Gm4742	2.08E+01
gene15743	213233	NC_000072.6	Tapbpl	2.08E+01
gene14810	434039	NC_000072.6	Igkv6-32	2.08E+01
gene26421	1E+08	NC_000076.6	Gm10120	2.07E+01
gene26675	13169	NC_000077.6	Dbnl	2.07E+01
gene14798	381783	NC_000072.6	Igkv5-43	2.07E+01
gene22205	ene;gene=L	NC_000074.6	LOC108167576	2.07E+01
gene882	=Gene;ger	NC_000067.6	LOC108167628	2.07E+01
gene42003	107242	NC_000085.6	AI837181	2.06E+01
gene5916	20461	NC_000068.7	Gm14270	2.05E+01
gene7452	57912	NC_000069.6	Cdc42se1	2.05E+01
gene24615	666904	NC_000076.6	Gm8355	2.05E+01
gene41061	72512	NC_000084.6	Tmem173	2.05E+01
gene7665	11928	NC_000069.6	Atp1a1	2.05E+01
gene39678	15018	NC_000083.6	H2-Q7	2.05E+01

gene39206	79059	NC_000083.6	Nme3	2.05E+01
gene18978	1E+08	NC_000073.6	Dnajc19-ps	2.05E+01
gene33812	238963	NC_000080.6	Gm4939	2.05E+01
gene23287	18746	NC_000075.6	Pkm	2.04E+01
gene30571	238330	NC_000078.6	Irf2bpl	2.04E+01
gene42826	277010	NC_000085.6	Marveld1	2.04E+01
gene1542	12767	NC_000067.6	Cxcr4	2.03E+01
gene2383	51810	NC_000067.6	Hnrnpu	2.03E+01
gene37123	1E+08	NC_000081.6	Uqcrh-ps1	2.03E+01
gene22862	15481	NC_000075.6	Hspa8	2.03E+01
gene11022	74761	NC_000070.6	Mxra8	2.03E+01
gene18392	ene;gene=L	NC_000073.6	LOC108167440	2.02E+01
gene6000	664901	NC_000068.7	Vamp7-ps	2.02E+01
gene16060	56615	NC_000072.6	Mgst1	2.02E+01
gene16658	22192	NC_000073.6	Ube2m	2.02E+01
gene5664	16658	NC_000068.7	Mafb	2.01E+01
gene22447	53356	NC_000075.6	Eif3g	2.01E+01
gene37133	70152	NC_000081.6	Mettl7a1	2.01E+01
gene34797	16391	NC_000080.6	Irf9	2.01E+01
gene21069	84094	NC_000074.6	Plvap	2.01E+01
gene41349	240289	NC_000084.6	Gm4950	2.01E+01
gene28879	68066	NC_000077.6	Slc25a39	2.00E+01
gene19543	17188	NC_000073.6	Maz	2.00E+01
gene13279	14720	NC_000071.6	Got2-ps1	2.00E+01
gene32106	22151	NC_000079.6	Tubb2a	2.00E+01
gene14803	620017	NC_000072.6	Igkv5-39	2.00E+01
gene14797	545851	NC_000072.6	Igkv12-44	1.99E+01
gene13709	1E+08	NC_000072.6	Gm9825	1.98E+01
gene10075	12331	NC_000070.6	Cap1	1.98E+01
gene36868	140570	NC_000081.6	Plxnb2	1.98E+01
gene8283	16007	NC_000069.6	Cyr61	1.98E+01
gene6548	11747	NC_000069.6	Anxa5	1.97E+01
gene9878	66576	NC_000070.6	Uqcrh	1.97E+01
gene5401	1E+08	NC_000068.7	Gm14150	1.97E+01
gene37729	13682	NC_000082.6	Eif4a2	1.97E+01
gene21146	15368	NC_000074.6	Hmox1	1.97E+01
gene15012	22377	NC_000072.6	Wbp1	1.96E+01
gene39181	65962	NC_000083.6	Slc9a3r2	1.96E+01
gene36468	72027	NC_000081.6	Slc39a4	1.96E+01
gene30349	238276	NC_000078.6	Akap5	1.96E+01
gene31115	104759	NC_000078.6	Pld4	1.96E+01
gene17269	18807	NC_000073.6	Pld3	1.96E+01
gene30192	1E+08	NC_000078.6	Rpl17-ps3	1.95E+01
gene13194	26433	NC_000071.6	Plod3	1.95E+01
gene23579	1E+08	NC_000075.6	Gm2981	1.95E+01
gene20058	69189	NC_000074.6	Mcemp1	1.95E+01
gene42165	66836	NC_000085.6	Tmem223	1.95E+01
gene27824	53624	NC_000077.6	Cldn7	1.94E+01

gene37160	11482	NC_000081.6	Acvrl1	1.94E+01
gene3403	227746	NC_000068.7	Rabepk	1.94E+01
gene12186	14825	NC_000071.6	Cxcl1	1.94E+01
gene25003	1E+08	NC_000076.6	Gm9803	1.94E+01
gene18482	636544	NC_000073.6	Gm7180	1.93E+01
gene18537	69579	NC_000073.6	2310034P14Rik	1.93E+01
gene39823	14990	NC_000083.6	H2-M2	1.92E+01
gene1074	17975	NC_000067.6	Ncl	1.92E+01
gene34146	66092	NC_000080.6	Ghitm	1.91E+01
gene35176	213469	NC_000080.6	Lgi3	1.91E+01
gene28402	110809	NC_000077.6	Srsf1	1.90E+01
gene12968	65105	NC_000071.6	Arl6ip4	1.90E+01
gene9261	59001	NC_000070.6	Pole3	1.90E+01
gene24474	80901	NC_000075.6	Cxcr6	1.90E+01
gene22355	434373	NC_000075.6	Gm5611	1.90E+01
gene33865	66121	NC_000080.6	Chchd1	1.90E+01
gene36301	665945	NC_000081.6	Gm7859	1.89E+01
gene26703	19291	NC_000077.6	Purb	1.89E+01
gene41452	225594	NC_000084.6	Gm4841	1.89E+01
gene39254	214917	NC_000083.6	Fam173a	1.89E+01
gene15021	232157	NC_000072.6	Mob1a	1.88E+01
gene25601	66043	NC_000076.6	Atp5d	1.88E+01
gene40832	1E+08	NC_000084.6	Gm10036	1.88E+01
gene19891	107702	NC_000073.6	Rnh1	1.88E+01
gene21345	17159	NC_000074.6	Man2b1	1.88E+01
gene12571	57914	NC_000071.6	Crlf2	1.88E+01
gene5612	56045	NC_000068.7	Samhd1	1.88E+01
gene16253	50918	NC_000073.6	Myadm	1.87E+01
gene4897	378702	NC_000068.7	Serf2	1.87E+01
gene3995	103324	NC_000068.7	Gm4735	1.87E+01
gene3109	59022	NC_000068.7	Edf1	1.86E+01
gene21499	17748	NC_000074.6	Mt1	1.86E+01
gene887	227292	NC_000067.6	Ctdsp1	1.86E+01
gene16264	66091	NC_000073.6	Ndufa3	1.86E+01
gene4627	383739	NC_000068.7	Gm13904	1.86E+01
gene42051	29805	NC_000085.6	Znhit2	1.86E+01
gene29071	19084	NC_000077.6	Prkar1a	1.85E+01
gene36316	1E+08	NC_000081.6	Gm2999	1.85E+01
gene14826	620400	NC_000072.6	Igkv8-21	1.85E+01
gene4424	ene=LOC1	NC_000068.7	LOC102633523	1.85E+01
gene15871	57436	NC_000072.6	Gabarapl1	1.85E+01
gene39917	73504	NC_000083.6	1700071M16Rik	1.85E+01
gene42983	67788	NC_000085.6	Sfr1	1.85E+01
gene14346	231931	NC_000072.6	Gimap6	1.84E+01
gene17177	77124	NC_000073.6	9130221H12Rik	1.84E+01
gene33380	75616	NC_000079.6	Smim15	1.84E+01
gene30318	1E+08	NC_000078.6	Gm15283	1.84E+01
gene37803	15205	NC_000082.6	Hes1	1.84E+01

gene14823	637227	NC_000072.6	Igkv6-23	1.84E+01
gene38389	1E+08	NC_000082.6	Gm10791	1.84E+01
gene42588	60533	NC_000085.6	Cd274	1.84E+01
gene39562	15001	NC_000083.6	H2-Oa	1.83E+01
gene38948	665187	NC_000083.6	Gm7535	1.83E+01
gene17695	ene;gene=L	NC_000073.6	LOC105247253	1.83E+01
gene25072	14728	NC_000076.6	Lilrb4a	1.83E+01
gene27810	70310	NC_000077.6	Plscr3	1.82E+01
gene27023	666646	NC_000077.6	Gm12115	1.82E+01
gene5076	20638	NC_000068.7	Snrpb	1.82E+01
gene37340	66911	NC_000082.6	Nudt16l1	1.82E+01
gene12731	1E+08	NC_000071.6	Rpl37rt	1.81E+01
gene34825	545056	NC_000080.6	Gm5801	1.81E+01
gene27860	574428	NC_000077.6	Zmynd15	1.81E+01
gene7139	15191	NC_000069.6	Hdgf	1.81E+01
gene14731	667550	NC_000072.6	Igkv10-94	1.81E+01
gene42100	14227	NC_000085.6	Fkbp2	1.81E+01
gene6149	72075	NC_000068.7	Ogfr	1.81E+01
gene39129	638247	NC_000083.6	9530082P21Rik	1.80E+01
gene33201	620648	NC_000079.6	Gm6169	1.79E+01
gene1628	108954	NC_000067.6	Ppp1r15b	1.79E+01
gene10470	26394	NC_000070.6	Lypla2	1.79E+01
gene25214	20224	NC_000076.6	Sar1a	1.79E+01
gene41715	1E+08	NC_000084.6	Gm20570	1.78E+01
gene41300	66676	NC_000084.6	Tmed7	1.78E+01
gene42062	433216	NC_000085.6	Gm5510	1.77E+01
gene2183	14131	NC_000067.6	Fcgr3	1.77E+01
gene21039	76808	NC_000074.6	Rpl18a	1.77E+01
gene28799	19285	NC_000077.6	Ptrf	1.77E+01
gene40865	665674	NC_000084.6	Tpi-rs10	1.76E+01
gene10462	665334	NC_000070.6	Gm12988	1.76E+01
gene26692	103694	NC_000077.6	Tmed4	1.76E+01
gene39625	15511	NC_000083.6	Hspa1b	1.76E+01
gene9899	18477	NC_000070.6	Prdx1	1.75E+01
gene39973	28064	NC_000083.6	Yipf3	1.75E+01
gene11143	1E+08	NC_000071.6	Tubb4b-ps1	1.75E+01
gene17725	628372	NC_000073.6	Gm6872	1.75E+01
gene11472	ene;gene=L	NC_000071.6	LOC102640024	1.75E+01
gene20325	1E+08	NC_000074.6	Gm15056	1.74E+01
gene41704	407819	NC_000084.6	BC031181	1.74E+01
gene10831	11610	NC_000070.6	Agtrap	1.74E+01
gene9473	242521	NC_000070.6	Klhl9	1.74E+01
gene11526	266781	NC_000071.6	Snx17	1.73E+01
gene7477	13601	NC_000069.6	Ecm1	1.73E+01
gene25577	19205	NC_000076.6	Ptbp1	1.73E+01
gene5775	18830	NC_000068.7	Pltp	1.73E+01
gene916	227298	NC_000067.6	Fam134a	1.73E+01
gene14786	385253	NC_000072.6	Igkv4-55	1.73E+01

gene17411	68332	NC_000073.6	Sdhaf1	1.73E+01
gene13119	22384	NC_000071.6	Eif4h	1.73E+01
gene39778	76497	NC_000083.6	Ppp1r11	1.73E+01
gene32046	66694	NC_000079.6	Uqcrfs1	1.73E+01
gene5093	99045	NC_000068.7	Mrps26	1.72E+01
gene26209	215449	NC_000076.6	Rap1b	1.72E+01
gene2009	1E+08	NC_000067.6	Gm15429	1.72E+01
gene7219	66059	NC_000069.6	Krtcap2	1.72E+01
gene14879	12525	NC_000072.6	Cd8a	1.72E+01
gene6663	545519	NC_000069.6	Gm5846	1.72E+01
gene21964	15900	NC_000074.6	Irf8	1.72E+01
gene7431	20341	NC_000069.6	Selenbp1	1.72E+01
gene3099	227622	NC_000068.7	BC029214	1.71E+01
gene9138	16600	NC_000070.6	Klf4	1.71E+01
gene38490	1E+08	NC_000082.6	Gm2805	1.71E+01
gene610	666793	NC_000067.6	Gm8292	1.71E+01
gene17262	668045	NC_000073.6	Pgam1-ps2	1.70E+01
gene23662	69981	NC_000075.6	Tmem30a	1.70E+01
gene28916	192231	NC_000077.6	Hexim1	1.70E+01
gene28264	1E+08	NC_000077.6	AI662270	1.70E+01
gene2275	74748	NC_000067.6	Slamf8	1.70E+01
gene4035	56791	NC_000068.7	Ube2l6	1.70E+01
gene25595	66420	NC_000076.6	Polr2e	1.70E+01
gene39652	11629	NC_000083.6	Aif1	1.70E+01
gene21264	19216	NC_000074.6	Ptger1	1.70E+01
gene23451	67941	NC_000075.6	Rps27l	1.69E+01
gene3138	109299	NC_000068.7	C330006A16Rik	1.69E+01
gene28532	14710	NC_000077.6	Gngt2	1.69E+01
gene7275	20615	NC_000069.6	Snapin	1.69E+01
gene8818	12017	NC_000070.6	Bag1	1.69E+01
gene38136	1E+08	NC_000082.6	Mir6363	1.69E+01
gene39944	18037	NC_000083.6	Nfkbie	1.68E+01
gene22695	gene=LOC1	NC_000075.6	LOC108167643	1.68E+01
gene39602	12268	NC_000083.6	C4b	1.68E+01
gene38494	20655	NC_000082.6	Sod1	1.68E+01
gene16884	22323	NC_000073.6	Vasp	1.68E+01
gene38655	1E+08	NC_000083.6	Gm10232	1.68E+01
gene42031	72289	NC_000085.6	Malat1	1.68E+01
gene42001	ene=LOC1	NC_000085.6	LOC102633050	1.67E+01
gene10823	77034	NC_000070.6	2510039O18Rik	1.67E+01
gene22519	11433	NC_000075.6	Acp5	1.66E+01
gene16206	66184	NC_000072.6	Rps4l	1.66E+01
gene21202	107730	NC_000074.6	Tpd52-ps	1.66E+01
gene2470	13244	NC_000067.6	Degs1	1.66E+01
gene41147	ene=LOC1	NC_000084.6	LOC101055737	1.66E+01
gene7228	11490	NC_000069.6	Adam15	1.66E+01
gene17854	69683	NC_000073.6	Emc10	1.65E+01
gene6375	gene=LOC1	NC_000069.6	LOC102633274	1.65E+01

gene7221	19729	NC_000069.6	Slc50a1	1.65E+01
gene26455	23943	NC_000076.6	Esyt1	1.65E+01
gene9518	ene=LOC1	NC_000070.6	LOC108169000	1.65E+01
gene38386	11957	NC_000082.6	Atp5j	1.64E+01
gene35905	12465	NC_000081.6	Cct5	1.64E+01
gene21622	68523	NC_000074.6	Fam96b	1.64E+01
gene41087	67843	NC_000084.6	Slc35a4	1.64E+01
gene14943	668145	NC_000072.6	Gm9001	1.64E+01
gene15825	14228	NC_000072.6	Fkbp4	1.64E+01
gene16782	69094	NC_000073.6	Tmem160	1.64E+01
gene33365	621156	NC_000079.6	Apoo-ps	1.64E+01
gene10561	17965	NC_000070.6	Nbl1	1.64E+01
gene18312	66412	NC_000073.6	Arrdc4	1.63E+01
gene25420	103172	NC_000076.6	Chchd10	1.63E+01
gene23043	67010	NC_000075.6	Rbm7	1.63E+01
gene39122	27279	NC_000083.6	Tnfrsf12a	1.63E+01
gene22206	16412	NC_000074.6	Itgb1	1.62E+01
gene39805	14991	NC_000083.6	H2-M3	1.62E+01
gene11658	117197	NC_000071.6	Bloc1s4	1.62E+01
gene3640	666936	NC_000068.7	Gm14032	1.62E+01
gene23981	22041	NC_000075.6	Trf	1.61E+01
gene37116	66379	NC_000081.6	Cox14	1.61E+01
gene41256	1E+08	NC_000084.6	Eif3j2	1.61E+01
gene18	620009	NC_000067.6	Gm6123	1.61E+01
gene36547	19354	NC_000081.6	Rac2	1.61E+01
gene31053	ene=LOC1	NC_000078.6	LOC108168031	1.60E+01
gene32335	18081	NC_000079.6	Ninj1	1.60E+01
gene10900	ene=LOC1	NC_000070.6	LOC108168715	1.60E+01
gene5051	545461	NC_000068.7	Gm14026	1.60E+01
gene28251	327978	NC_000077.6	Slfn5	1.60E+01
gene39720	15042	NC_000083.6	H2-T24	1.60E+01
gene11569	22629	NC_000071.6	Ywhah	1.60E+01
gene29862	72123	NC_000078.6	Ccdc71l	1.60E+01
gene39400	56462	NC_000083.6	Mtch1	1.60E+01
gene20435	69068	NC_000074.6	1810011O10Rik	1.60E+01
gene41366	19038	NC_000084.6	Ppic	1.60E+01
gene21331	21672	NC_000074.6	Prdx2	1.59E+01
gene12878	67397	NC_000071.6	Erp29	1.59E+01
gene14778	381831	NC_000072.6	Igkv4-58	1.59E+01
gene14782	235952	NC_000072.6	Igkv4-57	1.59E+01
gene6592	1E+08	NC_000069.6	Gm2965	1.59E+01
gene7895	1E+08	NC_000069.6	Gm4596	1.59E+01
gene11828	666377	NC_000071.6	Gm8069	1.58E+01
gene19096	16202	NC_000073.6	Ilk	1.58E+01
gene12445	545790	NC_000071.6	Gm5870	1.58E+01
gene41085	24068	NC_000084.6	Sra1	1.58E+01
gene3092	227620	NC_000068.7	Uap1l1	1.58E+01
gene19594	101502	NC_000073.6	Hsd3b7	1.58E+01

gene42089	67674	NC_000085.6	Trmt112	1.58E+01
gene29974	27362	NC_000078.6	Dnajb9	1.57E+01
gene38876	67912	NC_000083.6	1600012H06Rik	1.57E+01
gene346	214854	NC_000067.6	Neurl3	1.57E+01
gene25640	17347	NC_000076.6	Mknk2	1.57E+01
gene499	12825	NC_000067.6	Col3a1	1.57E+01
gene40598	12314	NC_000083.6	Calm2	1.57E+01
gene15752	21937	NC_000072.6	Tnfrsf1a	1.57E+01
gene23784	66111	NC_000075.6	Tmed3	1.57E+01
gene40934	271505	NC_000084.6	Gm5064	1.56E+01
gene6171	66496	NC_000068.7	Ppdpf	1.56E+01
gene21007	66462	NC_000074.6	2810428I15Rik	1.56E+01
gene29209	22378	NC_000077.6	Wbp2	1.56E+01
gene12884	11669	NC_000071.6	Aldh2	1.56E+01
gene5689	67996	NC_000068.7	Srsf6	1.56E+01
gene23284	15211	NC_000075.6	Hexa	1.55E+01
gene25929	1E+08	NC_000076.6	Gm3571	1.55E+01
gene7185	83409	NC_000069.6	Lamtor2	1.55E+01
gene14820	677858	NC_000072.6	Igkv8-24	1.55E+01
gene39626	193740	NC_000083.6	Hspa1a	1.55E+01
gene42156	17254	NC_000085.6	Slc3a2	1.55E+01
gene10074	19063	NC_000070.6	Ppt1	1.55E+01
gene1701	27280	NC_000067.6	Phlda3	1.55E+01
gene39540	14976	NC_000083.6	Pfdn6	1.55E+01
gene5560	19124	NC_000068.7	Procr	1.55E+01
gene10505	12540	NC_000070.6	Cdc42	1.54E+01
gene2262	140559	NC_000067.6	Igsf8	1.54E+01
gene36491	117171	NC_000081.6	1110038F14Rik	1.54E+01
gene12875	231712	NC_000071.6	Trafd1	1.54E+01
gene14760	385109	NC_000072.6	Igkv4-72	1.54E+01
gene12531	11958	NC_000071.6	Atp5k	1.54E+01
gene27858	66172	NC_000077.6	Med11	1.54E+01
gene17941	18220	NC_000073.6	Nucb1	1.54E+01
gene29515	20969	NC_000078.6	Sdc1	1.54E+01
gene66	59014	NC_000067.6	Rrs1	1.54E+01
gene38623	15312	NC_000082.6	Hmgn1	1.53E+01
gene11034	20318	NC_000070.6	Sdf4	1.53E+01
gene22708	21376	NC_000075.6	Tbrg1	1.53E+01
gene8522	1E+08	NC_000070.6	Gm12918	1.53E+01
gene981	433326	NC_000067.6	Gm5529	1.53E+01
gene12745	69076	NC_000071.6	Triap1	1.53E+01
gene5081	170718	NC_000068.7	Idh3b	1.53E+01
gene7446	69769	NC_000069.6	Tnfaip8l2	1.53E+01
gene28229	20296	NC_000077.6	Ccl2	1.53E+01
gene39645	13001	NC_000083.6	Csnk2b	1.53E+01
gene21027	234384	NC_000074.6	Mpv17l2	1.53E+01
gene704	667100	NC_000067.6	Rpl18-ps1	1.53E+01
gene39945	73836	NC_000083.6	Slc35b2	1.52E+01

gene18228	384631	NC_000073.6	Gm5333	1.52E+01
gene25135	56442	NC_000076.6	Serinc1	1.52E+01
gene9315	242497	NC_000070.6	Gm11224	1.52E+01
gene15451	68089	NC_000072.6	Arpc4	1.52E+01
gene26475	622655	NC_000076.6	Rpsa-ps2	1.52E+01
gene7146	18008	NC_000069.6	Nes	1.52E+01
gene374	76178	NC_000067.6	Coa5	1.52E+01
gene26187	12461	NC_000076.6	Cct2	1.52E+01
gene13394	19353	NC_000071.6	Rac1	1.52E+01
gene21989	67443	NC_000074.6	Map1lc3b	1.51E+01
gene27247	12330	NC_000077.6	Canx	1.51E+01
gene32486	212032	NC_000079.6	Hk3	1.51E+01
gene13159	17448	NC_000071.6	Mdh2	1.51E+01
gene20807	76687	NC_000074.6	Spcs3	1.50E+01
gene5721	54401	NC_000068.7	Ywhab	1.50E+01
gene7374	20194	NC_000069.6	S100a10	1.50E+01
gene16420	628030	NC_000073.6	Gm6828	1.50E+01
gene22463	12539	NC_000075.6	Cdc37	1.50E+01
gene13135	66114	NC_000071.6	Dnajc30	1.50E+01
gene28686	16010	NC_000077.6	Igfbp4	1.50E+01
gene15750	17000	NC_000072.6	Ltbr	1.50E+01
gene1668	72674	NC_000067.6	Adipor1	1.50E+01
gene23078	66925	NC_000075.6	Sdhd	1.50E+01
gene36563	1E+08	NC_000081.6	Gm30368	1.50E+01
gene15057	108176	NC_000072.6	Npm3-ps1	1.49E+01
gene15011	57377	NC_000072.6	Mogs	1.49E+01
gene24480	12772	NC_000075.6	Ccr2	1.49E+01
gene41098	66492	NC_000084.6	Zmat2	1.49E+01
gene8146	ene=LOC1	NC_000069.6	LOC102633210	1.49E+01
gene28054	17913	NC_000077.6	Myo1c	1.49E+01
gene21326	13423	NC_000074.6	Dnase2a	1.49E+01
gene13755	66520	NC_000072.6	2610001J05Rik	1.49E+01
gene23000	21345	NC_000075.6	Tagln	1.49E+01
gene28791	19345	NC_000077.6	Rab5c	1.49E+01
gene477	gene=LOC1	NC_000067.6	LOC102632770	1.48E+01
gene20929	1E+08	NC_000074.6	Gm9755	1.48E+01
gene37640	1E+08	NC_000082.6	Gm10241	1.48E+01
gene8925	21894	NC_000070.6	Tln1	1.48E+01
gene37659	21762	NC_000082.6	Psmd2	1.47E+01
gene17872	107503	NC_000073.6	Atf5	1.47E+01
gene42293	ene=LOC1	NC_000085.6	LOC108168403	1.47E+01
gene4411	11432	NC_000068.7	Acp2	1.47E+01
gene3111	75454	NC_000068.7	Phpt1	1.47E+01
gene42525	672195	NC_000085.6	Gm10053	1.47E+01
gene28167	18126	NC_000077.6	Nos2	1.47E+01
gene27768	245828	NC_000077.6	Trappc1	1.47E+01
gene13241	69871	NC_000071.6	Ppp1r35	1.47E+01
gene3125	107771	NC_000068.7	Bmyc	1.46E+01

gene9190	14700	NC_000070.6	Gng10	1.46E+01
gene34440	58809	NC_000080.6	Rnase4	1.46E+01
gene32239	70078	NC_000079.6	Nol7	1.46E+01
gene41924	66990	NC_000085.6	Tmem134	1.46E+01
gene15162	103963	NC_000072.6	Rpn1	1.46E+01
gene23483	12306	NC_000075.6	Anxa2	1.46E+01
gene24383	68969	NC_000075.6	Eif1b	1.46E+01
gene28957	ene=LOC1	NC_000077.6	LOC108167927	1.46E+01
gene35841	624863	NC_000081.6	Gm6533	1.45E+01
gene3157	353156	NC_000068.7	Egfl7	1.45E+01
gene11710	208522	NC_000071.6	Gm4754	1.45E+01
gene12047	74318	NC_000071.6	Hopx	1.45E+01
gene11508	100952	NC_000071.6	Emilin1	1.45E+01
gene5375	13011	NC_000068.7	Cst7	1.45E+01
gene7494	67220	NC_000069.6	Plekho1	1.45E+01
gene4935	67092	NC_000068.7	Gatm	1.45E+01
gene34734	68836	NC_000080.6	Mrpl52	1.45E+01
gene29184	69674	NC_000077.6	Mif4gd	1.45E+01
gene25633	216169	NC_000076.6	Abhd17a	1.45E+01
gene25178	74747	NC_000076.6	Ddit4	1.45E+01
gene41904	225887	NC_000085.6	Ndufs8	1.45E+01
gene38134	672778	NC_000082.6	Gm9575	1.45E+01
gene20293	1E+08	NC_000074.6	Gm15359	1.45E+01
gene33913	20088	NC_000080.6	Rps24	1.44E+01
gene22517	66126	NC_000075.6	Elof1	1.44E+01
gene643	66882	NC_000067.6	Bzw1	1.44E+01
gene39691	433102	NC_000083.6	Sfta2	1.44E+01
gene7620	545556	NC_000069.6	Gm12428	1.44E+01
gene35732	621001	NC_000081.6	Rpl19-ps6	1.44E+01
gene41915	17995	NC_000085.6	Ndufv1	1.44E+01
gene22487	69773	NC_000075.6	1810026J23Rik	1.44E+01
gene8086	68436	NC_000069.6	Rpl34	1.43E+01
gene12724	23962	NC_000071.6	Oasl2	1.43E+01
gene360	12859	NC_000067.6	Cox5b	1.43E+01
gene7213	14466	NC_000069.6	Gba	1.43E+01
gene28502	217124	NC_000077.6	Ppp1r9b	1.43E+01
gene9174	22166	NC_000070.6	Txn1	1.42E+01
gene32201	621629	NC_000079.6	Gm6245	1.42E+01
gene42172	68693	NC_000085.6	Hnrnpul2	1.42E+01
gene1241	108657	NC_000067.6	Rnpepl1	1.42E+01
gene37633	239739	NC_000082.6	Lamp3	1.42E+01
gene12873	19988	NC_000071.6	Rpl6	1.42E+01
gene6007	14683	NC_000068.7	Gnas	1.42E+01
gene36204	66218	NC_000081.6	Ndufb9	1.42E+01
gene19893	15461	NC_000073.6	Hras	1.42E+01
gene13996	14489	NC_000072.6	Mtpn	1.41E+01
gene6797	69227	NC_000069.6	Selt	1.41E+01
gene23044	434402	NC_000075.6	Gm5617	1.41E+01

gene2977	12238	NC_000068.7	Commd3	1.41E+01
gene32686	630951	NC_000079.6	Gm7049	1.41E+01
gene3080	227615	NC_000068.7	Tmem203	1.41E+01
gene25623	17192	NC_000076.6	Mbd3	1.41E+01
gene37205	16691	NC_000081.6	Krt8	1.41E+01
gene10444	29876	NC_000070.6	Clic4	1.41E+01
gene32545	21810	NC_000079.6	Tgfbi	1.40E+01
gene40666	621501	NC_000084.6	Gm6235	1.40E+01
gene3623	625631	NC_000068.7	Bloc1s2-ps	1.40E+01
gene26479	66844	NC_000076.6	Ormdl2	1.40E+01
gene8261	93684	NC_000069.6	sep-15	1.40E+01
gene5579	67067	NC_000068.7	Romo1	1.40E+01
gene31972	79555	NC_000079.6	BC005537	1.40E+01
gene24085	15587	NC_000075.6	Hyal2	1.40E+01
gene19044	233637	NC_000073.6	Gm4887	1.39E+01
gene19980	12520	NC_000073.6	Cd81	1.39E+01
gene28995	19184	NC_000077.6	Psmc5	1.39E+01
gene12908	56378	NC_000071.6	Arpc3	1.39E+01
gene38947	670250	NC_000083.6	Gm9481	1.39E+01
gene19613	16411	NC_000073.6	Itgax	1.39E+01
gene6115	1E+08	NC_000068.7	Gm14292	1.39E+01
gene36228	399603	NC_000081.6	Fam84b	1.39E+01
gene33539	1E+08	NC_000079.6	Gm21370	1.39E+01
gene10534	625646	NC_000070.6	Rpl38-ps1	1.39E+01
gene15740	66493	NC_000072.6	Mrpl51	1.39E+01
gene30587	217737	NC_000078.6	Ahsa1	1.39E+01
gene28103	1E+08	NC_000077.6	Gm12734	1.39E+01
gene34328	63985	NC_000080.6	Gmfb	1.38E+01
gene28283	1E+08	NC_000077.6	E230016K23Rik	1.38E+01
gene21300	101966	NC_000074.6	D8Ertd738e	1.38E+01
gene35283	1E+08	NC_000080.6	Fkbp1a-ps1	1.38E+01
gene4937	433470	NC_000068.7	AA467197	1.38E+01
gene26590	ene=LOC1	NC_000077.6	LOC108167876	1.38E+01
gene13487	68895	NC_000071.6	Rasl11a	1.38E+01
gene39248	56047	NC_000083.6	Msln	1.38E+01
gene33543	1E+08	NC_000079.6	Gm31544	1.38E+01
gene20696	211895	NC_000074.6	Smarce1-ps1	1.37E+01
gene39555	14979	NC_000083.6	H2-Ke6	1.37E+01
gene31068	12709	NC_000078.6	Ckb	1.37E+01
gene1939	1E+08	NC_000067.6	Gm10031	1.37E+01
gene37661	208643	NC_000082.6	Eif4g1	1.37E+01
gene25159	268301	NC_000076.6	Sowahc	1.37E+01
gene21266	26364	NC_000074.6	Adgre5	1.37E+01
gene16030	627022	NC_000072.6	Gm6728	1.37E+01
gene24084	80385	NC_000075.6	Tusc2	1.37E+01
gene28056	22627	NC_000077.6	Ywhae	1.37E+01
gene15002	64704	NC_000072.6	Htra2	1.37E+01
gene1984	75255	NC_000067.6	4930562F07Rik	1.37E+01

gene28499	12842	NC_000077.6	Col1a1	1.37E+01
gene29183	50529	NC_000077.6	Mrps7	1.36E+01
gene14915	12332	NC_000072.6	Capg	1.36E+01
gene6842	74012	NC_000069.6	Rap2b	1.36E+01
gene24036	11655	NC_000075.6	Alas1	1.36E+01
gene32951	621824	NC_000079.6	Gm6263	1.36E+01
gene28092	1E+08	NC_000077.6	Gm12345	1.36E+01
gene27830	11370	NC_000077.6	Acadvl	1.36E+01
gene13479	667172	NC_000071.6	Gm8494	1.36E+01
gene39925	83965	NC_000083.6	Enpp5	1.36E+01
gene13220	74097	NC_000071.6	Pop7	1.36E+01
gene12058	29817	NC_000071.6	Igfbp7	1.35E+01
gene25649	18245	NC_000076.6	Oaz1	1.35E+01
gene40587	433144	NC_000083.6	Gm5499	1.35E+01
gene4024	72657	NC_000068.7	2700094K13Rik	1.35E+01
gene13982	68487	NC_000072.6	Tmem140	1.35E+01
gene32572	15387	NC_000079.6	Hnrnpk	1.35E+01
gene22264	71929	NC_000075.6	Tmem123	1.35E+01
gene10939	19934	NC_000070.6	Rpl22	1.35E+01
gene41975	108902	NC_000085.6	B4gat1	1.35E+01
gene11621	16976	NC_000071.6	Lrpap1	1.35E+01
gene10723	666215	NC_000070.6	Gm13127	1.35E+01
gene11012	=Gene;ger	NC_000070.6	LOC102636313	1.34E+01
gene36853	223775	NC_000081.6	Pim3	1.34E+01
gene19526	56420	NC_000073.6	Ppp4c	1.34E+01
gene39599	12915	NC_000083.6	Atf6b	1.34E+01
gene41916	14870	NC_000085.6	Gstp1	1.34E+01
gene8527	ene=LOC1	NC_000070.6	LOC102638891	1.34E+01
gene7211	1E+08	NC_000069.6	Gm16069	1.34E+01
gene7238	229534	NC_000069.6	Pbxip1	1.34E+01
gene34843	14939	NC_000080.6	Gzmb	1.34E+01
gene5253	56431	NC_000068.7	Dstn	1.34E+01
gene25471	108705	NC_000076.6	Pttg1ip	1.34E+01
gene24121	102791	NC_000075.6	Tcta	1.34E+01
gene16866	272359	NC_000073.6	Irf2bp1	1.33E+01
gene16656	21849	NC_000073.6	Trim28	1.33E+01
gene13238	624083	NC_000071.6	Gm15753	1.33E+01
gene38914	ene=LOC1	NC_000083.6	LOC108168360	1.33E+01
gene25700	13611	NC_000076.6	S1pr4	1.33E+01
gene39389	12575	NC_000083.6	Cdkn1a	1.33E+01
gene25678	66047	NC_000076.6	Mrpl54	1.33E+01
gene39997	17256	NC_000083.6	Mea1	1.33E+01
gene36410	69146	NC_000081.6	Gsdmd	1.33E+01
gene32659	1E+08	NC_000079.6	Gm19985	1.33E+01
gene8936	242408	NC_000070.6	Fam221b	1.33E+01
gene32209	66154	NC_000079.6	Tmem14c	1.33E+01
gene14765	384515	NC_000072.6	Igkv4-68	1.33E+01
gene18188	1E+08	NC_000073.6	Gm10297	1.32E+01

gene28282	20308	NC_000077.6	Ccl9	1.32E+01
gene923	22145	NC_000067.6	Tuba4a	1.32E+01
gene5354	241715	NC_000068.7	Gm14131	1.32E+01
gene25853	71712	NC_000076.6	Dram1	1.32E+01
gene35456	211286	NC_000080.6	Cln5	1.32E+01
gene42885	67264	NC_000085.6	Ndufb8	1.32E+01
gene1880	626096	NC_000067.6	Gm6652	1.32E+01
gene2733	666495	NC_000068.7	Gm13181	1.32E+01
gene19568	1E+08	NC_000073.6	Gm33080	1.32E+01
gene3041	215253	NC_000068.7	Gm13410	1.32E+01
gene40056	83433	NC_000083.6	Trem2	1.32E+01
gene26440	56530	NC_000076.6	Cnpy2	1.32E+01
gene25410	13202	NC_000076.6	Ddt	1.32E+01
gene33903	620499	NC_000080.6	Gm6158	1.32E+01
gene37924	209200	NC_000082.6	Dtx3l	1.31E+01
gene1942	30935	NC_000067.6	Tor3a	1.31E+01
gene19524	66090	NC_000073.6	Ypel3	1.31E+01
gene24284	67213	NC_000075.6	Cmtm6	1.31E+01
gene9724	56374	NC_000070.6	Tmem59	1.31E+01
gene39164	654822	NC_000083.6	D330041H03Rik	1.31E+01
gene7692	18176	NC_000069.6	Nras	1.31E+01
gene6924	667207	NC_000069.6	Gm8515	1.31E+01
gene21057	77090	NC_000074.6	Ocel1	1.31E+01
gene17921	12493	NC_000073.6	Cd37	1.31E+01
gene20582	67887	NC_000074.6	Saraf	1.31E+01
gene12104	545767	NC_000071.6	Gm5869	1.31E+01
gene18460	18550	NC_000073.6	Furin	1.31E+01
gene7885	22329	NC_000069.6	Vcam1	1.31E+01
gene30036	1E+08	NC_000078.6	Gm32220	1.31E+01
gene21736	68023	NC_000074.6	Pdf	1.31E+01
gene5405	14225	NC_000068.7	Fkbp1a	1.30E+01
gene935	74241	NC_000067.6	Chpf	1.30E+01
gene2999	71198	NC_000068.7	Otud1	1.30E+01
gene39282	68611	NC_000083.6	Mrpl28	1.30E+01
gene4045	20833	NC_000068.7	Ssrp1	1.30E+01
gene15997	12576	NC_000072.6	Cdkn1b	1.30E+01
gene7466	13038	NC_000069.6	Ctsk	1.30E+01
gene15580	101358	NC_000072.6	Fbxl14	1.29E+01
gene19600	27973	NC_000073.6	Vkorc1	1.29E+01
gene17343	51798	NC_000073.6	Ech1	1.29E+01
gene33970	11843	NC_000080.6	Arf4	1.29E+01
gene7188	66256	NC_000069.6	Ssr2	1.29E+01
gene22944	12282	NC_000075.6	Hyou1	1.29E+01
gene26882	12464	NC_000077.6	Cct4	1.29E+01
gene37339	1E+08	NC_000082.6	Gm16861	1.29E+01
gene24033	67905	NC_000075.6	Ppm1m	1.29E+01
gene27745	71923	NC_000077.6	Borcs6	1.29E+01
gene1285	666025	NC_000067.6	Gm7895	1.28E+01

gene13779	12389	NC_000072.6	Cav1	1.28E+01
gene28321	12351	NC_000077.6	Car4	1.28E+01
gene10260	76799	NC_000070.6	Tmem234	1.28E+01
gene21256	66916	NC_000074.6	Ndufb7	1.28E+01
gene2214	226646	NC_000067.6	Ndufs2	1.28E+01
gene31553	1E+08	NC_000079.6	Gm2423	1.28E+01
gene19541	67278	NC_000073.6	Pagr1a	1.28E+01
gene7881	626061	NC_000069.6	Gm6649	1.28E+01
gene17210	26362	NC_000073.6	Axl	1.27E+01
gene42230	13194	NC_000085.6	Ddb1	1.27E+01
gene13652	66066	NC_000072.6	Gng11	1.27E+01
gene15171	53421	NC_000072.6	Sec61a1	1.27E+01
gene15701	15170	NC_000072.6	Ptpn6	1.27E+01
gene41346	1E+08	NC_000084.6	Gm3815	1.27E+01
gene39651	53761	NC_000083.6	Prrc2a	1.27E+01
gene39963	22339	NC_000083.6	Vegfa	1.27E+01
gene3069	67122	NC_000068.7	Nrarp	1.27E+01
gene6579	24063	NC_000069.6	Spry1	1.27E+01
gene16765	259300	NC_000073.6	Ehd2	1.27E+01
gene30374	1E+08	NC_000078.6	Gm19025	1.27E+01
gene4564	433458	NC_000068.7	Gm13880	1.26E+01
gene16762	68077	NC_000073.6	Gltscr2	1.26E+01
gene27190	1.1E+08	NC_000077.6	Gm42074	1.26E+01
gene23660	12866	NC_000075.6	Cox7a2	1.26E+01
gene32287	544928	NC_000079.6	Gm5790	1.26E+01
gene25645	78670	NC_000076.6	Plekhj1	1.26E+01
gene32496	66890	NC_000079.6	Lman2	1.26E+01
gene27629	626482	NC_000077.6	Wsb2-ps	1.26E+01
gene10863	110208	NC_000070.6	Pgd	1.26E+01
gene42676	15957	NC_000085.6	Ifit1	1.26E+01
gene42200	619371	NC_000085.6	Stxbp3-ps	1.25E+01
gene14661	66870	NC_000072.6	Serbp1	1.25E+01
gene10908	22319	NC_000070.6	Vamp3	1.25E+01
gene5603	67388	NC_000068.7	1110008F13Rik	1.25E+01
gene39926	224794	NC_000083.6	Enpp4	1.25E+01
gene10236	75234	NC_000070.6	Rnf19b	1.24E+01
gene7804	14862	NC_000069.6	Gstm1	1.24E+01
gene10134	68920	NC_000070.6	1110065P20Rik	1.24E+01
gene32613	14451	NC_000079.6	Gas1	1.24E+01
gene28802	27419	NC_000077.6	Naglu	1.24E+01
gene32418	23882	NC_000079.6	Gadd45g	1.24E+01
gene7101	20091	NC_000069.6	Rps3a1	1.24E+01
gene10556	66825	NC_000070.6	Rnf186	1.24E+01
gene22521	66268	NC_000075.6	Pigyl	1.24E+01
gene31447	14569	NC_000079.6	Gdi2	1.24E+01
gene29801	20115	NC_000078.6	Rps7	1.24E+01
gene15041	21766	NC_000072.6	Tex261	1.23E+01
gene36695	67130	NC_000081.6	Ndufa6	1.23E+01

gene5571	66366	NC_000068.7	Ergic3	1.23E+01
gene6443	68028	NC_000069.6	Rpl22l1	1.23E+01
gene42216	69038	NC_000085.6	Tmem258	1.23E+01
gene22702	69524	NC_000075.6	Esam	1.23E+01
gene39202	26426	NC_000083.6	Nubp2	1.23E+01
gene19907	21351	NC_000073.6	Taldo1	1.23E+01
gene6424	22035	NC_000069.6	Tnfsf10	1.23E+01
gene31422	627557	NC_000078.6	Gm6768	1.23E+01
gene5066	19261	NC_000068.7	Sirpa	1.23E+01
gene21079	66171	NC_000074.6	Pgls	1.23E+01
gene7803	14863	NC_000069.6	Gstm2	1.23E+01
gene16002	232431	NC_000072.6	Gprc5a	1.23E+01
gene42018	68209	NC_000085.6	Rnaseh2c	1.23E+01
gene19546	101602	NC_000073.6	AI467606	1.23E+01
gene21130	66818	NC_000074.6	Smim7	1.22E+01
gene886	18173	NC_000067.6	Slc11a1	1.22E+01
gene27198	1E+08	NC_000077.6	Gm12188	1.22E+01
gene28490	111266	NC_000077.6	Cdc34b	1.22E+01
gene22183	270110	NC_000074.6	Irf2bp2	1.22E+01
gene4898	tor 4;gbkey	NC_000068.7	Serinc4	1.22E+01
gene21081	234407	NC_000074.6	Colgalt1	1.22E+01
gene28485	98238	NC_000077.6	Lrrc59	1.22E+01
gene39635	51793	NC_000083.6	Ddah2	1.22E+01
gene9280	66290	NC_000070.6	Atp6v1g1	1.22E+01
gene34425	18950	NC_000080.6	Pnp	1.22E+01
gene6769	17112	NC_000069.6	Tm4sf1	1.22E+01
gene14816	434041	NC_000072.6	Igkv8-27	1.22E+01
gene34802	67881	NC_000080.6	Mdp1	1.22E+01
gene36427	67959	NC_000081.6	Puf60	1.21E+01
gene17350	73830	NC_000073.6	Eif3k	1.21E+01
gene15464	101314	NC_000072.6	Brk1	1.21E+01
gene38409	12469	NC_000082.6	Cct8	1.21E+01
gene30840	628900	NC_000078.6	Serpina3i	1.21E+01
gene32518	78521	NC_000079.6	B230219D22Rik	1.21E+01
gene28156	21927	NC_000077.6	Tnfaip1	1.21E+01
gene28815	28084	NC_000077.6	Vps25	1.21E+01
gene29409	217370	NC_000077.6	BC017643	1.21E+01
gene27564	14248	NC_000077.6	Flii	1.21E+01
gene18862	56212	NC_000073.6	Rhog	1.20E+01
gene27794	1E+08	NC_000077.6	Tnfsf13os	1.20E+01
gene21754	17463	NC_000074.6	Psmd7	1.20E+01
gene27828	78246	NC_000077.6	Phf23	1.20E+01
gene40813	623867	NC_000084.6	Gm6457	1.20E+01
gene40120	1E+08	NC_000083.6	Mrps36-ps1	1.20E+01
gene21252	546077	NC_000074.6	Gm5910	1.20E+01
gene21942	72042	NC_000074.6	Cotl1	1.20E+01
gene39318	12018	NC_000083.6	Bak1	1.20E+01
gene17364	72275	NC_000073.6	2200002D01Rik	1.20E+01

gene24122	11848	NC_000075.6	Rhoa	1.19E+01
gene13072	110006	NC_000071.6	Gusb	1.19E+01
gene19308	26440	NC_000073.6	Psma1	1.19E+01
gene18018	67893	NC_000073.6	Tmem86a	1.19E+01
gene5564	16418	NC_000068.7	Eif6	1.19E+01
gene37062	11842	NC_000081.6	Arf3	1.19E+01
gene35743	268781	NC_000081.6	Gm5043	1.18E+01
gene21820	72544	NC_000074.6	Exosc6	1.18E+01
gene28475	71452	NC_000077.6	Ankrd40	1.18E+01
gene35573	1E+08	NC_000080.6	Gm4674	1.18E+01
gene6214	383815	NC_000069.6	Rps24-ps2	1.18E+01
gene24392	1E+08	NC_000075.6	Gm18101	1.18E+01
gene24136	66706	NC_000075.6	Ndufaf3	1.18E+01
gene36485	66398	NC_000081.6	Commd5	1.18E+01
gene17295	23996	NC_000073.6	Psmc4	1.18E+01
gene39621	18010	NC_000083.6	Neu1	1.18E+01
gene10441	68592	NC_000070.6	Syf2	1.18E+01
gene24140	83669	NC_000075.6	Wdr6	1.17E+01
gene13231	1E+08	NC_000071.6	Gm16089	1.17E+01
gene6414	621017	NC_000069.6	Gm6197	1.17E+01
gene11755	66988	NC_000071.6	Lap3	1.17E+01
gene7831	67495	NC_000069.6	Tmem167b	1.17E+01
gene21209	546075	NC_000074.6	Gm5909	1.17E+01
gene41841	66054	NC_000084.6	Cndp2	1.17E+01
gene5152	18538	NC_000068.7	Pcna	1.17E+01
gene5330	15376	NC_000068.7	Foxa2	1.16E+01
gene2176	246256	NC_000067.6	Fcgr4	1.16E+01
gene11148	117167	NC_000071.6	Steap4	1.16E+01
gene42166	67706	NC_000085.6	Tmem179b	1.16E+01
gene23087	69253	NC_000075.6	Hspb2	1.16E+01
gene29868	59027	NC_000078.6	Nampt	1.16E+01
gene12294	666231	NC_000071.6	Gm7993	1.16E+01
gene40003	67101	NC_000083.6	2310039H08Rik	1.16E+01
gene29223	15223	NC_000077.6	Foxj1	1.16E+01
gene27310	21402	NC_000077.6	Skp1a	1.16E+01
gene14800	619960	NC_000072.6	Igkv12-41	1.16E+01
gene3795	20823	NC_000068.7	Ssb	1.15E+01
gene6185	72699	NC_000068.7	Lime1	1.15E+01
gene24104	74153	NC_000075.6	Uba7	1.15E+01
gene18846	66508	NC_000073.6	Lamtor1	1.15E+01
gene13336	16848	NC_000071.6	Lfng	1.15E+01
gene19426	80719	NC_000073.6	Igsf6	1.15E+01
gene14789	546213	NC_000072.6	Igkv4-53	1.15E+01
gene28999	15985	NC_000077.6	Cd79b	1.15E+01
gene15105	66881	NC_000072.6	Pcyox1	1.15E+01
gene1818	19225	NC_000067.6	Ptgs2	1.15E+01
gene10431	76824	NC_000070.6	Mtfr1l	1.15E+01
gene24109	331026	NC_000075.6	Gmppb	1.15E+01

gene18415	17304	NC_000073.6	Mfge8	1.15E+01
gene35984	68036	NC_000081.6	Zfp706	1.15E+01
gene21880	17132	NC_000074.6	Maf	1.15E+01
gene12451	626578	NC_000071.6	Gbp10	1.14E+01
gene17286	404705	NC_000073.6	Prkcz2	1.14E+01
gene25141	57319	NC_000076.6	Smpdl3a	1.14E+01
gene42189	23942	NC_000085.6	Mta2	1.14E+01
gene3339	73737	NC_000068.7	1110008P14Rik	1.14E+01
gene15326	666019	NC_000072.6	Gm7892	1.14E+01
gene37272	80910	NC_000081.6	Gpr84	1.14E+01
gene23511	1E+08	NC_000075.6	Gm3436	1.14E+01
gene42072	240505	NC_000085.6	Cdc42bpg	1.14E+01
gene26788	56193	NC_000077.6	Plek	1.14E+01
gene32647	624140	NC_000079.6	Gm6474	1.14E+01
gene879	76709	NC_000067.6	Arpc2	1.14E+01
gene17402	69920	NC_000073.6	Polr2i	1.13E+01
gene42020	19697	NC_000085.6	Rela	1.13E+01
gene25591	216157	NC_000076.6	Tmem259	1.13E+01
gene17441	110323	NC_000073.6	Cox6b1	1.13E+01
gene19550	70233	NC_000073.6	Cd2bp2	1.13E+01
gene30072	12632	NC_000078.6	Cfl2	1.13E+01
gene1332	383650	NC_000067.6	Psmb6-ps2	1.13E+01
gene28872	217218	NC_000077.6	Atxn7l3	1.12E+01
gene27873	70510	NC_000077.6	Rnf167	1.12E+01
gene38579	12408	NC_000082.6	Cbr1	1.12E+01
gene3419	227753	NC_000068.7	Gsn	1.12E+01
gene7438	19185	NC_000069.6	Psmd4	1.12E+01
gene14912	232087	NC_000072.6	Mat2a	1.12E+01
gene36851	76737	NC_000081.6	Creld2	1.12E+01
gene39654	16988	NC_000083.6	Lst1	1.12E+01
gene19869	101489	NC_000073.6	Ric8a	1.12E+01
gene6770	383862	NC_000069.6	Gm5276	1.12E+01
gene33457	69590	NC_000079.6	Gpx8	1.12E+01
gene970	620521	NC_000067.6	Gm6159	1.12E+01
gene30543	14281	NC_000078.6	Fos	1.11E+01
gene24792	628697	NC_000076.6	Gm6906	1.11E+01
gene34791	28199	NC_000080.6	Dcaf11	1.11E+01
gene20445	69742	NC_000074.6	Tm2d2	1.11E+01
gene28820	19192	NC_000077.6	Psme3	1.11E+01
gene853	16011	NC_000067.6	Igfbp5	1.11E+01
gene2674	12490	NC_000067.6	Cd34	1.11E+01
gene37210	75705	NC_000081.6	Eif4b	1.11E+01
gene19868	54399	NC_000073.6	Bet1l	1.11E+01
gene8716	108755	NC_000070.6	Lyrm2	1.11E+01
gene10349	11983	NC_000070.6	Atpif1	1.11E+01
gene25585	109284	NC_000076.6	R3hdm4	1.11E+01
gene3176	20930	NC_000068.7	Surf1	1.11E+01
gene15724	26894	NC_000072.6	Cops7a	1.10E+01

gene8681	14703	NC_000070.6	Gng2-ps1	1.10E+01
gene20997	59042	NC_000074.6	Cope	1.10E+01
gene33882	22334	NC_000080.6	Vdac2	1.10E+01
gene848	19981	NC_000067.6	Rpl37a	1.10E+01
gene12502	72342	NC_000071.6	Gm9727	1.10E+01
gene10684	ene;gene=L	NC_000070.6	LOC100044633	1.10E+01
gene22325	1E+08	NC_000075.6	Mif-ps6	1.10E+01
gene24818	432447	NC_000076.6	Gm9824	1.10E+01
gene26593	114679	NC_000077.6	Selm	1.10E+01
gene3174	20933	NC_000068.7	Med22	1.10E+01
gene8435	1E+08	NC_000069.6	Gm17843	1.10E+01
gene28139	72503	NC_000077.6	2610507B11Rik	1.10E+01
gene7280	67860	NC_000069.6	S100a16	1.10E+01
gene39669	667636	NC_000083.6	Gm8741	1.10E+01
gene15311	65106	NC_000072.6	Arl6ip5	1.09E+01
gene34800	74140	NC_000080.6	Tm9sf1	1.09E+01
gene15870	1E+08	NC_000072.6	1700101I11Rik	1.09E+01
gene41934	19367	NC_000085.6	Rad9a	1.09E+01
gene13659	12843	NC_000072.6	Col1a2	1.09E+01
gene9885	66805	NC_000070.6	Tspan1	1.09E+01
gene15059	20751	NC_000072.6	Spr	1.09E+01
gene42071	13660	NC_000085.6	Ehd1	1.09E+01
gene14795	692245	NC_000072.6	Igkv12-46	1.09E+01
gene37213	16012	NC_000081.6	Igfbp6	1.09E+01
gene22302	665329	NC_000075.6	Gm7588	1.09E+01
gene11108	67210	NC_000071.6	Gatad1	1.09E+01
gene16917	1E+08	NC_000073.6	Gm34744	1.09E+01
gene12184	330122	NC_000071.6	Cxcl3	1.09E+01
gene39729	15024	NC_000083.6	H2-T10	1.09E+01
gene5958	1.1E+08	NC_000068.7	Gm40009	1.09E+01
gene13880	69573	NC_000072.6	Hilpda	1.09E+01
gene14369	93695	NC_000072.6	Gpnmb	1.09E+01
gene36144	105853	NC_000081.6	Mal2	1.08E+01
gene673	14369	NC_000067.6	Fzd7	1.08E+01
gene8917	21754	NC_000070.6	Tesk1	1.08E+01
gene22930	84004	NC_000075.6	Mcam	1.08E+01
gene12242	ene=LOC1	NC_000071.6	LOC108169055	1.08E+01
gene34872	668090	NC_000080.6	Gm8971	1.08E+01
gene29433	74173	NC_000078.6	Rab10os	1.08E+01
gene20413	1E+08	NC_000074.6	Gm18675	1.08E+01
gene1787	19735	NC_000067.6	Rgs2	1.08E+01
gene26376	1E+08	NC_000076.6	F420014N23Rik	1.08E+01
gene13161	15507	NC_000071.6	Hspb1	1.08E+01
gene33407	20620	NC_000079.6	Plk2	1.08E+01
gene13883	12321	NC_000072.6	Calu	1.08E+01
gene39650	224727	NC_000083.6	Bag6	1.08E+01
gene19709	18242	NC_000073.6	Oat	1.08E+01
gene41852	ene=LOC1	NC_000084.6	LOC101055696	1.08E+01

gene12520	73130	NC_000071.6	Tmed5	1.08E+01
gene10612	66147	NC_000070.6	Necap2	1.07E+01
gene21505	64209	NC_000074.6	Herpud1	1.07E+01
gene17365	20733	NC_000073.6	Spint2	1.07E+01
gene17938	12028	NC_000073.6	Bax	1.07E+01
gene21316	19358	NC_000074.6	Rad23a	1.07E+01
gene22947	60409	NC_000075.6	Trappc4	1.07E+01
gene41553	433193	NC_000084.6	Rps15-ps3	1.07E+01
gene36877	105847	NC_000081.6	Lmf2	1.07E+01
gene13782	12343	NC_000072.6	Capza2	1.07E+01
gene39668	15006	NC_000083.6	H2-Q1	1.07E+01
gene415	67702	NC_000067.6	Rnf149	1.07E+01
gene29362	27376	NC_000077.6	Slc25a10	1.07E+01
gene42589	1E+08	NC_000085.6	Gm36043	1.07E+01
gene17783	668630	NC_000073.6	Gm9278	1.07E+01
gene6839	666853	NC_000069.6	Gm8325	1.07E+01
gene17974	68137	NC_000073.6	Kdelr1	1.07E+01
gene8344	15061	NC_000069.6	Ifi44l	1.06E+01
gene31105	11651	NC_000078.6	Akt1	1.06E+01
gene2430	69051	NC_000067.6	Pycr2	1.06E+01
gene39704	76448	NC_000083.6	Ppp1r18	1.06E+01
gene42099	18938	NC_000085.6	Ppp1r14b	1.06E+01
gene26162	668071	NC_000076.6	Gm8960	1.06E+01
gene2623	1E+08	NC_000067.6	Gm20203	1.06E+01
gene9427	624465	NC_000070.6	Gm12420	1.06E+01
gene42213	76267	NC_000085.6	Fads1	1.06E+01
gene27507	432565	NC_000077.6	Gm12263	1.06E+01
gene41903	27060	NC_000085.6	Tcirg1	1.06E+01
gene1206	55927	NC_000067.6	Hes6	1.06E+01
gene26737	626887	NC_000077.6	Gm11993	1.05E+01
gene27844	104457	NC_000077.6	0610010K14Rik	1.05E+01
gene14334	76252	NC_000072.6	Atp6v0e2	1.05E+01
gene26478	74330	NC_000076.6	Dnajc14	1.05E+01
gene25742	666611	NC_000076.6	Gm8188	1.05E+01
gene10391	55948	NC_000070.6	Sfn	1.05E+01
gene19601	12041	NC_000073.6	Bckdk	1.05E+01
gene12685	20345	NC_000071.6	Selplg	1.05E+01
gene26672	64660	NC_000077.6	Mrps24	1.05E+01
gene3095	18146	NC_000068.7	Npdc1	1.05E+01
gene23971	56332	NC_000075.6	Amotl2	1.05E+01
gene35926	1.1E+08	NC_000081.6	Gm41291	1.05E+01
gene36522	17886	NC_000081.6	Myh9	1.04E+01
gene4811	72136	NC_000068.7	Chst14	1.04E+01
gene22468	50868	NC_000075.6	Keap1	1.04E+01
gene10172	230752	NC_000070.6	Eva1b	1.04E+01
gene34761	54196	NC_000080.6	Pabpn1	1.04E+01
gene37222	56612	NC_000081.6	Pfdn5	1.04E+01
gene11830	666405	NC_000071.6	Gm8085	1.04E+01

gene7447	668436	NC_000069.6	Gm9169	1.04E+01
gene17590	12611	NC_000073.6	Cebpg	1.04E+01
gene9690	433742	NC_000070.6	Gm12722	1.04E+01
gene23454	22003	NC_000075.6	Tpm1	1.04E+01
gene25691	102115	NC_000076.6	Dohh	1.04E+01
gene38771	1E+08	NC_000083.6	Gm17087	1.04E+01
gene24708	235392	NC_000076.6	Gm4895	1.04E+01
gene6379	12870	NC_000069.6	Cp	1.04E+01
gene3053	664938	NC_000068.7	Gm13416	1.04E+01
gene248	665903	NC_000067.6	Gm7846	1.04E+01
gene33178	14062	NC_000079.6	F2r	1.04E+01
gene32935	218335	NC_000079.6	Clptm1l	1.04E+01
gene5261	69178	NC_000068.7	Snx5	1.04E+01
gene2219	66155	NC_000067.6	Ufc1	1.04E+01
gene36342	666113	NC_000081.6	Gm7935	1.04E+01
gene12241	12453	NC_000071.6	Ccni	1.04E+01
gene25818	21859	NC_000076.6	Timp3	1.03E+01
gene24133	97541	NC_000075.6	Qars	1.03E+01
gene2658	14373	NC_000067.6	G0s2	1.03E+01
gene25520	18114	NC_000076.6	Rrp1	1.03E+01
gene21498	17750	NC_000074.6	Mt2	1.03E+01
gene33011	382823	NC_000079.6	Atp5c1-ps	1.03E+01
gene25688	73822	NC_000076.6	Mfsd12	1.03E+01
gene17342	15388	NC_000073.6	Hnrnpl	1.03E+01
gene36446	14731	NC_000081.6	Gpaa1	1.03E+01
gene19507	56347	NC_000073.6	Eif3c	1.03E+01
gene30485	71952	NC_000078.6	2410016O06Rik	1.03E+01
gene14780	384514	NC_000072.6	Igkv4-57-1	1.03E+01
gene36534	12983	NC_000081.6	Csf2rb	1.03E+01
gene18410	57444	NC_000073.6	Isg20	1.03E+01
gene34795	19188	NC_000080.6	Psme2	1.03E+01
gene9739	433743	NC_000070.6	Gm12906	1.02E+01
gene12447	236573	NC_000071.6	Gbp9	1.02E+01
gene40399	240160	NC_000083.6	Gm4948	1.02E+01
gene42473	11668	NC_000085.6	Aldh1a1	1.02E+01
gene36595	67040	NC_000081.6	Ddx17	1.02E+01
gene3186	227659	NC_000068.7	Slc2a6	1.02E+01
gene25181	94214	NC_000076.6	Spock2	1.02E+01
gene29339	208501	NC_000077.6	1810043H04Rik	1.02E+01
gene21729	214987	NC_000074.6	Chtf8	1.02E+01
gene41314	628477	NC_000084.6	Gm6883	1.02E+01
gene15071	12468	NC_000072.6	Cct7	1.02E+01
gene28230	20306	NC_000077.6	Ccl7	1.02E+01
gene18703	68197	NC_000073.6	Ndufc2	1.01E+01
gene7244	70093	NC_000069.6	Ube2q1	1.01E+01
gene21315	102060	NC_000074.6	Gadd45gip1	1.01E+01
gene15751	20276	NC_000072.6	Scnn1a	1.01E+01
gene9964	666596	NC_000070.6	Gm12857	1.01E+01

gene16877	107686	NC_000073.6	Snrpd2	1.01E+01
gene38393	1E+08	NC_000082.6	Gm2541	1.01E+01
gene10530	68943	NC_000070.6	Pink1	1.01E+01
gene29471	626534	NC_000078.6	Gm6682	1.01E+01
gene14828	108024	NC_000072.6	Igkv6-20	1.01E+01
gene32528	212937	NC_000079.6	Tifab	1.01E+01
gene28163	69109	NC_000077.6	Fam58b	1.01E+01
gene18448	23991	NC_000073.6	Cib1	1.01E+01
gene20495	353310	NC_000074.6	Zfp703	1.01E+01
gene9646	230514	NC_000070.6	Leprot	1.01E+01
gene28996	83796	NC_000077.6	Smarcd2	1.01E+01
gene39972	20016	NC_000083.6	Polr1c	1.01E+01
gene41923	11632	NC_000085.6	Aip	1.01E+01
gene39267	68347	NC_000083.6	0610011F06Rik	1.01E+01
gene34423	11792	NC_000080.6	Apex1	1.01E+01
gene14815	434040	NC_000072.6	Igkv8-28	1.01E+01
gene41301	13664	NC_000084.6	Eif1a	1.01E+01
gene3981	320769	NC_000068.7	Prdx6b	1.01E+01
gene33870	18792	NC_000080.6	Plau	1.01E+01
gene34424	219024	NC_000080.6	Tmem55b	1.01E+01
gene12743	108014	NC_000071.6	Srsf9	1.00E+01
gene33805	66480	NC_000080.6	Rpl15	1.00E+01
gene22052	234852	NC_000074.6	Chmp1a	1.00E+01
gene18754	1E+08	NC_000073.6	Gm18943	1.00E+01
gene25297	211488	NC_000076.6	Ado	1.00E+01
gene2035	55990	NC_000067.6	Fmo2	1.00E+01
gene14349	16205	NC_000072.6	Gimap1	1.00E+01
gene21037	234388	NC_000074.6	Ccdc124	1.00E+01
gene255	433283	NC_000067.6	Gm5525	1.00E+01
gene29395	80879	NC_000077.6	Slc16a3	9.99E+00
gene7237	68911	NC_000069.6	Pygo2	9.98E+00
gene5819	433503	NC_000068.7	Gm29679	9.98E+00
gene11502	68796	NC_000071.6	Tmem214	9.96E+00
gene17884	1E+08	NC_000073.6	Gm31028	9.96E+00
gene12673	1.1E+08	NC_000071.6	Gm42002	9.96E+00
gene29003	15896	NC_000077.6	Icam2	9.95E+00
gene27621	69221	NC_000077.6	2410006H16Rik	9.94E+00
gene39734	667977	NC_000083.6	Gm8909	9.94E+00
gene14351	83408	NC_000072.6	Gimap3	9.94E+00
gene24064	109095	NC_000075.6	Rbm15b	9.94E+00
gene27284	72947	NC_000077.6	Phykpl	9.93E+00
gene17434	233073	NC_000073.6	U2af1l4	9.92E+00
gene21465	54352	NC_000074.6	Irx5	9.90E+00
gene28668	22123	NC_000077.6	Psmd3	9.89E+00
gene42615	83410	NC_000085.6	Cstf2t	9.89E+00
gene12183	56744	NC_000071.6	Pf4	9.88E+00
gene2947	227588	NC_000068.7	Gm13368	9.86E+00
gene4201	668432	NC_000068.7	Gm1826	9.86E+00

gene10259	54709	NC_000070.6	Eif3i	9.86E+00
gene8518	666840	NC_000070.6	Gm11814	9.86E+00
gene3107	ene=LOC1	NC_000068.7	LOC102638962	9.86E+00
gene30238	1E+08	NC_000078.6	Gm19221	9.85E+00
gene17265	55942	NC_000073.6	Sertad1	9.85E+00
gene3047	16181	NC_000068.7	Il1rn	9.85E+00
gene38086	11615	NC_000082.6	Gm4737	9.84E+00
gene26141	544737	NC_000076.6	Gm26596	9.84E+00
gene41483	12978	NC_000084.6	Csf1r	9.83E+00
gene21116	1E+08	NC_000074.6	Gm10282	9.82E+00
gene31693	432732	NC_000079.6	AK157302	9.81E+00
gene17942	17872	NC_000073.6	Ppp1r15a	9.81E+00
gene32493	19336	NC_000079.6	Rab24	9.81E+00
gene10569	626000	NC_000070.6	Ccnd3-ps	9.80E+00
gene40580	13819	NC_000083.6	Epas1	9.78E+00
gene39591	18515	NC_000083.6	Pbx2	9.76E+00
gene16015	13730	NC_000072.6	Emp1	9.76E+00
gene4645	22343	NC_000068.7	Lin7c	9.75E+00
gene615	626009	NC_000067.6	Gm6644	9.75E+00
gene39337	106672	NC_000083.6	AI413582	9.75E+00
gene27243	103534	NC_000077.6	Mgat4b	9.74E+00
gene7276	66511	NC_000069.6	Chtop	9.74E+00
gene14075	23845	NC_000072.6	Clec5a	9.73E+00
gene11734	12182	NC_000071.6	Bst1	9.72E+00
gene24743	22361	NC_000076.6	Vnn1	9.72E+00
gene30530	ubcomplex	NC_000078.6	LOC106345153	9.72E+00
gene29365	192173	NC_000077.6	Fam195b	9.71E+00
gene26477	73827	NC_000076.6	Tmem198b	9.70E+00
gene10439	71817	NC_000070.6	Tmem50a	9.69E+00
gene32571	70153	NC_000079.6	2210016F16Rik	9.69E+00
gene29146	140497	NC_000077.6	Cd300c2	9.68E+00
gene13314	17135	NC_000071.6	Mafk	9.67E+00
gene39718	14670	NC_000083.6	Gnl1	9.67E+00
gene10609	74772	NC_000070.6	Atp13a2	9.66E+00
gene14260	12304	NC_000072.6	Pdia4	9.65E+00
gene24151	22273	NC_000075.6	Uqcrc1	9.64E+00
gene5001	13537	NC_000068.7	Dusp2	9.64E+00
gene17925	68667	NC_000073.6	Trpm4	9.63E+00
gene7263	23922	NC_000069.6	Jtb	9.63E+00
gene30519	74316	NC_000078.6	Isca2	9.62E+00
gene33204	15212	NC_000079.6	Hexb	9.61E+00
gene34803	18002	NC_000080.6	Nedd8	9.59E+00
gene7140	67707	NC_000069.6	Mrpl24	9.59E+00
gene42169	67710	NC_000085.6	Polr2g	9.59E+00
gene28517	110172	NC_000077.6	Slc35b1	9.59E+00
gene1456	226351	NC_000067.6	Tmem185b	9.58E+00
gene42183	14376	NC_000085.6	Ganab	9.58E+00
gene24912	624165	NC_000076.6	Gm6477	9.58E+00

gene10223	384059	NC_000070.6	Tlr12	9.57E+00
gene26476	58223	NC_000076.6	Mmp19	9.57E+00
gene13191	66437	NC_000071.6	Fis1	9.56E+00
gene15430	434076	NC_000072.6	Gm15519	9.56E+00
gene34885	67840	NC_000080.6	Mrpl57	9.55E+00
gene40006	72726	NC_000083.6	Tbcc	9.54E+00
gene22027	59005	NC_000074.6	Trappc2l	9.54E+00
gene19625	628781	NC_000073.6	Gm6916	9.54E+00
gene27286	52530	NC_000077.6	Nhp2	9.53E+00
gene8148	=Gene;ger	NC_000069.6	LOC108168903	9.51E+00
gene27879	16562	NC_000077.6	Kif1c	9.51E+00
gene42578	67329	NC_000085.6	1700018L02Rik	9.51E+00
gene10066	666937	NC_000070.6	Gm12892	9.51E+00
gene13193	70103	NC_000071.6	Znhit1	9.50E+00
gene20163	665363	NC_000074.6	Gm7600	9.50E+00
gene36692	17939	NC_000081.6	Naga	9.49E+00
gene39536	81630	NC_000083.6	Zbtb22	9.46E+00
gene2513	226777	NC_000067.6	C130074G19Rik	9.45E+00
gene29225	668255	NC_000077.6	Rps11-ps2	9.45E+00
gene20187	1E+08	NC_000074.6	Gm15350	9.44E+00
gene27826	67181	NC_000077.6	Ctdnep1	9.44E+00
gene10834	50762	NC_000070.6	Fbxo6	9.43E+00
gene11004	192185	NC_000070.6	Nadk	9.43E+00
gene25699	103425	NC_000076.6	Ncln	9.43E+00
gene38288	ene=LOC1	NC_000082.6	LOC108168274	9.42E+00
gene23165	26441	NC_000075.6	Psma4	9.42E+00
gene12524	13486	NC_000071.6	Dr1	9.41E+00
gene18538	67943	NC_000073.6	Mesdc2	9.41E+00
gene39494	631266	NC_000083.6	Gm7059	9.41E+00
gene10907	1E+08	NC_000070.6	Gm38529	9.41E+00
gene33852	66242	NC_000080.6	Mrps16	9.39E+00
gene14791	381782	NC_000072.6	Igkv4-50	9.38E+00
gene39593	54197	NC_000083.6	Rnf5	9.38E+00
gene40592	193813	NC_000083.6	Mcfd2	9.37E+00
gene37986	11544	NC_000082.6	Adprh	9.37E+00
gene21344	414077	NC_000074.6	Wdr83os	9.37E+00
gene11515	381629	NC_000071.6	Atraid	9.37E+00
gene42063	74481	NC_000085.6	Batf2	9.37E+00
gene21075	73711	NC_000074.6	Mvb12a	9.37E+00
gene12354	50926	NC_000071.6	Hnrnpdl	9.37E+00
gene15861	232413	NC_000072.6	Clec12a	9.36E+00
gene13228	18542	NC_000071.6	Pcolce	9.36E+00
gene17443	68035	NC_000073.6	Rbm42	9.36E+00
gene41055	69816	NC_000084.6	Mzb1	9.36E+00
gene11829	1E+08	NC_000071.6	Gm15975	9.34E+00
gene25238	19073	NC_000076.6	Srgn	9.34E+00
gene24078	56395	NC_000075.6	Tmem115	9.34E+00
gene7279	66166	NC_000069.6	S100a14	9.34E+00

gene39163	27410	NC_000083.6	Abca3	9.33E+00
gene42670	15958	NC_000085.6	Ifit2	9.33E+00
gene3268	227697	NC_000068.7	Dolk	9.32E+00
gene26781	sphatase 1	NC_000077.6	LOC105274305	9.32E+00
gene35663	668700	NC_000080.6	Gm9308	9.32E+00
gene42946	54130	NC_000085.6	Actr1a	9.32E+00
gene27303	19052	NC_000077.6	Ppp2ca	9.32E+00
gene38374	547311	NC_000082.6	Gm6032	9.31E+00
gene28337	21385	NC_000077.6	Tbx2	9.30E+00
gene24131	16779	NC_000075.6	Lamb2	9.30E+00
gene25960	71207	NC_000076.6	Nudt4	9.30E+00
gene39642	193742	NC_000083.6	Abhd16a	9.30E+00
gene19618	12862	NC_000073.6	Cox6a2	9.29E+00
gene12232	52331	NC_000071.6	Stbd1	9.29E+00
gene42239	65221	NC_000085.6	Slc15a3	9.29E+00
gene17880	84113	NC_000073.6	Ptov1	9.27E+00
gene37743	67775	NC_000082.6	Rtp4	9.26E+00
gene28871	72053	NC_000077.6	Tmub2	9.26E+00
gene10736	21938	NC_000070.6	Tnfrsf1b	9.26E+00
gene8343	99899	NC_000069.6	Ifi44	9.26E+00
gene35179	213484	NC_000080.6	Nudt18	9.25E+00
gene4718	68032	NC_000068.7	Emc4	9.25E+00
gene9865	13120	NC_000070.6	Cyp4b1	9.25E+00
gene30317	15251	NC_000078.6	Hif1a	9.22E+00
gene6923	66868	NC_000069.6	Mfsd1	9.21E+00
gene39996	71765	NC_000083.6	Klhdc3	9.20E+00
gene9631	277707	NC_000070.6	Ccdc50-ps	9.19E+00
gene24049	235587	NC_000075.6	Parp3	9.18E+00
gene5099	77006	NC_000068.7	Ddrgk1	9.18E+00
gene30846	20716	NC_000078.6	Serpina3n	9.17E+00
gene15661	17474	NC_000072.6	Clec4d	9.16E+00
gene2752	1E+08	NC_000068.7	Gm13192	9.15E+00
gene32073	20708	NC_000079.6	Serpinb6b	9.14E+00
gene34476	214922	NC_000080.6	Slc39a2	9.13E+00
gene26153	668041	NC_000076.6	Gm8942	9.13E+00
gene6605	1E+08	NC_000069.6	Gm9845	9.12E+00
gene37921	547253	NC_000082.6	Parp14	9.12E+00
gene4561	53872	NC_000068.7	Caprin1	9.11E+00
gene41943	110355	NC_000085.6	Adrbk1	9.10E+00
gene2065	11931	NC_000067.6	Atp1b1	9.10E+00
gene5746	78928	NC_000068.7	Pigt	9.09E+00
gene19986	12577	NC_000073.6	Cdkn1c	9.09E+00
gene30294	1E+08	NC_000078.6	Ly6e-ps1	9.07E+00
gene8934	68917	NC_000070.6	Hint2	9.05E+00
gene929	13346	NC_000067.6	Des	9.05E+00
gene10299	20970	NC_000070.6	Sdc3	9.03E+00
gene21611	68119	NC_000074.6	Cmtm3	9.03E+00
gene8003	69895	NC_000069.6	Snhg8	9.02E+00

gene36990	67760	NC_000081.6	Slc38a2	9.02E+00
gene2173	14130	NC_000067.6	Fcgr2b	9.02E+00
gene11533	69815	NC_000071.6	Krtcap3	9.01E+00
gene36525	55944	NC_000081.6	Eif3d	9.01E+00
gene4763	433464	NC_000068.7	Gm13991	9.00E+00
gene41982	68090	NC_000085.6	Yif1a	8.99E+00
gene21335	56495	NC_000074.6	Asna1	8.99E+00
gene36449	106025	NC_000081.6	Sharpin	8.98E+00
gene42007	240514	NC_000085.6	Ccdc85b	8.98E+00
gene17912	68845	NC_000073.6	Pih1d1	8.97E+00
gene16764	1E+08	NC_000073.6	Gm24576	8.96E+00
gene16313	243819	NC_000073.6	Ppp6r1	8.96E+00
gene10474	67025	NC_000070.6	Rpl11	8.95E+00
gene11756	66999	NC_000071.6	Med28	8.95E+00
gene1239	67446	NC_000067.6	Dusp28	8.95E+00
gene42241	68539	NC_000085.6	Tmem109	8.94E+00
gene15660	56620	NC_000072.6	Clec4n	8.94E+00
gene33851	108671	NC_000080.6	Dnajc9	8.94E+00
gene9537	69136	NC_000070.6	Tusc1	8.94E+00
gene28045	192159	NC_000077.6	Prpf8	8.93E+00
gene26609	21452	NC_000077.6	Tcn2	8.92E+00
gene28168	16859	NC_000077.6	Lgals9	8.92E+00
gene31071	70369	NC_000078.6	Bag5	8.92E+00
gene2675	320400	NC_000067.6	Gm16897	8.91E+00
gene5831	98999	NC_000068.7	Znfx1	8.90E+00
gene1265	18000	NC_000067.6	sep-02	8.89E+00
gene29370	21681	NC_000077.6	Alyref	8.89E+00
gene41459	667597	NC_000084.6	BC023105	8.89E+00
gene41684	74322	NC_000084.6	Cxxc1	8.89E+00
gene12220	67111	NC_000071.6	Naaa	8.88E+00
gene3417	68365	NC_000068.7	Rab14	8.88E+00
gene16793	232910	NC_000073.6	Ap2s1	8.87E+00
gene8930	1E+08	NC_000070.6	Msmp	8.87E+00
gene20581	68192	NC_000074.6	Leprotl1	8.86E+00
gene22976	12500	NC_000075.6	Cd3d	8.86E+00
gene22272	665108	NC_000075.6	Gm7495	8.86E+00
gene22475	68682	NC_000075.6	Slc44a2	8.86E+00
gene22500	19340	NC_000075.6	Rab3d	8.85E+00
gene43156	12982	NC_000085.6	Csf2ra	8.85E+00
gene26859	17449	NC_000077.6	Mdh1	8.85E+00
gene146	545306	NC_000067.6	Gm5828	8.84E+00
gene8956	12757	NC_000070.6	Clta	8.84E+00
gene922	20872	NC_000067.6	Stk16	8.84E+00
gene28461	18103	NC_000077.6	Nme2	8.84E+00
gene40524	20463	NC_000083.6	Cox7a2l	8.84E+00
gene33289	544973	NC_000079.6	Gm10257	8.83E+00
gene21030	72093	NC_000074.6	2010320M18Rik	8.83E+00
gene41989	319322	NC_000085.6	Sf3b2	8.82E+00

gene11150	109552	NC_000071.6	Sri	8.82E+00
gene28400	68097	NC_000077.6	Dynll2	8.81E+00
gene19826	93747	NC_000073.6	Echs1	8.80E+00
gene3352	13805	NC_000068.7	Eng	8.80E+00
gene8143	66105	NC_000069.6	Ube2d3	8.80E+00
gene14655	13197	NC_000072.6	Gadd45a	8.79E+00
gene19592	269999	NC_000073.6	Orai3	8.77E+00
gene40017	545208	NC_000083.6	Gm5814	8.77E+00
gene21058	13864	NC_000074.6	Nr2f6	8.76E+00
gene10467	71665	NC_000070.6	Fuca1	8.75E+00
gene28819	56208	NC_000077.6	Becn1	8.74E+00
gene29781	22169	NC_000078.6	Cmpk2	8.74E+00
gene39535	13163	NC_000083.6	Daxx	8.74E+00
gene1231	66915	NC_000067.6	Myeov2	8.74E+00
gene11325	671222	NC_000071.6	Gm9523	8.72E+00
gene5741	20971	NC_000068.7	Sdc4	8.72E+00
gene35243	18826	NC_000080.6	Lcp1	8.71E+00
gene27252	59013	NC_000077.6	Hnrnph1	8.71E+00
gene8918	12517	NC_000070.6	Cd72	8.71E+00
gene25578	216152	NC_000076.6	Plppr3	8.71E+00
gene7802	624701	NC_000069.6	Gm12497	8.71E+00
gene30786	320351	NC_000078.6	Tmem251	8.70E+00
gene25622	1E+08	NC_000076.6	Gm30823	8.70E+00
gene39214	26373	NC_000083.6	Clcn7	8.70E+00
gene6274	16592	NC_000069.6	Fabp5	8.70E+00
gene7112	12479	NC_000069.6	Cd1d1	8.70E+00
gene22510	19089	NC_000075.6	Prkcsh	8.69E+00
gene18354	233391	NC_000073.6	Kansl2-ps	8.69E+00
gene10175	245877	NC_000070.6	Map7d1	8.68E+00
gene32921	16372	NC_000079.6	Irx2	8.68E+00
gene8045	211550	NC_000069.6	Tifa	8.67E+00
gene33763	66413	NC_000080.6	Psmd6	8.66E+00
gene10271	19244	NC_000070.6	Ptp4a2	8.66E+00
gene34752	58248	NC_000080.6	1700123O20Rik	8.65E+00
gene15405	677205	NC_000072.6	Gm16433	8.65E+00
gene5301	1E+08	NC_000068.7	Gm14117	8.65E+00
gene35074	66854	NC_000080.6	Trim35	8.64E+00
gene21604	12562	NC_000074.6	Cdh5	8.64E+00
gene13516	66537	NC_000071.6	Pomp	8.63E+00
gene39594	55979	NC_000083.6	Agpat1	8.62E+00
gene37815	224093	NC_000082.6	Fam43a	8.62E+00
gene28676	74026	NC_000077.6	Msl1	8.62E+00
gene25782	216197	NC_000076.6	Ckap4	8.61E+00
gene39619	110147	NC_000083.6	Ehmt2	8.61E+00
gene28469	22057	NC_000077.6	Tob1	8.61E+00
gene1721	226442	NC_000067.6	Zfp281	8.60E+00
gene21847	85305	NC_000074.6	Kars	8.59E+00
gene41497	93687	NC_000084.6	Csnk1a1	8.59E+00

gene7700	102926	NC_000069.6	Atg4a-ps	8.58E+00
gene28835	217203	NC_000077.6	Tmem106a	8.57E+00
gene34821	654795	NC_000080.6	Sdr39u1	8.57E+00
gene25472	20610	NC_000076.6	Sumo3	8.57E+00
gene7561	60365	NC_000069.6	Rbm8a	8.55E+00
gene22483	17083	NC_000075.6	Tmed1	8.55E+00
gene1472	170706	NC_000067.6	Tmem37	8.55E+00
gene21066	66498	NC_000074.6	Dda1	8.54E+00
gene3105	30839	NC_000068.7	Fbxw5	8.54E+00
gene39916	27226	NC_000083.6	Pla2g7	8.54E+00
gene38140	627543	NC_000082.6	Gm6767	8.54E+00
gene20946	11966	NC_000074.6	Atp6v1b2	8.54E+00
gene22486	74766	NC_000075.6	Yipf2	8.53E+00
gene28278	1E+08	NC_000077.6	Gm32650	8.53E+00
gene13061	66258	NC_000071.6	Mrps17	8.53E+00
gene32986	93692	NC_000079.6	Glrx	8.53E+00
gene42028	56390	NC_000085.6	Sssca1	8.53E+00
gene42572	74411	NC_000085.6	Plpp6	8.53E+00
gene13250	666429	NC_000071.6	Gm8099	8.52E+00
gene25812	28088	NC_000076.6	Rtcb	8.51E+00
gene10170	116748	NC_000070.6	Lsm10	8.51E+00
gene2506	15284	NC_000067.6	Hlx	8.51E+00
gene14243	1E+08	NC_000072.6	Gm18584	8.50E+00
gene27814	216858	NC_000077.6	Kctd11	8.50E+00
gene37399	58239	NC_000082.6	Dexi	8.50E+00
gene42271	69774	NC_000085.6	Ms4a6b	8.49E+00
gene12450	1E+08	NC_000071.6	Gm31785	8.48E+00
gene27205	17308	NC_000077.6	Mgat1	8.48E+00
gene10792	1E+08	NC_000070.6	Gm13142	8.47E+00
gene3346	13481	NC_000068.7	Dpm2	8.47E+00
gene40481	225027	NC_000083.6	Srsf7	8.46E+00
gene4839	66602	NC_000068.7	1700020I14Rik	8.46E+00
gene37699	545152	NC_000082.6	Gm5809	8.46E+00
gene25687	15353	NC_000076.6	Hmg20b	8.46E+00
gene26826	19324	NC_000077.6	Rab1a	8.45E+00
gene27752	22318	NC_000077.6	Vamp2	8.45E+00
gene2231	16456	NC_000067.6	F11r	8.44E+00
gene15967	381820	NC_000072.6	Smim10l1	8.44E+00
gene10991	67830	NC_000070.6	Rer1	8.44E+00
gene23653	235509	NC_000075.6	Gm4896	8.43E+00
gene37217	16421	NC_000081.6	Itgb7	8.42E+00
gene19159	626661	NC_000073.6	Gm10156	8.41E+00
gene27644	18858	NC_000077.6	Pmp22	8.41E+00
gene35413	1E+08	NC_000080.6	Rpl36a-ps1	8.39E+00
gene12452	100702	NC_000071.6	Gbp6	8.39E+00
gene27928	18432	NC_000077.6	Mybbp1a	8.39E+00
gene10671	14726	NC_000070.6	Pdpn	8.38E+00
gene4800	1E+08	NC_000068.7	Inafm2	8.38E+00

gene26683	56418	NC_000077.6	Ykt6	8.38E+00
gene35162	213019	NC_000080.6	Pdlim2	8.36E+00
gene35669	666167	NC_000081.6	Gm7962	8.35E+00
gene20414	1E+08	NC_000074.6	Gm10043	8.34E+00
gene10351	19204	NC_000070.6	Ptafr	8.34E+00
gene42113	107260	NC_000085.6	Otub1	8.34E+00
gene19917	1E+08	NC_000073.6	Gm10575	8.34E+00
gene39015	51792	NC_000083.6	Ppp2r1a	8.34E+00
gene33710	666976	NC_000080.6	Gm8396	8.34E+00
gene9819	ene=LOC1	NC_000070.6	LOC102636060	8.33E+00
gene3363	30933	NC_000068.7	Tor2a	8.33E+00
gene24658	15979	NC_000076.6	Ifngr1	8.33E+00
gene42009	13041	NC_000085.6	Ctsw	8.33E+00
gene19603	76560	NC_000073.6	Prss8	8.33E+00
gene23720	212943	NC_000075.6	Fam46a	8.32E+00
gene7691	229663	NC_000069.6	Csde1	8.32E+00
gene2362	15365	NC_000067.6	Hmga2-ps1	8.29E+00
gene30312	238257	NC_000078.6	Tmem30b	8.29E+00
gene24577	67844	NC_000076.6	Rab32	8.28E+00
gene42077	22668	NC_000085.6	Sf1	8.28E+00
gene31082	70257	NC_000078.6	2010107E04Rik	8.28E+00
gene915	69171	NC_000067.6	Cnppd1	8.27E+00
gene21477	104158	NC_000074.6	Ces1d	8.27E+00
gene13242	231803	NC_000071.6	Mepce	8.27E+00
gene28771	16480	NC_000077.6	Jup	8.27E+00
gene15431	434077	NC_000072.6	Gm5578	8.27E+00
gene41037	15526	NC_000084.6	Hspa9	8.27E+00
gene1945	433368	NC_000067.6	Gm15428	8.26E+00
gene23001	214597	NC_000075.6	Sidt2	8.26E+00
gene12681	66383	NC_000071.6	Iscu	8.26E+00
gene13435	231889	NC_000071.6	Bud31	8.26E+00
gene26823	66713	NC_000077.6	Actr2	8.25E+00
gene12739	50849	NC_000071.6	Rnf10	8.25E+00
gene32933	210992	NC_000079.6	Lpcat1	8.24E+00
gene29382	20892	NC_000077.6	Stra13	8.23E+00
gene18876	20821	NC_000073.6	Trim21	8.23E+00
gene36324	93696	NC_000081.6	Chrac1	8.23E+00
gene11531	192292	NC_000071.6	Nrbp1	8.22E+00
gene24650	21929	NC_000076.6	Tnfaip3	8.22E+00
gene29361	56282	NC_000077.6	Mrpl12	8.22E+00
gene16532	628596	NC_000073.6	Gm6900	8.21E+00
gene26367	216440	NC_000076.6	Os9	8.21E+00
gene19561	16408	NC_000073.6	Itgal	8.21E+00
gene4212	668447	NC_000068.7	Gm13743	8.20E+00
gene21019	50783	NC_000074.6	Lsm4	8.20E+00
gene26964	1E+08	NC_000077.6	Gm12096	8.19E+00
gene3919	18024	NC_000068.7	Nfe2l2	8.18E+00
gene17266	19153	NC_000073.6	Prx	8.18E+00

gene40169	14154	NC_000083.6	Fem1a	8.16E+00
gene27775	67020	NC_000077.6	Tmem88	8.16E+00
gene41951	19671	NC_000085.6	Rce1	8.16E+00
gene29499	668388	NC_000078.6	Gm9144	8.16E+00
gene39699	14251	NC_000083.6	Flot1	8.15E+00
gene33458	14938	NC_000079.6	Gzma	8.15E+00
gene37372	52502	NC_000082.6	Carhsp1	8.14E+00
gene17207	21803	NC_000073.6	Tgfb1	8.14E+00
gene40004	66229	NC_000083.6	Rpl7l1	8.13E+00
gene2627	22782	NC_000067.6	Slc30a1	8.13E+00
gene37982	12856	NC_000082.6	Cox17	8.12E+00
gene22233	664889	NC_000075.6	Gm7390	8.12E+00
gene32181	105245	NC_000079.6	Txndc5	8.10E+00
gene28157	55978	NC_000077.6	Ift20	8.10E+00
gene601	15528	NC_000067.6	Hspe1	8.10E+00
gene5843	81018	NC_000068.7	Rnf114	8.09E+00
gene14584	68140	NC_000072.6	Tigd2	8.09E+00
gene32172	107513	NC_000079.6	Ssr1	8.09E+00
gene13943	27205	NC_000072.6	Podxl	8.09E+00
gene10536	66259	NC_000070.6	Camk2n1	8.08E+00
gene25450	12833	NC_000076.6	Col6a1	8.07E+00
gene4602	19672	NC_000068.7	Rcn1	8.07E+00
gene33352	631097	NC_000079.6	Gm7054	8.07E+00
gene41764	433204	NC_000084.6	Gm5509	8.05E+00
gene12900	654470	NC_000071.6	Tctn1	8.04E+00
gene34453	633238	NC_000080.6	Gm7107	8.04E+00
gene36082	665041	NC_000081.6	Gm7459	8.04E+00
gene11512	50907	NC_000071.6	Preb	8.04E+00
gene43043	433256	NC_000085.6	Acsl5	8.04E+00
gene9910	22275	NC_000070.6	Urod	8.03E+00
gene28850	72349	NC_000077.6	Dusp3	8.03E+00
gene17877	67605	NC_000073.6	Akt1s1	8.03E+00
gene36731	74778	NC_000081.6	Rrp7a	8.02E+00
gene38206	224250	NC_000082.6	Cldnd1	8.01E+00
gene24353	113868	NC_000075.6	Acaa1a	8.00E+00
gene42268	73656	NC_000085.6	Ms4a6c	7.99E+00
gene5878	69372	NC_000068.7	Mocs3	7.97E+00
gene38395	11504	NC_000082.6	Adamts1	7.96E+00
gene41548	629436	NC_000084.6	Gm6974	7.96E+00
gene29287	21858	NC_000077.6	Timp2	7.96E+00
gene7176	20351	NC_000069.6	Sema4a	7.96E+00
gene27020	432548	NC_000077.6	Rpsa-ps4	7.96E+00
gene27889	12261	NC_000077.6	C1qbp	7.95E+00
gene4593	668744	NC_000068.7	Gm13886	7.94E+00
gene35951	12864	NC_000081.6	Cox6c	7.94E+00
gene15834	545878	NC_000072.6	Gm5884	7.93E+00
gene28626	16796	NC_000077.6	Lasp1	7.93E+00
gene18274	109815	NC_000073.6	Vimp	7.92E+00

gene10285	94242	NC_000070.6	Tinagl1	7.92E+00
gene3059	107733	NC_000068.7	Mrpl41	7.91E+00
gene21060	68251	NC_000074.6	Babam1	7.91E+00
gene29204	14635	NC_000077.6	Galk1	7.91E+00
gene30436	20384	NC_000078.6	Srsf5	7.91E+00
gene2223	27045	NC_000067.6	Nit1	7.90E+00
gene10202	80284	NC_000070.6	Smim12	7.89E+00
gene30235	1E+08	NC_000078.6	Gm3086	7.89E+00
gene28125	1E+08	NC_000077.6	Gm12571	7.89E+00
gene27792	13681	NC_000077.6	Eif4a1	7.88E+00
gene43069	11554	NC_000085.6	Adrb1	7.86E+00
gene37716	67838	NC_000082.6	Dnajb11	7.86E+00
gene32624	13025	NC_000079.6	Ctla2b	7.85E+00
gene36556	104445	NC_000081.6	Cdc42ep1	7.85E+00
gene8907	66592	NC_000070.6	Stoml2	7.85E+00
gene16912	11812	NC_000073.6	Apoc1	7.85E+00
gene15733	107932	NC_000072.6	Chd4	7.85E+00
gene21544	17388	NC_000074.6	Mmp15	7.85E+00
gene37337	207740	NC_000082.6	Ubald1	7.84E+00
gene28541	268470	NC_000077.6	Ube2z	7.84E+00
gene22001	1E+08	NC_000074.6	Gm17709	7.83E+00
gene6815	74191	NC_000069.6	P2ry13	7.83E+00
gene34760	12050	NC_000080.6	Bcl2l2	7.83E+00
gene3276	72500	NC_000068.7	Ier5l	7.80E+00
gene5425	76650	NC_000068.7	Srxn1	7.80E+00
gene13327	27979	NC_000071.6	Eif3b	7.80E+00
gene39913	224792	NC_000083.6	Adgrf5	7.79E+00
gene12899	74096	NC_000071.6	Hvcn1	7.78E+00
gene11444	231070	NC_000071.6	Insig1	7.77E+00
gene41353	16948	NC_000084.6	Lox	7.77E+00
gene17469	1.1E+08	NC_000073.6	Gm38984	7.76E+00
gene31554	385049	NC_000079.6	Gm5374	7.75E+00
gene24600	66848	NC_000076.6	Fuca2	7.75E+00
gene27022	18148	NC_000077.6	Npm1	7.75E+00
gene15551	74471	NC_000072.6	4933440N22Rik	7.75E+00
gene21014	76900	NC_000074.6	Ssbp4	7.74E+00
gene22606	11804	NC_000075.6	Aplp2	7.74E+00
gene1570	17164	NC_000067.6	Mapkapk2	7.74E+00
gene42725	668251	NC_000085.6	Gm9067	7.74E+00
gene40165	28106	NC_000083.6	Mydgf	7.73E+00
gene2252	19298	NC_000067.6	Pex19	7.73E+00
gene39993	27398	NC_000083.6	Mrpl2	7.73E+00
gene13734	17992	NC_000072.6	Ndufa4	7.73E+00
gene11305	625004	NC_000071.6	Gm6543	7.72E+00
gene12159	353327	NC_000071.6	Mkrn1-ps1	7.72E+00
gene26460	18813	NC_000076.6	Pa2g4	7.72E+00
gene25608	75406	NC_000076.6	Ndufs7	7.71E+00
gene13689	545826	NC_000072.6	Gm5873	7.71E+00

gene42274	68774	NC_000085.6	Ms4a6d	7.71E+00
gene7840	66921	NC_000069.6	Prpf38b	7.70E+00
gene13200	18787	NC_000071.6	Serpine1	7.70E+00
gene21127	1E+08	NC_000074.6	Gm18646	7.68E+00
gene20195	14058	NC_000074.6	F10	7.67E+00
gene11002	14688	NC_000070.6	Gnb1	7.67E+00
gene20986	75692	NC_000074.6	Nr2c2ap	7.67E+00
gene19551	68449	NC_000073.6	Tbc1d10b	7.67E+00
gene24072	12700	NC_000075.6	Cish	7.67E+00
gene15465	22346	NC_000072.6	Vhl	7.67E+00
gene22311	66070	NC_000075.6	Cwc15	7.66E+00
gene25638	103236	NC_000076.6	Csnk1g2	7.65E+00
gene9260	17025	NC_000070.6	Alad	7.65E+00
gene25602	59090	NC_000076.6	Midn	7.65E+00
gene22472	12581	NC_000075.6	Cdkn2d	7.65E+00
gene39723	547347	NC_000083.6	Gm6034	7.64E+00
gene23492	12175	NC_000075.6	Bnip2	7.64E+00
gene15500	352968	NC_000072.6	D830050J10Rik	7.64E+00
gene13162	22628	NC_000071.6	Ywhag	7.64E+00
gene41520	11555	NC_000084.6	Adrb2	7.63E+00
gene23780	12045	NC_000075.6	Bcl2a1b	7.62E+00
gene29210	217333	NC_000077.6	Trim47	7.62E+00
gene28980	13056	NC_000077.6	Cyb561	7.61E+00
gene884	69660	NC_000067.6	Tmbim1	7.61E+00
gene24811	1E+08	NC_000076.6	Gm10145	7.61E+00
gene23213	66199	NC_000075.6	Commd4	7.61E+00
gene39662	53817	NC_000083.6	Ddx39b	7.61E+00
gene7413	78523	NC_000069.6	Mrpl9	7.60E+00
gene4998	69470	NC_000068.7	Tmem127	7.60E+00
gene38542	28080	NC_000082.6	Atp5o	7.59E+00
gene35314	629709	NC_000080.6	Gm6997	7.59E+00
gene4001	433550	NC_000068.7	Gm13675	7.59E+00
gene9002	624430	NC_000070.6	Gm12444	7.59E+00
gene8287	12042	NC_000069.6	Bcl10	7.58E+00
gene13128	12740	NC_000071.6	Cldn4	7.58E+00
gene37115	14555	NC_000081.6	Gpd1	7.56E+00
gene17401	66411	NC_000073.6	Tbcb	7.56E+00
gene37104	1E+08	NC_000081.6	Gm34765	7.56E+00
gene38705	66467	NC_000083.6	Gtf2h5	7.56E+00
gene12081	1E+08	NC_000071.6	Hmgn2-ps1	7.55E+00
gene42948	93679	NC_000085.6	Trim8	7.55E+00
gene13271	66096	NC_000071.6	Lamtor4	7.55E+00
gene39186	71893	NC_000083.6	Noxo1	7.54E+00
gene13167	100647	NC_000071.6	Upk3b	7.54E+00
gene5431	24105	NC_000068.7	Rbck1	7.54E+00
gene5598	98932	NC_000068.7	Myl9	7.54E+00
gene38517	15080	NC_000082.6	H3f3a-ps2	7.53E+00
gene19482	16190	NC_000073.6	Il4ra	7.53E+00

gene37956	15163	NC_000082.6	Hcls1	7.53E+00
gene32330	68128	NC_000079.6	Fam120aos	7.52E+00
gene20056	20911	NC_000074.6	Stxbp2	7.52E+00
gene20062	66682	NC_000074.6	Trappc5	7.52E+00
gene36437	671535	NC_000081.6	Parp10	7.52E+00
gene14955	434050	NC_000072.6	Gm5576	7.51E+00
gene25745	544716	NC_000076.6	Ap3m1-ps	7.51E+00
gene34043	218877	NC_000080.6	Sema3g	7.50E+00
gene42163	LOC10264	NC_000085.6	LOC102640359	7.50E+00
gene16632	1E+08	NC_000073.6	Gm15773	7.49E+00
gene23719	666672	NC_000075.6	Gm8228	7.49E+00
gene15013	70020	NC_000072.6	Ino80b	7.48E+00
gene29592	1E+08	NC_000078.6	Gm9202	7.48E+00
gene6227	667759	NC_000069.6	Gm8797	7.47E+00
gene13199	11769	NC_000071.6	Ap1s1	7.47E+00
gene13892	27056	NC_000072.6	Irf5	7.47E+00
gene21468	17390	NC_000074.6	Mmp2	7.46E+00
gene37418	20621	NC_000082.6	Snn	7.46E+00
gene43142	13669	NC_000085.6	Eif3a	7.45E+00
gene26380	69654	NC_000076.6	Dctn2	7.45E+00
gene11374	100910	NC_000071.6	Chpf2	7.45E+00
gene30175	623123	NC_000078.6	Gm6395	7.44E+00
gene16056	20901	NC_000072.6	Strap	7.44E+00
gene19254	11535	NC_000073.6	Adm	7.44E+00
gene17317	54624	NC_000073.6	Paf1	7.43E+00
gene22404	665751	NC_000075.6	Gm7769	7.42E+00
gene19540	78388	NC_000073.6	Mvp	7.42E+00
gene7857	1E+08	NC_000069.6	Gm5548	7.42E+00
gene35788	20024	NC_000081.6	Sub1	7.41E+00
gene11610	68294	NC_000071.6	Mfsd10	7.41E+00
gene2612	19327	NC_000067.6	Rab11b-ps1	7.41E+00
gene42941	67116	NC_000085.6	Cuedc2	7.41E+00
gene21414	71607	NC_000074.6	Snx20	7.41E+00
gene28805	21428	NC_000077.6	Mlx	7.41E+00
gene1987	12301	NC_000067.6	Cacybp	7.40E+00
gene3332	72931	NC_000068.7	Swi5	7.40E+00
gene28664	1E+08	NC_000077.6	Gm12355	7.39E+00
gene39989	20807	NC_000083.6	Srf	7.38E+00
gene27544	268420	NC_000077.6	Alkbh5	7.38E+00
gene23228	12858	NC_000075.6	Cox5a	7.38E+00
gene9555	1E+08	NC_000070.6	Gm12693	7.38E+00
gene19510	171504	NC_000073.6	Apobr	7.38E+00
gene23003	1E+08	NC_000075.6	A830035O19Rik	7.38E+00
gene25120	14609	NC_000076.6	Gja1	7.37E+00
gene34002	80795	NC_000080.6	Selk	7.37E+00
gene22975	12502	NC_000075.6	Cd3g	7.37E+00
gene11695	22388	NC_000071.6	Wdr1	7.36E+00
gene36358	68311	NC_000081.6	Lypd2	7.36E+00

gene42024	26403	NC_000085.6	Map3k11	7.36E+00
gene25368	216082	NC_000076.6	Gm4798	7.35E+00
gene39657	21926	NC_000083.6	Tnf	7.35E+00
gene8954	384009	NC_000070.6	Glipr2	7.35E+00
gene14749	545848	NC_000072.6	Igkv4-80	7.33E+00
gene38532	77975	NC_000082.6	Tmem50b	7.33E+00
gene1683	68724	NC_000067.6	Arl8a	7.32E+00
gene20073	664888	NC_000074.6	Gm7389	7.30E+00
gene23117	19684	NC_000075.6	Rdx	7.30E+00
gene42288	622845	NC_000085.6	Gm6365	7.30E+00
gene16859	21946	NC_000073.6	Pglyrp1	7.29E+00
gene26958	20742	NC_000077.6	Sptbn1	7.29E+00
gene27876	216873	NC_000077.6	Spag7	7.28E+00
gene15533	213393	NC_000072.6	8430408G22Rik	7.28E+00
gene34016	21881	NC_000080.6	Tkt	7.28E+00
gene38615	23872	NC_000082.6	Ets2	7.27E+00
gene26601	544752	NC_000077.6	Tug1	7.27E+00
gene37581	19385	NC_000082.6	Ranbp1	7.27E+00
gene17334	24030	NC_000073.6	Mrps12	7.27E+00
gene28596	16211	NC_000077.6	Kpnb1	7.26E+00
gene3149	66865	NC_000068.7	Pmpca	7.25E+00
gene9509	12579	NC_000070.6	Cdkn2b	7.25E+00
gene17246	112406	NC_000073.6	Egln2	7.25E+00
gene19916	213573	NC_000073.6	Cracr2b	7.25E+00
gene22928	213211	NC_000075.6	Rnf26	7.24E+00
gene41053	67869	NC_000084.6	Paip2	7.24E+00
gene40417	67864	NC_000083.6	Yipf4	7.24E+00
gene19363	56209	NC_000073.6	Gde1	7.23E+00
gene7766	12508	NC_000069.6	Cd53	7.22E+00
gene40299	65960	NC_000083.6	Twsg1	7.22E+00
gene5605	1E+08	NC_000068.7	Gm14248	7.22E+00
gene42008	58249	NC_000085.6	Fibp	7.22E+00
gene12420	13602	NC_000071.6	Sparcl1	7.21E+00
gene28829	70110	NC_000077.6	Ifi35	7.21E+00
gene29315	14387	NC_000077.6	Gaa	7.21E+00
gene25563	216150	NC_000076.6	Cdc34	7.20E+00
gene26397	20852	NC_000076.6	Stat6	7.20E+00
gene15828	667598	NC_000072.6	Gm8719	7.19E+00
gene29178	udogene;g	NC_000077.6	LOC102634812	7.19E+00
gene8263	54673	NC_000069.6	Sh3glb1	7.17E+00
gene42222	278795	NC_000085.6	Lrrc10b	7.17E+00
gene28866	68401	NC_000077.6	G6pc3	7.17E+00
gene3090	83768	NC_000068.7	Dpp7	7.16E+00
gene24457	21922	NC_000075.6	Clec3b	7.16E+00
gene26354	52468	NC_000076.6	Ctdsp2	7.16E+00
gene24564	1.1E+08	NC_000076.6	Gm40600	7.15E+00
gene23073	76509	NC_000075.6	Plet1	7.14E+00
gene11561	19046	NC_000071.6	Ppp1cb	7.13E+00

gene1687	13710	NC_000067.6	Elf3	7.13E+00
gene8819	76959	NC_000070.6	Chmp5	7.13E+00
gene21276	666704	NC_000074.6	Samd1	7.13E+00
gene40736	665176	NC_000084.6	Gm7527	7.12E+00
gene11674	231162	NC_000071.6	Cytl1	7.11E+00
gene10936	57295	NC_000070.6	Icmt	7.11E+00
gene18467	29875	NC_000073.6	Iqgap1	7.10E+00
gene41604	67951	NC_000084.6	Tubb6	7.10E+00
gene34117	1E+08	NC_000080.6	Gm3219	7.10E+00
gene11575	13016	NC_000071.6	Ctbp1	7.10E+00
gene10902	57320	NC_000070.6	Park7	7.10E+00
gene9444	11520	NC_000070.6	Plin2	7.09E+00
gene18440	16790	NC_000073.6	Anpep	7.09E+00
gene32409	1E+08	NC_000079.6	Gm29787	7.08E+00
gene36475	76282	NC_000081.6	Gpt	7.08E+00
gene4040	58207	NC_000068.7	Slc43a3	7.08E+00
gene32565	75731	NC_000079.6	Idnk	7.08E+00
gene8921	taining 107	NC_000070.6	Ccdc107	7.07E+00
gene6349	545508	NC_000069.6	Gm5844	7.07E+00
gene27789	24070	NC_000077.6	Mpdu1	7.07E+00
gene9789	29864	NC_000070.6	Rnf11	7.05E+00
gene29803	104923	NC_000078.6	Adi1	7.05E+00
gene7236	20416	NC_000069.6	Shc1	7.05E+00
gene16351	13854	NC_000073.6	Epn1	7.03E+00
gene42085	56613	NC_000085.6	Rps6ka4	7.03E+00
gene8085	66357	NC_000069.6	Ostc	7.03E+00
gene21357	56445	NC_000074.6	Dnaja2	7.02E+00
gene28784	71966	NC_000077.6	Nkiras2	7.02E+00
gene40131	22060	NC_000083.6	Trp53-ps	7.02E+00
gene36889	11883	NC_000081.6	Arsa	7.01E+00
gene2211	641376	NC_000067.6	Tomm40l	7.00E+00
gene4048	23796	NC_000068.7	Aplnr	7.00E+00
gene25684	70312	NC_000076.6	Cactin	7.00E+00
gene20150	12826	NC_000074.6	Col4a1	6.99E+00
gene27800	75580	NC_000077.6	Zbtb4	6.99E+00
gene24731	21412	NC_000076.6	Tcf21	6.99E+00
gene31576	75398	NC_000079.6	Mrpl32	6.99E+00
gene42562	1E+08	NC_000085.6	Gm35781	6.99E+00
gene76	ene=LOC1	NC_000067.6	LOC108167613	6.98E+00
gene28935	104582	NC_000077.6	Rprml	6.98E+00
gene40400	22436	NC_000083.6	Xdh	6.98E+00
gene36603	223697	NC_000081.6	Sun2	6.98E+00
gene28782	12799	NC_000077.6	Cnp	6.97E+00
gene928	13437	NC_000067.6	Dnpep	6.97E+00
gene3254	56086	NC_000068.7	Set	6.97E+00
gene17159	20085	NC_000073.6	Rps19	6.97E+00
gene23649	666456	NC_000075.6	Gm8116	6.96E+00
gene42521	226040	NC_000085.6	Tmem252	6.96E+00

gene18220	17984	NC_000073.6	Ndn	6.96E+00
gene40387	77889	NC_000083.6	Lbh	6.96E+00
gene7513	14129	NC_000069.6	Fcgr1	6.95E+00
gene38824	67681	NC_000083.6	Mrpl18	6.95E+00
gene39386	20383	NC_000083.6	Srsf3	6.95E+00
gene11366	66587	NC_000071.6	Fastk	6.95E+00
gene19495	16797	NC_000073.6	Lat	6.94E+00
gene39557	20182	NC_000083.6	Rxrb	6.94E+00
gene19112	668139	NC_000073.6	Gm8995	6.94E+00
gene21685	497652	NC_000074.6	Acd	6.94E+00
gene21570	333331	NC_000074.6	Gm5131	6.94E+00
gene35930	1E+08	NC_000081.6	Gm18949	6.93E+00
gene20941	16956	NC_000074.6	Lpl	6.93E+00
gene27495	14923	NC_000077.6	Guk1	6.93E+00
gene12733	106840	NC_000071.6	Unc119b	6.93E+00
gene12042	67054	NC_000071.6	Paics	6.92E+00
gene5526	75608	NC_000068.7	Chmp4b	6.91E+00
gene42668	16889	NC_000085.6	Lipa	6.91E+00
gene4786	21825	NC_000068.7	Thbs1	6.91E+00
gene17952	1E+08	NC_000073.6	A030001D20Rik	6.91E+00
gene23382	330959	NC_000075.6	Snapc5	6.90E+00
gene23900	50797	NC_000075.6	Copb2	6.90E+00
gene22766	67776	NC_000075.6	Vwa5a	6.90E+00
gene12904	56433	NC_000071.6	Vps29	6.90E+00
gene29170	71679	NC_000077.6	Atp5h	6.90E+00
gene17940	56734	NC_000073.6	Tulp2	6.90E+00
gene33693	432817	NC_000080.6	Gm5456	6.89E+00
gene26437	20847	NC_000076.6	Stat2	6.89E+00
gene19457	73951	NC_000073.6	4930413G21Rik	6.89E+00
gene18451	83485	NC_000073.6	Ngrn	6.88E+00
gene32958	66945	NC_000079.6	Sdha	6.88E+00
gene23792	621414	NC_000075.6	Gm6223	6.88E+00
gene25506	18641	NC_000076.6	Pfkl	6.88E+00
gene18883	1E+08	NC_000073.6	Gm2996	6.87E+00
gene36463	30840	NC_000081.6	Fbxl6	6.86E+00
gene3368	227737	NC_000068.7	Fam129b	6.86E+00
gene6582	381438	NC_000069.6	Gm5148	6.86E+00
gene109	72265	NC_000067.6	Tram1	6.86E+00
gene39714	224742	NC_000083.6	Abcf1	6.86E+00
gene29626	18263	NC_000078.6	Odc1	6.86E+00
gene41093	24010	NC_000084.6	Ik	6.84E+00
gene582	70396	NC_000067.6	Asnsd1	6.84E+00
gene42482	22682	NC_000085.6	Zfand5	6.84E+00
gene17181	18476	NC_000073.6	Pafah1b3	6.83E+00
gene24137	67789	NC_000075.6	Dalrd3	6.82E+00
gene16900	232946	NC_000073.6	Bloc1s3	6.82E+00
gene27395	11927	NC_000077.6	Atox1	6.82E+00
gene17651	72244	NC_000073.6	1600014C10Rik	6.82E+00

gene38531	15980	NC_000082.6	Ifngr2	6.82E+00
gene32292	110052	NC_000079.6	Dek	6.81E+00
gene13201	384309	NC_000071.6	Trim56	6.80E+00
gene37398	12265	NC_000082.6	Ciita	6.80E+00
gene15251	74122	NC_000072.6	Tmem43	6.80E+00
gene33758	1E+08	NC_000080.6	Gm18576	6.80E+00
gene15526	434080	NC_000072.6	Gm5580	6.80E+00
gene42021	20469	NC_000085.6	Sipa1	6.79E+00
gene21650	97440	NC_000074.6	B3gnt9	6.79E+00
gene26696	52915	NC_000077.6	Zmiz2	6.79E+00
gene28260	20555	NC_000077.6	Slfn1	6.79E+00
gene19548	20737	NC_000073.6	Spn	6.79E+00
gene6206	67005	NC_000068.7	Polr3k	6.77E+00
gene6221	620283	NC_000069.6	Gm6140	6.77E+00
gene34317	12159	NC_000080.6	Bmp4	6.77E+00
gene34062	105638	NC_000080.6	Dph3	6.77E+00
gene40212	98053	NC_000083.6	Gtf2f1	6.77E+00
gene6199	56470	NC_000068.7	Rgs19	6.76E+00
gene39134	67673	NC_000083.6	Tceb2	6.75E+00
gene17899	14132	NC_000073.6	Fcgrt	6.75E+00
gene25207	13688	NC_000076.6	Eif4ebp2	6.74E+00
gene13865	74375	NC_000072.6	Gcc1	6.74E+00
gene15448	52163	NC_000072.6	Camk1	6.73E+00
gene22062	66855	NC_000074.6	Tcf25	6.72E+00
gene8657	383998	NC_000070.6	Gm11889	6.72E+00
gene13878	23917	NC_000072.6	Impdh1	6.72E+00
gene2048	665237	NC_000067.6	Rpsa-ps1	6.72E+00
gene26399	17937	NC_000076.6	Nab2	6.71E+00
gene10112	54170	NC_000070.6	Rragc	6.71E+00
gene28055	12928	NC_000077.6	Crk	6.71E+00
gene41305	67526	NC_000084.6	Atg12	6.71E+00
gene24034	23999	NC_000075.6	Twf2	6.71E+00
gene42128	225845	NC_000085.6	Pla2g16	6.70E+00
gene13438	57423	NC_000071.6	Atp5j2	6.69E+00
gene26925	626571	NC_000077.6	Gm6685	6.68E+00
gene5778	=Gene;ger	NC_000068.7	LOC108168813	6.68E+00
gene36445	109075	NC_000081.6	Exosc4	6.68E+00
gene361	226977	NC_000067.6	Actr1b	6.68E+00
gene34163	1E+08	NC_000080.6	Eif1-ps1	6.68E+00
gene37561	224024	NC_000082.6	Scarf2	6.67E+00
gene33885	218820	NC_000080.6	Zfp503	6.67E+00
gene16275	18733	NC_000073.6	Pirb	6.67E+00
gene8254	16911	NC_000069.6	Lmo4	6.67E+00
gene33863	73068	NC_000080.6	Fut11	6.67E+00
gene19981	56844	NC_000073.6	Tssc4	6.66E+00
gene39727	14963	NC_000083.6	H2-Bl	6.66E+00
gene32471	74325	NC_000079.6	Cltb	6.66E+00
gene12229	12492	NC_000071.6	Scarb2	6.65E+00

gene14874	57896	NC_000072.6	Krcc1	6.65E+00
gene3356	107951	NC_000068.7	Cdk9	6.65E+00
gene9962	21846	NC_000070.6	Tie1	6.65E+00
gene37756	1E+08	NC_000082.6	Gm4521	6.65E+00
gene39185	11692	NC_000083.6	Gfer	6.65E+00
gene8843	69961	NC_000070.6	Rpp25l	6.65E+00
gene17890	54131	NC_000073.6	Irf3	6.64E+00
gene31770	319158	NC_000079.6	Hist1h4i	6.64E+00
gene28919	1E+08	NC_000077.6	Gm20511	6.64E+00
gene26007	1E+08	NC_000076.6	Gm34921	6.63E+00
gene23042	104444	NC_000075.6	Rexo2	6.63E+00
gene17161	16801	NC_000073.6	Arhgef1	6.63E+00
gene25902	216229	NC_000076.6	Gm4800	6.62E+00
gene41454	240328	NC_000084.6	F830016B08Rik	6.62E+00
gene30304	ene=LOC1	NC_000078.6	LOC102636900	6.62E+00
gene7501	69168	NC_000069.6	Bola1	6.61E+00
gene25882	544719	NC_000076.6	Gm5780	6.61E+00
gene10964	68859	NC_000070.6	Smim1	6.60E+00
gene7695	68183	NC_000069.6	Bcas2	6.60E+00
gene15642	667107	NC_000072.6	Gm8460	6.60E+00
gene8285	1E+08	NC_000069.6	Gm35187	6.59E+00
gene16795	243853	NC_000073.6	Fkrp	6.59E+00
gene28677	1E+08	NC_000077.6	Gm12359	6.59E+00
gene15121	11746	NC_000072.6	Anxa4	6.58E+00
gene14228	546897	NC_000072.6	Gm5990	6.57E+00
gene42269	1E+08	NC_000085.6	BE692007	6.56E+00
gene21662	66320	NC_000074.6	Tmem208	6.56E+00
gene15411	192193	NC_000072.6	Edem1	6.55E+00
gene2978	12151	NC_000068.7	Bmi1	6.55E+00
gene18353	1E+08	NC_000073.6	Rpl17-ps10	6.55E+00
gene3251	20740	NC_000068.7	Sptan1	6.55E+00
gene21155	666036	NC_000074.6	Gm7901	6.55E+00
gene14809	243461	NC_000072.6	Igkv7-33	6.55E+00
gene15726	319352	NC_000072.6	Pianp	6.54E+00
gene22237	12362	NC_000075.6	Casp1	6.54E+00
gene37170	74127	NC_000081.6	Krt80	6.54E+00
gene41059	68545	NC_000084.6	Ecscr	6.53E+00
gene11977	68095	NC_000071.6	Ociad1	6.52E+00
gene36063	432947	NC_000081.6	Gm5470	6.52E+00
gene15721	442825	NC_000072.6	A230083G16Rik	6.51E+00
gene13083	66711	NC_000071.6	Sbds	6.51E+00
gene30398	53612	NC_000078.6	Vti1b	6.51E+00
gene1475	13167	NC_000067.6	Dbi	6.51E+00
gene21109	17274	NC_000074.6	Rab8a	6.50E+00
gene17367	68458	NC_000073.6	Ppp1r14a	6.50E+00
gene33329	328330	NC_000079.6	D130037M23Rik	6.50E+00
gene15150	69834	NC_000072.6	Rab43	6.49E+00
gene10526	15441	NC_000070.6	Hp1bp3	6.49E+00

gene17176	606496	NC_000073.6	Gsk3a	6.49E+00
gene39928	224796	NC_000083.6	Clic5	6.48E+00
gene22450	14739	NC_000075.6	S1pr2	6.48E+00
gene42798	107358	NC_000085.6	Tm9sf3	6.47E+00
gene42673	667370	NC_000085.6	Ifit3b	6.47E+00
gene20726	102294	NC_000074.6	Cyp4v3	6.47E+00
gene22962	213827	NC_000075.6	Arcn1	6.46E+00
gene12245	12452	NC_000071.6	Ccng2	6.46E+00
gene25682	18717	NC_000076.6	Pip5k1c	6.46E+00
gene37231	18521	NC_000081.6	Pcbp2	6.46E+00
gene27840	12029	NC_000077.6	Bcl6b	6.45E+00
gene39302	11974	NC_000083.6	Atp6v0e	6.45E+00
gene20952	665553	NC_000074.6	Gm7684	6.45E+00
gene39316	474156	NC_000083.6	Zbtb9	6.45E+00
gene28050	18738	NC_000077.6	Pitpna	6.45E+00
gene41436	66307	NC_000084.6	Isoc1	6.45E+00
gene41054	20522	NC_000084.6	Slc23a1	6.45E+00
gene6253	15213	NC_000069.6	Hey1	6.44E+00
gene40171	66905	NC_000083.6	Plin3	6.44E+00
gene16271	353499	NC_000073.6	Tmc4	6.44E+00
gene25599	20869	NC_000076.6	Stk11	6.43E+00
gene20024	72284	NC_000073.6	Oraov1	6.43E+00
gene15697	14791	NC_000072.6	Emg1	6.43E+00
gene28590	107732	NC_000077.6	Mrpl10	6.43E+00
gene29832	629656	NC_000078.6	Gm6992	6.42E+00
gene34045	104416	NC_000080.6	Bap1	6.41E+00
gene3005	667609	NC_000068.7	Gm13328	6.41E+00
gene42182	109077	NC_000085.6	Ints5	6.40E+00
gene37559	ene=LOC1	NC_000082.6	LOC102637743	6.40E+00
gene10608	67680	NC_000070.6	Sdhb	6.40E+00
gene33440	16195	NC_000079.6	Il6st	6.40E+00
gene14344	317758	NC_000072.6	Gimap9	6.39E+00
gene11600	17122	NC_000071.6	Mxd4	6.39E+00
gene330	226971	NC_000067.6	Plekhb2	6.38E+00
gene32467	97820	NC_000079.6	4833439L19Rik	6.38E+00
gene38323	110920	NC_000082.6	Hspa13	6.38E+00
gene7770	16491	NC_000069.6	Kcna3	6.38E+00
gene42073	17283	NC_000085.6	Men1	6.38E+00
gene27838	216867	NC_000077.6	Slc16a11	6.38E+00
gene38423	ene=LOC1	NC_000082.6	LOC108168279	6.38E+00
gene26774	432537	NC_000077.6	Gm12009	6.37E+00
gene16777	66300	NC_000073.6	Inafm1	6.37E+00
gene11834	666455	NC_000071.6	Gm8115	6.37E+00
gene29154	26941	NC_000077.6	Slc9a3r1	6.37E+00
gene1436	22099	NC_000067.6	Tsn	6.37E+00
gene15460	66087	NC_000072.6	Emc3	6.37E+00
gene5542	67068	NC_000068.7	Dynlrb1	6.36E+00
gene19562	233890	NC_000073.6	Zfp768	6.36E+00

gene30594	protein;gb	NC_000078.6	Nrp	6.36E+00
gene42937	1E+08	NC_000085.6	4833438C02Rik	6.36E+00
gene40154	68047	NC_000083.6	Mpnd	6.36E+00
gene34808	52585	NC_000080.6	Dhrs1	6.36E+00
gene36215	211770	NC_000081.6	Trib1	6.35E+00
gene41585	70361	NC_000084.6	Lman1	6.35E+00
gene39613	27632	NC_000083.6	Nelfe	6.35E+00
gene3526	74192	NC_000068.7	Arpc5l	6.35E+00
gene27891	116891	NC_000077.6	Derl2	6.34E+00
gene17150	68891	NC_000073.6	Cd177	6.34E+00
gene13370	14086	NC_000071.6	Fscn1	6.34E+00
gene10687	1E+08	NC_000070.6	Gm13082	6.33E+00
gene40157	66530	NC_000083.6	Ubxn6	6.33E+00
gene34815	56532	NC_000080.6	Ripk3	6.33E+00
gene5786	21939	NC_000068.7	Cd40	6.33E+00
gene1986	64659	NC_000067.6	Mrps14	6.33E+00
gene7126	27049	NC_000069.6	Etv3	6.32E+00
gene28874	21429	NC_000077.6	Ubtf	6.32E+00
gene15043	56174	NC_000072.6	Nagk	6.31E+00
gene21262	67903	NC_000074.6	Gipc1	6.31E+00
gene3145	52838	NC_000068.7	Dnlz	6.31E+00
gene26441	12974	NC_000076.6	Cs	6.30E+00
gene28936	56494	NC_000077.6	Gosr2	6.30E+00
gene20158	26356	NC_000074.6	Ing1	6.30E+00
gene12709	56356	NC_000071.6	Gltp	6.30E+00
gene11852	14380	NC_000071.6	G6pd2	6.30E+00
gene3071	58202	NC_000068.7	Nelfb	6.30E+00
gene27856	216869	NC_000077.6	Arrb2	6.29E+00
gene11046	=Gene;ger	NC_000070.6	LOC108168990	6.29E+00
gene8526	383992	NC_000070.6	Rps11-ps3	6.29E+00
gene21490	23802	NC_000074.6	Amfr	6.29E+00
gene19596	20909	NC_000073.6	Stx4a	6.29E+00
gene1010	1E+08	NC_000067.6	Gm17764	6.29E+00
gene37925	80285	NC_000082.6	Parp9	6.29E+00
gene12715	68420	NC_000071.6	Ankrd13a	6.29E+00
gene36537	22117	NC_000081.6	Tst	6.28E+00
gene39835	654468	NC_000083.6	Gm7335	6.28E+00
gene32283	97863	NC_000079.6	Fam8a1	6.28E+00
gene19014	319236	NC_000073.6	Trim12c	6.27E+00
gene25463	80294	NC_000076.6	Pofut2	6.27E+00
gene42192	66395	NC_000085.6	Ahnak	6.27E+00
gene42095	107173	NC_000085.6	Gpr137	6.27E+00
gene37346	66049	NC_000082.6	Rogdi	6.26E+00
gene34732	69089	NC_000080.6	Oxa1l	6.26E+00
gene2228	22278	NC_000067.6	Usf1	6.26E+00
gene25412	14871	NC_000076.6	Gstt1	6.26E+00
gene35220	67168	NC_000080.6	Lpar6	6.26E+00
gene29331	208092	NC_000077.6	Chmp6	6.26E+00

gene42010	58859	NC_000085.6	Efemp2	6.25E+00
gene2250	59287	NC_000067.6	Ncstn	6.24E+00
gene16791	434127	NC_000073.6	Gm5586	6.24E+00
gene3083	99152	NC_000068.7	Anapc2	6.24E+00
gene41195	240219	NC_000084.6	Gm4949	6.24E+00
gene12300	11745	NC_000071.6	Anxa3	6.24E+00
gene1545	gene;gbkey	NC_000067.6	LOC100534274	6.22E+00
gene310	69668	NC_000067.6	Ccdc115	6.22E+00
gene29371	66156	NC_000077.6	Anapc11	6.22E+00
gene5944	1E+08	NC_000068.7	Gm14274	6.22E+00
gene42215	14156	NC_000085.6	Fen1	6.22E+00
gene20054	1E+08	NC_000074.6	Pet100	6.21E+00
gene4985	66552	NC_000068.7	Sppl2a	6.21E+00
gene4997	26371	NC_000068.7	Ciao1	6.20E+00
gene36549	72318	NC_000081.6	Cyth4	6.20E+00
gene33030	105171	NC_000079.6	Arrdc3	6.20E+00
gene32851	320163	NC_000079.6	4930525G20Rik	6.20E+00
gene18738	72981	NC_000073.6	Prkrir	6.20E+00
gene30859	73046	NC_000078.6	Glrx5	6.20E+00
gene31108	382639	NC_000078.6	Zbtb42	6.19E+00
gene37345	432995	NC_000082.6	Smim22	6.19E+00
gene17573	14751	NC_000073.6	Gpi1	6.19E+00
gene33526	208715	NC_000079.6	Hmgcs1	6.18E+00
gene20103	622568	NC_000074.6	Gm6334	6.18E+00
gene11045	11603	NC_000070.6	Agrn	6.18E+00
gene3297	30934	NC_000068.7	Tor1b	6.17E+00
gene42664	11475	NC_000085.6	Acta2	6.17E+00
gene42029	16998	NC_000085.6	Ltbp3	6.17E+00
gene41558	70223	NC_000084.6	Nars	6.16E+00
gene37549	69009	NC_000082.6	Thap7	6.16E+00
gene24453	66079	NC_000075.6	Tmem42	6.16E+00
gene26386	216445	NC_000076.6	Arhgap9	6.15E+00
gene37647	224045	NC_000082.6	Eif2b5	6.14E+00
gene40726	433158	NC_000084.6	Gm5500	6.14E+00
gene38727	22350	NC_000083.6	Ezr	6.14E+00
gene28531	66610	NC_000077.6	Abi3	6.14E+00
gene4879	17151	NC_000068.7	Ccndbp1	6.14E+00
gene39526	57875	NC_000083.6	Angptl4	6.13E+00
gene22987	16154	NC_000075.6	Il10ra	6.13E+00
gene36428	223649	NC_000081.6	Nrbp2	6.13E+00
gene5616	20014	NC_000068.7	Rpn2	6.13E+00
gene28134	19376	NC_000077.6	Rab34	6.12E+00
gene10883	100198	NC_000070.6	H6pd	6.12E+00
gene9336	623115	NC_000070.6	Gm11488	6.12E+00
gene26377	117150	NC_000076.6	Pip4k2c	6.11E+00
gene19505	233871	NC_000073.6	Atxn2l	6.11E+00
gene8800	1E+08	NC_000070.6	Toporsos	6.11E+00
gene2794	1E+08	NC_000068.7	Gm13217	6.11E+00

gene2216	57370	NC_000067.6	B4galt3	6.10E+00
gene311	27993	NC_000067.6	Imp4	6.10E+00
gene11648	17713	NC_000071.6	Grpel1	6.10E+00
gene7748	11950	NC_000069.6	Atp5f1	6.10E+00
gene5473	68559	NC_000068.7	Pdrg1	6.09E+00
gene42825	69534	NC_000085.6	Avpi1	6.09E+00
gene29909	66109	NC_000078.6	Tspan13	6.09E+00
gene32531	57266	NC_000079.6	Cxcl14	6.08E+00
gene2227	226652	NC_000067.6	Arhgap30	6.08E+00
gene19101	27397	NC_000073.6	Mrpl17	6.08E+00
gene20214	14456	NC_000074.6	Gas6	6.08E+00
gene21738	66164	NC_000074.6	Nip7	6.07E+00
gene39793	1E+08	NC_000083.6	Gm19807	6.07E+00
gene2251	12847	NC_000067.6	Copa	6.07E+00
gene42050	14109	NC_000085.6	Fau	6.07E+00
gene21658	104394	NC_000074.6	E2f4	6.07E+00
gene12589	gene=LOC	NC_000071.6	LOC102639044	6.07E+00
gene34805	28113	NC_000080.6	Tinf2	6.06E+00
gene22938	13478	NC_000075.6	Dpagt1	6.06E+00
gene41998	414115	NC_000085.6	D330050I16Rik	6.06E+00
gene15788	12444	NC_000072.6	Ccnd2	6.06E+00
gene9165	619973	NC_000070.6	Gm12538	6.05E+00
gene3259	194642	NC_000068.7	Slc39a1-ps	6.05E+00
gene1930	240832	NC_000067.6	Tor1aip2	6.05E+00
gene41015	13476	NC_000084.6	Reep5	6.05E+00
gene7214	17827	NC_000069.6	Mtx1	6.04E+00
gene17851	66124	NC_000073.6	Josd2	6.04E+00
gene27123	382510	NC_000077.6	Gm12161	6.03E+00
gene4721	73024	NC_000068.7	Emc7	6.03E+00
gene329	214469	NC_000067.6	Fam168b	6.03E+00
gene38388	11820	NC_000082.6	App	6.02E+00
gene27301	22210	NC_000077.6	Ube2b	6.02E+00
gene41451	=Gene;ger	NC_000084.6	LOC108167336	6.01E+00
gene7463	ene=LOC1	NC_000069.6	LOC101055724	6.01E+00
gene9250	20530	NC_000070.6	Slc31a2	6.01E+00
gene32343	54339	NC_000079.6	Tes3-ps	6.00E+00
gene2366	30928	NC_000067.6	Zbtb18	6.00E+00
gene5717	22403	NC_000068.7	Wisp2	6.00E+00
gene6528	665655	NC_000069.6	Gm7733	6.00E+00
gene20751	14081	NC_000074.6	Acsl1	5.99E+00
gene15709	14794	NC_000072.6	Spsb2	5.99E+00
gene21085	16453	NC_000074.6	Jak3	5.99E+00
gene21620	56437	NC_000074.6	Rrad	5.99E+00
gene21706	71986	NC_000074.6	Ddx28	5.98E+00
gene42049	18120	NC_000085.6	Mrpl49	5.98E+00
gene26650	78926	NC_000077.6	Gas2l1	5.98E+00
gene7778	68576	NC_000069.6	Lamtor5	5.98E+00
gene2080	108735	NC_000067.6	Sft2d2	5.98E+00

gene15755	12527	NC_000072.6	Cd9	5.97E+00
gene39219	214505	NC_000083.6	Gnptg	5.97E+00
gene25594	70719	NC_000076.6	Hmha1	5.97E+00
gene41778	27366	NC_000084.6	Txnl4a	5.97E+00
gene28506	16400	NC_000077.6	Itga3	5.96E+00
gene9884	68273	NC_000070.6	Pomgnt1	5.96E+00
gene5292	1.1E+08	NC_000068.7	Gm39954	5.96E+00
gene11529	14208	NC_000071.6	Ppm1g	5.96E+00
gene28099	216963	NC_000077.6	Git1	5.96E+00
gene33854	11750	NC_000080.6	Anxa7	5.95E+00
gene36901	380959	NC_000081.6	Alg10b	5.95E+00
gene41907	1E+08	NC_000085.6	Gm29846	5.95E+00
gene39058	74670	NC_000083.6	Zfp943	5.95E+00
gene29377	17134	NC_000077.6	Mafg	5.95E+00
gene22451	66163	NC_000075.6	Mrpl4	5.95E+00
gene8685	1E+08	NC_000070.6	Gm11914	5.94E+00
gene15157	243529	NC_000072.6	H1fx	5.94E+00
gene16165	79362	NC_000072.6	Bhlhe41	5.93E+00
gene18459	14159	NC_000073.6	Fes	5.93E+00
gene21227	236010	NC_000074.6	Gm4899	5.93E+00
gene32474	105239	NC_000079.6	Rnf44	5.93E+00
gene21015	211228	NC_000074.6	Lrrc25	5.93E+00
gene39630	22321	NC_000083.6	Vars	5.92E+00
gene8844	53598	NC_000070.6	Dctn3	5.92E+00
gene11527	101023	NC_000071.6	Zfp513	5.92E+00
gene30092	79459	NC_000078.6	Aldoart2	5.92E+00
gene16285	664841	NC_000073.6	Gm7363	5.92E+00
gene32809	67530	NC_000079.6	Uqcrb	5.91E+00
gene22501	235043	NC_000075.6	Tmem205	5.91E+00
gene28630	ene=LOC1	NC_000077.6	LOC102633230	5.91E+00
gene40701	16573	NC_000084.6	Kif5b	5.91E+00
gene14335	243371	NC_000072.6	Lrrc61	5.91E+00
gene40217	224912	NC_000083.6	Crb3	5.90E+00
gene39311	21652	NC_000083.6	Phf1	5.90E+00
gene32347	18295	NC_000079.6	Ogn	5.90E+00
gene17951	69903	NC_000073.6	Rasip1	5.90E+00
gene11528	=Gene;ger	NC_000071.6	LOC108169037	5.90E+00
gene37970	1.1E+08	NC_000082.6	Gm41452	5.89E+00
gene18660	233490	NC_000073.6	Crebzf	5.89E+00
gene19871	23997	NC_000073.6	Psmd13	5.89E+00
gene28287	20302	NC_000077.6	Ccl3	5.89E+00
gene19230	26451	NC_000073.6	Rpl27a	5.89E+00
gene15159	19349	NC_000072.6	Rab7	5.88E+00
gene7491	66471	NC_000069.6	Anp32e	5.88E+00
gene26182	544741	NC_000076.6	Gm5781	5.88E+00
gene30247	72736	NC_000078.6	Tmx1	5.87E+00
gene39742	ene=LOC1	NC_000083.6	LOC102636255	5.87E+00
gene28992	67163	NC_000077.6	Ccdc47	5.87E+00

gene11574	100689	NC_000071.6	Spon2	5.87E+00
gene34275	1E+08	NC_000080.6	Gm16439	5.86E+00
gene3089	227619	NC_000068.7	Man1b1	5.86E+00
gene26923	1E+08	NC_000077.6	Gm3810	5.86E+00
gene25668	16969	NC_000076.6	Zbtb7a	5.85E+00
gene1727	1E+08	NC_000067.6	Gm29718	5.85E+00
gene39286	14570	NC_000083.6	Arhgdig	5.85E+00
gene8	20671	NC_000067.6	Sox17	5.85E+00
gene24883	72480	NC_000076.6	Tspyl4	5.84E+00
gene17417	23900	NC_000073.6	Hcst	5.84E+00
gene36471	54151	NC_000081.6	Cyhr1	5.84E+00
gene39154	245847	NC_000083.6	Amdhd2	5.84E+00
gene40196	106639	NC_000083.6	Vmac	5.84E+00
gene28110	1E+08	NC_000077.6	Gm36875	5.83E+00
gene7793	12977	NC_000069.6	Csf1	5.83E+00
gene15470	110379	NC_000072.6	Sec13	5.83E+00
gene13019	665760	NC_000071.6	Gm7774	5.83E+00
gene39542	54218	NC_000083.6	B3galt4	5.83E+00
gene28145	22248	NC_000077.6	Unc119	5.82E+00
gene21506	434339	NC_000074.6	Ap3s1-ps2	5.82E+00
gene4557	12359	NC_000068.7	Cat	5.81E+00
gene41035	13653	NC_000084.6	Egr1	5.80E+00
gene23939	667456	NC_000075.6	Gm8641	5.80E+00
gene8054	668831	NC_000069.6	Gm9387	5.80E+00
gene41036	225363	NC_000084.6	Etf1	5.80E+00
gene8984	230125	NC_000070.6	Slc25a51	5.79E+00
gene23421	102595	NC_000075.6	Plekho2	5.79E+00
gene3621	75423	NC_000068.7	Arl5a	5.79E+00
gene23425	18247	NC_000075.6	Oaz2	5.79E+00
gene27853	668466	NC_000077.6	Gm12312	5.79E+00
gene29063	14674	NC_000077.6	Gna13	5.79E+00
gene43147	11757	NC_000085.6	Prdx3	5.79E+00
gene37352	239691	NC_000082.6	AU021092	5.78E+00
gene3298	30931	NC_000068.7	Tor1a	5.78E+00
gene40348	21815	NC_000083.6	Tgif1	5.77E+00
gene42047	74126	NC_000085.6	Syvn1	5.77E+00
gene37216	68744	NC_000081.6	Zfp740	5.77E+00
gene4475	12521	NC_000068.7	Cd82	5.77E+00
gene27498	67862	NC_000077.6	2310033P09Rik	5.76E+00
gene24114	235606	NC_000075.6	Apeh	5.76E+00
gene3261	13804	NC_000068.7	Endog	5.76E+00
gene28462	18102	NC_000077.6	Nme1	5.76E+00
gene3598	227885	NC_000068.7	Gm13498	5.75E+00
gene33861	218811	NC_000080.6	Sec24c	5.75E+00
gene39317	70772	NC_000083.6	Ggnbp1	5.75E+00
gene4894	212670	NC_000068.7	Catsper2	5.75E+00
gene1507	74117	NC_000067.6	Actr3	5.75E+00
gene4046	228140	NC_000068.7	Tnks1bp1	5.74E+00

gene5832	68949	NC_000068.7	Zfas1	5.74E+00
gene19692	12237	NC_000073.6	Bub3	5.73E+00
gene10417	77490	NC_000070.6	E130218I03Rik	5.73E+00
gene36012	21847	NC_000081.6	Klf10	5.73E+00
gene30801	27225	NC_000078.6	Ddx24	5.73E+00
gene10265	109658	NC_000070.6	Txlna	5.73E+00
gene17970	19158	NC_000073.6	Cyth2	5.72E+00
gene15388	70047	NC_000072.6	Trnt1	5.72E+00
gene23246	20361	NC_000075.6	Sema7a	5.71E+00
gene2071	56429	NC_000067.6	Dpt	5.71E+00
gene30139	18949	NC_000078.6	Pnn	5.71E+00
gene34116	14661	NC_000080.6	Glud1	5.71E+00
gene8976	68512	NC_000070.6	Tomm5	5.71E+00
gene14743	434036	NC_000072.6	Igkv13-85	5.71E+00
gene28987	71833	NC_000077.6	Dcaf7	5.71E+00
gene33540	1E+08	NC_000079.6	Gm36161	5.71E+00
gene39182	381073	NC_000083.6	Npw	5.70E+00
gene17691	1E+08	NC_000073.6	Gm2058	5.70E+00
gene36123	19357	NC_000081.6	Rad21	5.70E+00
gene35691	67281	NC_000081.6	Rpl37	5.69E+00
gene23843	54371	NC_000075.6	Chst2	5.69E+00
gene5507	18843	NC_000068.7	Bpifa1	5.69E+00
gene5604	77799	NC_000068.7	Sla2	5.68E+00
gene26680	17898	NC_000077.6	Myl7	5.68E+00
gene7180	16905	NC_000069.6	Lmna	5.68E+00
gene7945	71994	NC_000069.6	Cnn3	5.68E+00
gene13745	71900	NC_000072.6	Tmem106b	5.68E+00
gene16915	1.1E+08	NC_000073.6	Gm38955	5.67E+00
gene34947	219144	NC_000080.6	Arl11	5.67E+00
gene11313	19181	NC_000071.6	Psmc2	5.67E+00
gene17305	386655	NC_000073.6	Eid2	5.67E+00
gene14757	236047	NC_000072.6	Igkv4-74	5.67E+00
gene28888	57265	NC_000077.6	Fzd2	5.67E+00
gene24477	12768	NC_000075.6	Ccr1	5.66E+00
gene38524	16155	NC_000082.6	Il10rb	5.66E+00
gene30062	104725	NC_000078.6	Sptssa	5.66E+00
gene23866	19823	NC_000075.6	Rnf7	5.66E+00
gene22687	13663	NC_000075.6	Ei24	5.66E+00
gene13434	231887	NC_000071.6	Pdap1	5.66E+00
gene41547	1E+08	NC_000084.6	Gm19076	5.65E+00
gene3422	13830	NC_000068.7	Stom	5.65E+00
gene17337	18036	NC_000073.6	Nfkbib	5.65E+00
gene38535	20658	NC_000082.6	Son	5.64E+00
gene6905	ene=LOC1	NC_000069.6	LOC105244463	5.64E+00
gene15711	22225	NC_000072.6	Usp5	5.64E+00
gene3947	623706	NC_000068.7	Gm13651	5.63E+00
gene11299	75172	NC_000071.6	Ccdc146	5.63E+00
gene27773	327951	NC_000077.6	Cyb5d1	5.63E+00

gene9848	67182	NC_000070.6	Pdzk1ip1	5.63E+00
gene12855	71990	NC_000071.6	Ddx54	5.63E+00
gene36861	67976	NC_000081.6	Trabd	5.63E+00
gene23389	53869	NC_000075.6	Rab11a	5.63E+00
gene30738	19179	NC_000078.6	Psmc1	5.62E+00
gene33223	218490	NC_000079.6	Btf3	5.62E+00
gene27381	11749	NC_000077.6	Anxa6	5.62E+00
gene30538	68581	NC_000078.6	Tmed10	5.62E+00
gene40214	16549	NC_000083.6	Khsrp	5.61E+00
gene38656	432993	NC_000083.6	Gm5479	5.61E+00
gene35593	1E+08	NC_000080.6	Dnajc3	5.60E+00
gene28479	71889	NC_000077.6	Epn3	5.60E+00
gene38148	433027	NC_000082.6	Gm5487	5.60E+00
gene16794	20514	NC_000073.6	Slc1a5	5.60E+00
gene30776	19141	NC_000078.6	Lgmn	5.59E+00
gene26262	68212	NC_000076.6	Tmbim4	5.59E+00
gene21744	66427	NC_000074.6	Cyb5b	5.59E+00
gene24118	13138	NC_000075.6	Dag1	5.58E+00
gene6014	66390	NC_000068.7	Slmo2	5.58E+00
gene36531	17972	NC_000081.6	Ncf4	5.58E+00
gene21782	52335	NC_000074.6	Atxn1l	5.58E+00
gene29756	20135	NC_000078.6	Rrm2	5.58E+00
gene6725	67890	NC_000069.6	Ufm1	5.58E+00
gene36356	223626	NC_000081.6	Them6	5.57E+00
gene41940	245857	NC_000085.6	Ssh3	5.57E+00
gene16920	12051	NC_000073.6	Bcl3	5.57E+00
gene17348	60595	NC_000073.6	Actn4	5.56E+00
gene5494	13589	NC_000068.7	Mapre1	5.56E+00
gene14347	231932	NC_000072.6	Gimap7	5.56E+00
gene10844	20810	NC_000070.6	Srm	5.55E+00
gene42507	gene=LOC1	NC_000085.6	LOC102638448	5.55E+00
gene27148	1E+08	NC_000077.6	Gm12168	5.54E+00
gene19598	233905	NC_000073.6	Zfp646	5.54E+00
gene40710	19330	NC_000084.6	Rab18	5.54E+00
gene12918	18438	NC_000071.6	P2rx4	5.54E+00
gene21265	68278	NC_000074.6	Ddx39	5.53E+00
gene29396	104318	NC_000077.6	Csnk1d	5.53E+00
gene34313	625377	NC_000080.6	Gm6580	5.53E+00
gene10546	18782	NC_000070.6	Pla2g2d	5.53E+00
gene7732	1.1E+08	NC_000069.6	Gm40117	5.52E+00
gene24965	20598	NC_000076.6	Smpd2	5.52E+00
gene11378	53312	NC_000071.6	Nub1	5.52E+00
gene20564	19053	NC_000074.6	Ppp2cb	5.52E+00
gene13226	68929	NC_000071.6	Mospd3	5.52E+00
gene29290	76025	NC_000077.6	Cant1	5.51E+00
gene28804	71743	NC_000077.6	Coasy	5.51E+00
gene5278	228715	NC_000068.7	Gm561	5.51E+00
gene24079	56368	NC_000075.6	Cyb561d2	5.51E+00

gene30531	217715	NC_000078.6	Eif2b2	5.51E+00
gene32509	72935	NC_000079.6	Ddx41	5.51E+00
gene17857	272382	NC_000073.6	Spib	5.51E+00
gene12910	11938	NC_000071.6	Atp2a2	5.51E+00
gene34327	12793	NC_000080.6	Cnih1	5.50E+00
gene23863	11933	NC_000075.6	Atp1b3	5.50E+00
gene15410	1E+08	NC_000072.6	Gm35417	5.50E+00
gene6465	69276	NC_000069.6	Sec62	5.50E+00
gene28970	73431	NC_000077.6	1700052K11Rik	5.49E+00
gene1793	50778	NC_000067.6	Rgs1	5.49E+00
gene36667	57259	NC_000081.6	Tob2	5.49E+00
gene36361	23936	NC_000081.6	Lynx1	5.49E+00
gene10234	11637	NC_000070.6	Ak2	5.49E+00
gene14735	434033	NC_000072.6	Igkv4-91	5.47E+00
gene1667	72017	NC_000067.6	Cyb5r1	5.47E+00
gene34305	382884	NC_000080.6	Gm5206	5.47E+00
gene36523	56551	NC_000081.6	Txn2	5.47E+00
gene11169	652925	NC_000071.6	Tmem243	5.46E+00
gene28358	67300	NC_000077.6	Cltc	5.46E+00
gene21659	234683	NC_000074.6	Elmo3	5.46E+00
gene9786	666144	NC_000070.6	Gm12749	5.46E+00
gene20199	=Gene;ger	NC_000074.6	LOC108167522	5.45E+00
gene24132	71472	NC_000075.6	Usp19	5.45E+00
gene37423	622271	NC_000082.6	Gm6305	5.45E+00
gene9958	80509	NC_000070.6	Med8	5.45E+00
gene25667	26396	NC_000076.6	Map2k2	5.45E+00
gene12867	246730	NC_000071.6	Oas1a	5.45E+00
gene17160	12518	NC_000073.6	Cd79a	5.44E+00
gene29866	1E+08	NC_000078.6	Gm18726	5.44E+00
gene21711	192654	NC_000074.6	Pla2g15	5.44E+00
gene7487	229600	NC_000069.6	BC028528	5.44E+00
gene42030	78891	NC_000085.6	Scyl1	5.44E+00
gene20083	26400	NC_000074.6	Map2k7	5.44E+00
gene8980	66362	NC_000070.6	Exosc3	5.44E+00
gene925	56812	NC_000067.6	Dnajb2	5.44E+00
gene9030	666473	NC_000070.6	Gm12430	5.43E+00
gene5086	56264	NC_000068.7	Cpxm1	5.43E+00
gene28797	20848	NC_000077.6	Stat3	5.43E+00
gene25562	110012	NC_000076.6	Tpgs1	5.43E+00
gene27568	237782	NC_000077.6	Smcr8	5.43E+00
gene10846	230908	NC_000070.6	Tardbp	5.43E+00
gene39968	56055	NC_000083.6	Gtpbp2	5.42E+00
gene43020	1E+08	NC_000085.6	Nutf2-ps1	5.42E+00
gene36416	22122	NC_000081.6	Tsta3	5.42E+00
gene30236	64010	NC_000078.6	Sav1	5.42E+00
gene40679	664931	NC_000084.6	Gm7411	5.41E+00
gene28994	56095	NC_000077.6	Ftsj3	5.41E+00
gene5338	56488	NC_000068.7	Nxt1	5.41E+00

gene10615	213491	NC_000070.6	Szrd1	5.41E+00
gene3148	68112	NC_000068.7	Sdccag3	5.41E+00
gene21064	64296	NC_000074.6	Abhd8	5.40E+00
gene42087	78317	NC_000085.6	Ccdc88b	5.40E+00
gene40115	1E+08	NC_000083.6	Rab5a-ps	5.40E+00
gene16655	1E+08	NC_000073.6	Gm31649	5.40E+00
gene36575	223691	NC_000081.6	Eif3l	5.40E+00
gene8836	76992	NC_000070.6	Fam219aos	5.39E+00
gene41969	66419	NC_000085.6	Mrpl11	5.39E+00
gene21263	320795	NC_000074.6	Pkn1	5.39E+00
gene37413	56722	NC_000082.6	Litaf	5.39E+00
gene3439	68375	NC_000068.7	Ndufa8	5.39E+00
gene29212	60441	NC_000077.6	Mrpl38	5.39E+00
gene28381	18952	NC_000077.6	sep-04	5.39E+00
gene28137	20316	NC_000077.6	Sdf2	5.38E+00
gene34025	66175	NC_000080.6	Mustn1	5.37E+00
gene32917	1E+08	NC_000079.6	Gm4057	5.37E+00
gene29875	19027	NC_000078.6	Sypl	5.37E+00
gene42451	1E+08	NC_000085.6	Gm17819	5.36E+00
gene5430	67231	NC_000068.7	Tbc1d20	5.36E+00
gene2017	67292	NC_000067.6	Pigc	5.36E+00
gene7827	20226	NC_000069.6	Sars	5.36E+00
gene24985	54198	NC_000076.6	Snx3	5.35E+00
gene15856	93694	NC_000072.6	Clec2d	5.35E+00
gene39552	19763	NC_000083.6	Ring1	5.35E+00
gene14775	546244	NC_000072.6	Igkv4-61	5.34E+00
gene36955	19230	NC_000081.6	Twf1	5.34E+00
gene3349	20448	NC_000068.7	St6galnac4	5.34E+00
gene38752	1E+08	NC_000083.6	Rnaset2a	5.33E+00
gene17309	66525	NC_000073.6	Timm50	5.33E+00
gene29375	209011	NC_000077.6	Sirt7	5.32E+00
gene13131	68758	NC_000071.6	Abhd11	5.31E+00
gene600	15510	NC_000067.6	Hspd1	5.31E+00
gene39648	114585	NC_000083.6	D17H6S53E	5.31E+00
gene16273	66078	NC_000073.6	Tsen34	5.31E+00
gene19929	54473	NC_000073.6	Tollip	5.30E+00
gene17463	22282	NC_000073.6	Usf2	5.30E+00
gene21124	270066	NC_000074.6	Slc35e1	5.30E+00
gene12370	231507	NC_000071.6	Plac8	5.30E+00
gene4407	20375	NC_000068.7	Spi1	5.30E+00
gene42174	14705	NC_000085.6	Bscl2	5.29E+00
gene32969	434490	NC_000079.6	Gm5626	5.29E+00
gene26944	67939	NC_000077.6	Prorsd1	5.29E+00
gene17777	110826	NC_000073.6	Etfb	5.29E+00
gene4433	1.1E+08	NC_000068.7	Gm39876	5.29E+00
gene38897	19170	NC_000083.6	Psmb1	5.29E+00
gene20439	15930	NC_000074.6	Ido1	5.29E+00
gene23348	14155	NC_000075.6	Fem1b	5.29E+00

gene13508	606519	NC_000071.6	Gm6054	5.28E+00
gene17898	52377	NC_000073.6	Rcn3	5.28E+00
gene37242	67488	NC_000081.6	Calcoco1	5.28E+00
gene11025	79554	NC_000070.6	Cptp	5.28E+00
gene27322	623205	NC_000077.6	Gm11189	5.27E+00
gene37071	1.1E+08	NC_000081.6	Gm41396	5.27E+00
gene42108	20867	NC_000085.6	Stip1	5.27E+00
gene31564	665008	NC_000079.6	Gm7446	5.27E+00
gene10321	623230	NC_000070.6	Tmem200b	5.27E+00
gene8811	1E+08	NC_000070.6	Gm34213	5.27E+00
gene12794	59043	NC_000071.6	Wsb2	5.27E+00
gene26881	22183	NC_000077.6	Zrsr1	5.26E+00
gene7488	226548	NC_000069.6	Aph1a	5.26E+00
gene25088	52014	NC_000076.6	Nus1	5.26E+00
gene42262	109225	NC_000085.6	Ms4a7	5.26E+00
gene37841	677155	NC_000082.6	Gm15703	5.25E+00
gene28423	217069	NC_000077.6	Trim25	5.25E+00
gene20247	634008	NC_000074.6	Gm7128	5.25E+00
gene24040	235584	NC_000075.6	Dusp7	5.24E+00
gene12017	16542	NC_000071.6	Kdr	5.24E+00
gene7812	14679	NC_000069.6	Gnai3	5.24E+00
gene20996	234374	NC_000074.6	Ddx49	5.24E+00
gene17891	233208	NC_000073.6	Scaf1	5.24E+00
gene40297	19765	NC_000083.6	Ralbp1	5.23E+00
gene29009	18613	NC_000077.6	Pecam1	5.23E+00
gene22040	1E+08	NC_000074.6	2810013P06Rik	5.23E+00
gene23384	93841	NC_000075.6	Uchl4	5.22E+00
gene28581	103711	NC_000077.6	Pnpo	5.22E+00
gene29193	140721	NC_000077.6	Caskin2	5.22E+00
gene41803	1E+08	NC_000084.6	Gm17383	5.22E+00
gene31441	667411	NC_000079.6	Gm8616	5.22E+00
gene2466	27058	NC_000067.6	Srp9	5.21E+00
gene278	1E+08	NC_000067.6	Gm19680	5.21E+00
gene19410	67003	NC_000073.6	Uqcrc2	5.21E+00
gene28241	66983	NC_000077.6	Zfp830	5.20E+00
gene29457	1E+08	NC_000078.6	Gm17816	5.20E+00
gene41997	20227	NC_000085.6	Sart1	5.19E+00
gene8430	1.1E+08	NC_000069.6	Gm40187	5.19E+00
gene18007	1.1E+08	NC_000073.6	Gm39011	5.19E+00
gene29473	66055	NC_000078.6	Sf3b6	5.19E+00
gene25641	208228	NC_000076.6	Mob3a	5.19E+00
gene9031	56737	NC_000070.6	Alg2	5.19E+00
gene2605	11910	NC_000067.6	Atf3	5.18E+00
gene14692	243431	NC_000072.6	Igkv9-124	5.17E+00
gene6687	66377	NC_000069.6	Ndufc1	5.17E+00
gene33874	1E+08	NC_000080.6	Gm30274	5.17E+00
gene39609	112403	NC_000083.6	Dxo	5.17E+00
gene19095	101867	NC_000073.6	Rrp8	5.17E+00

gene21029	18709	NC_000074.6	Pik3r2	5.16E+00
gene28233	20293	NC_000077.6	Ccl12	5.16E+00
gene26716	665339	NC_000077.6	Gm11977	5.16E+00
gene15467	381801	NC_000072.6	Tatdn2	5.16E+00
gene10302	1E+08	NC_000070.6	Gm38599	5.16E+00
gene4857	98878	NC_000068.7	Ehd4	5.16E+00
gene24083	56289	NC_000075.6	Rassf1	5.16E+00
gene12557	545792	NC_000071.6	Gm5871	5.15E+00
gene7163	12462	NC_000069.6	Cct3	5.15E+00
gene33090	logene;gbk	NC_000079.6	LOC100534278	5.14E+00
gene41976	107392	NC_000085.6	Brms1	5.14E+00
gene43127	12979	NC_000085.6	Csf1r-ps	5.14E+00
gene19327	56372	NC_000073.6	1110004F10Rik	5.14E+00
gene41913	66387	NC_000085.6	Nudt8	5.14E+00
gene37582	15547	NC_000082.6	Trmt2a	5.14E+00
gene4451	228368	NC_000068.7	Slc35c1	5.13E+00
gene3056	215705	NC_000068.7	Arrdc1	5.13E+00
gene15911	56449	NC_000072.6	Ybx3	5.13E+00
gene13212	22051	NC_000071.6	Trip6	5.13E+00
gene16908	56457	NC_000073.6	Clptm1	5.13E+00
gene42930	16825	NC_000085.6	Ldb1	5.12E+00
gene1558	212439	NC_000067.6	AA986860	5.12E+00
gene38523	15976	NC_000082.6	Ifnar2	5.12E+00
gene11344	66169	NC_000071.6	Tomm7	5.11E+00
gene41996	69860	NC_000085.6	Eif1ad	5.11E+00
gene21380	14322	NC_000074.6	Fth-ps3	5.11E+00
gene19960	16985	NC_000073.6	Lsp1	5.11E+00
gene5851	407243	NC_000068.7	Tmem189	5.11E+00
gene27892	67139	NC_000077.6	Mis12	5.11E+00
gene42185	72727	NC_000085.6	B3gat3	5.10E+00
gene42059	66406	NC_000085.6	Sac3d1	5.10E+00
gene9193	433712	NC_000070.6	Rpsa-ps11	5.10E+00
gene28622	65019	NC_000077.6	Rpl23	5.10E+00
gene41075	109169	NC_000084.6	Igip	5.10E+00
gene37351	27426	NC_000082.6	Nagpa	5.09E+00
gene24486	75135	NC_000075.6	4930526I15Rik	5.09E+00
gene1647	116847	NC_000067.6	Prelp	5.09E+00
gene36201	75841	NC_000081.6	Rnf139	5.09E+00
gene22229	78100	NC_000075.6	Msantd4	5.08E+00
gene26721	16009	NC_000077.6	Igfbp3	5.08E+00
gene28899	75689	NC_000077.6	Higd1b	5.08E+00
gene27081	108645	NC_000077.6	Mat2b	5.08E+00
gene28580	217138	NC_000077.6	Prr15l	5.07E+00
gene13337	78339	NC_000071.6	Ttyh3	5.07E+00
gene42899	226153	NC_000085.6	Peo1	5.07E+00
gene33205	13803	NC_000079.6	Enc1	5.07E+00
gene26014	71832	NC_000076.6	Csl	5.07E+00
gene36613	18591	NC_000081.6	Pdgfb	5.06E+00

gene9956	68180	NC_000070.6	Hyi	5.06E+00
gene28206	22680	NC_000077.6	Zfp207	5.06E+00
gene20155	102132	NC_000074.6	E230013L22Rik	5.05E+00
gene4019	54598	NC_000068.7	Calcrl	5.05E+00
gene19513	1.1E+08	NC_000073.6	Gm39087	5.05E+00
gene12760	77407	NC_000071.6	Rab35	5.05E+00
gene42886	319594	NC_000085.6	Hif1an	5.05E+00
gene15512	67784	NC_000072.6	Plxnd1	5.05E+00
gene42210	60527	NC_000085.6	Fads3	5.05E+00
gene17340	320435	NC_000073.6	Rinl	5.05E+00
gene24035	81897	NC_000075.6	Tlr9	5.04E+00
gene27750	66910	NC_000077.6	Tmem107	5.04E+00
gene14460	52440	NC_000072.6	Tax1bp1	5.04E+00
gene41845	109672	NC_000084.6	Cyb5a	5.04E+00
gene40229	78308	NC_000083.6	Gpr108	5.04E+00
gene24763	53331	NC_000076.6	Stx7	5.03E+00
gene20685	11988	NC_000074.6	Slc7a2	5.03E+00
gene12848	71772	NC_000071.6	Plbd2	5.02E+00
gene27057	211347	NC_000077.6	Pank3	5.02E+00
gene11554	14284	NC_000071.6	Fosl2	5.02E+00
gene14788	385291	NC_000072.6	Igkv4-54	5.02E+00
gene19896	66985	NC_000073.6	Rassf7	5.01E+00
gene20014	14082	NC_000073.6	Fadd	5.01E+00
gene40604	17075	NC_000083.6	Epcam	5.01E+00
gene18689	260347	NC_000073.6	Gm5037	5.01E+00
gene1929	208263	NC_000067.6	Tor1aip1	5.01E+00
gene22685	16430	NC_000075.6	Stt3a	5.01E+00
gene14053	54484	NC_000072.6	Mkrn1	5.01E+00
gene23773	12044	NC_000075.6	Bcl2a1a	5.01E+00
gene19497	73658	NC_000073.6	Spns1	5.00E+00
gene3294	64292	NC_000068.7	Ptges	5.00E+00
gene34018	18753	NC_000080.6	Prkcd	5.00E+00
gene24091	20347	NC_000075.6	Sema3b	5.00E+00
gene4994	73338	NC_000068.7	Itpripl1	5.00E+00
gene38667	624144	NC_000083.6	Gm6475	5.00E+00
gene1688	215615	NC_000067.6	Rnpep	4.99E+00
gene42102	22340	NC_000085.6	Vegfb	4.99E+00
gene3275	110854	NC_000068.7	Ptpa	4.99E+00
gene19236	56786	NC_000073.6	Tmem9b	4.99E+00
gene12789	23980	NC_000071.6	Pebp1	4.99E+00
gene12604	100608	NC_000071.6	Noc4l	4.99E+00
gene403	68833	NC_000067.6	Pdcl3	4.99E+00
gene24021	664981	NC_000075.6	Gm7436	4.98E+00
gene17202	232984	NC_000073.6	B3gnt8	4.98E+00
gene20164	624710	NC_000074.6	Gm6524	4.98E+00
gene10356	230787	NC_000070.6	Themis2	4.98E+00
gene10596	108911	NC_000070.6	Rcc2	4.98E+00
gene25411	103140	NC_000076.6	Gstt3	4.97E+00

gene33784	667205	NC_000080.6	Gm8514	4.97E+00
gene22957	13209	NC_000075.6	Ddx6	4.97E+00
gene26392	407790	NC_000076.6	Ndufa4l2	4.97E+00
gene375	67387	NC_000067.6	Unc50	4.97E+00
gene29780	58185	NC_000078.6	Rsad2	4.96E+00
gene11821	12425	NC_000071.6	Cckar	4.96E+00
gene42171	70387	NC_000085.6	Ttc9c	4.96E+00
gene11661	17160	NC_000071.6	Man2b2	4.95E+00
gene5766	68612	NC_000068.7	Ube2c	4.95E+00
gene10416	68040	NC_000070.6	Zfp593	4.95E+00
gene41014	66384	NC_000084.6	Srp19	4.95E+00
gene33866	268721	NC_000080.6	Zswim8	4.94E+00
gene8535	60599	NC_000070.6	Trp53inp1	4.94E+00
gene38164	66391	NC_000082.6	Zbtb11os1	4.94E+00
gene20131	68904	NC_000074.6	Abhd13	4.94E+00
gene15650	12267	NC_000072.6	C3ar1	4.94E+00
gene24946	1E+08	NC_000076.6	Gm18671	4.94E+00
gene7642	74645	NC_000069.6	Fam46c	4.93E+00
gene4029	98985	NC_000068.7	Clp1	4.93E+00
gene36018	54375	NC_000081.6	Azin1	4.93E+00
gene40197	69875	NC_000083.6	Ndufa11	4.93E+00
gene42987	14873	NC_000085.6	Gsto1	4.93E+00
gene9911	76608	NC_000070.6	Hectd3	4.93E+00
gene27811	83813	NC_000077.6	Tnk1	4.93E+00
gene9903	68276	NC_000070.6	Toe1	4.92E+00
gene39203	79043	NC_000083.6	Spsb3	4.92E+00
gene17879	59047	NC_000073.6	Pnkp	4.92E+00
gene9969	69072	NC_000070.6	Ebna1bp2	4.91E+00
gene4548	12505	NC_000068.7	Cd44	4.91E+00
gene41304	12583	NC_000084.6	Cdo1	4.91E+00
gene22151	102058	NC_000074.6	Exoc8	4.91E+00
gene18839	16332	NC_000073.6	Inppl1	4.91E+00
gene6177	229003	NC_000068.7	Helz2	4.91E+00
gene41922	18739	NC_000085.6	Pitpnm1	4.91E+00
gene32957	18570	NC_000079.6	Pdcd6	4.91E+00
gene597	81898	NC_000067.6	Sf3b1	4.91E+00
gene11733	242960	NC_000071.6	Fbxl5	4.90E+00
gene10178	100206	NC_000070.6	Adprhl2	4.90E+00
gene13720	1E+08	NC_000072.6	Gm16042	4.90E+00
gene13122	13717	NC_000071.6	Eln	4.90E+00
gene42186	19881	NC_000085.6	Rom1	4.90E+00
gene15153	54161	NC_000072.6	Copg1	4.90E+00
gene19171	1E+08	NC_000073.6	Gm4199	4.90E+00
gene28143	11676	NC_000077.6	Aldoc	4.90E+00
gene29590	1E+08	NC_000078.6	Gm9847	4.89E+00
gene12182	57349	NC_000071.6	Ppbp	4.89E+00
gene22999	18554	NC_000075.6	Pcsk7	4.89E+00
gene23985	321022	NC_000075.6	Cdv3	4.89E+00

gene16770	108124	NC_000073.6	Napa	4.89E+00
gene37556	1E+08	NC_000082.6	Gm18335	4.89E+00
gene11335	269630	NC_000071.6	5031425E22Rik	4.89E+00
gene29179	15374	NC_000077.6	Hn1	4.89E+00
gene28144	276846	NC_000077.6	Pigs	4.89E+00
gene35169	668450	NC_000080.6	Gm9174	4.88E+00
gene10386	18221	NC_000070.6	Nudc	4.88E+00
gene34052	74427	NC_000080.6	Eaf1	4.88E+00
gene26469	19344	NC_000076.6	Rab5b	4.88E+00
gene30537	217718	NC_000078.6	Nek9	4.88E+00
gene36588	17133	NC_000081.6	Maff	4.88E+00
gene18719	1E+08	NC_000073.6	Gm19204	4.88E+00
gene18153	621446	NC_000073.6	Gm6226	4.88E+00
gene23557	72278	NC_000075.6	Ccpg1	4.88E+00
gene41905	67689	NC_000085.6	Aldh3b1	4.87E+00
gene28076	1E+08	NC_000077.6	Gm18373	4.87E+00
gene26484	14533	NC_000076.6	Bloc1s1	4.87E+00
gene33042	12626	NC_000079.6	Cetn3	4.87E+00
gene1183	108679	NC_000067.6	Cops8	4.87E+00
gene35084	12177	NC_000080.6	Bnip3l	4.87E+00
gene24015	75686	NC_000075.6	Nudt16	4.86E+00
gene7690	66641	NC_000069.6	Sike1	4.86E+00
gene35976	22631	NC_000081.6	Ywhaz	4.86E+00
gene32230	13614	NC_000079.6	Edn1	4.86E+00
gene431	16182	NC_000067.6	Il18r1	4.85E+00
gene35797	66629	NC_000081.6	Golph3	4.85E+00
gene8718	665298	NC_000070.6	Gm11942	4.85E+00
gene5777	228866	NC_000068.7	Pcif1	4.85E+00
gene21182	19656	NC_000074.6	Rbmxl1	4.85E+00
gene5622	53619	NC_000068.7	Blcap	4.84E+00
gene17961	56632	NC_000073.6	Sphk2	4.84E+00
gene41449	240327	NC_000084.6	Gm4951	4.84E+00
gene25176	=Gene;ger	NC_000076.6	LOC108167812	4.84E+00
gene7805	ene=LOC1	NC_000069.6	LOC102640327	4.84E+00
gene26793	66249	NC_000077.6	Pno1	4.84E+00
gene2313	15950	NC_000067.6	Ifi203	4.84E+00
gene6188	66314	NC_000068.7	Tpd52l2	4.83E+00
gene19094	1E+08	NC_000073.6	Mrps36-ps2	4.83E+00
gene3292	72323	NC_000068.7	Asb6	4.83E+00
gene27894	382536	NC_000077.6	Gm12321	4.82E+00
gene3213	118451	NC_000068.7	Mrps2	4.82E+00
gene1995	14455	NC_000067.6	Gas5	4.82E+00
gene23447	623488	NC_000075.6	Ppp1r2-ps4	4.82E+00
gene39255	70083	NC_000083.6	Metrn	4.82E+00
gene42019	81601	NC_000085.6	Kat5	4.82E+00
gene12717	330173	NC_000071.6	2610524H06Rik	4.82E+00
gene4868	66606	NC_000068.7	Lrrc57	4.82E+00
gene17192	1E+08	NC_000073.6	Gm15495	4.81E+00

gene586	98267	NC_000067.6	Stk17b	4.81E+00
gene9352	1E+08	NC_000070.6	Gm11405	4.81E+00
gene11367	64295	NC_000071.6	Tmub1	4.81E+00
gene42004	1E+08	NC_000085.6	Gm25432	4.81E+00
gene28155	67811	NC_000077.6	Poldip2	4.80E+00
gene16904	69731	NC_000073.6	Gemin7	4.80E+00
gene22179	67892	NC_000074.6	Coa6	4.80E+00
gene41079	15200	NC_000084.6	Hbegf	4.80E+00
gene8929	242406	NC_000070.6	Rgp1	4.80E+00
gene18003	14884	NC_000073.6	Gtf2h1	4.80E+00
gene11585	68366	NC_000071.6	Tmem129	4.79E+00
gene20086	330695	NC_000074.6	Ctxn1	4.79E+00
gene37057	74351	NC_000081.6	Ddx23	4.79E+00
gene5580	170791	NC_000068.7	Rbm39	4.79E+00
gene18458	140481	NC_000073.6	Man2a2	4.79E+00
gene28403	22344	NC_000077.6	Vezf1	4.79E+00
gene28807	67998	NC_000077.6	Fam134c	4.79E+00
gene13794	545829	NC_000072.6	Gm5874	4.79E+00
gene21352	65114	NC_000074.6	Vps35	4.79E+00
gene5255	81910	NC_000068.7	Rrbp1	4.78E+00
gene39779	66136	NC_000083.6	Znrd1	4.78E+00
gene10135	230734	NC_000070.6	Yrdc	4.78E+00
gene28119	14252	NC_000077.6	Flot2	4.78E+00
gene3927	72421	NC_000068.7	Ttc30b	4.78E+00
gene29166	68572	NC_000077.6	Ict1	4.77E+00
gene9953	19268	NC_000070.6	Ptprf	4.77E+00
gene5799	381406	NC_000068.7	Trp53rka	4.76E+00
gene36679	20826	NC_000081.6	Nhp2l1	4.76E+00
gene26989	1E+08	NC_000077.6	Gm18743	4.76E+00
gene30086	26443	NC_000078.6	Psma6	4.76E+00
gene38905	1E+08	NC_000083.6	Gm3507	4.76E+00
gene36559	106039	NC_000081.6	Gga1	4.75E+00
gene22267	11796	NC_000075.6	Birc3	4.75E+00
gene36179	67819	NC_000081.6	Derl1	4.75E+00
gene40384	383295	NC_000083.6	Ypel5	4.75E+00
gene17290	1E+08	NC_000073.6	Gm10651	4.75E+00
gene28840	80981	NC_000077.6	Arl4d	4.74E+00
gene26667	104479	NC_000077.6	Ccdc117	4.74E+00
gene33751	432822	NC_000080.6	Gm5457	4.74E+00
gene24031	77305	NC_000075.6	Wdr82	4.74E+00
gene35457	50789	NC_000080.6	Fbxl3	4.74E+00
gene17247	19342	NC_000073.6	Rab4b	4.74E+00
gene11997	68939	NC_000071.6	Rasl11b	4.73E+00
gene3489	67466	NC_000068.7	Pdcl	4.73E+00
gene28578	80280	NC_000077.6	Cdk5rap3	4.73E+00
gene35177	72549	NC_000080.6	Reep4	4.73E+00
gene27192	667219	NC_000077.6	Gm12186	4.73E+00
gene11525	13667	NC_000071.6	Eif2b4	4.73E+00

gene1022	622958	NC_000067.6	Gm6374	4.73E+00
gene25702	14672	NC_000076.6	Gna11	4.72E+00
gene10176	27096	NC_000070.6	Trappc3	4.72E+00
gene37424	66409	NC_000082.6	Rsl1d1	4.72E+00
gene26457	103284	NC_000076.6	Zc3h10	4.71E+00
gene34777	105663	NC_000080.6	Thtpa	4.71E+00
gene14045	243771	NC_000072.6	Parp12	4.71E+00
gene42716	15242	NC_000085.6	Hhex	4.71E+00
gene27323	15525	NC_000077.6	Hspa4	4.71E+00
gene7432	53970	NC_000069.6	Rfx5	4.71E+00
gene14913	78108	NC_000072.6	Particl	4.71E+00
gene31577	19166	NC_000079.6	Psma2	4.70E+00
gene11019	66448	NC_000070.6	Mrpl20	4.70E+00
gene30917	1E+08	NC_000078.6	Gm15636	4.70E+00
gene41197	65113	NC_000084.6	Ndfip1	4.70E+00
gene11372	27407	NC_000071.6	Abcf2	4.70E+00
gene40023	53414	NC_000083.6	Bysl	4.69E+00
gene41094	67936	NC_000084.6	Wdr55	4.69E+00
gene917	68818	NC_000067.6	Zfand2b	4.69E+00
gene34727	13135	NC_000080.6	Dad1	4.69E+00
gene37926	1E+08	NC_000082.6	Gm15564	4.69E+00
gene37605	18100	NC_000082.6	Mrpl40	4.69E+00
gene4942	18457	NC_000068.7	Bloc1s6	4.68E+00
gene13524	24109	NC_000071.6	Ubl3	4.68E+00
gene40308	19328	NC_000083.6	Rab12	4.68E+00
gene3177	20931	NC_000068.7	Surf2	4.68E+00
gene2206	66052	NC_000067.6	Sdhc	4.67E+00
gene19091	76932	NC_000073.6	Arfip2	4.67E+00
gene10040	56222	NC_000070.6	Cited4	4.67E+00
gene30772	=Gene;ger	NC_000078.6	LOC102631912	4.67E+00
gene15719	16768	NC_000072.6	Lag3	4.66E+00
gene42107	108101	NC_000085.6	Fermt3	4.66E+00
gene27300	52626	NC_000077.6	Cdkn2aipnl	4.66E+00
gene33720	68263	NC_000080.6	Pdhb	4.66E+00
gene15455	67767	NC_000072.6	Jagn1	4.66E+00
gene10857	670864	NC_000070.6	Gm9506	4.66E+00
gene2476	=Gene;ger	NC_000067.6	LOC108167751	4.65E+00
gene34296	67089	NC_000080.6	Psmc6	4.65E+00
gene9009	67628	NC_000070.6	Anp32b	4.65E+00
gene39383	66989	NC_000083.6	Kctd20	4.65E+00
gene17887	15469	NC_000073.6	Prmt1	4.65E+00
gene28794	80860	NC_000077.6	Ghdc	4.65E+00
gene19582	233900	NC_000073.6	Rnf40	4.64E+00
gene27941	53313	NC_000077.6	Atp2a3	4.64E+00
gene19730	66165	NC_000073.6	Bccip	4.64E+00
gene6189	13002	NC_000068.7	Dnajc5	4.64E+00
gene10901	74155	NC_000070.6	Errfi1	4.64E+00
gene7764	67171	NC_000069.6	Dram2	4.63E+00

gene11604	19822	NC_000071.6	Rnf4	4.63E+00
gene14763	385120	NC_000072.6	Igkv4-70	4.63E+00
gene36878	52683	NC_000081.6	Ncaph2	4.62E+00
gene29532	382567	NC_000078.6	Gm5182	4.62E+00
gene9101	66536	NC_000070.6	Nipsnap3b	4.62E+00
gene5061	329514	NC_000068.7	Il1bos	4.62E+00
gene39199	68636	NC_000083.6	Fahd1	4.61E+00
gene3262	227695	NC_000068.7	D2Wsu81e	4.61E+00
gene23236	12988	NC_000075.6	Csk	4.61E+00
gene24552	215751	NC_000076.6	Ginm1	4.61E+00
gene42061	56327	NC_000085.6	Arl2	4.61E+00
gene36561	20401	NC_000081.6	Sh3bp1	4.61E+00
gene36413	66656	NC_000081.6	Eef1d	4.61E+00
gene17476	20266	NC_000073.6	Scn1b	4.61E+00
gene41727	639576	NC_000084.6	Gm7273	4.60E+00
gene21309	66830	NC_000074.6	Nacc1	4.60E+00
gene26893	1E+08	NC_000077.6	Gm12060	4.60E+00
gene2423	15078	NC_000067.6	H3f3a	4.60E+00
gene11709	1E+08	NC_000071.6	Gm2810	4.60E+00
gene27798	20020	NC_000077.6	Polr2a	4.60E+00
gene25679	57267	NC_000076.6	Apba3	4.60E+00
gene39521	328801	NC_000083.6	Zfp414	4.60E+00
gene35631	110168	NC_000080.6	Gpr18	4.60E+00
gene29694	11491	NC_000078.6	Adam17	4.59E+00
gene25829	14074	NC_000076.6	Fabp3-ps1	4.59E+00
gene3983	66861	NC_000068.7	Dnajc10	4.59E+00
gene39172	319259	NC_000083.6	Bricd5	4.59E+00
gene35083	672614	NC_000080.6	Gm9570	4.59E+00
gene24311	21813	NC_000075.6	Tgfbr2	4.59E+00
gene28635	19921	NC_000077.6	Rpl19	4.59E+00
gene22854	235281	NC_000075.6	Scn3b	4.59E+00
gene41918	225884	NC_000085.6	BC021614	4.59E+00
gene28418	74617	NC_000077.6	Scpep1	4.59E+00
gene4022	12388	NC_000068.7	Ctnnd1	4.58E+00
gene31035	13424	NC_000078.6	Dync1h1	4.58E+00
gene6002	228961	NC_000068.7	Npepl1	4.58E+00
gene41467	15531	NC_000084.6	Ndst1	4.58E+00
gene20047	140482	NC_000074.6	Zfp358	4.58E+00
gene28020	104662	NC_000077.6	Tsr1	4.57E+00
gene41074	19290	NC_000084.6	Pura	4.57E+00
gene3169	669582	NC_000068.7	Gm13359	4.57E+00
gene27083	52653	NC_000077.6	Nudcd2	4.57E+00
gene28061	76566	NC_000077.6	Fam101b	4.57E+00
gene15117	17119	NC_000072.6	Mxd1	4.57E+00
gene4830	228545	NC_000068.7	Vps18	4.57E+00
gene15389	58799	NC_000072.6	Crbn	4.56E+00
gene13643	209086	NC_000072.6	Samd9l	4.56E+00
gene28662	66612	NC_000077.6	Ormdl3	4.56E+00

gene34041	64652	NC_000080.6	Nisch	4.56E+00
gene26704	246177	NC_000077.6	Myo1g	4.55E+00
gene42043	12333	NC_000085.6	Capn1	4.55E+00
gene39271	14755	NC_000083.6	Pigq	4.55E+00
gene39251	68977	NC_000083.6	Haghl	4.55E+00
gene36480	223665	NC_000081.6	C030006K11Rik	4.55E+00
gene10963	72946	NC_000070.6	Lrrc47	4.55E+00
gene1840	117198	NC_000067.6	Ivns1abp	4.55E+00
gene20498	244373	NC_000074.6	Erlin2	4.55E+00
gene5866	19246	NC_000068.7	Ptpn1	4.55E+00
gene10245	19646	NC_000070.6	Rbbp4	4.55E+00
gene23804	434428	NC_000075.6	Gm5620	4.54E+00
gene10473	27224	NC_000070.6	Tceb3	4.54E+00
gene12644	54723	NC_000071.6	Tfip11	4.54E+00
gene6382	27357	NC_000069.6	Gyg	4.54E+00
gene39560	414076	NC_000083.6	BC051537	4.54E+00
gene17611	110959	NC_000073.6	Nudt19	4.54E+00
gene29208	70450	NC_000077.6	Unc13d	4.54E+00
gene25624	66594	NC_000076.6	Uqcr11	4.54E+00
gene13467	74132	NC_000071.6	Rnf6	4.54E+00
gene37211	209039	NC_000081.6	Tns2	4.54E+00
gene36599	1E+08	NC_000081.6	Gm30877	4.53E+00
gene33971	211948	NC_000080.6	Pde12	4.53E+00
gene7200	229524	NC_000069.6	Msto1	4.53E+00
gene19605	233908	NC_000073.6	Fus	4.52E+00
gene38265	67609	NC_000082.6	4930453N24Rik	4.52E+00
gene3633	1E+08	NC_000068.7	Gm38481	4.52E+00
gene39127	76498	NC_000083.6	Paqr4	4.52E+00
gene285	633498	NC_000067.6	Gm7114	4.52E+00
gene17136	210146	NC_000073.6	Irgq	4.52E+00
gene9095	632982	NC_000070.6	Cct3-ps1	4.52E+00
gene18778	66624	NC_000073.6	Spcs2	4.51E+00
gene41835	225791	NC_000084.6	Zadh2	4.51E+00
gene27793	80886	NC_000077.6	Senp3	4.51E+00
gene37279	105855	NC_000081.6	Nckap1l	4.51E+00
gene9302	21898	NC_000070.6	Tlr4	4.51E+00
gene18446	269951	NC_000073.6	Idh2	4.50E+00
gene35382	328451	NC_000080.6	Gm5088	4.50E+00
gene26576	236604	NC_000077.6	Pisd-ps1	4.50E+00
gene29855	73389	NC_000078.6	Hbp1	4.50E+00
gene36532	12984	NC_000081.6	Csf2rb2	4.50E+00
gene1263	110611	NC_000067.6	Hdlbp	4.50E+00
gene15540	621462	NC_000072.6	Gm6227	4.50E+00
gene6475	20482	NC_000069.6	Skil	4.50E+00
gene11956	27784	NC_000071.6	Commd8	4.49E+00
gene40585	56724	NC_000083.6	Cript	4.49E+00
gene42900	226154	NC_000085.6	Lzts2	4.49E+00
gene27596	23939	NC_000077.6	Mapk7	4.49E+00

gene32501	26385	NC_000079.6	Grk6	4.49E+00
gene41052	17184	NC_000084.6	Matr3	4.49E+00
gene20476	14272	NC_000074.6	Fnta	4.48E+00
gene25672	13144	NC_000076.6	Dapk3	4.48E+00
gene16001	67755	NC_000072.6	Ddx47	4.48E+00
gene34796	268749	NC_000080.6	Rnf31	4.48E+00
gene30353	15512	NC_000078.6	Hspa2	4.48E+00
gene25646	20222	NC_000076.6	Sf3a2	4.48E+00
gene28484	12643	NC_000077.6	Chad	4.47E+00
gene3216	19730	NC_000068.7	Ralgds	4.47E+00
gene26431	66701	NC_000076.6	Spryd4	4.47E+00
gene25071	14727	NC_000076.6	Lilr4b	4.47E+00
gene3492	241322	NC_000068.7	Zbtb6	4.47E+00
gene37002	105827	NC_000081.6	Amigo2	4.46E+00
gene25511	28295	NC_000076.6	D10Jhu81e	4.46E+00
gene28778	104112	NC_000077.6	Acly	4.46E+00
gene23436	68250	NC_000075.6	Fam96a	4.46E+00
gene36207	268809	NC_000081.6	Gm5045	4.46E+00
gene40631	1E+08	NC_000083.6	Gm10184	4.46E+00
gene26842	216551	NC_000077.6	Lgalsl	4.46E+00
gene8808	15502	NC_000070.6	Dnaja1	4.45E+00
gene40210	66400	NC_000083.6	Alkbh7	4.45E+00
gene125	269105	NC_000067.6	Gm5048	4.45E+00
gene32732	218301	NC_000079.6	Gm4811	4.45E+00
gene39263	214952	NC_000083.6	Rhot2	4.44E+00
gene9745	17149	NC_000070.6	Magoh	4.44E+00
gene25531	71709	NC_000076.6	Syde1	4.44E+00
gene38037	72117	NC_000082.6	Naa50	4.44E+00
gene9252	20529	NC_000070.6	Slc31a1	4.44E+00
gene34728	105501	NC_000080.6	Abhd4	4.43E+00
gene7235	54124	NC_000069.6	Cks1b	4.43E+00
gene40191	74142	NC_000083.6	Lonp1	4.43E+00
gene28808	103733	NC_000077.6	Tubg1	4.42E+00
gene31641	432730	NC_000079.6	Gm5446	4.42E+00
gene26393	108037	NC_000076.6	Shmt2	4.42E+00
gene37589	12846	NC_000082.6	Comt	4.41E+00
gene39539	19732	NC_000083.6	Rgl2	4.41E+00
gene24964	171580	NC_000076.6	Mical1	4.41E+00
gene347	214855	NC_000067.6	Arid5a	4.41E+00
gene9010	94181	NC_000070.6	Nans	4.41E+00
gene19913	67186	NC_000073.6	Rplp2	4.41E+00
gene42575	67072	NC_000085.6	Cdc37l1	4.40E+00
gene5685	497655	NC_000068.7	Gm11451	4.40E+00
gene12020	606516	NC_000071.6	Gm6051	4.39E+00
gene9796	12580	NC_000070.6	Cdkn2c	4.39E+00
gene3801	64406	NC_000068.7	Sp5	4.39E+00
gene15599	11973	NC_000072.6	Atp6v1e1	4.39E+00
gene12154	231413	NC_000071.6	Grsf1	4.39E+00

gene15704	13498	NC_000072.6	Atn1	4.39E+00
gene42126	20168	NC_000085.6	Rtn3	4.38E+00
gene12043	66661	NC_000071.6	Srp72	4.38E+00
gene24347	546165	NC_000075.6	Gm10608	4.38E+00
gene37438	1E+08	NC_000082.6	Gm19571	4.38E+00
gene39948	63959	NC_000083.6	Slc29a1	4.38E+00
gene30596	66354	NC_000078.6	Snw1	4.37E+00
gene11234	nit 5 pseud	NC_000071.6	LOC100534357	4.37E+00
gene15017	13191	NC_000072.6	Dctn1	4.37E+00
gene8721	56228	NC_000070.6	Ube2j1	4.37E+00
gene17363	77254	NC_000073.6	Yif1b	4.37E+00
gene25423	22364	NC_000076.6	Vpreb3	4.36E+00
gene26952	68585	NC_000077.6	Rtn4	4.36E+00
gene35401	360130	NC_000080.6	Gnb1-ps2	4.36E+00
gene35610	76108	NC_000080.6	Rap2a	4.36E+00
gene21746	1E+08	NC_000074.6	Rps18-ps3	4.36E+00
gene13210	83701	NC_000071.6	Srrt	4.36E+00
gene35494	24064	NC_000080.6	Spry2	4.36E+00
gene23550	17999	NC_000075.6	Nedd4	4.36E+00
gene23419	27965	NC_000075.6	Spg21	4.36E+00
gene7456	75007	NC_000069.6	Fam63a	4.36E+00
gene6217	1E+08	NC_000069.6	Gm8775	4.35E+00
gene6555	12428	NC_000069.6	Ccna2	4.35E+00
gene40001	72029	NC_000083.6	Cnpy3	4.35E+00
gene13686	20422	NC_000072.6	Shfm1	4.35E+00
gene33653	1E+08	NC_000080.6	Gm17046	4.34E+00
gene25891	1E+08	NC_000076.6	Gm18705	4.34E+00
gene6496	66046	NC_000069.6	Ndufb5	4.34E+00
gene24324	27215	NC_000075.6	Azi2	4.34E+00
gene17310	20924	NC_000073.6	Supt5	4.34E+00
gene11275	12491	NC_000071.6	Cd36	4.34E+00
gene33502	13723	NC_000079.6	Emb	4.33E+00
gene43044	1E+08	NC_000085.6	Gm31595	4.33E+00
gene28250	76392	NC_000077.6	Slfn5os	4.33E+00
gene32519	69672	NC_000079.6	Txndc15	4.33E+00
gene42746	667587	NC_000085.6	Gm8717	4.33E+00
gene21725	12550	NC_000074.6	Cdh1	4.33E+00
gene22321	14345	NC_000075.6	Fut4	4.33E+00
gene36127	1E+08	NC_000081.6	Gm7543	4.33E+00
gene10328	664903	NC_000070.6	Rps15a-ps4	4.33E+00
gene33883	69156	NC_000080.6	Comtd1	4.33E+00
gene2769	791411	NC_000068.7	Gm13199	4.32E+00
gene22893	235293	NC_000075.6	Sc5d	4.32E+00
gene36109	665146	NC_000081.6	Gm7517	4.32E+00
gene22020	66965	NC_000074.6	Ctu2	4.32E+00
gene19348	267019	NC_000073.6	Rps15a	4.32E+00
gene38830	ene=LOC1	NC_000083.6	LOC108168333	4.32E+00
gene10195	71514	NC_000070.6	Sfpq	4.32E+00

gene8848	16157	NC_000070.6	Il11ra1	4.32E+00
gene35323	211255	NC_000080.6	Kbtbd7	4.32E+00
gene13303	80290	NC_000071.6	Gpr146	4.31E+00
gene36467	94230	NC_000081.6	Cpsf1	4.31E+00
gene13244	231805	NC_000071.6	Pilra	4.31E+00
gene18641	14366	NC_000073.6	Fzd4	4.31E+00
gene10103	68050	NC_000070.6	Akirin1	4.31E+00
gene38036	11964	NC_000082.6	Atp6v1a	4.31E+00
gene29319	27029	NC_000077.6	Sgsh	4.31E+00
gene28788	80861	NC_000077.6	Dhx58	4.31E+00
gene17208	52132	NC_000073.6	Ccdc97	4.31E+00
gene38660	224487	NC_000083.6	Gm4829	4.30E+00
gene9608	230484	NC_000070.6	Usp1	4.30E+00
gene39220	68327	NC_000083.6	Tsr3	4.30E+00
gene17865	71960	NC_000073.6	Myh14	4.30E+00
gene30122	15375	NC_000078.6	Foxa1	4.30E+00
gene38761	320111	NC_000083.6	Prr18	4.30E+00
gene21342	330817	NC_000074.6	Dhps	4.30E+00
gene5745	ene;gene=L	NC_000068.7	LOC102638515	4.30E+00
gene17338	64383	NC_000073.6	Sirt2	4.30E+00
gene27771	216848	NC_000077.6	Chd3	4.30E+00
gene4027	228136	NC_000068.7	Zdhhc5	4.29E+00
gene13505	1E+08	NC_000071.6	Gm29778	4.29E+00
gene10001	69216	NC_000070.6	Svbp	4.29E+00
gene25963	628119	NC_000076.6	Anapc15-ps	4.29E+00
gene14906	68364	NC_000072.6	0610030E20Rik	4.29E+00
gene39544	224705	NC_000083.6	Vps52	4.29E+00
gene10432	74777	NC_000070.6	Sepn1	4.29E+00
gene23155	664850	NC_000075.6	Gm7368	4.29E+00
gene29340	72055	NC_000077.6	Slc38a10	4.28E+00
gene13307	100494	NC_000071.6	Zfand2a	4.28E+00
gene41427	1E+08	NC_000084.6	Gm18740	4.28E+00
gene14837	667881	NC_000072.6	Igkv6-14	4.28E+00
gene37269	56447	NC_000081.6	Copz1	4.28E+00
gene7972	99480	NC_000069.6	Dnttip2	4.28E+00
gene39444	78330	NC_000083.6	Ndufv3	4.28E+00
gene3256	66220	NC_000068.7	Zdhhc12	4.28E+00
gene33387	668129	NC_000079.6	Gm8990	4.28E+00
gene26620	71673	NC_000077.6	Rnf215	4.27E+00
gene40901	59057	NC_000084.6	Zfp24	4.27E+00
gene23980	20818	NC_000075.6	Srprb	4.27E+00
gene19909	213350	NC_000073.6	Pddc1	4.27E+00
gene5642	16803	NC_000068.7	Lbp	4.26E+00
gene42610	546723	NC_000085.6	Ppp1r2-ps3	4.26E+00
gene5951	67017	NC_000068.7	Fam210b	4.26E+00
gene36538	246221	NC_000081.6	Mpst	4.26E+00
gene40671	621666	NC_000084.6	Gm6248	4.26E+00
gene23217	74211	NC_000075.6	1700017B05Rik	4.26E+00

gene11474	1E+08	NC_000071.6	Gm1969	4.26E+00
gene39217	214489	NC_000083.6	BC003965	4.26E+00
gene932	69080	NC_000067.6	Gmppa	4.26E+00
gene23038	434401	NC_000075.6	Gm5616	4.26E+00
gene10535	68350	NC_000070.6	Mul1	4.25E+00
gene13799	16345	NC_000072.6	Eif3s6-ps4	4.25E+00
gene30527	78920	NC_000078.6	Dlst	4.25E+00
gene40950	666094	NC_000084.6	Gm7926	4.24E+00
gene10350	68598	NC_000070.6	Dnajc8	4.24E+00
gene27147	66213	NC_000077.6	Med7	4.24E+00
gene37395	26425	NC_000082.6	Nubp1	4.24E+00
gene15531	213391	NC_000072.6	Rassf4	4.24E+00
gene6778	52245	NC_000069.6	Commd2	4.24E+00
gene32605	13996	NC_000079.6	Etohd2	4.24E+00
gene18810	19346	NC_000073.6	Rab6a	4.23E+00
gene33470	170625	NC_000079.6	Snx18	4.23E+00
gene4827	20732	NC_000068.7	Spint1	4.23E+00
gene15236	665685	NC_000072.6	Ppp1r2-ps2	4.22E+00
gene42191	67160	NC_000085.6	Eef1g	4.22E+00
gene42283	94063	NC_000085.6	Mrpl16	4.22E+00
gene23186	26611	NC_000075.6	Rcn2	4.22E+00
gene34242	13586	NC_000080.6	Ear1	4.22E+00
gene31939	15216	NC_000079.6	Hfe	4.22E+00
gene39876	gene=LOC1	NC_000083.6	LOC101056014	4.22E+00
gene32730	432767	NC_000079.6	Eif1-ps2	4.22E+00
gene12767	19079	NC_000071.6	Prkab1	4.21E+00
gene4426	228359	NC_000068.7	Arhgap1	4.21E+00
gene19424	59052	NC_000073.6	Mettl9	4.21E+00
gene26206	17246	NC_000076.6	Mdm2	4.20E+00
gene6248	620966	NC_000069.6	Gm6194	4.20E+00
gene13489	66596	NC_000071.6	Gtf3a	4.20E+00
gene10186	230757	NC_000070.6	5730409E04Rik	4.20E+00
gene5121	69596	NC_000068.7	Ap5s1	4.19E+00
gene16292	243813	NC_000073.6	Leng9	4.19E+00
gene16667	232887	NC_000073.6	Gm4879	4.19E+00
gene32247	12522	NC_000079.6	Cd83	4.19E+00
gene23339	ene;gene=L	NC_000075.6	LOC102638888	4.18E+00
gene25662	52551	NC_000076.6	Sgta	4.18E+00
gene37844	68092	NC_000082.6	Ncbp2	4.18E+00
gene12352	11991	NC_000071.6	Hnrnpd	4.18E+00
gene12416	114664	NC_000071.6	Hsd17b11	4.17E+00
gene42923	18150	NC_000085.6	Npm3	4.17E+00
gene21311	17095	NC_000074.6	Lyl1	4.17E+00
gene39610	108077	NC_000083.6	Skiv2l	4.17E+00
gene28399	574528	NC_000077.6	Gm11507	4.16E+00
gene42267	60361	NC_000085.6	Ms4a4b	4.16E+00
gene332	666261	NC_000067.6	Gm8009	4.16E+00
gene28530	237928	NC_000077.6	Phospho1	4.16E+00

gene15841	80782	NC_000072.6	Klrb1b	4.16E+00
gene28944	11841	NC_000077.6	Arf2	4.16E+00
gene27921	327959	NC_000077.6	Xaf1	4.15E+00
gene8801	230075	NC_000070.6	Ndufb6	4.15E+00
gene20824	664948	NC_000074.6	Gm7419	4.15E+00
gene3775	667443	NC_000068.7	Gm13612	4.15E+00
gene25571	83554	NC_000076.6	Fstl3	4.15E+00
gene27531	20787	NC_000077.6	Srebf1	4.15E+00
gene32855	408058	NC_000079.6	BC048507	4.14E+00
gene28594	73174	NC_000077.6	Tbkbp1	4.14E+00
gene25240	56200	NC_000076.6	Ddx21	4.14E+00
gene21613	234663	NC_000074.6	Dync1li2	4.14E+00
gene13266	21343	NC_000071.6	Taf6	4.14E+00
gene26583	13494	NC_000077.6	Drg1	4.14E+00
gene2614	1E+08	NC_000067.6	Gm2272	4.14E+00
gene793	11363	NC_000067.6	Acadl	4.13E+00
gene22019	68718	NC_000074.6	Rnf166	4.13E+00
gene23628	14860	NC_000075.6	Gsta4	4.13E+00
gene11482	97212	NC_000071.6	Hadha	4.13E+00
gene29245	107817	NC_000077.6	Jmjd6	4.13E+00
gene820	14268	NC_000067.6	Fn1	4.13E+00
gene8169	51788	NC_000069.6	H2afz	4.12E+00
gene41906	1.1E+08	NC_000085.6	Gm42059	4.12E+00
gene7231	22724	NC_000069.6	Zbtb7b	4.12E+00
gene42944	75579	NC_000085.6	2310034G01Rik	4.12E+00
gene17179	71722	NC_000073.6	Cic	4.12E+00
gene7222	13636	NC_000069.6	Efna1	4.12E+00
gene34494	268741	NC_000080.6	Tox4	4.12E+00
gene40209	53895	NC_000083.6	Clpp	4.12E+00
gene24825	68031	NC_000076.6	Rnf146	4.12E+00
gene8904	269523	NC_000070.6	Vcp	4.12E+00
gene41186	75599	NC_000084.6	Pcdh1	4.11E+00
gene42044	1E+08	NC_000085.6	Gm10814	4.11E+00
gene19573	14123	NC_000073.6	Fbrs	4.11E+00
gene5078	67134	NC_000068.7	Nop56	4.10E+00
gene36743	1.1E+08	NC_000081.6	Gm41368	4.10E+00
gene17209	232989	NC_000073.6	Hnrnpul1	4.10E+00
gene34487	15381	NC_000080.6	Hnrnpc	4.10E+00
gene42708	69104	NC_000085.6	March5	4.10E+00
gene16341	66056	NC_000073.6	Zfp524	4.10E+00
gene26451	68094	NC_000076.6	Smarcc2	4.10E+00
gene37826	66849	NC_000082.6	Ppp1r2	4.09E+00
gene3561	433424	NC_000068.7	Zeb2os	4.09E+00
gene17187	16890	NC_000073.6	Lipe	4.09E+00
gene24095	20350	NC_000075.6	Sema3f	4.09E+00
gene26191	64050	NC_000076.6	Yeats4	4.09E+00
gene17133	18793	NC_000073.6	Plaur	4.09E+00
gene27288	212706	NC_000077.6	N4bp3	4.09E+00

gene27187	620913	NC_000077.6	Gm12185	4.09E+00
gene4502	11800	NC_000068.7	Api5	4.09E+00
gene29843	13382	NC_000078.6	Dld	4.08E+00
gene4654	67606	NC_000068.7	Fibin	4.08E+00
gene40782	66878	NC_000084.6	Riok3	4.08E+00
gene13390	66913	NC_000071.6	Kdelr2	4.08E+00
gene37302	74120	NC_000082.6	Zfp263	4.08E+00
gene36478	223664	NC_000081.6	Lrrc14	4.08E+00
gene35230	629578	NC_000080.6	Cbx3-ps6	4.08E+00
gene29389	19719	NC_000077.6	Rfng	4.07E+00
gene29303	12418	NC_000077.6	Cbx4	4.07E+00
gene19428	668556	NC_000073.6	Gm9240	4.07E+00
gene39527	80880	NC_000083.6	Kank3	4.07E+00
gene18811	27276	NC_000073.6	Plekhb1	4.07E+00
gene25865	104303	NC_000076.6	Arl1	4.06E+00
gene7540	668500	NC_000069.6	Gm9207	4.06E+00
gene13272	231807	NC_000071.6	BC037034	4.06E+00
gene32413	13610	NC_000079.6	S1pr3	4.06E+00
gene36565	97961	NC_000081.6	Nol12	4.06E+00
gene39523	76936	NC_000083.6	Hnrnpm	4.05E+00
gene28295	676191	NC_000077.6	Gm11434	4.05E+00
gene12372	15442	NC_000071.6	Hpse	4.05E+00
gene7194	19769	NC_000069.6	Rit1	4.05E+00
gene20503	75767	NC_000074.6	Rab11fip1	4.05E+00
gene16324	664968	NC_000073.6	Tmem238	4.04E+00
gene34905	219132	NC_000080.6	Phf11d	4.04E+00
gene18456	68695	NC_000073.6	Hddc3	4.04E+00
gene36229	320469	NC_000081.6	9930014A18Rik	4.04E+00
gene10985	230979	NC_000070.6	Tnfrsf14	4.04E+00
gene7822	20661	NC_000069.6	Sort1	4.03E+00
gene548	109019	NC_000067.6	Nabp1	4.03E+00
gene33504	623365	NC_000079.6	Gm6421	4.03E+00
gene42242	28000	NC_000085.6	Prpf19	4.03E+00
gene19620	233912	NC_000073.6	Armc5	4.03E+00
gene13551	11690	NC_000071.6	Alox5ap	4.03E+00
gene10354	100340	NC_000070.6	Smpdl3b	4.02E+00
gene25203	18646	NC_000076.6	Prf1	4.02E+00
gene23252	26968	NC_000075.6	Islr	4.02E+00
gene17263	170742	NC_000073.6	Sertad3	4.02E+00
gene26693	ene;gene=L	NC_000077.6	LOC108167883	4.02E+00
gene8189	11532	NC_000069.6	Adh5	4.02E+00
gene30376	230765	NC_000078.6	Gm4864	4.02E+00
gene3085	227618	NC_000068.7	Lrrc26	4.01E+00
gene37548	66863	NC_000082.6	Lztr1	4.01E+00
gene13214	13846	NC_000071.6	Ephb4	4.01E+00
gene10583	242721	NC_000070.6	Klhdc7a	4.01E+00
gene16125	75320	NC_000072.6	Etnk1	4.00E+00
gene33142	1E+08	NC_000079.6	Gm30155	4.00E+00

gene5458	14950	NC_000068.7	H13	4.00E+00
gene8142	319587	NC_000069.6	4930539J05Rik	4.00E+00
gene20995	26558	NC_000074.6	Homer3	4.00E+00
gene6321	12349	NC_000069.6	Car2	4.00E+00
gene11365	20535	NC_000071.6	Slc4a2	4.00E+00
gene6730	50706	NC_000069.6	Postn	3.99E+00
gene35680	106064	NC_000081.6	AW549877	3.99E+00
gene13366	666788	NC_000071.6	Gm15770	3.99E+00
gene8847	14430	NC_000070.6	Galt	3.99E+00
gene30678	20338	NC_000078.6	Sel1l	3.98E+00
gene38106	245195	NC_000082.6	Retnlg	3.98E+00
gene35968	68839	NC_000081.6	Ankrd46	3.98E+00
gene3412	15139	NC_000068.7	Hc	3.98E+00
gene23072	69631	NC_000075.6	Plet1os	3.98E+00
gene22562	235072	NC_000075.6	sep-07	3.98E+00
gene40383	1E+08	NC_000083.6	Gm19183	3.98E+00
gene11878	16599	NC_000071.6	Klf3	3.98E+00
gene23578	59046	NC_000075.6	Arpp19	3.98E+00
gene38527	15975	NC_000082.6	Ifnar1	3.98E+00
gene25858	212862	NC_000076.6	Chpt1	3.98E+00
gene21819	234734	NC_000074.6	Aars	3.98E+00
gene14342	243374	NC_000072.6	Gimap8	3.98E+00
gene852	16008	NC_000067.6	Igfbp2	3.97E+00
gene656	12633	NC_000067.6	Cflar	3.97E+00
gene10392	503610	NC_000070.6	Zdhhc18	3.97E+00
gene12920	59008	NC_000071.6	Anapc5	3.97E+00
gene36541	72844	NC_000081.6	Kctd17	3.97E+00
gene23074	19286	NC_000075.6	Pts	3.97E+00
gene3364	329384	NC_000068.7	Ptrh1	3.96E+00
gene25603	12696	NC_000076.6	Cirbp	3.96E+00
gene37744	625969	NC_000082.6	Gm6640	3.96E+00
gene17882	70300	NC_000073.6	Fuz	3.96E+00
gene13179	1E+08	NC_000071.6	Gm30408	3.96E+00
gene26679	18972	NC_000077.6	Pold2	3.96E+00
gene7791	229709	NC_000069.6	Ahcyl1	3.96E+00
gene7363	1E+08	NC_000069.6	Gm18432	3.95E+00
gene42039	104252	NC_000085.6	Cdc42ep2	3.95E+00
gene28053	19062	NC_000077.6	Inpp5k	3.95E+00
gene7657	12481	NC_000069.6	Cd2	3.95E+00
gene16151	16653	NC_000072.6	Kras	3.95E+00
gene83	26754	NC_000067.6	Cops5	3.95E+00
gene3404	30050	NC_000068.7	Fbxw2	3.95E+00
gene27497	67212	NC_000077.6	Mrpl55	3.95E+00
gene42055	81909	NC_000085.6	Zfpl1	3.95E+00
gene36766	68653	NC_000081.6	Samm50	3.94E+00
gene20464	71910	NC_000074.6	Plpp5	3.94E+00
gene26678	11568	NC_000077.6	Aebp1	3.94E+00
gene36629	66538	NC_000081.6	Rps19bp1	3.94E+00

gene42127	109168	NC_000085.6	Atl3	3.94E+00
gene15192	18844	NC_000072.6	Plxna1	3.94E+00
gene37545	67474	NC_000082.6	Snap29	3.94E+00
gene38167	52575	NC_000082.6	Trmt10c	3.94E+00
gene21702	20498	NC_000074.6	Slc12a4	3.94E+00
gene14626	68169	NC_000072.6	Ndnf	3.94E+00
gene34744	19173	NC_000080.6	Psmb5	3.93E+00
gene16304	232807	NC_000073.6	Ppp1r12c	3.93E+00
gene39433	21785	NC_000083.6	Tff2	3.93E+00
gene23465	665794	NC_000075.6	Gm7787	3.93E+00
gene9997	20525	NC_000070.6	Slc2a1	3.93E+00
gene16272	77582	NC_000073.6	Mboat7	3.93E+00
gene21113	11767	NC_000074.6	Ap1m1	3.92E+00
gene20152	12827	NC_000074.6	Col4a2	3.92E+00
gene19533	381921	NC_000073.6	Taok2	3.92E+00
gene2805	11949	NC_000068.7	Atp5c1	3.92E+00
gene3807	70231	NC_000068.7	Gorasp2	3.92E+00
gene9708	74754	NC_000070.6	Dhcr24	3.91E+00
gene25199	20397	NC_000076.6	Sgpl1	3.91E+00
gene26805	627716	NC_000077.6	Gm12020	3.91E+00
gene1559	1E+08	NC_000067.6	Gm28857	3.91E+00
gene21521	20312	NC_000074.6	Cx3cl1	3.91E+00
gene16429	12727	NC_000073.6	Clcn4	3.90E+00
gene42240	98170	NC_000085.6	Tmem132a	3.90E+00
gene15530	67255	NC_000072.6	Zfp422	3.90E+00
gene6154	76425	NC_000068.7	Gid8	3.90E+00
gene6628	70804	NC_000069.6	Pgrmc2	3.90E+00
gene40309	1.1E+08	NC_000083.6	Gm41608	3.90E+00
gene22941	71732	NC_000075.6	Vps11	3.90E+00
gene42702	53412	NC_000085.6	Ppp1r3c	3.90E+00
gene36769	64099	NC_000081.6	Parvg	3.89E+00
gene42455	20409	NC_000085.6	Ostf1	3.89E+00
gene23338	11737	NC_000075.6	Anp32a	3.89E+00
gene41250	666594	NC_000084.6	Gm8181	3.89E+00
gene7278	20196	NC_000069.6	S100a13	3.89E+00
gene10429	71904	NC_000070.6	Paqr7	3.89E+00
gene21338	320466	NC_000074.6	A230103J11Rik	3.89E+00
gene21779	71955	NC_000074.6	Ist1	3.89E+00
gene27578	26397	NC_000077.6	Map2k3	3.89E+00
gene644	12747	NC_000067.6	Clk1	3.89E+00
gene28918	57778	NC_000077.6	Fmnl1	3.88E+00
gene36732	73826	NC_000081.6	Poldip3	3.88E+00
gene14878	12526	NC_000072.6	Cd8b1	3.88E+00
gene39324	224640	NC_000083.6	Lemd2	3.88E+00
gene42188	=Gene;ger	NC_000085.6	LOC102640526	3.87E+00
gene20186	50770	NC_000074.6	Atp11a	3.87E+00
gene21651	71609	NC_000074.6	Tradd	3.87E+00
gene41056	381148	NC_000084.6	Prob1	3.87E+00

gene29360	15239	NC_000077.6	Hgs	3.87E+00
gene28633	217151	NC_000077.6	Arl5c	3.87E+00
gene18371	1E+08	NC_000073.6	Fam174b	3.87E+00
gene5573	1E+08	NC_000068.7	Gm14240	3.86E+00
gene5559	108687	NC_000068.7	Edem2	3.86E+00
gene4832	54485	NC_000068.7	Dll4	3.86E+00
gene12967	66627	NC_000071.6	Ogfod2	3.86E+00
gene30837	667997	NC_000078.6	Gm8918	3.85E+00
gene25661	106947	NC_000076.6	Slc39a3	3.85E+00
gene5057	1E+08	NC_000068.7	Gm14023	3.85E+00
gene42245	108673	NC_000085.6	Ccdc86	3.84E+00
gene31128	399566	NC_000078.6	Btbd6	3.84E+00
gene30595	380773	NC_000078.6	Slirp	3.84E+00
gene27101	193116	NC_000077.6	Slu7	3.84E+00
gene38275	68942	NC_000082.6	Chmp2b	3.84E+00
gene25448	12834	NC_000076.6	Col6a2	3.84E+00
gene24261	18571	NC_000075.6	Pdcd6ip	3.84E+00
gene19242	233726	NC_000073.6	Ipo7	3.84E+00
gene3330	22245	NC_000068.7	Uck1	3.84E+00
gene17394	26466	NC_000073.6	Zfp260	3.84E+00
gene13068	14004	NC_000071.6	Chchd2	3.83E+00
gene2444	226757	NC_000067.6	Wdr26	3.83E+00
gene39694	12305	NC_000083.6	Ddr1	3.83E+00
gene20540	67920	NC_000074.6	Mak16	3.83E+00
gene30295	66375	NC_000078.6	Dhrs7	3.83E+00
gene16907	19698	NC_000073.6	Relb	3.83E+00
gene35437	66200	NC_000080.6	Commd6	3.83E+00
gene39703	106582	NC_000083.6	Nrm	3.83E+00
gene13139	12054	NC_000071.6	Bcl7b	3.83E+00
gene10150	230738	NC_000070.6	Zc3h12a	3.83E+00
gene24225	17002	NC_000075.6	Ltf	3.83E+00
gene22181	619653	NC_000074.6	Gm6091	3.82E+00
gene28518	20747	NC_000077.6	Spop	3.82E+00
gene41967	75221	NC_000085.6	Dpp3	3.82E+00
gene11146	667767	NC_000071.6	Gm8802	3.82E+00
gene17178	13875	NC_000073.6	Erf	3.82E+00
gene42053	68505	NC_000085.6	Vps51	3.82E+00
gene29946	11861	NC_000078.6	Arl4a	3.82E+00
gene29391	68730	NC_000077.6	Dus1l	3.82E+00
gene26128	73690	NC_000076.6	Glipr1	3.81E+00
gene42066	225849	NC_000085.6	Ppp2r5b	3.81E+00
gene31592	665191	NC_000079.6	Gm7537	3.81E+00
gene17306	434156	NC_000073.6	Eid2b	3.81E+00
gene27313	22333	NC_000077.6	Vdac1	3.81E+00
gene15546	232337	NC_000072.6	Zfp637	3.80E+00
gene8895	24047	NC_000070.6	Ccl19	3.80E+00
gene21737	97484	NC_000074.6	Cog8	3.80E+00
gene2750	16922	NC_000068.7	Phyh	3.80E+00

gene13080	71667	NC_000071.6	Tmem248	3.80E+00
gene10958	97159	NC_000070.6	A430005L14Rik	3.80E+00
gene18806	68185	NC_000073.6	Coa4	3.79E+00
gene37164	105968	NC_000081.6	AU021063	3.79E+00
gene41580	66286	NC_000084.6	Sec11c	3.79E+00
gene760	14367	NC_000067.6	Fzd5	3.79E+00
gene32357	1E+08	NC_000079.6	Gm10784	3.79E+00
gene22152	244666	NC_000074.6	Sprtn	3.79E+00
gene32465	1E+08	NC_000079.6	Gm2830	3.79E+00
gene26231	15978	NC_000076.6	Ifng	3.78E+00
gene5955	66404	NC_000068.7	Rtfdc1	3.78E+00
gene20194	14068	NC_000074.6	F7	3.78E+00
gene37019	380967	NC_000081.6	Tmem106c	3.78E+00
gene2475	12334	NC_000067.6	Capn2	3.78E+00
gene1883	19734	NC_000067.6	Rgs16	3.77E+00
gene22505	66962	NC_000075.6	Swsap1	3.77E+00
gene28215	103743	NC_000077.6	Tmem98	3.77E+00
gene11008	26561	NC_000070.6	Mmp23	3.77E+00
gene8149	67547	NC_000069.6	Slc39a8	3.77E+00
gene10983	66469	NC_000070.6	Fam213b	3.77E+00
gene228	665829	NC_000067.6	Tubb4b-ps2	3.76E+00
gene20156	69225	NC_000074.6	Carkd	3.76E+00
gene16266	68988	NC_000073.6	Prpf31	3.76E+00
gene29317	192170	NC_000077.6	Eif4a3	3.76E+00
gene25635	56214	NC_000076.6	Scamp4	3.76E+00
gene17216	13088	NC_000073.6	Cyp2b10	3.75E+00
gene28688	12775	NC_000077.6	Ccr7	3.75E+00
gene36131	1E+08	NC_000081.6	Gm10020	3.75E+00
gene20053	67439	NC_000074.6	Xab2	3.75E+00
gene18455	101869	NC_000073.6	Unc45a	3.75E+00
gene8550	14579	NC_000070.6	Gem	3.74E+00
gene39952	68463	NC_000083.6	Mrpl14	3.74E+00
gene29341	1E+08	NC_000077.6	Gm11769	3.74E+00
gene41730	66191	NC_000084.6	Ier3ip1	3.74E+00
gene24366	215418	NC_000075.6	Csrnp1	3.74E+00
gene37274	16402	NC_000081.6	Itga5	3.74E+00
gene15409	67166	NC_000072.6	Arl8b	3.74E+00
gene13990	67705	NC_000072.6	1810058I24Rik	3.74E+00
gene20975	67184	NC_000074.6	Ndufa13	3.73E+00
gene24337	1E+08	NC_000075.6	Gm10157	3.73E+00
gene3333	99412	NC_000068.7	Golga2	3.73E+00
gene5481	54711	NC_000068.7	Plagl2	3.73E+00
gene11608	11518	NC_000071.6	Add1	3.73E+00
gene42161	56389	NC_000085.6	Stx5a	3.73E+00
gene35637	68059	NC_000080.6	Tm9sf2	3.73E+00
gene20085	102209	NC_000074.6	Snapc2	3.73E+00
gene23969	69010	NC_000075.6	Anapc13	3.73E+00
gene22307	71946	NC_000075.6	Endod1	3.73E+00

gene21010	75620	NC_000074.6	Kxd1	3.73E+00
gene21734	116733	NC_000074.6	Vps4a	3.72E+00
gene26792	19058	NC_000077.6	Ppp3r1	3.72E+00
gene12223	109979	NC_000071.6	Art3	3.72E+00
gene33512	59054	NC_000079.6	Mrps30	3.72E+00
gene9730	652920	NC_000070.6	Gm12870	3.72E+00
gene14612	667115	NC_000072.6	Gm15534	3.72E+00
gene15756	1E+08	NC_000072.6	Gm38549	3.72E+00
gene39705	69192	NC_000083.6	Dhx16	3.72E+00
gene12787	71954	NC_000071.6	Suds3	3.72E+00
gene37347	74022	NC_000082.6	Glyr1	3.71E+00
gene13561	15505	NC_000071.6	Hsph1	3.71E+00
gene8115	114249	NC_000069.6	Npnt	3.71E+00
gene24459	72309	NC_000075.6	Tmem158	3.71E+00
gene29033	66997	NC_000077.6	Psmd12	3.71E+00
gene30712	14744	NC_000078.6	Gpr65	3.71E+00
gene16698	1E+08	NC_000073.6	Phf20-ps	3.70E+00
gene37354	208211	NC_000082.6	Alg1	3.70E+00
gene29827	211986	NC_000078.6	Tmem18	3.70E+00
gene26115	53605	NC_000076.6	Nap1l1	3.70E+00
gene23765	12047	NC_000075.6	Bcl2a1d	3.70E+00
gene22484	382062	NC_000075.6	AB124611	3.70E+00
gene39439	1E+08	NC_000083.6	Gm30090	3.70E+00
gene23152	67834	NC_000075.6	Idh3a	3.70E+00
gene26694	18293	NC_000077.6	Ogdh	3.69E+00
gene17935	20174	NC_000073.6	Ruvbl2	3.68E+00
gene39616	12263	NC_000083.6	C2	3.68E+00
gene17464	54135	NC_000073.6	Lsr	3.68E+00
gene20772	70925	NC_000074.6	Cdkn2aip	3.68E+00
gene1018	20297	NC_000067.6	Ccl20	3.68E+00
gene20970	1E+08	NC_000074.6	Gm33695	3.68E+00
gene8141	67006	NC_000069.6	Cisd2	3.68E+00
gene1943	215015	NC_000067.6	Fam20b	3.67E+00
gene13240	78829	NC_000071.6	Tsc22d4	3.67E+00
gene4049	1E+08	NC_000068.7	Gm13712	3.67E+00
gene5240	20639	NC_000068.7	Snrpb2	3.67E+00
gene31119	71963	NC_000078.6	Cdca4	3.67E+00
gene19920	64540	NC_000073.6	Tspan4	3.67E+00
gene26856	1E+08	NC_000077.6	Gm12045	3.67E+00
gene141	67923	NC_000067.6	Tceb1	3.66E+00
gene36457	20289	NC_000081.6	Scx	3.66E+00
gene30362	14776	NC_000078.6	Gpx2	3.66E+00
gene26467	211389	NC_000076.6	Suox	3.66E+00
gene1303	623430	NC_000067.6	Gm6430	3.66E+00
gene8415	53861	NC_000069.6	Zranb2	3.66E+00
gene17212	74134	NC_000073.6	Cyp2s1	3.66E+00
gene17876	233204	NC_000073.6	Tbc1d17	3.66E+00
gene13267	66455	NC_000071.6	Cnpy4	3.65E+00

gene2218	30941	NC_000067.6	Usp21	3.65E+00
gene19611	16409	NC_000073.6	Itgam	3.65E+00
gene41217	67180	NC_000084.6	Yipf5	3.65E+00
gene22932	270150	NC_000075.6	Ccdc153	3.65E+00
gene39520	17916	NC_000083.6	Myo1f	3.65E+00
gene41669	240396	NC_000084.6	Mex3c	3.64E+00
gene24989	14628	NC_000076.6	Ostm1	3.64E+00
gene33202	59050	NC_000079.6	Nsa2	3.64E+00
gene32480	74257	NC_000079.6	Tspan17	3.64E+00
gene20897	66234	NC_000074.6	Msmo1	3.64E+00
gene25589	216156	NC_000076.6	Wdr18	3.63E+00
gene40228	106628	NC_000083.6	Trip10	3.63E+00
gene39187	213773	NC_000083.6	Tbl3	3.63E+00
gene25215	71767	NC_000076.6	Tysnd1	3.63E+00
gene39174	224619	NC_000083.6	Traf7	3.63E+00
gene21542	79233	NC_000074.6	Zfp319	3.63E+00
gene2127	68944	NC_000067.6	Tmco1	3.62E+00
gene35145	105513	NC_000080.6	Chmp7	3.62E+00
gene16890	70333	NC_000073.6	Cd3eap	3.62E+00
gene12976	67956	NC_000071.6	Kmt5a	3.62E+00
gene21709	77411	NC_000074.6	Esrp2	3.62E+00
gene39455	17691	NC_000083.6	Sik1	3.62E+00
gene4544	14221	NC_000068.7	Fjx1	3.62E+00
gene38595	170765	NC_000082.6	Ripply3	3.62E+00
gene15458	171508	NC_000072.6	Creld1	3.61E+00
gene34820	219094	NC_000080.6	Khnyn	3.61E+00
gene8824	230082	NC_000070.6	Nol6	3.61E+00
gene10282	67898	NC_000070.6	Pef1	3.61E+00
gene42902	94280	NC_000085.6	Sfxn3	3.61E+00
gene11054	57741	NC_000070.6	Noc2l	3.61E+00
gene42090	26379	NC_000085.6	Esrra	3.61E+00
gene16801	19222	NC_000073.6	Ptgir	3.61E+00
gene42037	19708	NC_000085.6	Dpf2	3.61E+00
gene28029	246257	NC_000077.6	Ovca2	3.60E+00
gene17457	12483	NC_000073.6	Cd22	3.60E+00
gene28834	17966	NC_000077.6	Nbr1	3.60E+00
gene40302	68767	NC_000083.6	Wash1	3.60E+00
gene28173	78889	NC_000077.6	Wsb1	3.60E+00
gene12732	11409	NC_000071.6	Acads	3.60E+00
gene25681	27375	NC_000076.6	Tjp3	3.60E+00
gene18656	233489	NC_000073.6	Picalm	3.59E+00
gene6182	76688	NC_000068.7	Arfrp1	3.59E+00
gene13172	20022	NC_000071.6	Polr2j	3.59E+00
gene5870	58220	NC_000068.7	Pard6b	3.59E+00
gene26981	69556	NC_000077.6	Bod1	3.59E+00
gene12754	19303	NC_000071.6	Pxn	3.59E+00
gene19307	70349	NC_000073.6	Copb1	3.59E+00
gene3681	1E+08	NC_000068.7	Gm13552	3.58E+00

gene1682	320139	NC_000067.6	Ptpn7	3.58E+00
gene3116	51875	NC_000068.7	Tmem141	3.58E+00
gene5371	ene=LOC1	NC_000068.7	LOC102631547	3.58E+00
gene16916	19294	NC_000073.6	Nectin2	3.58E+00
gene30551	58520	NC_000078.6	0610007P14Rik	3.58E+00
gene3400	623269	NC_000068.7	Gm13414	3.58E+00
gene1937	20652	NC_000067.6	Soat1	3.58E+00
gene11511	57742	NC_000071.6	Abhd1	3.58E+00
gene19621	21804	NC_000073.6	Tgfb1i1	3.58E+00
gene16177	67529	NC_000072.6	Fgfr1op2	3.58E+00
gene42228	225912	NC_000085.6	Cyb561a3	3.57E+00
gene972	57814	NC_000067.6	Kcne4	3.57E+00
gene8947	1E+08	NC_000070.6	Gm12492	3.57E+00
gene26406	11536	NC_000076.6	Gpr182	3.57E+00
gene14058	68198	NC_000072.6	Ndufb2	3.57E+00
gene19382	233802	NC_000073.6	Thumpd1	3.57E+00
gene32566	56085	NC_000079.6	Ubqln1	3.57E+00
gene41146	24074	NC_000084.6	Taf7	3.57E+00
gene21428	384862	NC_000074.6	Gm5356	3.57E+00
gene31596	66308	NC_000079.6	Mplkip	3.56E+00
gene23593	50875	NC_000075.6	Tmod3	3.56E+00
gene15103	66488	NC_000072.6	Fam136a	3.56E+00
gene15586	232341	NC_000072.6	Wnk1	3.56E+00
gene10147	76793	NC_000070.6	Snip1	3.56E+00
gene24275	56693	NC_000075.6	Crtap	3.56E+00
gene25637	1E+08	NC_000076.6	Gm31298	3.55E+00
gene29021	69066	NC_000077.6	1810010H24Rik	3.55E+00
gene17339	1E+08	NC_000073.6	Gm19897	3.55E+00
gene978	69163	NC_000067.6	Mrpl44	3.55E+00
gene3008	433408	NC_000068.7	Gm13375	3.55E+00
gene25762	28109	NC_000076.6	D10Wsu102e	3.55E+00
gene28864	76547	NC_000077.6	Tmem101	3.55E+00
gene9259	72748	NC_000070.6	Hdhd3	3.54E+00
gene36378	1.1E+08	NC_000081.6	Gm41348	3.54E+00
gene14170	76263	NC_000072.6	Gstk1	3.54E+00
gene22470	235040	NC_000075.6	Atg4d	3.53E+00
gene7703	15257	NC_000069.6	Hipk1	3.53E+00
gene39845	1E+08	NC_000083.6	Gm18737	3.53E+00
gene14350	317757	NC_000072.6	Gimap5	3.53E+00
gene40754	435554	NC_000084.6	Gm5686	3.53E+00
gene12610	1E+08	NC_000071.6	Gm26515	3.53E+00
gene3274	12908	NC_000068.7	Crat	3.53E+00
gene11686	66309	NC_000071.6	Tmem128	3.53E+00
gene41508	1E+08	NC_000084.6	Gm17883	3.52E+00
gene34799	75751	NC_000080.6	Ipo4	3.52E+00
gene17397	26465	NC_000073.6	Zfp146	3.51E+00
gene3791	73373	NC_000068.7	Phospho2	3.51E+00
gene16042	60321	NC_000072.6	Wbp11	3.51E+00

gene12587	107999	NC_000071.6	Gtpbp6	3.51E+00
gene9844	66588	NC_000070.6	Cmpk1	3.51E+00
gene42932	70769	NC_000085.6	Nolc1	3.51E+00
gene28608	103551	NC_000077.6	E130012A19Rik	3.51E+00
gene2609	66208	NC_000067.6	Nenf	3.51E+00
gene30338	81535	NC_000078.6	Sgpp1	3.51E+00
gene43015	76479	NC_000085.6	Smndc1	3.50E+00
gene12982	56334	NC_000071.6	Tmed2	3.50E+00
gene7272	18160	NC_000069.6	Npr1	3.50E+00
gene1738	19264	NC_000067.6	Ptprc	3.50E+00
gene5058	16175	NC_000068.7	Il1a	3.50E+00
gene4820	67809	NC_000068.7	Rmdn3	3.50E+00
gene28014	18472	NC_000077.6	Pafah1b1	3.50E+00
gene4996	320632	NC_000068.7	Snrnp200	3.50E+00
gene17435	101883	NC_000073.6	Igflr1	3.49E+00
gene29254	74136	NC_000077.6	Sec14l1	3.49E+00
gene10921	230936	NC_000070.6	Phf13	3.49E+00
gene27239	76795	NC_000077.6	Tbc1d9b	3.49E+00
gene15122	67170	NC_000072.6	2610306M01Rik	3.49E+00
gene30385	73834	NC_000078.6	Atp6v1d	3.49E+00
gene22686	1E+08	NC_000075.6	Gm26787	3.48E+00
gene39258	268933	NC_000083.6	Wdr24	3.48E+00
gene39183	213753	NC_000083.6	Zfp598	3.48E+00
gene11753	110391	NC_000071.6	Qdpr	3.48E+00
gene23189	56434	NC_000075.6	Tspan3	3.47E+00
gene7189	16800	NC_000069.6	Arhgef2	3.47E+00
gene24046	76491	NC_000075.6	Abhd14b	3.47E+00
gene37757	68841	NC_000082.6	Lppos	3.47E+00
gene37232	26404	NC_000081.6	Map3k12	3.47E+00
gene14063	665247	NC_000072.6	Gm7554	3.47E+00
gene17357	73833	NC_000073.6	Fam98c	3.47E+00
gene27194	18356	NC_000077.6	Olfr56	3.47E+00
gene8566	545592	NC_000070.6	Gm11847	3.47E+00
gene10256	16818	NC_000070.6	Lck	3.47E+00
gene29141	217303	NC_000077.6	Cd300a	3.47E+00
gene37638	75785	NC_000082.6	Klhl24	3.47E+00
gene34809	67842	NC_000080.6	Nop9	3.46E+00
gene1288	67698	NC_000067.6	Fam174a	3.46E+00
gene7733	80281	NC_000069.6	Cttnbp2nl	3.46E+00
gene17257	108075	NC_000073.6	Ltbp4	3.46E+00
gene17224	667968	NC_000073.6	Gm8902	3.46E+00
gene17911	69748	NC_000073.6	Aldh16a1	3.45E+00
gene42034	67457	NC_000085.6	Frmd8	3.45E+00
gene8799	106021	NC_000070.6	Topors	3.45E+00
gene4398	66461	NC_000068.7	Ptpmt1	3.45E+00
gene23259	16949	NC_000075.6	Loxl1	3.45E+00
gene7241	56417	NC_000069.6	Adar	3.45E+00
gene32468	56795	NC_000079.6	Arl10	3.44E+00

gene40292	30960	NC_000083.6	Vapa	3.44E+00
gene19502	20399	NC_000073.6	Sh2b1	3.44E+00
gene39344	224647	NC_000083.6	D17Wsu92e	3.44E+00
gene5739	17183	NC_000068.7	Matn4	3.44E+00
gene12727	72357	NC_000071.6	2210016L21Rik	3.44E+00
gene36412	223646	NC_000081.6	Naprt	3.44E+00
gene37852	224109	NC_000082.6	Nrros	3.44E+00
gene26424	56351	NC_000076.6	Ptges3	3.43E+00
gene17433	66340	NC_000073.6	Psenen	3.43E+00
gene20842	97165	NC_000074.6	Hmgb2	3.43E+00
gene27291	66050	NC_000077.6	0610009B22Rik	3.43E+00
gene4037	30059	NC_000068.7	Timm10	3.43E+00
gene17785	12489	NC_000073.6	Cd33	3.43E+00
gene4869	66296	NC_000068.7	Haus2	3.42E+00
gene38708	1E+08	NC_000083.6	Gm15590	3.42E+00
gene38555	1E+08	NC_000082.6	1700048M11Rik	3.42E+00
gene42584	16452	NC_000085.6	Jak2	3.42E+00
gene13672	330260	NC_000072.6	Pon2	3.42E+00
gene25596	625249	NC_000076.6	Gpx4	3.42E+00
gene40127	435531	NC_000083.6	Gm5684	3.42E+00
gene7274	67781	NC_000069.6	Ilf2	3.42E+00
gene30929	22632	NC_000078.6	Yy1	3.42E+00
gene26840	ene;gene=L	NC_000077.6	LOC108167886	3.42E+00
gene42098	18797	NC_000085.6	Plcb3	3.42E+00
gene35414	76789	NC_000080.6	Mzt1	3.42E+00
gene24485	ene;gene=L	NC_000075.6	LOC108167694	3.42E+00
gene42576	56248	NC_000085.6	Ak3	3.42E+00
gene11496	74919	NC_000071.6	Slc35f6	3.42E+00
gene4432	17242	NC_000068.7	Mdk	3.41E+00
gene31500	382722	NC_000079.6	Gm5191	3.41E+00
gene28048	215113	NC_000077.6	Slc43a2	3.41E+00
gene20693	11886	NC_000074.6	Asah1	3.41E+00
gene8528	71801	NC_000070.6	Plekhf2	3.41E+00
gene25150	19386	NC_000076.6	Ranbp2	3.41E+00
gene39525	19326	NC_000083.6	Rab11b	3.41E+00
gene3634	67874	NC_000068.7	Rprm	3.41E+00
gene26068	52713	NC_000076.6	Ccdc59	3.41E+00
gene25237	30930	NC_000076.6	Vps26a	3.41E+00
gene41615	72124	NC_000084.6	Seh1l	3.40E+00
gene4025	66958	NC_000068.7	Tmx2	3.40E+00
gene25570	70294	NC_000076.6	Rnf126	3.40E+00
gene42096	12015	NC_000085.6	Bad	3.40E+00
gene23142	1E+08	NC_000075.6	Gm16380	3.40E+00
gene12977	80291	NC_000071.6	Rilpl2	3.39E+00
gene5054	20515	NC_000068.7	Slc20a1	3.39E+00
gene40968	69241	NC_000084.6	Polr2d	3.39E+00
gene26384	216443	NC_000076.6	Mars	3.39E+00
gene40732	14370	NC_000084.6	Fzd8	3.39E+00

gene19531	233875	NC_000073.6	Ino80e	3.39E+00
gene21773	64340	NC_000074.6	Dhx38	3.39E+00
gene39204	193838	NC_000083.6	Eme2	3.38E+00
gene42159	107071	NC_000085.6	Wdr74	3.38E+00
gene35023	20680	NC_000080.6	Sox7	3.38E+00
gene21519	20299	NC_000074.6	Ccl22	3.38E+00
gene30166	622746	NC_000078.6	Gm6353	3.38E+00
gene29614	217430	NC_000078.6	Pqlc3	3.38E+00
gene37167	68118	NC_000081.6	Atg101	3.38E+00
gene37467	18203	NC_000082.6	Ntan1	3.38E+00
gene26383	13198	NC_000076.6	Ddit3	3.38E+00
gene32244	76137	NC_000079.6	Mcur1	3.38E+00
gene24455	69035	NC_000075.6	Zdhhc3	3.37E+00
gene9852	384042	NC_000070.6	Gm12834	3.37E+00
gene42026	114601	NC_000085.6	Ehbp1l1	3.37E+00
gene8384	19352	NC_000069.6	Rabggtb	3.37E+00
gene38068	17470	NC_000082.6	Cd200	3.37E+00
gene28447	638903	NC_000077.6	Gm11516	3.37E+00
gene28041	192652	NC_000077.6	Wdr81	3.37E+00
gene37525	68606	NC_000082.6	Ppm1f	3.37E+00
gene16350	22185	NC_000073.6	U2af2	3.37E+00
gene39746	67676	NC_000083.6	Rpp21	3.36E+00
gene41096	15115	NC_000084.6	Hars	3.36E+00
gene1251	71874	NC_000067.6	2310007B03Rik	3.36E+00
gene29214	11430	NC_000077.6	Acox1	3.36E+00
gene26795	57316	NC_000077.6	C1d	3.36E+00
gene30220	207965	NC_000078.6	Vcpkmt	3.36E+00
gene19906	320871	NC_000073.6	B230206H07Rik	3.36E+00
gene8748	433693	NC_000070.6	Akirin2	3.36E+00
gene25887	21917	NC_000076.6	Tmpo	3.35E+00
gene33768	16188	NC_000080.6	Il3ra	3.35E+00
gene23977	24059	NC_000075.6	Slco2a1	3.35E+00
gene29248	69900	NC_000077.6	Mfsd11	3.35E+00
gene24637	73112	NC_000076.6	Abracl	3.35E+00
gene8924	22004	NC_000070.6	Tpm2	3.35E+00
gene16654	232879	NC_000073.6	Zbtb45	3.34E+00
gene31425	1E+08	NC_000078.6	Gm18032	3.34E+00
gene29155	66176	NC_000077.6	Nat9	3.34E+00
gene42187	225898	NC_000085.6	Eml3	3.34E+00
gene34772	68966	NC_000080.6	Ngdn	3.34E+00
gene3611	71684	NC_000068.7	Rbm43	3.34E+00
gene40719	622384	NC_000084.6	Fabp5l2	3.33E+00
gene25473	22213	NC_000076.6	Ube2g2	3.33E+00
gene15093	72102	NC_000072.6	Dusp11	3.33E+00
gene19366	66356	NC_000073.6	Knop1	3.33E+00
gene7184	53868	NC_000069.6	Rab25	3.33E+00
gene37651	27406	NC_000082.6	Abcf3	3.33E+00
gene19664	67872	NC_000073.6	Nsmce4a	3.33E+00

gene26375	80904	NC_000076.6	Dtx3	3.33E+00
gene2245	12523	NC_000067.6	Cd84	3.33E+00
gene30368	17187	NC_000078.6	Max	3.33E+00
gene7476	229595	NC_000069.6	Adamtsl4	3.32E+00
gene29534	105014	NC_000078.6	Rdh14	3.32E+00
gene4026	381379	NC_000068.7	Med19	3.32E+00
gene10922	242785	NC_000070.6	Klhl21	3.32E+00
gene27781	22059	NC_000077.6	Trp53	3.32E+00
gene13047	243277	NC_000071.6	Adgrd1	3.32E+00
gene42691	107765	NC_000085.6	Ankrd1	3.32E+00
gene26127	52705	NC_000076.6	Krr1	3.32E+00
gene7256	73545	NC_000069.6	1700094D03Rik	3.32E+00
gene25575	78906	NC_000076.6	Misp	3.31E+00
gene5233	71436	NC_000068.7	Flrt3	3.31E+00
gene22576	69091	NC_000075.6	Vps26b	3.31E+00
gene19239	19347	NC_000073.6	Dennd5a	3.31E+00
gene33193	15357	NC_000079.6	Hmgcr	3.31E+00
gene25690	56371	NC_000076.6	Fzr1	3.31E+00
gene18643	76453	NC_000073.6	Prss23	3.31E+00
gene25035	1E+08	NC_000076.6	2310075K07Rik	3.31E+00
gene35993	=Gene;ger	NC_000081.6	LOC105245924	3.31E+00
gene7504	319190	NC_000069.6	Hist2h2be	3.31E+00
gene5295	66877	NC_000068.7	Crnkl1	3.31E+00
gene34858	1E+08	NC_000080.6	Gm4037	3.31E+00
gene12417	74167	NC_000071.6	Nudt9	3.30E+00
gene32508	27261	NC_000079.6	Dok3	3.30E+00
gene6811	229323	NC_000069.6	Gpr171	3.30E+00
gene42335	74333	NC_000085.6	A330040F15Rik	3.30E+00
gene13795	76522	NC_000072.6	Lsm8	3.30E+00
gene36211	20775	NC_000081.6	Sqle	3.30E+00
gene7266	74343	NC_000069.6	Crtc2	3.30E+00
gene1589	226422	NC_000067.6	Rab29	3.30E+00
gene10166	12986	NC_000070.6	Csf3r	3.30E+00
gene24525	68632	NC_000076.6	Myct1	3.30E+00
gene5477	15162	NC_000068.7	Hck	3.30E+00
gene17169	232976	NC_000073.6	Zfp574	3.29E+00
gene39487	623809	NC_000083.6	Gm6452	3.29E+00
gene31881	30946	NC_000079.6	Abt1	3.29E+00
gene39646	81845	NC_000083.6	Gpank1	3.29E+00
gene37426	14852	NC_000082.6	Gspt1	3.29E+00
gene35858	52521	NC_000081.6	Zfp622	3.29E+00
gene4468	277414	NC_000068.7	Trp53i11	3.29E+00
gene10262	230767	NC_000070.6	Iqcc	3.28E+00
gene11806	67073	NC_000071.6	Pi4k2b	3.28E+00
gene8810	74255	NC_000070.6	Smu1	3.28E+00
gene8919	54390	NC_000070.6	Sit1	3.28E+00
gene42252	240539	NC_000085.6	Gm336	3.28E+00
gene13655	12068	NC_000072.6	Bet1	3.28E+00

gene25604	69770	NC_000076.6	1600002K03Rik	3.27E+00
gene1057	12283	NC_000067.6	Cab39	3.27E+00
gene39995	224823	NC_000083.6	Rrp36	3.27E+00
gene313	19253	NC_000067.6	Ptpn18	3.27E+00
gene16150	67636	NC_000072.6	Lyrm5	3.27E+00
gene20425	57437	NC_000074.6	Golga7	3.27E+00
gene18529	170460	NC_000073.6	Stard5	3.27E+00
gene4416	77038	NC_000068.7	Arfgap2	3.27E+00
gene27415	216760	NC_000077.6	Mfap3	3.27E+00
gene19532	233876	NC_000073.6	Hirip3	3.27E+00
gene30315	1E+08	NC_000078.6	Gm34189	3.27E+00
gene41463	66810	NC_000084.6	Rbm22	3.27E+00
gene37078	22146	NC_000081.6	Tuba1c	3.26E+00
gene11023	13542	NC_000070.6	Dvl1	3.26E+00
gene11877	1E+08	NC_000071.6	Gm38457	3.26E+00
gene6740	170767	NC_000069.6	Rfxap	3.26E+00
gene9870	100465	NC_000070.6	Mob3c	3.26E+00
gene32772	628746	NC_000079.6	Rybp-ps	3.26E+00
gene6376	70808	NC_000069.6	4632415L05Rik	3.26E+00
gene19619	1E+08	NC_000073.6	9130023H24Rik	3.26E+00
gene24130	72454	NC_000075.6	Ccdc71	3.26E+00
gene29113	16834	NC_000077.6	Cog1	3.26E+00
gene6247	545500	NC_000069.6	Gm5841	3.25E+00
gene15263	64658	NC_000072.6	Mrps25	3.25E+00
gene1927	104009	NC_000067.6	Qsox1	3.25E+00
gene32336	68480	NC_000079.6	Card19	3.25E+00
gene6892	433604	NC_000069.6	Gm5540	3.25E+00
gene15176	80283	NC_000072.6	Abtb1	3.25E+00
gene16664	666177	NC_000073.6	Gm7965	3.25E+00
gene33530	ene;gene=L	NC_000079.6	LOC108168049	3.25E+00
gene29171	70382	NC_000077.6	Kctd2	3.25E+00
gene6919	17035	NC_000069.6	Lxn	3.25E+00
gene2239	75345	NC_000067.6	Slamf7	3.25E+00
gene21531	14766	NC_000074.6	Adgrg1	3.24E+00
gene13002	22190	NC_000071.6	Ubc	3.24E+00
gene42675	1E+08	NC_000085.6	Cnn2-ps	3.24E+00
gene4737	11464	NC_000068.7	Actc1	3.24E+00
gene14909	53620	NC_000072.6	Vamp5	3.24E+00
gene37027	239650	NC_000081.6	Ccdc184	3.24E+00
gene7653	19221	NC_000069.6	Ptgfrn	3.24E+00
gene17387	625421	NC_000073.6	C230062I16Rik	3.24E+00
gene20213	272465	NC_000074.6	Tmem255b	3.24E+00
gene13432	56443	NC_000071.6	Arpc1a	3.24E+00
gene29153	246746	NC_000077.6	Cd300lf	3.23E+00
gene27803	380705	NC_000077.6	Tmem102	3.23E+00
gene4867	20619	NC_000068.7	Snap23	3.23E+00
gene21543	101985	NC_000074.6	Usb1	3.23E+00
gene42294	76303	NC_000085.6	Osbp	3.23E+00

gene40998	170459	NC_000084.6	Stard4	3.23E+00
gene16340	23877	NC_000073.6	Fiz1	3.23E+00
gene12872	19247	NC_000071.6	Ptpn11	3.23E+00
gene13841	68202	NC_000072.6	Ndufa5	3.23E+00
gene29551	627110	NC_000078.6	Tubb2a-ps2	3.22E+00
gene13671	269823	NC_000072.6	Pon3	3.22E+00
gene41686	17190	NC_000084.6	Mbd1	3.22E+00
gene14637	56150	NC_000072.6	Mad2l1	3.22E+00
gene15180	24100	NC_000072.6	Tpra1	3.22E+00
gene23615	14629	NC_000075.6	Gclc	3.22E+00
gene8813	14595	NC_000070.6	B4galt1	3.22E+00
gene18016	101685	NC_000073.6	Spty2d1	3.22E+00
gene33314	17079	NC_000079.6	Cd180	3.22E+00
gene5275	26450	NC_000068.7	Rbbp9	3.22E+00
gene7451	56772	NC_000069.6	Mllt11	3.22E+00
gene16674	1E+08	NC_000073.6	Rps8-ps4	3.21E+00
gene4570	16909	NC_000068.7	Lmo2	3.21E+00
gene27825	54351	NC_000077.6	Elp5	3.21E+00
gene33713	13421	NC_000080.6	Dnase1l3	3.21E+00
gene33485	17434	NC_000079.6	Mocs2	3.21E+00
gene5490	99311	NC_000068.7	Commd7	3.21E+00
gene34033	30877	NC_000080.6	Gnl3	3.21E+00
gene8339	623554	NC_000069.6	Gm6439	3.21E+00
gene32971	80898	NC_000079.6	Erap1	3.21E+00
gene26447	69917	NC_000076.6	Nabp2	3.20E+00
gene14917	67442	NC_000072.6	Retsat	3.20E+00
gene34882	20220	NC_000080.6	Sap18	3.20E+00
gene4891	12716	NC_000068.7	Ckmt1	3.20E+00
gene36788	18141	NC_000081.6	Nup50	3.20E+00
gene21376	666945	NC_000074.6	Gm10638	3.20E+00
gene8022	1E+08	NC_000069.6	Gm18916	3.19E+00
gene3690	66205	NC_000068.7	Cd302	3.19E+00
gene25639	208198	NC_000076.6	Btbd2	3.19E+00
gene32068	66222	NC_000079.6	Serpinb1a	3.19E+00
gene26736	22271	NC_000077.6	Upp1	3.19E+00
gene11927	67878	NC_000071.6	Tmem33	3.19E+00
gene34790	74551	NC_000080.6	Pck2	3.19E+00
gene28651	21393	NC_000077.6	Tcap	3.19E+00
gene25693	72273	NC_000076.6	Smim24	3.19E+00
gene26154	216344	NC_000076.6	Rab21	3.19E+00
gene21444	14339	NC_000074.6	Aktip	3.19E+00
gene4967	12848	NC_000068.7	Cops2	3.18E+00
gene775	15926	NC_000067.6	Idh1	3.18E+00
gene19876	212974	NC_000073.6	Athl1	3.18E+00
gene29374	68671	NC_000077.6	Pcyt2	3.18E+00
gene37370	66690	NC_000082.6	Tmem186	3.18E+00
gene2314	26388	NC_000067.6	Ifi202b	3.18E+00
gene41043	12385	NC_000084.6	Ctnna1	3.18E+00

gene15744	78668	NC_000072.6	E130112N10Rik	3.18E+00
gene28981	=Gene;ger	NC_000077.6	LOC108167928	3.17E+00
gene19557	66422	NC_000073.6	Dctpp1	3.17E+00
gene36683	20788	NC_000081.6	Srebf2	3.17E+00
gene10866	74310	NC_000070.6	Ube4bos3	3.17E+00
gene9655	433739	NC_000070.6	Gm12799	3.17E+00
gene4397	67136	NC_000068.7	Kbtbd4	3.17E+00
gene7032	229445	NC_000069.6	Ctso	3.17E+00
gene8798	230073	NC_000070.6	Ddx58	3.17E+00
gene5677	629655	NC_000068.7	Gm11448	3.17E+00
gene28047	380713	NC_000077.6	Scarf1	3.17E+00
gene36669	11429	NC_000081.6	Aco2	3.17E+00
gene21848	57321	NC_000074.6	Terf2ip	3.17E+00
gene31533	56048	NC_000079.6	Lgals8	3.17E+00
gene2308	15951	NC_000067.6	Ifi204	3.17E+00
gene25554	50784	NC_000076.6	Plpp2	3.16E+00
gene24488	ene;gene=l	NC_000075.6	LOC108167320	3.16E+00
gene21068	70359	NC_000074.6	Gtpbp3	3.16E+00
gene35014	210376	NC_000080.6	Mtmr9	3.16E+00
gene29477	217379	NC_000078.6	Ubxn2a	3.16E+00
gene25406	71733	NC_000076.6	Susd2	3.16E+00
gene22977	12501	NC_000075.6	Cd3e	3.16E+00
gene41177	106952	NC_000084.6	Arap3	3.16E+00
gene40681	240185	NC_000084.6	9430020K01Rik	3.16E+00
gene15717	=Gene;ger	NC_000072.6	LOC108169168	3.16E+00
gene25296	13654	NC_000076.6	Egr2	3.16E+00
gene14907	232086	NC_000072.6	Tmem150a	3.16E+00
gene23435	56440	NC_000075.6	Snx1	3.16E+00
gene3792	433432	NC_000068.7	Gm13624	3.16E+00
gene31104	30954	NC_000078.6	Siva1	3.16E+00
gene39130	224613	NC_000083.6	Flywch1	3.16E+00
gene28315	1E+08	NC_000077.6	Ggnbp2os	3.16E+00
gene37267	15382	NC_000081.6	Hnrnpa1	3.15E+00
gene32881	28114	NC_000079.6	Nsun2	3.15E+00
gene7956	14066	NC_000069.6	F3	3.15E+00
gene22015	192156	NC_000074.6	Mvd	3.15E+00
gene39166	13177	NC_000083.6	Eci1	3.15E+00
gene10890	13806	NC_000070.6	Eno1	3.15E+00
gene4794	171543	NC_000068.7	Bmf	3.15E+00
gene9960	107995	NC_000070.6	Cdc20	3.14E+00
gene10190	26445	NC_000070.6	Psmb2	3.14E+00
gene27563	16897	NC_000077.6	Llgl1	3.14E+00
gene15119	66618	NC_000072.6	Snrnp27	3.14E+00
gene8829	68970	NC_000070.6	Dcaf12	3.14E+00
gene29384	170758	NC_000077.6	Rac3	3.14E+00
gene10358	100226	NC_000070.6	Stx12	3.14E+00
gene17570	50995	NC_000073.6	Uba2	3.14E+00
gene12678	231630	NC_000071.6	Ficd	3.14E+00

gene21673	11972	NC_000074.6	Atp6v0d1	3.14E+00
gene23211	73744	NC_000075.6	Man2c1	3.14E+00
gene1367	20479	NC_000067.6	Vps4b	3.13E+00
gene42597	240614	NC_000085.6	Ranbp6	3.13E+00
gene1665	57439	NC_000067.6	Tmem183a	3.13E+00
gene9720	56280	NC_000070.6	Mrpl37	3.13E+00
gene30182	30795	NC_000078.6	Fkbp3	3.13E+00
gene7255	99650	NC_000069.6	4933434E20Rik	3.13E+00
gene28044	380712	NC_000077.6	Tlcd2	3.13E+00
gene19609	436010	NC_000073.6	Gm5738	3.13E+00
gene40301	72900	NC_000083.6	Ndufv2	3.12E+00
gene26878	53625	NC_000077.6	B3gnt2	3.12E+00
gene7711	1E+08	NC_000069.6	Phtf1os	3.12E+00
gene38749	72536	NC_000083.6	Tagap	3.12E+00
gene13714	68240	NC_000072.6	Rpa3	3.12E+00
gene39132	75956	NC_000083.6	Srrm2	3.12E+00
gene27877	216874	NC_000077.6	Camta2	3.12E+00
gene9917	12795	NC_000070.6	Plk3	3.11E+00
gene25643	11776	NC_000076.6	Ap3d1	3.11E+00
gene8552	381511	NC_000070.6	Pdp1	3.11E+00
gene17931	20637	NC_000073.6	Snrnp70	3.11E+00
gene10993	20481	NC_000070.6	Ski	3.11E+00
gene42703	74493	NC_000085.6	Tnks2	3.11E+00
gene42103	57431	NC_000085.6	Dnajc4	3.11E+00
gene20680	667052	NC_000074.6	Gm8436	3.11E+00
gene5742	66460	NC_000068.7	Sys1	3.11E+00
gene9062	28028	NC_000070.6	Mrpl50	3.11E+00
gene37114	1E+08	NC_000081.6	Gm16537	3.10E+00
gene17577	67070	NC_000073.6	Lsm14a	3.10E+00
gene36025	66335	NC_000081.6	Atp6v1c1	3.10E+00
gene10011	106564	NC_000070.6	Ppcs	3.10E+00
gene28386	20922	NC_000077.6	Supt4a	3.10E+00
gene10199	1E+08	NC_000070.6	Gm12942	3.10E+00
gene32208	68083	NC_000079.6	Pak1ip1	3.10E+00
gene29117	56699	NC_000077.6	Cdc42ep4	3.10E+00
gene29693	67732	NC_000078.6	Iah1	3.10E+00
gene28417	73921	NC_000077.6	Scpep1os	3.09E+00
gene19572	233895	NC_000073.6	Prr14	3.09E+00
gene15729	28019	NC_000072.6	Ing4	3.09E+00
gene26370	=Gene;ger	NC_000076.6	LOC108167763	3.09E+00
gene38121	16423	NC_000082.6	Cd47	3.09E+00
gene4410	22259	NC_000068.7	Nr1h3	3.09E+00
gene25530	216136	NC_000076.6	Ilvbl	3.09E+00
gene34477	29811	NC_000080.6	Ndrg2	3.09E+00
gene24230	77781	NC_000075.6	Epm2aip1	3.09E+00
gene2265	67556	NC_000067.6	Pigm	3.08E+00
gene919	245860	NC_000067.6	Atg9a	3.08E+00
gene16295	232801	NC_000073.6	Lilra5	3.08E+00

gene13189	67286	NC_000071.6	Ift22	3.08E+00
gene1437	67949	NC_000067.6	Nifk	3.08E+00
gene18471	1E+08	NC_000073.6	Gm32112	3.08E+00
gene16870	13401	NC_000073.6	Dmwd	3.08E+00
gene7850	99890	NC_000069.6	Prmt6	3.07E+00
gene9651	384033	NC_000070.6	Gm12792	3.07E+00
gene15859	12515	NC_000072.6	Cd69	3.07E+00
gene26463	668459	NC_000076.6	Gm9182	3.07E+00
gene13402	231872	NC_000071.6	Aimp2	3.07E+00
gene39208	52009	NC_000083.6	Hn1l	3.07E+00
gene28072	56322	NC_000077.6	Timm22	3.07E+00
gene27517	26936	NC_000077.6	Mprip	3.07E+00
gene41543	53382	NC_000084.6	Txnl1	3.07E+00
gene412	52846	NC_000067.6	Cnot11	3.06E+00
gene38135	68190	NC_000082.6	Dubr	3.06E+00
gene6161	228994	NC_000068.7	Ythdf1	3.06E+00
gene36435	18810	NC_000081.6	Plec	3.06E+00
gene28909	1E+08	NC_000077.6	Gm34620	3.06E+00
gene12990	269704	NC_000071.6	Zfp664	3.06E+00
gene16224	67246	NC_000072.6	2810474O19Rik	3.06E+00
gene34813	104110	NC_000080.6	Adcy4	3.06E+00
gene28082	12874	NC_000077.6	Cpd	3.06E+00
gene5480	241732	NC_000068.7	Tspyl3	3.06E+00
gene28366	74133	NC_000077.6	Smg8	3.05E+00
gene36543	16185	NC_000081.6	Il2rb	3.05E+00
gene41950	225875	NC_000085.6	Lrfn4	3.05E+00
gene25694	18029	NC_000076.6	Nfic	3.05E+00
gene34739	27374	NC_000080.6	Prmt5	3.05E+00
gene35319	66214	NC_000080.6	Rgcc	3.05E+00
gene2128	56752	NC_000067.6	Aldh9a1	3.05E+00
gene25597	216161	NC_000076.6	Sbno2	3.05E+00
gene16843	232930	NC_000073.6	Gm4880	3.05E+00
gene6104	228966	NC_000068.7	Ppp1r3d	3.05E+00
gene36873	77980	NC_000081.6	Sbf1	3.05E+00
gene39415	68597	NC_000083.6	Ccdc167	3.04E+00
gene41192	56736	NC_000084.6	Rnf14	3.04E+00
gene41985	16594	NC_000085.6	Klc2	3.04E+00
gene1606	74137	NC_000067.6	Nuak2	3.04E+00
gene11649	231151	NC_000071.6	Tada2b	3.04E+00
gene25844	22172	NC_000076.6	Tyms-ps	3.04E+00
gene27372	72729	NC_000077.6	Cdc42se2	3.04E+00
gene40455	68554	NC_000083.6	Cebpzos	3.04E+00
gene36668	68479	NC_000081.6	Phf5a	3.04E+00
gene1869	16782	NC_000067.6	Lamc2	3.03E+00
gene19581	233899	NC_000073.6	Ccdc189	3.03E+00
gene26608	103710	NC_000077.6	Slc35e4	3.03E+00
gene39683	106795	NC_000083.6	Tcf19	3.03E+00
gene13117	19718	NC_000071.6	Rfc2	3.03E+00

gene11882	21897	NC_000071.6	Tlr1	3.03E+00
gene13265	11781	NC_000071.6	Ap4m1	3.02E+00
gene40148	50498	NC_000083.6	Ebi3	3.02E+00
gene42847	627624	NC_000085.6	Gm6776	3.02E+00
gene3097	11305	NC_000068.7	Abca2	3.02E+00
gene11274	20348	NC_000071.6	Sema3c	3.02E+00
gene1105	26987	NC_000067.6	Eif4e2	3.02E+00
gene4966	70354	NC_000068.7	Secisbp2l	3.02E+00
gene19332	=Gene;ger	NC_000073.6	LOC102637815	3.02E+00
gene19961	677289	NC_000073.6	Prr33	3.02E+00
gene7174	229517	NC_000069.6	Slc25a44	3.02E+00
gene12984	209354	NC_000071.6	Eif2b1	3.02E+00
gene13261	17220	NC_000071.6	Mcm7	3.02E+00
gene10469	74246	NC_000070.6	Gale	3.01E+00
gene15592	16172	NC_000072.6	Il17ra	3.01E+00
gene10566	12345	NC_000070.6	Capzb	3.01E+00
gene2671	1E+08	NC_000067.6	Gm19777	3.01E+00
gene9866	546843	NC_000070.6	Gm12848	3.01E+00
gene10269	20218	NC_000070.6	Khdrbs1	3.01E+00
gene10874	65945	NC_000070.6	Clstn1	3.01E+00
gene5792	228875	NC_000068.7	Slc35c2	3.00E+00
gene23012	22687	NC_000075.6	Zpr1	3.00E+00
gene875	12765	NC_000067.6	Cxcr2	3.00E+00
gene16868	68188	NC_000073.6	Sympk	3.00E+00
gene35480	435420	NC_000080.6	Gm5671	3.00E+00
gene20823	15446	NC_000074.6	Hpgd	3.00E+00
gene15166	14461	NC_000072.6	Gata2	3.00E+00
gene788	66646	NC_000067.6	Rpe	3.00E+00
gene6282	55980	NC_000069.6	Impa1	3.00E+00
gene1052	=Gene;ger	NC_000067.6	LOC102634459	3.00E+00
gene13441	72611	NC_000071.6	Zfp655	3.00E+00
gene19553	54204	NC_000073.6	sep-01	3.00E+00
gene17910	14256	NC_000073.6	Flt3l	2.99E+00
gene41876	67988	NC_000084.6	Tmx3	2.99E+00
gene36656	56438	NC_000081.6	Rbx1	2.99E+00
gene22575	77862	NC_000075.6	Thyn1	2.99E+00
gene28674	217166	NC_000077.6	Nr1d1	2.99E+00
gene13134	66138	NC_000071.6	Wbscr22	2.99E+00
gene15983	1.1E+08	NC_000072.6	Gm17089	2.99E+00
gene34742	16475	NC_000080.6	Ajuba	2.99E+00
gene19880	665143	NC_000073.6	Gm7514	2.99E+00
gene31660	19720	NC_000079.6	Trim27	2.99E+00
gene17250	53607	NC_000073.6	Snrpa	2.99E+00
gene564	17936	NC_000067.6	Nab1	2.99E+00
gene42915	627889	NC_000085.6	Gm6807	2.99E+00
gene27379	57783	NC_000077.6	Tnip1	2.99E+00
gene6547	1E+08	NC_000069.6	Gm17887	2.99E+00
gene4838	56398	NC_000068.7	Chp1	2.98E+00

gene7562	18632	NC_000069.6	Pex11b	2.98E+00
gene4866	20402	NC_000068.7	Zfp106	2.98E+00
gene9134	19359	NC_000070.6	Rad23b	2.98E+00
gene36578	69833	NC_000081.6	Polr2f	2.98E+00
gene5378	109344	NC_000068.7	E130215H24Rik	2.98E+00
gene39529	66416	NC_000083.6	Ndufa7	2.98E+00
gene469	666642	NC_000067.6	Gm8210	2.98E+00
gene38511	68001	NC_000082.6	1110004E09Rik	2.98E+00
gene19633	29810	NC_000073.6	Bag3	2.98E+00
gene11232	670717	NC_000071.6	Gm10108	2.98E+00
gene37066	19082	NC_000081.6	Prkag1	2.98E+00
gene4976	15186	NC_000068.7	Hdc	2.98E+00
gene10930	1E+08	NC_000070.6	Gm20377	2.97E+00
gene29323	=Gene;ger	NC_000077.6	LOC102639982	2.97E+00
gene18424	18975	NC_000073.6	Polg	2.97E+00
gene37969	14314	NC_000082.6	Fstl1	2.97E+00
gene29434	19325	NC_000078.6	Rab10	2.97E+00
gene20499	114863	NC_000074.6	Prosc	2.96E+00
gene36652	70356	NC_000081.6	St13	2.96E+00
gene15179	17216	NC_000072.6	Mcm2	2.96E+00
gene23838	68861	NC_000075.6	1190002N15Rik	2.96E+00
gene35779	110960	NC_000081.6	Tars	2.96E+00
gene11795	13204	NC_000071.6	Dhx15	2.96E+00
gene15557	78752	NC_000072.6	Csgalnact2	2.96E+00
gene17261	233016	NC_000073.6	Blvrb	2.96E+00
gene10619	1E+08	NC_000070.6	Gm13056	2.96E+00
gene10464	14105	NC_000070.6	Srsf10	2.96E+00
gene7137	27371	NC_000069.6	Sh2d2a	2.96E+00
gene28773	66180	NC_000077.6	P3h4	2.96E+00
gene4517	621146	NC_000068.7	Gm13803	2.95E+00
gene6945	16649	NC_000069.6	Kpna4	2.95E+00
gene29062	1E+08	NC_000077.6	Gm11696	2.95E+00
gene12927	109305	NC_000071.6	Orai1	2.95E+00
gene39120	386612	NC_000083.6	Thoc6	2.95E+00
gene12738	117109	NC_000071.6	Pop5	2.94E+00
gene36185	22770	NC_000081.6	Zhx1	2.94E+00
gene12682	231633	NC_000071.6	Tmem119	2.94E+00
gene26999	78372	NC_000077.6	Snrnp25	2.94E+00
gene25583	11537	NC_000076.6	Cfd	2.94E+00
gene15550	98758	NC_000072.6	Hnrnpf	2.94E+00
gene36165	15124	NC_000081.6	Hba-ps3	2.93E+00
gene37496	17217	NC_000082.6	Mcm4	2.93E+00
gene3341	96979	NC_000068.7	Ptges2	2.93E+00
gene21930	72552	NC_000074.6	Hsdl1	2.93E+00
gene25414	14872	NC_000076.6	Gstt2	2.93E+00
gene24159	66167	NC_000075.6	Tma7	2.93E+00
gene26381	110962	NC_000076.6	Mbd6	2.93E+00
gene22023	67177	NC_000074.6	Cdt1	2.93E+00

gene19555	233887	NC_000073.6	Zfp553	2.93E+00
gene10167	66407	NC_000070.6	Mrps15	2.93E+00
gene14054	621427	NC_000072.6	Gm10244	2.93E+00
gene17189	232983	NC_000073.6	Cxcl17	2.93E+00
gene6737	69639	NC_000069.6	Exosc8	2.93E+00
gene34927	219135	NC_000080.6	Mtmr6	2.92E+00
gene21378	80750	NC_000074.6	N4bp1	2.92E+00
gene2443	98417	NC_000067.6	Cnih4	2.92E+00
gene28789	14534	NC_000077.6	Kat2a	2.92E+00
gene24422	59289	NC_000075.6	Ackr2	2.92E+00
gene37271	1.1E+08	NC_000081.6	Gm41406	2.92E+00
gene25921	15109	NC_000076.6	Hal	2.92E+00
gene35237	13885	NC_000080.6	Esd	2.92E+00
gene23289	1.1E+08	NC_000075.6	Gm39347	2.91E+00
gene30433	217684	NC_000078.6	Susd6	2.91E+00
gene25440	76964	NC_000076.6	2610028H24Rik	2.91E+00
gene20963	66869	NC_000074.6	Zfp869	2.91E+00
gene12021	57357	NC_000071.6	Srd5a3	2.91E+00
gene10480	22704	NC_000070.6	Zfp46	2.91E+00
gene14024	78781	NC_000072.6	Zc3hav1	2.91E+00
gene39647	1.1E+08	NC_000083.6	Gm20522	2.91E+00
gene4434	104418	NC_000068.7	Dgkz	2.91E+00
gene1605	213417	NC_000067.6	Klhdc8a	2.91E+00
gene25618	70335	NC_000076.6	Reep6	2.91E+00
gene18442	70420	NC_000073.6	Arpin	2.91E+00
gene3029	27377	NC_000068.7	Yme1l1	2.91E+00
gene4404	68427	NC_000068.7	Slc39a13	2.90E+00
gene21279	70134	NC_000074.6	2210011C24Rik	2.90E+00
gene22250	17381	NC_000075.6	Mmp12	2.90E+00
gene39003	381065	NC_000083.6	Gm5145	2.90E+00
gene16778	243846	NC_000073.6	Ccdc9	2.90E+00
gene22699	74144	NC_000075.6	Robo4	2.90E+00
gene12446	76074	NC_000071.6	Gbp8	2.90E+00
gene36662	19387	NC_000081.6	Rangap1	2.90E+00
gene6681	67746	NC_000069.6	4930577N17Rik	2.90E+00
gene7499	107701	NC_000069.6	Sf3b4	2.90E+00
gene25584	216154	NC_000076.6	Med16	2.90E+00
gene12674	14747	NC_000071.6	Cmklr1	2.90E+00
gene2611	226849	NC_000067.6	Ppp2r5a	2.90E+00
gene34804	105446	NC_000080.6	Gmpr2	2.90E+00
gene4713	99010	NC_000068.7	Lpcat4	2.89E+00
gene22446	235036	NC_000075.6	Ppan	2.89E+00
gene41932	108995	NC_000085.6	Tbc1d10c	2.89E+00
gene19563	269997	NC_000073.6	Zfp747	2.89E+00
gene15160	1E+08	NC_000072.6	Gm34872	2.89E+00
gene11128	64945	NC_000071.6	Cldn12	2.89E+00
gene1005	75734	NC_000067.6	Mff	2.89E+00
gene16901	67091	NC_000073.6	Trappc6a	2.89E+00

gene24080	56032	NC_000075.6	Nprl2	2.89E+00
gene11379	269633	NC_000071.6	Wdr86	2.89E+00
gene32692	665524	NC_000079.6	Tubb2a-ps1	2.89E+00
gene12831	21386	NC_000071.6	Tbx3	2.89E+00
gene1039	1E+08	NC_000067.6	Gm35498	2.89E+00
gene40584	18701	NC_000083.6	Pigf	2.89E+00
gene40333	22666	NC_000083.6	Zbtb14	2.89E+00
gene14884	66700	NC_000072.6	Chmp3	2.88E+00
gene32426	20963	NC_000079.6	Syk	2.88E+00
gene23229	78323	NC_000075.6	Fam219b	2.88E+00
gene6955	19952	NC_000069.6	Rpl32-ps	2.88E+00
gene21769	384864	NC_000074.6	Gm1943	2.88E+00
gene13317	66506	NC_000071.6	Psmg3	2.88E+00
gene9316	671064	NC_000070.6	Gm11225	2.88E+00
gene22703	57276	NC_000075.6	Vsig2	2.88E+00
gene39967	66591	NC_000083.6	Mad2l1bp	2.88E+00
gene19990	17955	NC_000073.6	Nap1l4	2.88E+00
gene1876	13211	NC_000067.6	Dhx9	2.88E+00
gene39999	224824	NC_000083.6	Pex6	2.87E+00
gene37812	224088	NC_000082.6	Atp13a3	2.87E+00
gene15778	72231	NC_000072.6	1700018A23Rik	2.87E+00
gene4580	15259	NC_000068.7	Hipk3	2.87E+00
gene20985	19727	NC_000074.6	Rfxank	2.87E+00
gene37223	60315	NC_000081.6	Myg1	2.87E+00
gene41981	381199	NC_000085.6	Tmem151a	2.87E+00
gene6194	229011	NC_000068.7	Samd10	2.87E+00
gene25621	237400	NC_000076.6	Mex3d	2.87E+00
gene25398	68778	NC_000076.6	Gucd1	2.87E+00
gene10568	110198	NC_000070.6	Akr7a5	2.87E+00
gene23955	77853	NC_000075.6	Msl2	2.87E+00
gene14979	56284	NC_000072.6	Mrpl19	2.87E+00
gene28912	18107	NC_000077.6	Nmt1	2.87E+00
gene1609	68875	NC_000067.6	Tmcc2	2.86E+00
gene37309	1.1E+08	NC_000082.6	Gm41410	2.86E+00
gene1072	227327	NC_000067.6	B3gnt7	2.86E+00
gene7595	20333	NC_000069.6	Sec22b	2.86E+00
gene23231	24044	NC_000075.6	Scamp2	2.86E+00
gene9003	666379	NC_000070.6	Gm12445	2.85E+00
gene5705	228859	NC_000068.7	Fitm2	2.85E+00
gene34807	56187	NC_000080.6	Rabggta	2.85E+00
gene39348	68776	NC_000083.6	Taf11	2.85E+00
gene33760	66231	NC_000080.6	Thoc7	2.85E+00
gene39618	193736	NC_000083.6	Zbtb12	2.85E+00
gene28569	74479	NC_000077.6	Snx11	2.84E+00
gene20968	170759	NC_000074.6	Atp13a1	2.84E+00
gene22495	235041	NC_000075.6	Kank2	2.84E+00
gene27293	66397	NC_000077.6	Sar1b	2.84E+00
gene32469	28126	NC_000079.6	Nop16	2.84E+00

gene37425	69918	NC_000082.6	2610020C07Rik	2.84E+00
gene3525	66489	NC_000068.7	Rpl35	2.84E+00
gene118	665391	NC_000067.6	Gm7617	2.84E+00
gene24939	67371	NC_000076.6	Gtf3c6	2.84E+00
gene39474	320484	NC_000083.6	Rasal3	2.84E+00
gene19687	66885	NC_000073.6	Acadsb	2.84E+00
gene11037	21936	NC_000070.6	Tnfrsf18	2.84E+00
gene40222	21950	NC_000083.6	Tnfsf9	2.83E+00
gene28818	68107	NC_000077.6	Cntd1	2.83E+00
gene35270	67467	NC_000080.6	Gpalpp1	2.83E+00
gene12057	231329	NC_000071.6	Polr2b	2.83E+00
gene7186	94232	NC_000069.6	Ubqln4	2.83E+00
gene26163	216350	NC_000076.6	Tspan8	2.83E+00
gene20840	60406	NC_000074.6	Sap30	2.83E+00
gene39769	22670	NC_000083.6	Trim26	2.83E+00
gene5436	20667	NC_000068.7	Sox12	2.83E+00
gene7141	229503	NC_000069.6	Rrnad1	2.83E+00
gene12889	16923	NC_000071.6	Sh2b3	2.83E+00
gene1572	16865	NC_000067.6	Eif2d	2.83E+00
gene14931	56451	NC_000072.6	Suclg1	2.83E+00
gene10610	17150	NC_000070.6	Mfap2	2.82E+00
gene3243	26569	NC_000068.7	Slc27a4	2.82E+00
gene19818	11501	NC_000073.6	Adam8	2.82E+00
gene22694	56857	NC_000075.6	Slc37a2	2.82E+00
gene1454	64143	NC_000067.6	Ralb	2.82E+00
gene5994	56491	NC_000068.7	Vapb	2.82E+00
gene19923	1E+08	NC_000073.6	Gm16982	2.82E+00
gene38204	12892	NC_000082.6	Cpox	2.82E+00
gene32495	17121	NC_000079.6	Mxd3	2.82E+00
gene27731	71998	NC_000077.6	Slc25a35	2.82E+00
gene18404	67308	NC_000073.6	Mrpl46	2.82E+00
gene27186	432555	NC_000077.6	Gm5431	2.82E+00
gene4884	329504	NC_000068.7	Lcmt2	2.82E+00
gene1535	67812	NC_000067.6	Ubxn4	2.81E+00
gene18768	109689	NC_000073.6	Arrb1	2.81E+00
gene4801	214239	NC_000068.7	A430105I19Rik	2.81E+00
gene39710	52040	NC_000083.6	Ppp1r10	2.81E+00
gene27786	23879	NC_000077.6	Fxr2	2.81E+00
gene871	21961	NC_000067.6	Tns1	2.81E+00
gene24106	69398	NC_000075.6	Cdhr4	2.81E+00
gene21059	234395	NC_000074.6	Ushbp1	2.81E+00
gene40452	19106	NC_000083.6	Eif2ak2	2.81E+00
gene33867	17423	NC_000080.6	Ndst2	2.80E+00
gene32104	19766	NC_000079.6	Ripk1	2.80E+00
gene20504	71082	NC_000074.6	4933416M07Rik	2.80E+00
gene28827	217201	NC_000077.6	Rundc1	2.80E+00
gene24369	13051	NC_000075.6	Cx3cr1	2.80E+00
gene3258	70296	NC_000068.7	Tbc1d13	2.80E+00

gene155	108212	NC_000067.6	Rbm6-ps1	2.80E+00
gene30296	19042	NC_000078.6	Ppm1a	2.80E+00
gene14376	101214	NC_000072.6	Tra2a	2.80E+00
gene42184	1E+08	NC_000085.6	Gm36629	2.80E+00
gene36577	27660	NC_000081.6	1700088E04Rik	2.80E+00
gene26691	52513	NC_000077.6	Ddx56	2.80E+00
gene39994	66515	NC_000083.6	Cul7	2.79E+00
gene10048	73172	NC_000070.6	Exo5	2.79E+00
gene42940	68431	NC_000085.6	Fbxl15	2.79E+00
gene10362	14191	NC_000070.6	Fgr	2.79E+00
gene15309	232286	NC_000072.6	Tmf1	2.79E+00
gene16914	53333	NC_000073.6	Tomm40	2.79E+00
gene12024	21982	NC_000071.6	Tmem165	2.79E+00
gene37864	13026	NC_000082.6	Pcyt1a	2.79E+00
gene22940	15288	NC_000075.6	Hmbs	2.79E+00
gene39628	27756	NC_000083.6	Lsm2	2.78E+00
gene286	213711	NC_000067.6	Gm4785	2.78E+00
gene19441	28018	NC_000073.6	Ubfd1	2.78E+00
gene5154	110911	NC_000068.7	Cds2	2.78E+00
gene32464	73666	NC_000079.6	Thoc3	2.78E+00
gene16796	97387	NC_000073.6	Strn4	2.78E+00
gene39524	224703	NC_000083.6	March2	2.78E+00
gene26293	192678	NC_000076.6	Rassf3	2.78E+00
gene38182	28185	NC_000082.6	Tomm70a	2.78E+00
gene9199	230257	NC_000070.6	Ptbp3	2.78E+00
gene27575	24083	NC_000077.6	Natd1	2.78E+00
gene11578	59003	NC_000071.6	Maea	2.78E+00
gene10562	433771	NC_000070.6	Minos1	2.78E+00
gene1784	20822	NC_000067.6	Trove2	2.78E+00
gene33864	76133	NC_000080.6	6230400D17Rik	2.78E+00
gene27174	211007	NC_000077.6	Trim41	2.77E+00
gene25376	52696	NC_000076.6	Zwint	2.77E+00
gene6334	229096	NC_000069.6	Ythdf3	2.77E+00
gene19020	20128	NC_000073.6	Trim30a	2.77E+00
gene28068	67201	NC_000077.6	Glod4	2.77E+00
gene358	20353	NC_000067.6	Sema4c	2.77E+00
gene8183	11522	NC_000069.6	Adh1	2.77E+00
gene22361	66917	NC_000075.6	Chordc1	2.77E+00
gene28064	116972	NC_000077.6	Fam57a	2.77E+00
gene176	226901	NC_000067.6	Gm4849	2.77E+00
gene15253	67678	NC_000072.6	Lsm3	2.77E+00
gene4571	1E+08	NC_000068.7	Gm38515	2.77E+00
gene23755	72655	NC_000075.6	Snhg5	2.76E+00
gene16179	108098	NC_000072.6	Med21	2.76E+00
gene42141	623030	NC_000085.6	Gm6386	2.76E+00
gene42105	68323	NC_000085.6	Nudt22	2.76E+00
gene28557	15413	NC_000077.6	Hoxb5	2.76E+00
gene36730	68607	NC_000081.6	Serhl	2.76E+00

gene3185	381356	NC_000068.7	Cacfd1	2.76E+00
gene12453	634650	NC_000071.6	Gbp11	2.76E+00
gene1298	227393	NC_000067.6	Gm1833	2.76E+00
gene11587	14184	NC_000071.6	Fgfr3	2.76E+00
gene28154	195040	NC_000077.6	Tmem199	2.76E+00
gene5339	74533	NC_000068.7	Gzf1	2.76E+00
gene40200	224907	NC_000083.6	Dus3l	2.75E+00
gene40905	225280	NC_000084.6	Ino80c	2.75E+00
gene21323	270076	NC_000074.6	Gcdh	2.75E+00
gene26623	67465	NC_000077.6	Sf3a1	2.75E+00
gene22493	16835	NC_000075.6	Ldlr	2.75E+00
gene36362	17068	NC_000081.6	Ly6d	2.75E+00
gene30760	319760	NC_000078.6	D130020L05Rik	2.75E+00
gene41965	70605	NC_000085.6	Zdhhc24	2.75E+00
gene9777	230598	NC_000070.6	Nrd1	2.75E+00
gene12595	72542	NC_000071.6	Pgam5	2.75E+00
gene27031	216643	NC_000077.6	Gabrp	2.75E+00
gene24841	1E+08	NC_000076.6	Gm20300	2.74E+00
gene608	212679	NC_000067.6	Mars2	2.74E+00
gene41616	75286	NC_000084.6	4930549G23Rik	2.74E+00
gene41777	68731	NC_000084.6	Rbfa	2.74E+00
gene12948	66593	NC_000071.6	Diablo	2.74E+00
gene21686	56513	NC_000074.6	Pard6a	2.74E+00
gene37375	69053	NC_000082.6	1810013L24Rik	2.74E+00
gene42582	433238	NC_000085.6	Gm5518	2.74E+00
gene5558	56407	NC_000068.7	Trpc4ap	2.74E+00
gene26615	103655	NC_000077.6	Sec14l4	2.74E+00
gene9769	319965	NC_000070.6	Cc2d1b	2.74E+00
gene386	107734	NC_000067.6	Mrpl30	2.74E+00
gene23595	70082	NC_000075.6	Lysmd2	2.74E+00
gene35609	1E+08	NC_000080.6	Gm26679	2.74E+00
gene18277	1E+08	NC_000073.6	Gm33442	2.74E+00
gene39288	106581	NC_000083.6	Fam234a	2.74E+00
gene23	619829	NC_000067.6	Gm6104	2.74E+00
gene31125	66174	NC_000078.6	Nudt14	2.74E+00
gene7443	69269	NC_000069.6	Scnm1	2.73E+00
gene11739	14181	NC_000071.6	Fgfbp1	2.73E+00
gene23618	68801	NC_000075.6	Elovl5	2.73E+00
gene10258	230766	NC_000070.6	Fam167b	2.73E+00
gene17881	75613	NC_000073.6	Med25	2.73E+00
gene16809	19060	NC_000073.6	Ppp5c	2.73E+00
gene28136	20926	NC_000077.6	Supt6	2.73E+00
gene29576	104721	NC_000078.6	Ddx1	2.73E+00
gene36237	17869	NC_000081.6	Myc	2.73E+00
gene21008	22186	NC_000074.6	Uba52	2.73E+00
gene31687	432731	NC_000079.6	Zscan26	2.73E+00
gene40170	106759	NC_000083.6	Ticam1	2.73E+00
gene1872	667795	NC_000067.6	Gm8818	2.72E+00

gene28673	21833	NC_000077.6	Thra	2.72E+00
gene16267	1E+08	NC_000073.6	Gm15927	2.72E+00
gene2056	20343	NC_000067.6	Sell	2.72E+00
gene18679	58238	NC_000073.6	Fam181b	2.72E+00
gene42752	1E+08	NC_000085.6	Gm3875	2.72E+00
gene20063	14128	NC_000074.6	Fcer2a	2.72E+00
gene1913	1E+08	NC_000067.6	Gm30922	2.72E+00
gene36789	223739	NC_000081.6	5031439G07Rik	2.72E+00
gene19752	17345	NC_000073.6	Mki67	2.72E+00
gene17897	66394	NC_000073.6	Nosip	2.72E+00
gene21696	234699	NC_000074.6	Edc4	2.72E+00
gene604	19070	NC_000067.6	Mob4	2.72E+00
gene16035	66857	NC_000072.6	Plbd1	2.72E+00
gene28438	67468	NC_000077.6	Mmd	2.71E+00
gene24450	66202	NC_000075.6	1110059G10Rik	2.71E+00
gene5532	19383	NC_000068.7	Raly	2.71E+00
gene36097	432951	NC_000081.6	Gm5472	2.71E+00
gene26395	16971	NC_000076.6	Lrp1	2.71E+00
gene22106	69581	NC_000074.6	Rhou	2.71E+00
gene3589	109129	NC_000068.7	Mmadhc	2.71E+00
gene5186	433478	NC_000068.7	Gm14038	2.71E+00
gene13490	76366	NC_000071.6	Mtif3	2.71E+00
gene30512	104776	NC_000078.6	Aldh6a1	2.71E+00
gene3063	56876	NC_000068.7	Nsmf	2.71E+00
gene5620	20779	NC_000068.7	Src	2.71E+00
gene14518	27055	NC_000072.6	Fkbp9	2.70E+00
gene40583	104215	NC_000083.6	Rhoq	2.70E+00
gene2373	11566	NC_000067.6	Adss	2.70E+00
gene26890	268390	NC_000077.6	Ahsa2	2.70E+00
gene37649	11773	NC_000082.6	Ap2m1	2.70E+00
gene26586	216505	NC_000077.6	Pik3ip1	2.70E+00
gene2420	170760	NC_000067.6	Acbd3	2.70E+00
gene37165	56149	NC_000081.6	Grasp	2.70E+00
gene32272	218203	NC_000079.6	Mylip	2.70E+00
gene3182	227656	NC_000068.7	Rexo4	2.70E+00
gene14014	665102	NC_000072.6	Gm7492	2.70E+00
gene40081	1E+08	NC_000083.6	Gm18735	2.70E+00
gene26611	64934	NC_000077.6	Pes1	2.69E+00
gene23398	57874	NC_000075.6	Hacd3	2.69E+00
gene19435	20278	NC_000073.6	Scnn1g	2.69E+00
gene37604	72307	NC_000082.6	2510002D24Rik	2.69E+00
gene30655	620915	NC_000078.6	Gm6190	2.69E+00
gene2232	56009	NC_000067.6	Alyref2	2.69E+00
gene39706	69662	NC_000083.6	2310061I04Rik	2.69E+00
gene26940	216618	NC_000077.6	Cfap36	2.69E+00
gene25421	333670	NC_000076.6	Gm867	2.69E+00
gene28126	22032	NC_000077.6	Traf4	2.69E+00
gene15457	171095	NC_000072.6	Il17rc	2.69E+00

gene27583	216825	NC_000077.6	Usp22	2.69E+00
gene39171	56716	NC_000083.6	Mlst8	2.69E+00
gene28255	276950	NC_000077.6	Slfn8	2.69E+00
gene24089	15983	NC_000075.6	Ifrd2	2.69E+00
gene38151	436421	NC_000082.6	Gm5769	2.69E+00
gene29513	80913	NC_000078.6	Pum2	2.69E+00
gene32329	218236	NC_000079.6	Fam120a	2.68E+00
gene36589	223693	NC_000081.6	Tmem184b	2.68E+00
gene29359	70317	NC_000077.6	Arl16	2.68E+00
gene7435	78266	NC_000069.6	Zfp687	2.68E+00
gene24285	102545	NC_000075.6	Cmtm7	2.68E+00
gene9746	74098	NC_000070.6	0610037L13Rik	2.68E+00
gene10401	20111	NC_000070.6	Rps6ka1	2.68E+00
gene22156	53424	NC_000074.6	Tsnax	2.68E+00
gene28350	76892	NC_000077.6	Rnft1	2.67E+00
gene3113	227624	NC_000068.7	Rabl6	2.67E+00
gene11888	71778	NC_000071.6	Klhl5	2.67E+00
gene185	17215	NC_000067.6	Mcm3	2.67E+00
gene3409	22029	NC_000068.7	Traf1	2.67E+00
gene2459	98386	NC_000067.6	Lbr	2.67E+00
gene40058	18044	NC_000083.6	Nfya	2.67E+00
gene6940	70099	NC_000069.6	Smc4	2.67E+00
gene21286	212123	NC_000074.6	Dcaf15	2.67E+00
gene10903	21942	NC_000070.6	Tnfrsf9	2.67E+00
gene27632	94092	NC_000077.6	Trim16	2.67E+00
gene1449	16324	NC_000067.6	Inhbb	2.67E+00
gene13541	15289	NC_000071.6	Hmgb1	2.66E+00
gene37348	170644	NC_000082.6	Ubn1	2.66E+00
gene8355	51886	NC_000069.6	Fubp1	2.66E+00
gene15777	66108	NC_000072.6	Ndufa9	2.66E+00
gene34443	78416	NC_000080.6	Rnase6	2.66E+00
gene21203	24015	NC_000074.6	Abce1	2.66E+00
gene35160	219158	NC_000080.6	Ccar2	2.66E+00
gene20444	11502	NC_000074.6	Adam9	2.66E+00
gene42351	433224	NC_000085.6	Gm5512	2.66E+00
gene28026	15248	NC_000077.6	Hic1	2.65E+00
gene216	70155	NC_000067.6	Ogfrl1	2.65E+00
gene3160	67512	NC_000068.7	Agpat2	2.65E+00
gene2435	13849	NC_000067.6	Ephx1	2.65E+00
gene12726	231655	NC_000071.6	Oasl1	2.65E+00
gene19530	13446	NC_000073.6	Doc2a	2.65E+00
gene13298	68033	NC_000071.6	Cox19	2.65E+00
gene19671	101476	NC_000073.6	Plekha1	2.64E+00
gene23210	76794	NC_000075.6	Man2c1os	2.64E+00
gene6690	19338	NC_000069.6	Rab33b	2.64E+00
gene21898	234776	NC_000074.6	Atmin	2.64E+00
gene10111	56309	NC_000070.6	Mycbp	2.64E+00
gene10675	242736	NC_000070.6	Pramef8	2.64E+00

gene2610	66950	NC_000067.6	Tmem206	2.64E+00
gene5092	321014	NC_000068.7	4930473A02Rik	2.64E+00
gene11523	17527	NC_000071.6	Mpv17	2.64E+00
gene33203	320806	NC_000079.6	Gfm2	2.64E+00
gene8423	69207	NC_000069.6	Srsf11	2.64E+00
gene27397	27041	NC_000077.6	G3bp1	2.63E+00
gene25399	67332	NC_000076.6	Snrpd3	2.63E+00
gene37928	69544	NC_000082.6	Wdr5b	2.63E+00
gene25625	21423	NC_000076.6	Tcf3	2.63E+00
gene27080	432549	NC_000077.6	Gm12138	2.63E+00
gene42458	225995	NC_000085.6	D030056L22Rik	2.63E+00
gene6405	675593	NC_000069.6	Spin3-ps	2.63E+00
gene9069	109331	NC_000070.6	Rnf20	2.63E+00
gene12774	80888	NC_000071.6	Hspb8	2.63E+00
gene17204	27998	NC_000073.6	Exosc5	2.63E+00
gene33806	69721	NC_000080.6	Nkiras1	2.63E+00
gene16832	384560	NC_000073.6	Gm5322	2.63E+00
gene21809	101943	NC_000074.6	Sf3b3	2.63E+00
gene7484	66292	NC_000069.6	Mrps21	2.62E+00
gene7251	23897	NC_000069.6	Hax1	2.62E+00
gene29234	114886	NC_000077.6	Cygb	2.62E+00
gene15613	24110	NC_000072.6	Usp18	2.62E+00
gene35187	13449	NC_000080.6	Dok2	2.62E+00
gene6226	19302	NC_000069.6	Pex2	2.62E+00
gene17472	56188	NC_000073.6	Fxyd1	2.62E+00
gene40241	545216	NC_000083.6	Gm5815	2.62E+00
gene5420	73847	NC_000068.7	Fam110a	2.62E+00
gene12960	29816	NC_000071.6	Hip1r	2.62E+00
gene11035	22163	NC_000070.6	Tnfrsf4	2.62E+00
gene9637	16451	NC_000070.6	Jak1	2.62E+00
gene39541	57315	NC_000083.6	Wdr46	2.62E+00
gene4002	269292	NC_000068.7	Gm13676	2.61E+00
gene12375	68735	NC_000071.6	Mrps18c	2.61E+00
gene1825	108989	NC_000067.6	Tpr	2.61E+00
gene18825	56018	NC_000073.6	Stard10	2.61E+00
gene41024	19348	NC_000084.6	Kif20a	2.61E+00
gene42074	26412	NC_000085.6	Map4k2	2.61E+00
gene22650	235169	NC_000075.6	Foxred1	2.61E+00
gene3338	68379	NC_000068.7	Ciz1	2.61E+00
gene31936	68024	NC_000079.6	Hist1h2bc	2.61E+00
gene12609	56361	NC_000071.6	Pus1	2.61E+00
gene41887	1E+08	NC_000085.6	Gm36532	2.61E+00
gene13292	18590	NC_000071.6	Pdgfa	2.61E+00
gene27060	104458	NC_000077.6	Rars	2.61E+00
gene23754	56403	NC_000075.6	Syncrip	2.61E+00
gene8471	242291	NC_000070.6	Impad1	2.61E+00
gene7963	214137	NC_000069.6	Arhgap29	2.61E+00
gene15450	101206	NC_000072.6	Tada3	2.60E+00

gene14220	54324	NC_000072.6	Arhgef5	2.60E+00
gene19579	68961	NC_000073.6	Phkg2	2.60E+00
gene24050	27966	NC_000075.6	Rrp9	2.60E+00
gene4405	1.1E+08	NC_000068.7	Gm39875	2.60E+00
gene37580	27801	NC_000082.6	Zdhhc8	2.60E+00
gene35357	629794	NC_000080.6	Gm7004	2.60E+00
gene38877	72057	NC_000083.6	Phf10	2.60E+00
gene15874	27007	NC_000072.6	Klrk1	2.60E+00
gene24473	17281	NC_000075.6	Fyco1	2.60E+00
gene41190	66839	NC_000084.6	0610009O20Rik	2.60E+00
gene9151	230234	NC_000070.6	Fam206a	2.60E+00
gene22150	14712	NC_000074.6	Gnpat	2.60E+00
gene12958	68184	NC_000071.6	Denr	2.59E+00
gene20106	436026	NC_000074.6	Gm5739	2.59E+00
gene15116	67855	NC_000072.6	Asprv1	2.59E+00
gene23400	74388	NC_000075.6	Dpp8	2.59E+00
gene72	70675	NC_000067.6	Vcpip1	2.59E+00
gene24475	23832	NC_000075.6	Xcr1	2.59E+00
gene7816	72023	NC_000069.6	Cyb561d1	2.59E+00
gene27129	216705	NC_000077.6	Clint1	2.59E+00
gene33173	66549	NC_000079.6	Aggf1	2.59E+00
gene42967	72691	NC_000085.6	Calhm2	2.58E+00
gene22153	112405	NC_000074.6	Egln1	2.58E+00
gene22631	23871	NC_000075.6	Ets1	2.58E+00
gene29390	209318	NC_000077.6	Gps1	2.58E+00
gene27818	216860	NC_000077.6	Neurl4	2.58E+00
gene12012	18595	NC_000071.6	Pdgfra	2.58E+00
gene42924	76055	NC_000085.6	Mgea5	2.58E+00
gene24119	66257	NC_000075.6	Nicn1	2.58E+00
gene30788	66622	NC_000078.6	Ubr7	2.57E+00
gene39998	21770	NC_000083.6	Ppp2r5d	2.57E+00
gene36776	223732	NC_000081.6	Ldoc1l	2.57E+00
gene35912	1E+08	NC_000081.6	Snhg18	2.57E+00
gene4393	56428	NC_000068.7	Mtch2	2.57E+00
gene28253	237886	NC_000077.6	Slfn9	2.57E+00
gene28769	15114	NC_000077.6	Hap1	2.56E+00
gene45469	26900	NC_000087.7	Ddx3y	2.56E+00
gene41571	240354	NC_000084.6	Malt1	2.56E+00
gene23349	76524	NC_000075.6	Cln6	2.56E+00
gene2148	19736	NC_000067.6	Rgs4	2.56E+00
gene37528	26413	NC_000082.6	Mapk1	2.56E+00
gene36686	72049	NC_000081.6	Tnfrsf13c	2.56E+00
gene3323	1E+08	NC_000068.7	Gm13609	2.56E+00
gene12205	68098	NC_000071.6	Rchy1	2.56E+00
gene34220	ene=LOC1	NC_000080.6	LOC102639170	2.56E+00
gene24060	21767	NC_000075.6	Tex264	2.56E+00
gene1174	12778	NC_000067.6	Ackr3	2.56E+00
gene8846	18391	NC_000070.6	Sigmar1	2.55E+00

gene20424	109145	NC_000074.6	Gins4	2.55E+00
gene36881	70113	NC_000081.6	Odf3b	2.55E+00
gene25179	52717	NC_000076.6	Anapc16	2.55E+00
gene6123	241846	NC_000068.7	Lsm14b	2.55E+00
gene15639	11810	NC_000072.6	Apobec1	2.54E+00
gene40520	106522	NC_000083.6	Pkdcc	2.54E+00
gene34814	1E+08	NC_000080.6	Gm32092	2.54E+00
gene12714	67642	NC_000071.6	4930515G01Rik	2.54E+00
gene14495	353172	NC_000072.6	Gars	2.54E+00
gene40456	12607	NC_000083.6	Cebpz	2.54E+00
gene36085	223527	NC_000081.6	Eny2	2.54E+00
gene13405	231874	NC_000071.6	Ccz1	2.54E+00
gene12700	117146	NC_000071.6	Ube3b	2.54E+00
gene31686	=Gene;ger	NC_000079.6	LOC105245359	2.54E+00
gene5829	383774	NC_000068.7	Gm11470	2.54E+00
gene21122	27967	NC_000074.6	Cherp	2.54E+00
gene28650	59045	NC_000077.6	Stard3	2.54E+00
gene39250	67563	NC_000083.6	Narfl	2.54E+00
gene41005	69749	NC_000084.6	Epb41l4aos	2.54E+00
gene30396	11847	NC_000078.6	Arg2	2.54E+00
gene13073	109900	NC_000071.6	Asl	2.53E+00
gene23240	102414	NC_000075.6	Clk3	2.53E+00
gene30211	69554	NC_000078.6	Klhdc2	2.53E+00
gene10181	236511	NC_000070.6	ago-01	2.53E+00
gene23589	50772	NC_000075.6	Mapk6	2.53E+00
gene27337	20362	NC_000077.6	sep-08	2.53E+00
gene21407	26992	NC_000074.6	Brd7	2.53E+00
gene21339	68628	NC_000074.6	Fbxw9	2.53E+00
gene1144	381280	NC_000067.6	Hjurp	2.53E+00
gene29219	217337	NC_000077.6	Srp68	2.53E+00
gene1957	69953	NC_000067.6	2810025M15Rik	2.53E+00
gene29233	217344	NC_000077.6	Rhbdf2	2.53E+00
gene27565	237781	NC_000077.6	Mief2	2.52E+00
gene7720	20501	NC_000069.6	Slc16a1	2.52E+00
gene15126	14583	NC_000072.6	Gfpt1	2.52E+00
gene12860	246727	NC_000071.6	Oas3	2.52E+00
gene2222	21945	NC_000067.6	Dedd	2.52E+00
gene27587	11671	NC_000077.6	Aldh3a2	2.52E+00
gene39596	54397	NC_000083.6	Ppt2	2.52E+00
gene28610	246198	NC_000077.6	Mllt6	2.52E+00
gene35171	67065	NC_000080.6	Polr3d	2.52E+00
gene39281	60455	NC_000083.6	Tmem8	2.52E+00
gene19887	24058	NC_000073.6	Sigirr	2.52E+00
gene39992	74764	NC_000083.6	Klc4	2.52E+00
gene40110	271457	NC_000083.6	Rab5a	2.51E+00
gene25252	432467	NC_000076.6	Hnrnph3	2.51E+00
gene3225	269252	NC_000068.7	Gtf3c4	2.51E+00
gene19088	11785	NC_000073.6	Apbb1	2.51E+00

gene7051	53317	NC_000069.6	Plrg1	2.51E+00
gene6554	50911	NC_000069.6	Exosc9	2.51E+00
gene25356	21780	NC_000076.6	Tfam	2.51E+00
gene5842	263876	NC_000068.7	Spata2	2.51E+00
gene20728	142980	NC_000074.6	Tlr3	2.51E+00
gene29753	194655	NC_000078.6	Klf11	2.51E+00
gene42157	83673	NC_000085.6	Snhg1	2.51E+00
gene29691	16413	NC_000078.6	Itgb1bp1	2.51E+00
gene39528	54127	NC_000083.6	Rps28	2.51E+00
gene17206	232987	NC_000073.6	B9d2	2.51E+00
gene23089	68721	NC_000075.6	1110032A03Rik	2.51E+00
gene11007	12537	NC_000070.6	Cdk11b	2.51E+00
gene19261	67150	NC_000073.6	Rnf141	2.51E+00
gene42634	19211	NC_000085.6	Pten	2.50E+00
gene38522	1E+08	NC_000082.6	Gm36587	2.50E+00
gene35073	69303	NC_000080.6	1700001G11Rik	2.50E+00
gene25505	67884	NC_000076.6	1810043G02Rik	2.50E+00
gene36474	73062	NC_000081.6	Ppp1r16a	2.50E+00
gene30241	110095	NC_000078.6	Pygl	2.50E+00
gene32606	214290	NC_000079.6	Zcchc6	2.50E+00
gene29064	13929	NC_000077.6	Amz2	2.50E+00
gene21527	114255	NC_000074.6	Dok4	2.50E+00
gene36567	1E+08	NC_000081.6	Gm10865	2.50E+00
gene2156	16589	NC_000067.6	Uhmk1	2.50E+00
gene29281	74451	NC_000077.6	Pgs1	2.50E+00
gene17190	434148	NC_000073.6	Gm5587	2.50E+00
gene3151	227648	NC_000068.7	Sec16a	2.50E+00
gene34778	11766	NC_000080.6	Ap1g2	2.50E+00
gene7889	64378	NC_000069.6	Gpr88	2.49E+00
gene29950	15982	NC_000078.6	Ifrd1	2.49E+00
gene13296	67604	NC_000071.6	Get4	2.49E+00
gene36574	223690	NC_000081.6	Ankrd54	2.49E+00
gene10468	15356	NC_000070.6	Hmgcl	2.49E+00
gene21647	12400	NC_000074.6	Cbfb	2.49E+00
gene2649	ene=LOC1	NC_000067.6	LOC108167768	2.49E+00
gene14428	71982	NC_000072.6	Snx10	2.49E+00
gene9665	71148	NC_000070.6	Mier1	2.49E+00
gene30477	19164	NC_000078.6	Psen1	2.49E+00
gene22323	56503	NC_000075.6	Ankrd49	2.49E+00
gene26676	56012	NC_000077.6	Pgam2	2.49E+00
gene29226	319370	NC_000077.6	Ubald2	2.49E+00
gene34070	665601	NC_000080.6	Gm7707	2.49E+00
gene10520	230857	NC_000070.6	Ece1	2.48E+00
gene19911	68267	NC_000073.6	Slc25a22	2.48E+00
gene41697	52538	NC_000084.6	Acaa2	2.48E+00
gene30486	171210	NC_000078.6	Acot2	2.48E+00
gene42493	1E+08	NC_000085.6	Gm27151	2.48E+00
gene13118	56743	NC_000071.6	Lat2	2.48E+00

gene7749	70465	NC_000069.6	Wdr77	2.48E+00
gene6784	18645	NC_000069.6	Pfn2	2.48E+00
gene39514	1E+08	NC_000083.6	Zfp422-ps	2.48E+00
gene7441	21427	NC_000069.6	Vps72	2.48E+00
gene24293	68292	NC_000075.6	Stt3b	2.48E+00
gene21946	102193	NC_000074.6	Zdhhc7	2.47E+00
gene16642	65020	NC_000073.6	Zfp110	2.47E+00
gene5776	ene;gene=l	NC_000068.7	LOC105244413	2.47E+00
gene27600	71520	NC_000077.6	Grap	2.47E+00
gene31581	670994	NC_000079.6	Gm9512	2.47E+00
gene36415	66194	NC_000081.6	Pycrl	2.47E+00
gene17409	233065	NC_000073.6	Alkbh6	2.47E+00
gene9880	230654	NC_000070.6	Lrrc41	2.47E+00
gene8093	15107	NC_000069.6	Hadh	2.47E+00
gene27228	104625	NC_000077.6	Cnot6	2.46E+00
gene36601	74158	NC_000081.6	Josd1	2.46E+00
gene38581	109857	NC_000082.6	Cbr3	2.46E+00
gene21320	66590	NC_000074.6	Farsa	2.46E+00
gene38899	18567	NC_000083.6	Pdcd2	2.46E+00
gene650	66495	NC_000067.6	Ndufb3	2.46E+00
gene21275	18747	NC_000074.6	Prkaca	2.46E+00
gene5144	215653	NC_000068.7	Rassf2	2.46E+00
gene19886	56460	NC_000073.6	Pkp3	2.46E+00
gene36336	19245	NC_000081.6	Ptp4a3	2.46E+00
gene3271	227700	NC_000068.7	Sh3glb2	2.46E+00
gene22437	23988	NC_000075.6	Pin1	2.46E+00
gene36029	170769	NC_000081.6	Gm4740	2.45E+00
gene21929	56453	NC_000074.6	Mbtps1	2.45E+00
gene27804	74341	NC_000077.6	G630025P09Rik	2.45E+00
gene9916	242646	NC_000070.6	Tctex1d4	2.45E+00
gene15256	21366	NC_000072.6	Slc6a6	2.45E+00
gene5469	14239	NC_000068.7	Foxs1	2.45E+00
gene2489	108909	NC_000067.6	Aida	2.45E+00
gene7485	1E+08	NC_000069.6	C920021L13Rik	2.45E+00
gene37227	20683	NC_000081.6	Sp1	2.45E+00
gene29230	20698	NC_000077.6	Sphk1	2.45E+00
gene27455	1E+08	NC_000077.6	Gm21927	2.44E+00
gene27634	67510	NC_000077.6	Tvp23b	2.44E+00
gene4412	107986	NC_000068.7	Ddb2	2.44E+00
gene18788	67164	NC_000073.6	Lipt2	2.44E+00
gene10570	69902	NC_000070.6	Mrto4	2.44E+00
gene41038	1E+08	NC_000084.6	Gm6724	2.44E+00
gene545	442821	NC_000067.6	9330175M20Rik	2.44E+00
gene24591	74732	NC_000076.6	Stx11	2.44E+00
gene42817	74168	NC_000085.6	Zdhhc16	2.44E+00
gene7212	68521	NC_000069.6	Fam189b	2.44E+00
gene6738	66248	NC_000069.6	Alg5	2.44E+00
gene23206	1E+08	NC_000075.6	Gm10658	2.44E+00

gene24730	1E+08	NC_000076.6	Gm10824	2.44E+00
gene30348	1.1E+08	NC_000078.6	Gm40443	2.44E+00
gene22266	11797	NC_000075.6	Birc2	2.44E+00
gene24557	68652	NC_000076.6	Tab2	2.44E+00
gene11033	117592	NC_000070.6	B3galt6	2.44E+00
gene11290	212090	NC_000071.6	Tmem60	2.43E+00
gene2743	67229	NC_000068.7	Prpf18	2.43E+00
gene39223	22196	NC_000083.6	Ube2i	2.43E+00
gene1200	50880	NC_000067.6	Scly	2.43E+00
gene32930	407785	NC_000079.6	Ndufs6	2.43E+00
gene37212	223918	NC_000081.6	Spryd3	2.43E+00
gene895	57751	NC_000067.6	Rnf25	2.43E+00
gene13268	330216	NC_000071.6	Mblac1	2.43E+00
gene30363	104886	NC_000078.6	Rab15	2.43E+00
gene40787	1E+08	NC_000084.6	Gm16072	2.43E+00
gene10471	66193	NC_000070.6	Pithd1	2.43E+00
gene24086	15586	NC_000075.6	Hyal1	2.43E+00
gene25163	18451	NC_000076.6	P4ha1	2.43E+00
gene34039	70021	NC_000080.6	Nt5dc2	2.43E+00
gene15653	269799	NC_000072.6	Clec4a1	2.43E+00
gene40971	67158	NC_000084.6	Sft2d3	2.43E+00
gene3614	64685	NC_000068.7	Nmi	2.43E+00
gene21147	17218	NC_000074.6	Mcm5	2.42E+00
gene2242	12506	NC_000067.6	Cd48	2.42E+00
gene42741	1.1E+08	NC_000085.6	Gm41844	2.42E+00
gene7215	21827	NC_000069.6	Thbs3	2.42E+00
gene3721	71586	NC_000068.7	Ifih1	2.42E+00
gene23171	71999	NC_000075.6	Fbxo22	2.42E+00
gene5771	71971	NC_000068.7	Zswim1	2.42E+00
gene37849	224105	NC_000082.6	Pak2	2.42E+00
gene26699	77605	NC_000077.6	H2afv	2.42E+00
gene21343	67836	NC_000074.6	Wdr83	2.42E+00
gene19883	213002	NC_000073.6	Ifitm6	2.42E+00
gene30563	238328	NC_000078.6	Vash1	2.42E+00
gene42824	84095	NC_000085.6	Pi4k2a	2.42E+00
gene4532	22034	NC_000068.7	Traf6	2.42E+00
gene25283	1E+08	NC_000076.6	Gm31763	2.42E+00
gene20222	67031	NC_000074.6	Upf3a	2.42E+00
gene25509	50723	NC_000076.6	Icosl	2.42E+00
gene27118	74315	NC_000077.6	Rnf145	2.42E+00
gene41658	17191	NC_000084.6	Mbd2	2.41E+00
gene2014	1E+08	NC_000067.6	Gm7496	2.41E+00
gene26470	12566	NC_000076.6	Cdk2	2.41E+00
gene3851	20687	NC_000068.7	Sp3	2.41E+00
gene1273	21915	NC_000067.6	Dtymk	2.41E+00
gene38788	19317	NC_000083.6	Qk	2.41E+00
gene2434	208795	NC_000067.6	Tmem63a	2.41E+00
gene38414	12013	NC_000082.6	Bach1	2.41E+00

gene17883	11771	NC_000073.6	Ap2a1	2.41E+00
gene37697	239760	NC_000082.6	Gm4943	2.41E+00
gene17201	66349	NC_000073.6	Atp5sl	2.41E+00
gene37334	66626	NC_000082.6	Cdip1	2.41E+00
gene38770	ene=LOC1	NC_000083.6	LOC102637711	2.41E+00
gene12645	70118	NC_000071.6	Srrd	2.40E+00
gene4980	84092	NC_000068.7	Usp8	2.40E+00
gene27287	66089	NC_000077.6	Rmnd5b	2.40E+00
gene40187	224903	NC_000083.6	Safb	2.40E+00
gene19575	629159	NC_000073.6	1700008J07Rik	2.40E+00
gene2442	14185	NC_000067.6	Fgfr3-ps	2.40E+00
gene4660	623301	NC_000068.7	Gm13961	2.40E+00
gene39211	407831	NC_000083.6	Tmem204	2.40E+00
gene24108	27399	NC_000075.6	Ip6k1	2.40E+00
gene38052	66067	NC_000082.6	Gtpbp8	2.39E+00
gene7203	72296	NC_000069.6	Rusc1	2.39E+00
gene29186	14784	NC_000077.6	Grb2	2.39E+00
gene36553	17305	NC_000081.6	Mfng	2.39E+00
gene12887	72399	NC_000071.6	Brap	2.39E+00
gene17685	434168	NC_000073.6	Gm5590	2.39E+00
gene10318	57317	NC_000070.6	Srsf4	2.39E+00
gene24000	66663	NC_000075.6	Uba5	2.39E+00
gene23245	69459	NC_000075.6	Ubl7	2.39E+00
gene21282	50931	NC_000074.6	Il27ra	2.39E+00
gene36121	78581	NC_000081.6	Utp23	2.39E+00
gene6276	1E+08	NC_000069.6	Gm9833	2.39E+00
gene13064	12466	NC_000071.6	Cct6a	2.38E+00
gene16270	69757	NC_000073.6	Leng1	2.38E+00
gene23256	18854	NC_000075.6	Pml	2.38E+00
gene13392	670671	NC_000071.6	Gm9497	2.38E+00
gene42168	1E+08	NC_000085.6	Gm2518	2.38E+00
gene18104	1E+08	NC_000073.6	Fancf	2.38E+00
gene18827	69710	NC_000073.6	Arap1	2.38E+00
gene7530	18473	NC_000069.6	Pafah1b1-ps1	2.38E+00
gene33096	66074	NC_000079.6	Tmem167	2.38E+00
gene247	1E+08	NC_000067.6	Gm19028	2.38E+00
gene8760	1E+08	NC_000070.6	Gm12362	2.38E+00
gene30020	1E+08	NC_000078.6	Rps11-ps4	2.38E+00
gene10192	26562	NC_000070.6	Ncdn	2.37E+00
gene15577	68465	NC_000072.6	Adipor2	2.37E+00
gene42334	107373	NC_000085.6	Fam111a	2.37E+00
gene11292	320770	NC_000071.6	A630072M18Rik	2.37E+00
gene18503	107769	NC_000073.6	Tm6sf1	2.37E+00
gene35214	668489	NC_000080.6	Gm9199	2.37E+00
gene12907	1E+08	NC_000071.6	Gm32853	2.37E+00
gene7165	71913	NC_000069.6	Tmem79	2.37E+00
gene30589	20773	NC_000078.6	Sptlc2	2.37E+00
gene7029	229443	NC_000069.6	Gm4857	2.37E+00

gene21000	19704	NC_000074.6	Upf1	2.37E+00
gene36598	73739	NC_000081.6	Cby1	2.37E+00
gene35290	210808	NC_000080.6	Lacc1	2.37E+00
gene29398	12516	NC_000077.6	Cd7	2.37E+00
gene40231	22324	NC_000083.6	Vav1	2.37E+00
gene8941	1E+08	NC_000070.6	Hrct1	2.37E+00
gene20097	1E+08	NC_000074.6	Gm30502	2.37E+00
gene9894	654318	NC_000070.6	C530005A16Rik	2.37E+00
gene10821	170731	NC_000070.6	Mfn2	2.36E+00
gene1670	98710	NC_000067.6	Rabif	2.36E+00
gene23135	110446	NC_000075.6	Acat1	2.36E+00
gene41431	20496	NC_000084.6	Slc12a2	2.36E+00
gene40783	76482	NC_000084.6	3110002H16Rik	2.36E+00
gene32118	66895	NC_000079.6	Pxdc1	2.36E+00
gene11010	230991	NC_000070.6	B930041F14Rik	2.36E+00
gene7771	229694	NC_000069.6	AI504432	2.36E+00
gene10342	399101	NC_000070.6	Snhg3	2.36E+00
gene24161	235611	NC_000075.6	Plxnb1	2.36E+00
gene13750	101118	NC_000072.6	Tmem168	2.36E+00
gene41848	67105	NC_000084.6	Timm21	2.36E+00
gene7220	68563	NC_000069.6	Dpm3	2.36E+00
gene13140	22385	NC_000071.6	Baz1b	2.36E+00
gene23449	235442	NC_000075.6	Rab8b	2.36E+00
gene15446	78783	NC_000072.6	Brpf1	2.35E+00
gene5013	17153	NC_000068.7	Mal	2.35E+00
gene41589	58801	NC_000084.6	Pmaip1	2.35E+00
gene26607	75219	NC_000077.6	Dusp18	2.35E+00
gene24364	74498	NC_000075.6	Gorasp1	2.35E+00
gene19090	55992	NC_000073.6	Trim3	2.35E+00
gene18239	50794	NC_000073.6	Klf13	2.35E+00
gene5118	70997	NC_000068.7	Spef1	2.35E+00
gene27040	16822	NC_000077.6	Lcp2	2.34E+00
gene11020	56036	NC_000070.6	Ccnl2	2.34E+00
gene7155	17261	NC_000069.6	Mef2d	2.34E+00
gene23157	58233	NC_000075.6	Dnaja4	2.34E+00
gene3362	338348	NC_000068.7	Ttc16	2.34E+00
gene22937	71764	NC_000075.6	C2cd2l	2.34E+00
gene37112	1E+08	NC_000081.6	Gm34939	2.34E+00
gene6009	57314	NC_000068.7	Nelfcd	2.34E+00
gene3918	229279	NC_000068.7	Hnrnpa3	2.34E+00
gene21523	109006	NC_000074.6	Ciapin1	2.34E+00
gene37355	70511	NC_000082.6	Eef2kmt	2.34E+00
gene32431	18030	NC_000079.6	Nfil3	2.34E+00
gene29002	75573	NC_000077.6	Prr29	2.34E+00
gene13384	231868	NC_000071.6	E130309D02Rik	2.34E+00
gene8492	12319	NC_000070.6	Car8	2.33E+00
gene9028	21812	NC_000070.6	Tgfbr1	2.33E+00
gene7252	1E+08	NC_000069.6	Gm19710	2.33E+00

gene6897	71004	NC_000069.6	4931440P22Rik	2.33E+00
gene11634	80911	NC_000071.6	Acox3	2.33E+00
gene33039	80289	NC_000079.6	Lysmd3	2.33E+00
gene1053	20684	NC_000067.6	Sp100	2.33E+00
gene42783	1E+08	NC_000085.6	Gm33305	2.32E+00
gene37546	12929	NC_000082.6	Crkl	2.32E+00
gene20508	13685	NC_000074.6	Eif4ebp1	2.32E+00
gene10375	52174	NC_000070.6	Tmem222	2.32E+00
gene28046	280408	NC_000077.6	Rilp	2.32E+00
gene29221	53413	NC_000077.6	Exoc7	2.32E+00
gene27000	13650	NC_000077.6	Rhbdf1	2.32E+00
gene29357	66431	NC_000077.6	Oxld1	2.32E+00
gene28685	21973	NC_000077.6	Top2a	2.32E+00
gene18861	233575	NC_000073.6	Pgap2	2.32E+00
gene32059	14238	NC_000079.6	Foxf2	2.32E+00
gene894	66821	NC_000067.6	Bcs1l	2.32E+00
gene30382	1E+08	NC_000078.6	Gm18899	2.32E+00
gene29358	67291	NC_000077.6	Ccdc137	2.32E+00
gene20011	13043	NC_000073.6	Cttn	2.32E+00
gene20990	234371	NC_000074.6	Tmem161a	2.32E+00
gene42601	77125	NC_000085.6	Il33	2.31E+00
gene35763	67832	NC_000081.6	Brix1	2.31E+00
gene36678	28075	NC_000081.6	Desi1	2.31E+00
gene24464	29806	NC_000075.6	Limd1	2.31E+00
gene7166	229512	NC_000069.6	Smg5	2.31E+00
gene42164	53319	NC_000085.6	Nxf1	2.31E+00
gene33739	667030	NC_000080.6	Slc25a5-ps	2.31E+00
gene40216	66972	NC_000083.6	Slc25a23	2.31E+00
gene24417	270210	NC_000075.6	Zfp651	2.31E+00
gene8581	433688	NC_000070.6	Gm11836	2.31E+00
gene11119	13121	NC_000071.6	Cyp51	2.31E+00
gene34779	1E+08	NC_000080.6	Gm31607	2.31E+00
gene42124	108899	NC_000085.6	2700081O15Rik	2.30E+00
gene12954	208606	NC_000071.6	Rsrc2	2.30E+00
gene17430	243912	NC_000073.6	Hspb6	2.30E+00
gene30553	21809	NC_000078.6	Tgfb3	2.30E+00
gene10840	71707	NC_000070.6	Ubiad1	2.30E+00
gene32959	67433	NC_000079.6	Ccdc127	2.30E+00
gene5048	=Gene;ger	NC_000068.7	LOC108168793	2.30E+00
gene25225	69894	NC_000076.6	2010107G23Rik	2.30E+00
gene21550	546088	NC_000074.6	Gm5912	2.30E+00
gene7947	1.1E+08	NC_000069.6	Gm38684	2.30E+00
gene20547	330731	NC_000074.6	Gm5117	2.30E+00
gene7045	79235	NC_000069.6	Lrat	2.30E+00
gene21337	212999	NC_000074.6	Tnpo2	2.29E+00
gene24414	26901	NC_000075.6	Deb1	2.29E+00
gene35927	67154	NC_000081.6	Mtdh	2.29E+00
gene27867	50932	NC_000077.6	Mink1	2.29E+00

gene29910	432649	NC_000078.6	Gm5434	2.29E+00
gene21526	20021	NC_000074.6	Polr2c	2.29E+00
gene41886	12894	NC_000085.6	Cpt1a	2.29E+00
gene1406	319901	NC_000067.6	Dsel	2.29E+00
gene28838	66599	NC_000077.6	Rdm1	2.29E+00
gene39384	106504	NC_000083.6	Stk38	2.29E+00
gene32525	26914	NC_000079.6	H2afy	2.29E+00
gene17842	20256	NC_000073.6	Clec11a	2.29E+00
gene20671	18983	NC_000074.6	Cnot7	2.29E+00
gene27916	67279	NC_000077.6	Med31	2.29E+00
gene11165	74114	NC_000071.6	Crot	2.29E+00
gene3328	227723	NC_000068.7	Prrc2b	2.28E+00
gene3446	19224	NC_000068.7	Ptgs1	2.28E+00
gene16036	1.1E+08	NC_000072.6	Gm40409	2.28E+00
gene18435	18631	NC_000073.6	Pex11a	2.28E+00
gene21042	270058	NC_000074.6	Map1s	2.28E+00
gene17259	192192	NC_000073.6	Shkbp1	2.28E+00
gene37218	19411	NC_000081.6	Rarg	2.28E+00
gene25212	67895	NC_000076.6	Ppa1	2.28E+00
gene883	56695	NC_000067.6	Pnkd	2.28E+00
gene10822	18822	NC_000070.6	Plod1	2.28E+00
gene10053	230709	NC_000070.6	Zmpste24	2.28E+00
gene10037	51797	NC_000070.6	Ctps	2.28E+00
gene30921	1.1E+08	NC_000078.6	Gm40571	2.28E+00
gene42672	667373	NC_000085.6	Ifit1bl1	2.28E+00
gene7249	54667	NC_000069.6	Atp8b2	2.27E+00
gene15851	408064	NC_000072.6	BC064078	2.27E+00
gene31112	217882	NC_000078.6	Cep170b	2.27E+00
gene8288	66421	NC_000069.6	2410004B18Rik	2.27E+00
gene38537	60364	NC_000082.6	Donson	2.27E+00
gene9948	230673	NC_000070.6	Ipo13	2.27E+00
gene24203	67169	NC_000075.6	Nradd	2.27E+00
gene13395	67238	NC_000071.6	Fam220a	2.27E+00
gene30258	56444	NC_000078.6	Actr10	2.27E+00
gene36452	59053	NC_000081.6	Hgh1	2.27E+00
gene13717	667028	NC_000072.6	Gm8428	2.27E+00
gene22294	77532	NC_000075.6	Jrkl	2.27E+00
gene23080	270156	NC_000075.6	AU019823	2.27E+00
gene8671	384001	NC_000070.6	Prdx6-ps2	2.27E+00
gene15695	317677	NC_000072.6	C1s2	2.27E+00
gene2343	14194	NC_000067.6	Fh1	2.27E+00
gene21863	270096	NC_000074.6	Mon1b	2.27E+00
gene13414	66898	NC_000071.6	Baiap2l1	2.27E+00
gene27526	192191	NC_000077.6	Med9	2.27E+00
gene13385	72881	NC_000071.6	Zdhhc4	2.26E+00
gene5793	140579	NC_000068.7	Elmo2	2.26E+00
gene38387	14390	NC_000082.6	Gabpa	2.26E+00
gene40166	224897	NC_000083.6	Dpp9	2.26E+00

gene42011	71711	NC_000085.6	Mus81	2.26E+00
gene2238	17085	NC_000067.6	Ly9	2.26E+00
gene29321	672511	NC_000077.6	Rnf213	2.26E+00
gene7762	72121	NC_000069.6	Dennd2d	2.26E+00
gene39475	664702	NC_000083.6	A530088E08Rik	2.26E+00
gene27110	66827	NC_000077.6	Ttc1	2.26E+00
gene5277	27054	NC_000068.7	Sec23b	2.26E+00
gene5740	19668	NC_000068.7	Rbpjl	2.26E+00
gene36269	268812	NC_000081.6	Gm5046	2.26E+00
gene18833	11871	NC_000073.6	Art2a-ps	2.26E+00
gene4445	18633	NC_000068.7	Pex16	2.26E+00
gene6494	56456	NC_000069.6	Actl6a	2.26E+00
gene28382	170749	NC_000077.6	Mtmr4	2.26E+00
gene10368	242687	NC_000070.6	Wasf2	2.26E+00
gene31042	72805	NC_000078.6	Zfp839	2.25E+00
gene13170	54153	NC_000071.6	Rasa4	2.25E+00
gene20292	670326	NC_000074.6	Gm16425	2.25E+00
gene11847	12397	NC_000071.6	Cbfa2t2-ps1	2.25E+00
gene15682	623374	NC_000072.6	Gm6423	2.25E+00
gene17896	65116	NC_000073.6	Prrg2	2.25E+00
gene17771	233186	NC_000073.6	Siglecf	2.25E+00
gene42224	66072	NC_000085.6	Sdhaf2	2.24E+00
gene28123	57837	NC_000077.6	Eral1	2.24E+00
gene3299	227707	NC_000068.7	BC005624	2.24E+00
gene29018	1E+08	NC_000077.6	Gm11708	2.24E+00
gene36194	210998	NC_000081.6	Fam91a1	2.24E+00
gene39375	26415	NC_000083.6	Mapk13	2.24E+00
gene35163	20410	NC_000080.6	Sorbs3	2.24E+00
gene23267	20320	NC_000075.6	Nptn	2.24E+00
gene31589	16323	NC_000079.6	Inhba	2.24E+00
gene38166	76302	NC_000082.6	Pcnp	2.24E+00
gene5382	110078	NC_000068.7	Pygb	2.23E+00
gene20423	102247	NC_000074.6	Gpat4	2.23E+00
gene20539	234138	NC_000074.6	Tti2	2.23E+00
gene683	12168	NC_000067.6	Bmpr2	2.23E+00
gene36544	72709	NC_000081.6	C1qtnf6	2.23E+00
gene6493	14696	NC_000069.6	Gnb4	2.23E+00
gene14338	58887	NC_000072.6	Repin1	2.23E+00
gene30580	14874	NC_000078.6	Gstz1	2.23E+00
gene22604	19143	NC_000075.6	St14	2.23E+00
gene25857	20962	NC_000076.6	Sycp3	2.23E+00
gene28570	12412	NC_000077.6	Cbx1	2.23E+00
gene24636	380629	NC_000076.6	Heca	2.23E+00
gene30882	18789	NC_000078.6	Papola	2.23E+00
gene5953	67337	NC_000068.7	Cstf1	2.23E+00
gene39246	106707	NC_000083.6	Rpusd1	2.23E+00
gene18127	93790	NC_000073.6	Nipa2	2.23E+00
gene18832	=Gene;ger	NC_000073.6	LOC102638993	2.23E+00

gene36184	75758	NC_000081.6	9130401M01Rik	2.23E+00
gene40785	18145	NC_000084.6	Npc1	2.23E+00
gene19612	ene=LOC1	NC_000073.6	LOC108167515	2.23E+00
gene31449	1E+08	NC_000079.6	Gm20277	2.23E+00
gene27203	22720	NC_000077.6	Zfp62	2.22E+00
gene32725	218298	NC_000079.6	Gm4810	2.22E+00
gene20	18777	NC_000067.6	Lypla1	2.22E+00
gene21288	244550	NC_000074.6	Podnl1	2.22E+00
gene19504	233870	NC_000073.6	Tufm	2.22E+00
gene4795	640461	NC_000068.7	Gm14016	2.22E+00
gene19821	69752	NC_000073.6	Zfp511	2.22E+00
gene20447	83436	NC_000074.6	Plekha2	2.22E+00
gene14463	ene=LOC1	NC_000072.6	LOC108169143	2.22E+00
gene27444	79566	NC_000077.6	Sh3bp5l	2.22E+00
gene41172	13367	NC_000084.6	Diaph1	2.22E+00
gene35756	16197	NC_000081.6	Il7r	2.22E+00
gene8840	69638	NC_000070.6	Enho	2.22E+00
gene11011	68991	NC_000070.6	Ssu72	2.22E+00
gene7842	329739	NC_000069.6	Fam102b	2.22E+00
gene18125	20430	NC_000073.6	Cyfip1	2.22E+00
gene2415	11545	NC_000067.6	Parp1	2.22E+00
gene26939	104570	NC_000077.6	Smek2	2.22E+00
gene18733	434215	NC_000073.6	Lrrc32	2.22E+00
gene27573	216821	NC_000077.6	Tmem11	2.22E+00
gene10427	16765	NC_000070.6	Stmn1	2.22E+00
gene5437	67917	NC_000068.7	Zcchc3	2.22E+00
gene5122	228607	NC_000068.7	Mavs	2.21E+00
gene9773	66073	NC_000070.6	Txndc12	2.21E+00
gene1693	77552	NC_000067.6	Shisa4	2.21E+00
gene5376	71881	NC_000068.7	Apmap	2.21E+00
gene25593	27403	NC_000076.6	Abca7	2.21E+00
gene6131	56436	NC_000068.7	Adrm1	2.21E+00
gene39373	26416	NC_000083.6	Mapk14	2.21E+00
gene31569	14593	NC_000079.6	Ggps1	2.21E+00
gene37859	666966	NC_000082.6	Gm8387	2.21E+00
gene41978	70445	NC_000085.6	Cd248	2.21E+00
gene40899	69256	NC_000084.6	Zfp397	2.20E+00
gene24698	20393	NC_000076.6	Sgk1	2.20E+00
gene5711	76080	NC_000068.7	Ttpal	2.20E+00
gene34959	328424	NC_000080.6	Kcnrg	2.20E+00
gene21408	1E+08	NC_000074.6	Gm34751	2.20E+00
gene21402	382030	NC_000074.6	Cnep1r1	2.20E+00
gene4771	114715	NC_000068.7	Spred1	2.20E+00
gene825	383528	NC_000067.6	Gm5256	2.20E+00
gene40059	106821	NC_000083.6	Oard1	2.20E+00
gene29270	217353	NC_000077.6	Tmc6	2.20E+00
gene39900	277225	NC_000083.6	Ldha-ps3	2.20E+00
gene25239	72320	NC_000076.6	Kif1bp	2.20E+00

gene42022	104401	NC_000085.6	Pcnx3	2.19E+00
gene28013	74148	NC_000077.6	Cluh	2.19E+00
gene15070	73327	NC_000072.6	Pradc1	2.19E+00
gene2722	227522	NC_000068.7	Rpp38	2.19E+00
gene36865	=Gene;ger	NC_000081.6	LOC108168256	2.19E+00
gene27930	1.1E+08	NC_000077.6	Gm40194	2.19E+00
gene20241	74901	NC_000074.6	Kbtbd11	2.19E+00
gene42727	226101	NC_000085.6	Myof	2.19E+00
gene24124	22258	NC_000075.6	Usp4	2.19E+00
gene6835	56758	NC_000069.6	Mbnl1	2.19E+00
gene25988	13179	NC_000076.6	Dcn	2.19E+00
gene3532	67857	NC_000068.7	Ppp6c	2.19E+00
gene1500	666971	NC_000067.6	Gm8392	2.19E+00
gene5668	18803	NC_000068.7	Plcg1	2.19E+00
gene11497	12615	NC_000071.6	Cenpa	2.19E+00
gene23201	235406	NC_000075.6	Snx33	2.18E+00
gene3150	64436	NC_000068.7	Inpp5e	2.18E+00
gene4415	80708	NC_000068.7	Pacsin3	2.18E+00
gene2224	18637	NC_000067.6	Pfdn2	2.18E+00
gene15712	14793	NC_000072.6	Cdca3	2.18E+00
gene5116	72630	NC_000068.7	Hspa12b	2.17E+00
gene16320	333182	NC_000073.6	Cox6b2	2.17E+00
gene36856	170790	NC_000081.6	Mlc1	2.17E+00
gene37701	20462	NC_000082.6	Tra2b	2.17E+00
gene11383	19744	NC_000071.6	Rheb	2.17E+00
gene9449	230379	NC_000070.6	Acer2	2.17E+00
gene22146	668444	NC_000074.6	Gm9172	2.17E+00
gene32516	12328	NC_000079.6	Caml	2.17E+00
gene3817	13427	NC_000068.7	Dync1i2	2.17E+00
gene7900	15247	NC_000069.6	Mfsd14a	2.17E+00
gene389	98258	NC_000067.6	Txndc9	2.17E+00
gene28406	64656	NC_000077.6	Mrps23	2.17E+00
gene22067	114896	NC_000074.6	Afg3l1	2.17E+00
gene21545	14894	NC_000074.6	Cfap20	2.17E+00
gene36984	72193	NC_000081.6	Scaf11	2.17E+00
gene24672	72567	NC_000076.6	Bclaf1	2.16E+00
gene29383	217366	NC_000077.6	Lrrc45	2.16E+00
gene34783	628176	NC_000080.6	Gm6852	2.16E+00
gene9032	66212	NC_000070.6	Sec61b	2.16E+00
gene28615	26446	NC_000077.6	Psmb3	2.16E+00
gene19445	59288	NC_000073.6	Dctn5	2.16E+00
gene19233	57373	NC_000073.6	Akip1	2.16E+00
gene31120	56696	NC_000078.6	Gpr132	2.16E+00
gene15651	67602	NC_000072.6	Necap1	2.16E+00
gene1590	98415	NC_000067.6	Nucks1	2.16E+00
gene24187	17758	NC_000075.6	Map4	2.16E+00
gene12215	1E+08	NC_000071.6	Gm15710	2.16E+00
gene36602	14904	NC_000081.6	Gtpbp1	2.16E+00

gene29093	16518	NC_000077.6	Kcnj2	2.16E+00
gene3796	75422	NC_000068.7	Mettl5	2.16E+00
gene1820	1E+08	NC_000067.6	Ptgs2os2	2.16E+00
gene18128	320845	NC_000073.6	A230056P14Rik	2.16E+00
gene8074	12368	NC_000069.6	Casp6	2.16E+00
gene3976	70599	NC_000068.7	Ssfa2	2.15E+00
gene34495	56335	NC_000080.6	Mettl3	2.15E+00
gene39423	109801	NC_000083.6	Glo1	2.15E+00
gene10448	51796	NC_000070.6	Srrm1	2.15E+00
gene12369	26891	NC_000071.6	Cops4	2.15E+00
gene25753	50493	NC_000076.6	Txnrd1	2.15E+00
gene21018	66522	NC_000074.6	Pgpep1	2.15E+00
gene12210	23881	NC_000071.6	G3bp2	2.15E+00
gene385	69028	NC_000067.6	Mitd1	2.15E+00
gene21657	=Gene;ger	NC_000074.6	LOC102636360	2.15E+00
gene7970	14630	NC_000069.6	Gclm	2.15E+00
gene26853	216558	NC_000077.6	Ugp2	2.15E+00
gene30138	78232	NC_000078.6	Trappc6b	2.15E+00
gene4886	99334	NC_000068.7	Zscan29	2.15E+00
gene13410	70381	NC_000071.6	Tecpr1	2.15E+00
gene12686	23790	NC_000071.6	Coro1c	2.15E+00
gene26167	19263	NC_000076.6	Ptprb	2.14E+00
gene16507	545911	NC_000073.6	Gm5888	2.14E+00
gene37058	223881	NC_000081.6	Rnd1	2.14E+00
gene37894	77446	NC_000082.6	Heg1	2.14E+00
gene33790	21974	NC_000080.6	Top2b	2.14E+00
gene19092	14356	NC_000073.6	Timm10b	2.14E+00
gene37538	22195	NC_000082.6	Ube2l3	2.14E+00
gene32736	66631	NC_000079.6	Mfsd14b	2.14E+00
gene15654	73149	NC_000072.6	Clec4a3	2.14E+00
gene5169	66926	NC_000068.7	Trmt6	2.14E+00
gene27744	20877	NC_000077.6	Aurkb	2.14E+00
gene26247	71902	NC_000076.6	Cand1	2.14E+00
gene18499	67148	NC_000073.6	Fam103a1	2.14E+00
gene28993	72047	NC_000077.6	Ddx42	2.14E+00
gene37308	74763	NC_000082.6	Naa60	2.14E+00
gene31060	74190	NC_000078.6	Exoc3l4	2.13E+00
gene28656	14786	NC_000077.6	Grb7	2.13E+00
gene30148	665644	NC_000078.6	Gm7727	2.13E+00
gene8942	71406	NC_000070.6	5430416O09Rik	2.13E+00
gene9767	230596	NC_000070.6	Prpf38a	2.13E+00
gene32408	667728	NC_000079.6	Hist1h2al	2.13E+00
gene4007	69082	NC_000068.7	Zc3h15	2.13E+00
gene12365	69162	NC_000071.6	Sec31a	2.13E+00
gene144	70397	NC_000067.6	Tmem70	2.13E+00
gene10762	627585	NC_000070.6	Gm13034	2.13E+00
gene37899	22247	NC_000082.6	Umps	2.13E+00
gene26423	17938	NC_000076.6	Naca	2.13E+00

gene18661	66271	NC_000073.6	Tmem126a	2.13E+00
gene22188	67952	NC_000074.6	Tomm20	2.13E+00
gene7568	74414	NC_000069.6	Polr3c	2.13E+00
gene33939	68173	NC_000080.6	Ppifos	2.13E+00
gene22901	69632	NC_000075.6	Arhgef12	2.13E+00
gene30521	1E+08	NC_000078.6	D030025P21Rik	2.13E+00
gene5334	20608	NC_000068.7	Sstr4	2.13E+00
gene37780	67501	NC_000082.6	Ccdc50	2.13E+00
gene14882	22644	NC_000072.6	Rnf103	2.13E+00
gene12594	71782	NC_000071.6	Ankle2	2.12E+00
gene7142	229504	NC_000069.6	Isg20l2	2.12E+00
gene36862	223776	NC_000081.6	Selo	2.12E+00
gene39471	56399	NC_000083.6	Akap8	2.12E+00
gene12605	69663	NC_000071.6	Ddx51	2.12E+00
gene22653	71989	NC_000075.6	Rpusd4	2.12E+00
gene5120	12531	NC_000068.7	Cdc25b	2.12E+00
gene23559	66364	NC_000075.6	2310009A05Rik	2.12E+00
gene7560	229613	NC_000069.6	6330549D23Rik	2.12E+00
gene34339	218975	NC_000080.6	Mapk1ip1l	2.12E+00
gene42259	12482	NC_000085.6	Ms4a1	2.12E+00
gene7425	70737	NC_000069.6	Cgn	2.11E+00
gene1235	14733	NC_000067.6	Gpc1	2.11E+00
gene16903	232947	NC_000073.6	Ppp1r37	2.11E+00
gene27951	76281	NC_000077.6	Tax1bp3	2.11E+00
gene7763	99712	NC_000069.6	Cept1	2.11E+00
gene31450	142688	NC_000079.6	Asb13	2.11E+00
gene36464	52710	NC_000081.6	Slc52a2	2.11E+00
gene13062	14467	NC_000071.6	Gbas	2.11E+00
gene1066	70247	NC_000067.6	Psmd1	2.11E+00
gene34040	192187	NC_000080.6	Stab1	2.11E+00
gene29883	28071	NC_000078.6	Twistnb	2.11E+00
gene21257	106529	NC_000074.6	Tecr	2.11E+00
gene28798	=Gene;ger	NC_000077.6	LOC108167926	2.11E+00
gene1116	1E+08	NC_000067.6	Gm33470	2.11E+00
gene10319	545681	NC_000070.6	Gm12992	2.11E+00
gene28867	15184	NC_000077.6	Hdac5	2.11E+00
gene16797	101540	NC_000073.6	Prkd2	2.11E+00
gene5576	266692	NC_000068.7	Cpne1	2.10E+00
gene35629	78312	NC_000080.6	1810041H14Rik	2.10E+00
gene4124	1E+08	NC_000068.7	Gm13719	2.10E+00
gene34748	56215	NC_000080.6	Acin1	2.10E+00
gene16339	68490	NC_000073.6	Zfp579	2.10E+00
gene41030	66306	NC_000084.6	Fam53c	2.10E+00
gene3406	66998	NC_000068.7	Psmd5	2.10E+00
gene21654	78688	NC_000074.6	Nol3	2.10E+00
gene37993	224143	NC_000082.6	Poglut1	2.10E+00
gene23004	18475	NC_000075.6	Pafah1b2	2.10E+00
gene27421	50724	NC_000077.6	Sap30l	2.10E+00

gene41963	56464	NC_000085.6	Ctsf	2.10E+00
gene17436	75410	NC_000073.6	Kmt2b	2.10E+00
gene7797	14866	NC_000069.6	Gstm5	2.10E+00
gene1259	66385	NC_000067.6	Ppp1r7	2.09E+00
gene9934	66233	NC_000070.6	Dmap1	2.09E+00
gene32890	28077	NC_000079.6	Med10	2.09E+00
gene9277	100182	NC_000070.6	Akna	2.09E+00
gene26362	216438	NC_000076.6	March9	2.09E+00
gene13174	72041	NC_000071.6	Alkbh4	2.09E+00
gene30583	1.1E+08	NC_000078.6	Gm40496	2.09E+00
gene28271	103737	NC_000077.6	Pex12	2.09E+00
gene33520	218693	NC_000079.6	Paip1	2.09E+00
gene13331	59031	NC_000071.6	Chst12	2.09E+00
gene41510	106877	NC_000084.6	Afap1l1	2.09E+00
gene9273	18405	NC_000070.6	Orm1	2.09E+00
gene20236	26889	NC_000074.6	Cln8	2.09E+00
gene36120	68135	NC_000081.6	Eif3h	2.09E+00
gene18106	75744	NC_000073.6	Svip	2.09E+00
gene10843	50912	NC_000070.6	Exosc10	2.09E+00
gene17933	78405	NC_000073.6	Ntf5	2.09E+00
gene25610	14431	NC_000076.6	Gamt	2.09E+00
gene28060	74230	NC_000077.6	1700016K19Rik	2.08E+00
gene19394	233806	NC_000073.6	Tmem159	2.08E+00
gene3173	20935	NC_000068.7	Surf6	2.08E+00
gene2155	107652	NC_000067.6	Uap1	2.08E+00
gene21222	1E+08	NC_000074.6	Gm31105	2.08E+00
gene24738	20042	NC_000076.6	Rps12	2.08E+00
gene79	73824	NC_000067.6	Snhg6	2.08E+00
gene11603	433874	NC_000071.6	Gm5553	2.08E+00
gene27001	268395	NC_000077.6	Mpg	2.08E+00
gene25631	118445	NC_000076.6	Klf16	2.08E+00
gene40155	20405	NC_000083.6	Sh3gl1	2.08E+00
gene27725	83431	NC_000077.6	Ndel1	2.08E+00
gene34164	218945	NC_000080.6	Gm4817	2.08E+00
gene41025	52563	NC_000084.6	Cdc23	2.08E+00
gene26360	17299	NC_000076.6	Mettl1	2.08E+00
gene24895	15182	NC_000076.6	Hdac2	2.08E+00
gene6246	633072	NC_000069.6	Gm7103	2.08E+00
gene17946	12036	NC_000073.6	Bcat2	2.08E+00
gene39737	625785	NC_000083.6	Gm6623	2.07E+00
gene19795	101631	NC_000073.6	Pwwp2b	2.07E+00
gene31489	56421	NC_000079.6	Pfkp	2.07E+00
gene25418	20587	NC_000076.6	Smarcb1	2.07E+00
gene42989	414801	NC_000085.6	Itprip	2.07E+00
gene14030	66117	NC_000072.6	1110001J03Rik	2.07E+00
gene39049	1E+08	NC_000083.6	Gm10509	2.07E+00
gene37055	11512	NC_000081.6	Adcy6	2.07E+00
gene819	108147	NC_000067.6	Atic	2.07E+00

gene36160	68537	NC_000081.6	Mrpl13	2.07E+00
gene29615	73667	NC_000078.6	2410004P03Rik	2.07E+00
gene38352	13052	NC_000082.6	Cxadr	2.07E+00
gene2425	208768	NC_000067.6	Sde2	2.07E+00
gene20641	621080	NC_000074.6	AI429214	2.07E+00
gene28989	26406	NC_000077.6	Map3k3	2.07E+00
gene34768	791403	NC_000080.6	Mhrt	2.06E+00
gene12187	20310	NC_000071.6	Cxcl2	2.06E+00
gene28487	94064	NC_000077.6	Mrpl27	2.06E+00
gene16108	16832	NC_000072.6	Ldhb	2.06E+00
gene5090	80743	NC_000068.7	Vps16	2.06E+00
gene36663	20286	NC_000081.6	Zc3h7b	2.06E+00
gene23082	235339	NC_000075.6	Dlat	2.06E+00
gene20385	207958	NC_000074.6	Alg11	2.06E+00
gene32339	76895	NC_000079.6	Bicd2	2.06E+00
gene15310	22200	NC_000072.6	Uba3	2.06E+00
gene11584	20492	NC_000071.6	Slbp	2.06E+00
gene2729	50497	NC_000068.7	Hspa14	2.06E+00
gene1013	15463	NC_000067.6	Agfg1	2.06E+00
gene29848	23985	NC_000078.6	Slc26a4	2.06E+00
gene11677	93895	NC_000071.6	Msx1os	2.06E+00
gene25913	13713	NC_000076.6	Elk3	2.06E+00
gene40779	1.1E+08	NC_000084.6	Gm41666	2.06E+00
gene25374	1E+08	NC_000076.6	Gm9923	2.06E+00
gene27620	22368	NC_000077.6	Trpv2	2.06E+00
gene31630	76205	NC_000079.6	Stard3nl	2.06E+00
gene39396	80748	NC_000083.6	BC004004	2.05E+00
gene24143	57279	NC_000075.6	Slc25a20	2.05E+00
gene27522	26572	NC_000077.6	Cops3	2.05E+00
gene18865	20133	NC_000073.6	Rrm1	2.05E+00
gene8590	100201	NC_000070.6	Tmem64	2.05E+00
gene19	27395	NC_000067.6	Mrpl15	2.05E+00
gene9202	66209	NC_000070.6	Inip	2.05E+00
gene14891	76614	NC_000072.6	Immt	2.05E+00
gene28828	19942	NC_000077.6	Rpl27	2.05E+00
gene28117	1E+08	NC_000077.6	Dhrs13os	2.05E+00
gene36073	16341	NC_000081.6	Eif3e	2.05E+00
gene11818	66278	NC_000071.6	Smim20	2.05E+00
gene41084	108112	NC_000084.6	Eif4ebp3	2.05E+00
gene32527	1E+08	NC_000079.6	Gm31657	2.05E+00
gene20048	94178	NC_000074.6	Mcoln1	2.05E+00
gene22247	76863	NC_000075.6	Dcun1d5	2.05E+00
gene12157	13178	NC_000071.6	Dck	2.05E+00
gene2061	74895	NC_000067.6	Ccdc181	2.05E+00
gene32057	15220	NC_000079.6	Foxq1	2.05E+00
gene23086	66952	NC_000075.6	2310030G06Rik	2.04E+00
gene37163	380977	NC_000081.6	A330009N23Rik	2.04E+00
gene26652	14030	NC_000077.6	Ewsr1	2.04E+00

gene17972	101612	NC_000073.6	Grwd1	2.04E+00
gene11026	71957	NC_000070.6	Cpsf3l	2.04E+00
gene39981	677168	NC_000083.6	Gm9706	2.04E+00
gene15745	21940	NC_000072.6	Cd27	2.04E+00
gene31415	1E+08	NC_000078.6	Gm18441	2.04E+00
gene20577	22428	NC_000074.6	Dctn6	2.04E+00
gene28616	108083	NC_000077.6	Pip4k2b	2.04E+00
gene14517	210973	NC_000072.6	Kbtbd2	2.04E+00
gene21525	67914	NC_000074.6	Coq9	2.04E+00
gene35698	19219	NC_000081.6	Ptger4	2.04E+00
gene10635	100165	NC_000070.6	AI507597	2.04E+00
gene27947	14841	NC_000077.6	Gsg2	2.04E+00
gene4490	70852	NC_000068.7	Alkbh3os1	2.04E+00
gene9107	ene;gene=l	NC_000070.6	LOC108168953	2.04E+00
gene27429	69125	NC_000077.6	Cnot8	2.03E+00
gene19508	12752	NC_000073.6	Cln3	2.03E+00
gene11399	73571	NC_000071.6	1700096K18Rik	2.03E+00
gene42032	66961	NC_000085.6	Neat1	2.03E+00
gene25419	17385	NC_000076.6	Mmp11	2.03E+00
gene23562	225215	NC_000075.6	Rsl24d1	2.03E+00
gene12744	384281	NC_000071.6	Gatc	2.03E+00
gene20801	11593	NC_000074.6	Aga	2.03E+00
gene7258	59069	NC_000069.6	Tpm3	2.03E+00
gene22504	LOC10004	NC_000075.6	LOC100049077	2.03E+00
gene13964	58246	NC_000072.6	Slc35b4	2.03E+00
gene3345	98952	NC_000068.7	Fam102a	2.03E+00
gene21133	20467	NC_000074.6	Sin3b	2.03E+00
gene13285	24135	NC_000071.6	Zfp68	2.03E+00
gene41193	26384	NC_000084.6	Gnpda1	2.03E+00
gene1199	ene=LOC1	NC_000067.6	LOC102639127	2.03E+00
gene17604	101831	NC_000073.6	Faap24	2.02E+00
gene34812	16995	NC_000080.6	Ltb4r1	2.02E+00
gene3987	50884	NC_000068.7	Nckap1	2.02E+00
gene21406	11513	NC_000074.6	Adcy7	2.02E+00
gene24105	68176	NC_000075.6	Fam212a	2.02E+00
gene301	433287	NC_000067.6	Gm15455	2.02E+00
gene21310	212528	NC_000074.6	Trmt1	2.02E+00
gene37371	54128	NC_000082.6	Pmm2	2.02E+00
gene33286	69048	NC_000079.6	Slc30a5	2.02E+00
gene36287	20491	NC_000081.6	Sla	2.02E+00
gene3937	83435	NC_000068.7	Plekha3	2.02E+00
gene19602	67773	NC_000073.6	Kat8	2.02E+00
gene18473	56529	NC_000073.6	Sec11a	2.02E+00
gene35261	1E+08	NC_000080.6	Gm4285	2.02E+00
gene9867	230648	NC_000070.6	Efcab14	2.02E+00
gene10996	67513	NC_000070.6	Faap20	2.02E+00
gene22952	1E+08	NC_000075.6	Gm9830	2.02E+00
gene28042	1E+08	NC_000077.6	Mir22hg	2.02E+00

gene34113	319508	NC_000080.6	Syt15	2.02E+00
gene9966	230678	NC_000070.6	Tmem125	2.02E+00
gene22945	14385	NC_000075.6	Slc37a4	2.02E+00
gene16115	12764	NC_000072.6	Cmas	2.01E+00
gene40440	225020	NC_000083.6	Fez2	2.01E+00
gene34490	114741	NC_000080.6	Supt16	2.01E+00
gene19336	53322	NC_000073.6	Nucb2	2.01E+00
gene21201	73945	NC_000074.6	Otud4	2.01E+00
gene3106	22030	NC_000068.7	Traf2	2.01E+00
gene34301	218952	NC_000080.6	Fermt2	2.01E+00
gene26887	103573	NC_000077.6	Xpo1	2.01E+00
gene11310	73078	NC_000071.6	Pmpcb	2.01E+00
gene34483	69890	NC_000080.6	Zfp219	2.01E+00
gene16342	319748	NC_000073.6	Zfp865	2.01E+00
gene30416	109711	NC_000078.6	Actn1	2.01E+00
gene23751	23959	NC_000075.6	Nt5e	2.01E+00
gene1004	=Gene;ger	NC_000067.6	LOC105243964	2.01E+00
gene24070	102626	NC_000075.6	Mapkapk3	2.01E+00
gene15865	243653	NC_000072.6	Clec1a	2.00E+00
gene23159	66317	NC_000075.6	Wdr61	2.00E+00
gene30773	51786	NC_000078.6	Cpsf2	2.00E+00
gene5578	18041	NC_000068.7	Nfs1	2.00E+00
gene6597	99696	NC_000069.6	Ankrd50	2.00E+00
gene21416	74256	NC_000074.6	Cyld	2.00E+00
gene17792	233189	NC_000073.6	Ctu1	2.00E+00
gene36476	69572	NC_000081.6	Mfsd3	2.00E+00
gene17936	14936	NC_000073.6	Gys1	2.00E+00
gene37147	207818	NC_000081.6	Smagp	2.00E+00
gene38827	224530	NC_000083.6	Acat3	2.00E+00
gene41298	225471	NC_000084.6	Ticam2	2.00E+00
gene13411	67277	NC_000071.6	2900089D17Rik	2.00E+00
gene1123	1E+08	NC_000067.6	Gm19582	2.00E+00
gene11612	=Gene;ger	NC_000071.6	LOC108169039	2.00E+00
gene38063	67841	NC_000082.6	Atg3	2.00E+00
gene22065	23854	NC_000074.6	Def8	2.00E+00
gene13044	13852	NC_000071.6	Stx2	2.00E+00
gene37869	22042	NC_000082.6	Tfrc	2.00E+00
gene25149	110829	NC_000076.6	Lims1	1.99E+00
gene32604	69046	NC_000079.6	Isca1	1.99E+00
gene29211	338364	NC_000077.6	Trim65	1.99E+00
gene15718	12504	NC_000072.6	Cd4	1.99E+00
gene7757	12655	NC_000069.6	Chil3	1.99E+00
gene40276	224938	NC_000083.6	Pja2	1.99E+00
gene2907	50755	NC_000068.7	Fbxo18	1.99E+00
gene34881	50523	NC_000080.6	Lats2	1.99E+00
gene14172	12366	NC_000072.6	Casp2	1.99E+00
gene40073	56738	NC_000083.6	Mocs1	1.99E+00
gene4815	269336	NC_000068.7	Ccdc32	1.99E+00

gene42980	20874	NC_000085.6	Slk	1.99E+00
gene22238	12363	NC_000075.6	Casp4	1.99E+00
gene2901	76938	NC_000068.7	Rbm17	1.99E+00
gene40426	72722	NC_000083.6	Fam98a	1.99E+00
gene28109	791303	NC_000077.6	Gm10277	1.98E+00
gene13138	27368	NC_000071.6	Tbl2	1.98E+00
gene8403	75540	NC_000069.6	Fpgt	1.98E+00
gene18268	68634	NC_000073.6	Tm2d3	1.98E+00
gene41174	15183	NC_000084.6	Hdac3	1.98E+00
gene37333	15369	NC_000082.6	Hmox2	1.98E+00
gene38069	385905	NC_000082.6	Gm5406	1.98E+00
gene1915	58244	NC_000067.6	Stx6	1.98E+00
gene7449	213054	NC_000069.6	Gabpb2	1.98E+00
gene5982	65112	NC_000068.7	Pmepa1	1.98E+00
gene22021	234839	NC_000074.6	Piezo1	1.98E+00
gene18848	101706	NC_000073.6	Numa1	1.98E+00
gene34904	236451	NC_000080.6	Phf11b	1.98E+00
gene10251	100383	NC_000070.6	Bsdc1	1.98E+00
gene21067	382014	NC_000074.6	Ano8	1.98E+00
gene21649	234678	NC_000074.6	D230025D16Rik	1.98E+00
gene33328	59079	NC_000079.6	Erbb2ip	1.98E+00
gene17362	52150	NC_000073.6	Kcnk6	1.98E+00
gene31578	105351	NC_000079.6	AW209491	1.98E+00
gene25931	56307	NC_000076.6	Metap2	1.98E+00
gene20087	21856	NC_000074.6	Timm44	1.98E+00
gene14905	28035	NC_000072.6	Usp39	1.98E+00
gene32574	74386	NC_000079.6	Rmi1	1.98E+00
gene41487	13521	NC_000084.6	Slc26a2	1.98E+00
gene12716	66236	NC_000071.6	1500011B03Rik	1.98E+00
gene17193	26365	NC_000073.6	Ceacam1	1.98E+00
gene24826	1E+08	NC_000076.6	Gm30698	1.98E+00
gene13155	215210	NC_000071.6	Tmem120a	1.97E+00
gene2659	16780	NC_000067.6	Lamb3	1.97E+00
gene2591	52477	NC_000067.6	Angel2	1.97E+00
gene36748	223722	NC_000081.6	Mcat	1.97E+00
gene24476	1E+08	NC_000075.6	Gm2774	1.97E+00
gene1269	59041	NC_000067.6	Stk25	1.97E+00
gene34374	70646	NC_000080.6	Naa30	1.97E+00
gene29421	210029	NC_000077.6	Metrnl	1.97E+00
gene13341	1E+08	NC_000071.6	Gm30745	1.97E+00
gene20754	12367	NC_000074.6	Casp3	1.97E+00
gene2254	98193	NC_000067.6	Dcaf8	1.97E+00
gene17313	101497	NC_000073.6	Plekhg2	1.97E+00
gene8353	67035	NC_000069.6	Dnajb4	1.97E+00
gene32067	78903	NC_000079.6	Wrnip1	1.97E+00
gene40239	674419	NC_000083.6	Rpl7a-ps5	1.97E+00
gene37338	17237	NC_000082.6	Mgrn1	1.97E+00
gene16882	403187	NC_000073.6	Opa3	1.97E+00

gene10357	100336	NC_000070.6	Ppp1r8	1.97E+00
gene35895	223453	NC_000081.6	Dap	1.97E+00
gene7957	19299	NC_000069.6	Abcd3	1.97E+00
gene13971	67861	NC_000072.6	Akr1b10	1.96E+00
gene23130	68304	NC_000075.6	Kdelc2	1.96E+00
gene33460	14945	NC_000079.6	Gzmk	1.96E+00
gene9547	18786	NC_000070.6	Plaa	1.96E+00
gene10920	69876	NC_000070.6	Thap3	1.96E+00
gene5845	20613	NC_000068.7	Snai1	1.96E+00
gene42035	107375	NC_000085.6	Slc25a45	1.96E+00
gene37474	1E+08	NC_000082.6	Gm15806	1.96E+00
gene4931	81913	NC_000068.7	Bambi-ps1	1.96E+00
gene36294	20442	NC_000081.6	St3gal1	1.96E+00
gene6511	67713	NC_000069.6	Dnajc19	1.96E+00
gene34769	140781	NC_000080.6	Myh7	1.96E+00
gene25856	432486	NC_000076.6	Gnptab	1.96E+00
gene21026	19339	NC_000074.6	Rab3a	1.96E+00
gene10389	100210	NC_000070.6	Gpn2	1.96E+00
gene21087	72297	NC_000074.6	B3gnt3	1.96E+00
gene660	70827	NC_000067.6	Trak2	1.96E+00
gene10420	230809	NC_000070.6	Pdik1l	1.96E+00
gene38174	21787	NC_000082.6	Tfg	1.96E+00
gene25175	56709	NC_000076.6	Dnajb12	1.95E+00
gene15820	72440	NC_000072.6	Rhno1	1.95E+00
gene12591	1E+08	NC_000071.6	Gm15787	1.95E+00
gene26708	21379	NC_000077.6	Tbrg4	1.95E+00
gene11052	231003	NC_000070.6	Klhl17	1.95E+00
gene41297	240263	NC_000084.6	Fem1c	1.95E+00
gene2027	53330	NC_000067.6	Vamp4	1.95E+00
gene24436	67469	NC_000075.6	Abhd5	1.95E+00
gene43030	1E+08	NC_000085.6	Bbip1	1.95E+00
gene2449	666519	NC_000067.6	Gm8146	1.95E+00
gene28488	217119	NC_000077.6	Xylt2	1.95E+00
gene29054	1E+08	NC_000077.6	Gm36876	1.95E+00
gene5724	58231	NC_000068.7	Stk4	1.95E+00
gene9898	1E+08	NC_000070.6	AV051173	1.95E+00
gene28562	103889	NC_000077.6	Hoxb2	1.95E+00
gene12919	207565	NC_000071.6	Camkk2	1.95E+00
gene18327	11819	NC_000073.6	Nr2f2	1.95E+00
gene13312	68510	NC_000071.6	Ints1	1.95E+00
gene5274	70408	NC_000068.7	Polr3f	1.95E+00
gene37420	106205	NC_000082.6	Zc3h7a	1.95E+00
gene35325	22380	NC_000080.6	Wbp4	1.94E+00
gene41462	67665	NC_000084.6	Dctn4	1.94E+00
gene22462	54721	NC_000075.6	Tyk2	1.94E+00
gene38534	14450	NC_000082.6	Gart	1.94E+00
gene3932	241490	NC_000068.7	Rbm45	1.94E+00
gene21033	16161	NC_000074.6	Il12rb1	1.94E+00

gene36357	57277	NC_000081.6	Slurp1	1.94E+00
gene28900	20624	NC_000077.6	Eftud2	1.94E+00
gene348	226976	NC_000067.6	Kansl3	1.94E+00
gene33938	105675	NC_000080.6	Ppif	1.94E+00
gene30653	667475	NC_000078.6	Gm8655	1.94E+00
gene34979	545062	NC_000080.6	Gm5802	1.94E+00
gene24219	235633	NC_000075.6	Als2cl	1.94E+00
gene12537	231580	NC_000071.6	Gak	1.94E+00
gene579	227102	NC_000067.6	Ormdl1	1.94E+00
gene8118	71793	NC_000069.6	Ints12	1.94E+00
gene6359	74252	NC_000069.6	Armc1	1.94E+00
gene26018	17311	NC_000076.6	Kitl	1.93E+00
gene28915	67131	NC_000077.6	Acbd4	1.93E+00
gene21606	57813	NC_000074.6	Tk2	1.93E+00
gene31507	69237	NC_000079.6	Gtpbp4	1.93E+00
gene36420	223648	NC_000081.6	Ccdc166	1.93E+00
gene15864	232414	NC_000072.6	Clec9a	1.93E+00
gene42951	226178	NC_000085.6	Wbp1l	1.93E+00
gene7434	107650	NC_000069.6	Pi4kb	1.93E+00
gene15337	66892	NC_000072.6	Eif4e3	1.93E+00
gene13152	18984	NC_000071.6	Por	1.93E+00
gene13589	667510	NC_000071.6	Gm8675	1.92E+00
gene36199	68260	NC_000081.6	Trmt12	1.92E+00
gene22051	13479	NC_000074.6	Dpep1	1.92E+00
gene16105	66964	NC_000072.6	Golt1b	1.92E+00
gene31073	16593	NC_000078.6	Klc1	1.92E+00
gene33875	55946	NC_000080.6	Ap3m1	1.92E+00
gene40906	14423	NC_000084.6	Galnt1	1.92E+00
gene30386	13665	NC_000078.6	Eif2s1	1.92E+00
gene15690	232371	NC_000072.6	C1rl	1.92E+00
gene27776	216850	NC_000077.6	Kdm6b	1.92E+00
gene32515	218271	NC_000079.6	B4galt7	1.92E+00
gene5781	17395	NC_000068.7	Mmp9	1.92E+00
gene43112	107368	NC_000085.6	Pdzd8	1.92E+00
gene37166	15370	NC_000081.6	Nr4a1	1.92E+00
gene9947	67728	NC_000070.6	Dph2	1.92E+00
gene26443	237615	NC_000076.6	Ankrd52	1.91E+00
gene5828	20853	NC_000068.7	Stau1	1.91E+00
gene25227	70423	NC_000076.6	Tspan15	1.91E+00
gene3048	215632	NC_000068.7	Psd4	1.91E+00
gene28100	216964	NC_000077.6	Trp53i13	1.91E+00
gene5768	101113	NC_000068.7	Snx21	1.91E+00
gene23090	382137	NC_000075.6	Fdxacb1	1.91E+00
gene7789	229707	NC_000069.6	Strip1	1.90E+00
gene28587	217140	NC_000077.6	Scrn2	1.90E+00
gene15151	57905	NC_000072.6	Isy1	1.90E+00
gene21663	234686	NC_000074.6	Fhod1	1.90E+00
gene23508	11487	NC_000075.6	Adam10	1.90E+00

gene40470	13078	NC_000083.6	Cyp1b1	1.90E+00
gene10261	1E+08	NC_000070.6	Dcdc2b	1.90E+00
gene36659	214669	NC_000081.6	L3mbtl2	1.90E+00
gene40967	225339	NC_000084.6	Ammecr1l	1.90E+00
gene41341	1E+08	NC_000084.6	Gm33376	1.90E+00
gene42101	1.1E+08	NC_000085.6	Gm41803	1.90E+00
gene10084	56031	NC_000070.6	Ppie	1.90E+00
gene16422	232853	NC_000073.6	Zfp954	1.90E+00
gene1914	15064	NC_000067.6	Mr1	1.90E+00
gene42815	18648	NC_000085.6	Pgam1	1.90E+00
gene28021	27364	NC_000077.6	Srr	1.90E+00
gene18859	1.1E+08	NC_000073.6	Gm39062	1.90E+00
gene9762	67305	NC_000070.6	Gpx7	1.90E+00
gene6164	228998	NC_000068.7	Arfgap1	1.89E+00
gene3212	69327	NC_000068.7	1700007K13Rik	1.89E+00
gene42226	68642	NC_000085.6	Tmem216	1.89E+00
gene21469	270084	NC_000074.6	Lpcat2	1.89E+00
gene6422	320024	NC_000069.6	Nceh1	1.89E+00
gene40360	64898	NC_000083.6	Lpin2	1.89E+00
gene21078	26457	NC_000074.6	Slc27a1	1.89E+00
gene10934	100129	NC_000070.6	Gpr153	1.89E+00
gene14445	1E+08	NC_000072.6	Mira	1.89E+00
gene10643	12371	NC_000070.6	Casp9	1.89E+00
gene32101	20719	NC_000079.6	Serpinb6a	1.89E+00
gene39395	68816	NC_000083.6	Ppil1	1.89E+00
gene3026	11308	NC_000068.7	Abi1	1.89E+00
gene21672	15484	NC_000074.6	Hsd11b2	1.89E+00
gene12608	1E+08	NC_000071.6	Gm15559	1.89E+00
gene37972	320184	NC_000082.6	Lrrc58	1.89E+00
gene21645	667774	NC_000074.6	Gm8804	1.89E+00
gene26237	69181	NC_000076.6	Dyrk2	1.88E+00
gene27520	216805	NC_000077.6	Flcn	1.88E+00
gene2748	109079	NC_000068.7	Sephs1	1.88E+00
gene8630	14590	NC_000070.6	Ggh	1.88E+00
gene28195	216987	NC_000077.6	Utp6	1.88E+00
gene36290	17988	NC_000081.6	Ndrg1	1.88E+00
gene3442	67889	NC_000068.7	Rbm18	1.88E+00
gene2411	320404	NC_000067.6	Itpkb	1.88E+00
gene20561	666630	NC_000074.6	Gm8201	1.88E+00
gene21443	19651	NC_000074.6	Rbl2	1.88E+00
gene33839	68045	NC_000080.6	2700060E02Rik	1.88E+00
gene36605	54152	NC_000081.6	Dnal4	1.88E+00
gene14492	107607	NC_000072.6	Nod1	1.88E+00
gene162	665564	NC_000067.6	Gm7690	1.88E+00
gene34032	76485	NC_000080.6	Glt8d1	1.88E+00
gene26442	210582	NC_000076.6	Coq10a	1.87E+00
gene3244	68205	NC_000068.7	Urm1	1.87E+00
gene16871	13400	NC_000073.6	Dmpk	1.87E+00

gene37111	83797	NC_000081.6	Smarcd1	1.87E+00
gene30071	72183	NC_000078.6	Snx6	1.87E+00
gene23560	11891	NC_000075.6	Rab27a	1.87E+00
gene3273	57170	NC_000068.7	Dolpp1	1.87E+00
gene26306	216395	NC_000076.6	Tmem5	1.87E+00
gene35874	223433	NC_000081.6	Fam105a	1.87E+00
gene42115	70999	NC_000085.6	Naa40	1.87E+00
gene30922	70059	NC_000078.6	Degs2	1.87E+00
gene18819	18442	NC_000073.6	P2ry2	1.87E+00
gene17347	60594	NC_000073.6	Capn12	1.87E+00
gene6471	18759	NC_000069.6	Prkci	1.87E+00
gene27234	26420	NC_000077.6	Mapk9	1.87E+00
gene10436	100017	NC_000070.6	Ldlrap1	1.87E+00
gene717	18187	NC_000067.6	Nrp2	1.87E+00
gene21652	50788	NC_000074.6	Fbxl8	1.87E+00
gene8932	230103	NC_000070.6	Npr2	1.87E+00
gene2033	14261	NC_000067.6	Fmo1	1.87E+00
gene18279	=Gene;ger	NC_000073.6	LOC108167435	1.87E+00
gene14052	19331	NC_000072.6	Rab19	1.87E+00
gene36456	12181	NC_000081.6	Bop1	1.87E+00
gene15907	16633	NC_000072.6	Klra2	1.87E+00
gene21289	212139	NC_000074.6	Cc2d1a	1.87E+00
gene2488	71678	NC_000067.6	Brox	1.86E+00
gene19276	50781	NC_000073.6	Dkk3	1.86E+00
gene22444	319278	NC_000075.6	A230050P20Rik	1.86E+00
gene3628	56194	NC_000068.7	Prpf40a	1.86E+00
gene15498	110157	NC_000072.6	Raf1	1.86E+00
gene10015	666766	NC_000070.6	Gm12955	1.86E+00
gene7472	229593	NC_000069.6	Golph3l	1.86E+00
gene37603	22230	NC_000082.6	Ufd1l	1.86E+00
gene42266	433221	NC_000085.6	Gm5511	1.86E+00
gene2107	71592	NC_000067.6	Pogk	1.86E+00
gene6759	71182	NC_000069.6	4933417G07Rik	1.86E+00
gene10360	230789	NC_000070.6	Fam76a	1.86E+00
gene39431	11307	NC_000083.6	Abcg1	1.86E+00
gene2490	338366	NC_000067.6	Mia3	1.86E+00
gene1783	69367	NC_000067.6	Glrx2	1.86E+00
gene32595	105348	NC_000079.6	Golm1	1.86E+00
gene4999	99138	NC_000068.7	Stard7	1.86E+00
gene17129	16534	NC_000073.6	Kcnn4	1.86E+00
gene12905	68948	NC_000071.6	Fam216a	1.86E+00
gene42850	14718	NC_000085.6	Got1	1.86E+00
gene23383	26395	NC_000075.6	Map2k1	1.86E+00
gene18273	68981	NC_000073.6	Snrpa1	1.86E+00
gene7566	229615	NC_000069.6	Pias3	1.86E+00
gene38156	80859	NC_000082.6	Nfkbiz	1.86E+00
gene9278	66783	NC_000070.6	Aknaos	1.85E+00
gene11530	621407	NC_000071.6	Gm9970	1.85E+00

gene43065	66866	NC_000085.6	Nhlrc2	1.85E+00
gene10355	19891	NC_000070.6	Rpa2	1.85E+00
gene10266	16650	NC_000070.6	Kpna6	1.85E+00
gene37748	12053	NC_000082.6	Bcl6	1.85E+00
gene30878	12061	NC_000078.6	Bdkrb1	1.85E+00
gene7267	229541	NC_000069.6	Dennd4b	1.85E+00
gene36520	71898	NC_000081.6	Apol9b	1.85E+00
gene21681	13018	NC_000074.6	Ctcf	1.85E+00
gene12239	78088	NC_000071.6	Sowahb	1.85E+00
gene29176	276905	NC_000077.6	Armc7	1.85E+00
gene20481	73754	NC_000074.6	Thap1	1.85E+00
gene10004	56401	NC_000070.6	P3h1	1.85E+00
gene40476	72692	NC_000083.6	Hnrnpll	1.84E+00
gene37330	246154	NC_000082.6	Vasn	1.84E+00
gene22963	76568	NC_000075.6	Ift46	1.84E+00
gene16203	67456	NC_000072.6	Ergic2	1.84E+00
gene17173	67379	NC_000073.6	Dedd2	1.84E+00
gene29240	20446	NC_000077.6	St6galnac2	1.84E+00
gene3197	1E+08	NC_000068.7	Gm20071	1.84E+00
gene5744	52840	NC_000068.7	Dbndd2	1.84E+00
gene33985	1E+08	NC_000080.6	Gm34702	1.84E+00
gene25210	1E+08	NC_000076.6	Gm17829	1.84E+00
gene28017	17428	NC_000077.6	Mnt	1.84E+00
gene15044	232164	NC_000072.6	Paip2b	1.84E+00
gene21825	434350	NC_000074.6	9430091E24Rik	1.84E+00
gene37568	27886	NC_000082.6	Dgcr14	1.84E+00
gene3344	71254	NC_000068.7	Naif1	1.84E+00
gene39534	407803	NC_000083.6	BC051226	1.84E+00
gene22016	1E+08	NC_000074.6	9330133O14Rik	1.84E+00
gene14689	723993	NC_000072.6	Gm9729	1.83E+00
gene2382	66359	NC_000067.6	Cox20	1.83E+00
gene29293	1E+08	NC_000077.6	Gm11747	1.83E+00
gene3788	228005	NC_000068.7	Ppig	1.83E+00
gene20616	67276	NC_000074.6	Eri1	1.83E+00
gene40153	106766	NC_000083.6	Stap2	1.83E+00
gene6864	11416	NC_000069.6	Slc33a1	1.83E+00
gene10131	16330	NC_000070.6	Inpp5b	1.83E+00
gene12740	52064	NC_000071.6	Coq5	1.83E+00
gene12932	23912	NC_000071.6	Rhof	1.83E+00
gene28627	68127	NC_000077.6	B230217C12Rik	1.83E+00
gene36823	223753	NC_000081.6	Cerk	1.83E+00
gene194	14859	NC_000067.6	Gsta3	1.83E+00
gene728	227197	NC_000067.6	Ndufs1	1.83E+00
gene39339	56409	NC_000083.6	Nudt3	1.83E+00
gene10820	14349	NC_000070.6	Fv1	1.83E+00
gene7234	319945	NC_000069.6	Flad1	1.83E+00
gene6898	99929	NC_000069.6	Tiparp	1.83E+00
gene27929	216892	NC_000077.6	Spns2	1.83E+00

gene37565	13356	NC_000082.6	Dgcr2	1.83E+00
gene25709	50721	NC_000076.6	Sirt6	1.83E+00
gene28774	14230	NC_000077.6	Fkbp10	1.83E+00
gene30429	328133	NC_000078.6	Slc39a9	1.83E+00
gene7423	20342	NC_000069.6	Selenbp2	1.82E+00
gene22527	235047	NC_000075.6	Zfp809	1.82E+00
gene28824	69684	NC_000077.6	Aarsd1	1.82E+00
gene11894	22235	NC_000071.6	Ugdh	1.82E+00
gene15696	14792	NC_000072.6	Lpcat3	1.82E+00
gene2054	69962	NC_000067.6	Mettl18	1.82E+00
gene40361	246707	NC_000083.6	Emilin2	1.82E+00
gene1588	98396	NC_000067.6	Slc41a1	1.82E+00
gene1664	1E+08	NC_000067.6	Gm32267	1.82E+00
gene20059	1E+08	NC_000074.6	Fcor	1.82E+00
gene16319	232811	NC_000073.6	Suv420h2	1.82E+00
gene2509	67247	NC_000067.6	Marc2	1.82E+00
gene27846	1.1E+08	NC_000077.6	Gm40191	1.82E+00
gene22954	80288	NC_000075.6	Bcl9l	1.82E+00
gene32288	105193	NC_000079.6	Nhlrc1	1.82E+00
gene28783	56354	NC_000077.6	Dnajc7	1.82E+00
gene10875	18707	NC_000070.6	Pik3cd	1.82E+00
gene20502	66653	NC_000074.6	Brf2	1.82E+00
gene39796	258052	NC_000083.6	Olfr753-ps1	1.82E+00
gene41097	70791	NC_000084.6	Hars2	1.82E+00
gene14924	74287	NC_000072.6	Kcmf1	1.82E+00
gene32618	1.1E+08	NC_000079.6	Gm40974	1.82E+00
gene21677	75687	NC_000074.6	Fam65a	1.81E+00
gene29977	67452	NC_000078.6	Pnpla8	1.81E+00
gene11363	12568	NC_000071.6	Cdk5	1.81E+00
gene42812	212398	NC_000085.6	Frat2	1.81E+00
gene20567	14782	NC_000074.6	Gsr	1.81E+00
gene19751	67765	NC_000073.6	5830432E09Rik	1.81E+00
gene24547	320642	NC_000076.6	A630066F11Rik	1.81E+00
gene37317	68015	NC_000082.6	Trap1	1.81E+00
gene37331	83945	NC_000082.6	Dnaja3	1.81E+00
gene27729	442801	NC_000077.6	Arhgef15	1.81E+00
gene13774	21753	NC_000072.6	Tes	1.81E+00
gene3929	78802	NC_000068.7	Ttc30a1	1.81E+00
gene8359	170822	NC_000069.6	Usp33	1.81E+00
gene20565	108159	NC_000074.6	Ubxn8	1.81E+00
gene1221	67273	NC_000067.6	Ndufa10	1.81E+00
gene5050	66170	NC_000068.7	Chchd5	1.81E+00
gene31460	105387	NC_000079.6	Akr1c14	1.81E+00
gene10124	67205	NC_000070.6	Utp11l	1.81E+00
gene18409	68048	NC_000073.6	Aen	1.81E+00
gene2266	98365	NC_000067.6	Slamf9	1.80E+00
gene9194	22234	NC_000070.6	Ugcg	1.80E+00
gene6904	56706	NC_000069.6	Ccnl1	1.80E+00

gene28925	544817	NC_000077.6	Arhgap27	1.80E+00
gene8232	229906	NC_000069.6	Gtf2b	1.80E+00
gene26155	67226	NC_000076.6	Tmem19	1.80E+00
gene29543	67241	NC_000078.6	Smc6	1.80E+00
gene33255	320893	NC_000079.6	6430562O15Rik	1.80E+00
gene19660	546005	NC_000073.6	Gm5903	1.80E+00
gene5592	68295	NC_000068.7	Aar2	1.80E+00
gene30880	66787	NC_000078.6	Gskip	1.80E+00
gene5478	99237	NC_000068.7	Tm9sf4	1.80E+00
gene16771	70394	NC_000073.6	Kptn	1.80E+00
gene21710	69771	NC_000074.6	1810019D21Rik	1.80E+00
gene42067	329015	NC_000085.6	Atg2a	1.80E+00
gene32123	19134	NC_000079.6	Prpf4b	1.80E+00
gene7559	280411	NC_000069.6	Lix1l	1.80E+00
gene28314	217039	NC_000077.6	Ggnbp2	1.80E+00
gene41002	27528	NC_000084.6	Nrep	1.80E+00
gene14890	66223	NC_000072.6	Mrpl35	1.79E+00
gene39191	16898	NC_000083.6	Rps2	1.79E+00
gene21006	12931	NC_000074.6	Crlf1	1.79E+00
gene8825	67615	NC_000070.6	Ube2r2	1.79E+00
gene34168	52588	NC_000080.6	Tspan14	1.79E+00
gene4807	56357	NC_000068.7	Ivd	1.79E+00
gene37056	12297	NC_000081.6	Cacnb3	1.79E+00
gene8387	11364	NC_000069.6	Acadm	1.79E+00
gene26427	116848	NC_000076.6	Baz2a	1.79E+00
gene20979	74549	NC_000074.6	Mau2	1.79E+00
gene6943	66949	NC_000069.6	Trim59	1.79E+00
gene28108	360013	NC_000077.6	Myo18a	1.79E+00
gene16419	1E+08	NC_000073.6	Pafah1b1-ps2	1.79E+00
gene12098	231386	NC_000071.6	Ythdc1	1.79E+00
gene13078	56715	NC_000071.6	Rabgef1	1.79E+00
gene17267	1.1E+08	NC_000073.6	Gm15541	1.79E+00
gene24047	59092	NC_000075.6	Pcbp4	1.79E+00
gene27854	75273	NC_000077.6	Pelp1	1.79E+00
gene5353	383754	NC_000068.7	Gm14132	1.79E+00
gene14064	665254	NC_000072.6	Gm7556	1.79E+00
gene20400	102032	NC_000074.6	Smim19	1.79E+00
gene20738	654824	NC_000074.6	Ankrd37	1.79E+00
gene10050	69780	NC_000070.6	Smap2	1.79E+00
gene17276	269881	NC_000073.6	Map3k10	1.79E+00
gene38834	436440	NC_000083.6	Gpr31b	1.79E+00
gene4573	57443	NC_000068.7	Fbxo3	1.78E+00
gene12246	1E+08	NC_000071.6	Kat2b-ps	1.78E+00
gene19584	320683	NC_000073.6	Zfp629	1.78E+00
gene32284	218210	NC_000079.6	Nup153	1.78E+00
gene43045	66980	NC_000085.6	Zdhhc6	1.78E+00
gene4626	212772	NC_000068.7	Arl14ep	1.78E+00
gene13897	319757	NC_000072.6	Smo	1.78E+00

gene39692	68915	NC_000083.6	Vars2	1.78E+00
gene1797	667877	NC_000067.6	Gm8856	1.78E+00
gene22140	69551	NC_000074.6	2310022B05Rik	1.78E+00
gene34786	28200	NC_000080.6	Dhrs4	1.78E+00
gene22132	108148	NC_000074.6	Galnt2	1.78E+00
gene13766	16543	NC_000072.6	Mdfic	1.78E+00
gene9083	666724	NC_000070.6	Gm12475	1.78E+00
gene38062	74102	NC_000082.6	Slc35a5	1.78E+00
gene34957	66597	NC_000080.6	Trim13	1.78E+00
gene3153	18128	NC_000068.7	Notch1	1.78E+00
gene36425	105782	NC_000081.6	Scrib	1.78E+00
gene38601	13185	NC_000082.6	Dscr3	1.78E+00
gene36611	80287	NC_000081.6	Apobec3	1.78E+00
gene2296	13349	NC_000067.6	Ackr1	1.77E+00
gene31981	380836	NC_000079.6	Mrs2	1.77E+00
gene41961	12460	NC_000085.6	Ccs	1.77E+00
gene38900	1E+08	NC_000083.6	Gm7177	1.77E+00
gene2084	70456	NC_000067.6	Mpc2	1.77E+00
gene42017	381201	NC_000085.6	Ap5b1	1.77E+00
gene32550	17129	NC_000079.6	Smad5	1.77E+00
gene28573	56358	NC_000077.6	Copz2	1.77E+00
gene27430	667947	NC_000077.6	Gm12247	1.77E+00
gene11307	67211	NC_000071.6	Armc10	1.77E+00
gene36938	67197	NC_000081.6	Zcrb1	1.77E+00
gene16307	436022	NC_000073.6	Dnaaf3	1.77E+00
gene26405	320080	NC_000076.6	Zbtb39	1.77E+00
gene20013	1E+08	NC_000073.6	Gm35626	1.77E+00
gene10174	230753	NC_000070.6	Thrap3	1.77E+00
gene28525	268469	NC_000077.6	Zfp652	1.77E+00
gene19576	1E+08	NC_000073.6	Srcap	1.76E+00
gene5521	13555	NC_000068.7	E2f1	1.76E+00
gene8303	70285	NC_000069.6	Rpf1	1.76E+00
gene18541	70178	NC_000073.6	Abhd17c	1.76E+00
gene41911	71670	NC_000085.6	Acy3	1.76E+00
gene11524	71752	NC_000071.6	Gtf3c2	1.76E+00
gene23360	69478	NC_000075.6	2300009A05Rik	1.76E+00
gene37234	21357	NC_000081.6	Tarbp2	1.76E+00
gene1063	1E+08	NC_000067.6	Gm18180	1.76E+00
gene33542	1E+08	NC_000079.6	Gm21188	1.76E+00
gene30522	68497	NC_000078.6	Arel1	1.76E+00
gene13324	68017	NC_000071.6	Mrm2	1.76E+00
gene16876	67369	NC_000073.6	Qpctl	1.76E+00
gene37381	1E+08	NC_000082.6	Gm18722	1.76E+00
gene38183	52633	NC_000082.6	Nit2	1.76E+00
gene30586	104799	NC_000078.6	Vipas39	1.76E+00
gene10327	213541	NC_000070.6	Ythdf2	1.76E+00
gene15716	14788	NC_000072.6	Gpr162	1.76E+00
gene10126	75062	NC_000070.6	Sf3a3	1.76E+00

gene13436	71799	NC_000071.6	Ptcd1	1.76E+00
gene25611	70248	NC_000076.6	Dazap1	1.76E+00
gene12643	22022	NC_000071.6	Tpst2	1.76E+00
gene1551	13136	NC_000067.6	Cd55	1.75E+00
gene40036	74123	NC_000083.6	Foxp4	1.75E+00
gene24597	353258	NC_000076.6	Ltv1	1.75E+00
gene30390	268567	NC_000078.6	Tmem229b	1.75E+00
gene8666	68493	NC_000070.6	Ndufaf4	1.75E+00
gene1028	14897	NC_000067.6	Trip12	1.75E+00
gene39899	17850	NC_000083.6	Mut	1.75E+00
gene8998	272027	NC_000070.6	Tstd2	1.75E+00
gene36665	21685	NC_000081.6	Tef	1.75E+00
gene1690	21854	NC_000067.6	Timm17a	1.75E+00
gene22201	18186	NC_000074.6	Nrp1	1.75E+00
gene30504	77219	NC_000078.6	Ptgr2	1.75E+00
gene35958	30945	NC_000081.6	Rnf19a	1.75E+00
gene13817	668022	NC_000072.6	Gm8930	1.75E+00
gene24494	666541	NC_000076.6	Gm8155	1.74E+00
gene28172	1E+08	NC_000077.6	Gm30470	1.74E+00
gene32310	15904	NC_000079.6	Id4	1.74E+00
gene13437	54188	NC_000071.6	Cpsf4	1.74E+00
gene27326	93736	NC_000077.6	Aff4	1.74E+00
gene11464	433864	NC_000071.6	Nom1	1.74E+00
gene10171	74178	NC_000070.6	Stk40	1.74E+00
gene31603	67008	NC_000079.6	Yae1d1	1.74E+00
gene16879	72205	NC_000073.6	Eml2	1.74E+00
gene20390	70160	NC_000074.6	Vps36	1.74E+00
gene6190	68556	NC_000068.7	Uckl1	1.74E+00
gene24288	333433	NC_000075.6	Gpd1l	1.74E+00
gene23350	677044	NC_000075.6	Gm10653	1.74E+00
gene10225	54383	NC_000070.6	Phc2	1.74E+00
gene25417	70377	NC_000076.6	Derl3	1.74E+00
gene3427	14594	NC_000068.7	Ggta1	1.74E+00
gene11607	24055	NC_000071.6	Sh3bp2	1.74E+00
gene1308	52392	NC_000067.6	D1Ertd622e	1.73E+00
gene7685	70747	NC_000069.6	Tspan2	1.73E+00
gene17175	210172	NC_000073.6	Zfp526	1.73E+00
gene12883	17165	NC_000071.6	Mapkapk5	1.73E+00
gene8433	68151	NC_000069.6	Wls	1.73E+00
gene20224	101994	NC_000074.6	Champ1	1.73E+00
gene18254	67973	NC_000073.6	Mphosph10	1.73E+00
gene4958	110074	NC_000068.7	Dut	1.73E+00
gene35279	21807	NC_000080.6	Tsc22d1	1.73E+00
gene13706	94192	NC_000072.6	C1galt1	1.73E+00
gene19580	=Gene;ger	NC_000073.6	LOC108167514	1.73E+00
gene32034	=Gene;ger	NC_000079.6	LOC108168071	1.73E+00
gene37396	383103	NC_000082.6	Tvp23a	1.73E+00
gene22648	117149	NC_000075.6	Tirap	1.73E+00

gene8626	242341	NC_000070.6	Atp6v0d2	1.73E+00
gene36494	71939	NC_000081.6	Apol6	1.73E+00
gene8110	13722	NC_000069.6	Aimp1	1.72E+00
gene11676	17701	NC_000071.6	Msx1	1.72E+00
gene29145	217305	NC_000077.6	Cd300ld	1.72E+00
gene6122	66730	NC_000068.7	4921531C22Rik	1.72E+00
gene18291	75099	NC_000073.6	Lysmd4	1.72E+00
gene38689	70544	NC_000083.6	Tmem242	1.72E+00
gene21805	244654	NC_000074.6	Mtss1l	1.72E+00
gene41557	14151	NC_000084.6	Fech	1.72E+00
gene29901	11622	NC_000078.6	Ahr	1.72E+00
gene37530	66053	NC_000082.6	Ppil2	1.72E+00
gene28892	52715	NC_000077.6	Ccdc43	1.72E+00
gene16082	11569	NC_000072.6	Aebp2	1.72E+00
gene29276	11799	NC_000077.6	Birc5	1.72E+00
gene29412	66840	NC_000077.6	Wdr45b	1.71E+00
gene13107	14886	NC_000071.6	Gtf2i	1.71E+00
gene26374	52666	NC_000076.6	Arhgef25	1.71E+00
gene22268	22601	NC_000075.6	Yap1	1.71E+00
gene15735	110109	NC_000072.6	Nop2	1.71E+00
gene19716	76683	NC_000073.6	1500002F19Rik	1.71E+00
gene5830	228889	NC_000068.7	Ddx27	1.71E+00
gene39589	18132	NC_000083.6	Notch4	1.71E+00
gene41362	67804	NC_000084.6	Snx2	1.71E+00
gene42931	226169	NC_000085.6	Pprc1	1.71E+00
gene16926	52118	NC_000073.6	Pvr	1.71E+00
gene29190	71947	NC_000077.6	Tmem94	1.71E+00
gene10990	668173	NC_000070.6	Pex10	1.71E+00
gene12713	26431	NC_000071.6	Git2	1.71E+00
gene34003	56249	NC_000080.6	Actr8	1.71E+00
gene35620	70572	NC_000080.6	Ipo5	1.71E+00
gene39622	68763	NC_000083.6	1110038B12Rik	1.71E+00
gene20969	78816	NC_000074.6	Gmip	1.71E+00
gene27934	11736	NC_000077.6	Ankfy1	1.71E+00
gene39693	14885	NC_000083.6	Gtf2h4	1.71E+00
gene15520	71779	NC_000072.6	March8	1.71E+00
gene19265	22083	NC_000073.6	Ctr9	1.71E+00
gene35061	67179	NC_000080.6	Ccdc25	1.71E+00
gene19483	60504	NC_000073.6	Il21r	1.71E+00
gene29559	70858	NC_000078.6	4921511I17Rik	1.71E+00
gene3485	1E+08	NC_000068.7	Gm13435	1.70E+00
gene17203	12039	NC_000073.6	Bckdha	1.70E+00
gene12648	1.1E+08	NC_000071.6	Gm20636	1.70E+00
gene25433	15468	NC_000076.6	Prmt2	1.70E+00
gene37310	73261	NC_000082.6	1700037C18Rik	1.70E+00
gene37117	71949	NC_000081.6	Cers5	1.70E+00
gene25361	52637	NC_000076.6	Cisd1	1.70E+00
gene24363	67561	NC_000075.6	Wdr48	1.70E+00

gene31058	217866	NC_000078.6	Cdc42bpb	1.70E+00
gene5523	59038	NC_000068.7	Pxmp4	1.70E+00
gene40054	328833	NC_000083.6	Treml2	1.70E+00
gene37863	66061	NC_000082.6	Tctex1d2	1.70E+00
gene38819	635617	NC_000083.6	Gm7162	1.70E+00
gene21236	30932	NC_000074.6	Zfp330	1.70E+00
gene23258	69106	NC_000075.6	Stoml1	1.70E+00
gene8069	68147	NC_000069.6	Gar1	1.70E+00
gene24947	1E+08	NC_000076.6	Gm17795	1.70E+00
gene30487	26897	NC_000078.6	Acot1	1.70E+00
gene28158	69071	NC_000077.6	Tmem97	1.70E+00
gene27508	268417	NC_000077.6	Zkscan17	1.69E+00
gene16005	15199	NC_000072.6	Hebp1	1.69E+00
gene36444	75475	NC_000081.6	Oplah	1.69E+00
gene7705	140917	NC_000069.6	Dclre1b	1.69E+00
gene22480	13430	NC_000075.6	Dnm2	1.69E+00
gene28474	67684	NC_000077.6	Luc7l3	1.69E+00
gene41538	108123	NC_000084.6	Napg	1.69E+00
gene28775	68106	NC_000077.6	Nt5c3b	1.69E+00
gene32472	76577	NC_000079.6	Faf2	1.69E+00
gene25642	71564	NC_000076.6	Izumo4	1.69E+00
gene5971	56190	NC_000068.7	Rbm38	1.69E+00
gene10335	1E+08	NC_000070.6	Snhg12	1.69E+00
gene16894	232943	NC_000073.6	Klc3	1.69E+00
gene7706	1E+08	NC_000069.6	Gm15471	1.69E+00
gene19905	98845	NC_000073.6	Eps8l2	1.69E+00
gene37880	69823	NC_000082.6	Fyttd1	1.69E+00
gene36727	74039	NC_000081.6	Nfam1	1.69E+00
gene630	68115	NC_000067.6	9430016H08Rik	1.69E+00
gene17318	233033	NC_000073.6	Samd4b	1.69E+00
gene24466	83493	NC_000075.6	Sacm1l	1.68E+00
gene3351	11636	NC_000068.7	Ak1	1.68E+00
gene39965	68565	NC_000083.6	Mrps18a	1.68E+00
gene8797	11428	NC_000070.6	Aco1	1.68E+00
gene11735	12494	NC_000071.6	Cd38	1.68E+00
gene8619	70568	NC_000070.6	Cpne3	1.68E+00
gene22043	270106	NC_000074.6	Rpl13	1.68E+00
gene34267	624862	NC_000080.6	Gm6532	1.68E+00
gene41780	66943	NC_000084.6	Pqlc1	1.68E+00
gene16325	19943	NC_000073.6	Rpl28	1.68E+00
gene17870	101568	NC_000073.6	Vrk3	1.68E+00
gene36867	19094	NC_000081.6	Mapk11	1.68E+00
gene37999	pseudogene	NC_000082.6	LOC100534330	1.68E+00
gene585	1E+08	NC_000067.6	Gm32311	1.68E+00
gene19825	69064	NC_000073.6	Fuom	1.68E+00
gene39966	75564	NC_000083.6	Rsph9	1.68E+00
gene423	26921	NC_000067.6	Map4k4	1.68E+00
gene22334	234959	NC_000075.6	Med17	1.68E+00

gene39178	18763	NC_000083.6	Pkd1	1.67E+00
gene2622	226856	NC_000067.6	Lpgat1	1.67E+00
gene27294	76901	NC_000077.6	Jade2	1.67E+00
gene38112	1E+08	NC_000082.6	Gm18694	1.67E+00
gene38554	68936	NC_000082.6	Smim11	1.67E+00
gene25107	66403	NC_000076.6	Asf1a	1.67E+00
gene5619	69161	NC_000068.7	Manbal	1.67E+00
gene10404	67422	NC_000070.6	Dhdds	1.67E+00
gene24204	235627	NC_000075.6	Nbeal2	1.67E+00
gene12611	22241	NC_000071.6	Ulk1	1.67E+00
gene29611	50496	NC_000078.6	E2f6	1.67E+00
gene27801	11443	NC_000077.6	Chrnb1	1.67E+00
gene3027	74159	NC_000068.7	Acbd5	1.67E+00
gene22252	17392	NC_000075.6	Mmp3	1.67E+00
gene20221	69957	NC_000074.6	Cdc16	1.67E+00
gene36813	72355	NC_000081.6	Cdpf1	1.67E+00
gene1576	56489	NC_000067.6	Ikbke	1.67E+00
gene20132	24099	NC_000074.6	Tnfsf13b	1.66E+00
gene11969	75991	NC_000071.6	Slain2	1.66E+00
gene1884	24014	NC_000067.6	Rnasel	1.66E+00
gene34422	66246	NC_000080.6	Osgep	1.66E+00
gene22980	270152	NC_000075.6	Amica1	1.66E+00
gene33025	666883	NC_000079.6	Gm8345	1.66E+00
gene24097	83486	NC_000075.6	Rbm5	1.66E+00
gene13173	71735	NC_000071.6	Lrwd1	1.66E+00
gene2472	67948	NC_000067.6	Fbxo28	1.66E+00
gene6491	67778	NC_000069.6	Zfp639	1.66E+00
gene7253	74383	NC_000069.6	Ubap2l	1.66E+00
gene37858	70238	NC_000082.6	Rnf168	1.66E+00
gene9858	230641	NC_000070.6	Gm12833	1.66E+00
gene32075	1E+08	NC_000079.6	Gm36181	1.66E+00
gene35851	70350	NC_000081.6	Basp1	1.66E+00
gene24582	71865	NC_000076.6	Fbxo30	1.66E+00
gene37682	76222	NC_000082.6	Magef1	1.65E+00
gene10353	381560	NC_000070.6	Xkr8	1.65E+00
gene41929	58988	NC_000085.6	Rps6kb2	1.65E+00
gene25441	216119	NC_000076.6	Ybey	1.65E+00
gene23820	18828	NC_000075.6	Plscr2	1.65E+00
gene19864	76974	NC_000073.6	Urah	1.65E+00
gene35679	106052	NC_000081.6	Fbxo4	1.65E+00
gene37141	68614	NC_000081.6	Letmd1	1.65E+00
gene3970	16401	NC_000068.7	Itga4	1.65E+00
gene28065	276919	NC_000077.6	Gemin4	1.65E+00
gene35307	219181	NC_000080.6	Akap11	1.65E+00
gene35065	1E+08	NC_000080.6	Gm19222	1.65E+00
gene39726	667782	NC_000083.6	Gm8810	1.65E+00
gene1508	74150	NC_000067.6	Slc35f5	1.65E+00
gene28034	68275	NC_000077.6	Rpa1	1.65E+00

gene23506	235461	NC_000075.6	Fam63b	1.65E+00
gene3073	68222	NC_000068.7	Fam166a	1.65E+00
gene36032	1.1E+08	NC_000081.6	Gm41310	1.65E+00
gene34149	382871	NC_000080.6	Gm5203	1.65E+00
gene29692	54451	NC_000078.6	Cpsf3	1.65E+00
gene25189	71279	NC_000076.6	Slc29a3	1.64E+00
gene14880	68477	NC_000072.6	Rmnd5a	1.64E+00
gene17249	12587	NC_000073.6	Mia	1.64E+00
gene27542	13495	NC_000077.6	Drg2	1.64E+00
gene6191	1E+08	NC_000068.7	Uckl1os	1.64E+00
gene29537	668435	NC_000078.6	Pgk1-rs7	1.64E+00
gene37481	66086	NC_000082.6	Fopnl	1.64E+00
gene37148	668218	NC_000081.6	Bin2	1.64E+00
gene37696	75826	NC_000082.6	Senp2	1.64E+00
gene12612	100900	NC_000071.6	Hscb	1.64E+00
gene18662	68472	NC_000073.6	Tmem126b	1.64E+00
gene37552	224022	NC_000082.6	Slc7a4	1.64E+00
gene42858	246696	NC_000085.6	Slc25a28	1.64E+00
gene37954	224139	NC_000082.6	Golgb1	1.64E+00
gene15231	=Gene;ger	NC_000072.6	LOC102631993	1.64E+00
gene23031	1E+08	NC_000075.6	Gm10677	1.64E+00
gene7497	229603	NC_000069.6	Otud7b	1.64E+00
gene28540	27681	NC_000077.6	Snf8	1.64E+00
gene28357	217057	NC_000077.6	Ptrh2	1.64E+00
gene10146	75563	NC_000070.6	Dnali1	1.63E+00
gene410	114641	NC_000067.6	Rpl31	1.63E+00
gene7740	53975	NC_000069.6	Ddx20	1.63E+00
gene5484	16569	NC_000068.7	Kif3b	1.63E+00
gene39080	240041	NC_000083.6	Zfp945	1.63E+00
gene21834	52815	NC_000074.6	Ldhd	1.63E+00
gene24991	140740	NC_000076.6	Sec63	1.63E+00
gene29695	22630	NC_000078.6	Ywhaq	1.63E+00
gene4939	22785	NC_000068.7	Slc30a4	1.63E+00
gene22537	55934	NC_000075.6	Rp9	1.63E+00
gene8480	71639	NC_000070.6	4930430E12Rik	1.63E+00
gene12906	68080	NC_000071.6	Gpn3	1.63E+00
gene41775	LOC10524	NC_000084.6	LOC105246506	1.63E+00
gene13974	12183	NC_000072.6	Bpgm	1.63E+00
gene20558	628412	NC_000074.6	Gm6877	1.63E+00
gene11808	52206	NC_000071.6	Anapc4	1.63E+00
gene24877	103511	NC_000076.6	Fam26e	1.63E+00
gene21845	93739	NC_000074.6	Gabarapl2	1.63E+00
gene17437	1E+08	NC_000073.6	Gm32082	1.63E+00
gene11588	56384	NC_000071.6	Letm1	1.63E+00
gene29228	67763	NC_000077.6	Prpsap1	1.63E+00
gene31117	217887	NC_000078.6	BC022687	1.63E+00
gene36502	223672	NC_000081.6	Apol9a	1.63E+00
gene30065	66266	NC_000078.6	Eapp	1.63E+00

gene30497	=Gene;ger	NC_000078.6	LOC108168019	1.63E+00
gene10961	1.1E+08	NC_000070.6	Gm42361	1.62E+00
gene28486	268465	NC_000077.6	Eme1	1.62E+00
gene28272	71770	NC_000077.6	Ap2b1	1.62E+00
gene30361	211151	NC_000078.6	Churc1	1.62E+00
gene1983	67647	NC_000067.6	4930523C07Rik	1.62E+00
gene21900	68133	NC_000074.6	Gcsh	1.62E+00
gene35232	629583	NC_000080.6	Gm6986	1.62E+00
gene42474	26358	NC_000085.6	Aldh1a7	1.62E+00
gene1193	16978	NC_000067.6	Lrrfip1	1.62E+00
gene35224	67381	NC_000080.6	Med4	1.62E+00
gene40462	260409	NC_000083.6	Cdc42ep3	1.62E+00
gene24550	16798	NC_000076.6	Lats1	1.62E+00
gene17415	1E+08	NC_000073.6	Gm32029	1.62E+00
gene8300	74245	NC_000069.6	Ctbs	1.62E+00
gene10453	74552	NC_000070.6	Nipal3	1.62E+00
gene34173	11720	NC_000080.6	Mat1a	1.62E+00
gene28777	217194	NC_000077.6	Klhl11	1.62E+00
gene9952	230674	NC_000070.6	Kdm4a	1.62E+00
gene19787	12176	NC_000073.6	Bnip3	1.62E+00
gene31132	116870	NC_000078.6	Mta1	1.62E+00
gene42225	269061	NC_000085.6	Cpsf7	1.62E+00
gene4799	18796	NC_000068.7	Plcb2	1.62E+00
gene7246	214547	NC_000069.6	She	1.62E+00
gene27236	59044	NC_000077.6	Rnf130	1.62E+00
gene16291	232798	NC_000073.6	Leng8	1.62E+00
gene10376	230796	NC_000070.6	Wdtc1	1.62E+00
gene30928	1E+08	NC_000078.6	Gm34220	1.61E+00
gene37138	18174	NC_000081.6	Slc11a2	1.61E+00
gene28311	217038	NC_000077.6	Mrm1	1.61E+00
gene5383	76192	NC_000068.7	Abhd12	1.61E+00
gene2899	170768	NC_000068.7	Pfkfb3	1.61E+00
gene13098	666038	NC_000071.6	Gm7902	1.61E+00
gene19439	74105	NC_000073.6	Gga2	1.61E+00
gene39476	1.1E+08	NC_000083.6	Gm41569	1.61E+00
gene17834	668661	NC_000073.6	2410002F23Rik	1.61E+00
gene40965	67453	NC_000084.6	Slc25a46	1.61E+00
gene15022	78653	NC_000072.6	Bola3	1.61E+00
gene21612	97487	NC_000074.6	Cmtm4	1.61E+00
gene3057	67187	NC_000068.7	Zmynd19	1.61E+00
gene13150	215160	NC_000071.6	Rhbdd2	1.61E+00
gene17184	269878	NC_000073.6	Megf8	1.61E+00
gene6654	545517	NC_000069.6	Gm10356	1.61E+00
gene1255	69821	NC_000067.6	Mterf4	1.61E+00
gene3510	19177	NC_000068.7	Psmb7	1.61E+00
gene705	12477	NC_000067.6	Ctla4	1.61E+00
gene30399	17252	NC_000078.6	Rdh11	1.61E+00
gene3079	78797	NC_000068.7	Ndor1	1.61E+00

gene24014	94062	NC_000075.6	Mrpl3	1.61E+00
gene42052	73166	NC_000085.6	Tm7sf2	1.61E+00
gene2310	1E+08	NC_000067.6	Mndal	1.61E+00
gene8147	18033	NC_000069.6	Nfkb1	1.61E+00
gene17376	74352	NC_000073.6	Zfp84	1.60E+00
gene12698	231646	NC_000071.6	Myo1h	1.60E+00
gene19638	207352	NC_000073.6	Sec23ip	1.60E+00
gene18463	1E+08	NC_000073.6	Gm17257	1.60E+00
gene15131	1E+08	NC_000072.6	Gm34170	1.60E+00
gene9282	230279	NC_000070.6	6330416G13Rik	1.60E+00
gene37286	1E+08	NC_000081.6	Gm21178	1.60E+00
gene39658	16992	NC_000083.6	Lta	1.60E+00
gene27131	66628	NC_000077.6	Thg1l	1.60E+00
gene25524	216134	NC_000076.6	Pdxk	1.60E+00
gene5413	228769	NC_000068.7	Psmf1	1.60E+00
gene2225	68874	NC_000067.6	Klhdc9	1.60E+00
gene29244	67622	NC_000077.6	Mxra7	1.60E+00
gene35484	76273	NC_000080.6	Ndfip2	1.60E+00
gene13144	100609	NC_000071.6	Nsun5	1.59E+00
gene36642	11564	NC_000081.6	Adsl	1.59E+00
gene23818	22038	NC_000075.6	Plscr1	1.59E+00
gene21512	102122	NC_000074.6	Fam192a	1.59E+00
gene8816	1E+08	NC_000070.6	Gm12397	1.59E+00
gene3303	320973	NC_000068.7	D330023K18Rik	1.59E+00
gene27866	18806	NC_000077.6	Pld2	1.59E+00
gene42896	73894	NC_000085.6	4930414N06Rik	1.59E+00
gene41896	72056	NC_000085.6	1810055G02Rik	1.59E+00
gene12586	403178	NC_000071.6	Plcxd1	1.59E+00
gene8496	59021	NC_000070.6	Rab2a	1.59E+00
gene19715	109359	NC_000073.6	Fam175b	1.59E+00
gene32712	238673	NC_000079.6	Zfp367	1.59E+00
gene42330	207521	NC_000085.6	Dtx4	1.59E+00
gene20397	ene=LOC1	NC_000074.6	LOC102631749	1.59E+00
gene11009	76580	NC_000070.6	Mib2	1.59E+00
gene7809	109674	NC_000069.6	Ampd2	1.59E+00
gene2217	19044	NC_000067.6	Ppox	1.59E+00
gene3201	140858	NC_000068.7	Wdr5	1.59E+00
gene8906	56703	NC_000070.6	Pigo	1.59E+00
gene36742	23970	NC_000081.6	Pacsin2	1.59E+00
gene17475	15451	NC_000073.6	Hpn	1.59E+00
gene16235	664817	NC_000073.6	Gm7353	1.59E+00
gene8927	230101	NC_000070.6	Gba2	1.59E+00
gene7270	26568	NC_000069.6	Slc27a3	1.59E+00
gene28318	448850	NC_000077.6	Znhit3	1.59E+00
gene21145	1E+08	NC_000074.6	Gm2059	1.59E+00
gene7138	94315	NC_000069.6	Prcc	1.59E+00
gene5482	140484	NC_000068.7	Pofut1	1.59E+00
gene1586	320718	NC_000067.6	Slc26a9	1.58E+00

gene390	226982	NC_000067.6	Eif5b	1.58E+00
gene7832	99730	NC_000069.6	Taf13	1.58E+00
gene25630	1E+08	NC_000076.6	Gm31057	1.58E+00
gene25216	71361	NC_000076.6	Aifm2	1.58E+00
gene32950	72948	NC_000079.6	Tppp	1.58E+00
gene9411	101739	NC_000070.6	Psip1	1.58E+00
gene23493	235459	NC_000075.6	Gtf2a2	1.58E+00
gene7727	12340	NC_000069.6	Capza1	1.58E+00
gene22335	70984	NC_000075.6	4931406C07Rik	1.58E+00
gene28504	18604	NC_000077.6	Pdk2	1.58E+00
gene36202	69694	NC_000081.6	Tatdn1	1.58E+00
gene38143	11658	NC_000082.6	Alcam	1.58E+00
gene15020	17768	NC_000072.6	Mthfd2	1.58E+00
gene3214	665412	NC_000068.7	Rpsa-ps9	1.58E+00
gene28787	1E+08	NC_000077.6	Gm38483	1.58E+00
gene21829	74568	NC_000074.6	Mlkl	1.58E+00
gene40915	106957	NC_000084.6	Slc39a6	1.58E+00
gene32437	268656	NC_000079.6	Sptlc1	1.58E+00
gene37851	66994	NC_000082.6	Cep19	1.57E+00
gene17982	211548	NC_000073.6	Nomo1	1.57E+00
gene20200	99375	NC_000074.6	Cul4a	1.57E+00
gene39278	26378	NC_000083.6	Decr2	1.57E+00
gene33419	73274	NC_000079.6	Gpbp1	1.57E+00
gene465	1.1E+08	NC_000067.6	Gm28782	1.57E+00
gene39951	224807	NC_000083.6	Tmem63b	1.57E+00
gene33856	19056	NC_000080.6	Ppp3cb	1.57E+00
gene27586	11670	NC_000077.6	Aldh3a1	1.57E+00
gene14493	110175	NC_000072.6	Ggct	1.57E+00
gene17166	232975	NC_000073.6	Atp1a3	1.57E+00
gene36443	1E+08	NC_000081.6	Smpd5	1.57E+00
gene27720	215723	NC_000077.6	Mfsd6l	1.57E+00
gene20805	1E+08	NC_000074.6	Gm33153	1.57E+00
gene3664	227929	NC_000068.7	Cytip	1.57E+00
gene23230	110119	NC_000075.6	Mpi	1.57E+00
gene30498	1.1E+08	NC_000078.6	Gm40476	1.57E+00
gene2212	11807	NC_000067.6	Apoa2	1.57E+00
gene3732	667291	NC_000068.7	Gm13578	1.57E+00
gene2407	19165	NC_000067.6	Psen2	1.57E+00
gene36212	223593	NC_000081.6	E430025E21Rik	1.57E+00
gene19300	67420	NC_000073.6	Far1	1.57E+00
gene24430	20623	NC_000075.6	Snrk	1.56E+00
gene5089	319513	NC_000068.7	Pced1a	1.56E+00
gene5779	329559	NC_000068.7	Zfp335	1.56E+00
gene22573	330907	NC_000075.6	Gm5120	1.56E+00
gene26434	21853	NC_000076.6	Timeless	1.56E+00
gene37660	1E+08	NC_000082.6	Gm4462	1.56E+00
gene10242	97130	NC_000070.6	C77080	1.56E+00
gene10871	69151	NC_000070.6	Lzic	1.56E+00

gene23933	83703	NC_000075.6	Dbr1	1.56E+00
gene25809	103136	NC_000076.6	Pwp1	1.56E+00
gene41211	14815	NC_000084.6	Nr3c1	1.56E+00
gene40639	668871	NC_000083.6	Gm9410	1.56E+00
gene42585	27356	NC_000085.6	Insl6	1.56E+00
gene37468	94184	NC_000082.6	Pdxdc1	1.56E+00
gene25308	170799	NC_000076.6	Rtkn2	1.56E+00
gene39776	386454	NC_000083.6	Rnf39	1.56E+00
gene24110	84585	NC_000075.6	Rnf123	1.56E+00
gene10003	230696	NC_000070.6	AU022252	1.56E+00
gene41507	17714	NC_000084.6	Grpel2	1.56E+00
gene20428	20377	NC_000074.6	Sfrp1	1.56E+00
gene14652	14701	NC_000072.6	Gng12	1.56E+00
gene31069	328162	NC_000078.6	Trmt61a	1.56E+00
gene8970	76238	NC_000070.6	Grhpr	1.55E+00
gene19801	14912	NC_000073.6	Nkx6-2	1.55E+00
gene26358	66399	NC_000076.6	Tsfm	1.55E+00
gene30042	11855	NC_000078.6	Arhgap5	1.55E+00
gene34295	50527	NC_000080.6	Ero1l	1.55E+00
gene16353	19428	NC_000073.6	Rasl2-9	1.55E+00
gene27839	69309	NC_000077.6	Slc16a13	1.55E+00
gene33842	67509	NC_000080.6	Saysd1	1.55E+00
gene15714	14789	NC_000072.6	P3h3	1.55E+00
gene30877	12062	NC_000078.6	Bdkrb2	1.55E+00
gene28593	57765	NC_000077.6	Tbx21	1.55E+00
gene5970	66679	NC_000068.7	Rae1	1.55E+00
gene43061	12369	NC_000085.6	Casp7	1.55E+00
gene15687	19305	NC_000072.6	Pex5	1.55E+00
gene13175	269717	NC_000071.6	Orai2	1.55E+00
gene40749	59025	NC_000084.6	Usp14	1.55E+00
gene5294	67877	NC_000068.7	Naa20	1.54E+00
gene40232	13733	NC_000083.6	Adgre1	1.54E+00
gene939	71728	NC_000067.6	Stk11ip	1.54E+00
gene22704	64011	NC_000075.6	Nrgn	1.54E+00
gene21828	ene=LOC1	NC_000074.6	LOC105243289	1.54E+00
gene31456	56349	NC_000079.6	Net1	1.54E+00
gene38756	667114	NC_000083.6	Gm8465	1.54E+00
gene32713	56541	NC_000079.6	Habp4	1.54E+00
gene22471	215194	NC_000075.6	Kri1	1.54E+00
gene14911	56316	NC_000072.6	Ggcx	1.54E+00
gene23288	546134	NC_000075.6	Gramd2	1.54E+00
gene25516	28169	NC_000076.6	Agpat3	1.54E+00
gene10731	20148	NC_000070.6	Dhrs3	1.54E+00
gene12909	56317	NC_000071.6	Anapc7	1.54E+00
gene10859	13347	NC_000070.6	Dffa	1.54E+00
gene30144	70611	NC_000078.6	Fbxo33	1.54E+00
gene16651	243834	NC_000073.6	Zfp324	1.54E+00
gene30510	72873	NC_000078.6	Bbof1	1.54E+00

gene38339	30940	NC_000082.6	Usp25	1.54E+00
gene36627	239555	NC_000081.6	Mief1	1.54E+00
gene8908	230088	NC_000070.6	Fam214b	1.54E+00
gene29114	28081	NC_000077.6	Fam104a	1.54E+00
gene9965	66451	NC_000070.6	2610528J11Rik	1.54E+00
gene36511	328561	NC_000081.6	Apol10b	1.54E+00
gene12214	56041	NC_000071.6	Uso1	1.54E+00
gene18671	74737	NC_000073.6	Pcf11	1.54E+00
gene21557	66083	NC_000074.6	Setd6	1.54E+00
gene85	211673	NC_000067.6	Arfgef1	1.54E+00
gene30582	382620	NC_000078.6	Tmed8	1.54E+00
gene11027	433813	NC_000070.6	Pusl1	1.54E+00
gene42486	226016	NC_000085.6	Abhd17b	1.54E+00
gene20492	72316	NC_000074.6	2310008N11Rik	1.54E+00
gene25187	74048	NC_000076.6	Vsir	1.54E+00
gene8902	56323	NC_000070.6	Dnajb5	1.53E+00
gene24618	637043	NC_000076.6	Gm7198	1.53E+00
gene28197	52615	NC_000077.6	Suz12	1.53E+00
gene7193	74200	NC_000069.6	2810403A07Rik	1.53E+00
gene4427	51897	NC_000068.7	Atg13	1.53E+00
gene2654	54139	NC_000067.6	Irf6	1.53E+00
gene20864	72612	NC_000074.6	Hpf1	1.53E+00
gene5151	70612	NC_000068.7	Tmem230	1.53E+00
gene35902	223455	NC_000081.6	March6	1.53E+00
gene21811	102339	NC_000074.6	Cog4	1.53E+00
gene16781	330474	NC_000073.6	Zc3h4	1.53E+00
gene14328	68910	NC_000072.6	Zfp467	1.53E+00
gene3936	14231	NC_000068.7	Fkbp7	1.53E+00
gene23319	21887	NC_000075.6	Tle3	1.53E+00
gene2380	=Gene;ger	NC_000067.6	LOC108167748	1.53E+00
gene21558	234594	NC_000074.6	Cnot1	1.53E+00
gene10450	53902	NC_000070.6	Rcan3	1.53E+00
gene7901	229782	NC_000069.6	Slc35a3	1.53E+00
gene4743	228491	NC_000068.7	Zfp770	1.53E+00
gene28678	192160	NC_000077.6	Casc3	1.53E+00
gene35933	19946	NC_000081.6	Rpl30	1.53E+00
gene18160	330544	NC_000073.6	C230091D08Rik	1.52E+00
gene21565	14719	NC_000074.6	Got2	1.52E+00
gene31123	16450	NC_000078.6	Jag2	1.52E+00
gene15732	1E+08	NC_000072.6	Gm32616	1.52E+00
gene22473	11768	NC_000075.6	Ap1m2	1.52E+00
gene42190	70044	NC_000085.6	Tut1	1.52E+00
gene21784	11765	NC_000074.6	Ap1g1	1.52E+00
gene6773	97064	NC_000069.6	Wwtr1	1.52E+00
gene340	50785	NC_000067.6	Hs6st1	1.52E+00
gene2106	27878	NC_000067.6	Tada1	1.52E+00
gene19480	67711	NC_000073.6	Nsmce1	1.52E+00
gene29142	217304	NC_000077.6	Cd300lb	1.52E+00

gene43145	67894	NC_000085.6	Fam45a	1.52E+00
gene19262	114332	NC_000073.6	Lyve1	1.52E+00
gene19288	78748	NC_000073.6	Rassf10	1.52E+00
gene38408	74112	NC_000082.6	Usp16	1.52E+00
gene35990	670911	NC_000081.6	Gm9509	1.52E+00
gene40194	71810	NC_000083.6	Ranbp3	1.52E+00
gene20688	78849	NC_000074.6	B430010I23Rik	1.52E+00
gene37374	252870	NC_000082.6	Usp7	1.51E+00
gene21004	67937	NC_000074.6	Tmem59l	1.51E+00
gene41404	56698	NC_000084.6	Phax	1.51E+00
gene9448	20104	NC_000070.6	Rps6	1.51E+00
gene17719	1E+08	NC_000073.6	Gm15470	1.51E+00
gene6812	140795	NC_000069.6	P2ry14	1.51E+00
gene10984	1.1E+08	NC_000070.6	Gm42362	1.51E+00
gene13247	545812	NC_000071.6	Pilrb2	1.51E+00
gene31873	218100	NC_000079.6	Zfp322a	1.51E+00
gene19458	19647	NC_000073.6	Rbbp6	1.51E+00
gene32108	73710	NC_000079.6	Tubb2b	1.51E+00
gene10078	16918	NC_000070.6	Mycl	1.51E+00
gene6489	18706	NC_000069.6	Pik3ca	1.51E+00
gene18714	12729	NC_000073.6	Clns1a	1.51E+00
gene28343	74038	NC_000077.6	Brip1os	1.51E+00
gene6090	329575	NC_000068.7	Gm14325	1.51E+00
gene9458	66775	NC_000070.6	Hacd4	1.51E+00
gene920	52231	NC_000067.6	Ankzf1	1.51E+00
gene29586	105005	NC_000078.6	Fam84a	1.51E+00
gene33480	14313	NC_000079.6	Fst	1.51E+00
gene3350	50935	NC_000068.7	St6galnac6	1.51E+00
gene35300	219170	NC_000080.6	Fam216b	1.51E+00
gene42780	12495	NC_000085.6	Entpd1	1.51E+00
gene29185	67283	NC_000077.6	Slc25a19	1.51E+00
gene11441	231069	NC_000071.6	Gm4865	1.51E+00
gene7262	68328	NC_000069.6	Rab13	1.51E+00
gene30814	20701	NC_000078.6	Serpina1b	1.51E+00
gene21055	17925	NC_000074.6	Myo9b	1.51E+00
gene20119	13642	NC_000074.6	Efnb2	1.51E+00
gene3542	1E+08	NC_000068.7	Gm13462	1.51E+00
gene6781	24017	NC_000069.6	Rnf13	1.51E+00
gene30099	1E+08	NC_000078.6	Gm19990	1.50E+00
gene33125	1.1E+08	NC_000079.6	Gm41018	1.50E+00
gene467	98404	NC_000067.6	AI597479	1.50E+00
gene39398	74116	NC_000083.6	Pi16	1.50E+00
gene39179	22084	NC_000083.6	Tsc2	1.50E+00
gene20624	71908	NC_000074.6	Cldn23	1.50E+00
gene37266	12419	NC_000081.6	Cbx5	1.50E+00
gene25701	14676	NC_000076.6	Gna15	1.50E+00
gene42628	17330	NC_000085.6	Minpp1	1.50E+00
gene19593	233904	NC_000073.6	Setd1a	1.50E+00

gene32506	67399	NC_000079.6	Pdlim7	1.50E+00
gene15873	16643	NC_000072.6	Klrd1	1.50E+00
gene6259	21985	NC_000069.6	Tpd52	1.50E+00
gene7014	66841	NC_000069.6	Etfdh	1.50E+00
gene13132	69812	NC_000071.6	Abhd11os	1.50E+00
gene12892	231717	NC_000071.6	Fam109a	1.50E+00
gene26726	319939	NC_000077.6	Tns3	1.50E+00
gene16297	18729	NC_000073.6	Pira6	1.50E+00
gene17653	66161	NC_000073.6	Pop4	1.50E+00
gene17258	1E+08	NC_000073.6	Gm30543	1.49E+00
gene32417	20354	NC_000079.6	Sema4d	1.49E+00
gene25393	11540	NC_000076.6	Adora2a	1.49E+00
gene34106	11752	NC_000080.6	Anxa8	1.49E+00
gene16315	66245	NC_000073.6	Hspbp1	1.49E+00
gene18395	434200	NC_000073.6	Gm5597	1.49E+00
gene27491	94091	NC_000077.6	Trim11	1.49E+00
gene17351	26411	NC_000073.6	Map4k1	1.49E+00
gene43037	14732	NC_000085.6	Gpam	1.49E+00
gene38610	16516	NC_000082.6	Kcnj15	1.49E+00
gene1707	66241	NC_000067.6	Tmem9	1.49E+00
gene7204	110196	NC_000069.6	Fdps	1.49E+00
gene10402	1E+08	NC_000070.6	Gm12977	1.49E+00
gene20578	234155	NC_000074.6	Mboat4	1.49E+00
gene30393	110417	NC_000078.6	Pigh	1.49E+00
gene5207	59030	NC_000068.7	Mkks	1.49E+00
gene364	22637	NC_000067.6	Zap70	1.49E+00
gene10333	66464	NC_000070.6	Taf12	1.49E+00
gene20661	20288	NC_000074.6	Msr1	1.48E+00
gene39738	15043	NC_000083.6	H2-T3	1.48E+00
gene262	624138	NC_000067.6	Gm6473	1.48E+00
gene8596	67460	NC_000070.6	Decr1	1.48E+00
gene38505	66578	NC_000082.6	Mis18a	1.48E+00
gene38382	27393	NC_000082.6	Mrpl39	1.48E+00
gene10485	269593	NC_000070.6	Luzp1	1.48E+00
gene4541	80985	NC_000068.7	Trim44	1.48E+00
gene35329	67955	NC_000080.6	Sugt1	1.48E+00
gene22660	67049	NC_000075.6	Pus3	1.48E+00
gene13209	70240	NC_000071.6	Ufsp1	1.48E+00
gene14516	78937	NC_000072.6	Avl9	1.48E+00
gene38895	13388	NC_000083.6	Dll1	1.48E+00
gene1637	20643	NC_000067.6	Snrpe	1.48E+00
gene8258	23908	NC_000069.6	Hs2st1	1.48E+00
gene1050	109032	NC_000067.6	Sp110	1.48E+00
gene27770	80515	NC_000077.6	Chd3os	1.47E+00
gene10340	71787	NC_000070.6	Trnau1ap	1.47E+00
gene37061	66120	NC_000081.6	Fkbp11	1.47E+00
gene22352	234967	NC_000075.6	Slc36a4	1.47E+00
gene5820	277360	NC_000068.7	Prex1	1.47E+00

gene20601	319520	NC_000074.6	Dusp4	1.47E+00
gene28988	70207	NC_000077.6	Taco1	1.47E+00
gene26839	216549	NC_000077.6	Aftph	1.47E+00
gene40186	224902	NC_000083.6	Safb2	1.47E+00
gene14171	109218	NC_000072.6	Tmem139	1.47E+00
gene25953	216233	NC_000076.6	Socs2	1.47E+00
gene29613	19878	NC_000078.6	Rock2	1.47E+00
gene26876	1E+08	NC_000077.6	Gm12057	1.47E+00
gene28299	78394	NC_000077.6	Ddx52	1.47E+00
gene19240	233724	NC_000073.6	Tmem41b	1.47E+00
gene27875	13808	NC_000077.6	Eno3	1.47E+00
gene19500	70314	NC_000073.6	Rabep2	1.47E+00
gene30609	1E+08	NC_000078.6	Gm21559	1.47E+00
gene30104	104798	NC_000078.6	E030019B13Rik	1.47E+00
gene26297	56480	NC_000076.6	Tbk1	1.47E+00
gene40478	ene;gene=L	NC_000083.6	LOC102635879	1.47E+00
gene32406	20729	NC_000079.6	Spin1	1.47E+00
gene12795	72151	NC_000071.6	Rfc5	1.47E+00
gene36459	13350	NC_000081.6	Dgat1	1.47E+00
gene11535	64339	NC_000071.6	Fndc4	1.47E+00
gene22558	80517	NC_000075.6	Herpud2	1.47E+00
gene36866	29857	NC_000081.6	Mapk12	1.47E+00
gene4810	228536	NC_000068.7	Bahd1	1.47E+00
gene40218	70785	NC_000083.6	Dennd1c	1.47E+00
gene26196	432508	NC_000076.6	Cpsf6	1.47E+00
gene37854	268882	NC_000082.6	Fbxo45	1.47E+00
gene28073	109934	NC_000077.6	Abr	1.47E+00
gene38831	60532	NC_000083.6	Wtap	1.46E+00
gene9999	195522	NC_000070.6	Zfp691	1.46E+00
gene11633	74364	NC_000071.6	4931431C16Rik	1.46E+00
gene40609	17688	NC_000083.6	Msh6	1.46E+00
gene40984	13872	NC_000084.6	Ercc3	1.46E+00
gene18771	1E+08	NC_000073.6	Tpbgl	1.46E+00
gene30717	75553	NC_000078.6	Zc3h14	1.46E+00
gene31100	70435	NC_000078.6	Inf2	1.46E+00
gene30285	104771	NC_000078.6	Jkamp	1.46E+00
gene13396	19159	NC_000071.6	Cyth3	1.46E+00
gene25248	73132	NC_000076.6	Slc25a16	1.46E+00
gene29353	71885	NC_000077.6	Faap100	1.46E+00
gene6130	1E+08	NC_000068.7	Gm29886	1.46E+00
gene12902	320717	NC_000071.6	Pptc7	1.46E+00
gene41851	545269	NC_000084.6	Gm5824	1.46E+00
gene12922	80751	NC_000071.6	Rnf34	1.46E+00
gene32517	212880	NC_000079.6	Ddx46	1.46E+00
gene29410	67608	NC_000077.6	Narf	1.46E+00
gene16268	232791	NC_000073.6	Cnot3	1.46E+00
gene28105	68564	NC_000077.6	Nufip2	1.46E+00
gene11293	19248	NC_000071.6	Ptpn12	1.46E+00

gene36303	633752	NC_000081.6	Gm7125	1.46E+00
gene37637	239743	NC_000082.6	Klhl6	1.46E+00
gene6510	14359	NC_000069.6	Fxr1	1.46E+00
gene37015	56233	NC_000081.6	Hdac7	1.46E+00
gene22431	77519	NC_000075.6	Zfp266	1.46E+00
gene33941	71918	NC_000080.6	Zcchc24	1.46E+00
gene22491	20586	NC_000075.6	Smarca4	1.45E+00
gene15069	232187	NC_000072.6	Smyd5	1.45E+00
gene30574	217732	NC_000078.6	Cipc	1.45E+00
gene12461	100604	NC_000071.6	Lrrc8c	1.45E+00
gene2655	319266	NC_000067.6	A130010J15Rik	1.45E+00
gene24546	18537	NC_000076.6	Pcmt1	1.45E+00
gene34049	24056	NC_000080.6	Sh3bp5	1.45E+00
gene19904	71448	NC_000073.6	Tmem80	1.45E+00
gene33788	71147	NC_000080.6	Oxsm	1.45E+00
gene40024	56771	NC_000083.6	Med20	1.45E+00
gene8678	242362	NC_000070.6	Manea	1.45E+00
gene28842	217207	NC_000077.6	Dhx8	1.45E+00
gene15399	58911	NC_000072.6	Sumf1	1.45E+00
gene342	320011	NC_000067.6	Uggt1	1.45E+00
gene21225	74841	NC_000074.6	Usp38	1.45E+00
gene14046	75379	NC_000072.6	4930599N23Rik	1.45E+00
gene14473	66873	NC_000072.6	Tril	1.45E+00
gene30768	23876	NC_000078.6	Fbln5	1.45E+00
gene2624	18005	NC_000067.6	Nek2	1.45E+00
gene31438	1E+08	NC_000079.6	Gm35043	1.45E+00
gene19866	338417	NC_000073.6	Scgb1c1	1.45E+00
gene36620	1E+08	NC_000081.6	Gm31134	1.45E+00
gene20412	244349	NC_000074.6	Kat6a	1.45E+00
gene19674	56213	NC_000073.6	Htra1	1.45E+00
gene15868	108078	NC_000072.6	Olr1	1.45E+00
gene12866	23960	NC_000071.6	Oas1g	1.45E+00
gene37665	245841	NC_000082.6	Polr2h	1.45E+00
gene19630	21843	NC_000073.6	Tial1	1.45E+00
gene28202	71956	NC_000077.6	Rnf135	1.44E+00
gene9251	338355	NC_000070.6	Fkbp15	1.44E+00
gene13601	69263	NC_000071.6	Rfc3	1.44E+00
gene33101	66475	NC_000079.6	Rps23	1.44E+00
gene12950	77573	NC_000071.6	Vps33a	1.44E+00
gene22973	140630	NC_000075.6	Ube4a	1.44E+00
gene20677	436137	NC_000074.6	Gm5749	1.44E+00
gene42499	16601	NC_000085.6	Klf9	1.44E+00
gene14546	66459	NC_000072.6	Pyurf	1.44E+00
gene17858	18971	NC_000073.6	Pold1	1.44E+00
gene22042	234847	NC_000074.6	Spg7	1.44E+00
gene42816	66583	NC_000085.6	Exosc1	1.44E+00
gene20120	234023	NC_000074.6	Arglu1	1.44E+00
gene39303	77128	NC_000083.6	Crebrf	1.44E+00

gene30293	209183	NC_000078.6	Gm4756	1.44E+00
gene28810	217198	NC_000077.6	Plekhh3	1.44E+00
gene36750	223723	NC_000081.6	Ttll12	1.44E+00
gene15706	16977	NC_000072.6	Lrrc23	1.44E+00
gene40021	12445	NC_000083.6	Ccnd3	1.44E+00
gene27245	17001	NC_000077.6	Ltc4s	1.44E+00
gene2942	75869	NC_000068.7	Arl5b	1.44E+00
gene988	26554	NC_000067.6	Cul3	1.44E+00
gene20409	18791	NC_000074.6	Plat	1.44E+00
gene39355	23853	NC_000083.6	Def6	1.44E+00
gene42217	225908	NC_000085.6	Myrf	1.44E+00
gene36879	1E+08	NC_000081.6	Sco2	1.44E+00
gene13984	101240	NC_000072.6	Wdr91	1.43E+00
gene2657	15483	NC_000067.6	Hsd11b1	1.43E+00
gene28081	53334	NC_000077.6	Gosr1	1.43E+00
gene33529	72465	NC_000079.6	Zfp131	1.43E+00
gene22997	23821	NC_000075.6	Bace1	1.43E+00
gene28514	217127	NC_000077.6	Kat7	1.43E+00
gene20618	1E+08	NC_000074.6	Gm16793	1.43E+00
gene4875	1E+08	NC_000068.7	AV039307	1.43E+00
gene6458	80732	NC_000069.6	Mynn	1.43E+00
gene39359	19896	NC_000083.6	Rpl10a	1.43E+00
gene24198	104831	NC_000075.6	Ptpn23	1.43E+00
gene3094	12496	NC_000068.7	Entpd2	1.43E+00
gene19935	18218	NC_000073.6	Dusp8	1.43E+00
gene19979	97423	NC_000073.6	R74862	1.43E+00
gene40846	664619	NC_000084.6	4921533I20Rik	1.43E+00
gene42919	226162	NC_000085.6	Dpcd	1.43E+00
gene16779	170770	NC_000073.6	Bbc3	1.43E+00
gene27829	13543	NC_000077.6	Dvl2	1.43E+00
gene18650	13626	NC_000073.6	Eed	1.43E+00
gene41670	17128	NC_000084.6	Smad4	1.43E+00
gene27887	19069	NC_000077.6	Nup88	1.43E+00
gene22662	627480	NC_000075.6	Gm6762	1.43E+00
gene25653	73218	NC_000076.6	Sppl2b	1.43E+00
gene12228	1.1E+08	NC_000071.6	Gm42114	1.43E+00
gene20410	64933	NC_000074.6	Ap3m2	1.43E+00
gene7815	229715	NC_000069.6	Amigo1	1.43E+00
gene7111	12480	NC_000069.6	Cd1d2	1.43E+00
gene25920	16993	NC_000076.6	Lta4h	1.43E+00
gene22123	102162	NC_000074.6	Taf5l	1.43E+00
gene8999	433702	NC_000070.6	Ncbp1	1.42E+00
gene10828	17769	NC_000070.6	Mthfr	1.42E+00
gene20154	19332	NC_000074.6	Rab20	1.42E+00
gene37509	70120	NC_000082.6	Yars2	1.42E+00
gene31075	68520	NC_000078.6	Zfyve21	1.42E+00
gene16031	=Gene;ger	NC_000072.6	LOC108169174	1.42E+00
gene37460	67118	NC_000082.6	Bfar	1.42E+00

gene7707	67489	NC_000069.6	Ap4b1	1.42E+00
gene43080	226252	NC_000085.6	Fam160b1	1.42E+00
gene27311	21414	NC_000077.6	Tcf7	1.42E+00
gene15447	18294	NC_000072.6	Ogg1	1.42E+00
gene11505	231093	NC_000071.6	Agbl5	1.42E+00
gene23279	23806	NC_000075.6	Arih1	1.42E+00
gene4989	109778	NC_000068.7	Blvra	1.42E+00
gene12307	231474	NC_000071.6	Paqr3	1.42E+00
gene36239	67552	NC_000081.6	H2afy3	1.42E+00
gene31559	18073	NC_000079.6	Nid1	1.42E+00
gene5312	24128	NC_000068.7	Xrn2	1.42E+00
gene3081	97031	NC_000068.7	Tprn	1.42E+00
gene9688	67916	NC_000070.6	Plpp3	1.42E+00
gene32838	170763	NC_000079.6	Zfp87	1.42E+00
gene22646	78934	NC_000075.6	4930581F22Rik	1.42E+00
gene42291	225929	NC_000085.6	Patl1	1.42E+00
gene23115	14148	NC_000075.6	Fdx1	1.42E+00
gene25600	1E+08	NC_000076.6	Cbarp	1.42E+00
gene39477	57757	NC_000083.6	Pglyrp2	1.42E+00
gene29215	69535	NC_000077.6	Ten1	1.41E+00
gene11509	16548	NC_000071.6	Khk	1.41E+00
gene15250	72170	NC_000072.6	Chchd4	1.41E+00
gene18080	53415	NC_000073.6	Htatip2	1.41E+00
gene13968	14187	NC_000072.6	Akr1b8	1.41E+00
gene14990	15277	NC_000072.6	Hk2	1.41E+00
gene26448	67588	NC_000076.6	Rnf41	1.41E+00
gene2277	68440	NC_000067.6	Dusp23	1.41E+00
gene16346	68992	NC_000073.6	Zfp580	1.41E+00
gene12854	100764	NC_000071.6	Rita1	1.41E+00
gene21241	71310	NC_000074.6	Tbc1d9	1.41E+00
gene41339	15488	NC_000084.6	Hsd17b4	1.41E+00
gene16112	16523	NC_000072.6	Kcnj8	1.41E+00
gene37524	21976	NC_000082.6	Top3b	1.41E+00
gene28085	104184	NC_000077.6	Blmh	1.41E+00
gene28443	69802	NC_000077.6	Cox11	1.41E+00
gene33655	1E+08	NC_000080.6	Gm17048	1.41E+00
gene26205	70574	NC_000076.6	Cpm	1.41E+00
gene43072	213993	NC_000085.6	Ccdc186	1.41E+00
gene19275	74996	NC_000073.6	Usp47	1.41E+00
gene18405	67994	NC_000073.6	Mrps11	1.41E+00
gene3343	227731	NC_000068.7	Slc25a25	1.41E+00
gene31973	66834	NC_000079.6	Acot13	1.41E+00
gene22985	214523	NC_000075.6	Tmprss4	1.41E+00
gene26895	72129	NC_000077.6	Pex13	1.41E+00
gene30759	68734	NC_000078.6	Smek1	1.41E+00
gene432	16174	NC_000067.6	Il18rap	1.41E+00
gene28800	11975	NC_000077.6	Atp6v0a1	1.41E+00
gene18418	54608	NC_000073.6	Abhd2	1.41E+00

gene17790	83382	NC_000073.6	Siglece	1.40E+00
gene12952	70650	NC_000071.6	Zcchc8	1.40E+00
gene17432	75660	NC_000073.6	Lin37	1.40E+00
gene19246	20947	NC_000073.6	Swap70	1.40E+00
gene21564	234595	NC_000074.6	Slc38a7	1.40E+00
gene23412	50996	NC_000075.6	Pdcd7	1.40E+00
gene28198	54394	NC_000077.6	Crlf3	1.40E+00
gene11336	69188	NC_000071.6	Kmt2e	1.40E+00
gene22516	12797	NC_000075.6	Cnn1	1.40E+00
gene5652	228852	NC_000068.7	Ppp1r16b	1.40E+00
gene10620	13836	NC_000070.6	Epha2	1.40E+00
gene3218	70239	NC_000068.7	Gtf3c5	1.40E+00
gene17770	69930	NC_000073.6	Zfp715	1.40E+00
gene3249	74412	NC_000068.7	Gle1	1.40E+00
gene22435	30843	NC_000075.6	Fbxl12	1.40E+00
gene20130	319583	NC_000074.6	Lig4	1.40E+00
gene24585	1E+08	NC_000076.6	Gm9797	1.40E+00
gene2682	12946	NC_000067.6	Cr1l	1.40E+00
gene891	58184	NC_000067.6	Cnot9	1.40E+00
gene15754	1E+08	NC_000072.6	Gm32673	1.40E+00
gene16306	21954	NC_000073.6	Tnni3	1.40E+00
gene12703	17855	NC_000071.6	Mvk	1.40E+00
gene40900	22694	NC_000084.6	Zfp35	1.40E+00
gene42106	107328	NC_000085.6	Trpt1	1.40E+00
gene6504	67120	NC_000069.6	Ttc14	1.40E+00
gene5069	654409	NC_000068.7	4932416H05Rik	1.40E+00
gene28671	23989	NC_000077.6	Med24	1.40E+00
gene40393	668709	NC_000083.6	Gm9311	1.40E+00
gene36272	76740	NC_000081.6	Efr3a	1.40E+00
gene1824	226499	NC_000067.6	BC003331	1.40E+00
gene24274	75116	NC_000075.6	4930520O04Rik	1.40E+00
gene4842	69702	NC_000068.7	Ndufaf1	1.40E+00
gene30029	207304	NC_000078.6	Hectd1	1.40E+00
gene19898	101471	NC_000073.6	Phrf1	1.40E+00
gene24446	235682	NC_000075.6	Zfp445	1.39E+00
gene12533	243197	NC_000071.6	Mfsd7a	1.39E+00
gene38354	67102	NC_000082.6	D16Ertd472e	1.39E+00
gene15264	78287	NC_000072.6	Rbsn	1.39E+00
gene21516	67801	NC_000074.6	Pllp	1.39E+00
gene8311	18749	NC_000069.6	Prkacb	1.39E+00
gene11016	246228	NC_000070.6	Vwa1	1.39E+00
gene8564	=Gene;ger	NC_000070.6	LOC108168930	1.39E+00
gene4396	68349	NC_000068.7	Ndufs3	1.39E+00
gene19499	12478	NC_000073.6	Cd19	1.39E+00
gene27911	109212	NC_000077.6	Fam64a	1.39E+00
gene28216	=Gene;ger	NC_000077.6	LOC108167346	1.39E+00
gene31600	56044	NC_000079.6	Rala	1.39E+00
gene42512	381217	NC_000085.6	Fam189a2	1.39E+00

gene8409	624519	NC_000069.6	Gm6510	1.39E+00
gene27427	73158	NC_000077.6	Larp1	1.39E+00
gene19655	14183	NC_000073.6	Fgfr2	1.39E+00
gene33848	70601	NC_000080.6	Ecd	1.39E+00
gene24197	235623	NC_000075.6	Scap	1.39E+00
gene680	55989	NC_000067.6	Nop58	1.39E+00
gene13032	100561	NC_000071.6	Slc15a4	1.39E+00
gene29246	74319	NC_000077.6	Mettl23	1.39E+00
gene6796	229317	NC_000069.6	Eif2a	1.39E+00
gene4596	98221	NC_000068.7	Eif3m	1.39E+00
gene29354	217365	NC_000077.6	Nploc4	1.39E+00
gene11368	213990	NC_000071.6	Agap3	1.39E+00
gene25793	103266	NC_000076.6	Tmem263	1.38E+00
gene22055	234854	NC_000074.6	Cdk10	1.38E+00
gene32379	109082	NC_000079.6	Fbxw17	1.38E+00
gene20408	16150	NC_000074.6	Ikbkb	1.38E+00
gene1274	66262	NC_000067.6	Ing5	1.38E+00
gene12963	330192	NC_000071.6	Vps37b	1.38E+00
gene39401	26382	NC_000083.6	Fgd2	1.38E+00
gene37479	67203	NC_000082.6	Nde1	1.38E+00
gene1581	667238	NC_000067.6	Gm8532	1.38E+00
gene11362	74610	NC_000071.6	Abcb8	1.38E+00
gene41407	16906	NC_000084.6	Lmnb1	1.38E+00
gene21713	66432	NC_000074.6	Slc7a6os	1.38E+00
gene20769	69260	NC_000074.6	Ing2	1.38E+00
gene25362	69718	NC_000076.6	Ipmk	1.38E+00
gene28600	67036	NC_000077.6	Mrpl45	1.38E+00
gene37300	54483	NC_000082.6	Mefv	1.38E+00
gene13145	107939	NC_000071.6	Pom121	1.38E+00
gene8302	14707	NC_000069.6	Gng5	1.38E+00
gene15440	14910	NC_000072.6	Gt(ROSA)26Sor	1.38E+00
gene25695	1E+08	NC_000076.6	Gm16104	1.38E+00
gene26654	279766	NC_000077.6	Rhbdd3	1.38E+00
gene39322	67267	NC_000083.6	Uqcc2	1.38E+00
gene16892	13871	NC_000073.6	Ercc2	1.37E+00
gene11685	17089	NC_000071.6	Lyar	1.37E+00
gene7835	229725	NC_000069.6	Clcc1	1.37E+00
gene5123	74450	NC_000068.7	Pank2	1.37E+00
gene4534	76501	NC_000068.7	Commd9	1.37E+00
gene29195	217325	NC_000077.6	Llgl2	1.37E+00
gene476	1E+08	NC_000067.6	Gm16103	1.37E+00
gene36566	110253	NC_000081.6	Triobp	1.37E+00
gene11895	68552	NC_000071.6	Smim14	1.37E+00
gene6148	73247	NC_000068.7	Mrgbp	1.37E+00
gene26635	75452	NC_000077.6	Ascc2	1.37E+00
gene22934	270151	NC_000075.6	Nlrx1	1.37E+00
gene40158	15193	NC_000083.6	Hdgfrp2	1.37E+00
gene31515	1E+08	NC_000079.6	Gm36423	1.37E+00

gene5361	104348	NC_000068.7	Zfp120	1.37E+00
gene13315	231832	NC_000071.6	Tmem184a	1.37E+00
gene21413	93960	NC_000074.6	Nkd1	1.37E+00
gene16287	52855	NC_000073.6	Lair1	1.37E+00
gene36198	74868	NC_000081.6	Tmem65	1.37E+00
gene12305	670358	NC_000071.6	Gm9484	1.37E+00
gene23429	1E+08	NC_000075.6	Gm30314	1.37E+00
gene8299	99167	NC_000069.6	Ssx2ip	1.37E+00
gene3696	1E+08	NC_000068.7	Gm13566	1.37E+00
gene28360	67487	NC_000077.6	Dhx40	1.37E+00
gene18518	108797	NC_000073.6	Mex3b	1.37E+00
gene8834	329828	NC_000070.6	AI464131	1.37E+00
gene31102	11565	NC_000078.6	Adssl1	1.37E+00
gene13701	27053	NC_000072.6	Asns	1.37E+00
gene33220	105372	NC_000079.6	Utp15	1.36E+00
gene21932	21341	NC_000074.6	Taf1c	1.36E+00
gene23883	192287	NC_000075.6	Slc25a36	1.36E+00
gene22965	192653	NC_000075.6	Ttc36	1.36E+00
gene40450	53951	NC_000083.6	Gpatch11	1.36E+00
gene28618	67480	NC_000077.6	Cwc25	1.36E+00
gene25751	18045	NC_000076.6	Nfyb	1.36E+00
gene42979	1E+08	NC_000085.6	Gm19557	1.36E+00
gene1556	18640	NC_000067.6	Pfkfb2	1.36E+00
gene5623	18111	NC_000068.7	Nnat	1.36E+00
gene33884	319486	NC_000080.6	A430057M04Rik	1.36E+00
gene30101	217588	NC_000078.6	Mbip	1.36E+00
gene16178	67623	NC_000072.6	Tm7sf3	1.36E+00
gene42236	12507	NC_000085.6	Cd5	1.36E+00
gene42587	67759	NC_000085.6	Plgrkt	1.36E+00
gene36576	27008	NC_000081.6	Micall1	1.36E+00
gene5215	228662	NC_000068.7	Btbd3	1.36E+00
gene380	=Gene;ger	NC_000067.6	LOC102637491	1.36E+00
gene26625	71962	NC_000077.6	Gatsl3	1.36E+00
gene37703	104156	NC_000082.6	Etv5	1.36E+00
gene27113	16160	NC_000077.6	Il12b	1.36E+00
gene6717	212114	NC_000069.6	Nhlrc3	1.36E+00
gene10925	74035	NC_000070.6	Nol9	1.36E+00
gene29302	30951	NC_000077.6	Cbx8	1.36E+00
gene18809	ene=LOC1	NC_000073.6	LOC108167449	1.36E+00
gene35045	54616	NC_000080.6	Extl3	1.36E+00
gene6574	229228	NC_000069.6	Nudt6	1.36E+00
gene658	12370	NC_000067.6	Casp8	1.36E+00
gene483	72050	NC_000067.6	Kdelc1	1.36E+00
gene23093	73699	NC_000075.6	Ppp2r1b	1.36E+00
gene33447	19012	NC_000079.6	Plpp1	1.36E+00
gene10145	230737	NC_000070.6	Gnl2	1.36E+00
gene39644	266614	NC_000083.6	Ly6g5b	1.36E+00
gene21816	13680	NC_000074.6	Ddx19a	1.36E+00

gene13342	14673	NC_000071.6	Gna12	1.36E+00
gene41091	71983	NC_000084.6	Tmco6	1.36E+00
gene4861	269338	NC_000068.7	Vps39	1.36E+00
gene17724	75835	NC_000073.6	Vmn2r-ps54	1.36E+00
gene30207	109065	NC_000078.6	Dnaaf2	1.35E+00
gene22494	67629	NC_000075.6	Spc24	1.35E+00
gene42828	319740	NC_000085.6	Zfyve27	1.35E+00
gene31130	217893	NC_000078.6	Pacs2	1.35E+00
gene35157	13655	NC_000080.6	Egr3	1.35E+00
gene27149	171285	NC_000077.6	Havcr2	1.35E+00
gene7035	54195	NC_000069.6	Gucy1b3	1.35E+00
gene10394	230801	NC_000070.6	Pigv	1.35E+00
gene35682	414066	NC_000081.6	BC037032	1.35E+00
gene5117	67326	NC_000068.7	1700037H04Rik	1.35E+00
gene15728	78202	NC_000072.6	4930557K07Rik	1.35E+00
gene14266	232784	NC_000072.6	Zfp212	1.35E+00
gene8046	211556	NC_000069.6	Ap1ar	1.35E+00
gene39077	1E+08	NC_000083.6	Gm20008	1.35E+00
gene34371	105504	NC_000080.6	Exoc5	1.35E+00
gene42918	56626	NC_000085.6	Poll	1.35E+00
gene12592	269682	NC_000071.6	Golga3	1.35E+00
gene35872	432940	NC_000081.6	Otulin	1.35E+00
gene13361	74781	NC_000071.6	Wipi2	1.35E+00
gene18300	233335	NC_000073.6	Synm	1.35E+00
gene22621	1E+08	NC_000075.6	Gm3756	1.35E+00
gene15648	60611	NC_000072.6	Foxj2	1.35E+00
gene28036	20317	NC_000077.6	Serpinf1	1.35E+00
gene21031	546071	NC_000074.6	Mast3	1.35E+00
gene42800	83490	NC_000085.6	Pik3ap1	1.35E+00
gene2062	66352	NC_000067.6	Blzf1	1.34E+00
gene34810	12684	NC_000080.6	Cideb	1.34E+00
gene25468	108707	NC_000076.6	Fam207a	1.34E+00
gene21392	12404	NC_000074.6	Cbln1	1.34E+00
gene36673	29858	NC_000081.6	Pmm1	1.34E+00
gene39501	240067	NC_000083.6	Zfp952	1.34E+00
gene12169	231430	NC_000071.6	Cox18	1.34E+00
gene13009	76809	NC_000071.6	Bri3bp	1.34E+00
gene14986	66979	NC_000072.6	Pole4	1.34E+00
gene19556	244216	NC_000073.6	Zfp771	1.34E+00
gene1190	171531	NC_000067.6	Mlph	1.34E+00
gene31074	74335	NC_000078.6	Xrcc3	1.34E+00
gene17864	16504	NC_000073.6	Kcnc3	1.34E+00
gene32947	1.1E+08	NC_000079.6	Gm41002	1.34E+00
gene12937	67151	NC_000071.6	Psmd9	1.34E+00
gene40589	56468	NC_000083.6	Socs5	1.34E+00
gene3372	56017	NC_000068.7	Slc2a8	1.34E+00
gene646	65102	NC_000067.6	Nif3l1	1.34E+00
gene31510	217980	NC_000079.6	Larp4b	1.34E+00

gene15456	57890	NC_000072.6	Il17re	1.34E+00
gene17251	414069	NC_000073.6	BC024978	1.34E+00
gene35227	20916	NC_000080.6	Sucla2	1.34E+00
gene12618	56305	NC_000071.6	Pitpnb	1.34E+00
gene29766	1E+08	NC_000078.6	Gm36496	1.34E+00
gene11683	18196	NC_000071.6	Nsg1	1.34E+00
gene38384	67374	NC_000082.6	Jam2	1.34E+00
gene5047	69737	NC_000068.7	Ttl	1.34E+00
gene39480	70101	NC_000083.6	Cyp4f16	1.34E+00
gene34924	71844	NC_000080.6	Nupl1	1.34E+00
gene8637	51813	NC_000070.6	Ccnc	1.34E+00
gene25391	74392	NC_000076.6	Specc1l	1.34E+00
gene30476	67039	NC_000078.6	Rbm25	1.33E+00
gene8807	622208	NC_000070.6	Gm6297	1.33E+00
gene13245	170741	NC_000071.6	Pilrb1	1.33E+00
gene41191	53601	NC_000084.6	Pcdh12	1.33E+00
gene35683	67041	NC_000081.6	Oxct1	1.33E+00
gene5403	386649	NC_000068.7	Nsfl1c	1.33E+00
gene22514	26940	NC_000075.6	Ecsit	1.33E+00
gene27363	12981	NC_000077.6	Csf2	1.33E+00
gene40225	50930	NC_000083.6	Tnfsf14	1.33E+00
gene23162	64602	NC_000075.6	Ireb2	1.33E+00
gene7569	67845	NC_000069.6	Rnf115	1.33E+00
gene33793	1E+08	NC_000080.6	Gm19061	1.33E+00
gene37096	22379	NC_000081.6	Fmnl3	1.33E+00
gene918	74104	NC_000067.6	Abcb6	1.33E+00
gene5827	110750	NC_000068.7	Cse1l	1.33E+00
gene25241	94213	NC_000076.6	Ddx50	1.33E+00
gene15877	16641	NC_000072.6	Klrc1	1.33E+00
gene11650	66717	NC_000071.6	Ccdc96	1.33E+00
gene5091	19262	NC_000068.7	Ptpra	1.33E+00
gene40457	73694	NC_000083.6	Ndufaf7	1.33E+00
gene39304	224630	NC_000083.6	Bnip1	1.32E+00
gene25663	50492	NC_000076.6	Thop1	1.32E+00
gene42238	12511	NC_000085.6	Cd6	1.32E+00
gene42532	1E+08	NC_000085.6	Gm34186	1.32E+00
gene27539	66771	NC_000077.6	Gid4	1.32E+00
gene15000	16950	NC_000072.6	Loxl3	1.32E+00
gene35791	22763	NC_000081.6	Zfr	1.32E+00
gene32185	66143	NC_000079.6	Eef1e1	1.32E+00
gene20209	234076	NC_000074.6	Tmco3	1.32E+00
gene10288	230779	NC_000070.6	Serinc2	1.32E+00
gene24189	72831	NC_000075.6	Dhx30	1.32E+00
gene37537	69101	NC_000082.6	Ydjc	1.32E+00
gene5276	1E+08	NC_000068.7	Gm34292	1.32E+00
gene17576	233103	NC_000073.6	4931406P16Rik	1.32E+00
gene2209	66425	NC_000067.6	Pcp4l1	1.32E+00
gene13797	626180	NC_000072.6	Gm6655	1.32E+00

gene38909	68799	NC_000083.6	Rgmb	1.32E+00
gene5014	668892	NC_000068.7	Gm14245	1.32E+00
gene35695	105787	NC_000081.6	Prkaa1	1.32E+00
gene22058	72325	NC_000074.6	Vps9d1	1.32E+00
gene31127	72308	NC_000078.6	Brf1	1.32E+00
gene34337	67296	NC_000080.6	Socs4	1.32E+00
gene28275	66330	NC_000077.6	1700020L24Rik	1.32E+00
gene26687	209586	NC_000077.6	Nudcd3	1.32E+00
gene31986	214973	NC_000079.6	Gm11349	1.32E+00
gene27753	18626	NC_000077.6	Per1	1.32E+00
gene18850	1.1E+08	NC_000073.6	Gm39061	1.32E+00
gene13484	22217	NC_000071.6	Usp12	1.32E+00
gene1638	70579	NC_000067.6	Zc3h11a	1.32E+00
gene27505	194952	NC_000077.6	Jmjd4	1.32E+00
gene6802	20439	NC_000069.6	Siah2	1.32E+00
gene24082	1E+08	NC_000075.6	Gm34106	1.32E+00
gene10263	66264	NC_000070.6	Ccdc28b	1.32E+00
gene653	1E+08	NC_000067.6	Cbx3-ps7	1.32E+00
gene5887	11981	NC_000068.7	Atp9a	1.31E+00
gene28612	217149	NC_000077.6	Cisd3	1.31E+00
gene31389	56220	NC_000078.6	Zfp386	1.31E+00
gene22595	102607	NC_000075.6	Snx19	1.31E+00
gene25629	66932	NC_000076.6	Rexo1	1.31E+00
gene41394	629043	NC_000084.6	Gm6942	1.31E+00
gene42593	226090	NC_000085.6	Ermp1	1.31E+00
gene5667	21969	NC_000068.7	Top1	1.31E+00
gene6192	269401	NC_000068.7	Zfp512b	1.31E+00
gene9011	74735	NC_000070.6	Trim14	1.31E+00
gene22255	17394	NC_000075.6	Mmp8	1.31E+00
gene39702	240087	NC_000083.6	Mdc1	1.31E+00
gene38055	239849	NC_000082.6	Cd200r4	1.31E+00
gene34857	219105	NC_000080.6	Zmym5	1.31E+00
gene560	14660	NC_000067.6	Gls	1.31E+00
gene34866	14619	NC_000080.6	Gjb2	1.31E+00
gene7271	229543	NC_000069.6	Ints3	1.31E+00
gene9254	70052	NC_000070.6	Prpf4	1.31E+00
gene1538	17219	NC_000067.6	Mcm6	1.31E+00
gene34901	71330	NC_000080.6	Rcbtb1	1.31E+00
gene7036	60596	NC_000069.6	Gucy1a3	1.31E+00
gene32955	211446	NC_000079.6	Exoc3	1.31E+00
gene20500	78560	NC_000074.6	Adgra2	1.31E+00
gene1694	226432	NC_000067.6	Ipo9	1.31E+00
gene18500	67290	NC_000073.6	3110040N11Rik	1.31E+00
gene15562	213895	NC_000072.6	Bms1	1.31E+00
gene19622	246787	NC_000073.6	Slc5a2	1.31E+00
gene21640	72361	NC_000074.6	Ces2g	1.31E+00
gene24395	1E+08	NC_000075.6	Gm34425	1.31E+00
gene5171	66586	NC_000068.7	Crls1	1.31E+00

gene10486	99982	NC_000070.6	Kdm1a	1.31E+00
gene34954	66674	NC_000080.6	Sprydy7	1.31E+00
gene351	214895	NC_000067.6	Lman2l	1.31E+00
gene18470	71968	NC_000073.6	Wdr73	1.31E+00
gene26578	1E+08	NC_000077.6	Gm33862	1.30E+00
gene3304	277463	NC_000068.7	Gpr107	1.30E+00
gene25512	110816	NC_000076.6	Pwp2	1.30E+00
gene20737	192169	NC_000074.6	Ufsp2	1.30E+00
gene223	68002	NC_000067.6	Sdhaf4	1.30E+00
gene26640	18016	NC_000077.6	Nf2	1.30E+00
gene21749	67619	NC_000074.6	Nob1	1.30E+00
gene34859	1E+08	NC_000080.6	2410022M11Rik	1.30E+00
gene28971	17534	NC_000077.6	Mrc2	1.30E+00
gene42861	226139	NC_000085.6	Cox15	1.30E+00
gene37998	56375	NC_000082.6	B4galt4	1.30E+00
gene12849	170756	NC_000071.6	Slc8b1	1.30E+00
gene10241	107271	NC_000070.6	Yars	1.30E+00
gene36898	=Gene;ger	NC_000081.6	LOC102640133	1.30E+00
gene36310	383050	NC_000081.6	Gm5216	1.30E+00
gene1935	77352	NC_000067.6	Axdnd1	1.30E+00
gene39489	208292	NC_000083.6	Zfp871	1.30E+00
gene35929	114128	NC_000081.6	Laptm4b	1.30E+00
gene39224	1E+08	NC_000083.6	Gm17801	1.30E+00
gene4825	72008	NC_000068.7	Zfyve19	1.30E+00
gene27888	69723	NC_000077.6	Rpain	1.30E+00
gene36260	223601	NC_000081.6	Fam49b	1.30E+00
gene28288	20303	NC_000077.6	Ccl4	1.30E+00
gene16226	622481	NC_000072.6	Gm15784	1.29E+00
gene10637	69582	NC_000070.6	Plekhm2	1.29E+00
gene7999	210529	NC_000069.6	Mettl14	1.29E+00
gene11544	72195	NC_000071.6	Supt7l	1.29E+00
gene34418	11546	NC_000080.6	Parp2	1.29E+00
gene37635	108105	NC_000082.6	B3gnt5	1.29E+00
gene29458	69709	NC_000078.6	Ptrhd1	1.29E+00
gene21656	277978	NC_000074.6	Exoc3l	1.29E+00
gene25669	59004	NC_000076.6	Pias4	1.29E+00
gene4553	13661	NC_000068.7	Ehf	1.29E+00
gene14330	58894	NC_000072.6	Zfp862-ps	1.29E+00
gene28102	216965	NC_000077.6	Taok1	1.29E+00
gene39198	14651	NC_000083.6	Hagh	1.29E+00
gene34126	218914	NC_000080.6	Wapl	1.29E+00
gene40729	jene=LOC'	NC_000084.6	LOC101055995	1.29E+00
gene2454	403180	NC_000067.6	Ccdc121	1.29E+00
gene20402	22335	NC_000074.6	Vdac3	1.29E+00
gene9892	230657	NC_000070.6	Tmem69	1.29E+00
gene34139	72972	NC_000080.6	Ccser2	1.29E+00
gene19766	668771	NC_000073.6	Gm9347	1.29E+00
gene20604	666792	NC_000074.6	Gm8291	1.29E+00

gene34794	85308	NC_000080.6	Emc9	1.29E+00
gene18393	207952	NC_000073.6	Klhl25	1.29E+00
gene8170	70604	NC_000069.6	Dnajb14	1.28E+00
gene40595	225049	NC_000083.6	Ttc7	1.28E+00
gene36417	78834	NC_000081.6	Zfp623	1.28E+00
gene33262	20365	NC_000079.6	Serf1	1.28E+00
gene6618	18415	NC_000069.6	Hspa4l	1.28E+00
gene7575	14613	NC_000069.6	Gja5	1.28E+00
gene13439	67235	NC_000071.6	Zkscan14	1.28E+00
gene15174	23945	NC_000072.6	Mgll	1.28E+00
gene34491	67772	NC_000080.6	Chd8	1.28E+00
gene39291	66978	NC_000083.6	Luc7l	1.28E+00
gene37118	65970	NC_000081.6	Lima1	1.28E+00
gene21166	234463	NC_000074.6	Tmem184c	1.28E+00
gene21515	107566	NC_000074.6	Arl2bp	1.28E+00
gene34481	268739	NC_000080.6	Arhgef40	1.28E+00
gene13718	320312	NC_000072.6	A430035B10Rik	1.28E+00
gene8831	67123	NC_000070.6	Ubap1	1.28E+00
gene9065	67063	NC_000070.6	Tmem246	1.28E+00
gene7013	67738	NC_000069.6	Ppid	1.28E+00
gene7636	269470	NC_000069.6	Wdr3	1.28E+00
gene15993	232430	NC_000072.6	Crebl2	1.28E+00
gene28654	13866	NC_000077.6	Erbb2	1.28E+00
gene34236	652988	NC_000080.6	Gm7324	1.28E+00
gene26461	13867	NC_000076.6	Erbb3	1.28E+00
gene7880	66500	NC_000069.6	Slc30a7	1.28E+00
gene10395	93760	NC_000070.6	Arid1a	1.28E+00
gene5633	72340	NC_000068.7	2010009K17Rik	1.28E+00
gene17734	434175	NC_000073.6	Gm5593	1.28E+00
gene14862	19895	NC_000072.6	Rpia	1.28E+00
gene42170	75291	NC_000085.6	Zbtb3	1.28E+00
gene5037	17222	NC_000068.7	Anapc1	1.28E+00
gene34334	14528	NC_000080.6	Gch1	1.28E+00
gene22162	74393	NC_000074.6	Map10	1.28E+00
gene29601	217410	NC_000078.6	Trib2	1.28E+00
gene25619	66548	NC_000076.6	Adamtsl5	1.28E+00
gene43021	13006	NC_000085.6	Smc3	1.28E+00
gene22513	1E+08	NC_000075.6	Gm16845	1.28E+00
gene37927	16646	NC_000082.6	Kpna1	1.28E+00
gene36422	105732	NC_000081.6	Fam83h	1.28E+00
gene37573	13353	NC_000082.6	Dgcr6	1.27E+00
gene27615	20185	NC_000077.6	Ncor1	1.27E+00
gene39661	66237	NC_000083.6	Atp6v1g2	1.27E+00
gene8172	56692	NC_000069.6	Lamtor3	1.27E+00
gene10481	74326	NC_000070.6	Hnrnpr	1.27E+00
gene3360	27387	NC_000068.7	Sh2d3c	1.27E+00
gene42939	73728	NC_000085.6	Psd	1.27E+00
gene22978	14012	NC_000075.6	Mpzl2	1.27E+00

gene9188	67103	NC_000070.6	Ptgr1	1.27E+00
gene1492	72999	NC_000067.6	Insig2	1.27E+00
gene4738	1E+08	NC_000068.7	C130080G10Rik	1.27E+00
gene34396	239081	NC_000080.6	Tlr11	1.27E+00
gene37466	106298	NC_000082.6	Rrn3	1.27E+00
gene6183	229007	NC_000068.7	Zgpat	1.27E+00
gene11742	231225	NC_000071.6	Tapt1	1.27E+00
gene24941	11702	NC_000076.6	Amd1	1.27E+00
gene28927	353047	NC_000077.6	Plekhm1	1.27E+00
gene34038	66487	NC_000080.6	Smim4	1.27E+00
gene34048	12339	NC_000080.6	Capn7	1.27E+00
gene10477	242705	NC_000070.6	E2f2	1.27E+00
gene31379	668586	NC_000078.6	Gm9256	1.27E+00
gene41290	225467	NC_000084.6	Pggt1b	1.27E+00
gene30778	27277	NC_000078.6	Golga5	1.27E+00
gene9258	192120	NC_000070.6	Bspry	1.27E+00
gene14442	72628	NC_000072.6	Hoxaas3	1.27E+00
gene22564	666327	NC_000075.6	Gm8049	1.27E+00
gene27538	246782	NC_000077.6	Atpaf2	1.27E+00
gene6205	245867	NC_000068.7	Pcmtd2	1.27E+00
gene38586	338467	NC_000082.6	Morc3	1.27E+00
gene40280	17158	NC_000083.6	Man2a1	1.27E+00
gene5464	12048	NC_000068.7	Bcl2l1	1.27E+00
gene8584	72201	NC_000070.6	Otud6b	1.27E+00
gene7479	71807	NC_000069.6	Tars2	1.26E+00
gene13295	77053	NC_000071.6	Sun1	1.26E+00
gene21313	619292	NC_000074.6	G430095P16Rik	1.26E+00
gene37929	70186	NC_000082.6	Fam162a	1.26E+00
gene26382	1E+08	NC_000076.6	Gm20492	1.26E+00
gene21034	70807	NC_000074.6	Arrdc2	1.26E+00
gene31568	70430	NC_000079.6	Tbce	1.26E+00
gene41200	24066	NC_000084.6	Spry4	1.26E+00
gene7980	214459	NC_000069.6	Fnbp1l	1.26E+00
gene19786	52432	NC_000073.6	Ppp2r2d	1.26E+00
gene23730	109785	NC_000075.6	Pgm3	1.26E+00
gene11298	212167	NC_000071.6	Gsap	1.26E+00
gene20928	17961	NC_000074.6	Nat2	1.26E+00
gene15694	667277	NC_000072.6	C1rb	1.26E+00
gene13813	27999	NC_000072.6	Fam3c	1.26E+00
gene24223	235636	NC_000075.6	Rtp3	1.26E+00
gene17895	233210	NC_000073.6	Prr12	1.26E+00
gene26847	67245	NC_000077.6	Peli1	1.26E+00
gene12371	71883	NC_000071.6	Coq2	1.26E+00
gene19922	11772	NC_000073.6	Ap2a2	1.26E+00
gene25327	12534	NC_000076.6	Cdk1	1.26E+00
gene34170	70564	NC_000080.6	Fam213a	1.26E+00
gene14032	192196	NC_000072.6	Luc7l2	1.25E+00
gene31974	56196	NC_000079.6	Tdp2	1.25E+00

gene40742	225155	NC_000084.6	Gm4834	1.25E+00
gene23452	80907	NC_000075.6	Lactb	1.25E+00
gene32111	69666	NC_000079.6	Psmg4	1.25E+00
gene14244	26965	NC_000072.6	Cul1	1.25E+00
gene64	665138	NC_000067.6	Gm7512	1.25E+00
gene25259	93759	NC_000076.6	Sirt1	1.25E+00
gene24334	54214	NC_000075.6	Golga4	1.25E+00
gene265	19243	NC_000067.6	Ptp4a1	1.25E+00
gene16808	434130	NC_000073.6	Ccdc8	1.25E+00
gene25102	432460	NC_000076.6	Tmem229b-ps	1.25E+00
gene34886	75965	NC_000080.6	Zdhhc20	1.25E+00
gene36333	105841	NC_000081.6	Dennd3	1.25E+00
gene10572	69116	NC_000070.6	Ubr4	1.25E+00
gene40935	625997	NC_000084.6	Gm6643	1.25E+00
gene13339	231841	NC_000071.6	Brat1	1.25E+00
gene17319	63986	NC_000073.6	Gmfg	1.25E+00
gene32717	66129	NC_000079.6	Aaed1	1.25E+00
gene39709	66973	NC_000083.6	Mrps18b	1.25E+00
gene23946	17973	NC_000075.6	Nck1	1.25E+00
gene11987	1E+08	NC_000071.6	Gm34902	1.25E+00
gene24727	237336	NC_000076.6	Tbpl1	1.25E+00
gene3754	20272	NC_000068.7	Scn7a	1.25E+00
gene16265	69714	NC_000073.6	Tfpt	1.25E+00
gene36677	14375	NC_000081.6	Xrcc6	1.25E+00
gene21975	234814	NC_000074.6	Mthfsd	1.25E+00
gene24111	320844	NC_000075.6	Amigo3	1.25E+00
gene39938	1.1E+08	NC_000083.6	B230354K17Rik	1.25E+00
gene19637	210711	NC_000073.6	Mcmbp	1.25E+00
gene39117	240047	NC_000083.6	Mmp25	1.25E+00
gene13257	74570	NC_000071.6	Zkscan1	1.25E+00
gene6001	228960	NC_000068.7	Stx16	1.24E+00
gene7893	66368	NC_000069.6	Rtca	1.24E+00
gene2172	1.1E+08	NC_000067.6	Gm39701	1.24E+00
gene24144	19087	NC_000075.6	Prkar2a	1.24E+00
gene7450	1E+08	NC_000069.6	Gm16740	1.24E+00
gene21708	18021	NC_000074.6	Nfatc3	1.24E+00
gene7741	109050	NC_000069.6	Fam212b	1.24E+00
gene28690	57376	NC_000077.6	Smarce1	1.24E+00
gene10179	24084	NC_000070.6	Tekt2	1.24E+00
gene19301	69295	NC_000073.6	Far1os	1.24E+00
gene8451	17096	NC_000070.6	Lyn	1.24E+00
gene12543	116701	NC_000071.6	Fgfrl1	1.24E+00
gene36847	223770	NC_000081.6	Brd1	1.24E+00
gene16889	13870	NC_000073.6	Ercc1	1.24E+00
gene15065	52055	NC_000072.6	Rab11fip5	1.24E+00
gene37273	29813	NC_000081.6	Zfp385a	1.24E+00
gene11030	140499	NC_000070.6	Ube2j2	1.24E+00
gene21926	71839	NC_000074.6	Osgin1	1.24E+00

gene37656	208624	NC_000082.6	Alg3	1.24E+00
gene4943	59010	NC_000068.7	Sqrdl	1.24E+00
gene26584	56218	NC_000077.6	Patz1	1.24E+00
gene12880	ene=LOC1	NC_000071.6	LOC108169089	1.24E+00
gene4845	228550	NC_000068.7	Itpka	1.24E+00
gene1272	66615	NC_000067.6	Atg4b	1.24E+00
gene11565	320951	NC_000071.6	Pisd	1.23E+00
gene33239	238799	NC_000079.6	Tnpo1	1.23E+00
gene41944	225876	NC_000085.6	Kdm2a	1.23E+00
gene41063	56550	NC_000084.6	Ube2d2a	1.23E+00
gene8720	1E+08	NC_000070.6	4933421O10Rik	1.23E+00
gene27414	67726	NC_000077.6	Fam114a2	1.23E+00
gene28657	1.1E+08	NC_000077.6	Gm38975	1.23E+00
gene6195	68879	NC_000068.7	Prpf6	1.23E+00
gene20088	15568	NC_000074.6	Elavl1	1.23E+00
gene42235	107305	NC_000085.6	Vps37c	1.23E+00
gene11947	67980	NC_000071.6	Gnpda2	1.23E+00
gene21787	73608	NC_000074.6	Marveld3	1.23E+00
gene4775	68215	NC_000068.7	Fam98b	1.23E+00
gene9779	100273	NC_000070.6	Osbpl9	1.23E+00
gene32103	18105	NC_000079.6	Nqo2	1.23E+00
gene21618	382051	NC_000074.6	Pdp2	1.23E+00
gene42060	69024	NC_000085.6	Snx15	1.23E+00
gene5105	613258	NC_000068.7	A730017L22Rik	1.23E+00
gene35217	105670	NC_000080.6	Rcbtb2	1.23E+00
gene37521	436405	NC_000082.6	Gm5768	1.23E+00
gene31044	67236	NC_000078.6	Cinp	1.23E+00
gene39530	54219	NC_000083.6	Cd320	1.23E+00
gene5840	56336	NC_000068.7	B4galt5	1.23E+00
gene8992	72535	NC_000070.6	Aldh1b1	1.23E+00
gene22301	72826	NC_000075.6	Fam76b	1.23E+00
gene2734	66540	NC_000068.7	Fam107b	1.23E+00
gene37995	12549	NC_000082.6	Arhgap31	1.23E+00
gene18676	72461	NC_000073.6	Prcp	1.23E+00
gene2595	226844	NC_000067.6	Mfsd7b	1.23E+00
gene39155	11984	NC_000083.6	Atp6v0c	1.23E+00
gene9729	230584	NC_000070.6	Yipf1	1.23E+00
gene8190	75624	NC_000069.6	Metap1	1.23E+00
gene29010	380732	NC_000077.6	Milr1	1.23E+00
gene41206	14164	NC_000084.6	Fgf1	1.23E+00
gene5476	228788	NC_000068.7	Ccm2l	1.22E+00
gene32176	109620	NC_000079.6	Dsp	1.22E+00
gene355	94218	NC_000067.6	Cnnm3	1.22E+00
gene12006	74277	NC_000071.6	Chic2	1.22E+00
gene23212	72774	NC_000075.6	Neil1	1.22E+00
gene921	74577	NC_000067.6	Glb1l	1.22E+00
gene7015	75939	NC_000069.6	4930579G24Rik	1.22E+00
gene22165	66566	NC_000074.6	Ntpcr	1.22E+00

gene24134	69232	NC_000075.6	Qrich1	1.22E+00
gene1204	67444	NC_000067.6	Ilkap	1.22E+00
gene27612	11541	NC_000077.6	Adora2b	1.22E+00
gene28243	16882	NC_000077.6	Lig3	1.22E+00
gene10819	28010	NC_000070.6	Miip	1.22E+00
gene24870	212892	NC_000076.6	Rsph4a	1.22E+00
gene2529	107508	NC_000067.6	Eprs	1.22E+00
gene39576	381091	NC_000083.6	H2-Eb2	1.22E+00
gene24881	1E+08	NC_000076.6	Gm26564	1.22E+00
gene32839	212276	NC_000079.6	Zfp748	1.22E+00
gene42901	1E+08	NC_000085.6	Pdzd7	1.22E+00
gene37648	13544	NC_000082.6	Dvl3	1.22E+00
gene23795	67016	NC_000075.6	Tbc1d2b	1.22E+00
gene10960	230967	NC_000070.6	Cep104	1.22E+00
gene22861	71566	NC_000075.6	Clmp	1.22E+00
gene598	67876	NC_000067.6	Coq10b	1.22E+00
gene35159	57784	NC_000080.6	Bin3	1.22E+00
gene24421	56295	NC_000075.6	Higd1a	1.22E+00
gene15439	14911	NC_000072.6	Thumpd3	1.22E+00
gene2525	381314	NC_000067.6	Iars2	1.22E+00
gene24372	208638	NC_000075.6	Slc25a38	1.22E+00
gene23556	546143	NC_000075.6	Ccpg1os	1.22E+00
gene30030	544864	NC_000078.6	Gm5785	1.22E+00
gene23624	71538	NC_000075.6	Fbxo9	1.22E+00
gene36658	328572	NC_000081.6	Ep300	1.22E+00
gene41599	225651	NC_000084.6	Mppe1	1.22E+00
gene25763	ene=LOC1	NC_000076.6	LOC102636894	1.22E+00
gene25427	64453	NC_000076.6	Zfp280b	1.22E+00
gene34024	69179	NC_000080.6	Tmem110	1.21E+00
gene21774	234723	NC_000074.6	Txnl4b	1.21E+00
gene41482	18596	NC_000084.6	Pdgfrb	1.21E+00
gene36670	78929	NC_000081.6	Polr3h	1.21E+00
gene37890	69150	NC_000082.6	Snx4	1.21E+00
gene5533	67204	NC_000068.7	Eif2s2	1.21E+00
gene24554	67418	NC_000076.6	Ppil4	1.21E+00
gene20966	330788	NC_000074.6	Zfp866	1.21E+00
gene33112	69085	NC_000079.6	Zcchc9	1.21E+00
gene37477	223989	NC_000082.6	Marf1	1.21E+00
gene37687	72190	NC_000082.6	2510009E07Rik	1.21E+00
gene31971	57441	NC_000079.6	Gmnn	1.21E+00
gene12998	20778	NC_000071.6	Scarb1	1.21E+00
gene8109	668857	NC_000069.6	Gm9402	1.21E+00
gene36864	170787	NC_000081.6	Hdac10	1.21E+00
gene29860	30955	NC_000078.6	Pik3cg	1.21E+00
gene26754	22778	NC_000077.6	Ikzf1	1.21E+00
gene27689	66358	NC_000077.6	Adprm	1.21E+00
gene9041	76299	NC_000070.6	Erp44	1.21E+00
gene18816	207212	NC_000073.6	Arhgef17	1.21E+00

gene9877	72181	NC_000070.6	Nsun4	1.21E+00
gene11928	109108	NC_000071.6	Slc30a9	1.21E+00
gene27100	30939	NC_000077.6	Pttg1	1.21E+00
gene40149	1E+08	NC_000083.6	Gm16712	1.21E+00
gene31516	66505	NC_000079.6	Zmynd11	1.21E+00
gene18550	14085	NC_000073.6	Fah	1.21E+00
gene24529	67141	NC_000076.6	Fbxo5	1.21E+00
gene9784	13858	NC_000070.6	Eps15	1.21E+00
gene28200	68550	NC_000077.6	Tefm	1.20E+00
gene41675	107029	NC_000084.6	Me2	1.20E+00
gene30143	217615	NC_000078.6	Ctage5	1.20E+00
gene14272	69228	NC_000072.6	Zfp746	1.20E+00
gene3864	215280	NC_000068.7	Wipf1	1.20E+00
gene35180	239170	NC_000080.6	Fam160b2	1.20E+00
gene7894	13171	NC_000069.6	Dbt	1.20E+00
gene26264	66225	NC_000076.6	Llph	1.20E+00
gene17273	11652	NC_000073.6	Akt2	1.20E+00
gene13381	231866	NC_000071.6	Zfp12	1.20E+00
gene32812	19210	NC_000079.6	Ptdss1	1.20E+00
gene29846	104836	NC_000078.6	Cbll1	1.20E+00
gene28813	14055	NC_000077.6	Ezh1	1.20E+00
gene937	98733	NC_000067.6	Obsl1	1.20E+00
gene26179	71086	NC_000076.6	4933412E12Rik	1.20E+00
gene23366	66939	NC_000075.6	Aagab	1.20E+00
gene18501	83962	NC_000073.6	Btbd1	1.20E+00
gene38033	=Gene;ger	NC_000082.6	LOC108168290	1.20E+00
gene36661	214685	NC_000081.6	Chadl	1.20E+00
gene7726	17454	NC_000069.6	Mov10	1.20E+00
gene12590	231600	NC_000071.6	Chfr	1.20E+00
gene36132	69790	NC_000081.6	Med30	1.20E+00
gene32051	16364	NC_000079.6	Irf4	1.20E+00
gene26472	13139	NC_000076.6	Dgka	1.20E+00
gene19569	69234	NC_000073.6	Zfp688	1.20E+00
gene17113	210135	NC_000073.6	Zfp180	1.20E+00
gene30351	268564	NC_000078.6	Zbtb1	1.20E+00
gene9299	69807	NC_000070.6	Trim32	1.20E+00
gene3325	241303	NC_000068.7	Fam78a	1.20E+00
gene41900	225888	NC_000085.6	Suv420h1	1.20E+00
gene37850	72084	NC_000082.6	Pigx	1.20E+00
gene22299	77116	NC_000075.6	Mtmr2	1.20E+00
gene22661	76832	NC_000075.6	Hyls1	1.20E+00
gene9751	242608	NC_000070.6	Podn	1.20E+00
gene19685	214580	NC_000073.6	Pstk	1.20E+00
gene37090	51812	NC_000081.6	Mcrs1	1.20E+00
gene2072	1E+08	NC_000067.6	Gm32569	1.20E+00
gene1584	226421	NC_000067.6	Rab7b	1.20E+00
gene20903	73067	NC_000074.6	Tmem192	1.19E+00
gene43019	240672	NC_000085.6	Dusp5	1.19E+00

gene42632	67979	NC_000085.6	Atad1	1.19E+00
gene1562	=Gene;ger	NC_000067.6	LOC102640771	1.19E+00
gene33942	11744	NC_000080.6	Anxa11	1.19E+00
gene40994	225348	NC_000084.6	Wdr36	1.19E+00
gene35441	380928	NC_000080.6	Lmo7	1.19E+00
gene42870	12675	NC_000085.6	Chuk	1.19E+00
gene33528	1.1E+08	NC_000079.6	Gm41077	1.19E+00
gene4831	1E+08	NC_000068.7	Gm14207	1.19E+00
gene17329	70584	NC_000073.6	Pak4	1.19E+00
gene6136	252966	NC_000068.7	Cables2	1.19E+00
gene645	70225	NC_000067.6	Ppil3	1.19E+00
gene26594	29856	NC_000077.6	Smtn	1.19E+00
gene5693	245866	NC_000068.7	Ift52	1.19E+00
gene24879	212898	NC_000076.6	Dse	1.19E+00
gene35718	18414	NC_000081.6	Osmr	1.19E+00
gene20766	192174	NC_000074.6	Rwdd4a	1.19E+00
gene36040	75766	NC_000081.6	Dcstamp	1.19E+00
gene41500	545261	NC_000084.6	Bvht	1.19E+00
gene1557	226418	NC_000067.6	Yod1	1.19E+00
gene42427	107272	NC_000085.6	Psat1	1.19E+00
gene5723	67145	NC_000068.7	Tomm34	1.19E+00
gene3601	74194	NC_000068.7	Rnd3	1.19E+00
gene29181	445007	NC_000077.6	Nup85	1.19E+00
gene1212	65247	NC_000067.6	Asb1	1.19E+00
gene19411	67983	NC_000073.6	Pdzd9	1.19E+00
gene5773	415115	NC_000068.7	Neurl2	1.19E+00
gene34770	1E+08	NC_000080.6	Gm31251	1.19E+00
gene12583	1E+08	NC_000071.6	Gm17655	1.19E+00
gene42957	57344	NC_000085.6	As3mt	1.19E+00
gene42790	72672	NC_000085.6	Zfp518a	1.19E+00
gene37510	74006	NC_000082.6	Dnm1l	1.19E+00
gene32946	105246	NC_000079.6	Brd9	1.19E+00
gene15518	28006	NC_000072.6	Fam21	1.18E+00
gene27599	13855	NC_000077.6	Epn2	1.18E+00
gene11611	75416	NC_000071.6	Nop14	1.18E+00
gene38538	1E+08	NC_000082.6	Gm10785	1.18E+00
gene3507	59126	NC_000068.7	Nek6	1.18E+00
gene6442	1E+08	NC_000069.6	Gm32950	1.18E+00
gene11606	231130	NC_000071.6	Tnip2	1.18E+00
gene28277	70439	NC_000077.6	Taf15	1.18E+00
gene17477	52857	NC_000073.6	Gramd1a	1.18E+00
gene10203	1E+08	NC_000070.6	Gm33269	1.18E+00
gene36615	1.1E+08	NC_000081.6	Gm41361	1.18E+00
gene3379	71834	NC_000068.7	Zbtb43	1.18E+00
gene25911	237459	NC_000076.6	Cdk17	1.18E+00
gene21670	70796	NC_000074.6	Zdhhc1	1.18E+00
gene42201	623688	NC_000085.6	Gm6445	1.18E+00
gene41884	353242	NC_000085.6	Mrpl21	1.18E+00

gene6746	229285	NC_000069.6	Spg20	1.18E+00
gene22227	67618	NC_000075.6	Aasdhppt	1.18E+00
gene10626	29818	NC_000070.6	Hspb7	1.18E+00
gene22203	1E+08	NC_000074.6	Gm32643	1.18E+00
gene35623	223255	NC_000080.6	Stk24	1.18E+00
gene26261	1E+08	NC_000076.6	Gm38500	1.18E+00
gene36921	66725	NC_000081.6	Lrrk2	1.18E+00
gene29015	66313	NC_000077.6	Smurf2	1.18E+00
gene34950	16648	NC_000080.6	Kpna3	1.17E+00
gene31639	353346	NC_000079.6	Gpr141	1.17E+00
gene12750	18778	NC_000071.6	Pla2g1b	1.17E+00
gene15252	22591	NC_000072.6	Xpc	1.17E+00
gene1465	66343	NC_000067.6	Tmem177	1.17E+00
gene38644	72388	NC_000082.6	Ripk4	1.17E+00
gene33333	66975	NC_000079.6	Trappc13	1.17E+00
gene42704	72514	NC_000085.6	Fgfbp3	1.17E+00
gene22448	13433	NC_000075.6	Dnmt1	1.17E+00
gene20613	244416	NC_000074.6	Ppp1r3b	1.17E+00
gene26600	1E+08	NC_000077.6	Gm11951	1.17E+00
gene16298	17086	NC_000073.6	Ncr1	1.17E+00
gene34806	21816	NC_000080.6	Tgm1	1.17E+00
gene1871	226519	NC_000067.6	Lamc1	1.17E+00
gene21301	67873	NC_000074.6	Mri1	1.17E+00
gene11986	24051	NC_000071.6	Sgcb	1.17E+00
gene42265	64380	NC_000085.6	Ms4a4c	1.17E+00
gene11546	100740	NC_000071.6	AI839979	1.17E+00
gene29997	217536	NC_000078.6	Gm1818	1.17E+00
gene38051	212547	NC_000082.6	Nepro	1.17E+00
gene38392	224405	NC_000082.6	Cyyr1	1.17E+00
gene39151	69259	NC_000083.6	Kctd5	1.16E+00
gene28359	1.1E+08	NC_000077.6	Gm42085	1.16E+00
gene10545	1E+08	NC_000070.6	Gm32871	1.16E+00
gene5838	19223	NC_000068.7	Ptgis	1.16E+00
gene42173	14704	NC_000085.6	Gng3	1.16E+00
gene24415	18087	NC_000075.6	Nktr	1.16E+00
gene27718	320207	NC_000077.6	Pik3r5	1.16E+00
gene28316	70325	NC_000077.6	Pigw	1.16E+00
gene18818	233571	NC_000073.6	P2ry6	1.16E+00
gene1151	1.1E+08	NC_000067.6	Gm41912	1.16E+00
gene43135	78832	NC_000085.6	Cacul1	1.16E+00
gene40916	58523	NC_000084.6	Elp2	1.16E+00
gene136	98711	NC_000067.6	Rdh10	1.16E+00
gene24824	52665	NC_000076.6	Echdc1	1.16E+00
gene34419	21745	NC_000080.6	Tep1	1.16E+00
gene27815	216859	NC_000077.6	Acap1	1.16E+00
gene32960	432779	NC_000079.6	Lrrc14b	1.16E+00
gene25198	13180	NC_000076.6	Pcbd1	1.16E+00
gene11586	21335	NC_000071.6	Tacc3	1.16E+00

gene5463	84682	NC_000068.7	Cox4i2	1.15E+00
gene39747	79263	NC_000083.6	Trim39	1.15E+00
gene11516	69719	NC_000071.6	Cad	1.15E+00
gene10785	626288	NC_000070.6	Gm13140	1.15E+00
gene20471	23808	NC_000074.6	Ash2l	1.15E+00
gene12987	21871	NC_000071.6	Atp6v0a2	1.15E+00
gene8983	242418	NC_000070.6	Dcaf10	1.15E+00
gene29025	68979	NC_000077.6	Nol11	1.15E+00
gene12975	243272	NC_000071.6	Sbno1	1.15E+00
gene27009	268396	NC_000077.6	Sh3pxd2b	1.15E+00
gene12804	231672	NC_000071.6	Fbxw8	1.15E+00
gene3234	76220	NC_000068.7	6530402F18Rik	1.15E+00
gene11636	231147	NC_000071.6	Sh3tc1	1.15E+00
gene264	213109	NC_000067.6	Phf3	1.15E+00
gene21943	234796	NC_000074.6	Klhl36	1.15E+00
gene9622	72157	NC_000070.6	Pgm2	1.15E+00
gene1283	18566	NC_000067.6	Pdcd1	1.15E+00
gene40758	20641	NC_000084.6	Snrpd1	1.15E+00
gene22300	74360	NC_000075.6	Cep57	1.15E+00
gene8058	13809	NC_000069.6	Enpep	1.15E+00
gene28101	67477	NC_000077.6	Abhd15	1.15E+00
gene38621	56088	NC_000082.6	Psmg1	1.15E+00
gene1593	213006	NC_000067.6	Mfsd4a	1.15E+00
gene41772	240442	NC_000084.6	Adnp2	1.14E+00
gene5114	20612	NC_000068.7	Siglec1	1.14E+00
gene21353	56452	NC_000074.6	Orc6	1.14E+00
gene8751	24060	NC_000070.6	Slc35a1	1.14E+00
gene27598	27078	NC_000077.6	B9d1	1.14E+00
gene10836	230903	NC_000070.6	Fbxo44	1.14E+00
gene12947	231727	NC_000071.6	B3gnt4	1.14E+00
gene37161	11479	NC_000081.6	Acvr1b	1.14E+00
gene6692	73251	NC_000069.6	Setd7	1.14E+00
gene15858	76432	NC_000072.6	2310001H17Rik	1.14E+00
gene28521	18673	NC_000077.6	Phb	1.14E+00
gene16776	12273	NC_000073.6	C5ar1	1.14E+00
gene13273	69004	NC_000071.6	6330418K02Rik	1.14E+00
gene32216	108934	NC_000079.6	Smim13	1.14E+00
gene21534	74187	NC_000074.6	Katnb1	1.14E+00
gene7489	23831	NC_000069.6	Car14	1.14E+00
gene35935	15473	NC_000081.6	Rida	1.14E+00
gene14544	67138	NC_000072.6	Herc6	1.14E+00
gene7239	68603	NC_000069.6	Pmvk	1.14E+00
gene4717	1.1E+08	NC_000068.7	Gm39908	1.14E+00
gene15824	101142	NC_000072.6	Itfg2	1.14E+00
gene6848	72162	NC_000069.6	Dhx36	1.13E+00
gene13221	57330	NC_000071.6	Gigyf1	1.13E+00
gene4425	381410	NC_000068.7	Zfp408	1.13E+00
gene1640	240754	NC_000067.6	Lax1	1.13E+00

gene42227	72982	NC_000085.6	Tmem138	1.13E+00
gene2474	209456	NC_000067.6	Trp53bp2	1.13E+00
gene42567	1E+08	NC_000085.6	Gm35922	1.13E+00
gene6290	667978	NC_000069.6	Gm8910	1.13E+00
gene3794	277396	NC_000068.7	Klhl23	1.13E+00
gene4477	1E+08	NC_000068.7	Gm10804	1.13E+00
gene21298	212168	NC_000074.6	Zswim4	1.13E+00
gene2768	53893	NC_000068.7	Nudt5	1.13E+00
gene26827	216543	NC_000077.6	Cep68	1.13E+00
gene25810	72843	NC_000076.6	Prdm4	1.13E+00
gene36850	223774	NC_000081.6	Alg12	1.13E+00
gene24087	56441	NC_000075.6	Nat6	1.13E+00
gene43107	668626	NC_000085.6	Gm9276	1.13E+00
gene32217	18003	NC_000079.6	Nedd9	1.13E+00
gene20977	234366	NC_000074.6	Gatad2a	1.13E+00
gene2747	666973	NC_000068.7	Gm13176	1.13E+00
gene15362	232314	NC_000072.6	Ppp4r2	1.13E+00
gene39549	1.1E+08	NC_000083.6	Gm41575	1.13E+00
gene25815	27204	NC_000076.6	Syn3	1.13E+00
gene31683	72739	NC_000079.6	Zkscan3	1.13E+00
gene26946	78294	NC_000077.6	Rps27a	1.13E+00
gene16294	384521	NC_000073.6	Gm5319	1.13E+00
gene3232	269254	NC_000068.7	Setx	1.13E+00
gene4776	19419	NC_000068.7	Rasgrp1	1.13E+00
gene37215	246277	NC_000081.6	Csad	1.13E+00
gene15999	381823	NC_000072.6	Apold1	1.13E+00
gene12538	72392	NC_000071.6	Tmem175	1.13E+00
gene16343	654801	NC_000073.6	Zfp784	1.13E+00
gene22955	12145	NC_000075.6	Cxcr5	1.13E+00
gene36031	14368	NC_000081.6	Fzd6	1.13E+00
gene20449	320165	NC_000074.6	Tacc1	1.13E+00
gene38816	16004	NC_000083.6	Igf2r	1.13E+00
gene25036	19072	NC_000076.6	Prep	1.13E+00
gene26655	=Gene;ger	NC_000077.6	LOC108167880	1.13E+00
gene19628	67865	NC_000073.6	Rgs10	1.13E+00
gene3011	208967	NC_000068.7	Thnsl1	1.12E+00
gene29286	72344	NC_000077.6	Usp36	1.12E+00
gene30548	53314	NC_000078.6	Batf	1.12E+00
gene17652	72287	NC_000073.6	Plekhf1	1.12E+00
gene27510	216799	NC_000077.6	Nlrp3	1.12E+00
gene6918	28030	NC_000069.6	Gfm1	1.12E+00
gene29274	21877	NC_000077.6	Tk1	1.12E+00
gene18668	66365	NC_000073.6	Ccdc90b	1.12E+00
gene28338	21387	NC_000077.6	Tbx4	1.12E+00
gene9726	77809	NC_000070.6	Lrrc42	1.12E+00
gene39253	381077	NC_000083.6	Ccdc78	1.12E+00
gene23915	23873	NC_000075.6	Faim	1.12E+00
gene9895	ntaining 17;	NC_000070.6	Ccdc17	1.12E+00

gene26428	56516	NC_000076.6	Rbms2	1.12E+00
gene22149	66523	NC_000074.6	2810004N23Rik	1.12E+00
gene37	=Gene;ger	NC_000067.6	LOC108167611	1.12E+00
gene21511	1E+08	NC_000074.6	Gm38555	1.12E+00
gene40771	14465	NC_000084.6	Gata6	1.12E+00
gene37332	67824	NC_000082.6	Nmral1	1.12E+00
gene41077	66060	NC_000084.6	Cystm1	1.12E+00
gene2507	1E+08	NC_000067.6	Gm34342	1.12E+00
gene1742	59125	NC_000067.6	Nek7	1.12E+00
gene5991	19334	NC_000068.7	Rab22a	1.12E+00
gene8192	13684	NC_000069.6	Eif4e	1.12E+00
gene39358	72775	NC_000083.6	Fance	1.12E+00
gene30208	414085	NC_000078.6	9330151L19Rik	1.12E+00
gene20765	320714	NC_000074.6	Trappc11	1.12E+00
gene14057	57869	NC_000072.6	Adck2	1.12E+00
gene40668	68010	NC_000084.6	Bambi	1.12E+00
gene30347	1E+08	NC_000078.6	Gm34868	1.12E+00
gene17399	12865	NC_000073.6	Cox7a1	1.12E+00
gene8638	77032	NC_000070.6	Tstd3	1.12E+00
gene24146	80987	NC_000075.6	Nckipsd	1.12E+00
gene3926	228061	NC_000068.7	Agps	1.12E+00
gene27443	319475	NC_000077.6	Zfp672	1.12E+00
gene2160	665574	NC_000067.6	Gm7694	1.12E+00
gene23897	19659	NC_000075.6	Rbp1	1.12E+00
gene755	12912	NC_000067.6	Creb1	1.11E+00
gene29818	69675	NC_000078.6	Pxdn	1.11E+00
gene6441	208691	NC_000069.6	Eif5a2	1.11E+00
gene33334	81003	NC_000079.6	Trim23	1.11E+00
gene29163	217310	NC_000077.6	Hid1	1.11E+00
gene42117	13728	NC_000085.6	Mark2	1.11E+00
gene29213	217335	NC_000077.6	Fbf1	1.11E+00
gene3266	241296	NC_000068.7	Lrrc8a	1.11E+00
gene11543	74254	NC_000071.6	Gpn1	1.11E+00
gene38649	114565	NC_000082.6	Zbtb21	1.11E+00
gene11893	79464	NC_000071.6	Lias	1.11E+00
gene10415	194231	NC_000070.6	Cnksr1	1.11E+00
gene17572	68079	NC_000073.6	Pdcd2l	1.11E+00
gene30181	328110	NC_000078.6	Prpf39	1.11E+00
gene21705	319446	NC_000074.6	Dpep2	1.11E+00
gene24145	76500	NC_000075.6	Ip6k2	1.11E+00
gene28703	94179	NC_000077.6	Krt23	1.11E+00
gene20961	234362	NC_000074.6	Zfp868	1.11E+00
gene3002	1E+08	NC_000068.7	Gm13361	1.11E+00
gene34766	17888	NC_000080.6	Myh6	1.11E+00
gene33453	238896	NC_000079.6	Cdc20b	1.11E+00
gene10403	15331	NC_000070.6	Hmgn2	1.11E+00
gene25230	15275	NC_000076.6	Hk1	1.11E+00
gene21890	75465	NC_000074.6	Dynlrb2	1.11E+00

gene28214	1E+08	NC_000077.6	Gm31228	1.11E+00
gene25513	216131	NC_000076.6	Trappc10	1.11E+00
gene21796	234728	NC_000074.6	Cmtr2	1.11E+00
gene26103	13008	NC_000076.6	Csrp2	1.11E+00
gene39336	15361	NC_000083.6	Hmga1	1.11E+00
gene12679	53890	NC_000071.6	Sart3	1.11E+00
gene37643	381038	NC_000082.6	Parl	1.11E+00
gene12428	18764	NC_000071.6	Pkd2	1.11E+00
gene10933	70025	NC_000070.6	Acot7	1.11E+00
gene32148	68404	NC_000079.6	Nrn1	1.11E+00
gene42284	20908	NC_000085.6	Stx3	1.11E+00
gene15513	330401	NC_000072.6	Tmcc1	1.11E+00
gene1842	98685	NC_000067.6	Trmt1l	1.11E+00
gene29474	14226	NC_000078.6	Fkbp1b	1.10E+00
gene22649	1.1E+08	NC_000075.6	Gm39318	1.10E+00
gene41527	107035	NC_000084.6	Fbxo38	1.10E+00
gene27061	211652	NC_000077.6	Wwc1	1.10E+00
gene42483	14544	NC_000085.6	Gda	1.10E+00
gene40523	=Gene;ger	NC_000083.6	LOC108168338	1.10E+00
gene12877	231713	NC_000071.6	Naa25	1.10E+00
gene20660	434304	NC_000074.6	Gm5609	1.10E+00
gene4887	51885	NC_000068.7	Tubgcp4	1.10E+00
gene38908	12648	NC_000083.6	Chd1	1.10E+00
gene19998	79196	NC_000073.6	Osbpl5	1.10E+00
gene19589	13019	NC_000073.6	Ctf1	1.10E+00
gene6129	228983	NC_000068.7	Osbpl2	1.10E+00
gene19484	233863	NC_000073.6	Gtf3c1	1.10E+00
gene28312	192970	NC_000077.6	Dhrs11	1.10E+00
gene36651	20524	NC_000081.6	Slc25a17	1.10E+00
gene32687	14120	NC_000079.6	Fbp2	1.10E+00
gene38600	1E+08	NC_000082.6	Gm31323	1.10E+00
gene35326	13709	NC_000080.6	Elf1	1.10E+00
gene27808	216856	NC_000077.6	Nlgn2	1.10E+00
gene21164	70345	NC_000074.6	0610038B21Rik	1.10E+00
gene10232	242669	NC_000070.6	Azin2	1.10E+00
gene10294	66585	NC_000070.6	Snrnp40	1.10E+00
gene5613	19650	NC_000068.7	Rbl1	1.10E+00
gene1838	=Gene;ger	NC_000067.6	LOC108167732	1.10E+00
gene3314	227715	NC_000068.7	Exosc2	1.10E+00
gene5184	52837	NC_000068.7	Tmx4	1.10E+00
gene25568	216151	NC_000076.6	Polrmt	1.10E+00
gene119	17681	NC_000067.6	Msc	1.09E+00
gene9900	67096	NC_000070.6	Mmachc	1.09E+00
gene27952	83429	NC_000077.6	Ctns	1.09E+00
gene36954	266632	NC_000081.6	Irak4	1.09E+00
gene10575	212647	NC_000070.6	Aldh4a1	1.09E+00
gene8933	433700	NC_000070.6	Spag8	1.09E+00
gene10833	71890	NC_000070.6	Mad2l2	1.09E+00

gene17800	259277	NC_000073.6	Klk8	1.09E+00
gene15089	69786	NC_000072.6	Tprkb	1.09E+00
gene29338	78777	NC_000077.6	Enthd2	1.09E+00
gene42212	56473	NC_000085.6	Fads2	1.09E+00
gene1844	19821	NC_000067.6	Rnf2	1.09E+00
gene39595	81701	NC_000083.6	Egfl8	1.09E+00
gene13301	73212	NC_000071.6	3110082I17Rik	1.09E+00
gene15839	17059	NC_000072.6	Klrb1c	1.09E+00
gene4936	214616	NC_000068.7	Spata5l1	1.09E+00
gene38597	56176	NC_000082.6	Pigp	1.09E+00
gene12951	56430	NC_000071.6	Clip1	1.09E+00
gene33488	109700	NC_000079.6	Itga1	1.09E+00
gene34750	75913	NC_000080.6	4930579G18Rik	1.09E+00
gene23091	1E+08	NC_000075.6	Gm38476	1.09E+00
gene12338	320292	NC_000071.6	Rasgef1b	1.09E+00
gene4589	241593	NC_000068.7	Pin1rt1	1.09E+00
gene28602	192157	NC_000077.6	Socs7	1.09E+00
gene23416	70784	NC_000075.6	Rasl12	1.09E+00
gene43010	27360	NC_000085.6	Add3	1.09E+00
gene4862	211499	NC_000068.7	Tmem87a	1.09E+00
gene37329	78885	NC_000082.6	Coro7	1.09E+00
gene16898	232944	NC_000073.6	Mark4	1.09E+00
gene3238	107746	NC_000068.7	Rapgef1	1.09E+00
gene40296	70351	NC_000083.6	Ppp4r1	1.09E+00
gene11534	67661	NC_000071.6	Ift172	1.09E+00
gene2303	236312	NC_000067.6	Pyhin1	1.08E+00
gene25885	67454	NC_000076.6	Ikbip	1.08E+00
gene23909	546155	NC_000075.6	Gm5920	1.08E+00
gene6688	74838	NC_000069.6	Naa15	1.08E+00
gene5645	228850	NC_000068.7	Ralgapb	1.08E+00
gene29802	19819	NC_000078.6	Rnaseh1	1.08E+00
gene30027	94186	NC_000078.6	Strn3	1.08E+00
gene8543	66185	NC_000070.6	1110037F02Rik	1.08E+00
gene15497	67027	NC_000072.6	Mkrn2	1.08E+00
gene23984	235559	NC_000075.6	Topbp1	1.08E+00
gene24456	66446	NC_000075.6	Exosc7	1.08E+00
gene40458	75292	NC_000083.6	Prkd3	1.08E+00
gene9893	77110	NC_000070.6	Gpbp1l1	1.08E+00
gene41420	73137	NC_000084.6	Prrc1	1.08E+00
gene9673	665451	NC_000070.6	Gm12716	1.08E+00
gene21615	234664	NC_000074.6	Nae1	1.08E+00
gene5040	72477	NC_000068.7	Tmem87b	1.08E+00
gene24099	72825	NC_000075.6	Mon1a	1.08E+00
gene5291	74030	NC_000068.7	Rin2	1.08E+00
gene12515	1E+08	NC_000071.6	Rpl5	1.08E+00
gene15308	101351	NC_000072.6	Eogt	1.08E+00
gene26627	18413	NC_000077.6	Osm	1.08E+00
gene25572	73106	NC_000076.6	Prss57	1.08E+00

gene36936	223827	NC_000081.6	Gxylt1	1.08E+00
gene13213	83704	NC_000071.6	Slc12a9	1.08E+00
gene19421	12585	NC_000073.6	Cdr2	1.08E+00
gene12221	231452	NC_000071.6	Sdad1	1.08E+00
gene3078	68846	NC_000068.7	Rnf208	1.08E+00
gene2170	98752	NC_000067.6	Fcrla	1.07E+00
gene30752	238377	NC_000078.6	Gpr68	1.07E+00
gene31696	93681	NC_000079.6	Zkscan8	1.07E+00
gene7557	69870	NC_000069.6	Polr3gl	1.07E+00
gene26595	1E+08	NC_000077.6	Gm11946	1.07E+00
gene32497	51791	NC_000079.6	Rgs14	1.07E+00
gene25765	319277	NC_000076.6	A230046K03Rik	1.07E+00
gene30509	12499	NC_000078.6	Entpd5	1.07E+00
gene37168	77717	NC_000081.6	6030408B16Rik	1.07E+00
gene33711	286940	NC_000080.6	Flnb	1.07E+00
gene43102	1.1E+08	NC_000085.6	Gm41877	1.07E+00
gene40562	213760	NC_000083.6	Prepl	1.07E+00
gene12934	208043	NC_000071.6	Setd1b	1.07E+00
gene3722	1.1E+08	NC_000068.7	Gm39839	1.07E+00
gene24922	667964	NC_000076.6	Gm8899	1.07E+00
gene29381	68938	NC_000077.6	Aspscr1	1.07E+00
gene6132	16776	NC_000068.7	Lama5	1.07E+00
gene32291	218214	NC_000079.6	Kdm1b	1.07E+00
gene24784	13822	NC_000076.6	Epb41l2	1.07E+00
gene1564	69169	NC_000067.6	Fcmr	1.07E+00
gene27836	216864	NC_000077.6	Mgl2	1.07E+00
gene33263	20595	NC_000079.6	Smn1	1.07E+00
gene25576	414101	NC_000076.6	E130317F20Rik	1.07E+00
gene980	20720	NC_000067.6	Serpine2	1.07E+00
gene16103	232491	NC_000072.6	Pyroxd1	1.07E+00
gene29411	68837	NC_000077.6	Foxk2	1.07E+00
gene21698	244631	NC_000074.6	Pskh1	1.07E+00
gene23591	235497	NC_000075.6	Leo1	1.07E+00
gene40799	16210	NC_000084.6	Impact	1.07E+00
gene29194	76265	NC_000077.6	Tsen54	1.07E+00
gene30345	108156	NC_000078.6	Mthfd1	1.07E+00
gene6540	229211	NC_000069.6	Acad9	1.07E+00
gene2092	226594	NC_000067.6	Rcsd1	1.07E+00
gene22485	59035	NC_000075.6	Carm1	1.07E+00
gene38061	67896	NC_000082.6	Ccdc80	1.07E+00
gene5421	69698	NC_000068.7	Slc52a3	1.07E+00
gene13077	212919	NC_000071.6	Kctd7	1.07E+00
gene28130	268449	NC_000077.6	Rpl23a	1.07E+00
gene11427	1E+08	NC_000071.6	Gm18957	1.07E+00
gene25284	28193	NC_000076.6	Reep3	1.07E+00
gene19890	27388	NC_000073.6	Ptdss2	1.07E+00
gene1592	13714	NC_000067.6	Elk4	1.06E+00
gene2596	1E+08	NC_000067.6	A230020J21Rik	1.06E+00

gene42272	66607	NC_000085.6	Ms4a4d	1.06E+00
gene34121	105450	NC_000080.6	Mmrn2	1.06E+00
gene38533	246738	NC_000082.6	Dnajc28	1.06E+00
gene42167	225895	NC_000085.6	Taf6l	1.06E+00
gene14177	13835	NC_000072.6	Epha1	1.06E+00
gene27611	1.1E+08	NC_000077.6	Gm39985	1.06E+00
gene5147	54338	NC_000068.7	Slc23a2	1.06E+00
gene10341	100088	NC_000070.6	Rcc1	1.06E+00
gene9077	1E+08	NC_000070.6	Amd-ps4	1.06E+00
gene1864	17970	NC_000067.6	Ncf2	1.06E+00
gene3401	66691	NC_000068.7	Gapvd1	1.06E+00
gene28845	17285	NC_000077.6	Meox1	1.06E+00
gene33825	434616	NC_000080.6	Gm5630	1.06E+00
gene13662	213819	NC_000072.6	Casd1	1.06E+00
gene36591	27373	NC_000081.6	Csnk1e	1.06E+00
gene14067	243780	NC_000072.6	E330009J07Rik	1.06E+00
gene27890	216877	NC_000077.6	Dhx33	1.06E+00
gene5131	228608	NC_000068.7	Smox	1.06E+00
gene41032	277250	NC_000084.6	Kdm3b	1.06E+00
gene7567	78373	NC_000069.6	Nudt17	1.06E+00
gene38407	53858	NC_000082.6	Rwdd2b	1.06E+00
gene5023	12125	NC_000068.7	Bcl2l11	1.06E+00
gene40150	72886	NC_000083.6	Ccdc94	1.06E+00
gene3255	263803	NC_000068.7	Pkn3	1.06E+00
gene19921	68038	NC_000073.6	Chid1	1.06E+00
gene4739	=Gene;ger	NC_000068.7	LOC108168784	1.06E+00
gene18278	233328	NC_000073.6	Lrrk1	1.06E+00
gene33442	1E+08	NC_000079.6	Gm3226	1.06E+00
gene28118	70451	NC_000077.6	Dhrs13	1.06E+00
gene40206	64144	NC_000083.6	Mllt1	1.06E+00
gene5377	68738	NC_000068.7	Acss1	1.06E+00
gene14836	620497	NC_000072.6	Gm6157	1.05E+00
gene14490	68235	NC_000072.6	Mturn	1.05E+00
gene14561	626672	NC_000072.6	Gm6695	1.05E+00
gene35608	105559	NC_000080.6	Mbnl2	1.05E+00
gene9115	52076	NC_000070.6	Tmem38b	1.05E+00
gene21076	434325	NC_000074.6	Tmem221	1.05E+00
gene31031	26931	NC_000078.6	Ppp2r5c	1.05E+00
gene2031	226562	NC_000067.6	Prrc2c	1.05E+00
gene20198	234069	NC_000074.6	Pcid2	1.05E+00
gene25147	70297	NC_000076.6	Gcc2	1.05E+00
gene20694	14300	NC_000074.6	Frg1	1.05E+00
gene35072	19229	NC_000080.6	Ptk2b	1.05E+00
gene7132	73182	NC_000069.6	Pear1	1.05E+00
gene17889	75736	NC_000073.6	Bcl2l12	1.05E+00
gene17380	233056	NC_000073.6	Zfp790	1.05E+00
gene8749	50793	NC_000070.6	Orc3	1.05E+00
gene17787	210105	NC_000073.6	Zfp719	1.05E+00

gene1244	64095	NC_000067.6	Gpr35	1.05E+00
gene11028	140500	NC_000070.6	Acap3	1.05E+00
gene26706	216527	NC_000077.6	Ccm2	1.05E+00
gene30326	671971	NC_000078.6	Gm9544	1.05E+00
gene2052	240880	NC_000067.6	Scyl3	1.05E+00
gene708	54167	NC_000067.6	Icos	1.05E+00
gene5583	70873	NC_000068.7	Cnbd2	1.05E+00
gene27521	1E+08	NC_000077.6	Gm16062	1.05E+00
gene22430	235028	NC_000075.6	Zfp426	1.05E+00
gene42649	381236	NC_000085.6	Lipo3	1.05E+00
gene8705	26409	NC_000070.6	Map3k7	1.05E+00
gene9915	78611	NC_000070.6	Btbd19	1.05E+00
gene3267	227696	NC_000068.7	Phyhd1	1.05E+00
gene23952	66904	NC_000075.6	Pccb	1.05E+00
gene12212	ene;gene=l	NC_000071.6	LOC108169096	1.05E+00
gene30520	16997	NC_000078.6	Ltbp2	1.04E+00
gene9712	72354	NC_000070.6	Ttc4	1.04E+00
gene22025	50917	NC_000074.6	Galns	1.04E+00
gene13323	17766	NC_000071.6	Nudt1	1.04E+00
gene8422	433667	NC_000069.6	Ankrd13c	1.04E+00
gene9102	11303	NC_000070.6	Abca1	1.04E+00
gene13412	55950	NC_000071.6	Bri3	1.04E+00
gene13547	231915	NC_000071.6	Uspl1	1.04E+00
gene27246	103806	NC_000077.6	Maml1	1.04E+00
gene18292	17258	NC_000073.6	Mef2a	1.04E+00
gene39473	22404	NC_000083.6	Wiz	1.04E+00
gene18255	73724	NC_000073.6	Mcee	1.04E+00
gene3302	14269	NC_000068.7	Fnbp1	1.04E+00
gene10418	72690	NC_000070.6	Grrp1	1.04E+00
gene26649	11764	NC_000077.6	Ap1b1	1.04E+00
gene18726	244152	NC_000073.6	Tsku	1.04E+00
gene9553	21687	NC_000070.6	Tek	1.04E+00
gene4028	1E+08	NC_000068.7	Gm32289	1.04E+00
gene22239	12364	NC_000075.6	Casp12	1.04E+00
gene10466	12802	NC_000070.6	Cnr2	1.04E+00
gene9876	14073	NC_000070.6	Faah	1.04E+00
gene3844	635960	NC_000068.7	Ak3l2-ps	1.04E+00
gene41022	78656	NC_000084.6	Brd8	1.04E+00
gene22141	234875	NC_000074.6	Ttc13	1.04E+00
gene35144	71843	NC_000080.6	R3hcc1	1.04E+00
gene30911	52690	NC_000078.6	Setd3	1.04E+00
gene5784	228869	NC_000068.7	Ncoa5	1.04E+00
gene42515	21873	NC_000085.6	Tjp2	1.04E+00
gene23209	=Gene;ger	NC_000075.6	LOC108167365	1.04E+00
gene3717	13482	NC_000068.7	Dpp4	1.04E+00
gene26588	193670	NC_000077.6	Rnf185	1.04E+00
gene3209	56177	NC_000068.7	Olfm1	1.04E+00
gene27136	11492	NC_000077.6	Adam19	1.04E+00

gene10140	194268	NC_000070.6	9930104L06Rik	1.04E+00
gene19437	20277	NC_000073.6	Scnn1b	1.04E+00
gene26001	67972	NC_000076.6	Atp2b1	1.04E+00
gene35633	321019	NC_000080.6	Gpr183	1.04E+00
gene18493	434204	NC_000073.6	Whamm	1.04E+00
gene3042	215257	NC_000068.7	Il1f9	1.04E+00
gene15442	72895	NC_000072.6	Setd5	1.04E+00
gene24278	1E+08	NC_000075.6	Gm30254	1.03E+00
gene39265	68241	NC_000083.6	Fam195a	1.03E+00
gene38931	67045	NC_000083.6	Riok2	1.03E+00
gene15014	20166	NC_000072.6	Rtkn	1.03E+00
gene27014	103583	NC_000077.6	Fbxw11	1.03E+00
gene36314	383051	NC_000081.6	Gm5217	1.03E+00
gene13365	1E+08	NC_000071.6	Gm31365	1.03E+00
gene39571	15002	NC_000083.6	H2-Ob	1.03E+00
gene27357	18452	NC_000077.6	P4ha2	1.03E+00
gene38259	19128	NC_000082.6	Pros1	1.03E+00
gene11789	242987	NC_000071.6	Gm4962	1.03E+00
gene21841	66817	NC_000074.6	Tmem170	1.03E+00
gene6152	1E+08	NC_000068.7	Gm30345	1.03E+00
gene35803	77877	NC_000081.6	6030458C11Rik	1.03E+00
gene28464	70834	NC_000077.6	Spag9	1.03E+00
gene23207	20466	NC_000075.6	Sin3a	1.03E+00
gene24283	235661	NC_000075.6	Dync1li1	1.03E+00
gene4447	19099	NC_000068.7	Mapk8ip1	1.03E+00
gene25439	67523	NC_000076.6	1700094J05Rik	1.03E+00
gene11919	77569	NC_000071.6	Limch1	1.03E+00
gene32327	18676	NC_000079.6	Phf2	1.03E+00
gene30807	217847	NC_000078.6	Serpina10	1.03E+00
gene20159	102334	NC_000074.6	Ankrd10	1.03E+00
gene24155	270198	NC_000075.6	Pfkfb4	1.03E+00
gene40969	74320	NC_000084.6	Wdr33	1.03E+00
gene36035	69906	NC_000081.6	Slc25a32	1.03E+00
gene28205	70591	NC_000077.6	5730455P16Rik	1.03E+00
gene41732	76987	NC_000084.6	Hdhd2	1.03E+00
gene32129	23986	NC_000079.6	Eci2	1.03E+00
gene38561	54720	NC_000082.6	Rcan1	1.03E+00
gene26366	216439	NC_000076.6	Agap2	1.03E+00
gene3067	241275	NC_000068.7	Noxa1	1.03E+00
gene17451	73712	NC_000073.6	Dmkn	1.03E+00
gene36968	667849	NC_000081.6	Gm8843	1.03E+00
gene28353	75909	NC_000077.6	Vmp1	1.03E+00
gene9740	1E+08	NC_000070.6	Gm12907	1.03E+00
gene39717	75210	NC_000083.6	Prr3	1.03E+00
gene42666	14102	NC_000085.6	Fas	1.02E+00
gene26624	103724	NC_000077.6	Tbc1d10a	1.02E+00
gene13518	71706	NC_000071.6	Slc46a3	1.02E+00
gene23431	68026	NC_000075.6	2810417H13Rik	1.02E+00

gene33264	17948	NC_000079.6	Naip2	1.02E+00
gene21923	56690	NC_000074.6	Mlycd	1.02E+00
gene29849	12033	NC_000078.6	Bcap29	1.02E+00
gene3692	17076	NC_000068.7	Ly75	1.02E+00
gene45	319263	NC_000067.6	Pcmtd1	1.02E+00
gene4847	68925	NC_000068.7	Rpap1	1.02E+00
gene6315	71934	NC_000069.6	Car13	1.02E+00
gene16780	56459	NC_000073.6	Sae1	1.02E+00
gene7734	1.1E+08	NC_000069.6	Gm40116	1.02E+00
gene7846	229731	NC_000069.6	Slc25a24	1.02E+00
gene231	68421	NC_000067.6	Lmbrd1	1.02E+00
gene41893	16973	NC_000085.6	Lrp5	1.02E+00
gene39620	70129	NC_000083.6	Slc44a4	1.02E+00
gene41711	17131	NC_000084.6	Smad7	1.02E+00
gene3559	24136	NC_000068.7	Zeb2	1.02E+00
gene1243	23830	NC_000067.6	Capn10	1.02E+00
gene12457	433926	NC_000071.6	Lrrc8b	1.02E+00
gene42079	19395	NC_000085.6	Rasgrp2	1.02E+00
gene15016	71837	NC_000072.6	1700003E16Rik	1.02E+00
gene8928	1.1E+08	NC_000070.6	Gm42278	1.02E+00
gene14482	78004	NC_000072.6	Prr15	1.02E+00
gene7195	229521	NC_000069.6	Syt11	1.02E+00
gene40374	72515	NC_000083.6	Wdr43	1.02E+00
gene37898	16419	NC_000082.6	Itgb5	1.02E+00
gene31536	664862	NC_000079.6	Gpr137b-ps	1.02E+00
gene33159	328309	NC_000079.6	Gm9776	1.02E+00
gene36006	70790	NC_000081.6	Ubr5	1.02E+00
gene33320	432800	NC_000079.6	Gm5454	1.02E+00
gene30508	217707	NC_000078.6	Coq6	1.02E+00
gene23975	20187	NC_000075.6	Ryk	1.02E+00
gene17713	1E+08	NC_000073.6	Gm17791	1.02E+00
gene28865	268490	NC_000077.6	Lsm12	1.02E+00
gene42965	66477	NC_000085.6	Usmg5	1.02E+00
gene16873	20475	NC_000073.6	Six5	1.02E+00
gene42545	67155	NC_000085.6	Smarca2	1.01E+00
gene756	67099	NC_000067.6	Mettl21a	1.01E+00
gene18713	=Gene;ger	NC_000073.6	LOC102641859	1.01E+00
gene36610	109361	NC_000081.6	D730005E14Rik	1.01E+00
gene10973	230972	NC_000070.6	Arhgef16	1.01E+00
gene29255	53860	NC_000077.6	sep-09	1.01E+00
gene19282	57342	NC_000073.6	Parva	1.01E+00
gene41611	107047	NC_000084.6	Psmg2	1.01E+00
gene27282	12750	NC_000077.6	Clk4	1.01E+00
gene1769	12628	NC_000067.6	Cfh	1.01E+00
gene39679	667669	NC_000083.6	Gm8752	1.01E+00
gene33554	625360	NC_000079.6	BC147527	1.01E+00
gene29200	57230	NC_000077.6	Sap30bp	1.01E+00
gene29456	52504	NC_000078.6	Cenpo	1.01E+00

gene33221	68558	NC_000079.6	Ankra2	1.01E+00
gene26582	74203	NC_000077.6	Eif4enif1	1.01E+00
gene42503	1.1E+08	NC_000085.6	Gm41821	1.01E+00
gene37411	223970	NC_000082.6	Rmi2	1.01E+00
gene24515	83397	NC_000076.6	Akap12	1.01E+00
gene40722	225131	NC_000084.6	Wac	1.01E+00
gene28811	12777	NC_000077.6	Ccr10	1.01E+00
gene10347	230784	NC_000070.6	Sesn2	1.01E+00
gene33873	22330	NC_000080.6	Vcl	1.01E+00
gene27493	216792	NC_000077.6	Iba57	1.01E+00
gene13914	76788	NC_000072.6	Klhdc10	1.01E+00
gene42203	16319	NC_000085.6	Incenp	1.01E+00
gene28459	217109	NC_000077.6	Utp18	1.01E+00
gene13876	68272	NC_000072.6	Rbm28	1.01E+00
gene33976	72993	NC_000080.6	Appl1	1.01E+00
gene37011	223864	NC_000081.6	Rapgef3	1.01E+00
gene8558	77604	NC_000070.6	Rbm12b2	1.01E+00
gene25685	21390	NC_000076.6	Tbxa2r	1.01E+00
gene679	22218	NC_000067.6	Sumo1	1.01E+00
gene12015	16590	NC_000071.6	Kit	1.01E+00
gene31488	69617	NC_000079.6	Pitrm1	1.01E+00
gene7712	18685	NC_000069.6	Phtf1	1.01E+00
gene42040	18969	NC_000085.6	Pola2	1.01E+00
gene38898	21374	NC_000083.6	Tbp	1.01E+00
gene1757	226470	NC_000067.6	Zbtb41	1.01E+00
gene8835	73721	NC_000070.6	1110017D15Rik	1.01E+00
gene41057	71242	NC_000084.6	Spata24	1.00E+00
gene41013	11789	NC_000084.6	Apc	1.00E+00
gene10647	=Gene;ger	NC_000070.6	LOC108168984	1.00E+00
gene40016	14913	NC_000083.6	Guca1a	1.00E+00
gene11896	53323	NC_000071.6	Ube2k	1.00E+00
gene38828	1E+08	NC_000083.6	Gm20591	1.00E+00
gene7201	65111	NC_000069.6	Dap3	1.00E+00
gene7807	14865	NC_000069.6	Gstm4	1.00E+00
gene4872	=Gene;ger	NC_000068.7	LOC108168839	1.00E+00
gene20672	52348	NC_000074.6	Vps37a	1.00E+00
gene32983	192657	NC_000079.6	Ell2	1.00E+00
gene37679	13845	NC_000082.6	Ephb3	1.00E+00
gene18798	72590	NC_000073.6	Ppme1	1.00E+00
gene29066	104681	NC_000077.6	Slc16a6	1.00E+00
gene29182	260302	NC_000077.6	Gga3	1.00E+00
gene23304	72565	NC_000075.6	Uaca	1.00E+00
gene36801	54138	NC_000081.6	Atxn10	1.00E+00
gene11514	330064	NC_000071.6	Slc5a6	1.00E+00
gene20473	52120	NC_000074.6	Hgsnat	1.00E+00
gene35161	268759	NC_000080.6	9930012K11Rik	1.00E+00
gene12859	246728	NC_000071.6	Oas2	1.00E+00
gene38624	71446	NC_000082.6	Wrb	1.00E+00

gene4963	271849	NC_000068.7	Shc4	9.99E-01
gene11946	231279	NC_000071.6	Guf1	9.99E-01
gene4731	228482	NC_000068.7	Arhgap11a	9.99E-01
gene33154	107767	NC_000079.6	Scamp1	9.98E-01
gene24073	69536	NC_000075.6	Hemk1	9.98E-01
gene8342	170757	NC_000069.6	Adgrl4	9.98E-01
gene24551	23924	NC_000076.6	Katna1	9.97E-01
gene19518	75764	NC_000073.6	Slx1b	9.97E-01
gene39660	18038	NC_000083.6	Nfkbil1	9.97E-01
gene24081	114602	NC_000075.6	Zmynd10	9.97E-01
gene8916	329831	NC_000070.6	Fam166b	9.97E-01
gene31395	52635	NC_000078.6	Esyt2	9.97E-01
gene2013	226551	NC_000067.6	Suco	9.97E-01
gene33171	72114	NC_000079.6	Zbed3	9.96E-01
gene16905	63872	NC_000073.6	Zfp296	9.95E-01
gene17939	71755	NC_000073.6	Dhdh	9.94E-01
gene22059	57247	NC_000074.6	Zfp276	9.94E-01
gene28908	69739	NC_000077.6	2410004I01Rik	9.93E-01
gene27253	216724	NC_000077.6	Rufy1	9.93E-01
gene13425	75788	NC_000071.6	Smurf1	9.93E-01
gene8482	18201	NC_000070.6	Nsmaf	9.92E-01
gene25873	213326	NC_000076.6	Scyl2	9.92E-01
gene12054	19712	NC_000071.6	Rest	9.91E-01
gene32841	1E+08	NC_000079.6	Zfp729b	9.91E-01
gene37127	207214	NC_000081.6	Larp4	9.91E-01
gene39210	106633	NC_000083.6	Ift140	9.90E-01
gene36696	56448	NC_000081.6	Cyp2d22	9.90E-01
gene13781	17295	NC_000072.6	Met	9.90E-01
gene34910	71891	NC_000080.6	Cdadc1	9.90E-01
gene32207	=Gene;ger	NC_000079.6	LOC102633880	9.89E-01
gene39371	20815	NC_000083.6	Srpk1	9.89E-01
gene30918	1E+08	NC_000078.6	Gm16596	9.89E-01
gene25084	94221	NC_000076.6	Gopc	9.89E-01
gene19534	68742	NC_000073.6	Tmem219	9.88E-01
gene21274	66929	NC_000074.6	Asf1b	9.88E-01
gene21510	234577	NC_000074.6	Cpne2	9.87E-01
gene20466	67384	NC_000074.6	Bag4	9.87E-01
gene35453	1.1E+08	NC_000080.6	Gm41235	9.87E-01
gene39991	71461	NC_000083.6	Ptk7	9.86E-01
gene2012	14103	NC_000067.6	Fasl	9.86E-01
gene33303	18708	NC_000079.6	Pik3r1	9.86E-01
gene23901	64655	NC_000075.6	Mrps22	9.85E-01
gene3853	1E+08	NC_000068.7	Sp3os	9.85E-01
gene41233	107045	NC_000084.6	Lars	9.85E-01
gene12240	52398	NC_000071.6	sep-11	9.85E-01
gene27356	30794	NC_000077.6	Pdlim4	9.85E-01
gene25573	18483	NC_000076.6	Palm	9.84E-01
gene8754	66291	NC_000070.6	Smim8	9.84E-01

gene17418	243910	NC_000073.6	Nfkbid	9.84E-01
gene4399	13046	NC_000068.7	Celf1	9.84E-01
gene20918	66282	NC_000074.6	Tma16	9.83E-01
gene35762	78244	NC_000081.6	Dnajc21	9.83E-01
gene8034	28036	NC_000069.6	Larp7	9.82E-01
gene34122	12166	NC_000080.6	Bmpr1a	9.82E-01
gene42547	70942	NC_000085.6	4931403E22Rik	9.82E-01
gene35004	14137	NC_000080.6	Fdft1	9.81E-01
gene27626	216829	NC_000077.6	Mmgt2	9.81E-01
gene36458	15499	NC_000081.6	Hsf1	9.81E-01
gene3204	20181	NC_000068.7	Rxra	9.81E-01
gene25245	67500	NC_000076.6	Ccar1	9.81E-01
gene29271	217356	NC_000077.6	Tmc8	9.80E-01
gene17254	76889	NC_000073.6	Adck4	9.80E-01
gene38528	665939	NC_000082.6	Gm7856	9.80E-01
gene13297	231821	NC_000071.6	Adap1	9.80E-01
gene3366	20910	NC_000068.7	Stxbp1	9.80E-01
gene7792	1.1E+08	NC_000069.6	Gm40121	9.80E-01
gene29466	20403	NC_000078.6	Itsn2	9.80E-01
gene21546	13000	NC_000074.6	Csnk2a2	9.77E-01
gene26152	66687	NC_000076.6	Tbc1d15	9.77E-01
gene1573	54354	NC_000067.6	Rassf5	9.77E-01
gene41902	12660	NC_000085.6	Chka	9.76E-01
gene27945	66874	NC_000077.6	Ncbp3	9.76E-01
gene22896	272589	NC_000075.6	Tbcel	9.76E-01
gene42818	72199	NC_000085.6	Mms19	9.75E-01
gene20148	384783	NC_000074.6	Irs2	9.75E-01
gene8542	634834	NC_000070.6	Gm11821	9.75E-01
gene27	108664	NC_000067.6	Atp6v1h	9.75E-01
gene35123	67712	NC_000080.6	Slc25a37	9.75E-01
gene42868	226144	NC_000085.6	Erlin1	9.74E-01
gene34733	20540	NC_000080.6	Slc7a7	9.74E-01
gene37663	78408	NC_000082.6	Fam131a	9.74E-01
gene3104	69379	NC_000068.7	C8g	9.74E-01
gene30912	12454	NC_000078.6	Ccnk	9.72E-01
gene31531	217995	NC_000079.6	Heatr1	9.72E-01
gene9	664830	NC_000067.6	Gm7357	9.72E-01
gene4409	228355	NC_000068.7	Madd	9.71E-01
gene8090	545568	NC_000069.6	Gm5855	9.70E-01
gene34289	19217	NC_000080.6	Ptger2	9.70E-01
gene6495	74600	NC_000069.6	Mrpl47	9.70E-01
gene11883	21899	NC_000071.6	Tlr6	9.70E-01
gene24834	212813	NC_000076.6	Gm232	9.70E-01
gene40814	268996	NC_000084.6	Ss18	9.69E-01
gene6532	76295	NC_000069.6	Atp11b	9.69E-01
gene9171	242484	NC_000070.6	D630039A03Rik	9.69E-01
gene42488	83921	NC_000085.6	Tmem2	9.69E-01
gene11991	69727	NC_000071.6	Usp46	9.68E-01

gene36454	223658	NC_000081.6	Mroh1	9.68E-01
gene36087	55960	NC_000081.6	Ebag9	9.68E-01
gene3257	227693	NC_000068.7	Zer1	9.68E-01
gene42556	52874	NC_000085.6	Pum3	9.67E-01
gene40734	67974	NC_000084.6	Ccny	9.67E-01
gene6857	17380	NC_000069.6	Mme	9.66E-01
gene464	73122	NC_000067.6	Tgfbrap1	9.66E-01
gene12056	56412	NC_000071.6	Noa1	9.66E-01
gene9943	53418	NC_000070.6	B4galt2	9.65E-01
gene20225	66423	NC_000074.6	Coprs	9.65E-01
gene34329	68755	NC_000080.6	Cgrrf1	9.65E-01
gene41890	52036	NC_000085.6	Ppp6r3	9.65E-01
gene16646	232878	NC_000073.6	Zscan22	9.64E-01
gene13364	231861	NC_000071.6	Tnrc18	9.62E-01
gene21361	71927	NC_000074.6	Itfg1	9.61E-01
gene30835	628883	NC_000078.6	Serpina3e-ps	9.61E-01
gene38425	54420	NC_000082.6	Cldn8	9.60E-01
gene20474	74653	NC_000074.6	Pomk	9.60E-01
gene40713	664778	NC_000084.6	Gm10350	9.59E-01
gene23851	76477	NC_000075.6	Pcolce2	9.59E-01
gene6667	73173	NC_000069.6	Pcdh18	9.59E-01
gene10381	100342	NC_000070.6	Fam46b	9.58E-01
gene13486	19933	NC_000071.6	Rpl21	9.58E-01
gene30320	75627	NC_000078.6	Snapc1	9.58E-01
gene21492	270086	NC_000074.6	Ogfod1	9.58E-01
gene41274	70640	NC_000084.6	Dcp2	9.58E-01
gene21955	272551	NC_000074.6	Gins2	9.58E-01
gene28831	11858	NC_000077.6	Rnd2	9.57E-01
gene21508	434341	NC_000074.6	Nlrc5	9.57E-01
gene15503	67088	NC_000072.6	Cand2	9.56E-01
gene13844	73178	NC_000072.6	Wasl	9.56E-01
gene38243	67014	NC_000082.6	Mina	9.56E-01
gene35168	213053	NC_000080.6	Slc39a14	9.55E-01
gene27572	216820	NC_000077.6	Dhrs7b	9.55E-01
gene12000	66899	NC_000071.6	Fip1l1	9.55E-01
gene22998	235315	NC_000075.6	Rnf214	9.55E-01
gene23396	69882	NC_000075.6	Vwa9	9.54E-01
gene10867	63954	NC_000070.6	Rbp7	9.54E-01
gene29629	53602	NC_000078.6	Hpcal1	9.54E-01
gene24963	268294	NC_000076.6	Zbtb24	9.53E-01
gene24019	235574	NC_000075.6	Atp2c1	9.53E-01
gene3832	228026	NC_000068.7	Pdk1	9.53E-01
gene17569	101543	NC_000073.6	Wtip	9.53E-01
gene15382	384470	NC_000072.6	Gm5315	9.52E-01
gene12295	231464	NC_000071.6	Cnot6l	9.52E-01
gene12974	13445	NC_000071.6	Cdk2ap1	9.52E-01
gene15466	108960	NC_000072.6	Irak2	9.51E-01
gene3146	332579	NC_000068.7	Card9	9.51E-01

gene8233	109333	NC_000069.6	Pkn2	9.51E-01
gene20093	67260	NC_000074.6	Cers4	9.50E-01
gene9386	52829	NC_000070.6	Lurap1l	9.50E-01
gene21750	66894	NC_000074.6	Wwp2	9.50E-01
gene11892	20005	NC_000071.6	Rpl9	9.50E-01
gene6569	207175	NC_000069.6	Cetn4	9.49E-01
gene20043	102098	NC_000074.6	Arhgef18	9.49E-01
gene21219	93762	NC_000074.6	Smarca5	9.49E-01
gene13893	320938	NC_000072.6	Tnpo3	9.49E-01
gene26733	15574	NC_000077.6	Hus1	9.48E-01
gene3242	227683	NC_000068.7	Coq4	9.48E-01
gene23503	12442	NC_000075.6	Ccnb2	9.48E-01
gene28519	104079	NC_000077.6	Nxph3	9.48E-01
gene7908	114301	NC_000069.6	Palmd	9.47E-01
gene33251	68927	NC_000079.6	Ptcd2	9.47E-01
gene29292	56745	NC_000077.6	C1qtnf1	9.46E-01
gene38944	14293	NC_000083.6	Fpr1	9.45E-01
gene37026	78541	NC_000081.6	Asb8	9.45E-01
gene12979	76167	NC_000071.6	Snrnp35	9.45E-01
gene16283	18726	NC_000073.6	Lilra6	9.45E-01
gene25606	68114	NC_000076.6	Mum1	9.45E-01
gene10742	545700	NC_000070.6	Smarca5-ps	9.45E-01
gene34372	74385	NC_000080.6	Ap5m1	9.45E-01
gene1291	20452	NC_000067.6	St8sia4	9.44E-01
gene21824	319518	NC_000074.6	Pdpr	9.44E-01
gene5172	320974	NC_000068.7	Lrrn4	9.44E-01
gene20000	244238	NC_000073.6	Mrgpre	9.44E-01
gene36398	22701	NC_000081.6	Zfp41	9.43E-01
gene24265	22221	NC_000075.6	Ubp1	9.43E-01
gene40875	56515	NC_000084.6	Rnf138	9.42E-01
gene28597	19155	NC_000077.6	Npepps	9.42E-01
gene26298	73192	NC_000076.6	Xpot	9.42E-01
gene7838	20912	NC_000069.6	Stxbp3	9.42E-01
gene3558	1E+08	NC_000068.7	Gm13477	9.42E-01
gene1711	16565	NC_000067.6	Kif21b	9.42E-01
gene5913	228913	NC_000068.7	Zfp217	9.42E-01
gene32985	1.1E+08	NC_000079.6	Gm41005	9.42E-01
gene17316	67224	NC_000073.6	Med29	9.42E-01
gene20167	54126	NC_000074.6	Arhgef7	9.42E-01
gene33717	67053	NC_000080.6	Rpp14	9.41E-01
gene22012	76014	NC_000074.6	Zc3h18	9.41E-01
gene10552	73162	NC_000070.6	Otud3	9.41E-01
gene1300	329217	NC_000067.6	Panct2	9.40E-01
gene16302	67425	NC_000073.6	Eps8l1	9.40E-01
gene36419	69020	NC_000081.6	Zfp707	9.40E-01
gene28967	52686	NC_000077.6	Mettl2	9.40E-01
gene31048	74251	NC_000078.6	Ankrd9	9.40E-01
gene37067	381022	NC_000081.6	Kmt2d	9.39E-01

gene40617	14236	NC_000083.6	Foxn2	9.39E-01
gene2159	26904	NC_000067.6	Sh2d1b1	9.39E-01
gene19111	209380	NC_000073.6	Gm4759	9.39E-01
gene39467	1.1E+08	NC_000083.6	Gm41568	9.38E-01
gene28796	20850	NC_000077.6	Stat5a	9.38E-01
gene27604	29869	NC_000077.6	Ulk2	9.38E-01
gene40986	30948	NC_000084.6	Bin1	9.38E-01
gene17302	13549	NC_000073.6	Dyrk1b	9.37E-01
gene22888	20660	NC_000075.6	Sorl1	9.37E-01
gene23205	56294	NC_000075.6	Ptpn9	9.37E-01
gene5825	99371	NC_000068.7	Arfgef2	9.37E-01
gene4551	56369	NC_000068.7	Apip	9.36E-01
gene12757	231659	NC_000071.6	Gcn1l1	9.36E-01
gene4461	1E+08	NC_000068.7	Gm29694	9.36E-01
gene14519	107569	NC_000072.6	Nt5c3	9.36E-01
gene21827	234736	NC_000074.6	Rfwd3	9.36E-01
gene33234	380863	NC_000079.6	Tmem171	9.35E-01
gene4493	56348	NC_000068.7	Hsd17b12	9.35E-01
gene40873	75964	NC_000084.6	Trappc8	9.35E-01
gene21003	234378	NC_000074.6	Klhl26	9.34E-01
gene12472	546882	NC_000071.6	Gm5987	9.34E-01
gene28203	59040	NC_000077.6	Rhot1	9.34E-01
gene26398	=Gene;ger	NC_000076.6	LOC105245328	9.34E-01
gene6987	73124	NC_000069.6	Golim4	9.34E-01
gene8905	60534	NC_000070.6	Fancg	9.34E-01
gene9410	77634	NC_000070.6	Snapc3	9.33E-01
gene15441	ene;gene=l	NC_000072.6	LOC108169182	9.32E-01
gene18275	269941	NC_000073.6	Chsy1	9.32E-01
gene1118	77040	NC_000067.6	Atg16l1	9.32E-01
gene1014	665246	NC_000067.6	Gm7553	9.32E-01
gene11352	52323	NC_000071.6	Klhl7	9.32E-01
gene15602	94044	NC_000072.6	Bcl2l13	9.31E-01
gene1312	1E+08	NC_000067.6	Gm31771	9.30E-01
gene26622	216516	NC_000077.6	Ccdc157	9.30E-01
gene33275	18260	NC_000079.6	Ocln	9.30E-01
gene45465	26908	NC_000087.7	Eif2s3y	9.30E-01
gene19511	246779	NC_000073.6	Il27	9.29E-01
gene10644	13706	NC_000070.6	Cela2a	9.29E-01
gene40020	63856	NC_000083.6	Taf8	9.29E-01
gene13355	231855	NC_000071.6	Ap5z1	9.29E-01
gene40053	224840	NC_000083.6	Treml4	9.29E-01
gene2129	66447	NC_000067.6	Mgst3	9.29E-01
gene14394	13063	NC_000072.6	Cycs	9.28E-01
gene9881	19366	NC_000070.6	Rad54l	9.28E-01
gene18551	65098	NC_000073.6	Zfand6	9.27E-01
gene17237	13087	NC_000073.6	Cyp2a5	9.27E-01
gene6380	12807	NC_000069.6	Hps3	9.27E-01
gene3035	76857	NC_000068.7	Spopl	9.27E-01

gene38065	208154	NC_000082.6	Btla	9.27E-01
gene40473	56298	NC_000083.6	Atl2	9.26E-01
gene39018	224585	NC_000083.6	Zfp160	9.26E-01
gene24305	73748	NC_000075.6	Gadl1	9.25E-01
gene1198	67921	NC_000067.6	Ube2f	9.25E-01
gene35174	12153	NC_000080.6	Bmp1	9.24E-01
gene19919	66491	NC_000073.6	Polr2l	9.24E-01
gene1575	1E+08	NC_000067.6	Gm29953	9.24E-01
gene34475	52535	NC_000080.6	Mettl17	9.24E-01
gene10377	20544	NC_000070.6	Slc9a1	9.24E-01
gene4873	68968	NC_000068.7	Cdan1	9.24E-01
gene5554	14854	NC_000068.7	Gss	9.23E-01
gene13008	208144	NC_000071.6	Dhx37	9.22E-01
gene15636	50530	NC_000072.6	Mfap5	9.22E-01
gene32875	210106	NC_000079.6	Papd7	9.22E-01
gene5540	16396	NC_000068.7	Itch	9.21E-01
gene6680	69257	NC_000069.6	Elf2	9.21E-01
gene32510	=Gene;ger	NC_000079.6	LOC108168085	9.21E-01
gene29762	77480	NC_000078.6	Kidins220	9.21E-01
gene13916	72649	NC_000072.6	Tmem209	9.20E-01
gene25814	69754	NC_000076.6	Fbxo7	9.19E-01
gene23102	18985	NC_000075.6	Pou2af1	9.19E-01
gene10094	11426	NC_000070.6	Macf1	9.19E-01
gene24090	434436	NC_000075.6	Lsmem2	9.18E-01
gene15631	13619	NC_000072.6	Phc1	9.18E-01
gene8048	70617	NC_000069.6	5730508B09Rik	9.18E-01
gene15808	68498	NC_000072.6	Tspan11	9.18E-01
gene1716	67886	NC_000067.6	Camsap2	9.18E-01
gene1850	66967	NC_000067.6	Edem3	9.18E-01
gene22707	22619	NC_000075.6	Siae	9.18E-01
gene13192	60363	NC_000071.6	Cldn15	9.18E-01
gene13376	57782	NC_000071.6	Rbak	9.18E-01
gene20002	13360	NC_000073.6	Dhcr7	9.16E-01
gene33191	68018	NC_000079.6	Col4a3bp	9.16E-01
gene9017	1E+08	NC_000070.6	Gm31850	9.16E-01
gene31638	319293	NC_000079.6	A530099J19Rik	9.16E-01
gene7173	67037	NC_000069.6	Pmf1	9.15E-01
gene16222	320204	NC_000072.6	Mettl20	9.15E-01
gene9871	17346	NC_000070.6	Mknk1	9.15E-01
gene42056	67849	NC_000085.6	Cdca5	9.15E-01
gene7439	18720	NC_000069.6	Pip5k1a	9.14E-01
gene29285	19157	NC_000077.6	Cyth1	9.14E-01
gene13670	18979	NC_000072.6	Pon1	9.13E-01
gene27226	14257	NC_000077.6	Flt4	9.13E-01
gene36863	328580	NC_000081.6	Tubgcp6	9.13E-01
gene23232	71742	NC_000075.6	Ulk3	9.13E-01
gene34887	68514	NC_000080.6	Micu2	9.13E-01
gene32899	218333	NC_000079.6	Ice1	9.13E-01

gene32457	14057	NC_000079.6	Sfxn1	9.12E-01
gene27382	=Gene;ger	NC_000077.6	LOC102633930	9.12E-01
gene26762	1.1E+08	NC_000077.6	Gm39581	9.12E-01
gene36414	105734	NC_000081.6	Tigd5	9.12E-01
gene24943	78334	NC_000076.6	Cdk19	9.12E-01
gene40144	224893	NC_000083.6	Zfp959	9.12E-01
gene5432	228775	NC_000068.7	Trib3	9.12E-01
gene37845	320213	NC_000082.6	Senp5	9.12E-01
gene35572	58245	NC_000080.6	Gpr180	9.12E-01
gene1115	16331	NC_000067.6	Inpp5d	9.12E-01
gene33236	218503	NC_000079.6	Fcho2	9.11E-01
gene8447	72098	NC_000070.6	Tmem68	9.11E-01
gene8971	230119	NC_000070.6	Zbtb5	9.10E-01
gene20049	50767	NC_000074.6	Pnpla6	9.10E-01
gene6956	97112	NC_000069.6	Nmd3	9.10E-01
gene3211	241289	NC_000068.7	Ppp1r26	9.10E-01
gene23558	55981	NC_000075.6	Pigb	9.10E-01
gene188	74229	NC_000067.6	Paqr8	9.09E-01
gene19729	22276	NC_000073.6	Uros	9.09E-01
gene8670	67490	NC_000070.6	Ufl1	9.09E-01
gene29011	50776	NC_000077.6	Polg2	9.09E-01
gene21838	12927	NC_000074.6	Bcar1	9.08E-01
gene1582	13034	NC_000067.6	Ctse	9.08E-01
gene39708	=Gene;ger	NC_000083.6	LOC102635200	9.08E-01
gene8563	68099	NC_000070.6	Fam92a	9.08E-01
gene23373	17130	NC_000075.6	Smad6	9.08E-01
gene39640	70274	NC_000083.6	Ly6g6e	9.08E-01
gene2260	98660	NC_000067.6	Atp1a2	9.07E-01
gene23721	108837	NC_000075.6	Ibtk	9.07E-01
gene12534	69587	NC_000071.6	Pcgf3	9.07E-01
gene24184	12530	NC_000075.6	Cdc25a	9.07E-01
gene37265	71726	NC_000081.6	Smug1	9.06E-01
gene8915	100213	NC_000070.6	Rusc2	9.06E-01
gene42843	192236	NC_000085.6	Hps1	9.06E-01
gene2152	15490	NC_000067.6	Hsd17b7	9.05E-01
gene11170	23857	NC_000071.6	Dmtf1	9.05E-01
gene42711	15925	NC_000085.6	Ide	9.05E-01
gene14051	72144	NC_000072.6	Slc37a3	9.05E-01
gene19645	207425	NC_000073.6	Wdr11	9.05E-01
gene20980	70616	NC_000074.6	Sugp1	9.04E-01
gene43158	1E+08	NC_000085.6	Gm18999	9.04E-01
gene41472	21453	NC_000084.6	Tcof1	9.04E-01
gene11617	71729	NC_000071.6	Rgs12	9.04E-01
gene35688	109828	NC_000081.6	C7	9.04E-01
gene12692	100756	NC_000071.6	Usp30	9.03E-01
gene21333	170833	NC_000074.6	Hook2	9.03E-01
gene37171	1E+08	NC_000081.6	Gm35853	9.03E-01
gene29899	217463	NC_000078.6	Snx13	9.03E-01

gene15738	68298	NC_000072.6	Ncapd2	9.03E-01
gene18449	269952	NC_000073.6	Gdpgp1	9.03E-01
gene11651	100855	NC_000071.6	Tbc1d14	9.03E-01
gene37870	51789	NC_000082.6	Tnk2	9.02E-01
gene40913	68046	NC_000084.6	2700062C07Rik	9.02E-01
gene24045	68644	NC_000075.6	Abhd14a	9.02E-01
gene21917	68533	NC_000074.6	Mphosph6	9.02E-01
gene28582	319371	NC_000077.6	D030028A08Rik	9.02E-01
gene17459	17136	NC_000073.6	Mag	9.02E-01
gene4844	76246	NC_000068.7	Rtf1	9.02E-01
gene9330	68285	NC_000070.6	C630043F03Rik	9.02E-01
gene1539	226414	NC_000067.6	Dars	9.01E-01
gene37658	73047	NC_000082.6	Camk2n2	9.01E-01
gene23668	215351	NC_000075.6	Senp6	9.01E-01
gene10435	1.1E+08	NC_000070.6	Gm42326	9.01E-01
gene10102	595136	NC_000070.6	Ndufs5	9.01E-01
gene32276	76000	NC_000079.6	5033430I15Rik	9.01E-01
gene6153	23856	NC_000068.7	Dido1	9.00E-01
gene12034	69940	NC_000071.6	Exoc1	9.00E-01
gene16033	54343	NC_000072.6	Atf7ip	8.99E-01
gene39168	13560	NC_000083.6	E4f1	8.99E-01
gene18009	22088	NC_000073.6	Tsg101	8.98E-01
gene37542	224020	NC_000082.6	Pi4ka	8.98E-01
gene33448	72198	NC_000079.6	Skiv2l2	8.98E-01
gene38184	67581	NC_000082.6	Tbc1d23	8.98E-01
gene22137	76332	NC_000074.6	Cog2	8.98E-01
gene1357	227449	NC_000067.6	Zcchc2	8.97E-01
gene22599	235130	NC_000075.6	Adamts15	8.97E-01
gene39399	=Gene;ger	NC_000083.6	LOC108168349	8.97E-01
gene15006	69837	NC_000072.6	Pcgf1	8.96E-01
gene8996	100121	NC_000070.6	Tdrd7	8.96E-01
gene5098	241638	NC_000068.7	Lzts3	8.96E-01
gene20757	16363	NC_000074.6	Irf2	8.96E-01
gene33881	67630	NC_000080.6	Samd8	8.95E-01
gene3629	65103	NC_000068.7	Arl6ip6	8.95E-01
gene2766	98828	NC_000068.7	Cdc123	8.95E-01
gene2906	16169	NC_000068.7	Il15ra	8.94E-01
gene23847	67958	NC_000075.6	U2surp	8.94E-01
gene40163	20359	NC_000083.6	Sema6b	8.94E-01
gene23408	270166	NC_000075.6	Clpx	8.93E-01
gene3831	16403	NC_000068.7	Itga6	8.93E-01
gene22	21399	NC_000067.6	Tcea1	8.92E-01
gene5486	228790	NC_000068.7	Asxl1	8.92E-01
gene27893	103712	NC_000077.6	6330403K07Rik	8.92E-01
gene5807	17979	NC_000068.7	Ncoa3	8.92E-01
gene16186	67533	NC_000072.6	Ppfibp1	8.92E-01
gene4455	76969	NC_000068.7	Chst1	8.92E-01
gene37484	20583	NC_000082.6	Snai2	8.91E-01

gene6713	67542	NC_000069.6	Cog6	8.91E-01
gene15026	27369	NC_000072.6	Dguok	8.91E-01
gene39607	625018	NC_000083.6	C4a	8.91E-01
gene21826	20340	NC_000074.6	Glg1	8.91E-01
gene22477	16201	NC_000075.6	Ilf3	8.91E-01
gene21687	102124	NC_000074.6	Enkd1	8.91E-01
gene6360	67472	NC_000069.6	Mtfr1	8.90E-01
gene24527	1.1E+08	NC_000076.6	Gm40595	8.90E-01
gene10087	56198	NC_000070.6	Heyl	8.90E-01
gene36036	223499	NC_000081.6	Dcaf13	8.90E-01
gene15444	97287	NC_000072.6	Mtmr14	8.90E-01
gene35085	71978	NC_000080.6	Ppp2r2a	8.89E-01
gene32492	18193	NC_000079.6	Nsd1	8.89E-01
gene39608	54402	NC_000083.6	Stk19	8.89E-01
gene29465	1.1E+08	NC_000078.6	Gm40835	8.88E-01
gene14487	231997	NC_000072.6	Fkbp14	8.88E-01
gene37877	224116	NC_000082.6	Muc20	8.88E-01
gene28245	19364	NC_000077.6	Rad51d	8.88E-01
gene43141	332397	NC_000085.6	Nanos1	8.88E-01
gene40750	19877	NC_000084.6	Rock1	8.88E-01
gene40408	66310	NC_000083.6	Dpy30	8.88E-01
gene16672	16881	NC_000073.6	Lig1	8.87E-01
gene14995	20355	NC_000072.6	Sema4f	8.87E-01
gene38829	110460	NC_000083.6	Acat2	8.87E-01
gene37464	432999	NC_000082.6	A930007A09Rik	8.87E-01
gene26180	1.1E+08	NC_000076.6	Gm40768	8.87E-01
gene1704	21956	NC_000067.6	Tnnt2	8.87E-01
gene31514	1E+08	NC_000079.6	Gm19191	8.87E-01
gene3370	269261	NC_000068.7	Rpl12	8.87E-01
gene16104	19691	NC_000072.6	Recql	8.86E-01
gene43029	18569	NC_000085.6	Pdcd4	8.86E-01
gene7743	433637	NC_000069.6	Gm5547	8.86E-01
gene26486	=Gene;ger	NC_000076.6	LOC108167764	8.86E-01
gene9747	12896	NC_000070.6	Cpt2	8.86E-01
gene13114	269713	NC_000071.6	Clip2	8.85E-01
gene32830	72807	NC_000079.6	Zfp429	8.85E-01
gene34036	66923	NC_000080.6	Pbrm1	8.85E-01
gene25656	16907	NC_000076.6	Lmnb2	8.85E-01
gene22026	1E+08	NC_000074.6	Gm34962	8.84E-01
gene32813	71226	NC_000079.6	4933433G19Rik	8.84E-01
gene2205	75472	NC_000067.6	Cfap126	8.84E-01
gene41605	69597	NC_000084.6	Afg3l2	8.84E-01
gene29976	1E+08	NC_000078.6	Gm2027	8.84E-01
gene26207	215436	NC_000076.6	Slc35e3	8.83E-01
gene26642	18082	NC_000077.6	Nipsnap1	8.83E-01
gene21813	20444	NC_000074.6	St3gal2	8.82E-01
gene35714	23880	NC_000081.6	Fyb	8.82E-01
gene21362	1.1E+08	NC_000074.6	Gm39214	8.82E-01

gene26673	72046	NC_000077.6	Urgcp	8.81E-01
gene4032	1E+08	NC_000068.7	Gm19426	8.81E-01
gene24135	23918	NC_000075.6	Impdh2	8.80E-01
gene20687	102103	NC_000074.6	Mtus1	8.80E-01
gene25785	216198	NC_000076.6	Tcp11l2	8.80E-01
gene33965	83997	NC_000080.6	Slmap	8.80E-01
gene26644	107829	NC_000077.6	Thoc5	8.79E-01
gene23698	75782	NC_000075.6	Lca5	8.79E-01
gene20262	52123	NC_000074.6	Agpat5	8.79E-01
gene15909	66441	NC_000072.6	Magohb	8.79E-01
gene23277	72141	NC_000075.6	Adpgk	8.78E-01
gene33449	218629	NC_000079.6	Dhx29	8.78E-01
gene6320	12350	NC_000069.6	Car3	8.78E-01
gene12943	208104	NC_000071.6	Mlxip	8.78E-01
gene28806	19183	NC_000077.6	Psmc3ip	8.78E-01
gene1611	213464	NC_000067.6	Rbbp5	8.78E-01
gene26632	74302	NC_000077.6	Mtmr3	8.77E-01
gene41402	110695	NC_000084.6	Aldh7a1	8.77E-01
gene15727	269800	NC_000072.6	Zfp384	8.76E-01
gene27178	94089	NC_000077.6	Trim7	8.76E-01
gene21330	69724	NC_000074.6	Rnaseh2a	8.76E-01
gene21997	20539	NC_000074.6	Slc7a5	8.76E-01
gene37917	20357	NC_000082.6	Sema5b	8.76E-01
gene3248	18286	NC_000068.7	Odf2	8.76E-01
gene21125	=Gene;ger	NC_000074.6	LOC102639683	8.75E-01
gene21096	236193	NC_000074.6	Zfp709	8.75E-01
gene11453	20423	NC_000071.6	Shh	8.75E-01
gene19082	244189	NC_000073.6	Gm4972	8.75E-01
gene9752	20280	NC_000070.6	Scp2	8.75E-01
gene12915	18439	NC_000071.6	P2rx7	8.74E-01
gene648	1E+08	NC_000067.6	Gm15834	8.74E-01
gene30784	1E+08	NC_000078.6	Gm20604	8.74E-01
gene30770	1E+08	NC_000078.6	Gm20036	8.74E-01
gene11898	71521	NC_000071.6	Pds5a	8.74E-01
gene10633	74202	NC_000070.6	Fblim1	8.74E-01
gene27389	246049	NC_000077.6	Slc36a2	8.73E-01
gene36529	67042	NC_000081.6	Ift27	8.73E-01
gene19686	67143	NC_000073.6	Ikzf5	8.73E-01
gene38896	67544	NC_000083.6	Fam120b	8.73E-01
gene34493	76338	NC_000080.6	Rab2b	8.72E-01
gene15629	625931	NC_000072.6	Gm6637	8.72E-01
gene26008	382407	NC_000076.6	Gad1-ps	8.72E-01
gene950	21782	NC_000067.6	Tfdp1-ps	8.71E-01
gene30535	66204	NC_000078.6	Acyp1	8.71E-01
gene20012	233977	NC_000073.6	Ppfia1	8.71E-01
gene6279	11770	NC_000069.6	Fabp4	8.71E-01
gene16875	243867	NC_000073.6	Fbxo46	8.71E-01
gene25444	74953	NC_000076.6	4930483K19Rik	8.70E-01

gene26485	16404	NC_000076.6	Itga7	8.70E-01
gene15990	70686	NC_000072.6	Dusp16	8.69E-01
gene34759	105651	NC_000080.6	Ppp1r3e	8.69E-01
gene21528	234582	NC_000074.6	Ccdc102a	8.68E-01
gene5764	76233	NC_000068.7	Dnttip1	8.68E-01
gene34949	68177	NC_000080.6	Ebpl	8.68E-01
gene28116	268448	NC_000077.6	Phf12	8.67E-01
gene25443	16987	NC_000076.6	Lss	8.67E-01
gene16276	18722	NC_000073.6	Pira1	8.67E-01
gene27732	57785	NC_000077.6	Rangrf	8.67E-01
gene36188	76773	NC_000081.6	Wdyhv1	8.66E-01
gene15591	214899	NC_000072.6	Kdm5a	8.66E-01
gene9200	72479	NC_000070.6	Hsdl2	8.65E-01
gene29777	108089	NC_000078.6	Rnf144a	8.65E-01
gene8599	209212	NC_000070.6	Osgin2	8.65E-01
gene7828	229722	NC_000069.6	5330417C22Rik	8.65E-01
gene23188	19200	NC_000075.6	Pstpip1	8.64E-01
gene33807	22194	NC_000080.6	Ube2e1	8.64E-01
gene21375	20437	NC_000074.6	Siah1a	8.63E-01
gene21740	21750	NC_000074.6	Terf2	8.63E-01
gene37814	224092	NC_000082.6	Lsg1	8.63E-01
gene16212	320727	NC_000072.6	Ipo8	8.63E-01
gene29065	LOC10050	NC_000077.6	LOC100503496	8.62E-01
gene16331	664994	NC_000073.6	Isoc2a	8.62E-01
gene40553	213575	NC_000083.6	Dync2li1	8.62E-01
gene28653	320655	NC_000077.6	Pgap3	8.62E-01
gene37203	223917	NC_000081.6	Krt79	8.62E-01
gene36896	68708	NC_000081.6	Rabl2	8.61E-01
gene28613	22658	NC_000077.6	Pcgf2	8.61E-01
gene1270	51800	NC_000067.6	Bok	8.61E-01
gene26439	103135	NC_000076.6	Pan2	8.60E-01
gene21374	66887	NC_000074.6	Lonp2	8.60E-01
gene32157	17084	NC_000079.6	Ly86	8.59E-01
gene6718	1E+08	NC_000069.6	Gm16206	8.59E-01
gene37919	224132	NC_000082.6	Dirc2	8.59E-01
gene33721	71393	NC_000080.6	Kctd6	8.59E-01
gene26988	67579	NC_000077.6	Cpeb4	8.59E-01
gene21720	22751	NC_000074.6	Zfp90	8.58E-01
gene17138	22594	NC_000073.6	Xrcc1	8.57E-01
gene28638	19014	NC_000077.6	Med1	8.57E-01
gene11031	67389	NC_000070.6	Fam132a	8.57E-01
gene41078	67199	NC_000084.6	Pfdn1	8.57E-01
gene11860	100532	NC_000071.6	Rell1	8.56E-01
gene32972	12380	NC_000079.6	Cast	8.56E-01
gene5610	320706	NC_000068.7	Soga1	8.56E-01
gene27690	52892	NC_000077.6	Sco1	8.56E-01
gene15359	1E+08	NC_000072.6	Gm6565	8.56E-01
gene33521	67392	NC_000079.6	4833420G17Rik	8.56E-01

gene8464	18619	NC_000070.6	Penk	8.55E-01
gene572	16329	NC_000067.6	Inpp1	8.55E-01
gene41621	108654	NC_000084.6	Fam210a	8.54E-01
gene33969	211922	NC_000080.6	Dennd6a	8.54E-01
gene11957	231287	NC_000071.6	Atp10d	8.54E-01
gene11055	231004	NC_000070.6	Samd11	8.53E-01
gene30305	76357	NC_000078.6	Trmt5	8.53E-01
gene39180	18207	NC_000083.6	Nthl1	8.53E-01
gene9711	230577	NC_000070.6	Pars2	8.52E-01
gene628	73467	NC_000067.6	1700066M21Rik	8.52E-01
gene26302	270802	NC_000076.6	BC048403	8.52E-01
gene35696	67515	NC_000081.6	Ttc33	8.52E-01
gene34482	1E+08	NC_000080.6	Gm16617	8.52E-01
gene10814	194189	NC_000070.6	Gm13136	8.52E-01
gene21186	78651	NC_000074.6	Lsm6	8.51E-01
gene20741	66756	NC_000074.6	Cfap97	8.51E-01
gene40748	225160	NC_000084.6	Thoc1	8.51E-01
gene39283	12005	NC_000083.6	Axin1	8.50E-01
gene38198	54613	NC_000082.6	St3gal6	8.50E-01
gene3291	66617	NC_000068.7	Ntmt1	8.49E-01
gene13120	16885	NC_000071.6	Limk1	8.49E-01
gene22295	66396	NC_000075.6	Ccdc82	8.49E-01
gene22936	102423	NC_000075.6	Hinfp	8.48E-01
gene29152	58222	NC_000077.6	Rab37	8.48E-01
gene6619	20873	NC_000069.6	Plk4	8.48E-01
gene34853	75339	NC_000080.6	Mphosph8	8.48E-01
gene40978	73473	NC_000084.6	Iws1	8.48E-01
gene21778	244646	NC_000074.6	Pkd1l3	8.47E-01
gene16176	71177	NC_000072.6	Asun	8.47E-01
gene39970	72322	NC_000083.6	Xpo5	8.46E-01
gene5263	74528	NC_000068.7	Mgme1	8.46E-01
gene5567	14563	NC_000068.7	Gdf5	8.46E-01
gene32842	212281	NC_000079.6	Zfp729a	8.46E-01
gene39340	67097	NC_000083.6	Rps10	8.46E-01
gene18649	67669	NC_000073.6	I7Rn6	8.46E-01
gene5180	12156	NC_000068.7	Bmp2	8.45E-01
gene20216	19414	NC_000074.6	Rasa3	8.45E-01
gene2399	226747	NC_000067.6	Ahctf1	8.45E-01
gene2528	23827	NC_000067.6	Bpnt1	8.45E-01
gene7465	11863	NC_000069.6	Arnt	8.44E-01
gene30384	56217	NC_000078.6	Mpp5	8.44E-01
gene5160	74182	NC_000068.7	Gpcpd1	8.44E-01
gene24120	434437	NC_000075.6	Amt	8.44E-01
gene37130	11908	NC_000081.6	Atf1	8.44E-01
gene2226	71740	NC_000067.6	Nectin4	8.44E-01
gene665	381259	NC_000067.6	Tmem237	8.44E-01
gene28636	217154	NC_000077.6	Stac2	8.43E-01
gene24517	381990	NC_000076.6	Zbtb2	8.43E-01

gene39274	50817	NC_000083.6	Capn15	8.43E-01
gene40678	67440	NC_000084.6	Mtpap	8.43E-01
gene30496	238317	NC_000078.6	Elmsan1	8.43E-01
gene18886	101700	NC_000073.6	Trim68	8.43E-01
gene21986	76454	NC_000074.6	Fbxo31	8.42E-01
gene16204	320737	NC_000072.6	4732416N19Rik	8.42E-01
gene17715	233168	NC_000073.6	AI987944	8.42E-01
gene14885	104263	NC_000072.6	Kdm3a	8.42E-01
gene22362	1E+08	NC_000075.6	Gm19178	8.42E-01
gene25251	70432	NC_000076.6	Rufy2	8.41E-01
gene38539	66609	NC_000082.6	Cryzl1	8.41E-01
gene3141	227638	NC_000068.7	Qsox2	8.41E-01
gene221	98366	NC_000067.6	Smap1	8.40E-01
gene24351	108737	NC_000075.6	Oxsr1	8.40E-01
gene41465	104027	NC_000084.6	Synpo	8.40E-01
gene35032	78569	NC_000080.6	9630015K15Rik	8.39E-01
gene25877	75089	NC_000076.6	Uhrf1bp1l	8.39E-01
gene33277	218518	NC_000079.6	Marveld2	8.39E-01
gene2410	277333	NC_000067.6	Gm5069	8.39E-01
gene20992	76813	NC_000074.6	Armc6	8.39E-01
gene40665	71745	NC_000084.6	Cul2	8.39E-01
gene37695	239759	NC_000082.6	Liph	8.39E-01
gene18002	246694	NC_000073.6	Hps5	8.39E-01
gene9253	66440	NC_000070.6	Cdc26	8.39E-01
gene9186	230249	NC_000070.6	AI314180	8.38E-01
gene27391	215335	NC_000077.6	Slc36a1	8.38E-01
gene34056	26363	NC_000080.6	Btd	8.37E-01
gene36972	105722	NC_000081.6	Ano6	8.37E-01
gene37144	19009	NC_000081.6	Pou6f1	8.37E-01
gene36004	382985	NC_000081.6	Rrm2b	8.37E-01
gene19333	18704	NC_000073.6	Pik3c2a	8.37E-01
gene13316	=Gene;ger	NC_000071.6	LOC108169079	8.37E-01
gene18453	233406	NC_000073.6	Prc1	8.37E-01
gene30023	1.1E+08	NC_000078.6	Gm40422	8.37E-01
gene9305	21455	NC_000070.6	Tcp1-ps1	8.36E-01
gene42603	109113	NC_000085.6	Uhrf2	8.36E-01
gene37063	22410	NC_000081.6	Wnt10b	8.36E-01
gene35653	223267	NC_000080.6	Ggact	8.36E-01
gene2806	16588	NC_000068.7	Kin	8.35E-01
gene13258	22697	NC_000071.6	Zscan21	8.35E-01
gene17934	16866	NC_000073.6	Lhb	8.35E-01
gene37506	623331	NC_000082.6	D16Ertd727e	8.35E-01
gene30767	74413	NC_000078.6	Tc2n	8.35E-01
gene32221	621976	NC_000079.6	Tmem170b	8.35E-01
gene34298	54342	NC_000080.6	Gnpnat1	8.35E-01
gene37224	223921	NC_000081.6	Aaas	8.35E-01
gene39974	224813	NC_000083.6	Lrrc73	8.35E-01
gene25686	209047	NC_000076.6	Gipc3	8.34E-01

gene12439	666726	NC_000071.6	Gm8258	8.34E-01
gene38917	1E+08	NC_000083.6	Gm3320	8.34E-01
gene24489	19054	NC_000075.6	Ppp2r3d	8.34E-01
gene34120	20618	NC_000080.6	Sncg	8.33E-01
gene42531	1E+08	NC_000085.6	Gm34235	8.33E-01
gene27527	19416	NC_000077.6	Rasd1	8.33E-01
gene22038	77087	NC_000074.6	Ankrd11	8.33E-01
gene12999	1E+08	NC_000071.6	Gm32701	8.33E-01
gene18444	209225	NC_000073.6	Zfp710	8.33E-01
gene35	12421	NC_000067.6	Rb1cc1	8.32E-01
gene10919	230935	NC_000070.6	Dnajc11	8.32E-01
gene38662	C10816831	NC_000083.6	LOC108168313	8.32E-01
gene6240	67306	NC_000069.6	Zc2hc1a	8.32E-01
gene22056	78779	NC_000074.6	Spata2l	8.32E-01
gene19820	74237	NC_000073.6	Tubgcp2	8.32E-01
gene6469	241915	NC_000069.6	Phc3	8.32E-01
gene10515	170707	NC_000070.6	Usp48	8.31E-01
gene15759	22371	NC_000072.6	Vwf	8.31E-01
gene4436	26427	NC_000068.7	Creb3l1	8.30E-01
gene4008	16410	NC_000068.7	Itgav	8.30E-01
gene39519	224697	NC_000083.6	Adamts10	8.30E-01
gene36740	66251	NC_000081.6	Arfgap3	8.30E-01
gene28294	217026	NC_000077.6	Heatr6	8.29E-01
gene19234	57355	NC_000073.6	BC051019	8.29E-01
gene42787	240665	NC_000085.6	Ccnj	8.29E-01
gene19597	244219	NC_000073.6	Zfp668	8.29E-01
gene19516	20887	NC_000073.6	Sult1a1	8.29E-01
gene37070	74775	NC_000081.6	Lmbr1l	8.28E-01
gene2307	226691	NC_000067.6	AI607873	8.28E-01
gene10962	1E+08	NC_000070.6	Gm31566	8.28E-01
gene25205	27355	NC_000076.6	Pald1	8.28E-01
gene39319	16440	NC_000083.6	Itpr3	8.28E-01
gene24005	=Gene;ger	NC_000075.6	LOC108167681	8.28E-01
gene31725	ene;gene=l	NC_000079.6	LOC108168067	8.28E-01
gene10125	14201	NC_000070.6	Fhl3	8.28E-01
gene38697	66616	NC_000083.6	Snx9	8.28E-01
gene26755	60530	NC_000077.6	Fignl1	8.27E-01
gene8458	66433	NC_000070.6	Chchd7	8.27E-01
gene13493	20018	NC_000071.6	Polr1d	8.27E-01
gene27736	68964	NC_000077.6	Ctc1	8.27E-01
gene42345	107321	NC_000085.6	Lpxn	8.27E-01
gene25390	110279	NC_000076.6	Bcr	8.26E-01
gene32521	78111	NC_000079.6	4930451E10Rik	8.26E-01
gene10642	214063	NC_000070.6	Dnajc16	8.25E-01
gene8145	110173	NC_000069.6	Manba	8.25E-01
gene15173	1E+08	NC_000072.6	Gm26588	8.25E-01
gene25928	1E+08	NC_000076.6	Gm15915	8.25E-01
gene35481	1E+08	NC_000080.6	Gm17066	8.25E-01

gene10133	17764	NC_000070.6	Mtf1	8.25E-01
gene3136	98766	NC_000068.7	Ubac1	8.25E-01
gene39347	224648	NC_000083.6	Uhrf1bp1	8.25E-01
gene5210	16449	NC_000068.7	Jag1	8.24E-01
gene38087	58998	NC_000082.6	Nectin3	8.24E-01
gene36483	71591	NC_000081.6	Zfp251	8.24E-01
gene30301	20471	NC_000078.6	Six1	8.23E-01
gene12663	=Gene;ger	NC_000071.6	LOC105246973	8.23E-01
gene11312	22791	NC_000071.6	Dnajc2	8.23E-01
gene26321	67074	NC_000076.6	Mon2	8.23E-01
gene3253	71820	NC_000068.7	Wdr34	8.22E-01
gene35150	246710	NC_000080.6	Rhobtb2	8.22E-01
gene13440	22757	NC_000071.6	Zkscan5	8.22E-01
gene32128	1E+08	NC_000079.6	Gm16984	8.22E-01
gene3778	329416	NC_000068.7	Nostrin	8.21E-01
gene18441	11778	NC_000073.6	Ap3s2	8.21E-01
gene22122	56199	NC_000074.6	Abcb10	8.21E-01
gene24613	69412	NC_000076.6	1700016L04Rik	8.21E-01
gene27141	76884	NC_000077.6	Cyfip2	8.21E-01
gene24689	1E+08	NC_000076.6	Gm33803	8.20E-01
gene110	212442	NC_000067.6	Lactb2	8.20E-01
gene27883	327957	NC_000077.6	Scimp	8.20E-01
gene11716	665775	NC_000071.6	Bod1l	8.20E-01
gene5269	228714	NC_000068.7	Csrp2bp	8.20E-01
gene37597	18951	NC_000082.6	sep-05	8.20E-01
gene21840	23837	NC_000074.6	Cfdp1	8.19E-01
gene28300	217030	NC_000077.6	Synrg	8.19E-01
gene3723	227960	NC_000068.7	Gca	8.19E-01
gene9725	546840	NC_000070.6	Ldlrad1	8.19E-01
gene8101	23971	NC_000069.6	Papss1	8.19E-01
gene479	22019	NC_000067.6	Tpp2	8.18E-01
gene43017	ne;gene=L	NC_000085.6	LOC73899	8.18E-01
gene28293	66107	NC_000077.6	Wfdc21	8.18E-01
gene34414	219022	NC_000080.6	Ttc5	8.17E-01
gene11051	231002	NC_000070.6	Plekhn1	8.17E-01
gene3215	227671	NC_000068.7	Gbgt1	8.16E-01
gene37855	68980	NC_000082.6	Wdr53	8.16E-01
gene31067	17169	NC_000078.6	Mark3	8.16E-01
gene6719	212127	NC_000069.6	Proser1	8.16E-01
gene16024	668137	NC_000072.6	Gm8994	8.16E-01
gene32348	70930	NC_000079.6	Nol8	8.16E-01
gene2088	68481	NC_000067.6	Mpzl1	8.15E-01
gene17186	654804	NC_000073.6	4732471J01Rik	8.15E-01
gene35690	239319	NC_000081.6	Card6	8.14E-01
gene23151	56506	NC_000075.6	Cib2	8.14E-01
gene15821	14235	NC_000072.6	Foxm1	8.14E-01
gene3272	108958	NC_000068.7	Fam73b	8.14E-01
gene23639	67968	NC_000075.6	Ooep	8.13E-01

gene13953	319849	NC_000072.6	Plxna4os1	8.13E-01
gene9896	50927	NC_000070.6	Nasp	8.13E-01
gene33153	218454	NC_000079.6	Lhfpl2	8.13E-01
gene36125	239435	NC_000081.6	Aard	8.12E-01
gene11631	18818	NC_000071.6	Plk-ps1	8.12E-01
gene26830	55963	NC_000077.6	Slc1a4	8.12E-01
gene42209	74760	NC_000085.6	Rab3il1	8.12E-01
gene12355	67870	NC_000071.6	Enoph1	8.12E-01
gene11935	11980	NC_000071.6	Atp8a1	8.12E-01
gene16784	232906	NC_000073.6	Arhgap35	8.11E-01
gene41700	16891	NC_000084.6	Lipg	8.11E-01
gene30851	192119	NC_000078.6	Dicer1	8.11E-01
gene18267	1E+08	NC_000073.6	Gm33234	8.11E-01
gene15862	71183	NC_000072.6	Clec12b	8.11E-01
gene39113	449521	NC_000083.6	Zfp213	8.11E-01
gene10374	269589	NC_000070.6	Sytl1	8.10E-01
gene31090	668303	NC_000078.6	Kif26a	8.10E-01
gene8820	74164	NC_000070.6	Nfx1	8.10E-01
gene1122	227333	NC_000067.6	Dgkd	8.10E-01
gene28128	68385	NC_000077.6	Tlcd1	8.10E-01
gene37645	27416	NC_000082.6	Abcc5	8.10E-01
gene9757	414872	NC_000070.6	Zyg11b	8.09E-01
gene698	12487	NC_000067.6	Cd28	8.09E-01
gene32835	408067	NC_000079.6	Zfp874b	8.09E-01
gene26617	67900	NC_000077.6	Mtfp1	8.09E-01
gene37307	71063	NC_000082.6	Zfp597	8.09E-01
gene3625	56324	NC_000068.7	Stam2	8.08E-01
gene24470	93730	NC_000075.6	Lztfl1	8.08E-01
gene13063	100678	NC_000071.6	Psph	8.08E-01
gene41058	76594	NC_000084.6	Dnajc18	8.07E-01
gene8833	66401	NC_000070.6	Nudt2	8.07E-01
gene13246	1E+08	NC_000071.6	Gm36551	8.07E-01
gene34854	1E+08	NC_000080.6	Gm16973	8.06E-01
gene38165	271377	NC_000082.6	Zbtb11	8.06E-01
gene962	23874	NC_000067.6	Farsb	8.06E-01
gene24502	76142	NC_000076.6	Ppp1r14c	8.06E-01
gene3965	22193	NC_000068.7	Ube2e3	8.05E-01
gene40610	225055	NC_000083.6	Fbxo11	8.05E-01
gene28825	73635	NC_000077.6	Ptges3l	8.05E-01
gene36285	239510	NC_000081.6	Phf20l1	8.05E-01
gene30180	328108	NC_000078.6	Fam179b	8.05E-01
gene30239	18080	NC_000078.6	Nin	8.05E-01
gene10852	=Gene;ger	NC_000070.6	LOC108168987	8.05E-01
gene10227	230761	NC_000070.6	Zfp362	8.05E-01
gene23053	26951	NC_000075.6	Zw10	8.05E-01
gene11862	66681	NC_000071.6	Pgm1	8.04E-01
gene7747	433638	NC_000069.6	I830077J02Rik	8.04E-01
gene29164	237988	NC_000077.6	Cdr2l	8.04E-01

gene8621	107568	NC_000070.6	Wwp1	8.04E-01
gene19013	434218	NC_000073.6	Trim34b	8.04E-01
gene26474	78428	NC_000076.6	Pym1	8.04E-01
gene24482	1.1E+08	NC_000075.6	Gm39469	8.04E-01
gene37770	12737	NC_000082.6	Cldn1	8.04E-01
gene37120	1E+08	NC_000081.6	Gm17057	8.04E-01
gene4481	14043	NC_000068.7	Ext2	8.03E-01
gene36186	70472	NC_000081.6	Atad2	8.02E-01
gene26938	71701	NC_000077.6	Pnpt1	8.02E-01
gene31624	107574	NC_000079.6	Tcrg-C2	8.02E-01
gene42592	226089	NC_000085.6	Ric1	8.02E-01
gene13926	17294	NC_000072.6	Mest	8.02E-01
gene41623	67897	NC_000084.6	Rnmt	8.01E-01
gene5714	18769	NC_000068.7	Pkig	8.00E-01
gene30084	59032	NC_000078.6	Ppp2r3c	8.00E-01
gene26587	16886	NC_000077.6	Limk2	8.00E-01
gene13147	215114	NC_000071.6	Hip1	8.00E-01
gene28327	66884	NC_000077.6	Appbp2	8.00E-01
gene26851	245944	NC_000077.6	Vps54	8.00E-01
gene3221	69987	NC_000068.7	Spaca9	8.00E-01
gene28069	67390	NC_000077.6	Rnmtl1	8.00E-01
gene43031	56392	NC_000085.6	Shoc2	7.99E-01
gene11581	74504	NC_000071.6	Fam53a	7.99E-01
gene28583	78912	NC_000077.6	Sp2	7.98E-01
gene16799	629378	NC_000073.6	Dact3	7.98E-01
gene10864	16561	NC_000070.6	Kif1b	7.98E-01
gene17420	11803	NC_000073.6	Aplp1	7.97E-01
gene19245	22390	NC_000073.6	Wee1	7.96E-01
gene27724	77579	NC_000077.6	Myh10	7.96E-01
gene13976	109624	NC_000072.6	Cald1	7.96E-01
gene37327	83396	NC_000082.6	Glis2	7.96E-01
gene11291	242860	NC_000071.6	Rsbn1l	7.96E-01
gene21458	319388	NC_000074.6	Irx3os	7.95E-01
gene37017	1E+08	NC_000081.6	Gm33690	7.95E-01
gene25588	13496	NC_000076.6	Arid3a	7.95E-01
gene16045	109978	NC_000072.6	Art4	7.95E-01
gene6250	20257	NC_000069.6	Stmn2	7.94E-01
gene28953	217232	NC_000077.6	Cdc27	7.94E-01
gene4983	58800	NC_000068.7	Trpm7	7.94E-01
gene23305	1E+08	NC_000075.6	Gm33643	7.94E-01
gene22574	66948	NC_000075.6	Acad8	7.94E-01
gene41671	ene;gene=L	NC_000084.6	LOC105246496	7.94E-01
gene13444	666311	NC_000071.6	Zscan25	7.94E-01
gene32504	56320	NC_000079.6	Dbn1	7.93E-01
gene29528	23967	NC_000078.6	Osr1	7.93E-01
gene40295	106572	NC_000083.6	Rab31	7.93E-01
gene38405	67768	NC_000082.6	N6amt1	7.93E-01
gene17270	66367	NC_000073.6	2310022A10Rik	7.93E-01

gene39909	94185	NC_000083.6	Tnfrsf21	7.93E-01
gene20740	102141	NC_000074.6	Snx25	7.93E-01
gene28201	216991	NC_000077.6	Adap2	7.92E-01
gene22961	102693	NC_000075.6	Phldb1	7.92E-01
gene6736	56790	NC_000069.6	Supt20	7.92E-01
gene28152	22370	NC_000077.6	Vtn	7.91E-01
gene5688	1E+08	NC_000068.7	Cisd3b	7.91E-01
gene17960	13170	NC_000073.6	Dbp	7.91E-01
gene37053	1E+08	NC_000081.6	9330020H09Rik	7.91E-01
gene41936	=Gene;ger	NC_000085.6	LOC102631992	7.90E-01
gene17283	71163	NC_000073.6	Zfp626	7.90E-01
gene42078	19309	NC_000085.6	Pygm	7.90E-01
gene33166	218460	NC_000079.6	Wdr41	7.90E-01
gene28795	20851	NC_000077.6	Stat5b	7.90E-01
gene20477	320191	NC_000074.6	Hook3	7.89E-01
gene506	73674	NC_000067.6	Wdr75	7.89E-01
gene24044	109652	NC_000075.6	Acy1	7.89E-01
gene7742	109905	NC_000069.6	Rap1a	7.88E-01
gene28910	68087	NC_000077.6	Dcakd	7.88E-01
gene28351	72508	NC_000077.6	Rps6kb1	7.88E-01
gene15640	14562	NC_000072.6	Gdf3	7.87E-01
gene27664	68626	NC_000077.6	Elac2	7.87E-01
gene28954	17896	NC_000077.6	Myl4	7.86E-01
gene30505	52708	NC_000078.6	Zfp410	7.86E-01
gene37908	278725	NC_000082.6	E130310I04Rik	7.86E-01
gene39987	381101	NC_000083.6	Dnph1	7.86E-01
gene17646	19777	NC_000073.6	Uri1	7.86E-01
gene37593	21380	NC_000082.6	Tbx1	7.86E-01
gene8538	72656	NC_000070.6	Ints8	7.86E-01
gene798	14768	NC_000067.6	Lancl1	7.85E-01
gene13967	11677	NC_000072.6	Akr1b3	7.85E-01
gene10656	214359	NC_000070.6	Tmem51	7.85E-01
gene25442	54387	NC_000076.6	Mcm3ap	7.85E-01
gene36441	1E+08	NC_000081.6	Mirt2	7.84E-01
gene30789	238386	NC_000078.6	Btbd7	7.84E-01
gene2626	98736	NC_000067.6	1700034H15Rik	7.84E-01
gene31557	83924	NC_000079.6	Gpr137b	7.84E-01
gene38856	17356	NC_000083.6	Mllt4	7.84E-01
gene33327	218543	NC_000079.6	Srek1	7.83E-01
gene42730	19662	NC_000085.6	Rbp4	7.83E-01
gene571	98682	NC_000067.6	Mfsd6	7.83E-01
gene25357	216080	NC_000076.6	Ube2d1	7.83E-01
gene26891	72246	NC_000077.6	1700030C12Rik	7.83E-01
gene9085	14211	NC_000070.6	Smc2	7.82E-01
gene33718	218699	NC_000080.6	Pxk	7.82E-01
gene34988	1E+08	NC_000080.6	Gm26536	7.82E-01
gene31682	22758	NC_000079.6	Zscan12	7.82E-01
gene23239	353190	NC_000075.6	Edc3	7.81E-01

gene18670	1E+08	NC_000073.6	Gm26944	7.81E-01
gene13409	17341	NC_000071.6	Bhlha15	7.81E-01
gene14042	1E+08	NC_000072.6	Gm38489	7.81E-01
gene10437	66146	NC_000070.6	Tmem57	7.80E-01
gene41723	17126	NC_000084.6	Smad2	7.80E-01
gene27325	67966	NC_000077.6	Zcchc10	7.80E-01
gene8764	73205	NC_000070.6	3110043O21Rik	7.79E-01
gene37010	1E+08	NC_000081.6	Gm33531	7.78E-01
gene37553	77626	NC_000082.6	Smpd4	7.78E-01
gene3809	228012	NC_000068.7	Tlk1	7.78E-01
gene42945	75146	NC_000085.6	Tmem180	7.77E-01
gene30546	81703	NC_000078.6	Jdp2	7.77E-01
gene14491	387524	NC_000072.6	Znrf2	7.77E-01
gene15262	=Gene;ger	NC_000072.6	LOC108169155	7.77E-01
gene5602	74487	NC_000068.7	5430405H02Rik	7.76E-01
gene7761	69206	NC_000069.6	2010016I18Rik	7.76E-01
gene39272	621239	NC_000083.6	Nhlrc4	7.76E-01
gene21099	170938	NC_000074.6	Zfp617	7.76E-01
gene10638	68817	NC_000070.6	Ddi2	7.76E-01
gene20722	1E+08	NC_000074.6	Gm30504	7.76E-01
gene10968	59002	NC_000070.6	Wrap73	7.75E-01
gene33340	67288	NC_000079.6	Srek1ip1	7.75E-01
gene1635	20668	NC_000067.6	Sox13	7.75E-01
gene23528	68178	NC_000075.6	Cgnl1	7.75E-01
gene551	17912	NC_000067.6	Myo1b	7.75E-01
gene10632	320456	NC_000070.6	B330016D10Rik	7.75E-01
gene16886	20167	NC_000073.6	Rtn2	7.74E-01
gene13287	80752	NC_000071.6	Fam20c	7.74E-01
gene6492	67414	NC_000069.6	Mfn1	7.74E-01
gene25967	216238	NC_000076.6	Eea1	7.73E-01
gene10630	56381	NC_000070.6	Spen	7.73E-01
gene23386	213550	NC_000075.6	Dis3l	7.73E-01
gene4716	107723	NC_000068.7	Slc12a6	7.72E-01
gene37555	94112	NC_000082.6	Med15	7.72E-01
gene5007	68126	NC_000068.7	Fahd2a	7.72E-01
gene17614	56330	NC_000073.6	Pdcd5	7.72E-01
gene29437	619325	NC_000078.6	I920046F24Rik	7.72E-01
gene23642	214763	NC_000075.6	Mb21d1	7.72E-01
gene22069	104346	NC_000074.6	Gas8	7.72E-01
gene29834	104943	NC_000078.6	Fam110c	7.72E-01
gene9957	230676	NC_000070.6	Szt2	7.72E-01
gene30397	1.1E+08	NC_000078.6	Gm40462	7.72E-01
gene8078	99683	NC_000069.6	Sec24b	7.71E-01
gene6105	71532	NC_000068.7	Fam217b	7.71E-01
gene18263	21872	NC_000073.6	Tjp1	7.71E-01
gene5509	228801	NC_000068.7	Bpifb1	7.71E-01
gene22981	1E+08	NC_000075.6	Gm10684	7.70E-01
gene5070	67333	NC_000068.7	Stk35	7.70E-01

gene33282	12572	NC_000079.6	Cdk7	7.70E-01
gene37879	1E+08	NC_000082.6	Rubcn	7.69E-01
gene15657	26888	NC_000072.6	Clec4a2	7.69E-01
gene30771	110616	NC_000078.6	Atxn3	7.69E-01
gene25457	12822	NC_000076.6	Col18a1	7.68E-01
gene21245	244548	NC_000074.6	Elmod2	7.68E-01
gene4977	14391	NC_000068.7	Gabpb1	7.68E-01
gene7573	67549	NC_000069.6	Gpr89	7.68E-01
gene12791	231668	NC_000071.6	Vsig10	7.67E-01
gene34822	17228	NC_000080.6	Cma1	7.67E-01
gene34333	1E+08	NC_000080.6	Gm10371	7.67E-01
gene26602	74522	NC_000077.6	Morc2a	7.67E-01
gene11664	22393	NC_000071.6	Wfs1	7.67E-01
gene17613	245886	NC_000073.6	Ankrd27	7.66E-01
gene37306	385674	NC_000082.6	Zfp174	7.66E-01
gene18860	269966	NC_000073.6	Nup98	7.66E-01
gene12393	=Gene;ger	NC_000071.6	LOC108169124	7.66E-01
gene27370	216742	NC_000077.6	Fnip1	7.66E-01
gene13912	22214	NC_000072.6	Ube2h	7.65E-01
gene3779	66442	NC_000068.7	Spc25	7.65E-01
gene18000	20208	NC_000073.6	Saa1	7.65E-01
gene20082	72267	NC_000074.6	Lrrc8e	7.65E-01
gene21944	22224	NC_000074.6	Usp10	7.64E-01
gene12702	77697	NC_000071.6	Mmab	7.64E-01
gene9012	=Gene;ger	NC_000070.6	LOC108168940	7.64E-01
gene193	1E+08	NC_000067.6	Gm28836	7.64E-01
gene684	72750	NC_000067.6	Fam117b	7.64E-01
gene19368	64297	NC_000073.6	Gprc5b	7.64E-01
gene9399	68268	NC_000070.6	Zdhhc21	7.64E-01
gene31535	67475	NC_000079.6	Ero1lb	7.63E-01
gene11545	20534	NC_000071.6	Slc4a1ap	7.63E-01
gene40425	240168	NC_000083.6	Rasgrp3	7.63E-01
gene32105	68021	NC_000079.6	Bphl	7.62E-01
gene13584	381695	NC_000071.6	N4bp2l2	7.62E-01
gene28922	1E+08	NC_000077.6	Gm34823	7.62E-01
gene7059	20319	NC_000069.6	Sfrp2	7.62E-01
gene38833	1E+08	NC_000083.6	Gm36117	7.62E-01
gene7455	229589	NC_000069.6	Prune	7.62E-01
gene3819	107435	NC_000068.7	Hat1	7.61E-01
gene17957	56546	NC_000073.6	Sec1	7.61E-01
gene4740	11834	NC_000068.7	Aqr	7.61E-01
gene32174	71340	NC_000079.6	Riok1	7.61E-01
gene29023	207165	NC_000077.6	Bptf	7.61E-01
gene25438	18541	NC_000076.6	Pcnt	7.61E-01
gene26936	216616	NC_000077.6	Efemp1	7.61E-01
gene31597	69562	NC_000079.6	Cdk13	7.60E-01
gene2397	109232	NC_000067.6	Sccpdh	7.59E-01
gene43151	414758	NC_000085.6	Zfp950	7.59E-01

gene21941	74347	NC_000074.6	Tldc1	7.59E-01
gene41865	54607	NC_000084.6	Socs6	7.59E-01
gene14047	338523	NC_000072.6	Kdm7a	7.58E-01
gene26110	71769	NC_000076.6	Bbs10	7.58E-01
gene33987	320234	NC_000080.6	Ccdc66	7.58E-01
gene37507	67451	NC_000082.6	Pkp2	7.57E-01
gene42966	18572	NC_000085.6	Pdcd11	7.57E-01
gene26592	170835	NC_000077.6	Inpp5j	7.57E-01
gene27946	16407	NC_000077.6	Itgae	7.57E-01
gene13469	264064	NC_000071.6	Cdk8	7.56E-01
gene3712	59029	NC_000068.7	Psmd14	7.56E-01
gene25503	28240	NC_000076.6	Trpm2	7.56E-01
gene29068	52639	NC_000077.6	Wipi1	7.55E-01
gene35571	76355	NC_000080.6	Tgds	7.55E-01
gene23175	110842	NC_000075.6	Etfa	7.55E-01
gene13583	100637	NC_000071.6	N4bp2l1	7.55E-01
gene40173	18140	NC_000083.6	Uhrf1	7.55E-01
gene14348	1E+08	NC_000072.6	Gimap1os	7.55E-01
gene2721	18108	NC_000068.7	Nmt2	7.55E-01
gene28346	327987	NC_000077.6	Med13	7.54E-01
gene38080	208177	NC_000082.6	Phldb2	7.54E-01
gene37349	19041	NC_000082.6	Ppl	7.54E-01
gene21535	16582	NC_000074.6	Kifc3	7.54E-01
gene39438	22092	NC_000083.6	Rsph1	7.54E-01
gene15129	69538	NC_000072.6	Antxr1	7.54E-01
gene3854	1.1E+08	NC_000068.7	Gm39850	7.54E-01
gene27504	67826	NC_000077.6	Snap47	7.54E-01
gene3096	14347	NC_000068.7	Fut7	7.53E-01
gene19359	12850	NC_000073.6	Coq7	7.53E-01
gene26579	=Gene;ger	NC_000077.6	LOC108167875	7.53E-01
gene26577	78887	NC_000077.6	Sfi1	7.53E-01
gene5632	70470	NC_000068.7	Rprd1b	7.53E-01
gene3711	21353	NC_000068.7	Tank	7.53E-01
gene6230	620570	NC_000069.6	Gm6162	7.53E-01
gene1158	98402	NC_000067.6	Sh3bp4	7.53E-01
gene32671	71508	NC_000079.6	Zfp935	7.53E-01
gene20865	12725	NC_000074.6	Clcn3	7.52E-01
gene9723	66526	NC_000070.6	Tceanc2	7.52E-01
gene42811	14296	NC_000085.6	Frat1	7.52E-01
gene15029	70527	NC_000072.6	Stambp	7.52E-01
gene38406	78913	NC_000082.6	Ltn1	7.52E-01
gene3689	57438	NC_000068.7	March7	7.52E-01
gene23594	50876	NC_000075.6	Tmod2	7.52E-01
gene13512	14254	NC_000071.6	Flt1	7.52E-01
gene30428	13877	NC_000078.6	Erh	7.51E-01
gene10571	230866	NC_000070.6	Emc1	7.50E-01
gene1299	227394	NC_000067.6	Slco4c1	7.50E-01
gene16648	381845	NC_000073.6	Rnf225	7.50E-01

gene29842	16777	NC_000078.6	Lamb1	7.50E-01
gene11591	107823	NC_000071.6	Whsc1	7.50E-01
gene12688	231637	NC_000071.6	Ssh1	7.50E-01
gene23266	102657	NC_000075.6	Cd276	7.50E-01
gene24448	22646	NC_000075.6	Zfp105	7.50E-01
gene24951	71713	NC_000076.6	Cdc40	7.50E-01
gene2963	1E+08	NC_000068.7	Gm17762	7.49E-01
gene39598	56299	NC_000083.6	Fkbpl	7.49E-01
gene25110	17155	NC_000076.6	Man1a	7.49E-01
gene25924	69878	NC_000076.6	Snrpf	7.49E-01
gene41413	77422	NC_000084.6	C330018D20Rik	7.48E-01
gene21730	21771	NC_000074.6	Cirh1a	7.48E-01
gene22645	20443	NC_000075.6	St3gal4	7.48E-01
gene10512	15530	NC_000070.6	Hspg2	7.47E-01
gene13870	192198	NC_000072.6	Lrrc4	7.47E-01
gene36351	11838	NC_000081.6	Arc	7.47E-01
gene16839	546936	NC_000073.6	Gm5997	7.47E-01
gene26651	75668	NC_000077.6	Rasl10a	7.47E-01
gene18439	17293	NC_000073.6	Mesp2	7.47E-01
gene40761	225164	NC_000084.6	Mib1	7.46E-01
gene17280	22718	NC_000073.6	Zfp60	7.46E-01
gene34348	16709	NC_000080.6	Ktn1	7.46E-01
gene23136	75717	NC_000075.6	Cul5	7.46E-01
gene19438	233824	NC_000073.6	Cog7	7.46E-01
gene9047	230157	NC_000070.6	Tmeff1	7.45E-01
gene4885	75894	NC_000068.7	Adal	7.45E-01
gene13969	664942	NC_000072.6	Gm13858	7.45E-01
gene20950	234358	NC_000074.6	Zfp930	7.45E-01
gene1785	56207	NC_000067.6	Uchl5	7.45E-01
gene28301	56405	NC_000077.6	Dusp14	7.45E-01
gene35095	105440	NC_000080.6	Kctd9	7.44E-01
gene24371	12776	NC_000075.6	Ccr8	7.43E-01
gene661	227154	NC_000067.6	Stradb	7.43E-01
gene38038	207806	NC_000082.6	Usf3	7.43E-01
gene500	12832	NC_000067.6	Col5a2	7.43E-01
gene22312	75723	NC_000075.6	Amotl1	7.43E-01
gene41806	17196	NC_000084.6	Mbp	7.43E-01
gene30022	76983	NC_000078.6	Scfd1	7.42E-01
gene38053	57781	NC_000082.6	Cd200r1	7.42E-01
gene20398	57312	NC_000074.6	Mrps31	7.41E-01
gene16282	1E+08	NC_000073.6	Gm14548	7.41E-01
gene12206	381650	NC_000071.6	Thap6	7.41E-01
gene18476	233410	NC_000073.6	Zfp592	7.40E-01
gene3060	241274	NC_000068.7	Pnpla7	7.40E-01
gene32228	110521	NC_000079.6	Hivep1	7.40E-01
gene29438	16570	NC_000078.6	Kif3c	7.40E-01
gene41370	225523	NC_000084.6	Cep120	7.40E-01
gene12898	19047	NC_000071.6	Ppp1cc	7.40E-01

gene39975	74094	NC_000083.6	Tjap1	7.40E-01
gene18251	233315	NC_000073.6	Mtmr10	7.40E-01
gene35416	72662	NC_000080.6	Dis3	7.39E-01
gene26108	237542	NC_000076.6	Osbpl8	7.39E-01
gene16163	71323	NC_000072.6	Rassf8	7.39E-01
gene25959	93765	NC_000076.6	Ube2n	7.39E-01
gene43106	71653	NC_000085.6	Shtn1	7.39E-01
gene35259	67554	NC_000080.6	Slc25a30	7.39E-01
gene24202	235626	NC_000075.6	Setd2	7.39E-01
gene15787	319801	NC_000072.6	Tigar	7.38E-01
gene27933	67128	NC_000077.6	Ube2g1	7.38E-01
gene33745	218734	NC_000080.6	3830406C13Rik	7.38E-01
gene30769	109181	NC_000078.6	Trip11	7.38E-01
gene727	1E+08	NC_000067.6	Ino80dos	7.38E-01
gene33986	218850	NC_000080.6	Fam208a	7.38E-01
gene37052	12455	NC_000081.6	Ccnt1	7.37E-01
gene29324	338371	NC_000077.6	Endov	7.37E-01
gene35923	239364	NC_000081.6	Tspyl5	7.37E-01
gene32397	1E+08	NC_000079.6	Gm2654	7.37E-01
gene13011	78894	NC_000071.6	Aacs	7.37E-01
gene30558	70373	NC_000078.6	Gpatch2l	7.37E-01
gene21939	67866	NC_000074.6	Wfdc1	7.37E-01
gene23466	=Gene;ger	NC_000075.6	LOC102633151	7.37E-01
gene37101	75284	NC_000081.6	Bcdin3d	7.36E-01
gene42956	66439	NC_000085.6	Borcs7	7.36E-01
gene35938	71637	NC_000081.6	4930413F20Rik	7.35E-01
gene36636	17444	NC_000081.6	Grap2	7.35E-01
gene12985	209357	NC_000071.6	Gtf2h3	7.35E-01
gene16301	108841	NC_000073.6	Rdh13	7.34E-01
gene17385	73729	NC_000073.6	Zfp383	7.34E-01
gene3311	320267	NC_000068.7	Fubp3	7.34E-01
gene31474	56043	NC_000079.6	Akr1e1	7.34E-01
gene8623	=Gene;ger	NC_000070.6	LOC108168933	7.34E-01
gene10634	213989	NC_000070.6	Tmem82	7.34E-01
gene6126	52856	NC_000068.7	Mtg2	7.33E-01
gene39466	18131	NC_000083.6	Notch3	7.33E-01
gene11978	433904	NC_000071.6	Ociad2	7.33E-01
gene33849	105428	NC_000080.6	Fam149b	7.33E-01
gene21693	320394	NC_000074.6	Cenpt	7.33E-01
gene21100	234413	NC_000074.6	Zfp961	7.33E-01
gene3365	227736	NC_000068.7	Cfap157	7.33E-01
gene26088	17931	NC_000076.6	Ppp1r12a	7.33E-01
gene3861	68526	NC_000068.7	Gpr155	7.32E-01
gene3904	ene;gene=L	NC_000068.7	LOC102635707	7.32E-01
gene37795	74143	NC_000082.6	Opa1	7.32E-01
gene4419	228357	NC_000068.7	Lrp4	7.32E-01
gene40972	225341	NC_000084.6	Lims2	7.31E-01
gene27656	78883	NC_000077.6	9630013K17Rik	7.31E-01

gene37108	26934	NC_000081.6	Racgap1	7.31E-01
gene21777	56749	NC_000074.6	Dhodh	7.30E-01
gene10629	22642	NC_000070.6	Zbtb17	7.30E-01
gene32342	66336	NC_000079.6	Cenpp	7.30E-01
gene24458	109332	NC_000075.6	Cdcp1	7.30E-01
gene35747	68646	NC_000081.6	Nadk2	7.30E-01
gene12412	17355	NC_000071.6	Aff1	7.30E-01
gene22030	12398	NC_000074.6	Cbfa2t3	7.30E-01
gene10388	242691	NC_000070.6	Gpatch3	7.30E-01
gene32701	19206	NC_000079.6	Ptch1	7.29E-01
gene20827	50753	NC_000074.6	Fbxo8	7.29E-01
gene26799	68145	NC_000077.6	Etaa1	7.29E-01
gene33754	218739	NC_000080.6	Sntn	7.29E-01
gene35898	67434	NC_000081.6	Ankrd33b	7.29E-01
gene37560	224023	NC_000082.6	Klhl22	7.29E-01
gene24612	66201	NC_000076.6	Vta1	7.29E-01
gene14940	621306	NC_000072.6	Gm6210	7.28E-01
gene10927	85030	NC_000070.6	Tnfrsf25	7.28E-01
gene41485	106894	NC_000084.6	Hmgxb3	7.28E-01
gene7921	76561	NC_000069.6	Snx7	7.28E-01
gene38064	1.1E+08	NC_000082.6	Gm41456	7.28E-01
gene33070	66671	NC_000079.6	Ccnh	7.28E-01
gene13071	69568	NC_000071.6	Vkorc1l1	7.27E-01
gene30212	66244	NC_000078.6	Nemf	7.27E-01
gene977	69368	NC_000067.6	Wdfy1	7.27E-01
gene38657	328734	NC_000083.6	Pisd-ps2	7.27E-01
gene37023	223870	NC_000081.6	Senp1	7.26E-01
gene7645	17156	NC_000069.6	Man1a2	7.26E-01
gene14430	54353	NC_000072.6	Skap2	7.25E-01
gene24332	13813	NC_000075.6	Eomes	7.25E-01
gene8600	192656	NC_000070.6	Ripk2	7.25E-01
gene41945	11854	NC_000085.6	Rhod	7.25E-01
gene7150	66614	NC_000069.6	Gpatch4	7.25E-01
gene42872	73689	NC_000085.6	Bloc1s2	7.25E-01
gene28776	66720	NC_000077.6	Klhl10	7.24E-01
gene28682	19401	NC_000077.6	Rara	7.24E-01
gene10231	1E+08	NC_000070.6	Gm33901	7.24E-01
gene1003	12828	NC_000067.6	Col4a3	7.23E-01
gene27582	57916	NC_000077.6	Tnfrsf13b	7.23E-01
gene26968	103554	NC_000077.6	Psme4	7.23E-01
gene40743	140792	NC_000084.6	Colec12	7.23E-01
gene25403	23887	NC_000076.6	Ggt5	7.23E-01
gene7883	58193	NC_000069.6	Extl2	7.23E-01
gene12208	53886	NC_000071.6	Cdkl2	7.23E-01
gene31052	217864	NC_000078.6	Rcor1	7.23E-01
gene39126	268930	NC_000083.6	Pkmyt1	7.22E-01
gene34067	27057	NC_000080.6	Ncoa4	7.22E-01
gene36939	223828	NC_000081.6	Pphln1	7.22E-01

gene22330	55991	NC_000075.6	Panx1	7.21E-01
gene1621	17248	NC_000067.6	Mdm4	7.21E-01
gene29522	75516	NC_000078.6	Ttc32	7.20E-01
gene1458	226352	NC_000067.6	Epb41l5	7.20E-01
gene26618	67815	NC_000077.6	Sec14l2	7.20E-01
gene40757	77805	NC_000084.6	Esco1	7.19E-01
gene6670	26570	NC_000069.6	Slc7a11	7.19E-01
gene41612	19255	NC_000084.6	Ptpn2	7.19E-01
gene23872	245007	NC_000075.6	Zbtb38	7.19E-01
gene2656	215243	NC_000067.6	Traf3ip3	7.19E-01
gene2998	1E+08	NC_000068.7	Gm3230	7.19E-01
gene13340	231842	NC_000071.6	Amz1	7.18E-01
gene20463	234135	NC_000074.6	Whsc1l1	7.18E-01
gene14378	57895	NC_000072.6	Ccdc126	7.18E-01
gene484	246229	NC_000067.6	Bivm	7.18E-01
gene30452	69792	NC_000078.6	Med6	7.18E-01
gene26970	66753	NC_000077.6	Erlec1	7.18E-01
gene1663	68507	NC_000067.6	Ppfia4	7.17E-01
gene4848	22174	NC_000068.7	Tyro3	7.17E-01
gene17474	17178	NC_000073.6	Fxyd3	7.17E-01
gene35055	74195	NC_000080.6	Elp3	7.17E-01
gene19488	74204	NC_000073.6	Xpo6	7.17E-01
gene11114	545725	NC_000071.6	Mterf1a	7.17E-01
gene24601	56535	NC_000076.6	Pex3	7.17E-01
gene16389	72667	NC_000073.6	Zfp444	7.17E-01
gene42033	69595	NC_000085.6	Frmd8os	7.16E-01
gene20537	68867	NC_000074.6	Rnf122	7.16E-01
gene1242	1E+08	NC_000067.6	9430060I03Rik	7.16E-01
gene32337	66329	NC_000079.6	Susd3	7.15E-01
gene6865	229363	NC_000069.6	Gmps	7.15E-01
gene41956	66704	NC_000085.6	Rbm4b	7.15E-01
gene26880	17846	NC_000077.6	Commd1	7.15E-01
gene15789	77558	NC_000072.6	9330179D12Rik	7.15E-01
gene7076	320782	NC_000069.6	Tmem154	7.15E-01
gene16084	1E+08	NC_000072.6	Gm30215	7.15E-01
gene36594	105785	NC_000081.6	Kdelr3	7.15E-01
gene42949	56350	NC_000085.6	Arl3	7.15E-01
gene42229	225913	NC_000085.6	Tkfc	7.14E-01
gene35271	27275	NC_000080.6	Nufip1	7.14E-01
gene18454	269955	NC_000073.6	Rccd1	7.13E-01
gene31448	105203	NC_000079.6	Fam208b	7.13E-01
gene13896	232670	NC_000072.6	Tspan33	7.13E-01
gene28175	18015	NC_000077.6	Nf1	7.13E-01
gene5849	66589	NC_000068.7	Ube2v1	7.12E-01
gene26390	71750	NC_000076.6	R3hdm2	7.12E-01
gene33850	1E+08	NC_000080.6	Gm30054	7.12E-01
gene24555	237256	NC_000076.6	Zc3h12d	7.12E-01
gene35715	=Gene;ger	NC_000081.6	LOC102632231	7.11E-01

gene10987	269614	NC_000070.6	Pank4	7.11E-01
gene39152	18607	NC_000083.6	Pdpk1	7.11E-01
gene39472	54194	NC_000083.6	Akap8l	7.11E-01
gene22966	214162	NC_000075.6	Kmt2a	7.11E-01
gene40164	66443	NC_000083.6	Tnfaip8l1	7.11E-01
gene37737	320099	NC_000082.6	BC106179	7.10E-01
gene32240	56705	NC_000079.6	Ranbp9	7.10E-01
gene15810	109246	NC_000072.6	Tspan9	7.10E-01
gene5429	12995	NC_000068.7	Csnk2a1	7.10E-01
gene42776	56454	NC_000085.6	Aldh18a1	7.10E-01
gene13775	=Gene;ger	NC_000072.6	LOC108167381	7.09E-01
gene42743	240660	NC_000085.6	Slc35g1	7.09E-01
gene1366	70750	NC_000067.6	Kdsr	7.09E-01
gene35767	75646	NC_000081.6	Rai14	7.08E-01
gene30136	20334	NC_000078.6	Sec23a	7.08E-01
gene26157	66816	NC_000076.6	Thap2	7.08E-01
gene6535	114893	NC_000069.6	Dcun1d1	7.08E-01
gene20391	56229	NC_000074.6	Thsd1	7.08E-01
gene13354	17425	NC_000071.6	Foxk1	7.08E-01
gene37823	78618	NC_000082.6	Acap2	7.07E-01
gene21405	214627	NC_000074.6	Papd5	7.07E-01
gene41674	114615	NC_000084.6	Elac1	7.07E-01
gene26945	76784	NC_000077.6	Mtif2	7.07E-01
gene31566	97884	NC_000079.6	B3galnt2	7.07E-01
gene13075	12909	NC_000071.6	Crcp	7.07E-01
gene12519	17765	NC_000071.6	Mtf2	7.07E-01
gene30179	66689	NC_000078.6	Klhl28	7.07E-01
gene28571	629750	NC_000077.6	Gm11517	7.07E-01
gene23505	66660	NC_000075.6	Sltm	7.06E-01
gene7418	76742	NC_000069.6	Snx27	7.06E-01
gene39070	224598	NC_000083.6	Zfp758	7.06E-01
gene33462	71690	NC_000079.6	Esm1	7.06E-01
gene12026	12753	NC_000071.6	Clock	7.05E-01
gene22125	382038	NC_000074.6	Urb2	7.05E-01
gene30458	54604	NC_000078.6	Pcnx	7.05E-01
gene30453	69480	NC_000078.6	Ttc9	7.05E-01
gene1525	72949	NC_000067.6	Ccnt2	7.05E-01
gene13236	231801	NC_000071.6	Agfg2	7.05E-01
gene24003	235567	NC_000075.6	Dnajc13	7.05E-01
gene8827	68926	NC_000070.6	Ubap2	7.04E-01
gene17888	1E+08	NC_000073.6	Gm15545	7.04E-01
gene40893	212307	NC_000084.6	Mapre2	7.04E-01
gene41475	545260	NC_000084.6	Arsi	7.04E-01
gene14893	69956	NC_000072.6	Ptcd3	7.04E-01
gene26794	103784	NC_000077.6	Wdr92	7.04E-01
gene34042	21924	NC_000080.6	Tnnc1	7.03E-01
gene20468	67207	NC_000074.6	Lsm1	7.03E-01
gene6097	626848	NC_000068.7	Zfp971	7.03E-01

gene18852	75212	NC_000073.6	Rnf121	7.03E-01
gene17649	12447	NC_000073.6	Ccne1	7.03E-01
gene27013	20868	NC_000077.6	Stk10	7.03E-01
gene22189	52202	NC_000074.6	Rbm34	7.03E-01
gene19672	434249	NC_000073.6	Gm5602	7.03E-01
gene26178	216363	NC_000076.6	Rab3ip	7.02E-01
gene26660	84035	NC_000077.6	Kremen1	7.02E-01
gene39041	22709	NC_000083.6	Zfp51	7.02E-01
gene1051	434484	NC_000067.6	Sp140	7.02E-01
gene634	51960	NC_000067.6	Kctd18	7.02E-01
gene13808	71777	NC_000072.6	Ing3	7.01E-01
gene8072	66350	NC_000069.6	Pla2g12a	7.01E-01
gene19828	212508	NC_000073.6	Mtg1	7.01E-01
gene41018	225358	NC_000084.6	Fam13b	7.00E-01
gene10091	230721	NC_000070.6	Pabpc4	7.00E-01
gene21655	74356	NC_000074.6	4931428F04Rik	7.00E-01
gene3058	67228	NC_000068.7	Dph7	7.00E-01
gene1086	18582	NC_000067.6	Pde6d	7.00E-01
gene39158	72016	NC_000083.6	1600002H07Rik	6.99E-01
gene7232	545539	NC_000069.6	Gm15417	6.99E-01
gene8957	50798	NC_000070.6	Gne	6.99E-01
gene16167	16651	NC_000072.6	Sspn	6.99E-01
gene25989	17022	NC_000076.6	Lum	6.99E-01
gene10385	69073	NC_000070.6	Kdf1	6.99E-01
gene40485	378462	NC_000083.6	Morn2	6.98E-01
gene14899	20454	NC_000072.6	St3gal5	6.97E-01
gene43077	226251	NC_000085.6	Ablim1	6.97E-01
gene23013	215051	NC_000075.6	Bud13	6.97E-01
gene20208	102323	NC_000074.6	Dcun1d2	6.97E-01
gene43002	170750	NC_000085.6	Xpnpep1	6.96E-01
gene18504	29877	NC_000073.6	Hdgfrp3	6.96E-01
gene17410	233066	NC_000073.6	Syne4	6.96E-01
gene12509	14581	NC_000071.6	Gfi1	6.96E-01
gene29447	13435	NC_000078.6	Dnmt3a	6.96E-01
gene1865	226517	NC_000067.6	Smg7	6.95E-01
gene25945	67723	NC_000076.6	Cep83os	6.95E-01
gene9040	67727	NC_000070.6	Stx17	6.95E-01
gene1527	226407	NC_000067.6	Rab3gap1	6.95E-01
gene30919	14026	NC_000078.6	Evl	6.95E-01
gene18701	622320	NC_000073.6	Kctd21	6.94E-01
gene25336	66859	NC_000076.6	Slc16a9	6.93E-01
gene24483	630836	NC_000075.6	2010315B03Rik	6.93E-01
gene15261	22026	NC_000072.6	Nr2c2	6.93E-01
gene24891	1E+08	NC_000076.6	Amd2	6.93E-01
gene40008	224826	NC_000083.6	Ubr2	6.93E-01
gene21312	18032	NC_000074.6	Nfix	6.93E-01
gene16303	320749	NC_000073.6	D630041G03Rik	6.92E-01
gene39496	240066	NC_000083.6	Zfp870	6.92E-01

gene27144	16428	NC_000077.6	Itk	6.92E-01
gene35418	12224	NC_000080.6	Klf5	6.91E-01
gene7825	56742	NC_000069.6	Psrc1	6.91E-01
gene6012	545486	NC_000068.7	Tubb1	6.91E-01
gene38753	75296	NC_000083.6	Fgfr1op	6.91E-01
gene39780	76416	NC_000083.6	Znrd1as	6.91E-01
gene32204	14538	NC_000079.6	Gcnt2	6.91E-01
gene10196	68310	NC_000070.6	Zmym1	6.91E-01
gene37991	76916	NC_000082.6	Timmdc1	6.90E-01
gene19623	233913	NC_000073.6	BC017158	6.90E-01
gene10842	654812	NC_000070.6	Angptl7	6.89E-01
gene39048	73229	NC_000083.6	3110052M02Rik	6.89E-01
gene27688	338369	NC_000077.6	Tmem220	6.89E-01
gene38512	104015	NC_000082.6	Synj1	6.89E-01
gene4020	21788	NC_000068.7	Tfpi	6.89E-01
gene34878	68043	NC_000080.6	Eef1akmt1	6.88E-01
gene18129	233280	NC_000073.6	Nipa1	6.88E-01
gene14548	232023	NC_000072.6	Vopp1	6.88E-01
gene39119	212733	NC_000083.6	Ccdc64b	6.88E-01
gene15796	101187	NC_000072.6	Parp11	6.88E-01
gene33279	1E+08	NC_000079.6	Ak6	6.88E-01
gene30415	69548	NC_000078.6	2310015A10Rik	6.88E-01
gene40618	73825	NC_000083.6	Ppp1r21	6.87E-01
gene19959	21953	NC_000073.6	Tnni2	6.87E-01
gene15819	22158	NC_000072.6	Tulp3	6.87E-01
gene30269	59036	NC_000078.6	Dact1	6.87E-01
gene41749	619308	NC_000084.6	F830208F22Rik	6.87E-01
gene27802	14166	NC_000077.6	Fgf11	6.87E-01
gene12588	675812	NC_000071.6	Zfp605	6.87E-01
gene28207	69077	NC_000077.6	Psmd11	6.86E-01
gene11541	269639	NC_000071.6	Zfp512	6.86E-01
gene40412	50850	NC_000083.6	Spast	6.86E-01
gene5769	170789	NC_000068.7	Acot8	6.86E-01
gene3860	74616	NC_000068.7	Scrn3	6.86E-01
gene40055	320148	NC_000083.6	B430306N03Rik	6.86E-01
gene34741	219072	NC_000080.6	Haus4	6.86E-01
gene27474	75429	NC_000077.6	Fam183b	6.85E-01
gene40014	64657	NC_000083.6	Mrps10	6.85E-01
gene7071	229474	NC_000069.6	Fhdc1	6.84E-01
gene34346	1E+08	NC_000080.6	Atg14	6.84E-01
gene39543	20084	NC_000083.6	Rps18	6.84E-01
gene12170	81702	NC_000071.6	Ankrd17	6.84E-01
gene36554	105844	NC_000081.6	Card10	6.84E-01
gene28503	217125	NC_000077.6	Samd14	6.84E-01
gene2058	1E+08	NC_000067.6	Gm16548	6.84E-01
gene39300	67458	NC_000083.6	Ergic1	6.84E-01
gene2168	80915	NC_000067.6	Dusp12	6.83E-01
gene17311	20055	NC_000073.6	Rps16	6.83E-01

gene33071	218397	NC_000079.6	Rasa1	6.83E-01
gene31979	14756	NC_000079.6	Gpld1	6.83E-01
gene12788	330177	NC_000071.6	Taok3	6.83E-01
gene15603	12122	NC_000072.6	Bid	6.82E-01
gene9758	69893	NC_000070.6	Coa7	6.82E-01
gene11653	320661	NC_000071.6	D5Ertd579e	6.82E-01
gene25747	544717	NC_000076.6	1190007I07Rik	6.81E-01
gene42771	54132	NC_000085.6	Pdlim1	6.81E-01
gene21993	234825	NC_000074.6	Klhdc4	6.80E-01
gene32721	105278	NC_000079.6	Cdk20	6.80E-01
gene3553	320872	NC_000068.7	Arhgap15os	6.80E-01
gene28433	18559	NC_000077.6	Pctp	6.79E-01
gene21843	234740	NC_000074.6	Tmem231	6.79E-01
gene3190	77794	NC_000068.7	Adamtsl2	6.79E-01
gene16312	68255	NC_000073.6	Tmem86b	6.79E-01
gene37315	52864	NC_000082.6	Slx4	6.79E-01
gene31137	104732	NC_000078.6	4930427A07Rik	6.78E-01
gene17285	545938	NC_000073.6	Zfp607	6.78E-01
gene40019	224833	NC_000083.6	AI661453	6.78E-01
gene33176	14063	NC_000079.6	F2rl1	6.78E-01
gene25644	208266	NC_000076.6	Dot1l	6.77E-01
gene25556	70427	NC_000076.6	Mier2	6.77E-01
gene33181	14064	NC_000079.6	F2rl2	6.77E-01
gene9405	69863	NC_000070.6	Ttc39b	6.77E-01
gene13325	231834	NC_000071.6	Snx8	6.76E-01
gene11486	28042	NC_000071.6	Ept1	6.76E-01
gene29158	14149	NC_000077.6	Fdxr	6.76E-01
gene33124	218442	NC_000079.6	Serinc5	6.75E-01
gene12227	269113	NC_000071.6	Nup54	6.75E-01
gene7247	16194	NC_000069.6	Il6ra	6.75E-01
gene7985	242202	NC_000069.6	Pde5a	6.74E-01
gene42729	107221	NC_000085.6	Ffar4	6.74E-01
gene21529	382045	NC_000074.6	Adgrg5	6.73E-01
gene23385	66131	NC_000075.6	Tipin	6.73E-01
gene1847	63913	NC_000067.6	Fam129a	6.73E-01
gene24356	208194	NC_000075.6	Exog	6.73E-01
gene23525	102371	NC_000075.6	Myzap	6.73E-01
gene7072	99889	NC_000069.6	Arfip1	6.73E-01
gene39980	57908	NC_000083.6	Zfp318	6.73E-01
gene5462	1E+08	NC_000068.7	Gm32057	6.72E-01
gene7161	58176	NC_000069.6	Rhbg	6.72E-01
gene26422	19075	NC_000076.6	Prim1	6.72E-01
gene647	18393	NC_000067.6	Orc2	6.72E-01
gene19306	66922	NC_000073.6	Rras2	6.72E-01
gene37906	107589	NC_000082.6	Mylk	6.72E-01
gene37994	67846	NC_000082.6	Tmem39a	6.72E-01
gene12540	15932	NC_000071.6	Idua	6.72E-01
gene8537	12448	NC_000070.6	Ccne2	6.71E-01

gene32619	69635	NC_000079.6	Dapk1	6.71E-01
gene17130	71997	NC_000073.6	Smg9	6.71E-01
gene7167	68957	NC_000069.6	Paqr6	6.71E-01
gene30034	328092	NC_000078.6	Dtd2	6.70E-01
gene31463	27384	NC_000079.6	Akr1c13	6.70E-01
gene36767	170736	NC_000081.6	Parvb	6.70E-01
gene12606	75560	NC_000071.6	Ep400	6.70E-01
gene19535	233877	NC_000073.6	Kctd13	6.70E-01
gene21947	234797	NC_000074.6	6430548M08Rik	6.70E-01
gene21513	67610	NC_000074.6	Rspry1	6.70E-01
gene9504	66902	NC_000070.6	Mtap	6.70E-01
gene36682	76457	NC_000081.6	Ccdc134	6.70E-01
gene19995	244237	NC_000073.6	Tnfrsf26	6.69E-01
gene17117	56707	NC_000073.6	Zfp111	6.69E-01
gene12155	68473	NC_000071.6	Mob1b	6.69E-01
gene28051	70987	NC_000077.6	4931413K12Rik	6.69E-01
gene19731	101437	NC_000073.6	Dhx32	6.69E-01
gene7433	109334	NC_000069.6	B230398E01Rik	6.68E-01
gene5553	74945	NC_000068.7	Acss2os	6.68E-01
gene32811	66410	NC_000079.6	Mterf3	6.68E-01
gene2569	226823	NC_000067.6	Kctd3	6.68E-01
gene19365	71517	NC_000073.6	9030624J02Rik	6.67E-01
gene3198	67382	NC_000068.7	Brd3	6.67E-01
gene14192	77574	NC_000072.6	Tcaf1	6.67E-01
gene21973	68790	NC_000074.6	Fendrr	6.67E-01
gene11133	74051	NC_000071.6	Steap2	6.67E-01
gene19800	1E+08	NC_000073.6	Gm33697	6.67E-01
gene25258	67345	NC_000076.6	Herc4	6.66E-01
gene12986	67978	NC_000071.6	Tctn2	6.66E-01
gene35248	67302	NC_000080.6	Zc3h13	6.66E-01
gene31055	22031	NC_000078.6	Traf3	6.66E-01
gene9942	381546	NC_000070.6	Ccdc24	6.66E-01
gene8158	19055	NC_000069.6	Ppp3ca	6.66E-01
gene23096	244864	NC_000075.6	Layn	6.65E-01
gene19903	54006	NC_000073.6	Deaf1	6.65E-01
gene5225	319909	NC_000068.7	Ism1	6.64E-01
gene18725	66190	NC_000073.6	Acer3	6.64E-01
gene32845	235907	NC_000079.6	Zfp65	6.64E-01
gene39360	21678	NC_000083.6	Tead3	6.64E-01
gene20096	665021	NC_000074.6	Gm7451	6.63E-01
gene8350	1E+08	NC_000069.6	Gm31881	6.63E-01
gene8598	27354	NC_000070.6	Nbn	6.63E-01
gene10941	16498	NC_000070.6	Kcnab2	6.63E-01
gene6314	69126	NC_000069.6	1810022K09Rik	6.62E-01
gene36353	16469	NC_000081.6	Jrk	6.62E-01
gene7724	71887	NC_000069.6	Ppm1j	6.62E-01
gene42571	381218	NC_000085.6	4430402I18Rik	6.62E-01
gene1387	20725	NC_000067.6	Serpinb8	6.62E-01

gene3316	11350	NC_000068.7	Abl1	6.61E-01
gene15120	23885	NC_000072.6	Gmcl1	6.61E-01
gene36284	213068	NC_000081.6	Tmem71	6.61E-01
gene3700	56878	NC_000068.7	Rbms1	6.61E-01
gene30590	1E+08	NC_000078.6	Gm33444	6.61E-01
gene17453	233079	NC_000073.6	Ffar2	6.60E-01
gene25598	=Gene;ger	NC_000076.6	LOC108167824	6.60E-01
gene23499	71602	NC_000075.6	Myo1e	6.60E-01
gene3990	68082	NC_000068.7	Dusp19	6.60E-01
gene7878	68161	NC_000069.6	A930005H10Rik	6.60E-01
gene32824	1E+08	NC_000079.6	F630042J09Rik	6.59E-01
gene9840	17301	NC_000070.6	Foxd2	6.59E-01
gene14458	58875	NC_000072.6	Hibadh	6.59E-01
gene30874	1.1E+08	NC_000078.6	Gm40566	6.59E-01
gene7459	84505	NC_000069.6	Setdb1	6.59E-01
gene28150	52466	NC_000077.6	Slc46a1	6.59E-01
gene8539	381510	NC_000070.6	Dpy19l4	6.58E-01
gene18845	791260	NC_000073.6	Tomt	6.58E-01
gene12333	1.1E+08	NC_000071.6	Gm42123	6.58E-01
gene37949	106347	NC_000082.6	Ildr1	6.58E-01
gene21089	74015	NC_000074.6	Fcho1	6.57E-01
gene28476	76408	NC_000077.6	Abcc3	6.57E-01
gene12957	243270	NC_000071.6	Hcar1	6.57E-01
gene18391	75547	NC_000073.6	Akap13	6.57E-01
gene6139	14464	NC_000068.7	Gata5	6.57E-01
gene22211	72139	NC_000074.6	2610044O15Rik8	6.57E-01
gene20052	69697	NC_000074.6	Camsap3	6.57E-01
gene13400	15467	NC_000071.6	Eif2ak1	6.56E-01
gene40983	26405	NC_000084.6	Map3k2	6.56E-01
gene13176	66801	NC_000071.6	Prkrip1	6.56E-01
gene30474	217695	NC_000078.6	Zfyve1	6.56E-01
gene36513	666661	NC_000081.6	Gm8221	6.56E-01
gene26322	14479	NC_000076.6	Usp15	6.55E-01
gene5568	16328	NC_000068.7	Cep250	6.55E-01
gene6099	353208	NC_000068.7	Zfp931	6.55E-01
gene511	53945	NC_000067.6	Slc40a1	6.55E-01
gene1165	1.1E+08	NC_000067.6	Gm41914	6.55E-01
gene28991	72149	NC_000077.6	Strada	6.55E-01
gene25074	=Gene;ger	NC_000076.6	LOC108167810	6.55E-01
gene3444	ene;gene=L	NC_000068.7	LOC108168747	6.54E-01
gene38637	17857	NC_000082.6	Mx1	6.54E-01
gene33278	19356	NC_000079.6	Rad17	6.54E-01
gene34763	13644	NC_000080.6	Efs	6.54E-01
gene21990	142682	NC_000074.6	Zcchc14	6.53E-01
gene41082	108857	NC_000084.6	Ankhd1	6.53E-01
gene5952	20878	NC_000068.7	Aurka	6.53E-01
gene2264	16513	NC_000067.6	Kcnj10	6.53E-01
gene16387	67109	NC_000073.6	Zfp787	6.53E-01

gene3137	67991	NC_000068.7	Nacc2	6.53E-01
gene20045	69129	NC_000074.6	Pex11g	6.53E-01
gene23580	17918	NC_000075.6	Myo5a	6.52E-01
gene39042	24132	NC_000083.6	Zfp53	6.52E-01
gene10541	212190	NC_000070.6	Ubxn10	6.52E-01
gene13712	252875	NC_000072.6	Mios	6.52E-01
gene25023	11793	NC_000076.6	Atg5	6.52E-01
gene28591	71240	NC_000077.6	Osbpl7	6.52E-01
gene12850	252972	NC_000071.6	Tpcn1	6.52E-01
gene11006	1E+08	NC_000070.6	Gm16023	6.52E-01
gene29506	58240	NC_000078.6	Hs1bp3	6.51E-01
gene34818	277154	NC_000080.6	Nynrin	6.51E-01
gene38189	78749	NC_000082.6	Filip1l	6.51E-01
gene18159	22215	NC_000073.6	Ube3a	6.51E-01
gene28248	217012	NC_000077.6	Unc45b	6.50E-01
gene36484	223669	NC_000081.6	Zfp7	6.50E-01
gene23462	75697	NC_000075.6	C2cd4b	6.50E-01
gene24602	66757	NC_000076.6	Adat2	6.50E-01
gene27884	54189	NC_000077.6	Rabep1	6.50E-01
gene23446	208117	NC_000075.6	Aph1b	6.50E-01
gene14265	101095	NC_000072.6	Zfp282	6.49E-01
gene10616	213499	NC_000070.6	Fbxo42	6.49E-01
gene32349	105148	NC_000079.6	Iars	6.49E-01
gene20875	234309	NC_000074.6	Cbr4	6.49E-01
gene30515	19300	NC_000078.6	Abcd4	6.48E-01
gene4423	14061	NC_000068.7	F2	6.48E-01
gene5796	228876	NC_000068.7	Zfp334	6.48E-01
gene30100	=Gene;ger	NC_000078.6	LOC108168001	6.48E-01
gene42705	107182	NC_000085.6	Btaf1	6.48E-01
gene22496	319899	NC_000075.6	Dock6	6.48E-01
gene33335	238831	NC_000079.6	Ppwd1	6.48E-01
gene19713	77938	NC_000073.6	Fam53b	6.47E-01
gene11111	70797	NC_000071.6	Ankib1	6.47E-01
gene19398	12971	NC_000073.6	Crym	6.46E-01
gene10424	100163	NC_000070.6	Pafah2	6.46E-01
gene35856	66270	NC_000081.6	Fam134b	6.46E-01
gene13230	71176	NC_000071.6	Fbxo24	6.46E-01
gene2364	23797	NC_000067.6	Akt3	6.46E-01
gene28088	237859	NC_000077.6	Nsrp1	6.46E-01
gene36421	332110	NC_000081.6	Mapk15	6.46E-01
gene40398	57440	NC_000083.6	Ehd3	6.46E-01
gene25707	1E+08	NC_000076.6	Gm32024	6.45E-01
gene26274	320183	NC_000076.6	Msrb3	6.45E-01
gene12969	19679	NC_000071.6	Pitpnm2	6.45E-01
gene13205	1.1E+08	NC_000071.6	Gm40349	6.45E-01
gene24266	72179	NC_000075.6	Fbxl2	6.44E-01
gene23725	21983	NC_000075.6	Tpbg	6.44E-01
gene11712	100972	NC_000071.6	Rab28	6.44E-01

gene25037	1E+08	NC_000076.6	Gm15934	6.44E-01
gene20964	620419	NC_000074.6	Zfp963	6.44E-01
gene8713	26885	NC_000070.6	Casp8ap2	6.44E-01
gene24705	ene=LOC1	NC_000076.6	LOC108167767	6.43E-01
gene5808	72043	NC_000068.7	Sulf2	6.43E-01
gene13751	101148	NC_000072.6	Bmt2	6.43E-01
gene39941	224805	NC_000083.6	Aars2	6.43E-01
gene19785	69546	NC_000073.6	Mapk1ip1	6.42E-01
gene26674	76508	NC_000077.6	Ube2d-ps	6.42E-01
gene29620	68775	NC_000078.6	Atp6v1c2	6.42E-01
gene38075	14525	NC_000082.6	Gcsam	6.42E-01
gene3840	66953	NC_000068.7	Cdca7	6.42E-01
gene24276	12091	NC_000075.6	Glb1	6.42E-01
gene26035	237500	NC_000076.6	Tmtc3	6.42E-01
gene37341	72615	NC_000082.6	Anks3	6.42E-01
gene37419	106200	NC_000082.6	Txndc11	6.41E-01
gene19870	64384	NC_000073.6	Sirt3	6.41E-01
gene3240	227682	NC_000068.7	Trub2	6.41E-01
gene37951	57738	NC_000082.6	Slc15a2	6.40E-01
gene20406	18970	NC_000074.6	Polb	6.40E-01
gene9846	21349	NC_000070.6	Tal1	6.40E-01
gene33451	218630	NC_000079.6	Ccno	6.40E-01
gene32940	20499	NC_000079.6	Slc12a7	6.40E-01
gene38258	68159	NC_000082.6	Stx19	6.39E-01
gene39245	214901	NC_000083.6	Chtf18	6.39E-01
gene12465	231549	NC_000071.6	Lrrc8d	6.39E-01
gene21284	19724	NC_000074.6	Rfx1	6.39E-01
gene17253	233011	NC_000073.6	Itpkc	6.39E-01
gene4849	29808	NC_000068.7	Mga	6.39E-01
gene13913	232679	NC_000072.6	Zc3hc1	6.38E-01
gene37947	12524	NC_000082.6	Cd86	6.38E-01
gene8837	71901	NC_000070.6	Fam219a	6.38E-01
gene33188	67463	NC_000079.6	Poc5	6.38E-01
gene4575	12509	NC_000068.7	Cd59a	6.37E-01
gene39597	260297	NC_000083.6	Prrt1	6.37E-01
gene21322	71846	NC_000074.6	Syce2	6.37E-01
gene5022	1.1E+08	NC_000068.7	Gm39926	6.36E-01
gene27605	56697	NC_000077.6	Akap10	6.36E-01
gene2904	1.1E+08	NC_000068.7	Gm39760	6.36E-01
gene13408	231876	NC_000071.6	Lmtk2	6.36E-01
gene24981	56484	NC_000076.6	Foxo3	6.36E-01
gene10865	63958	NC_000070.6	Ube4b	6.36E-01
gene24407	67095	NC_000075.6	Trak1	6.36E-01
gene12980	75695	NC_000071.6	Rilpl1	6.36E-01
gene3579	26428	NC_000068.7	Orc4	6.35E-01
gene24649	64058	NC_000076.6	Perp	6.35E-01
gene4890	327655	NC_000068.7	Ppip5k1	6.35E-01
gene3230	22130	NC_000068.7	Ttf1	6.34E-01

gene25287	641340	NC_000076.6	Nrbf2	6.34E-01
gene17301	14113	NC_000073.6	Fbl	6.34E-01
gene20692	18536	NC_000074.6	Pcm1	6.34E-01
gene13899	74340	NC_000072.6	Ahcyl2	6.33E-01
gene29408	238023	NC_000077.6	Hexdc	6.33E-01
gene7269	229542	NC_000069.6	Gatad2b	6.33E-01
gene10923	100090	NC_000070.6	Zbtb48	6.32E-01
gene3062	ene=LOC1	NC_000068.7	LOC102638359	6.32E-01
gene6794	72033	NC_000069.6	Tsc22d2	6.32E-01
gene40974	574402	NC_000084.6	Gpr17	6.32E-01
gene25236	74706	NC_000076.6	4930507D05Rik	6.32E-01
gene10149	70088	NC_000070.6	Meaf6	6.32E-01
gene29557	76820	NC_000078.6	Fam49a	6.32E-01
gene19544	110033	NC_000073.6	Kif22	6.32E-01
gene5650	109275	NC_000068.7	Actr5	6.32E-01
gene40917	68591	NC_000084.6	Mocos	6.31E-01
gene33789	59007	NC_000080.6	Ngly1	6.31E-01
gene39435	21784	NC_000083.6	Tff1	6.31E-01
gene36190	67731	NC_000081.6	Fbxo32	6.31E-01
gene357	109346	NC_000067.6	Ankrd39	6.31E-01
gene39498	170716	NC_000083.6	Cyp4f13	6.31E-01
gene15628	50928	NC_000072.6	Klrg1	6.31E-01
gene40112	18519	NC_000083.6	Kat2b	6.31E-01
gene30028	11782	NC_000078.6	Ap4s1	6.30E-01
gene9063	230162	NC_000070.6	Zfp189	6.30E-01
gene18847	69358	NC_000073.6	Lrrc51	6.30E-01
gene9901	68394	NC_000070.6	Ccdc163	6.30E-01
gene2755	71648	NC_000068.7	Optn	6.29E-01
gene637	71724	NC_000067.6	Aox3	6.29E-01
gene38028	1E+08	NC_000082.6	Tigit	6.29E-01
gene21143	70823	NC_000074.6	Hmgxb4	6.29E-01
gene40990	71683	NC_000084.6	Gypc	6.29E-01
gene41060	73449	NC_000084.6	1700066B19Rik	6.28E-01
gene35795	268783	NC_000081.6	Mtmr12	6.28E-01
gene22906	102644	NC_000075.6	Oaf	6.28E-01
gene27085	1E+08	NC_000077.6	Hmgb1-ps1	6.28E-01
gene34817	73181	NC_000080.6	Nfatc4	6.28E-01
gene21684	234695	NC_000074.6	Rltpr	6.27E-01
gene36621	20972	NC_000081.6	Syngr1	6.27E-01
gene40246	67993	NC_000083.6	Nudt12	6.27E-01
gene3870	11909	NC_000068.7	Atf2	6.27E-01
gene42895	226151	NC_000085.6	Fam178a	6.26E-01
gene14624	414084	NC_000072.6	Tnip3	6.26E-01
gene19827	212503	NC_000073.6	Paox	6.26E-01
gene38000	22268	NC_000082.6	Upk1b	6.26E-01
gene41016	53871	NC_000084.6	Pkd2l2	6.26E-01
gene29569	18109	NC_000078.6	Mycn	6.26E-01
gene21302	67736	NC_000074.6	Ccdc130	6.25E-01

gene29415	238024	NC_000077.6	Fn3krp	6.25E-01
gene20727	212326	NC_000074.6	Fam149a	6.25E-01
gene7669	242125	NC_000069.6	Mab21l3	6.25E-01
gene28637	72194	NC_000077.6	Fbxl20	6.25E-01
gene7799	68312	NC_000069.6	Gstm7	6.25E-01
gene35764	19355	NC_000081.6	Rad1	6.25E-01
gene10735	1.1E+08	NC_000070.6	Gm42343	6.24E-01
gene5380	12497	NC_000068.7	Entpd6	6.24E-01
gene40676	26410	NC_000084.6	Map3k8	6.24E-01
gene26677	54125	NC_000077.6	Polm	6.24E-01
gene7483	70767	NC_000069.6	Prpf3	6.24E-01
gene40380	78785	NC_000083.6	Clip4	6.24E-01
gene13294	433956	NC_000071.6	Dnaaf5	6.23E-01
gene35467	13618	NC_000080.6	Ednrb	6.23E-01
gene1610	213452	NC_000067.6	Dstyk	6.23E-01
gene24742	26464	NC_000076.6	Vnn3	6.23E-01
gene20095	233987	NC_000074.6	Zfp958	6.23E-01
gene42253	69369	NC_000085.6	1700017D01Rik	6.23E-01
gene19350	233789	NC_000073.6	Smg1	6.23E-01
gene10667	110593	NC_000070.6	Prdm2	6.23E-01
gene15482	232334	NC_000072.6	Vgll4	6.23E-01
gene28368	66140	NC_000077.6	Ska2	6.22E-01
gene24023	75669	NC_000075.6	Pik3r4	6.22E-01
gene12539	110524	NC_000071.6	Dgkq	6.22E-01
gene1311	18484	NC_000067.6	Pam	6.22E-01
gene26974	68044	NC_000077.6	Chac2	6.22E-01
gene6541	241944	NC_000069.6	D3Ertd254e	6.22E-01
gene21403	234549	NC_000074.6	Heatr3	6.22E-01
gene5607	29812	NC_000068.7	Ndrg3	6.21E-01
gene11592	24116	NC_000071.6	Nelfa	6.21E-01
gene32847	22746	NC_000079.6	Zfp85	6.20E-01
gene12802	231670	NC_000071.6	Fbxo21	6.20E-01
gene11393	231050	NC_000071.6	Galnt11	6.20E-01
gene19420	26939	NC_000073.6	Polr3e	6.20E-01
gene28365	66569	NC_000077.6	Gdpd1	6.20E-01
gene33519	18115	NC_000079.6	Nnt	6.20E-01
gene15634	108653	NC_000072.6	Rimklb	6.20E-01
gene691	77951	NC_000067.6	Cyp20a1	6.19E-01
gene306	=Gene;ger	NC_000067.6	LOC108167617	6.18E-01
gene37961	74197	NC_000082.6	Gtf2e1	6.18E-01
gene6350	59058	NC_000069.6	Bhlhe22	6.18E-01
gene41175	225392	NC_000084.6	Rell2	6.18E-01
gene6937	68725	NC_000069.6	1110032F04Rik	6.18E-01
gene16858	243864	NC_000073.6	Mill2	6.18E-01
gene41176	319262	NC_000084.6	Fchsd1	6.18E-01
gene28959	16416	NC_000077.6	Itgb3	6.17E-01
gene15137	232201	NC_000072.6	Arhgap25	6.17E-01
gene42630	23972	NC_000085.6	Papss2	6.16E-01

gene34307	114874	NC_000080.6	Ddhd1	6.16E-01
gene29348	=Gene;ger	NC_000077.6	LOC105246895	6.16E-01
gene17140	66071	NC_000073.6	Ethe1	6.16E-01
gene40050	58217	NC_000083.6	Trem1	6.16E-01
gene7156	76642	NC_000069.6	1700113A16Rik	6.15E-01
gene39500	224691	NC_000083.6	Zfp472	6.15E-01
gene28505	1E+08	NC_000077.6	Gm11513	6.14E-01
gene25407	104248	NC_000076.6	Cabin1	6.14E-01
gene43131	76539	NC_000085.6	Fam204a	6.14E-01
gene1309	227399	NC_000067.6	Ppip5k2	6.14E-01
gene12040	231327	NC_000071.6	Ppat	6.13E-01
gene37583	94223	NC_000082.6	Dgcr8	6.13E-01
gene10853	=Gene;ger	NC_000070.6	LOC102635786	6.13E-01
gene2210	12355	NC_000067.6	Nr1i3	6.13E-01
gene7576	75888	NC_000069.6	4930573H18Rik	6.13E-01
gene893	77264	NC_000067.6	Zfp142	6.13E-01
gene35630	68889	NC_000080.6	Ubac2	6.13E-01
gene24181	54369	NC_000075.6	Nme6	6.12E-01
gene36582	18693	NC_000081.6	Pick1	6.12E-01
gene14271	72306	NC_000072.6	Zfp777	6.12E-01
gene25677	17179	NC_000076.6	Matk	6.12E-01
gene7408	19885	NC_000069.6	Rorc	6.12E-01
gene29301	12416	NC_000077.6	Cbx2	6.11E-01
gene7710	229675	NC_000069.6	Rsbn1	6.11E-01
gene28235	20290	NC_000077.6	Ccl1	6.11E-01
gene15238	54563	NC_000072.6	Nup210	6.11E-01
gene9714	230579	NC_000070.6	Fam151a	6.11E-01
gene9016	381605	NC_000070.6	Tbc1d2	6.10E-01
gene19591	233902	NC_000073.6	Fbxl19	6.10E-01
gene39937	71702	NC_000083.6	Cdc5l	6.10E-01
gene22306	75747	NC_000075.6	Sesn3	6.09E-01
gene25766	216190	NC_000076.6	Appl2	6.09E-01
gene28816	69847	NC_000077.6	Wnk4	6.09E-01
gene36746	12124	NC_000081.6	Bik	6.09E-01
gene6163	58237	NC_000068.7	Nkain4	6.09E-01
gene12809	76792	NC_000071.6	2410131K14Rik	6.09E-01
gene19364	101565	NC_000073.6	Ccp110	6.08E-01
gene10292	619605	NC_000070.6	Zcchc17	6.08E-01
gene33122	218441	NC_000079.6	Zfyve16	6.08E-01
gene40687	1E+08	NC_000084.6	Gm34326	6.08E-01
gene6381	20585	NC_000069.6	Hltf	6.07E-01
gene36892	58234	NC_000081.6	Shank3	6.07E-01
gene8257	ene=LOC1	NC_000069.6	LOC102637878	6.07E-01
gene19446	18817	NC_000073.6	Plk1	6.07E-01
gene38118	1E+08	NC_000082.6	Gm15518	6.07E-01
gene39207	30957	NC_000083.6	Mapk8ip3	6.06E-01
gene22693	66279	NC_000075.6	Tmem218	6.06E-01
gene7131	74485	NC_000069.6	Lrrc71	6.06E-01

gene32836	238693	NC_000079.6	Zfp58	6.06E-01
gene29278	71776	NC_000077.6	Tha1	6.06E-01
gene3797	68795	NC_000068.7	Ubr3	6.06E-01
gene17356	233046	NC_000073.6	Rasgrp4	6.05E-01
gene21833	170737	NC_000074.6	Znrf1	6.05E-01
gene39469	57261	NC_000083.6	Brd4	6.04E-01
gene36571	26912	NC_000081.6	Gcat	6.04E-01
gene22931	12402	NC_000075.6	Cbl	6.04E-01
gene11902	74734	NC_000071.6	Rhoh	6.04E-01
gene13492	140887	NC_000071.6	Lnx2	6.04E-01
gene19079	74349	NC_000073.6	Fam160a2	6.04E-01
gene39216	1E+08	NC_000083.6	Gm38655	6.04E-01
gene34051	67011	NC_000080.6	Mettl6	6.04E-01
gene23134	244879	NC_000075.6	Npat	6.04E-01
gene2154	18214	NC_000067.6	Ddr2	6.03E-01
gene37132	1E+08	NC_000081.6	Gm33666	6.03E-01
gene1464	1E+08	NC_000067.6	Gm28209	6.03E-01
gene2026	71449	NC_000067.6	Mettl13	6.03E-01
gene8356	68810	NC_000069.6	Nexn	6.03E-01
gene35853	17909	NC_000081.6	Myo10	6.03E-01
gene25089	22639	NC_000076.6	Zfa-ps	6.02E-01
gene28276	118453	NC_000077.6	Mmp28	6.02E-01
gene6312	71710	NC_000069.6	Lrrcc1	6.02E-01
gene4428	241547	NC_000068.7	Harbi1	6.02E-01
gene12464	1.1E+08	NC_000071.6	Gm42166	6.02E-01
gene10143	192199	NC_000070.6	Rspo1	6.02E-01
gene18147	434189	NC_000073.6	Gm5596	6.02E-01
gene30221	20663	NC_000078.6	Sos2	6.01E-01
gene8358	215708	NC_000069.6	Fam73a	6.01E-01
gene10322	269587	NC_000070.6	Epb41	6.00E-01
gene22549	244745	NC_000075.6	Dpy19l1	6.00E-01
gene15154	232210	NC_000072.6	Hmces	6.00E-01
gene35041	219150	NC_000080.6	Hmbox1	6.00E-01
gene39068	631624	NC_000083.6	Gm7072	6.00E-01
gene40560	19043	NC_000083.6	Ppm1b	5.99E-01
gene42819	226122	NC_000085.6	Ubtd1	5.99E-01
gene28629	72324	NC_000077.6	Plxdc1	5.99E-01
gene14040	1E+08	NC_000072.6	Gm33767	5.99E-01
gene13989	70699	NC_000072.6	Nup205	5.99E-01
gene8641	66625	NC_000070.6	Pnisr	5.99E-01
gene41236	225432	NC_000084.6	Rbm27	5.98E-01
gene11801	246316	NC_000071.6	Lgi2	5.98E-01
gene23428	56404	NC_000075.6	Trip4	5.98E-01
gene13166	74198	NC_000071.6	Dtx2	5.98E-01
gene36987	105727	NC_000081.6	Slc38a1	5.98E-01
gene10555	1.1E+08	NC_000070.6	Gm42333	5.98E-01
gene30775	217835	NC_000078.6	Rin3	5.97E-01
gene9702	329908	NC_000070.6	Usp24	5.97E-01

gene5601	228839	NC_000068.7	Tgif2	5.97E-01
gene15227	80292	NC_000072.6	Zxdc	5.97E-01
gene23265	319477	NC_000075.6	6030419C18Rik	5.97E-01
gene8525	67157	NC_000070.6	2610301B20Rik	5.97E-01
gene15028	1.1E+08	NC_000072.6	Gm38844	5.96E-01
gene40874	67664	NC_000084.6	Rnf125	5.96E-01
gene8173	26377	NC_000069.6	Dapp1	5.96E-01
gene11106	269623	NC_000071.6	Rbm48	5.96E-01
gene39212	71718	NC_000083.6	Telo2	5.96E-01
gene20465	72108	NC_000074.6	Ddhd2	5.96E-01
gene23444	235441	NC_000075.6	Usp3	5.95E-01
gene8448	116940	NC_000070.6	Tgs1	5.95E-01
gene10352	14050	NC_000070.6	Eya3	5.95E-01
gene20777	52357	NC_000074.6	Wwc2	5.95E-01
gene7454	171388	NC_000069.6	Bnipl	5.95E-01
gene12332	110075	NC_000071.6	Bmp3	5.95E-01
gene30579	217734	NC_000078.6	Pomt2	5.94E-01
gene27350	20520	NC_000077.6	Slc22a5	5.94E-01
gene19305	233744	NC_000073.6	Spon1	5.94E-01
gene12973	72650	NC_000071.6	2810006K23Rik	5.94E-01
gene34044	71838	NC_000080.6	Phf7	5.94E-01
gene18786	67967	NC_000073.6	Pold3	5.93E-01
gene40413	210148	NC_000083.6	Slc30a6	5.93E-01
gene11566	215476	NC_000071.6	Prr14l	5.93E-01
gene18704	21835	NC_000073.6	Thrsp	5.93E-01
gene33841	14702	NC_000080.6	Gng2	5.92E-01
gene41977	225870	NC_000085.6	Rin1	5.92E-01
gene15047	18139	NC_000072.6	Zfp638	5.92E-01
gene15508	17193	NC_000072.6	Mbd4	5.92E-01
gene10877	230917	NC_000070.6	Tmem201	5.92E-01
gene35258	338337	NC_000080.6	Cog3	5.92E-01
gene25870	237436	NC_000076.6	Gas2l3	5.92E-01
gene17798	69540	NC_000073.6	Klk10	5.92E-01
gene39354	224656	NC_000083.6	Zfp523	5.91E-01
gene14446	15404	NC_000072.6	Hoxa7	5.91E-01
gene8585	72519	NC_000070.6	Tmem55a	5.91E-01
gene25235	338359	NC_000076.6	Supv3l1	5.91E-01
gene3490	319817	NC_000068.7	Rc3h2	5.91E-01
gene15111	381792	NC_000072.6	2310040G24Rik	5.89E-01
gene32491	14186	NC_000079.6	Fgfr4	5.89E-01
gene6635	269424	NC_000069.6	Jade1	5.89E-01
gene18452	233405	NC_000073.6	Vps33b	5.89E-01
gene42973	14218	NC_000085.6	Sh3pxd2a	5.89E-01
gene38703	=Gene;ger	NC_000083.6	LOC108168335	5.89E-01
gene10443	12399	NC_000070.6	Runx3	5.89E-01
gene26685	1.1E+08	NC_000077.6	Gm40812	5.89E-01
gene32345	66695	NC_000079.6	Aspn	5.88E-01
gene24413	215474	NC_000075.6	Sec22c	5.88E-01

gene11014	108888	NC_000070.6	Atad3a	5.88E-01
gene3616	51869	NC_000068.7	Rif1	5.88E-01
gene22402	244713	NC_000075.6	Zfp317	5.88E-01
gene18717	18479	NC_000073.6	Pak1	5.87E-01
gene16774	71723	NC_000073.6	Dhx34	5.87E-01
gene19842	622973	NC_000073.6	Gm6376	5.87E-01
gene13424	100683	NC_000071.6	Trrap	5.86E-01
gene7946	213603	NC_000069.6	Slc44a3	5.86E-01
gene29231	217342	NC_000077.6	Ube2o	5.86E-01
gene27506	237775	NC_000077.6	Zfp867	5.86E-01
gene26872	103765	NC_000077.6	Tmem17	5.86E-01
gene7370	99681	NC_000069.6	Tchh	5.86E-01
gene4860	271844	NC_000068.7	Pla2g4f	5.86E-01
gene21169	74475	NC_000074.6	4933431K23Rik	5.85E-01
gene38706	68842	NC_000083.6	Tulp4	5.85E-01
gene28415	11640	NC_000077.6	Akap1	5.84E-01
gene42580	59028	NC_000085.6	Rcl1	5.84E-01
gene19149	244198	NC_000073.6	Olfml1	5.84E-01
gene39898	83815	NC_000083.6	Cenpq	5.83E-01
gene42195	66514	NC_000085.6	Asrgl1	5.83E-01
gene30532	217716	NC_000078.6	Mlh3	5.83E-01
gene30756	1E+08	NC_000078.6	A630072L19Rik	5.82E-01
gene19885	330671	NC_000073.6	B4galnt4	5.82E-01
gene2060	116914	NC_000067.6	Slc19a2	5.82E-01
gene40034	21425	NC_000083.6	Tfeb	5.82E-01
gene37694	66664	NC_000082.6	Tmem41a	5.82E-01
gene16773	17537	NC_000073.6	Meis3	5.82E-01
gene37883	69707	NC_000082.6	Iqcg	5.82E-01
gene40147	240120	NC_000083.6	Zfp119b	5.82E-01
gene39969	80905	NC_000083.6	Polh	5.82E-01
gene36782	73167	NC_000081.6	Arhgap8	5.81E-01
gene38566	209195	NC_000082.6	Clic6	5.81E-01
gene35215	70086	NC_000080.6	Cysltr2	5.81E-01
gene19280	320878	NC_000073.6	Mical2	5.81E-01
gene10567	212555	NC_000070.6	Pqlc2	5.80E-01
gene33424	26401	NC_000079.6	Map3k1	5.80E-01
gene41949	18563	NC_000085.6	Pcx	5.79E-01
gene11360	18127	NC_000071.6	Nos3	5.79E-01
gene41336	106869	NC_000084.6	Tnfaip8	5.79E-01
gene30479	170721	NC_000078.6	Papln	5.78E-01
gene11697	100515	NC_000071.6	Zfp518b	5.78E-01
gene16335	269854	NC_000073.6	Nat14	5.78E-01
gene13708	1E+08	NC_000072.6	Gm35446	5.78E-01
gene30692	72159	NC_000078.6	Gm9726	5.78E-01
gene27927	71522	NC_000077.6	Ggt6	5.78E-01
gene2312	381308	NC_000067.6	Mnda	5.78E-01
gene28658	22780	NC_000077.6	Ikzf3	5.78E-01
gene10209	14622	NC_000070.6	Gjb5	5.77E-01

gene15295	243574	NC_000072.6	Kbtbd8	5.77E-01
gene19570	71131	NC_000073.6	Zfp689	5.77E-01
gene22647	69305	NC_000075.6	Dcps	5.77E-01
gene3416	26920	NC_000068.7	Cntrl	5.76E-01
gene30035	14762	NC_000078.6	Gpr33	5.76E-01
gene28483	264895	NC_000077.6	Acsf2	5.76E-01
gene23582	208943	NC_000075.6	Myo5c	5.76E-01
gene37860	224111	NC_000082.6	Ubxn7	5.76E-01
gene8351	54120	NC_000069.6	Gipc2	5.76E-01
gene31502	207615	NC_000079.6	Wdr37	5.75E-01
gene19259	11717	NC_000073.6	Ampd3	5.75E-01
gene19387	71151	NC_000073.6	Eri2	5.75E-01
gene29206	217331	NC_000077.6	Unk	5.74E-01
gene31407	217944	NC_000078.6	Rapgef5	5.74E-01
gene41790	18018	NC_000084.6	Nfatc1	5.73E-01
gene37498	72083	NC_000082.6	Mzt2	5.73E-01
gene39279	56520	NC_000083.6	Nme4	5.72E-01
gene34014	75901	NC_000080.6	Dcp1a	5.72E-01
gene30021	217558	NC_000078.6	G2e3	5.72E-01
gene15986	16974	NC_000072.6	Lrp6	5.72E-01
gene39908	12488	NC_000083.6	Cd2ap	5.71E-01
gene24920	103213	NC_000076.6	Traf3ip2	5.71E-01
gene19934	101513	NC_000073.6	Mob2	5.71E-01
gene24158	235610	NC_000075.6	Atrip	5.71E-01
gene7697	94093	NC_000069.6	Trim33	5.71E-01
gene35298	66148	NC_000080.6	Dnajc15	5.71E-01
gene19991	27267	NC_000073.6	Cars	5.71E-01
gene15240	232232	NC_000072.6	Hdac11	5.71E-01
gene36937	67057	NC_000081.6	Yaf2	5.71E-01
gene35673	1.1E+08	NC_000081.6	Gm41254	5.71E-01
gene42552	22359	NC_000085.6	Vldlr	5.70E-01
gene4841	108907	NC_000068.7	Nusap1	5.70E-01
gene2469	ene;gene=l	NC_000067.6	LOC105244034	5.70E-01
gene30484	1E+08	NC_000078.6	Heatr4	5.70E-01
gene11005	320541	NC_000070.6	Slc35e2	5.70E-01
gene27675	52712	NC_000077.6	Zkscan6	5.70E-01
gene39269	224624	NC_000083.6	Rab40c	5.69E-01
gene17589	1E+08	NC_000073.6	Gm35484	5.69E-01
gene19702	1E+08	NC_000073.6	Gm10584	5.69E-01
gene34020	328370	NC_000080.6	Rft1	5.68E-01
gene20939	70885	NC_000074.6	Ints10	5.68E-01
gene16293	58804	NC_000073.6	Cdc42ep5	5.68E-01
gene28555	75395	NC_000077.6	Hoxb5os	5.68E-01
gene36286	21819	NC_000081.6	Tg	5.68E-01
gene27673	26398	NC_000077.6	Map2k4	5.68E-01
gene6846	622434	NC_000069.6	Arhgef26	5.68E-01
gene15107	21841	NC_000072.6	Tia1	5.67E-01
gene36644	105835	NC_000081.6	Sgsm3	5.67E-01

gene9000	22590	NC_000070.6	Xpa	5.67E-01
gene27518	69320	NC_000077.6	1700007J10Rik	5.67E-01
gene14547	71835	NC_000072.6	Lancl2	5.67E-01
gene39419	21769	NC_000083.6	Zfand3	5.67E-01
gene10345	100169	NC_000070.6	Phactr4	5.67E-01
gene29115	276852	NC_000077.6	D11Wsu47e	5.67E-01
gene19718	360216	NC_000073.6	Zranb1	5.66E-01
gene1477	68428	NC_000067.6	Steap3	5.66E-01
gene4491	69113	NC_000068.7	Alkbh3	5.66E-01
gene12578	ene;gene=L	NC_000071.6	LOC105246961	5.66E-01
gene14864	13666	NC_000072.6	Eif2ak3	5.65E-01
gene24340	69274	NC_000075.6	Ctdspl	5.65E-01
gene3899	53375	NC_000068.7	Mtx2	5.65E-01
gene14069	381760	NC_000072.6	Ssbp1	5.64E-01
gene15595	214932	NC_000072.6	Cecr5	5.64E-01
gene39111	320020	NC_000083.6	6330415G19Rik	5.64E-01
gene30494	105000	NC_000078.6	Dnal1	5.64E-01
gene11467	100763	NC_000071.6	Ube3c	5.64E-01
gene19498	18020	NC_000073.6	Nfatc2ip	5.63E-01
gene11884	68303	NC_000071.6	Fam114a1	5.63E-01
gene41086	225372	NC_000084.6	Apbb3	5.63E-01
gene28331	53892	NC_000077.6	Ppm1d	5.63E-01
gene16009	74525	NC_000072.6	Fam234b	5.63E-01
gene34755	50934	NC_000080.6	Slc7a8	5.63E-01
gene9891	16351	NC_000070.6	Ipp	5.63E-01
gene3656	14571	NC_000068.7	Gpd2	5.63E-01
gene28247	217011	NC_000077.6	Nle1	5.63E-01
gene25004	67851	NC_000076.6	1700021F05Rik	5.63E-01
gene23141	66402	NC_000075.6	Sln	5.63E-01
gene22706	20686	NC_000075.6	Spa17	5.62E-01
gene40686	225115	NC_000084.6	Svil	5.62E-01
gene24142	23807	NC_000075.6	Arih2	5.62E-01
gene8199	229877	NC_000069.6	Rap1gds1	5.62E-01
gene6984	56426	NC_000069.6	Pdcd10	5.62E-01
gene16919	80794	NC_000073.6	Cblc	5.62E-01
gene12525	433931	NC_000071.6	Pigg	5.61E-01
gene18840	=Gene;ger	NC_000073.6	LOC102639173	5.61E-01
gene22145	69627	NC_000074.6	Fam89a	5.61E-01
gene18721	12337	NC_000073.6	Capn5	5.61E-01
gene40828	11829	NC_000084.6	Aqp4	5.61E-01
gene25334	76551	NC_000076.6	Ccdc6	5.61E-01
gene29407	66179	NC_000077.6	Ogfod3	5.61E-01
gene9046	66665	NC_000070.6	Msantd3	5.60E-01
gene32265	94245	NC_000079.6	Dtnbp1	5.60E-01
gene7597	18129	NC_000069.6	Notch2	5.60E-01
gene33868	12325	NC_000080.6	Camk2g	5.60E-01
gene16190	232536	NC_000072.6	Mrps35	5.59E-01
gene40438	50766	NC_000083.6	Crim1	5.59E-01

gene17617	668501	NC_000073.6	Zfp507	5.59E-01
gene8301	70951	NC_000069.6	Spata1	5.59E-01
gene2081	226591	NC_000067.6	Tiprl	5.59E-01
gene6178	229004	NC_000068.7	Gmeb2	5.58E-01
gene16308	53420	NC_000073.6	Syt5	5.58E-01
gene25018	1.1E+08	NC_000076.6	Gm40636	5.57E-01
gene37480	17880	NC_000082.6	Myh11	5.57E-01
gene16628	67370	NC_000073.6	Zfp606	5.57E-01
gene18757	17760	NC_000073.6	Map6	5.57E-01
gene38250	e;gbkey=G	NC_000082.6	LOC102636734	5.57E-01
gene26402	210035	NC_000076.6	Tmem194	5.57E-01
gene4562	1.1E+08	NC_000068.7	Gm39894	5.57E-01
gene21415	257632	NC_000074.6	Nod2	5.56E-01
gene139	66799	NC_000067.6	Ube2w	5.56E-01
gene11760	54392	NC_000071.6	Ncapg	5.56E-01
gene26419	1.1E+08	NC_000076.6	Gm40806	5.56E-01
gene3220	64930	NC_000068.7	Tsc1	5.56E-01
gene39443	57773	NC_000083.6	Wdr4	5.55E-01
gene30418	320808	NC_000078.6	Dcaf5	5.55E-01
gene41809	ene;gene=l	NC_000084.6	LOC108168376	5.55E-01
gene27145	1E+08	NC_000077.6	Gm12167	5.55E-01
gene37689	1E+08	NC_000082.6	1300002E11Rik	5.55E-01
gene2016	75368	NC_000067.6	4930558K02Rik	5.55E-01
gene12989	215707	NC_000071.6	Ccdc92	5.54E-01
gene33285	268697	NC_000079.6	Ccnb1	5.54E-01
gene42728	74107	NC_000085.6	Cep55	5.54E-01
gene23917	74769	NC_000075.6	Pik3cb	5.54E-01
gene8562	72397	NC_000070.6	Rbm12b1	5.54E-01
gene38325	67742	NC_000082.6	Samsn1	5.54E-01
gene27837	17312	NC_000077.6	Clec10a	5.54E-01
gene24530	108853	NC_000076.6	Mtrf1l	5.54E-01
gene1188	12835	NC_000067.6	Col6a3	5.53E-01
gene1087	26895	NC_000067.6	Cops7b	5.53E-01
gene21054	76478	NC_000074.6	Haus8	5.53E-01
gene36150	319944	NC_000081.6	Taf2	5.53E-01
gene28018	97761	NC_000077.6	Sgsm2	5.53E-01
gene8398	12972	NC_000069.6	Cryz	5.52E-01
gene17383	22689	NC_000073.6	Zfp27	5.52E-01
gene649	213056	NC_000067.6	Fam126b	5.52E-01
gene5350	77705	NC_000068.7	9230104L09Rik	5.52E-01
gene17976	70209	NC_000073.6	Tmem143	5.52E-01
gene9108	100434	NC_000070.6	Slc44a1	5.51E-01
gene25843	16000	NC_000076.6	Igf1	5.51E-01
gene27189	667214	NC_000077.6	9930111J21Rik1	5.51E-01
gene10881	74646	NC_000070.6	Spsb1	5.51E-01
gene32970	ene;gene=l	NC_000079.6	LOC108168101	5.51E-01
gene3698	16420	NC_000068.7	Itgb6	5.51E-01
gene24772	70208	NC_000076.6	Med23	5.50E-01

gene40654	76781	NC_000083.6	Mettl4	5.50E-01
gene20756	1E+08	NC_000074.6	Gm16675	5.50E-01
gene21817	234733	NC_000074.6	Ddx19b	5.50E-01
gene836	381269	NC_000067.6	Mreg	5.49E-01
gene27344	19360	NC_000077.6	Rad50	5.48E-01
gene11890	19687	NC_000071.6	Rfc1	5.48E-01
gene3862	1E+08	NC_000068.7	Gm13709	5.48E-01
gene39777	1E+08	NC_000083.6	Gm33595	5.48E-01
gene10296	80912	NC_000070.6	Pum1	5.48E-01
gene9944	1.1E+08	NC_000070.6	Gm40233	5.48E-01
gene28561	=Gene;ger	NC_000077.6	LOC102632302	5.47E-01
gene26837	58172	NC_000077.6	Sertad2	5.47E-01
gene23081	72614	NC_000075.6	Pih1d2	5.47E-01
gene36624	17309	NC_000081.6	Mgat3	5.47E-01
gene38244	224273	NC_000082.6	Crybg3	5.47E-01
gene4877	96957	NC_000068.7	Tmem62	5.47E-01
gene32862	210009	NC_000079.6	Mtrr	5.46E-01
gene41710	1E+08	NC_000084.6	Gm20544	5.46E-01
gene7420	22156	NC_000069.6	Tuft1	5.46E-01
gene3674	227937	NC_000068.7	Pkp4	5.45E-01
gene12694	231642	NC_000071.6	Alkbh2	5.44E-01
gene38663	106583	NC_000083.6	Scaf8	5.44E-01
gene39707	73242	NC_000083.6	Atat1	5.44E-01
gene41242	56070	NC_000084.6	Tcerg1	5.43E-01
gene21328	1E+08	NC_000074.6	Gm38426	5.43E-01
gene3269	227699	NC_000068.7	Nup188	5.43E-01
gene35207	319448	NC_000080.6	Fndc3a	5.43E-01
gene38553	246133	NC_000082.6	Kcne2	5.43E-01
gene13069	71970	NC_000071.6	Zbed5	5.43E-01
gene27361	71056	NC_000077.6	4933405E24Rik	5.43E-01
gene41460	106878	NC_000084.6	Smim3	5.43E-01
gene37048	69612	NC_000081.6	Kansl2	5.42E-01
gene12317	71914	NC_000071.6	Antxr2	5.42E-01
gene26889	17847	NC_000077.6	Usp34	5.42E-01
gene4449	12953	NC_000068.7	Cry2	5.41E-01
gene5039	17289	NC_000068.7	Mertk	5.41E-01
gene16652	269870	NC_000073.6	Zfp446	5.41E-01
gene42978	108689	NC_000085.6	Obfc1	5.41E-01
gene38599	22129	NC_000082.6	Ttc3	5.41E-01
gene12596	19301	NC_000071.6	Pxmp2	5.41E-01
gene40156	27221	NC_000083.6	Chaf1a	5.40E-01
gene26257	117599	NC_000076.6	Helb	5.40E-01
gene19727	214764	NC_000073.6	Edrf1	5.40E-01
gene1498	66942	NC_000067.6	Ddx18	5.40E-01
gene38935	240028	NC_000083.6	Lnpep	5.40E-01
gene4805	51944	NC_000068.7	Knstrn	5.40E-01
gene26899	216578	NC_000077.6	Papolg	5.40E-01
gene37892	22661	NC_000082.6	Zfp148	5.40E-01

gene30716	24000	NC_000078.6	Ptpn21	5.40E-01
gene13076	22021	NC_000071.6	Tpst1	5.39E-01
gene8997	21916	NC_000070.6	Tmod1	5.39E-01
gene6701	56458	NC_000069.6	Foxo1	5.39E-01
gene2075	16963	NC_000067.6	Xcl1	5.39E-01
gene35299	108670	NC_000080.6	Epsti1	5.39E-01
gene7580	66659	NC_000069.6	Acp6	5.38E-01
gene28516	215512	NC_000077.6	Fam117a	5.38E-01
gene2984	18718	NC_000068.7	Pip4k2a	5.38E-01
gene8478	68053	NC_000070.6	Ubxn2b	5.38E-01
gene39684	240084	NC_000083.6	Cchcr1	5.38E-01
gene4720	72425	NC_000068.7	Katnbl1	5.38E-01
gene24871	72580	NC_000076.6	Zufsp	5.37E-01
gene17291	338354	NC_000073.6	Zfp780b	5.37E-01
gene10478	230837	NC_000070.6	Asap3	5.37E-01
gene23859	24127	NC_000075.6	Xrn1	5.37E-01
gene37268	18022	NC_000081.6	Nfe2	5.37E-01
gene1659	53311	NC_000067.6	Mybph	5.37E-01
gene7248	=Gene;ger	NC_000069.6	LOC108168879	5.37E-01
gene42119	68229	NC_000085.6	AI846148	5.36E-01
gene15610	74043	NC_000072.6	Pex26	5.36E-01
gene33847	67725	NC_000080.6	Nudt13	5.36E-01
gene19817	381933	NC_000073.6	6430531B16Rik	5.36E-01
gene31691	544922	NC_000079.6	Zkscan4	5.36E-01
gene11563	22612	NC_000071.6	Yes1	5.36E-01
gene20227	66822	NC_000074.6	Fbxo25	5.36E-01
gene17689	1E+08	NC_000073.6	Gm33989	5.36E-01
gene2247	30925	NC_000067.6	Slamf6	5.36E-01
gene31503	319554	NC_000079.6	Idi1	5.36E-01
gene5555	=Gene;ger	NC_000068.7	LOC102636309	5.35E-01
gene7020	68659	NC_000069.6	Fam198b	5.35E-01
gene9754	52430	NC_000070.6	Echdc2	5.35E-01
gene20210	21781	NC_000074.6	Tfdp1	5.35E-01
gene13393	231871	NC_000071.6	Daglb	5.35E-01
gene10557	77056	NC_000070.6	Tmco4	5.35E-01
gene42721	1E+08	NC_000085.6	Gm32342	5.35E-01
gene4971	69185	NC_000068.7	Dtwd1	5.35E-01
gene23352	56469	NC_000075.6	Pias1	5.35E-01
gene18752	67800	NC_000073.6	Dgat2	5.34E-01
gene24842	66847	NC_000076.6	Hint3	5.34E-01
gene11112	79264	NC_000071.6	Krit1	5.34E-01
gene39165	19826	NC_000083.6	Rnps1	5.34E-01
gene30229	74724	NC_000078.6	4930512B01Rik	5.34E-01
gene32829	408065	NC_000079.6	Zfp456	5.34E-01
gene4421	75786	NC_000068.7	Ckap5	5.34E-01
gene24193	1.1E+08	NC_000075.6	Gm39429	5.33E-01
gene32833	238692	NC_000079.6	Zfp874a	5.33E-01
gene23393	102442	NC_000075.6	Dennd4a	5.33E-01

gene43013	17859	NC_000085.6	Mxi1	5.33E-01
gene17403	381867	NC_000073.6	Ovol3	5.33E-01
gene36147	18606	NC_000081.6	Enpp2	5.33E-01
gene298	19335	NC_000067.6	Rab23	5.33E-01
gene8973	64424	NC_000070.6	Polr1e	5.32E-01
gene10193	100317	NC_000070.6	AU040320	5.32E-01
gene38257	68146	NC_000082.6	Arl13b	5.32E-01
gene10139	52276	NC_000070.6	Cdca8	5.32E-01
gene13942	27418	NC_000072.6	Mkln1	5.32E-01
gene43064	55947	NC_000085.6	Dclre1a	5.32E-01
gene25117	73390	NC_000076.6	Msl3l2	5.31E-01
gene41702	319195	NC_000084.6	Rpl17	5.31E-01
gene2166	226641	NC_000067.6	Atf6	5.31E-01
gene22429	22688	NC_000075.6	Zfp26	5.31E-01
gene19586	12055	NC_000073.6	Bcl7c	5.31E-01
gene33981	71704	NC_000080.6	Arhgef3	5.31E-01
gene35244	1E+08	NC_000080.6	Gm15628	5.31E-01
gene25729	1E+08	NC_000076.6	Gm33378	5.31E-01
gene23968	28135	NC_000075.6	Cep63	5.31E-01
gene24129	78267	NC_000075.6	Klhdc8b	5.30E-01
gene23103	1E+08	NC_000075.6	Gm684	5.30E-01
gene17335	71984	NC_000073.6	Sars2	5.30E-01
gene26612	53897	NC_000077.6	Gal3st1	5.30E-01
gene16896	12715	NC_000073.6	Ckm	5.30E-01
gene41747	225745	NC_000084.6	Haus1	5.30E-01
gene21097	382019	NC_000074.6	Zfp882	5.30E-01
gene24519	73419	NC_000076.6	Armt1	5.30E-01
gene30033	320487	NC_000078.6	Heatr5a	5.30E-01
gene2378	78825	NC_000067.6	Desi2	5.30E-01
gene21910	234779	NC_000074.6	Plcg2	5.29E-01
gene31976	73326	NC_000079.6	4932702P03Rik	5.29E-01
gene4876	22222	NC_000068.7	Ubr1	5.29E-01
gene742	75619	NC_000067.6	Fastkd2	5.29E-01
gene7751	1E+08	NC_000069.6	Pifo	5.29E-01
gene8424	67144	NC_000069.6	Lrrc40	5.29E-01
gene20654	80286	NC_000074.6	Tusc3	5.29E-01
gene38495	224432	NC_000082.6	Scaf4	5.29E-01
gene6840	18441	NC_000069.6	P2ry1	5.29E-01
gene32863	69577	NC_000079.6	Fastkd3	5.29E-01
gene42690	54364	NC_000085.6	Rpp30	5.28E-01
gene7997	69608	NC_000069.6	Sec24d	5.28E-01
gene13305	319772	NC_000071.6	C130050O18Rik	5.28E-01
gene15781	19362	NC_000072.6	Rad51ap1	5.28E-01
gene30278	208846	NC_000078.6	Daam1	5.28E-01
gene24281	78893	NC_000075.6	Cnot10	5.28E-01
gene30265	30056	NC_000078.6	Timm9	5.27E-01
gene28680	68524	NC_000077.6	Wipf2	5.27E-01
gene2301	545384	NC_000067.6	BC094916	5.27E-01

gene33933	328365	NC_000080.6	Zmiz1	5.27E-01
gene29805	217449	NC_000078.6	Trappc12	5.27E-01
gene38646	207781	NC_000082.6	C2cd2	5.27E-01
gene25826	66263	NC_000076.6	1810014B01Rik	5.27E-01
gene25738	ene=LOC1	NC_000076.6	LOC105245240	5.27E-01
gene18530	16170	NC_000073.6	Il16	5.26E-01
gene27103	69183	NC_000077.6	C1qtnf2	5.26E-01
gene3818	78830	NC_000068.7	Slc25a12	5.26E-01
gene27936	192986	NC_000077.6	Cyb5d2	5.26E-01
gene20104	669393	NC_000074.6	Gm9457	5.26E-01
gene29224	217340	NC_000077.6	Rnf157	5.26E-01
gene31292	1E+08	NC_000078.6	Gm34465	5.26E-01
gene39513	1E+08	NC_000083.6	Zfp955b	5.26E-01
gene22469	94226	NC_000075.6	S1pr5	5.25E-01
gene15015	75659	NC_000072.6	Wdr54	5.25E-01
gene29275	71562	NC_000077.6	Afmid	5.25E-01
gene36557	107753	NC_000081.6	Lgals2	5.25E-01
gene39408	381085	NC_000083.6	Tbc1d22b	5.25E-01
gene32188	108652	NC_000079.6	Slc35b3	5.25E-01
gene39502	73451	NC_000083.6	Zfp763	5.25E-01
gene28699	16661	NC_000077.6	Krt10	5.25E-01
gene2524	1E+08	NC_000067.6	Gm34882	5.25E-01
gene37151	207839	NC_000081.6	Galnt6	5.25E-01
gene34913	219134	NC_000080.6	Shisa2	5.25E-01
gene32428	1E+08	NC_000079.6	Gm33315	5.24E-01
gene26454	654440	NC_000076.6	A430046D13Rik	5.24E-01
gene18124	233276	NC_000073.6	Tubgcp5	5.23E-01
gene26171	72068	NC_000076.6	Cnot2	5.23E-01
gene7780	229700	NC_000069.6	Rbm15	5.23E-01
gene33857	75602	NC_000080.6	1810062O18Rik	5.23E-01
gene22479	60507	NC_000075.6	Qtrt1	5.22E-01
gene29333	108100	NC_000077.6	Baiap2	5.22E-01
gene17920	21677	NC_000073.6	Tead2	5.22E-01
gene28603	58996	NC_000077.6	Arhgap23	5.22E-01
gene37861	277203	NC_000082.6	Tm4sf19	5.22E-01
gene1992	381305	NC_000067.6	Rc3h1	5.22E-01
gene42904	107250	NC_000085.6	Kazald1	5.22E-01
gene29295	217364	NC_000077.6	Engase	5.21E-01
gene23645	235504	NC_000075.6	Slc17a5	5.21E-01
gene8823	11828	NC_000070.6	Aqp3	5.21E-01
gene2316	226695	NC_000067.6	Ifi205	5.21E-01
gene35185	65246	NC_000080.6	Xpo7	5.20E-01
gene23687	83946	NC_000075.6	Phip	5.20E-01
gene24918	1E+08	NC_000076.6	Gm16365	5.20E-01
gene36735	239559	NC_000081.6	A4galt	5.20E-01
gene7709	19260	NC_000069.6	Ptpn22	5.19E-01
gene11376	66993	NC_000071.6	Smarcd3	5.19E-01
gene24461	102436	NC_000075.6	Lars2	5.19E-01

gene34762	59049	NC_000080.6	Slc22a17	5.19E-01
gene3684	72137	NC_000068.7	Wdsub1	5.19E-01
gene12730	74585	NC_000071.6	Sppl3	5.19E-01
gene22612	235134	NC_000075.6	Nfrkb	5.18E-01
gene23223	56807	NC_000075.6	Scamp5	5.18E-01
gene14434	1.1E+08	NC_000072.6	Hotairm1	5.18E-01
gene8076	66815	NC_000069.6	Ccdc109b	5.18E-01
gene15731	381810	NC_000072.6	Lpar5	5.18E-01
gene22336	75316	NC_000075.6	Taf1d	5.18E-01
gene35737	71175	NC_000081.6	Nipbl	5.18E-01
gene27419	76516	NC_000077.6	2010001A14Rik	5.18E-01
gene25905	17997	NC_000076.6	Nedd1	5.18E-01
gene32594	78689	NC_000079.6	Naa35	5.18E-01
gene28466	1.1E+08	NC_000077.6	Gm38951	5.17E-01
gene40364	74355	NC_000083.6	Smchd1	5.17E-01
gene32820	380855	NC_000079.6	Rsl1	5.17E-01
gene21907	1.1E+08	NC_000074.6	Gm39260	5.17E-01
gene12775	1E+08	NC_000071.6	Gm36841	5.17E-01
gene42779	67590	NC_000085.6	Tctn3	5.17E-01
gene24548	69912	NC_000076.6	Nup43	5.16E-01
gene1591	212980	NC_000067.6	Slc45a3	5.16E-01
gene6537	72039	NC_000069.6	Mccc1	5.16E-01
gene26188	327826	NC_000076.6	Frs2	5.16E-01
gene15578	22419	NC_000072.6	Wnt5b	5.16E-01
gene21552	78833	NC_000074.6	Gins3	5.16E-01
gene11349	84652	NC_000071.6	Fam126a	5.16E-01
gene41642	1E+08	NC_000084.6	Gm31439	5.16E-01
gene10518	11647	NC_000070.6	Alpl	5.16E-01
gene4951	317750	NC_000068.7	Slc24a5	5.16E-01
gene24062	321006	NC_000075.6	Vprbp	5.15E-01
gene23669	17920	NC_000075.6	Myo6	5.15E-01
gene28641	69131	NC_000077.6	Cdk12	5.15E-01
gene1677	329251	NC_000067.6	Ppp1r12b	5.15E-01
gene2579	226830	NC_000067.6	Smyd2	5.14E-01
gene7756	81600	NC_000069.6	Chia1	5.14E-01
gene34909	69008	NC_000080.6	Cab39l	5.14E-01
gene3934	23992	NC_000068.7	Prkra	5.14E-01
gene36334	106068	NC_000081.6	Slc45a4	5.14E-01
gene15783	28040	NC_000072.6	D6Wsu163e	5.14E-01
gene3354	14287	NC_000068.7	Fpgs	5.14E-01
gene23824	78575	NC_000075.6	B430319G15Rik	5.14E-01
gene34758	239099	NC_000080.6	Homez	5.14E-01
gene38079	213012	NC_000082.6	Abhd10	5.13E-01
gene17413	233067	NC_000073.6	Lrfn3	5.13E-01
gene18123	233274	NC_000073.6	Siglech	5.13E-01
gene11104	68152	NC_000071.6	Fam133b	5.13E-01
gene17995	78935	NC_000073.6	Saal1	5.12E-01
gene2915	66960	NC_000068.7	Fam188a	5.12E-01

gene20572	19663	NC_000074.6	Rbpms	5.12E-01
gene18777	50877	NC_000073.6	Neu3	5.12E-01
gene32920	328287	NC_000079.6	Gm20554	5.12E-01
gene27705	93896	NC_000077.6	Glp2r	5.12E-01
gene27926	276829	NC_000077.6	Smtnl2	5.12E-01
gene27340	16568	NC_000077.6	Kif3a	5.12E-01
gene7243	74034	NC_000069.6	4632404H12Rik	5.12E-01
gene1985	329278	NC_000067.6	Tnn	5.12E-01
gene38733	380608	NC_000083.6	Tagap1	5.11E-01
gene3115	=Gene;ger	NC_000068.7	LOC102639143	5.11E-01
gene67	76187	NC_000067.6	Adhfe1	5.11E-01
gene29318	170720	NC_000077.6	Card14	5.11E-01
gene40663	12916	NC_000084.6	Crem	5.11E-01
gene27570	625958	NC_000077.6	Gm12611	5.11E-01
gene23752	244962	NC_000075.6	Snx14	5.10E-01
gene3377	26360	NC_000068.7	Angptl2	5.10E-01
gene12996	20602	NC_000071.6	Ncor2	5.10E-01
gene25549	72128	NC_000076.6	2610008E11Rik	5.10E-01
gene8755	30046	NC_000070.6	Zfp292	5.10E-01
gene36084	67429	NC_000081.6	Nudcd1	5.10E-01
gene8540	207920	NC_000070.6	Esrp1	5.09E-01
gene3007	71435	NC_000068.7	Arhgap21	5.09E-01
gene19372	67133	NC_000073.6	Gp2	5.09E-01
gene19648	1E+08	NC_000073.6	Gm33248	5.09E-01
gene40220	1E+08	NC_000083.6	Gm11110	5.09E-01
gene22222	384911	NC_000075.6	Gm5362	5.09E-01
gene27782	11932	NC_000077.6	Atp1b2	5.08E-01
gene29913	217473	NC_000078.6	Ankmy2	5.08E-01
gene1999	226541	NC_000067.6	Klhl20	5.08E-01
gene18067	243983	NC_000073.6	Zdhhc13	5.08E-01
gene21747	54446	NC_000074.6	Nfat5	5.08E-01
gene20938	73609	NC_000074.6	1700125H03Rik	5.08E-01
gene27852	11687	NC_000077.6	Alox15	5.08E-01
gene32844	408068	NC_000079.6	Zfp738	5.07E-01
gene31032	=Gene;ger	NC_000078.6	LOC102638940	5.07E-01
gene30260	19167	NC_000078.6	Psma3	5.07E-01
gene2123	80914	NC_000067.6	Uck2	5.07E-01
gene39040	22712	NC_000083.6	Zfp54	5.07E-01
gene28424	56077	NC_000077.6	Dgke	5.07E-01
gene17390	243905	NC_000073.6	Zfp568	5.07E-01
gene30095	52592	NC_000078.6	Brms1l	5.06E-01
gene15845	93675	NC_000072.6	Clec2i	5.06E-01
gene22600	30806	NC_000075.6	Adamts8	5.06E-01
gene25632	1E+08	NC_000076.6	Gm26710	5.05E-01
gene21911	74032	NC_000074.6	Sdr42e1	5.05E-01
gene13066	67902	NC_000071.6	Sumf2	5.05E-01
gene15988	67729	NC_000072.6	Mansc1	5.04E-01
gene10240	74648	NC_000070.6	S100pbp	5.04E-01

gene1675	75605	NC_000067.6	Kdm5b	5.04E-01
gene1109	1E+08	NC_000067.6	Kcnj13	5.04E-01
gene25706	114606	NC_000076.6	Tle6	5.04E-01
gene42529	76088	NC_000085.6	Dock8	5.04E-01
gene30329	80837	NC_000078.6	Rhoj	5.04E-01
gene37574	19125	NC_000082.6	Prodh	5.04E-01
gene31610	218035	NC_000079.6	Vps41	5.04E-01
gene11501	100732	NC_000071.6	Mapre3	5.04E-01
gene13382	54201	NC_000071.6	Zfp316	5.04E-01
gene32695	1E+08	NC_000079.6	Gm16907	5.03E-01
gene25926	57764	NC_000076.6	Ntn4	5.03E-01
gene5868	1E+08	NC_000068.7	Gm33108	5.03E-01
gene11615	15194	NC_000071.6	Htt	5.03E-01
gene300	98403	NC_000067.6	Zfp451	5.03E-01
gene12862	114643	NC_000071.6	Oas1c	5.03E-01
gene21118	13859	NC_000074.6	Eps15l1	5.03E-01
gene1815	18783	NC_000067.6	Pla2g4a	5.03E-01
gene1211	74019	NC_000067.6	Traf3ip1	5.02E-01
gene32966	622175	NC_000079.6	E430024I08Rik	5.02E-01
gene35218	19645	NC_000080.6	Rb1	5.02E-01
gene36916	26874	NC_000081.6	Abcd2	5.02E-01
gene30674	83602	NC_000078.6	Gtf2a1	5.02E-01
gene20570	68153	NC_000074.6	Gtf2e2	5.01E-01
gene29440	75302	NC_000078.6	Asxl2	5.01E-01
gene38157	385658	NC_000082.6	Nxpe3	5.01E-01
gene32285	=Gene;ger	NC_000079.6	LOC108168080	5.01E-01
gene36623	66513	NC_000081.6	Tab1	5.01E-01
gene19463	70497	NC_000073.6	Arhgap17	5.00E-01
gene10346	67219	NC_000070.6	Med18	5.00E-01
gene3808	1E+08	NC_000068.7	Gm38528	5.00E-01
gene39356	19015	NC_000083.6	Ppard	5.00E-01
gene7498	194126	NC_000069.6	Mtmr11	5.00E-01
gene39785	22715	NC_000083.6	Zfp57	4.99E-01
gene18815	320100	NC_000073.6	Relt	4.99E-01
gene37934	433016	NC_000082.6	Gm5483	4.99E-01
gene41020	75533	NC_000084.6	Nme5	4.99E-01
gene1746	69549	NC_000067.6	2310009B15Rik	4.99E-01
gene28898	14615	NC_000077.6	Gjc1	4.99E-01
gene8923	230099	NC_000070.6	Car9	4.99E-01
gene37173	=Gene;ger	NC_000081.6	LOC102639385	4.99E-01
gene23625	56542	NC_000075.6	Ick	4.99E-01
gene28889	77132	NC_000077.6	2810433D01Rik	4.98E-01
gene16192	232539	NC_000072.6	Klhl42	4.98E-01
gene30739	217827	NC_000078.6	Nrde2	4.98E-01
gene21683	1E+08	NC_000074.6	Gm33578	4.98E-01
gene38864	240025	NC_000083.6	Dact2	4.97E-01
gene17958	243967	NC_000073.6	Ntn5	4.97E-01
gene19389	233805	NC_000073.6	Dcun1d3	4.97E-01

gene33803	353187	NC_000080.6	Nr1d2	4.97E-01
gene30423	97827	NC_000078.6	Exd2	4.96E-01
gene3321	227720	NC_000068.7	Nup214	4.96E-01
gene30261	73204	NC_000078.6	3110056K07Rik	4.96E-01
gene24426	215494	NC_000075.6	Pomgnt2	4.96E-01
gene38266	72020	NC_000082.6	Zfp654	4.96E-01
gene41877	ene;gene=L	NC_000084.6	LOC108168395	4.96E-01
gene1928	74081	NC_000067.6	Cep350	4.96E-01
gene29053	12006	NC_000077.6	Axin2	4.96E-01
gene39445	18771	NC_000083.6	Pknox1	4.96E-01
gene897	67534	NC_000067.6	Ttll4	4.96E-01
gene33992	22418	NC_000080.6	Wnt5a	4.95E-01
gene5016	228576	NC_000068.7	Mall	4.95E-01
gene27779	216853	NC_000077.6	Wrap53	4.95E-01
gene26286	74694	NC_000076.6	Tbc1d30	4.95E-01
gene2441	67459	NC_000067.6	Nvl	4.95E-01
gene37979	56637	NC_000082.6	Gsk3b	4.95E-01
gene40447	268980	NC_000083.6	Strn	4.94E-01
gene1596	18557	NC_000067.6	Cdk18	4.94E-01
gene32184	17828	NC_000079.6	Bloc1s5	4.94E-01
gene32489	26919	NC_000079.6	Zfp346	4.94E-01
gene20228	72148	NC_000074.6	Tdrp	4.94E-01
gene9039	18124	NC_000070.6	Nr4a3	4.93E-01
gene14916	232089	NC_000072.6	Elmod3	4.93E-01
gene5552	60525	NC_000068.7	Acss2	4.93E-01
gene16805	434128	NC_000073.6	Pnmal2	4.93E-01
gene28604	1E+08	NC_000077.6	4933428G20Rik	4.93E-01
gene26037	68281	NC_000076.6	4930430F08Rik	4.93E-01
gene2600	381319	NC_000067.6	Batf3	4.93E-01
gene21690	74334	NC_000074.6	Ranbp10	4.92E-01
gene30711	14420	NC_000078.6	Galc	4.92E-01
gene21101	72054	NC_000074.6	Cyp4f18	4.92E-01
gene1641	381290	NC_000067.6	Atp2b4	4.92E-01
gene29030	1E+08	NC_000077.6	Gm11716	4.92E-01
gene34873	1.1E+08	NC_000080.6	Gm41161	4.92E-01
gene30930	214663	NC_000078.6	Slc25a29	4.92E-01
gene15104	68011	NC_000072.6	Snrpg	4.92E-01
gene14433	15394	NC_000072.6	Hoxa1	4.92E-01
gene4501	66457	NC_000068.7	2810002D19Rik	4.91E-01
gene27433	216767	NC_000077.6	Mrpl22	4.91E-01
gene18655	78329	NC_000073.6	2310010J17Rik	4.91E-01
gene42501	226026	NC_000085.6	Smc5	4.89E-01
gene29197	170472	NC_000077.6	Recql5	4.89E-01
gene39988	78309	NC_000083.6	Cul9	4.89E-01
gene3300	74270	NC_000068.7	Usp20	4.89E-01
gene8365	329777	NC_000069.6	Pigk	4.89E-01
gene24289	74486	NC_000075.6	Osbpl10	4.88E-01
gene15453	101122	NC_000072.6	Rpusd3	4.88E-01

gene14153	13848	NC_000072.6	Ephb6	4.88E-01
gene29156	320534	NC_000077.6	Tmem104	4.88E-01
gene20945	110877	NC_000074.6	Slc18a1	4.88E-01
gene24160	66658	NC_000075.6	Ccdc51	4.88E-01
gene33015	1E+08	NC_000079.6	Gm38604	4.88E-01
gene10459	242700	NC_000070.6	Ifnlr1	4.88E-01
gene28901	73293	NC_000077.6	Ccdc103	4.87E-01
gene5954	320664	NC_000068.7	Cass4	4.87E-01
gene23756	71640	NC_000075.6	Zfp949	4.87E-01
gene39437	328795	NC_000083.6	Ubash3a	4.86E-01
gene39376	268936	NC_000083.6	Brpf3	4.86E-01
gene2914	338535	NC_000068.7	E030013I19Rik	4.86E-01
gene25190	107449	NC_000076.6	Unc5b	4.86E-01
gene654	1E+08	NC_000067.6	Gm20257	4.86E-01
gene2957	74103	NC_000068.7	Nebl	4.86E-01
gene37095	54614	NC_000081.6	Prpf40b	4.85E-01
gene5455	19700	NC_000068.7	Rem1	4.85E-01
gene5925	109054	NC_000068.7	Pfdn4	4.85E-01
gene14004	665024	NC_000072.6	Gm7452	4.84E-01
gene6486	22401	NC_000069.6	Zmat3	4.84E-01
gene2272	71870	NC_000067.6	Cfap45	4.84E-01
gene25957	67270	NC_000076.6	Mrpl42	4.84E-01
gene3023	54519	NC_000068.7	Apbb1ip	4.83E-01
gene35102	18039	NC_000080.6	Nefl	4.83E-01
gene40052	58218	NC_000083.6	Trem3	4.83E-01
gene34862	76007	NC_000080.6	Zmym2	4.83E-01
gene23286	67287	NC_000075.6	Parp6	4.83E-01
gene35906	68073	NC_000081.6	Fam173b	4.83E-01
gene27130	72290	NC_000077.6	Lsm11	4.83E-01
gene18844	75430	NC_000073.6	Anapc15	4.83E-01
gene18707	101861	NC_000073.6	Ints4	4.83E-01
gene27334	237761	NC_000077.6	Sowaha	4.83E-01
gene19749	19267	NC_000073.6	Ptpre	4.83E-01
gene30232	70934	NC_000078.6	4931403G20Rik	4.82E-01
gene27948	94045	NC_000077.6	P2rx5	4.82E-01
gene2045	98376	NC_000067.6	Gorab	4.82E-01
gene24638	19707	NC_000076.6	Reps1	4.82E-01
gene35181	13829	NC_000080.6	Dmtn	4.82E-01
gene34316	1E+08	NC_000080.6	Gm15217	4.82E-01
gene22602	235132	NC_000075.6	Zbtb44	4.81E-01
gene21165	102182	NC_000074.6	Prmt9	4.81E-01
gene36043	239393	NC_000081.6	Lrp12	4.81E-01
gene2803	1E+08	NC_000068.7	Gm31290	4.81E-01
gene36145	18133	NC_000081.6	Nov	4.81E-01
gene22008	22761	NC_000074.6	Zfpm1	4.81E-01
gene23468	320528	NC_000075.6	Vps13c	4.81E-01
gene10872	67087	NC_000070.6	Ctnnbip1	4.81E-01
gene14035	15258	NC_000072.6	Hipk2	4.81E-01

gene16631	637908	NC_000073.6	Vmn2r53	4.80E-01
gene24346	235674	NC_000075.6	Acaa1b	4.80E-01
gene37913	317717	NC_000082.6	Sec22a	4.80E-01
gene12600	381668	NC_000071.6	Fbrsl1	4.80E-01
gene42432	14682	NC_000085.6	Gnaq	4.80E-01
gene27160	276891	NC_000077.6	Timd4	4.80E-01
gene8979	69934	NC_000070.6	Trmt10b	4.80E-01
gene222	1.1E+08	NC_000067.6	Gm26524	4.80E-01
gene11116	100986	NC_000071.6	Akap9	4.80E-01
gene8327	99633	NC_000069.6	Adgrl2	4.80E-01
gene18823	=Gene;ger	NC_000073.6	LOC108167450	4.79E-01
gene20619	52065	NC_000074.6	Mfhas1	4.79E-01
gene4959	14118	NC_000068.7	Fbn1	4.79E-01
gene31072	68020	NC_000078.6	Apopt1	4.79E-01
gene890	319651	NC_000067.6	Usp37	4.79E-01
gene10239	384061	NC_000070.6	Fndc5	4.79E-01
gene5547	56406	NC_000068.7	Ncoa6	4.79E-01
gene39511	77652	NC_000083.6	Zfp955a	4.79E-01
gene21714	214572	NC_000074.6	Prmt7	4.78E-01
gene23448	68318	NC_000075.6	Aph1c	4.78E-01
gene12306	140780	NC_000071.6	Bmp2k	4.78E-01
gene23030	54725	NC_000075.6	Cadm1	4.78E-01
gene21812	234730	NC_000074.6	Fuk	4.77E-01
gene21272	330814	NC_000074.6	Adgrl1	4.77E-01
gene21712	330836	NC_000074.6	Slc7a6	4.77E-01
gene18813	319604	NC_000073.6	Fam168a	4.77E-01
gene20237	234094	NC_000074.6	Arhgef10	4.77E-01
gene24200	270201	NC_000075.6	Klhl18	4.77E-01
gene6954	26879	NC_000069.6	B3galnt1	4.76E-01
gene9761	=Gene;ger	NC_000070.6	LOC105244648	4.76E-01
gene18808	52443	NC_000073.6	Mrpl48	4.76E-01
gene1997	226539	NC_000067.6	Dars2	4.76E-01
gene36383	546643	NC_000081.6	I830127L07Rik	4.76E-01
gene20395	18408	NC_000074.6	Slc25a15	4.76E-01
gene13281	72154	NC_000071.6	Zfp157	4.76E-01
gene41488	225600	NC_000084.6	Pde6a	4.75E-01
gene34063	218885	NC_000080.6	Oxnad1	4.75E-01
gene4864	70933	NC_000068.7	4931402G19Rik	4.75E-01
gene3597	1.1E+08	NC_000068.7	Gm39811	4.75E-01
gene40079	76438	NC_000083.6	Rftn1	4.75E-01
gene38117	73916	NC_000082.6	Ift57	4.74E-01
gene10522	230861	NC_000070.6	Eif4g3	4.74E-01
gene12841	1E+08	NC_000071.6	Gm10390	4.74E-01
gene10841	56717	NC_000070.6	Mtor	4.74E-01
gene14920	1E+08	NC_000072.6	Gm15401	4.74E-01
gene38197	73379	NC_000082.6	Dcbld2	4.74E-01
gene17120	22755	NC_000073.6	Zfp93	4.74E-01
gene40419	12211	NC_000083.6	Birc6	4.74E-01

gene9045	269536	NC_000070.6	Tex10	4.73E-01
gene10173	66938	NC_000070.6	Sh3d21	4.73E-01
gene4444	228366	NC_000068.7	Gyltl1b	4.73E-01
gene13070	69034	NC_000071.6	Nupr1l	4.73E-01
gene30480	18222	NC_000078.6	Numb	4.73E-01
gene39051	381067	NC_000083.6	Zfp229	4.73E-01
gene35774	17117	NC_000081.6	Amacr	4.73E-01
gene35011	219148	NC_000080.6	Fam167a	4.72E-01
gene376	269181	NC_000067.6	Mgat4a	4.72E-01
gene2169	435653	NC_000067.6	Fcrlb	4.72E-01
gene23367	17127	NC_000075.6	Smad3	4.72E-01
gene7587	108097	NC_000069.6	Prkab2	4.71E-01
gene23443	74165	NC_000075.6	Fbxl22	4.71E-01
gene39441	224674	NC_000083.6	Slc37a1	4.71E-01
gene8806	66408	NC_000070.6	Aptx	4.71E-01
gene33261	544971	NC_000079.6	Bdp1	4.71E-01
gene10237	66260	NC_000070.6	Tmem54	4.71E-01
gene941	20536	NC_000067.6	Slc4a3	4.71E-01
gene32338	30938	NC_000079.6	Fgd3	4.71E-01
gene42485	66206	NC_000085.6	1110059E24Rik	4.71E-01
gene31076	21981	NC_000078.6	Ppp1r13b	4.71E-01
gene28249	1E+08	NC_000077.6	Unc45bos	4.70E-01
gene36815	239570	NC_000081.6	Ttc38	4.70E-01
gene13127	79565	NC_000071.6	Wbscr27	4.70E-01
gene1441	81879	NC_000067.6	Tfcp2l1	4.70E-01
gene2801	1E+08	NC_000068.7	Gm13262	4.70E-01
gene13259	56314	NC_000071.6	Zfp113	4.70E-01
gene20861	71306	NC_000074.6	Mfap3l	4.70E-01
gene37907	76649	NC_000082.6	1700119H24Rik	4.70E-01
gene27882	193043	NC_000077.6	Zfp3	4.70E-01
gene23095	1E+08	NC_000075.6	Gm32681	4.69E-01
gene4796	12236	NC_000068.7	Bub1b	4.69E-01
gene12983	67848	NC_000071.6	Ddx55	4.69E-01
gene19714	72096	NC_000073.6	Mettl10	4.68E-01
gene36860	69120	NC_000081.6	1810021B22Rik	4.68E-01
gene14268	101197	NC_000072.6	Zfp956	4.68E-01
gene37996	1E+08	NC_000082.6	Gm36482	4.68E-01
gene13104	94254	NC_000071.6	Wbscr16	4.68E-01
gene11278	14677	NC_000071.6	Gnai1	4.68E-01
gene27832	13385	NC_000077.6	Dlg4	4.68E-01
gene9882	76786	NC_000070.6	2510003B16Rik	4.68E-01
gene2053	381306	NC_000067.6	BC055324	4.67E-01
gene38515	67367	NC_000082.6	Paxbp1	4.67E-01
gene34420	1E+08	NC_000080.6	Gm26782	4.67E-01
gene25171	216001	NC_000076.6	Micu1	4.67E-01
gene17395	233060	NC_000073.6	Zfp382	4.66E-01
gene7480	75137	NC_000069.6	Rprd2	4.66E-01
gene34087	545030	NC_000080.6	Wdfy4	4.66E-01

gene5561	17391	NC_000068.7	Mmp24	4.66E-01
gene18406	76375	NC_000073.6	Det1	4.66E-01
gene34850	328417	NC_000080.6	Parp4	4.66E-01
gene12123	53315	NC_000071.6	Sult1d1	4.66E-01
gene7099	27059	NC_000069.6	Sh3d19	4.66E-01
gene12469	54367	NC_000071.6	Zfp326	4.66E-01
gene24663	18634	NC_000076.6	Pex7	4.65E-01
gene10829	1E+08	NC_000070.6	Gm13201	4.65E-01
gene27923	21689	NC_000077.6	Tekt1	4.64E-01
gene693	77300	NC_000067.6	Raph1	4.64E-01
gene26277	380664	NC_000076.6	Lemd3	4.64E-01
gene40219	22153	NC_000083.6	Tubb4a	4.64E-01
gene40453	1.1E+08	NC_000083.6	Gm26637	4.64E-01
gene24916	14360	NC_000076.6	Fyn	4.64E-01
gene5841	77031	NC_000068.7	Slc9a8	4.64E-01
gene37738	20440	NC_000082.6	St6gal1	4.64E-01
gene27109	70802	NC_000077.6	Pwwp2a	4.64E-01
gene3162	56279	NC_000068.7	Fam69b	4.64E-01
gene5600	1E+08	NC_000068.7	Gm14230	4.64E-01
gene38602	13548	NC_000082.6	Dyrk1a	4.64E-01
gene25306	216049	NC_000076.6	Zfp365	4.63E-01
gene7713	99470	NC_000069.6	Magi3	4.63E-01
gene37446	50505	NC_000082.6	Ercc4	4.63E-01
gene11155	27214	NC_000071.6	Dbf4	4.63E-01
gene31029	668245	NC_000078.6	Gm9063	4.63E-01
gene32179	12161	NC_000079.6	Bmp6	4.63E-01
gene18803	69387	NC_000073.6	Dnajb13	4.63E-01
gene42935	107338	NC_000085.6	Gbf1	4.63E-01
gene21170	13617	NC_000074.6	Ednra	4.63E-01
gene28030	116905	NC_000077.6	Dph1	4.62E-01
gene2620	77065	NC_000067.6	Ints7	4.62E-01
gene28016	67493	NC_000077.6	Mettl16	4.62E-01
gene39465	1E+08	NC_000083.6	Gm38585	4.62E-01
gene8295	68279	NC_000069.6	Mcoln2	4.62E-01
gene40818	75176	NC_000084.6	4930543D07Rik	4.62E-01
gene30073	217578	NC_000078.6	Baz1a	4.62E-01
gene19599	330657	NC_000073.6	Prss53	4.62E-01
gene34	654788	NC_000067.6	4732440D04Rik	4.61E-01
gene4387	59015	NC_000068.7	Nup160	4.61E-01
gene15258	232236	NC_000072.6	Ccdc174	4.61E-01
gene23290	71599	NC_000075.6	Senp8	4.61E-01
gene23823	235527	NC_000075.6	Plscr4	4.61E-01
gene24344	18799	NC_000075.6	Plcd1	4.61E-01
gene5716	11486	NC_000068.7	Ada	4.60E-01
gene35059	52033	NC_000080.6	Pbk	4.60E-01
gene39050	240034	NC_000083.6	Zfp760	4.60E-01
gene25250	327762	NC_000076.6	Dna2	4.60E-01
gene38245	56297	NC_000082.6	Arl6	4.60E-01

gene9718	=Gene;ger	NC_000070.6	LOC108168970	4.59E-01
gene17722	67607	NC_000073.6	Zfp788	4.59E-01
gene33160	11774	NC_000079.6	Ap3b1	4.59E-01
gene17965	73813	NC_000073.6	Fam83e	4.59E-01
gene3329	99011	NC_000068.7	Pomt1	4.59E-01
gene1920	19775	NC_000067.6	Xpr1	4.59E-01
gene10317	26922	NC_000070.6	Mecr	4.59E-01
gene32050	105352	NC_000079.6	Dusp22	4.59E-01
gene34851	1E+08	NC_000080.6	Gm16573	4.59E-01
gene25134	15500	NC_000076.6	Hsf2	4.59E-01
gene1669	240756	NC_000067.6	Klhl12	4.58E-01
gene21653	26386	NC_000074.6	Hsf4	4.58E-01
gene32577	1E+08	NC_000079.6	Gm38494	4.58E-01
gene42950	94279	NC_000085.6	Sfxn2	4.58E-01
gene15088	66116	NC_000072.6	Nat8f1	4.58E-01
gene427	16177	NC_000067.6	Il1r1	4.58E-01
gene27613	69747	NC_000077.6	Zswim7	4.58E-01
gene7264	78284	NC_000069.6	Creb3l4	4.58E-01
gene31411	20688	NC_000078.6	Sp4	4.58E-01
gene22445	70726	NC_000075.6	Angptl6	4.58E-01
gene14427	12417	NC_000072.6	Cbx3	4.58E-01
gene14359	76507	NC_000072.6	Aoc1	4.57E-01
gene41946	319314	NC_000085.6	A930001C03Rik	4.57E-01
gene16864	232934	NC_000073.6	Mypop	4.57E-01
gene28878	51799	NC_000077.6	Rundc3a	4.57E-01
gene482	75623	NC_000067.6	Tex30	4.57E-01
gene5892	22722	NC_000068.7	Zfp64	4.57E-01
gene20181	259279	NC_000074.6	Tubgcp3	4.57E-01
gene32991	218341	NC_000079.6	Rfesd	4.57E-01
gene4911	329506	NC_000068.7	Ctdspl2	4.57E-01
gene25586	114229	NC_000076.6	Kiss1r	4.57E-01
gene33140	57748	NC_000079.6	Jmy	4.56E-01
gene10837	230904	NC_000070.6	Fbxo2	4.56E-01
gene21780	75871	NC_000074.6	Zfp821	4.55E-01
gene1706	226438	NC_000067.6	Igfn1	4.55E-01
gene38755	1E+08	NC_000083.6	Gm33799	4.55E-01
gene38641	50528	NC_000082.6	Tmprss2	4.55E-01
gene40949	225326	NC_000084.6	Pik3c3	4.55E-01
gene28969	24086	NC_000077.6	Tlk2	4.54E-01
gene885	241112	NC_000067.6	Catip	4.54E-01
gene31511	667727	NC_000079.6	Gm8784	4.54E-01
gene14614	54486	NC_000072.6	Hpgds	4.54E-01
gene40354	=Gene;ger	NC_000083.6	LOC108168339	4.54E-01
gene19571	654803	NC_000073.6	B130055M24Rik	4.54E-01
gene7202	192195	NC_000069.6	Ash1l	4.54E-01
gene36639	106059	NC_000081.6	A430088P11Rik	4.54E-01
gene35755	75568	NC_000081.6	Capsl	4.53E-01
gene17115	56525	NC_000073.6	Zfp235	4.53E-01

gene2521	98732	NC_000067.6	Rab3gap2	4.53E-01
gene33421	1.1E+08	NC_000079.6	Gm15286	4.53E-01
gene42509	72351	NC_000085.6	Ptar1	4.53E-01
gene36562	57028	NC_000081.6	Pdxp	4.53E-01
gene8360	108946	NC_000069.6	Zzz3	4.52E-01
gene5629	75425	NC_000068.7	Tti1	4.52E-01
gene15260	232237	NC_000072.6	Fgd5	4.52E-01
gene2359	545389	NC_000067.6	Cep170	4.52E-01
gene38330	268903	NC_000082.6	Nrip1	4.52E-01
gene37732	106344	NC_000082.6	Rfc4	4.52E-01
gene27488	56631	NC_000077.6	Trim17	4.51E-01
gene5107	11990	NC_000068.7	Atrn	4.51E-01
gene8165	73284	NC_000069.6	Ddit4l	4.51E-01
gene3746	14425	NC_000068.7	Galnt3	4.51E-01
gene28884	237943	NC_000077.6	Gpatch8	4.51E-01
gene3494	227800	NC_000068.7	Rabgap1	4.51E-01
gene39235	71840	NC_000083.6	Tekt4	4.51E-01
gene26258	73914	NC_000076.6	Irak3	4.51E-01
gene3135	227634	NC_000068.7	Camsap1	4.51E-01
gene21728	15118	NC_000074.6	Has3	4.51E-01
gene12192	11839	NC_000071.6	Areg	4.50E-01
gene4851	26390	NC_000068.7	Mapkbp1	4.50E-01
gene9189	72429	NC_000070.6	Dnajc25	4.50E-01
gene23208	72588	NC_000075.6	2700012I20Rik	4.50E-01
gene2396	226744	NC_000067.6	Cnst	4.50E-01
gene1271	67026	NC_000067.6	Thap4	4.50E-01
gene23092	102580	NC_000075.6	Alg9	4.50E-01
gene7105	1.1E+08	NC_000069.6	Gm40083	4.50E-01
gene41352	67222	NC_000084.6	Srfbp1	4.50E-01
gene23407	214425	NC_000075.6	Cilp	4.50E-01
gene7637	14547	NC_000069.6	Gdap2	4.49E-01
gene40494	225028	NC_000083.6	Map4k3	4.49E-01
gene14585	243385	NC_000072.6	Gprin3	4.49E-01
gene1934	170484	NC_000067.6	Nphs2	4.48E-01
gene37311	76779	NC_000082.6	Cluap1	4.48E-01
gene636	11761	NC_000067.6	Aox1	4.48E-01
gene33105	1.1E+08	NC_000079.6	Gm41014	4.48E-01
gene18478	434203	NC_000073.6	Slc28a1	4.48E-01
gene22144	68865	NC_000074.6	Arv1	4.48E-01
gene5803	228880	NC_000068.7	Zmynd8	4.48E-01
gene42000	74931	NC_000085.6	4930481A15Rik	4.48E-01
gene29851	238123	NC_000078.6	Cog5	4.48E-01
gene15846	1.1E+08	NC_000072.6	Gm38905	4.48E-01
gene26373	216441	NC_000076.6	Slc26a10	4.48E-01
gene73	73331	NC_000067.6	1700034P13Rik	4.48E-01
gene3369	227738	NC_000068.7	Lrsam1	4.48E-01
gene30262	238247	NC_000078.6	Arid4a	4.47E-01
gene25590	170483	NC_000076.6	Grin3b	4.47E-01

gene26637	67178	NC_000077.6	Zmat5	4.47E-01
gene38571	1E+08	NC_000082.6	Gm30474	4.47E-01
gene6184	72147	NC_000068.7	Zbtb46	4.47E-01
gene5017	53885	NC_000068.7	Nphp1	4.47E-01
gene2991	76467	NC_000068.7	Msrb2	4.46E-01
gene2095	12503	NC_000067.6	Cd247	4.46E-01
gene2215	240913	NC_000067.6	Adamts4	4.46E-01
gene19412	233812	NC_000073.6	BC030336	4.46E-01
gene21802	234729	NC_000074.6	Vac14	4.46E-01
gene28127	140859	NC_000077.6	Nek8	4.45E-01
gene11442	1E+08	NC_000071.6	Gm35223	4.45E-01
gene16426	232855	NC_000073.6	Zfp772	4.45E-01
gene19492	104175	NC_000073.6	Sbk1	4.45E-01
gene21195	17125	NC_000074.6	Smad1	4.45E-01
gene41834	110796	NC_000084.6	Tshz1	4.45E-01
gene12695	22256	NC_000071.6	Ung	4.45E-01
gene5012	77721	NC_000068.7	Mrps5	4.45E-01
gene39492	240064	NC_000083.6	Zfp799	4.45E-01
gene28785	278304	NC_000077.6	Zfp385c	4.45E-01
gene25795	12952	NC_000076.6	Cry1	4.45E-01
gene20015	101772	NC_000073.6	Ano1	4.45E-01
gene42080	1.1E+08	NC_000085.6	Gm14965	4.45E-01
gene13277	1E+08	NC_000071.6	Pvrig	4.44E-01
gene1778	214498	NC_000067.6	Cdc73	4.44E-01
gene24851	69692	NC_000076.6	Hddc2	4.44E-01
gene2742	1E+08	NC_000068.7	Gm13179	4.44E-01
gene40619	77057	NC_000083.6	Ston1	4.44E-01
gene28940	18195	NC_000077.6	Nsf	4.44E-01
gene41897	1E+08	NC_000085.6	Gm36787	4.43E-01
gene37554	72315	NC_000082.6	Ccdc74a	4.43E-01
gene40896	328918	NC_000084.6	Zscan30	4.43E-01
gene10057	109263	NC_000070.6	Rlf	4.43E-01
gene8162	59308	NC_000069.6	Emcn	4.43E-01
gene9568	77963	NC_000070.6	Hook1	4.43E-01
gene5594	228836	NC_000068.7	Dlgap4	4.43E-01
gene4382	19271	NC_000068.7	Ptprj	4.43E-01
gene20921	18166	NC_000074.6	Npy1r	4.43E-01
gene10230	67525	NC_000070.6	Trim62	4.43E-01
gene460	69527	NC_000067.6	Mrps9	4.43E-01
gene37236	223922	NC_000081.6	Atf7	4.42E-01
gene3493	320633	NC_000068.7	Zbtb26	4.42E-01
gene26481	14561	NC_000076.6	Gdf11	4.42E-01
gene21906	74440	NC_000074.6	Cmip	4.42E-01
gene39428	14652	NC_000083.6	Glp1r	4.42E-01
gene37024	18642	NC_000081.6	Pfkm	4.41E-01
gene22531	235050	NC_000075.6	Zfp810	4.41E-01
gene1054	319997	NC_000067.6	A630001G21Rik	4.41E-01
gene17831	16612	NC_000073.6	Klk1	4.41E-01

gene42713	16551	NC_000085.6	Kif11	4.40E-01
gene8120	74776	NC_000069.6	Ppa2	4.40E-01
gene36586	53357	NC_000081.6	Pla2g6	4.40E-01
gene485	22592	NC_000067.6	Ercc5	4.40E-01
gene33774	218756	NC_000080.6	Slc4a7	4.39E-01
gene5824	76367	NC_000068.7	Trp53rkb	4.39E-01
gene37606	15260	NC_000082.6	Hira	4.39E-01
gene37098	380969	NC_000081.6	Nckap5l	4.39E-01
gene18863	20866	NC_000073.6	Stim1	4.39E-01
gene30217	76062	NC_000078.6	5830428M24Rik	4.39E-01
gene3038	140483	NC_000068.7	Hnmt	4.39E-01
gene28257	237887	NC_000077.6	Slfn10-ps	4.39E-01
gene2433	13590	NC_000067.6	Lefty1	4.38E-01
gene16645	243833	NC_000073.6	Zfp128	4.38E-01
gene4591	99003	NC_000068.7	Qser1	4.38E-01
gene34291	70561	NC_000080.6	Txndc16	4.38E-01
gene31139	238447	NC_000078.6	Igha	4.38E-01
gene4389	55935	NC_000068.7	Fnbp4	4.37E-01
gene28609	=Gene;ger	NC_000077.6	LOC108167924	4.37E-01
gene15896	16634	NC_000072.6	Klra3	4.37E-01
gene7821	26442	NC_000069.6	Psma5	4.37E-01
gene26044	237504	NC_000076.6	Rassf9	4.37E-01
gene28923	53859	NC_000077.6	Map3k14	4.36E-01
gene28952	76719	NC_000077.6	Kansl1	4.36E-01
gene7796	329735	NC_000069.6	4933431E20Rik	4.36E-01
gene39504	240068	NC_000083.6	Zfp563	4.36E-01
gene25884	11783	NC_000076.6	Apaf1	4.36E-01
gene35685	12274	NC_000081.6	C6	4.36E-01
gene40000	14711	NC_000083.6	Gnmt	4.36E-01
gene37482	17250	NC_000082.6	Abcc1	4.36E-01
gene29504	68832	NC_000078.6	Ldah	4.36E-01
gene30524	56531	NC_000078.6	Ylpm1	4.36E-01
gene34028	16424	NC_000080.6	Itih1	4.36E-01
gene28926	653030	NC_000077.6	Arhgap27os3	4.36E-01
gene6284	66361	NC_000069.6	Zfand1	4.35E-01
gene41610	225659	NC_000084.6	Cep76	4.35E-01
gene27045	319208	NC_000077.6	4930403D09Rik	4.35E-01
gene37449	1E+08	NC_000082.6	Gm15738	4.35E-01
gene13136	194309	NC_000071.6	Vps37d	4.34E-01
gene24925	213332	NC_000076.6	Mfsd4b4	4.34E-01
gene12658	320129	NC_000071.6	Adrbk2	4.34E-01
gene34030	23955	NC_000080.6	Nek4	4.34E-01
gene24228	71268	NC_000075.6	Lrrfip2	4.34E-01
gene33358	16563	NC_000079.6	Kif2a	4.34E-01
gene4896	269344	NC_000068.7	Ell3	4.34E-01
gene41785	67655	NC_000084.6	Ctdp1	4.33E-01
gene16860	232933	NC_000073.6	Ccdc61	4.33E-01
gene37983	64082	NC_000082.6	Popdc2	4.33E-01

gene2309	1E+08	NC_000067.6	Gm16340	4.33E-01
gene40694	21417	NC_000084.6	Zeb1	4.33E-01
gene15705	13807	NC_000072.6	Eno2	4.33E-01
gene33422	218613	NC_000079.6	Mier3	4.33E-01
gene40556	72416	NC_000083.6	Lrpprc	4.33E-01
gene9556	320713	NC_000070.6	Mysm1	4.33E-01
gene29401	209588	NC_000077.6	Sectm1a	4.32E-01
gene2930	17533	NC_000068.7	Mrc1	4.32E-01
gene1108	227331	NC_000067.6	Gigyf2	4.32E-01
gene13523	11987	NC_000071.6	Slc7a1	4.32E-01
gene6186	329584	NC_000068.7	Slc2a4rg-ps	4.31E-01
gene1998	70454	NC_000067.6	Cenpl	4.31E-01
gene7044	78212	NC_000069.6	Rbm46os	4.30E-01
gene12995	73121	NC_000071.6	Fam101a	4.30E-01
gene12484	52397	NC_000071.6	Zfp644	4.30E-01
gene10248	=Gene;ger	NC_000070.6	LOC108168979	4.30E-01
gene41335	240283	NC_000084.6	Dmxl1	4.30E-01
gene26159	216345	NC_000076.6	Zfc3h1	4.29E-01
gene19515	75565	NC_000073.6	Sgf29	4.29E-01
gene9941	14664	NC_000070.6	Slc6a9	4.29E-01
gene39462	72462	NC_000083.6	Rrp1b	4.29E-01
gene27509	74476	NC_000077.6	4933439C10Rik	4.29E-01
gene36818	12614	NC_000081.6	Celsr1	4.29E-01
gene24644	56016	NC_000076.6	Hebp2	4.29E-01
gene299	213539	NC_000067.6	Bag2	4.29E-01
gene9548	667250	NC_000070.6	Gm12657	4.28E-01
gene24611	=Gene;ger	NC_000076.6	LOC108167795	4.28E-01
gene16881	319197	NC_000073.6	Gpr4	4.28E-01
gene32503	432763	NC_000079.6	Prr7	4.28E-01
gene34104	105518	NC_000080.6	A630023A22Rik	4.28E-01
gene39276	215445	NC_000083.6	Rab11fip3	4.28E-01
gene13928	54160	NC_000072.6	Copg2	4.28E-01
gene366	56030	NC_000067.6	Tmem131	4.28E-01
gene17439	58206	NC_000073.6	Zbtb32	4.28E-01
gene30529	18654	NC_000078.6	Pgf	4.27E-01
gene24581	268281	NC_000076.6	Shprh	4.27E-01
gene13055	231769	NC_000071.6	Sfswap	4.27E-01
gene29468	74855	NC_000078.6	Fam228a	4.27E-01
gene4556	13711	NC_000068.7	Elf5	4.27E-01
gene21607	75458	NC_000074.6	Cklf	4.27E-01
gene38724	1E+08	NC_000083.6	Dynlt1f	4.27E-01
gene14062	14548	NC_000072.6	Mrps33	4.27E-01
gene2653	215193	NC_000067.6	Diexf	4.27E-01
gene15229	66277	NC_000072.6	Klf15	4.27E-01
gene24325	67899	NC_000075.6	Cmc1	4.26E-01
gene12516	67266	NC_000071.6	Fam69a	4.26E-01
gene8220	56376	NC_000069.6	Pdlim5	4.26E-01
gene30332	26932	NC_000078.6	Ppp2r5e	4.26E-01

gene5228	66580	NC_000068.7	Esf1	4.26E-01
gene1650	14264	NC_000067.6	Fmod	4.26E-01
gene14005	208647	NC_000072.6	Creb3l2	4.25E-01
gene17122	22719	NC_000073.6	Zfp61	4.25E-01
gene37068	69159	NC_000081.6	Rhebl1	4.25E-01
gene10255	433759	NC_000070.6	Hdac1	4.25E-01
gene7651	74044	NC_000069.6	Ttf2	4.25E-01
gene1002	12829	NC_000067.6	Col4a4	4.25E-01
gene668	74018	NC_000067.6	Als2	4.24E-01
gene33478	17993	NC_000079.6	Ndufs4	4.24E-01
gene29761	1E+08	NC_000078.6	Gm36236	4.24E-01
gene13338	74239	NC_000071.6	Iqce	4.23E-01
gene5056	70466	NC_000068.7	Ckap2l	4.23E-01
gene18379	108116	NC_000073.6	Slco3a1	4.23E-01
gene4913	214585	NC_000068.7	Spg11	4.23E-01
gene28274	237891	NC_000077.6	Gas2l2	4.23E-01
gene12234	27428	NC_000071.6	Shroom3	4.23E-01
gene28882	217219	NC_000077.6	Fam171a2	4.23E-01
gene37813	224090	NC_000082.6	Tmem44	4.23E-01
gene19860	13106	NC_000073.6	Cyp2e1	4.22E-01
gene23192	66867	NC_000075.6	Hmg20a	4.22E-01
gene21224	1E+08	NC_000074.6	Gm31223	4.22E-01
gene11854	212285	NC_000071.6	Arap2	4.22E-01
gene21012	13716	NC_000074.6	Ell	4.22E-01
gene23702	212531	NC_000075.6	Sh3bgrl2	4.22E-01
gene14613	13990	NC_000072.6	Smarcad1	4.22E-01
gene21868	67528	NC_000074.6	Nudt7	4.22E-01
gene9671	67013	NC_000070.6	Oma1	4.21E-01
gene6020	13616	NC_000068.7	Edn3	4.21E-01
gene19232	76954	NC_000073.6	St5	4.21E-01
gene28244	67338	NC_000077.6	Rffl	4.21E-01
gene41513	319713	NC_000084.6	Ablim3	4.21E-01
gene34958	668253	NC_000080.6	Dleu2	4.21E-01
gene16326	77891	NC_000073.6	Ube2s	4.21E-01
gene33200	320557	NC_000079.6	Fam169a	4.21E-01
gene9787	230603	NC_000070.6	Ttc39a	4.20E-01
gene10129	1E+08	NC_000070.6	Gm12915	4.20E-01
gene25038	78977	NC_000076.6	Popdc3	4.20E-01
gene17386	72723	NC_000073.6	Zfp74	4.20E-01
gene16671	321008	NC_000073.6	6330408A02Rik	4.20E-01
gene25739	237411	NC_000076.6	Zfp938	4.20E-01
gene23522	19378	NC_000075.6	Aldh1a2	4.20E-01
gene1197	51801	NC_000067.6	Ramp1	4.19E-01
gene41714	1E+08	NC_000084.6	Gm10532	4.19E-01
gene37881	70144	NC_000082.6	Lrch3	4.19E-01
gene24874	66521	NC_000076.6	Rwdd1	4.19E-01
gene19244	20841	NC_000073.6	Zfp143	4.18E-01
gene7218	360213	NC_000069.6	Trim46	4.18E-01

gene9935	1E+08	NC_000070.6	Gm34746	4.18E-01
gene24018	66595	NC_000075.6	Aste1	4.18E-01
gene4484	329470	NC_000068.7	Accs	4.18E-01
gene9446	329877	NC_000070.6	Dennd4c	4.18E-01
gene22174	16525	NC_000074.6	Kcnk1	4.17E-01
gene24640	215814	NC_000076.6	Ccdc28a	4.17E-01
gene30230	399510	NC_000078.6	Map4k5	4.17E-01
gene2928	20844	NC_000068.7	Stam	4.17E-01
gene41894	1E+08	NC_000085.6	Gm36672	4.17E-01
gene33912	218832	NC_000080.6	Polr3a	4.17E-01
gene14357	666105	NC_000072.6	Gm7932	4.17E-01
gene42221	54525	NC_000085.6	Syt7	4.17E-01
gene41988	107975	NC_000085.6	Pacs1	4.17E-01
gene11118	1.1E+08	NC_000071.6	Gm40260	4.16E-01
gene22121	234865	NC_000074.6	Nup133	4.16E-01
gene42813	107094	NC_000085.6	Rrp12	4.16E-01
gene6847	1E+08	NC_000069.6	Gm33923	4.16E-01
gene36076	66736	NC_000081.6	Emc2	4.16E-01
gene3093	72080	NC_000068.7	Sapcd2	4.15E-01
gene19928	74180	NC_000073.6	Muc5b	4.15E-01
gene10246	67106	NC_000070.6	Zbtb8os	4.15E-01
gene42524	1E+08	NC_000085.6	Gm38581	4.15E-01
gene37328	66449	NC_000082.6	Pam16	4.15E-01
gene9888	18710	NC_000070.6	Pik3r3	4.14E-01
gene590	98488	NC_000067.6	Gtf3c3	4.14E-01
gene23418	69606	NC_000075.6	Mtfmt	4.14E-01
gene38514	67394	NC_000082.6	4930404I05Rik	4.14E-01
gene19578	1.1E+08	NC_000073.6	Tmem265	4.14E-01
gene20388	80986	NC_000074.6	Ckap2	4.14E-01
gene37889	106326	NC_000082.6	Osbpl11	4.14E-01
gene39448	108121	NC_000083.6	U2af1	4.13E-01
gene41966	52028	NC_000085.6	Bbs1	4.13E-01
gene32076	20706	NC_000079.6	Serpinb9b	4.13E-01
gene4863	76051	NC_000068.7	Ganc	4.13E-01
gene6313	13559	NC_000069.6	E2f5	4.13E-01
gene2273	71319	NC_000067.6	4933439K11Rik	4.13E-01
gene17598	435965	NC_000073.6	Lrp3	4.13E-01
gene30243	1E+08	NC_000078.6	Gm38467	4.13E-01
gene36941	106042	NC_000081.6	Prickle1	4.13E-01
gene11342	72772	NC_000071.6	Rint1	4.13E-01
gene23861	211586	NC_000075.6	Tfdp2	4.13E-01
gene5701	66680	NC_000068.7	Oser1	4.12E-01
gene15027	11468	NC_000072.6	Actg2	4.12E-01
gene25456	20509	NC_000076.6	Slc19a1	4.12E-01
gene30306	625098	NC_000078.6	Slc38a6	4.12E-01
gene23170	109161	NC_000075.6	Ube2q2	4.12E-01
gene15108	232196	NC_000072.6	C87436	4.12E-01
gene3803	66748	NC_000068.7	Erich2	4.12E-01

gene27258	30944	NC_000077.6	Zfp354c	4.12E-01
gene7765	321000	NC_000069.6	Lrif1	4.12E-01
gene28951	17762	NC_000077.6	Mapt	4.11E-01
gene40522	78798	NC_000083.6	Eml4	4.11E-01
gene4021	672763	NC_000068.7	Gm13710	4.11E-01
gene2404	67426	NC_000067.6	Adck3	4.11E-01
gene6817	242050	NC_000069.6	Igsf10	4.11E-01
gene32112	73102	NC_000079.6	Slc22a23	4.11E-01
gene24431	102566	NC_000075.6	Ano10	4.11E-01
gene18365	69170	NC_000073.6	1810026B05Rik	4.11E-01
gene15736	320678	NC_000072.6	Iffo1	4.11E-01
gene27523	103850	NC_000077.6	Nt5m	4.11E-01
gene22507	71746	NC_000075.6	Rgl3	4.10E-01
gene25285	108829	NC_000076.6	Jmjd1c	4.10E-01
gene30798	65256	NC_000078.6	Asb2	4.10E-01
gene3527	76899	NC_000068.7	Golga1	4.10E-01
gene38108	224171	NC_000082.6	C330027C09Rik	4.10E-01
gene35741	20512	NC_000081.6	Slc1a3	4.10E-01
gene4821	320415	NC_000068.7	Gchfr	4.09E-01
gene2970	13418	NC_000068.7	Dnajc1	4.09E-01
gene40199	18188	NC_000083.6	Nrtn	4.09E-01
gene580	72085	NC_000067.6	Osgepl1	4.09E-01
gene10618	213649	NC_000070.6	Arhgef19	4.09E-01
gene12785	665073	NC_000071.6	Gm7478	4.08E-01
gene75	170755	NC_000067.6	Sgk3	4.08E-01
gene7027	1E+08	NC_000069.6	Gm29808	4.08E-01
gene10329	56809	NC_000070.6	Gmeb1	4.08E-01
gene28444	71943	NC_000077.6	Tom1l1	4.08E-01
gene13806	269831	NC_000072.6	Tspan12	4.08E-01
gene24425	245050	NC_000075.6	Fam198a	4.08E-01
gene24687	56422	NC_000076.6	Hbs1l	4.08E-01
gene22652	109229	NC_000075.6	Fam118b	4.08E-01
gene21541	73407	NC_000074.6	Tepp	4.08E-01
gene37008	71919	NC_000081.6	Rpap3	4.08E-01
gene34984	18130	NC_000080.6	Ints6	4.07E-01
gene14869	232078	NC_000072.6	Thnsl2	4.07E-01
gene22964	71687	NC_000075.6	Tmem25	4.07E-01
gene33337	108154	NC_000079.6	Adamts6	4.07E-01
gene18512	330577	NC_000073.6	Saxo2	4.07E-01
gene9203	209131	NC_000070.6	Snx30	4.07E-01
gene35060	71988	NC_000080.6	Esco2	4.07E-01
gene23542	17427	NC_000075.6	Mns1	4.07E-01
gene28421	12812	NC_000077.6	Coil	4.07E-01
gene17780	75690	NC_000073.6	Vsig10l	4.06E-01
gene27002	17168	NC_000077.6	Nprl3	4.06E-01
gene27614	72795	NC_000077.6	Ttc19	4.06E-01
gene14390	71720	NC_000072.6	Osbpl3	4.06E-01
gene18746	22411	NC_000073.6	Wnt11	4.06E-01

gene41968	240518	NC_000085.6	Peli3	4.05E-01
gene17602	67471	NC_000073.6	Gpatch1	4.05E-01
gene14326	77827	NC_000072.6	Krba1	4.05E-01
gene35165	19057	NC_000080.6	Ppp3cc	4.05E-01
gene24338	1.1E+08	NC_000075.6	Gm39454	4.05E-01
gene32415	66197	NC_000079.6	Cks2	4.05E-01
gene23713	=Gene;ger	NC_000075.6	LOC108167669	4.05E-01
gene37550	74685	NC_000082.6	Lrrc74b	4.05E-01
gene21273	1E+08	NC_000074.6	Gm10644	4.04E-01
gene3147	227644	NC_000068.7	Snapc4	4.04E-01
gene38587	110749	NC_000082.6	Chaf1b	4.04E-01
gene41306	11777	NC_000084.6	Ap3s1	4.04E-01
gene6094	665211	NC_000068.7	Gm14326	4.03E-01
gene21536	1E+08	NC_000074.6	Gm31224	4.03E-01
gene24607	15273	NC_000076.6	Hivep2	4.03E-01
gene3549	70789	NC_000068.7	Kynu	4.03E-01
gene12392	74596	NC_000071.6	Cds1	4.03E-01
gene13664	20392	NC_000072.6	Sgce	4.03E-01
gene521	227059	NC_000067.6	Slc39a10	4.02E-01
gene42871	72502	NC_000085.6	Cwf19l1	4.02E-01
gene1941	11352	NC_000067.6	Abl2	4.02E-01
gene417	263764	NC_000067.6	Creg2	4.02E-01
gene20826	382010	NC_000074.6	Cep44	4.02E-01
gene3584	227867	NC_000068.7	Epc2	4.02E-01
gene21193	109136	NC_000074.6	Mmaa	4.02E-01
gene13371	108086	NC_000071.6	Rnf216	4.02E-01
gene36693	338368	NC_000081.6	Fam109b	4.01E-01
gene9635	242570	NC_000070.6	Raver2	4.01E-01
gene18364	244059	NC_000073.6	Chd2	4.01E-01
gene5466	72119	NC_000068.7	Tpx2	4.01E-01
gene23119	244871	NC_000075.6	Zc3h12c	4.01E-01
gene39578	547431	NC_000083.6	Btnl2	4.01E-01
gene26090	114774	NC_000076.6	Pawr	4.01E-01
gene10409	67586	NC_000070.6	Ubxn11	4.01E-01
gene40099	1E+08	NC_000083.6	Gm19585	4.01E-01
gene20719	14107	NC_000074.6	Fat1	4.01E-01
gene11132	1.1E+08	NC_000071.6	Gm40262	4.00E-01
gene5544	228812	NC_000068.7	Pigu	4.00E-01
gene29337	12009	NC_000077.6	Cep131	4.00E-01
gene33164	21371	NC_000079.6	Tbca	4.00E-01
gene36329	1.1E+08	NC_000081.6	Gm41344	4.00E-01
gene38280	73569	NC_000082.6	Vgll3	4.00E-01
gene33128	382793	NC_000079.6	Mtx3	4.00E-01
gene29007	21763	NC_000077.6	Tex2	3.99E-01
gene23930	623534	NC_000075.6	Nme9	3.99E-01
gene17118	56869	NC_000073.6	Zfp109	3.99E-01
gene14264	272347	NC_000072.6	Zfp398	3.99E-01
gene41868	225825	NC_000084.6	Cd226	3.99E-01

gene18702	381903	NC_000073.6	Alg8	3.99E-01
gene29140	70355	NC_000077.6	Gprc5c	3.99E-01
gene13357	1.1E+08	NC_000071.6	Gm38732	3.98E-01
gene2341	23893	NC_000067.6	Grem2	3.98E-01
gene37955	68306	NC_000082.6	4930565N06Rik	3.98E-01
gene27847	11684	NC_000077.6	Alox12	3.98E-01
gene638	1.1E+08	NC_000067.6	Gm39643	3.98E-01
gene14902	71093	NC_000072.6	Atoh8	3.98E-01
gene41372	70425	NC_000084.6	Csnk1g3	3.98E-01
gene9288	326623	NC_000070.6	Tnfsf15	3.97E-01
gene37221	105988	NC_000081.6	Espl1	3.97E-01
gene36429	1.1E+08	NC_000081.6	Gm41349	3.97E-01
gene40966	269003	NC_000084.6	Sap130	3.97E-01
gene13397	76800	NC_000071.6	Usp42	3.97E-01
gene10433	230815	NC_000070.6	Man1c1	3.97E-01
gene21131	74166	NC_000074.6	Tmem38a	3.97E-01
gene25180	69090	NC_000076.6	Ascc1	3.97E-01
gene3399	1E+08	NC_000068.7	Gm34372	3.97E-01
gene20218	=Gene;ger	NC_000074.6	LOC108167523	3.96E-01
gene4889	17754	NC_000068.7	Map1a	3.96E-01
gene27115	79560	NC_000077.6	Ublcp1	3.96E-01
gene7728	229681	NC_000069.6	St7l	3.96E-01
gene4920	20322	NC_000068.7	Sord	3.96E-01
gene21110	209488	NC_000074.6	Hsh2d	3.96E-01
gene32416	75420	NC_000079.6	Secisbp2	3.95E-01
gene41506	240334	NC_000084.6	Pcyox1l	3.94E-01
gene25436	64451	NC_000076.6	Dip2a	3.94E-01
gene15125	56748	NC_000072.6	Nfu1	3.94E-01
gene42964	226182	NC_000085.6	Taf5	3.94E-01
gene41600	114663	NC_000084.6	Impa2	3.94E-01
gene23076	16173	NC_000075.6	Il18	3.94E-01
gene4816	271842	NC_000068.7	Rpusd2	3.94E-01
gene29439	1E+08	NC_000078.6	1110002L01Rik	3.94E-01
gene6567	60505	NC_000069.6	Il21	3.93E-01
gene42175	68852	NC_000085.6	Lrrn4cl	3.93E-01
gene17398	330503	NC_000073.6	Gm5113	3.93E-01
gene18447	20352	NC_000073.6	Sema4b	3.93E-01
gene7817	72522	NC_000069.6	Atxn7l2	3.93E-01
gene18069	108961	NC_000073.6	E2f8	3.93E-01
gene32344	407800	NC_000079.6	Ecm2	3.93E-01
gene19464	30949	NC_000073.6	Lcmt1	3.92E-01
gene27617	73139	NC_000077.6	Cenpv	3.92E-01
gene1841	66875	NC_000067.6	Swt1	3.92E-01
gene20091	664977	NC_000074.6	Gm7434	3.92E-01
gene13056	23948	NC_000071.6	Mmp17	3.92E-01
gene1958	226527	NC_000067.6	BC026585	3.91E-01
gene8986	230126	NC_000070.6	Shb	3.91E-01
gene9329	21885	NC_000070.6	Tle1	3.91E-01

gene34069	26430	NC_000080.6	Parg	3.91E-01
gene27431	216766	NC_000077.6	Gemin5	3.91E-01
gene36325	239528	NC_000081.6	ago-02	3.91E-01
gene11988	73472	NC_000071.6	Spata18	3.91E-01
gene25750	67933	NC_000076.6	Hcfc2	3.90E-01
gene21126	70625	NC_000074.6	Med26	3.90E-01
gene35804	14000	NC_000081.6	Drosha	3.90E-01
gene40605	17685	NC_000083.6	Msh2	3.90E-01
gene10818	242747	NC_000070.6	Zfp933	3.90E-01
gene7907	20321	NC_000069.6	Frrs1	3.90E-01
gene36044	105976	NC_000081.6	AU022793	3.90E-01
gene25875	67019	NC_000076.6	Actr6	3.90E-01
gene20239	1E+08	NC_000074.6	Gm16350	3.89E-01
gene42251	64381	NC_000085.6	Ms4a8a	3.89E-01
gene10247	73680	NC_000070.6	Zbtb8a	3.89E-01
gene22949	382073	NC_000075.6	Ccdc84	3.89E-01
gene38805	26407	NC_000083.6	Map3k4	3.89E-01
gene32487	20184	NC_000079.6	Uimc1	3.89E-01
gene9150	230233	NC_000070.6	Ikbkap	3.88E-01
gene22995	214552	NC_000075.6	Cep164	3.88E-01
gene8234	320229	NC_000069.6	9530052C20Rik	3.88E-01
gene24206	72654	NC_000075.6	Ccdc12	3.87E-01
gene24001	102632	NC_000075.6	Acad11	3.87E-01
gene20080	213027	NC_000074.6	Evi5l	3.87E-01
gene8711	414068	NC_000070.6	Bach2os	3.87E-01
gene4888	27223	NC_000068.7	Trp53bp1	3.87E-01
gene606	74013	NC_000067.6	Rftn2	3.87E-01
gene3407	77996	NC_000068.7	Cutal	3.87E-01
gene35482	74213	NC_000080.6	Rbm26	3.86E-01
gene27937	195018	NC_000077.6	Zzef1	3.86E-01
gene9247	22696	NC_000070.6	Zfp37	3.86E-01
gene747	93691	NC_000067.6	Klf7	3.86E-01
gene24336	104099	NC_000075.6	Itga9	3.86E-01
gene6286	74718	NC_000069.6	Snx16	3.86E-01
gene36000	1.1E+08	NC_000081.6	Gm41304	3.86E-01
gene30401	211978	NC_000078.6	Zfyve26	3.85E-01
gene38032	332175	NC_000082.6	Zdhhc23	3.85E-01
gene28033	1E+08	NC_000077.6	Gm35117	3.85E-01
gene6238	18767	NC_000069.6	Pkia	3.85E-01
gene40096	20230	NC_000083.6	Satb1	3.85E-01
gene21590	12552	NC_000074.6	Cdh11	3.85E-01
gene16279	1E+08	NC_000073.6	Gm15448	3.85E-01
gene35717	78757	NC_000081.6	Rictor	3.84E-01
gene25947	54712	NC_000076.6	Plxnc1	3.84E-01
gene35052	380912	NC_000080.6	Zfp395	3.84E-01
gene9441	230376	NC_000070.6	Haus6	3.83E-01
gene7675	229658	NC_000069.6	Vangl1	3.83E-01
gene19799	212111	NC_000073.6	Inpp5a	3.83E-01

gene6863	329659	NC_000069.6	E130311K13Rik	3.83E-01
gene41816	329003	NC_000084.6	Zfp516	3.83E-01
gene24190	20588	NC_000075.6	Smarcc1	3.83E-01
gene37142	207785	NC_000081.6	Csrnp2	3.83E-01
gene1496	70829	NC_000067.6	Ccdc93	3.83E-01
gene41646	381175	NC_000084.6	Ccdc68	3.82E-01
gene20638	50768	NC_000074.6	Dlc1	3.82E-01
gene11780	70693	NC_000071.6	Adgra3	3.82E-01
gene20147	665306	NC_000074.6	3930402G23Rik	3.82E-01
gene35710	13132	NC_000081.6	Dab2	3.82E-01
gene42747	57753	NC_000085.6	Noc3l	3.82E-01
gene30536	72350	NC_000078.6	Zc2hc1c	3.82E-01
gene411	54610	NC_000067.6	Tbc1d8	3.82E-01
gene15863	56760	NC_000072.6	Clec1b	3.81E-01
gene23643	68291	NC_000075.6	Mto1	3.81E-01
gene21945	78892	NC_000074.6	Crispld2	3.81E-01
gene25787	70428	NC_000076.6	Polr3b	3.81E-01
gene40143	104349	NC_000083.6	Zfp119a	3.81E-01
gene2546	67223	NC_000067.6	Rrp15	3.81E-01
gene4992	215387	NC_000068.7	Ncaph	3.81E-01
gene28367	270906	NC_000077.6	Prr11	3.81E-01
gene37657	107522	NC_000082.6	Ece2	3.80E-01
gene25017	11630	NC_000076.6	Aim1	3.80E-01
gene21207	15245	NC_000074.6	Hhip	3.80E-01
gene9721	230582	NC_000070.6	Cyb5rl	3.80E-01
gene15230	107747	NC_000072.6	Aldh1l1	3.80E-01
gene17473	243914	NC_000073.6	Lgi4	3.80E-01
gene34855	66645	NC_000080.6	Pspc1	3.80E-01
gene12646	192232	NC_000071.6	Hps4	3.80E-01
gene16398	22690	NC_000073.6	Zfp28	3.79E-01
gene5309	228730	NC_000068.7	Kiz	3.79E-01
gene15178	1E+08	NC_000072.6	Gm15612	3.79E-01
gene28883	16399	NC_000077.6	Itga2b	3.79E-01
gene45464	20592	NC_000087.7	Kdm5d	3.79E-01
gene5800	170441	NC_000068.7	Slc2a10	3.78E-01
gene30292	67708	NC_000078.6	Pcnx4	3.78E-01
gene28093	237860	NC_000077.6	Ssh2	3.78E-01
gene32822	218311	NC_000079.6	Zfp455	3.78E-01
gene29217	14027	NC_000077.6	Evpl	3.78E-01
gene33274	23894	NC_000079.6	Gtf2h2	3.78E-01
gene27371	192786	NC_000077.6	Rapgef6	3.78E-01
gene30314	18755	NC_000078.6	Prkch	3.77E-01
gene936	319998	NC_000067.6	Tmem198	3.77E-01
gene43124	74998	NC_000085.6	Rab11fip2	3.77E-01
gene3367	1.1E+08	NC_000068.7	Gm13524	3.77E-01
gene6432	208613	NC_000069.6	Tmem212	3.77E-01
gene7427	229584	NC_000069.6	Pogz	3.77E-01
gene39067	240038	NC_000083.6	Gm4944	3.77E-01

gene20844	108150	NC_000074.6	Galnt7	3.77E-01
gene4828	228543	NC_000068.7	Rhov	3.77E-01
gene20935	72281	NC_000074.6	Sh2d4a	3.76E-01
gene24447	382118	NC_000075.6	Zkscan7	3.76E-01
gene28170	790910	NC_000077.6	Gm11201	3.76E-01
gene25260	30045	NC_000076.6	Dnajc12	3.76E-01
gene17721	668572	NC_000073.6	2610021A01Rik	3.76E-01
gene36687	66570	NC_000081.6	Cenpm	3.76E-01
gene37018	22337	NC_000081.6	Vdr	3.76E-01
gene30833	16625	NC_000078.6	Serpina3c	3.76E-01
gene18735	233545	NC_000073.6	Emsy	3.76E-01
gene18821	207278	NC_000073.6	Fchsd2	3.75E-01
gene35627	105445	NC_000080.6	Dock9	3.75E-01
gene28586	1E+08	NC_000077.6	Gm11532	3.75E-01
gene23172	83961	NC_000075.6	Nrg4	3.75E-01
gene3433	69601	NC_000068.7	Dab2ip	3.75E-01
gene38583	70028	NC_000082.6	Dopey2	3.75E-01
gene32945	69716	NC_000079.6	Trip13	3.75E-01
gene4388	1.1E+08	NC_000068.7	Gm39872	3.75E-01
gene22060	14087	NC_000074.6	Fanca	3.75E-01
gene24741	76306	NC_000076.6	Slc18b1	3.75E-01
gene28124	216971	NC_000077.6	Fam222b	3.75E-01
gene19434	76179	NC_000073.6	Usp31	3.75E-01
gene428	107527	NC_000067.6	Il1rl2	3.74E-01
gene33405	238875	NC_000079.6	Gapt	3.74E-01
gene14543	243382	NC_000072.6	Ppm1k	3.74E-01
gene20480	77733	NC_000074.6	Rnf170	3.74E-01
gene15881	320407	NC_000072.6	Klri2	3.74E-01
gene7903	77559	NC_000069.6	Agl	3.73E-01
gene16057	232449	NC_000072.6	Dera	3.73E-01
gene25932	215008	NC_000076.6	Vezt	3.73E-01
gene5514	20648	NC_000068.7	Snta1	3.73E-01
gene25106	71567	NC_000076.6	Mcm9	3.73E-01
gene32177	67797	NC_000079.6	Snrnp48	3.73E-01
gene17583	233107	NC_000073.6	Kctd15	3.72E-01
gene10279	107581	NC_000070.6	Col16a1	3.72E-01
gene1090	208718	NC_000067.6	Dis3l2	3.72E-01
gene24454	331046	NC_000075.6	Tgm4	3.71E-01
gene25778	77976	NC_000076.6	Nuak1	3.71E-01
gene1347	27392	NC_000067.6	Pign	3.71E-01
gene42696	76073	NC_000085.6	Pcgf5	3.71E-01
gene9319	230316	NC_000070.6	Megf9	3.71E-01
gene34344	78938	NC_000080.6	Fbxo34	3.71E-01
gene22324	17535	NC_000075.6	Mre11a	3.71E-01
gene14894	20019	NC_000072.6	Polr1a	3.71E-01
gene28917	71059	NC_000077.6	Hexim2	3.71E-01
gene39349	224650	NC_000083.6	Anks1	3.70E-01
gene21121	72254	NC_000074.6	1700030K09Rik	3.70E-01

gene1571	226419	NC_000067.6	Dyrk3	3.70E-01
gene24587	22288	NC_000076.6	Utrn	3.70E-01
gene10085	170638	NC_000070.6	Hpcal4	3.69E-01
gene39264	106618	NC_000083.6	Wdr90	3.69E-01
gene7603	15360	NC_000069.6	Hmgcs2	3.69E-01
gene966	74205	NC_000067.6	Acsl3	3.69E-01
gene40867	13511	NC_000084.6	Dsg2	3.69E-01
gene17428	233071	NC_000073.6	Arhgap33	3.68E-01
gene11772	66768	NC_000071.6	Pacrgl	3.68E-01
gene18866	=Gene;ger	NC_000073.6	LOC108167452	3.68E-01
gene37837	13383	NC_000082.6	Dlg1	3.68E-01
gene35922	75880	NC_000081.6	4930592A05Rik	3.68E-01
gene9013	107684	NC_000070.6	Coro2a	3.68E-01
gene27537	74665	NC_000077.6	Lrrc48	3.68E-01
gene19614	1E+08	NC_000073.6	Gm17831	3.68E-01
gene36501	75761	NC_000081.6	Apol7a	3.68E-01
gene18781	108937	NC_000073.6	Rnf169	3.68E-01
gene12367	231506	NC_000071.6	Lin54	3.67E-01
gene29475	238037	NC_000078.6	BC068281	3.67E-01
gene40392	225010	NC_000083.6	Lclat1	3.67E-01
gene38398	=Gene;ger	NC_000082.6	LOC108168297	3.67E-01
gene2246	1.1E+08	NC_000067.6	Gm37065	3.67E-01
gene9262	214106	NC_000070.6	4933430I17Rik	3.66E-01
gene41369	1E+08	NC_000084.6	Gm19466	3.66E-01
gene11886	1E+08	NC_000071.6	Gm32374	3.66E-01
gene9112	246179	NC_000070.6	Fktn	3.66E-01
gene29312	207607	NC_000077.6	Ccdc40	3.66E-01
gene41655	26447	NC_000084.6	Poli	3.66E-01
gene12711	77832	NC_000071.6	Tchp	3.66E-01
gene304	13518	NC_000067.6	Dst	3.66E-01
gene39038	210503	NC_000083.6	Zfp677	3.65E-01
gene18133	15204	NC_000073.6	Herc2	3.65E-01
gene10826	26372	NC_000070.6	Clcn6	3.65E-01
gene29399	1.1E+08	NC_000077.6	Gm11775	3.65E-01
gene13311	231830	NC_000071.6	Micall2	3.65E-01
gene36690	24050	NC_000081.6	sep-03	3.65E-01
gene41736	17344	NC_000084.6	Pias2	3.65E-01
gene39146	30943	NC_000083.6	Prss30	3.65E-01
gene11825	116873	NC_000071.6	Stim2	3.65E-01
gene20459	14182	NC_000074.6	Fgfr1	3.65E-01
gene13103	80909	NC_000071.6	Gatsl2	3.65E-01
gene39221	545192	NC_000083.6	Baiap3	3.64E-01
gene26628	16878	NC_000077.6	Lif	3.64E-01
gene18284	117589	NC_000073.6	Asb7	3.64E-01
gene27861	75604	NC_000077.6	Tm4sf5	3.64E-01
gene27534	216810	NC_000077.6	Tom1l2	3.64E-01
gene6361	18583	NC_000069.6	Pde7a	3.64E-01
gene13675	27273	NC_000072.6	Pdk4	3.64E-01

gene34932	219140	NC_000080.6	Spata13	3.63E-01
gene28393	380718	NC_000077.6	Mks1	3.63E-01
gene12088	231380	NC_000071.6	Uba6	3.63E-01
gene35749	320506	NC_000081.6	Lmbrd2	3.63E-01
gene40919	66648	NC_000084.6	Tpgs2	3.63E-01
gene19684	68277	NC_000073.6	2310057M21Rik	3.63E-01
gene28322	237898	NC_000077.6	Usp32	3.63E-01
gene29882	75040	NC_000078.6	Efcab10	3.62E-01
gene29400	58210	NC_000077.6	Sectm1b	3.62E-01
gene8959	73469	NC_000070.6	Rnf38	3.62E-01
gene26480	66118	NC_000076.6	Sarnp	3.62E-01
gene899	241113	NC_000067.6	Prkag3	3.62E-01
gene2783	98910	NC_000068.7	Usp6nl	3.62E-01
gene1167	14472	NC_000067.6	Gbx2	3.62E-01
gene21732	20650	NC_000074.6	Sntb2	3.62E-01
gene23729	320615	NC_000075.6	Dopey1	3.62E-01
gene41064	67393	NC_000084.6	Cxxc5	3.61E-01
gene42920	30838	NC_000085.6	Fbxw4	3.61E-01
gene12751	75387	NC_000071.6	Sirt4	3.61E-01
gene6169	13628	NC_000068.7	Eef1a2	3.61E-01
gene15197	232223	NC_000072.6	Txnrd3	3.61E-01
gene2745	209645	NC_000068.7	Bend7	3.60E-01
gene10886	269604	NC_000070.6	Gpr157	3.60E-01
gene12512	14020	NC_000071.6	Evi5	3.60E-01
gene18481	18584	NC_000073.6	Pde8a	3.60E-01
gene34971	67153	NC_000080.6	Rnaseh2b	3.60E-01
gene18824	73683	NC_000073.6	Atg16l2	3.60E-01
gene30254	319710	NC_000078.6	Frmd6	3.60E-01
gene40530	=Gene;ger	NC_000083.6	LOC108168342	3.60E-01
gene40484	106794	NC_000083.6	Dhx57	3.60E-01
gene24545	237253	NC_000076.6	Lrp11	3.60E-01
gene4450	320507	NC_000068.7	D930015M05Rik	3.60E-01
gene17255	18223	NC_000073.6	Numbl	3.59E-01
gene32941	72293	NC_000079.6	Nkd2	3.59E-01
gene28370	68729	NC_000077.6	Trim37	3.59E-01
gene10861	69928	NC_000070.6	Apitd1	3.59E-01
gene34426	667034	NC_000080.6	Pnp2	3.59E-01
gene22908	72169	NC_000075.6	Trim29	3.59E-01
gene26589	71523	NC_000077.6	8430429K09Rik	3.59E-01
gene15269	1.1E+08	NC_000072.6	Gm38862	3.59E-01
gene40449	320473	NC_000083.6	Heatr5b	3.58E-01
gene12903	231724	NC_000071.6	Rad9b	3.58E-01
gene40529	116871	NC_000083.6	Mta3	3.58E-01
gene14154	64177	NC_000072.6	Trpv6	3.57E-01
gene31121	1E+08	NC_000078.6	Gm30461	3.57E-01
gene12863	23961	NC_000071.6	Oas1b	3.57E-01
gene28460	103537	NC_000077.6	Mbtd1	3.57E-01
gene33150	11881	NC_000079.6	Arsb	3.57E-01

gene6620	72175	NC_000069.6	Mfsd8	3.57E-01
gene24286	70031	NC_000075.6	Cmtm8	3.56E-01
gene6426	72007	NC_000069.6	Fndc3b	3.56E-01
gene4908	319996	NC_000068.7	Casc4	3.56E-01
gene23599	12160	NC_000075.6	Bmp5	3.56E-01
gene4952	17876	NC_000068.7	Myef2	3.56E-01
gene11632	78890	NC_000071.6	Trmt44	3.56E-01
gene36724	1E+08	NC_000081.6	Gm20324	3.56E-01
gene11436	55982	NC_000071.6	Paxip1	3.56E-01
gene24002	252837	NC_000075.6	Ackr4	3.55E-01
gene28273	276952	NC_000077.6	Rasl10b	3.55E-01
gene26896	74467	NC_000077.6	Pus10	3.55E-01
gene6714	108927	NC_000069.6	Lhfp	3.55E-01
gene6810	329650	NC_000069.6	Med12l	3.55E-01
gene17616	233115	NC_000073.6	Dpy19l3	3.55E-01
gene2919	20163	NC_000068.7	Rsu1	3.55E-01
gene15875	58179	NC_000072.6	Klrc3	3.55E-01
gene35044	210925	NC_000080.6	Ints9	3.55E-01
gene21355	66714	NC_000074.6	4921524J17Rik	3.54E-01
gene35768	1E+08	NC_000081.6	Gm10389	3.54E-01
gene4803	214240	NC_000068.7	Disp2	3.54E-01
gene20151	1E+08	NC_000074.6	Gm32709	3.54E-01
gene41791	50771	NC_000084.6	Atp9b	3.54E-01
gene26903	78200	NC_000077.6	4930538E20Rik	3.54E-01
gene3196	22325	NC_000068.7	Vav2	3.53E-01
gene17408	76686	NC_000073.6	Clip3	3.53E-01
gene16888	14282	NC_000073.6	Fosb	3.53E-01
gene40174	193796	NC_000083.6	Kdm4b	3.53E-01
gene6121	228980	NC_000068.7	Taf4	3.53E-01
gene15023	194388	NC_000072.6	Tet3	3.53E-01
gene37228	110542	NC_000081.6	Amhr2	3.52E-01
gene40705	13831	NC_000084.6	Epc1	3.52E-01
gene36289	22402	NC_000081.6	Wisp1	3.52E-01
gene10019	230700	NC_000070.6	Foxj3	3.52E-01
gene8314	70892	NC_000069.6	Ttll7	3.51E-01
gene10293	1E+08	NC_000070.6	Gm35074	3.51E-01
gene35918	15529	NC_000081.6	Sdc2	3.51E-01
gene36472	16581	NC_000081.6	Kifc2	3.51E-01
gene21792	234725	NC_000074.6	Zfp612	3.51E-01
gene24061	81000	NC_000075.6	Rad54l2	3.51E-01
gene25705	21886	NC_000076.6	Tle2	3.51E-01
gene90	109294	NC_000067.6	Prex2	3.51E-01
gene31116	1E+08	NC_000078.6	Ahnak2	3.50E-01
gene32711	70484	NC_000079.6	Slc35d2	3.50E-01
gene7868	67225	NC_000069.6	Rnpc3	3.50E-01
gene40306	68617	NC_000083.6	Mtcl1	3.50E-01
gene24471	12769	NC_000075.6	Ccr9	3.50E-01
gene38942	15116	NC_000083.6	Has1	3.50E-01

gene29417	108903	NC_000077.6	Tbcd	3.50E-01
gene17603	52428	NC_000073.6	Rhpn2	3.50E-01
gene15146	58229	NC_000072.6	Efcc1	3.49E-01
gene7585	68058	NC_000069.6	Chd1l	3.49E-01
gene26897	19696	NC_000077.6	Rel	3.49E-01
gene11483	231086	NC_000071.6	Hadhb	3.49E-01
gene29198	66528	NC_000077.6	Smim5	3.49E-01
gene30592	211064	NC_000078.6	Alkbh1	3.48E-01
gene38611	13876	NC_000082.6	Erg	3.48E-01
gene26208	103468	NC_000076.6	Nup107	3.48E-01
gene2395	15278	NC_000067.6	Tfb2m	3.48E-01
gene3378	241311	NC_000068.7	Zbtb34	3.48E-01
gene18795	70974	NC_000073.6	Pgm2l1	3.48E-01
gene16906	53609	NC_000073.6	Clasrp	3.48E-01
gene3855	67059	NC_000068.7	Ola1	3.47E-01
gene37586	27883	NC_000082.6	Tango2	3.47E-01
gene31871	75887	NC_000079.6	4930586N03Rik	3.47E-01
gene20955	378466	NC_000074.6	Gm10033	3.47E-01
gene16223	232566	NC_000072.6	Amn1	3.47E-01
gene41411	1E+08	NC_000084.6	Gm34632	3.47E-01
gene2773	326622	NC_000068.7	Upf2	3.46E-01
gene42590	58205	NC_000085.6	Pdcd1lg2	3.46E-01
gene36514	328563	NC_000081.6	Apol11b	3.46E-01
gene20847	1E+08	NC_000074.6	AW046200	3.46E-01
gene11354	231042	NC_000071.6	Nupl2	3.46E-01
gene35458	105689	NC_000080.6	Mycbp2	3.46E-01
gene2038	14262	NC_000067.6	Fmo3	3.46E-01
gene30350	109929	NC_000078.6	Zbtb25	3.46E-01
gene11242	20349	NC_000071.6	Sema3e	3.46E-01
gene26591	237625	NC_000077.6	Pla2g3	3.46E-01
gene25099	18821	NC_000076.6	Pln	3.46E-01
gene40300	106585	NC_000083.6	Ankrd12	3.46E-01
gene17378	22693	NC_000073.6	Zfp30	3.46E-01
gene2646	214791	NC_000067.6	Sertad4	3.45E-01
gene23329	71819	NC_000075.6	Kif23	3.45E-01
gene7586	14263	NC_000069.6	Fmo5	3.45E-01
gene25946	77048	NC_000076.6	Cep83	3.45E-01
gene11404	57434	NC_000071.6	Xrcc2	3.45E-01
gene35178	15460	NC_000080.6	Hr	3.45E-01
gene29308	207592	NC_000077.6	Tbc1d16	3.44E-01
gene4550	27402	NC_000068.7	Pdhx	3.44E-01
gene12542	1.1E+08	NC_000071.6	Gm42151	3.44E-01
gene4469	241556	NC_000068.7	Tspan18	3.44E-01
gene27943	55984	NC_000077.6	Camkk1	3.44E-01
gene6385	1E+08	NC_000069.6	Gm31320	3.44E-01
gene30913	668158	NC_000078.6	Ccdc85c	3.44E-01
gene17587	18624	NC_000073.6	Pepd	3.43E-01
gene32828	1E+08	NC_000079.6	Gm17039	3.43E-01

gene25075	77296	NC_000076.6	Fam162b	3.43E-01
gene4612	99349	NC_000068.7	Dnajc24	3.43E-01
gene28212	1.1E+08	NC_000077.6	Gm38907	3.43E-01
gene1678	1E+08	NC_000067.6	Gm32670	3.43E-01
gene17998	20211	NC_000073.6	Saa4	3.43E-01
gene11889	213081	NC_000071.6	Wdr19	3.43E-01
gene17743	434178	NC_000073.6	Zfp141	3.42E-01
gene690	269198	NC_000067.6	Nbeal1	3.42E-01
gene32052	66482	NC_000079.6	Exoc2	3.42E-01
gene1561	64435	NC_000067.6	Fcamr	3.42E-01
gene16147	16970	NC_000072.6	Lrmp	3.42E-01
gene10732	230895	NC_000070.6	Vps13d	3.41E-01
gene35164	1E+08	NC_000080.6	Gm26908	3.41E-01
gene37434	223978	NC_000082.6	Cpped1	3.41E-01
gene34849	219103	NC_000080.6	Cenpj	3.41E-01
gene18799	277939	NC_000073.6	C2cd3	3.41E-01
gene25850	67282	NC_000076.6	Ccdc53	3.41E-01
gene7938	99887	NC_000069.6	Tmem56	3.41E-01
gene30137	66603	NC_000078.6	Gemin2	3.41E-01
gene32819	268670	NC_000079.6	Zfp759	3.41E-01
gene23332	93683	NC_000075.6	Glce	3.41E-01
gene10926	269608	NC_000070.6	Plekhg5	3.41E-01
gene27021	14172	NC_000077.6	Fgf18	3.41E-01
gene19865	78520	NC_000073.6	C330022C24Rik	3.41E-01
gene22903	235300	NC_000075.6	Tmem136	3.41E-01
gene35733	170762	NC_000081.6	Nup155	3.40E-01
gene7836	76123	NC_000069.6	Gpsm2	3.40E-01
gene17141	232970	NC_000073.6	Phldb3	3.40E-01
gene31635	105298	NC_000079.6	Epdr1	3.40E-01
gene39377	=Gene;ger	NC_000083.6	LOC105246245	3.40E-01
gene9343	242505	NC_000070.6	Rasef	3.40E-01
gene28405	103841	NC_000077.6	Cuedc1	3.40E-01
gene42622	208449	NC_000085.6	Sgms1	3.40E-01
gene26215	17245	NC_000076.6	Mdm1	3.40E-01
gene31570	94246	NC_000079.6	Arid4b	3.40E-01
gene19564	233893	NC_000073.6	Zfp764	3.40E-01
gene27730	1E+08	NC_000077.6	Gm32273	3.40E-01
gene16891	333654	NC_000073.6	Ppp1r13l	3.40E-01
gene15737	14433	NC_000072.6	Gapdh	3.40E-01
gene9883	68075	NC_000070.6	Lurap1	3.39E-01
gene4930	269346	NC_000068.7	Slc28a2	3.39E-01
gene25186	22295	NC_000076.6	Cdh23	3.39E-01
gene42792	17060	NC_000085.6	Blnk	3.39E-01
gene15593	94047	NC_000072.6	Cecr6	3.39E-01
gene1528	226409	NC_000067.6	Zranb3	3.38E-01
gene7039	213582	NC_000069.6	Map9	3.38E-01
gene10452	1E+08	NC_000070.6	Gm30822	3.38E-01
gene40493	619329	NC_000083.6	F420015M19Rik	3.38E-01

gene8750	109093	NC_000070.6	Rars2	3.38E-01
gene27516	1E+08	NC_000077.6	Gm33998	3.38E-01
gene13566	1E+08	NC_000071.6	Gm36141	3.37E-01
gene34358	218989	NC_000080.6	Tmem260	3.37E-01
gene30455	338372	NC_000078.6	Map3k9	3.37E-01
gene354	94220	NC_000067.6	Cnnm4	3.37E-01
gene5581	228829	NC_000068.7	Phf20	3.37E-01
gene29029	71795	NC_000077.6	Pitpnc1	3.37E-01
gene41398	107022	NC_000084.6	Gramd3	3.37E-01
gene20203	66790	NC_000074.6	Grtp1	3.37E-01
gene19662	11907	NC_000073.6	Ate1	3.37E-01
gene18709	233532	NC_000073.6	Rsf1	3.37E-01
gene7198	76022	NC_000069.6	Gon4l	3.37E-01
gene32182	1E+08	NC_000079.6	Gm31834	3.36E-01
gene3578	11480	NC_000068.7	Acvr2a	3.36E-01
gene27851	11685	NC_000077.6	Alox12e	3.36E-01
gene22221	244672	NC_000075.6	Cwf19l2	3.36E-01
gene26089	1.1E+08	NC_000076.6	Gm40755	3.36E-01
gene23293	1E+08	NC_000075.6	Gm20275	3.36E-01
gene17369	74206	NC_000073.6	Sipa1l3	3.36E-01
gene31409	217946	NC_000078.6	Cdca7l	3.36E-01
gene356	78321	NC_000067.6	Ankrd23	3.36E-01
gene35039	16554	NC_000080.6	Kif13b	3.36E-01
gene27719	104709	NC_000077.6	Pik3r6	3.36E-01
gene41744	212163	NC_000084.6	8030462N17Rik	3.35E-01
gene2909	320816	NC_000068.7	Ankrd16	3.35E-01
gene35722	16880	NC_000081.6	Lifr	3.35E-01
gene37933	268885	NC_000082.6	Stfa2l1	3.35E-01
gene3387	72543	NC_000068.7	Mvb12b	3.35E-01
gene24365	74052	NC_000075.6	Ttc21a	3.35E-01
gene12395	1.1E+08	NC_000071.6	Gm42135	3.35E-01
gene31397	76044	NC_000078.6	Ncapg2	3.35E-01
gene20470	20845	NC_000074.6	Star	3.34E-01
gene32967	235956	NC_000079.6	Zfp825	3.34E-01
gene7010	329679	NC_000069.6	Fnip2	3.34E-01
gene629	68736	NC_000067.6	Tyw5	3.34E-01
gene5229	69487	NC_000068.7	Ndufaf5	3.34E-01
gene39201	16005	NC_000083.6	Igfals	3.33E-01
gene1859	19731	NC_000067.6	Rgl1	3.33E-01
gene29137	286942	NC_000077.6	Kif19a	3.33E-01
gene26894	71675	NC_000077.6	0610010F05Rik	3.33E-01
gene844	54380	NC_000067.6	Smarcal1	3.32E-01
gene9263	50780	NC_000070.6	Rgs3	3.32E-01
gene17135	232969	NC_000073.6	Zfp428	3.32E-01
gene10373	53608	NC_000070.6	Map3k6	3.32E-01
gene18748	78610	NC_000073.6	Uvrag	3.32E-01
gene10606	1E+08	NC_000070.6	Gm13031	3.32E-01
gene12153	52822	NC_000071.6	Rufy3	3.32E-01

gene17382	233058	NC_000073.6	Zfp420	3.32E-01
gene1630	1E+08	NC_000067.6	Gm19461	3.32E-01
gene18252	330554	NC_000073.6	Fan1	3.32E-01
gene30358	263406	NC_000078.6	Plekhg3	3.32E-01
gene20898	77113	NC_000074.6	Klhl2	3.32E-01
gene36983	77044	NC_000081.6	Arid2	3.32E-01
gene38716	21648	NC_000083.6	Dynlt1b	3.32E-01
gene25940	319880	NC_000076.6	Tmcc3	3.31E-01
gene30302	20474	NC_000078.6	Six4	3.31E-01
gene41883	20589	NC_000085.6	Ighmbp2	3.31E-01
gene17786	210104	NC_000073.6	Zfp658	3.31E-01
gene43148	14773	NC_000085.6	Grk5	3.31E-01
gene11805	211006	NC_000071.6	Sepsecs	3.31E-01
gene1124	227334	NC_000067.6	Usp40	3.31E-01
gene18490	1E+08	NC_000073.6	2900076A07Rik	3.31E-01
gene24907	16775	NC_000076.6	Lama4	3.31E-01
gene28336	69909	NC_000077.6	2610027K06Rik	3.30E-01
gene2244	27218	NC_000067.6	Slamf1	3.30E-01
gene35672	1E+08	NC_000081.6	Ccdc152	3.30E-01
gene5102	228602	NC_000068.7	4930402H24Rik	3.30E-01
gene436	1E+08	NC_000067.6	Gm37915	3.30E-01
gene23204	66069	NC_000075.6	Snupn	3.30E-01
gene37774	1E+08	NC_000082.6	Gm20319	3.30E-01
gene15234	232227	NC_000072.6	Iqsec1	3.30E-01
gene23691	1E+08	NC_000075.6	Gm2065	3.29E-01
gene37034	239652	NC_000081.6	Zfp641	3.29E-01
gene24263	76499	NC_000075.6	Clasp2	3.29E-01
gene12582	69504	NC_000071.6	Zfp932	3.29E-01
gene31034	1E+08	NC_000078.6	Gm35558	3.29E-01
gene5485	72326	NC_000068.7	2500004C02Rik	3.29E-01
gene26066	327812	NC_000076.6	Gm15663	3.29E-01
gene30053	112407	NC_000078.6	Egln3	3.29E-01
gene27665	216831	NC_000077.6	Arhgap44	3.29E-01
gene220	280645	NC_000067.6	B3gat2	3.29E-01
gene145	17087	NC_000067.6	Ly96	3.29E-01
gene23932	74125	NC_000075.6	Armc8	3.28E-01
gene32235	67046	NC_000079.6	Tbc1d7	3.28E-01
gene10244	68828	NC_000070.6	Sync	3.28E-01
gene25234	1E+08	NC_000076.6	Gm30322	3.28E-01
gene25794	74238	NC_000076.6	Mterf2	3.28E-01
gene27011	327900	NC_000077.6	Ubtd2	3.28E-01
gene19099	233651	NC_000073.6	Dchs1	3.28E-01
gene37930	381045	NC_000082.6	Ccdc58	3.28E-01
gene21638	234673	NC_000074.6	Ces2e	3.28E-01
gene13178	23921	NC_000071.6	Sh2b2	3.28E-01
gene17919	50722	NC_000073.6	Dkkl1	3.28E-01
gene16841	666831	NC_000073.6	Gm8314	3.28E-01
gene29135	117160	NC_000077.6	Ttyh2	3.28E-01

gene23437	13143	NC_000075.6	Dapk2	3.27E-01
gene3443	67871	NC_000068.7	Mrrf	3.27E-01
gene1856	66637	NC_000067.6	Tsen15	3.27E-01
gene8290	214804	NC_000069.6	Syde2	3.27E-01
gene3144	67839	NC_000068.7	Gpsm1	3.27E-01
gene32592	67269	NC_000079.6	Agtpbp1	3.27E-01
gene26943	108686	NC_000077.6	Ccdc88a	3.26E-01
gene1988	29809	NC_000067.6	Rabgap1l	3.26E-01
gene36741	69291	NC_000081.6	1700001L05Rik	3.26E-01
gene20157	71941	NC_000074.6	Cars2	3.26E-01
gene21896	66531	NC_000074.6	Cmc2	3.26E-01
gene39159	12449	NC_000083.6	Ccnf	3.26E-01
gene28262	20558	NC_000077.6	Slfn4	3.26E-01
gene9393	18028	NC_000070.6	Nfib	3.26E-01
gene32341	75678	NC_000079.6	Ippk	3.25E-01
gene5591	1E+08	NC_000068.7	Gm33977	3.25E-01
gene23678	319405	NC_000075.6	D430036J16Rik	3.25E-01
gene23504	93836	NC_000075.6	Rnf111	3.25E-01
gene25158	103080	NC_000076.6	sep-10	3.25E-01
gene8255	=Gene;ger	NC_000069.6	LOC108168905	3.24E-01
gene4560	98956	NC_000068.7	Nat10	3.24E-01
gene23685	65099	NC_000075.6	Irak1bp1	3.24E-01
gene11129	207704	NC_000071.6	Gtpbp10	3.24E-01
gene7227	13639	NC_000069.6	Efna4	3.24E-01
gene40488	20662	NC_000083.6	Sos1	3.23E-01
gene13180	13047	NC_000071.6	Cux1	3.23E-01
gene41750	19201	NC_000084.6	Pstpip2	3.23E-01
gene23928	17532	NC_000075.6	Mras	3.23E-01
gene38717	547127	NC_000083.6	Tmem181b-ps	3.23E-01
gene692	329165	NC_000067.6	Abi2	3.23E-01
gene29380	77583	NC_000077.6	Notum	3.23E-01
gene30879	76559	NC_000078.6	Atg2b	3.23E-01
gene13274	330217	NC_000071.6	Gal3st4	3.23E-01
gene37069	13363	NC_000081.6	Dhh	3.23E-01
gene34496	50524	NC_000080.6	Sall2	3.22E-01
gene16423	76373	NC_000073.6	Zfp773	3.22E-01
gene36722	21411	NC_000081.6	Tcf20	3.22E-01
gene24958	103199	NC_000076.6	Fig4	3.22E-01
gene12885	338350	NC_000071.6	Acad12	3.22E-01
gene14596	70945	NC_000072.6	Mmrn1	3.22E-01
gene39516	22643	NC_000083.6	Zfp101	3.21E-01
gene15402	16438	NC_000072.6	Itpr1	3.21E-01
gene9597	94043	NC_000070.6	Tm2d1	3.21E-01
gene39788	54393	NC_000083.6	Gabbr1	3.21E-01
gene34022	78467	NC_000080.6	1700087M22Rik	3.21E-01
gene36470	72749	NC_000081.6	Tonsl	3.21E-01
gene993	210293	NC_000067.6	Dock10	3.20E-01
gene35355	219228	NC_000080.6	Pcdh17	3.20E-01

gene21938	1E+08	NC_000074.6	Gm32352	3.20E-01
gene15555	70727	NC_000072.6	Rasgef1a	3.20E-01
gene23825	26432	NC_000075.6	Plod2	3.20E-01
gene9731	72787	NC_000070.6	Ndc1	3.20E-01
gene37688	74147	NC_000082.6	Ehhadh	3.20E-01
gene41808	329002	NC_000084.6	Zfp236	3.19E-01
gene15054	232174	NC_000072.6	Cyp26b1	3.19E-01
gene38803	68262	NC_000083.6	Agpat4	3.19E-01
gene28328	1.1E+08	NC_000077.6	Gm38935	3.19E-01
gene2913	241226	NC_000068.7	Itga8	3.19E-01
gene10479	21401	NC_000070.6	Tcea3	3.19E-01
gene13306	76854	NC_000071.6	Gper1	3.19E-01
gene6125	269397	NC_000068.7	Ss18l1	3.19E-01
gene39209	57354	NC_000083.6	Cramp1l	3.19E-01
gene25937	66414	NC_000076.6	Ndufa12	3.18E-01
gene25309	71371	NC_000076.6	Arid5b	3.18E-01
gene14245	14056	NC_000072.6	Ezh2	3.18E-01
gene27567	21975	NC_000077.6	Top3a	3.18E-01
gene38622	93871	NC_000082.6	Brwd1	3.18E-01
gene36872	71474	NC_000081.6	Ppp6r2	3.18E-01
gene10267	230770	NC_000070.6	Tmem39b	3.18E-01
gene30473	73828	NC_000078.6	Dcaf4	3.18E-01
gene5049	20017	NC_000068.7	Polr1b	3.18E-01
gene12942	77045	NC_000071.6	Bcl7a	3.18E-01
gene29085	217265	NC_000077.6	Abca5	3.18E-01
gene19997	79201	NC_000073.6	Tnfrsf23	3.17E-01
gene13293	19085	NC_000071.6	Prkar1b	3.17E-01
gene11471	23950	NC_000071.6	Dnajb6	3.17E-01
gene29460	17977	NC_000078.6	Ncoa1	3.17E-01
gene22628	14247	NC_000075.6	Fli1	3.17E-01
gene31038	69641	NC_000078.6	Wdr20	3.17E-01
gene23739	266690	NC_000075.6	Cyb5r4	3.17E-01
gene37551	18440	NC_000082.6	P2rx6	3.17E-01
gene39478	320997	NC_000083.6	Cyp4f39	3.16E-01
gene20102	20419	NC_000074.6	Shcbp1	3.16E-01
gene3776	241447	NC_000068.7	Cers6	3.16E-01
gene759	227210	NC_000067.6	Ccnyl1	3.16E-01
gene35463	105439	NC_000080.6	Slain1	3.16E-01
gene22220	67667	NC_000075.6	Alkbh8	3.16E-01
gene36816	29870	NC_000081.6	Gtse1	3.16E-01
gene38397	23794	NC_000082.6	Adamts5	3.16E-01
gene33809	1E+08	NC_000080.6	Gm32587	3.16E-01
gene3648	18227	NC_000068.7	Nr4a2	3.15E-01
gene39045	381066	NC_000083.6	Zfp948	3.15E-01
gene20971	53978	NC_000074.6	Lpar2	3.15E-01
gene28209	338367	NC_000077.6	Myo1d	3.15E-01
gene21501	71805	NC_000074.6	Nup93	3.15E-01
gene37639	208146	NC_000082.6	Yeats2	3.15E-01

gene4417	228356	NC_000068.7	1110051M20Rik	3.15E-01
gene17794	626834	NC_000073.6	Klk13	3.15E-01
gene2720	269233	NC_000068.7	Fam171a1	3.15E-01
gene20401	20516	NC_000074.6	Slc20a2	3.14E-01
gene27292	77371	NC_000077.6	Sec24a	3.14E-01
gene20923	234344	NC_000074.6	Naf1	3.14E-01
gene41310	69456	NC_000084.6	Commd10	3.14E-01
gene40176	19280	NC_000083.6	Ptprs	3.14E-01
gene3117	14133	NC_000068.7	Fcna	3.14E-01
gene19700	77590	NC_000073.6	Chst15	3.14E-01
gene40914	225283	NC_000084.6	Rprd1a	3.14E-01
gene25830	103466	NC_000076.6	Nt5dc3	3.14E-01
gene7120	11801	NC_000069.6	Cd5l	3.14E-01
gene15646	20527	NC_000072.6	Slc2a3	3.14E-01
gene41846	1.1E+08	NC_000084.6	Gm16146	3.13E-01
gene33281	76041	NC_000079.6	Ccdc125	3.13E-01
gene3815	73649	NC_000068.7	Cybrd1	3.13E-01
gene12933	100675	NC_000071.6	AI480526	3.13E-01
gene13586	100710	NC_000071.6	Pds5b	3.13E-01
gene11635	78558	NC_000071.6	Htra3	3.13E-01
gene9772	70533	NC_000070.6	Btf3l4	3.13E-01
gene23693	94353	NC_000075.6	Hmgn3	3.13E-01
gene7199	1.1E+08	NC_000069.6	Gm40091	3.12E-01
gene7994	99526	NC_000069.6	Usp53	3.12E-01
gene42257	75138	NC_000085.6	4930526L06Rik	3.12E-01
gene21689	70575	NC_000074.6	Gfod2	3.12E-01
gene6270	94212	NC_000069.6	Pag1	3.12E-01
gene30755	68339	NC_000078.6	Ccdc88c	3.12E-01
gene19894	70552	NC_000073.6	Lrrc56	3.12E-01
gene14486	69938	NC_000072.6	Scrn1	3.12E-01
gene36430	223650	NC_000081.6	Eppk1	3.12E-01
gene9808	67946	NC_000070.6	Spata6	3.11E-01
gene10816	1E+08	NC_000070.6	Gm13157	3.11E-01
gene12662	243219	NC_000071.6	2900026A02Rik	3.11E-01
gene38351	1E+08	NC_000082.6	E330011O21Rik	3.11E-01
gene2728	64707	NC_000068.7	Suv39h2	3.11E-01
gene4546	20511	NC_000068.7	Slc1a2	3.11E-01
gene22114	19341	NC_000074.6	Rab4a	3.10E-01
gene14059	109880	NC_000072.6	Braf	3.10E-01
gene14488	231999	NC_000072.6	Plekha8	3.10E-01
gene4846	17005	NC_000068.7	Ltk	3.10E-01
gene13343	1E+08	NC_000071.6	Gm30804	3.10E-01
gene35149	21933	NC_000080.6	Tnfrsf10b	3.10E-01
gene11018	242805	NC_000070.6	Ankrd65	3.09E-01
gene10194	67785	NC_000070.6	Zmym4	3.09E-01
gene21493	67378	NC_000074.6	Bbs2	3.09E-01
gene6695	211666	NC_000069.6	Mgst2	3.09E-01
gene8557	329795	NC_000070.6	Tmem67	3.09E-01

gene4903	67532	NC_000068.7	Mfap1a	3.08E-01
gene40379	1E+08	NC_000083.6	Gm30140	3.08E-01
gene11452	381626	NC_000071.6	Rbm33	3.08E-01
gene11993	=Gene;ger	NC_000071.6	LOC108169050	3.08E-01
gene1577	14270	NC_000067.6	Srgap2	3.08E-01
gene27041	76874	NC_000077.6	4930469K13Rik	3.08E-01
gene24029	235582	NC_000075.6	Glyctk	3.08E-01
gene41253	22240	NC_000084.6	Dpysl3	3.08E-01
gene24935	72472	NC_000076.6	Slc16a10	3.08E-01
gene42749	15201	NC_000085.6	Hells	3.08E-01
gene31490	1E+08	NC_000079.6	Gm35730	3.08E-01
gene26712	56089	NC_000077.6	Ramp3	3.07E-01
gene35798	68070	NC_000081.6	Pdzd2	3.07E-01
gene5609	66934	NC_000068.7	Dsn1	3.07E-01
gene7910	74654	NC_000069.6	4930455H04Rik	3.07E-01
gene2798	14462	NC_000068.7	Gata3	3.07E-01
gene19719	13017	NC_000073.6	Ctbp2	3.07E-01
gene10869	66454	NC_000070.6	Nmnat1	3.06E-01
gene36604	1E+08	NC_000081.6	Gm16576	3.06E-01
gene4874	140810	NC_000068.7	Ttbk2	3.06E-01
gene17597	53896	NC_000073.6	Slc7a10	3.06E-01
gene29754	12879	NC_000078.6	Cys1	3.06E-01
gene38701	20975	NC_000083.6	Synj2	3.06E-01
gene35950	1.1E+08	NC_000081.6	Gm41292	3.06E-01
gene10206	14620	NC_000070.6	Gjb3	3.05E-01
gene21346	244556	NC_000074.6	Zfp791	3.05E-01
gene418	425051	NC_000067.6	D930019O06Rik	3.05E-01
gene39145	1.1E+08	NC_000083.6	Gm41546	3.05E-01
gene38925	626854	NC_000083.6	Gm38396	3.05E-01
gene7986	=Gene;ger	NC_000069.6	LOC108168896	3.05E-01
gene32520	72562	NC_000079.6	Pcbd2	3.05E-01
gene28588	69297	NC_000077.6	Lrrc46	3.05E-01
gene9932	140546	NC_000070.6	Eri3	3.05E-01
gene11859	76261	NC_000071.6	0610040J01Rik	3.05E-01
gene9890	17776	NC_000070.6	Mast2	3.05E-01
gene33330	75805	NC_000079.6	Nln	3.04E-01
gene26104	320150	NC_000076.6	Zdhhc17	3.04E-01
gene22991	59095	NC_000075.6	Fxyd6	3.04E-01
gene40245	75039	NC_000083.6	Nudt12os	3.04E-01
gene11960	100978	NC_000071.6	Nfxl1	3.04E-01
gene9923	73804	NC_000070.6	Kif2c	3.04E-01
gene6327	320832	NC_000069.6	Sirpb1a	3.04E-01
gene898	104086	NC_000067.6	Cyp27a1	3.03E-01
gene24938	67239	NC_000076.6	Rpf2	3.03E-01
gene22338	319675	NC_000075.6	Cep295	3.03E-01
gene29150	382551	NC_000077.6	Cd300ld3	3.03E-01
gene32741	170936	NC_000079.6	Zfp369	3.03E-01
gene6679	12457	NC_000069.6	Noct	3.03E-01

gene2922	13434	NC_000068.7	Trdmt1	3.03E-01
gene40871	56386	NC_000084.6	B4galt6	3.03E-01
gene595	329154	NC_000067.6	Ankrd44	3.03E-01
gene39362	14229	NC_000083.6	Fkbp5	3.03E-01
gene23441	235439	NC_000075.6	Herc1	3.03E-01
gene7121	229499	NC_000069.6	Fcrl1	3.02E-01
gene36612	52609	NC_000081.6	Cbx7	3.02E-01
gene15853	232408	NC_000072.6	Klrb1f	3.02E-01
gene2598	68972	NC_000067.6	Tatdn3	3.02E-01
gene42620	54447	NC_000085.6	Asah2	3.02E-01
gene15984	=Gene;ger	NC_000072.6	LOC102639979	3.02E-01
gene20315	1E+08	NC_000074.6	Gm21092	3.02E-01
gene19008	94094	NC_000073.6	Trim34a	3.02E-01
gene23547	319758	NC_000075.6	Rfx7	3.02E-01
gene16205	387314	NC_000072.6	Tmtc1	3.02E-01
gene36327	14083	NC_000081.6	Ptk2	3.01E-01
gene22512	319601	NC_000075.6	Zfp653	3.01E-01
gene19988	18400	NC_000073.6	Slc22a18	3.01E-01
gene23278	102774	NC_000075.6	Bbs4	3.01E-01
gene6917	17349	NC_000069.6	Mlf1	3.01E-01
gene25862	20728	NC_000076.6	Spic	3.01E-01
gene4499	74569	NC_000068.7	Ttc17	3.01E-01
gene5101	269356	NC_000068.7	Slc4a11	3.01E-01
gene39157	18209	NC_000083.6	Ntn3	3.00E-01
gene12023	1E+08	NC_000071.6	Gm33938	3.00E-01
gene17718	101835	NC_000073.6	AW146154	3.00E-01
gene27530	19377	NC_000077.6	Rai1	3.00E-01
gene42862	66388	NC_000085.6	Cutc	3.00E-01
gene2500	63953	NC_000067.6	Dusp10	3.00E-01
gene3733	50915	NC_000068.7	Grb14	3.00E-01
gene35238	380916	NC_000080.6	Lrch1	3.00E-01
gene2636	214742	NC_000067.6	Rcor3	2.99E-01
gene24801	73910	NC_000076.6	Arhgap18	2.99E-01
gene32997	218343	NC_000079.6	Ttc37	2.99E-01
gene24603	66253	NC_000076.6	Aig1	2.99E-01
gene8639	77593	NC_000070.6	Usp45	2.98E-01
gene27625	192976	NC_000077.6	Lrrc75a	2.98E-01
gene21561	319960	NC_000074.6	4930513N10Rik	2.98E-01
gene33118	13361	NC_000079.6	Dhfr	2.98E-01
gene18699	14389	NC_000073.6	Gab2	2.98E-01
gene10878	70556	NC_000070.6	Slc25a33	2.98E-01
gene11476	16193	NC_000071.6	Il6	2.98E-01
gene22000	53325	NC_000074.6	Banp	2.98E-01
gene35462	64929	NC_000080.6	Scel	2.97E-01
gene32286	16553	NC_000079.6	Kif13a	2.97E-01
gene32463	12890	NC_000079.6	Cplx2	2.97E-01
gene12840	74111	NC_000071.6	Rbm19	2.97E-01
gene12296	94061	NC_000071.6	Mrpl1	2.97E-01

gene3010	71233	NC_000068.7	Enkur	2.97E-01
gene37715	70573	NC_000082.6	Tbccd1	2.96E-01
gene12087	56792	NC_000071.6	Stap1	2.96E-01
gene7128	213498	NC_000069.6	Arhgef11	2.96E-01
gene24343	22351	NC_000075.6	Vill	2.96E-01
gene7457	71790	NC_000069.6	Anxa9	2.96E-01
gene36645	223701	NC_000081.6	Mkl1	2.96E-01
gene7097	320302	NC_000069.6	Glt28d2	2.96E-01
gene40406	76890	NC_000083.6	Memo1	2.96E-01
gene22433	244721	NC_000075.6	Zfp846	2.96E-01
gene12018	1E+08	NC_000071.6	Gm32727	2.96E-01
gene37280	18574	NC_000081.6	Pde1b	2.96E-01
gene27480	69944	NC_000077.6	2810021J22Rik	2.96E-01
gene11338	433855	NC_000071.6	AI506816	2.96E-01
gene29877	68764	NC_000078.6	Cdhr3	2.96E-01
gene36638	213956	NC_000081.6	Fam83f	2.96E-01
gene21220	14388	NC_000074.6	Gab1	2.96E-01
gene13190	66752	NC_000071.6	4933404O12Rik	2.95E-01
gene5407	228765	NC_000068.7	Sdcbp2	2.95E-01
gene25849	69736	NC_000076.6	Nup37	2.95E-01
gene8725	242377	NC_000070.6	Pm20d2	2.95E-01
gene8642	230027	NC_000070.6	Coq3	2.95E-01
gene14027	320538	NC_000072.6	Ubn2	2.95E-01
gene11682	71116	NC_000071.6	Stx18	2.95E-01
gene25936	22025	NC_000076.6	Nr2c1	2.94E-01
gene41384	269023	NC_000084.6	Zfp608	2.94E-01
gene42823	226123	NC_000085.6	Morn4	2.94E-01
gene1925	72482	NC_000067.6	Acbd6	2.94E-01
gene28556	15414	NC_000077.6	Hoxb6	2.94E-01
gene40414	268973	NC_000083.6	Nlrc4	2.94E-01
gene9925	68777	NC_000070.6	Tmem53	2.94E-01
gene34903	219131	NC_000080.6	Phf11a	2.94E-01
gene36466	268822	NC_000081.6	Adck5	2.94E-01
gene28062	68299	NC_000077.6	Vps53	2.94E-01
gene5747	1E+08	NC_000068.7	Gm36090	2.94E-01
gene12001	16924	NC_000071.6	Lnx1	2.93E-01
gene27432	1E+08	NC_000077.6	Gm33350	2.93E-01
gene2144	66977	NC_000067.6	Nuf2	2.93E-01
gene15289	16206	NC_000072.6	Lrig1	2.93E-01
gene3868	329427	NC_000068.7	Chn1os3	2.93E-01
gene13869	56463	NC_000072.6	Snd1	2.93E-01
gene7153	404710	NC_000069.6	Iqgap3	2.93E-01
gene36971	791370	NC_000081.6	A130051J06Rik	2.93E-01
gene28142	54141	NC_000077.6	Spag5	2.93E-01
gene2577	19250	NC_000067.6	Ptpn14	2.93E-01
gene20323	1E+08	NC_000074.6	Gm20796	2.93E-01
gene27299	1.1E+08	NC_000077.6	Gm39822	2.93E-01
gene3880	69605	NC_000068.7	Lnp	2.93E-01

gene41653	269033	NC_000084.6	4930503L19Rik	2.93E-01
gene17134	260299	NC_000073.6	Cadm4	2.93E-01
gene15243	22421	NC_000072.6	Wnt7a	2.93E-01
gene20632	244421	NC_000074.6	Lonrf1	2.92E-01
gene29269	217351	NC_000077.6	Tnrc6c	2.92E-01
gene28481	1E+08	NC_000077.6	Gm35650	2.92E-01
gene41771	93737	NC_000084.6	Pard6g	2.92E-01
gene11619	54426	NC_000071.6	Hgfac	2.92E-01
gene5770	67538	NC_000068.7	Zswim3	2.92E-01
gene28681	23834	NC_000077.6	Cdc6	2.92E-01
gene42963	71041	NC_000085.6	Pcgf6	2.92E-01
gene40005	210982	NC_000083.6	Gltscr1l	2.92E-01
gene19102	77700	NC_000073.6	9130208D14Rik	2.91E-01
gene26453	216459	NC_000076.6	Myl6b	2.91E-01
gene13790	12638	NC_000072.6	Cftr	2.91E-01
gene39094	22700	NC_000083.6	Zfp40	2.91E-01
gene30178	217648	NC_000078.6	Gm527	2.91E-01
gene16425	232854	NC_000073.6	Zfp418	2.91E-01
gene15876	16642	NC_000072.6	Klrc2	2.91E-01
gene29881	75836	NC_000078.6	Atxn7l1os2	2.91E-01
gene19442	70316	NC_000073.6	Ndufab1	2.91E-01
gene25027	12142	NC_000076.6	Prdm1	2.90E-01
gene32294	218215	NC_000079.6	Rnf144b	2.90E-01
gene1001	=Gene;ger	NC_000067.6	LOC108167630	2.90E-01
gene11740	19126	NC_000071.6	Prom1	2.90E-01
gene9768	18392	NC_000070.6	Orc1	2.90E-01
gene27905	216881	NC_000077.6	Wscd1	2.89E-01
gene14191	232748	NC_000072.6	Tcaf2	2.89E-01
gene7584	1.1E+08	NC_000069.6	Gm40102	2.89E-01
gene36745	319953	NC_000081.6	Ttll1	2.89E-01
gene29911	66912	NC_000078.6	Bzw2	2.89E-01
gene6140	1E+08	NC_000068.7	Gata5os	2.88E-01
gene2576	108000	NC_000067.6	Cenpf	2.88E-01
gene24598	215789	NC_000076.6	Phactr2	2.88E-01
gene4870	668880	NC_000068.7	Stard9	2.88E-01
gene23190	244895	NC_000075.6	Peak1	2.88E-01
gene25965	1E+08	NC_000076.6	Gm33377	2.87E-01
gene5590	13821	NC_000068.7	Epb41l1	2.87E-01
gene41930	107239	NC_000085.6	Carns1	2.87E-01
gene25464	110532	NC_000076.6	Adarb1	2.87E-01
gene32280	666794	NC_000079.6	Rbm24	2.87E-01
gene35097	68813	NC_000080.6	Dock5	2.86E-01
gene14038	21391	NC_000072.6	Tbxas1	2.86E-01
gene18465	70461	NC_000073.6	Crtc3	2.86E-01
gene30799	1E+08	NC_000078.6	Gm15523	2.86E-01
gene36138	18383	NC_000081.6	Tnfrsf11b	2.86E-01
gene30431	217682	NC_000078.6	Plekhd1	2.86E-01
gene21129	=Gene;ger	NC_000074.6	LOC108167536	2.86E-01

gene13059	22648	NC_000071.6	Zfp11	2.85E-01
gene30916	68519	NC_000078.6	Eml1	2.85E-01
gene40332	654806	NC_000083.6	A930029G22Rik	2.85E-01
gene21899	69528	NC_000074.6	1700030J22Rik	2.85E-01
gene24444	382117	NC_000075.6	Tcaim	2.85E-01
gene42839	52013	NC_000085.6	R3hcc1l	2.85E-01
gene9577	13110	NC_000070.6	Cyp2j6	2.85E-01
gene6938	68259	NC_000069.6	Ift80	2.85E-01
gene24468	102680	NC_000075.6	Slc6a20a	2.85E-01
gene21148	75141	NC_000074.6	Rasd2	2.85E-01
gene30391	211945	NC_000078.6	Plekhh1	2.85E-01
gene38922	213811	NC_000083.6	BC002059	2.85E-01
gene7978	29815	NC_000069.6	Bcar3	2.85E-01
gene27481	22698	NC_000077.6	Zfp39	2.84E-01
gene16865	1E+08	NC_000073.6	Mypopos	2.84E-01
gene4968	69976	NC_000068.7	Galk2	2.84E-01
gene29069	=Gene;ger	NC_000077.6	LOC108167935	2.84E-01
gene21548	244608	NC_000074.6	Ccdc113	2.84E-01
gene10574	212632	NC_000070.6	Iffo2	2.84E-01
gene22034	257633	NC_000074.6	Acsf3	2.84E-01
gene33135	100715	NC_000079.6	Papd4	2.84E-01
gene18708	66273	NC_000073.6	Aamdc	2.84E-01
gene12696	100705	NC_000071.6	Acacb	2.83E-01
gene3785	72569	NC_000068.7	Bbs5	2.83E-01
gene960	433323	NC_000067.6	Sgpp2	2.83E-01
gene21533	330830	NC_000074.6	Drc7	2.83E-01
gene13356	231858	NC_000071.6	Radil	2.83E-01
gene34877	239114	NC_000080.6	Il17d	2.83E-01
gene4585	228410	NC_000068.7	Cstf3	2.83E-01
gene9719	72475	NC_000070.6	Ssbp3	2.83E-01
gene10035	29871	NC_000070.6	Scmh1	2.83E-01
gene37962	67657	NC_000082.6	Rabl3	2.83E-01
gene12377	231510	NC_000071.6	Agpat9	2.83E-01
gene15615	14411	NC_000072.6	Slc6a12	2.82E-01
gene38585	1E+08	NC_000082.6	Gm30881	2.82E-01
gene16336	269855	NC_000073.6	Ssc5d	2.82E-01
gene30886	22367	NC_000078.6	Vrk1	2.82E-01
gene7652	630146	NC_000069.6	Cd101	2.82E-01
gene16054	13860	NC_000072.6	Eps8	2.82E-01
gene40702	1.1E+08	NC_000084.6	Gm41664	2.82E-01
gene2544	21808	NC_000067.6	Tgfb2	2.81E-01
gene39069	319615	NC_000083.6	Zfp944	2.81E-01
gene25866	70683	NC_000076.6	Utp20	2.81E-01
gene20734	53318	NC_000074.6	Pdlim3	2.81E-01
gene725	227195	NC_000067.6	Ino80d	2.81E-01
gene20196	66901	NC_000074.6	Proz	2.81E-01
gene27569	20425	NC_000077.6	Shmt1	2.81E-01
gene35655	1.1E+08	NC_000080.6	Gm15735	2.80E-01

gene38760	106489	NC_000083.6	Sft2d1	2.80E-01
gene23426	214812	NC_000075.6	Zfp609	2.80E-01
gene27039	16533	NC_000077.6	Kcnmb1	2.80E-01
gene14371	75593	NC_000072.6	Malsu1	2.80E-01
gene1679	67196	NC_000067.6	Ube2t	2.80E-01
gene41687	74453	NC_000084.6	Cfap53	2.80E-01
gene35464	70898	NC_000080.6	Slain1os	2.80E-01
gene19460	233833	NC_000073.6	Tnrc6a	2.80E-01
gene437	211798	NC_000067.6	Mfsd9	2.80E-01
gene41641	21413	NC_000084.6	Tcf4	2.80E-01
gene1350	227446	NC_000067.6	2310035C23Rik	2.80E-01
gene42596	240613	NC_000085.6	9930021J03Rik	2.80E-01
gene34871	68631	NC_000080.6	Cryl1	2.79E-01
gene13719	170772	NC_000072.6	Glcci1	2.79E-01
gene42959	76952	NC_000085.6	Nt5c2	2.79E-01
gene7149	=Gene;ger	NC_000069.6	LOC105244487	2.79E-01
gene25564	16904	NC_000076.6	Gzmm	2.79E-01
gene24936	1E+08	NC_000076.6	Gm31930	2.79E-01
gene29850	71916	NC_000078.6	Dus4l	2.79E-01
gene5765	1.1E+08	NC_000068.7	Gm11457	2.79E-01
gene23935	72507	NC_000075.6	Dzip1l	2.79E-01
gene21948	436062	NC_000074.6	Fam92b	2.79E-01
gene42719	107371	NC_000085.6	Exoc6	2.78E-01
gene23529	21406	NC_000075.6	Tcf12	2.78E-01
gene12408	19249	NC_000071.6	Ptpn13	2.78E-01
gene36508	626615	NC_000081.6	Apol11a	2.78E-01
gene32511	212483	NC_000079.6	Fam193b	2.78E-01
gene19912	57913	NC_000073.6	Pidd1	2.78E-01
gene9765	230594	NC_000070.6	Zcchc11	2.78E-01
gene8028	108058	NC_000069.6	Camk2d	2.78E-01
gene22503	235044	NC_000075.6	Plppr2	2.78E-01
gene15980	14011	NC_000072.6	Etv6	2.78E-01
gene4649	75517	NC_000068.7	Ccdc34os	2.77E-01
gene2057	20344	NC_000067.6	Selp	2.77E-01
gene1000	76867	NC_000067.6	Rhbdd1	2.77E-01
gene30523	73736	NC_000078.6	Fcf1	2.77E-01
gene8952	53614	NC_000070.6	Reck	2.77E-01
gene28164	66274	NC_000077.6	Lyrm9	2.77E-01
gene13556	70717	NC_000071.6	Medag	2.77E-01
gene18469	22691	NC_000073.6	Zscan2	2.76E-01
gene17444	71909	NC_000073.6	Haus5	2.76E-01
gene4819	19361	NC_000068.7	Rad51	2.75E-01
gene36205	211401	NC_000081.6	Mtss1	2.75E-01
gene11161	18669	NC_000071.6	Abcb1b	2.75E-01
gene40788	16774	NC_000084.6	Lama3	2.75E-01
gene33692	1E+08	NC_000080.6	Gm16525	2.75E-01
gene10730	=Gene;ger	NC_000070.6	LOC108168986	2.75E-01
gene31561	17101	NC_000079.6	Lyst	2.75E-01

gene34342	218977	NC_000080.6	Dlgap5	2.75E-01
gene20040	16337	NC_000074.6	Insr	2.74E-01
gene8620	66302	NC_000070.6	Rmdn1	2.74E-01
gene39057	73233	NC_000083.6	Zfp942	2.74E-01
gene5856	1E+08	NC_000068.7	Gm32287	2.74E-01
gene28070	18230	NC_000077.6	Nxn	2.74E-01
gene10899	73348	NC_000070.6	1700045H11Rik	2.74E-01
gene32424	1E+08	NC_000079.6	Gm33168	2.74E-01
gene29004	78943	NC_000077.6	Ern1	2.74E-01
gene13344	108723	NC_000071.6	Card11	2.74E-01
gene13645	73288	NC_000072.6	Vps50	2.74E-01
gene32429	11992	NC_000079.6	Auh	2.74E-01
gene3055	77683	NC_000068.7	Ehmt1	2.74E-01
gene23129	320051	NC_000075.6	Exph5	2.74E-01
gene27704	14457	NC_000077.6	Gas7	2.73E-01
gene11264	15234	NC_000071.6	Hgf	2.73E-01
gene27082	15366	NC_000077.6	Hmmr	2.73E-01
gene11900	333789	NC_000071.6	N4bp2	2.73E-01
gene11337	20817	NC_000071.6	Srpk2	2.73E-01
gene20993	234373	NC_000074.6	Sugp2	2.72E-01
gene27734	237823	NC_000077.6	Pfas	2.72E-01
gene33844	16529	NC_000080.6	Kcnk5	2.72E-01
gene29622	217431	NC_000078.6	Nol10	2.72E-01
gene33761	246103	NC_000080.6	Atxn7	2.72E-01
gene11477	22171	NC_000071.6	Tyms	2.72E-01
gene11728	231207	NC_000071.6	Cpeb2	2.72E-01
gene41188	73516	NC_000084.6	1700086O06Rik	2.72E-01
gene19297	68815	NC_000073.6	Btbd10	2.71E-01
gene29418	319530	NC_000077.6	Zfp750	2.71E-01
gene7182	72640	NC_000069.6	Mex3a	2.71E-01
gene41537	494504	NC_000084.6	Apcdd1	2.71E-01
gene22249	17386	NC_000075.6	Mmp13	2.71E-01
gene20602	21951	NC_000074.6	Tnks	2.71E-01
gene40790	72747	NC_000084.6	Ttc39c	2.71E-01
gene19774	77252	NC_000073.6	9430038I01Rik	2.70E-01
gene100	240725	NC_000067.6	Sulf1	2.70E-01
gene11156	319653	NC_000071.6	Slc25a40	2.70E-01
gene23732	17436	NC_000075.6	Me1	2.70E-01
gene42569	20510	NC_000085.6	Slc1a1	2.70E-01
gene16280	18725	NC_000073.6	Pira2	2.70E-01
gene29521	74682	NC_000078.6	Wdr35	2.70E-01
gene23858	1E+08	NC_000075.6	Gm16794	2.70E-01
gene24609	215798	NC_000076.6	Adgrg6	2.69E-01
gene15353	56353	NC_000072.6	Rybp	2.69E-01
gene15985	66813	NC_000072.6	Bcl2l14	2.69E-01
gene42516	14297	NC_000085.6	Fxn	2.69E-01
gene35111	13522	NC_000080.6	Adam28	2.69E-01
gene24688	73990	NC_000076.6	4930455C13Rik	2.69E-01

gene23868	=Gene;ger	NC_000075.6	LOC108167675	2.69E-01
gene24384	638068	NC_000075.6	Gm7229	2.68E-01
gene15848	1E+08	NC_000072.6	Gm15987	2.68E-01
gene7495	22365	NC_000069.6	Vps45	2.68E-01
gene6434	18805	NC_000069.6	Pld1	2.68E-01
gene30083	1E+08	NC_000078.6	Srp54c	2.68E-01
gene21786	244650	NC_000074.6	Phlpp2	2.67E-01
gene20446	330723	NC_000074.6	Htra4	2.67E-01
gene42219	1E+08	NC_000085.6	Gm30042	2.67E-01
gene10504	22417	NC_000070.6	Wnt4	2.67E-01
gene23947	ene;gene=L	NC_000075.6	LOC108167700	2.67E-01
gene40659	1E+08	NC_000083.6	Gm20939	2.67E-01
gene40699	75415	NC_000084.6	Arhgap12	2.67E-01
gene32237	328232	NC_000079.6	Gfod1	2.66E-01
gene9656	18578	NC_000070.6	Pde4b	2.66E-01
gene24218	20776	NC_000075.6	Tmie	2.66E-01
gene10182	76850	NC_000070.6	ago-04	2.66E-01
gene23291	270163	NC_000075.6	Myo9a	2.66E-01
gene28263	20557	NC_000077.6	Slfn3	2.66E-01
gene37965	68194	NC_000082.6	Ndufb4	2.65E-01
gene19287	21676	NC_000073.6	Tead1	2.65E-01
gene21748	18104	NC_000074.6	Nqo1	2.65E-01
gene28344	70422	NC_000077.6	Ints2	2.65E-01
gene34421	546611	NC_000080.6	Klhl33	2.65E-01
gene35621	223254	NC_000080.6	Farp1	2.65E-01
gene21356	108682	NC_000074.6	Gpt2	2.65E-01
gene34989	268752	NC_000080.6	Wdfy2	2.65E-01
gene23742	382090	NC_000075.6	Cep162	2.65E-01
gene32826	218314	NC_000079.6	Zfp595	2.65E-01
gene37591	26462	NC_000082.6	Txnrd2	2.65E-01
gene37987	12519	NC_000082.6	Cd80	2.65E-01
gene37801	1E+08	NC_000082.6	4632428C04Rik	2.65E-01
gene9647	16847	NC_000070.6	Lepr	2.64E-01
gene11308	242864	NC_000071.6	Napepld	2.64E-01
gene4797	214230	NC_000068.7	Pak6	2.64E-01
gene12965	56325	NC_000071.6	Abcb9	2.64E-01
gene15480	74244	NC_000072.6	Atg7	2.63E-01
gene23133	11920	NC_000075.6	Atm	2.63E-01
gene7989	1E+08	NC_000069.6	Gm34577	2.63E-01
gene37400	74374	NC_000082.6	Clec16a	2.63E-01
gene8281	229937	NC_000069.6	Znhit6	2.63E-01
gene475	67883	NC_000067.6	Uxs1	2.63E-01
gene36403	72960	NC_000081.6	Top1mt	2.63E-01
gene41617	70799	NC_000084.6	Cep192	2.63E-01
gene16122	74741	NC_000072.6	C2cd5	2.63E-01
gene29478	320817	NC_000078.6	Atad2b	2.63E-01
gene40770	68434	NC_000084.6	1010001N08Rik	2.63E-01
gene40532	107766	NC_000083.6	Haao	2.62E-01

gene22343	170748	NC_000075.6	Smco4	2.62E-01
gene34021	54650	NC_000080.6	Sfmbt1	2.62E-01
gene36821	223752	NC_000081.6	Gramd4	2.62E-01
gene6932	545531	NC_000069.6	Gm5848	2.62E-01
gene21537	1E+08	NC_000074.6	Gm31036	2.62E-01
gene15398	74729	NC_000072.6	Setmar	2.62E-01
gene25015	76563	NC_000076.6	Qrsl1	2.62E-01
gene5626	66642	NC_000068.7	Ctnnbl1	2.61E-01
gene16182	232533	NC_000072.6	Stk38l	2.61E-01
gene3245	99151	NC_000068.7	Cercam	2.61E-01
gene20559	22427	NC_000074.6	Wrn	2.61E-01
gene26758	14783	NC_000077.6	Grb10	2.61E-01
gene21251	56367	NC_000074.6	Scoc	2.61E-01
gene30554	76411	NC_000078.6	Ift43	2.61E-01
gene24706	319887	NC_000076.6	E030030I06Rik	2.60E-01
gene23405	214424	NC_000075.6	Parp16	2.60E-01
gene29014	320162	NC_000077.6	Cep95	2.60E-01
gene28634	12295	NC_000077.6	Cacnb1	2.60E-01
gene39071	74149	NC_000083.6	Zfp946	2.60E-01
gene22535	68743	NC_000075.6	Anln	2.60E-01
gene5699	59091	NC_000068.7	Jph2	2.60E-01
gene32264	16468	NC_000079.6	Jarid2	2.60E-01
gene34073	67085	NC_000080.6	1700024G13Rik	2.60E-01
gene8922	230098	NC_000070.6	Arhgef39	2.59E-01
gene14015	21848	NC_000072.6	Trim24	2.59E-01
gene32567	56278	NC_000079.6	Gkap1	2.59E-01
gene16524	22775	NC_000073.6	Zik1	2.59E-01
gene3682	66860	NC_000068.7	Tanc1	2.59E-01
gene976	252903	NC_000067.6	Ap1s3	2.59E-01
gene16043	320135	NC_000072.6	BC049715	2.59E-01
gene24681	17863	NC_000076.6	Myb	2.58E-01
gene35748	27401	NC_000081.6	Skp2	2.58E-01
gene10406	1E+08	NC_000070.6	Zfp683	2.58E-01
gene10832	70433	NC_000070.6	Draxin	2.58E-01
gene8178	108943	NC_000069.6	Trmt10a	2.58E-01
gene7650	229644	NC_000069.6	Trim45	2.58E-01
gene5654	71715	NC_000068.7	Dhx35	2.58E-01
gene29690	1.1E+08	NC_000078.6	Gm40855	2.58E-01
gene24884	319638	NC_000076.6	Nt5dc1	2.58E-01
gene10407	230806	NC_000070.6	Aim1l	2.58E-01
gene22979	319742	NC_000075.6	Mpzl3	2.57E-01
gene4790	27103	NC_000068.7	Eif2ak4	2.57E-01
gene24921	327744	NC_000076.6	E130307A14Rik	2.57E-01
gene35317	1E+08	NC_000080.6	Gm4632	2.57E-01
gene34326	72391	NC_000080.6	Cdkn3	2.57E-01
gene1660	11539	NC_000067.6	Adora1	2.57E-01
gene18068	13009	NC_000073.6	Csrp3	2.57E-01
gene6505	51938	NC_000069.6	Ccdc39	2.57E-01

gene30372	53618	NC_000078.6	Fut8	2.57E-01
gene7833	99512	NC_000069.6	Wdr47	2.57E-01
gene687	57750	NC_000067.6	Wdr12	2.57E-01
gene2344	98256	NC_000067.6	Kmo	2.57E-01
gene5100	16434	NC_000068.7	Itpa	2.57E-01
gene24774	11846	NC_000076.6	Arg1	2.56E-01
gene12503	231570	NC_000071.6	A830010M20Rik	2.56E-01
gene24974	140742	NC_000076.6	Sesn1	2.56E-01
gene35773	81799	NC_000081.6	C1qtnf3	2.56E-01
gene18835	11872	NC_000073.6	Art2b	2.56E-01
gene37588	11877	NC_000082.6	Arvcf	2.56E-01
gene41012	633395	NC_000084.6	Gm10548	2.56E-01
gene7571	59020	NC_000069.6	Pdzk1	2.56E-01
gene29024	1.1E+08	NC_000077.6	Gm11715	2.56E-01
gene3206	12831	NC_000068.7	Col5a1	2.56E-01
gene3782	1E+08	NC_000068.7	Gm36393	2.56E-01
gene32150	1E+08	NC_000079.6	Gm30489	2.56E-01
gene4840	70645	NC_000068.7	Oip5	2.55E-01
gene34055	56794	NC_000080.6	Hacl1	2.55E-01
gene5566	56046	NC_000068.7	Uqcc1	2.55E-01
gene3233	171171	NC_000068.7	Ntng2	2.55E-01
gene8275	23844	NC_000069.6	Clca1	2.55E-01
gene9727	72938	NC_000070.6	Hspb11	2.55E-01
gene15585	19365	NC_000072.6	Rad52	2.54E-01
gene27627	170740	NC_000077.6	Zfp287	2.54E-01
gene27044	94176	NC_000077.6	Dock2	2.54E-01
gene42441	271564	NC_000085.6	Vps13a	2.54E-01
gene2083	74106	NC_000067.6	Dcaf6	2.54E-01
gene9666	242585	NC_000070.6	Slc35d1	2.54E-01
gene23498	1.1E+08	NC_000075.6	Gm26849	2.54E-01
gene30226	68055	NC_000078.6	Atp5s	2.54E-01
gene42013	225861	NC_000085.6	Snx32	2.54E-01
gene2208	17528	NC_000067.6	Mpz	2.54E-01
gene20753	408022	NC_000074.6	Primpol	2.53E-01
gene24781	432442	NC_000076.6	Akap7	2.53E-01
gene32584	18212	NC_000079.6	Ntrk2	2.53E-01
gene10177	329941	NC_000070.6	Col8a2	2.53E-01
gene10959	13368	NC_000070.6	Dffb	2.53E-01
gene105	17978	NC_000067.6	Ncoa2	2.53E-01
gene27607	432572	NC_000077.6	Specc1	2.52E-01
gene789	68691	NC_000067.6	Kansl1l	2.52E-01
gene13905	18181	NC_000072.6	Nrf1	2.52E-01
gene15009	243510	NC_000072.6	Ccdc142	2.52E-01
gene10079	66966	NC_000070.6	Trit1	2.52E-01
gene34241	1E+08	NC_000080.6	Gm32427	2.52E-01
gene17465	76415	NC_000073.6	Fam187b	2.52E-01
gene27653	54710	NC_000077.6	Hs3st3b1	2.52E-01
gene2778	67856	NC_000068.7	Echdc3	2.52E-01

gene24565	70097	NC_000076.6	Sash1	2.52E-01
gene4988	108011	NC_000068.7	Ap4e1	2.52E-01
gene26820	114716	NC_000077.6	Spred2	2.52E-01
gene3814	75763	NC_000068.7	Dcaf17	2.52E-01
gene1826	96875	NC_000067.6	Prg4	2.52E-01
gene5643	68108	NC_000068.7	Snhg17	2.52E-01
gene32151	74145	NC_000079.6	F13a1	2.52E-01
gene7718	269473	NC_000069.6	Lrig2	2.52E-01
gene3627	71409	NC_000068.7	Fmnl2	2.52E-01
gene2633	22033	NC_000067.6	Traf5	2.52E-01
gene10971	230971	NC_000070.6	Megf6	2.51E-01
gene15558	19713	NC_000072.6	Ret	2.51E-01
gene22924	53376	NC_000075.6	Usp2	2.51E-01
gene776	18711	NC_000067.6	Pikfyve	2.51E-01
gene17971	211480	NC_000073.6	Kcnj14	2.51E-01
gene19775	30926	NC_000073.6	Glrx3	2.51E-01
gene15509	81896	NC_000072.6	Ift122	2.51E-01
gene27714	18208	NC_000077.6	Ntn1	2.51E-01
gene10653	1E+08	NC_000070.6	Gm35295	2.51E-01
gene13786	22413	NC_000072.6	Wnt2	2.51E-01
gene34057	105522	NC_000080.6	Ankrd28	2.51E-01
gene18472	68039	NC_000073.6	Nmb	2.51E-01
gene4834	68142	NC_000068.7	Ino80	2.51E-01
gene11043	230996	NC_000070.6	9430015G10Rik	2.51E-01
gene42006	14283	NC_000085.6	Fosl1	2.50E-01
gene37470	93734	NC_000082.6	Mpv17l	2.50E-01
gene19388	434234	NC_000073.6	2610020H08Rik	2.50E-01
gene11972	72313	NC_000071.6	Fryl	2.50E-01
gene42522	226041	NC_000085.6	Pgm5	2.50E-01
gene20752	71876	NC_000074.6	Cenpu	2.50E-01
gene3395	227743	NC_000068.7	Mapkap1	2.50E-01
gene23116	629557	NC_000075.6	Gm6981	2.50E-01
gene22018	30927	NC_000074.6	Snai3	2.50E-01
gene3308	11898	NC_000068.7	Ass1	2.50E-01
gene27263	27274	NC_000077.6	Zfp354b	2.49E-01
gene3263	70266	NC_000068.7	Kyat1	2.49E-01
gene21806	76527	NC_000074.6	Il34	2.49E-01
gene130	21749	NC_000067.6	Terf1	2.49E-01
gene3662	77767	NC_000068.7	Ermn	2.49E-01
gene1724	1E+08	NC_000067.6	Gm19705	2.49E-01
gene35192	1E+08	NC_000080.6	Gm34588	2.49E-01
gene26259	1E+08	NC_000076.6	Gm34545	2.48E-01
gene23137	1E+08	NC_000075.6	Gm16124	2.48E-01
gene328	226970	NC_000067.6	Arhgef4	2.48E-01
gene42947	24069	NC_000085.6	Sufu	2.48E-01
gene984	241128	NC_000067.6	Fam124b	2.48E-01
gene6401	81004	NC_000069.6	Tbl1xr1	2.48E-01
gene15130	1.1E+08	NC_000072.6	Gm38851	2.48E-01

gene1758	1.1E+08	NC_000067.6	Gm41962	2.47E-01
gene13956	66075	NC_000072.6	Chchd3	2.47E-01
gene7404	75778	NC_000069.6	Them4	2.47E-01
gene14388	56524	NC_000072.6	Mpp6	2.47E-01
gene39600	81877	NC_000083.6	Tnxb	2.47E-01
gene7940	66789	NC_000069.6	Alg14	2.46E-01
gene2105	1E+08	NC_000067.6	Ildr2	2.46E-01
gene17605	72140	NC_000073.6	Cep89	2.46E-01
gene42860	93685	NC_000085.6	Entpd7	2.46E-01
gene42803	212391	NC_000085.6	Lcor	2.46E-01
gene10854	56273	NC_000070.6	Pex14	2.46E-01
gene12532	1E+08	NC_000071.6	Gm34319	2.46E-01
gene10892	68703	NC_000070.6	Rere	2.46E-01
gene7773	16490	NC_000069.6	Kcna2	2.46E-01
gene37529	106369	NC_000082.6	Ypel1	2.46E-01
gene27602	69640	NC_000077.6	Fam83g	2.46E-01
gene6008	1E+08	NC_000068.7	Gm30189	2.45E-01
gene7062	229473	NC_000069.6	D930015E06Rik	2.45E-01
gene14662	16162	NC_000072.6	Il12rb2	2.45E-01
gene7590	83679	NC_000069.6	Pde4dip	2.45E-01
gene7684	78491	NC_000069.6	Tspan2os	2.44E-01
gene14983	232146	NC_000072.6	Eva1a	2.44E-01
gene23060	235330	NC_000075.6	Ttc12	2.44E-01
gene18533	75556	NC_000073.6	Cfap161	2.44E-01
gene840	22596	NC_000067.6	Xrcc5	2.44E-01
gene9181	14745	NC_000070.6	Lpar1	2.44E-01
gene15576	211187	NC_000072.6	Lrtm2	2.44E-01
gene30873	268595	NC_000078.6	D430019H16Rik	2.44E-01
gene19494	1E+08	NC_000073.6	Gm30928	2.44E-01
gene5671	64899	NC_000068.7	Lpin3	2.44E-01
gene4474	639658	NC_000068.7	Gm13807	2.44E-01
gene39131	76917	NC_000083.6	Flywch2	2.44E-01
gene8719	52187	NC_000070.6	Rragd	2.44E-01
gene11655	1E+08	NC_000071.6	Gm32725	2.43E-01
gene35477	72486	NC_000080.6	Rnf219	2.43E-01
gene39196	328779	NC_000083.6	Hs3st6	2.43E-01
gene23145	244882	NC_000075.6	Tnfaip8l3	2.43E-01
gene35738	ene;gene=l	NC_000081.6	LOC102634078	2.43E-01
gene41847	50764	NC_000084.6	Fbxo15	2.43E-01
gene1532	226412	NC_000067.6	R3hdm1	2.42E-01
gene11107	71382	NC_000071.6	Pex1	2.42E-01
gene29453	217378	NC_000078.6	Dnajc27	2.42E-01
gene7581	77578	NC_000069.6	Bcl9	2.42E-01
gene3408	74016	NC_000068.7	Phf19	2.42E-01
gene27289	97775	NC_000077.6	D930048N14Rik	2.42E-01
gene30460	217692	NC_000078.6	Sipa1l1	2.42E-01
gene17119	54678	NC_000073.6	Zfp108	2.42E-01
gene6420	13605	NC_000069.6	Ect2	2.42E-01

gene9609	67299	NC_000070.6	Dock7	2.42E-01
gene34869	14623	NC_000080.6	Gjb6	2.41E-01
gene9007	74753	NC_000070.6	Trmo	2.41E-01
gene40548	1E+08	NC_000083.6	Gm36279	2.41E-01
gene4039	269295	NC_000068.7	Rtn4rl2	2.41E-01
gene23544	21778	NC_000075.6	Tex9	2.41E-01
gene36034	68588	NC_000081.6	Cthrc1	2.41E-01
gene5125	51902	NC_000068.7	Rnf24	2.41E-01
gene21835	22640	NC_000074.6	Zfp1	2.41E-01
gene32170	68750	NC_000079.6	Rreb1	2.41E-01
gene11048	74183	NC_000070.6	Perm1	2.41E-01
gene10364	230793	NC_000070.6	Ahdc1	2.40E-01
gene28585	83395	NC_000077.6	Sp6	2.40E-01
gene34880	57258	NC_000080.6	Xpo4	2.40E-01
gene2298	383619	NC_000067.6	Aim2	2.40E-01
gene21522	20295	NC_000074.6	Ccl17	2.40E-01
gene30780	217837	NC_000078.6	Itpk1	2.40E-01
gene23479	93697	NC_000075.6	Ice2	2.40E-01
gene10168	230751	NC_000070.6	Oscp1	2.40E-01
gene26926	216613	NC_000077.6	Ccdc85a	2.40E-01
gene28822	11754	NC_000077.6	Aoc3	2.40E-01
gene37060	105833	NC_000081.6	Ccdc65	2.40E-01
gene28809	103768	NC_000077.6	Tubg2	2.39E-01
gene38030	106248	NC_000082.6	Qtrtd1	2.39E-01
gene761	241075	NC_000067.6	Plekhm3	2.39E-01
gene29151	217306	NC_000077.6	Cd300e	2.39E-01
gene21364	102093	NC_000074.6	Phkb	2.39E-01
gene39351	21463	NC_000083.6	Tcp11	2.38E-01
gene37874	140474	NC_000082.6	Muc4	2.38E-01
gene6264	229055	NC_000069.6	Zbtb10	2.38E-01
gene17606	1.1E+08	NC_000073.6	Gm42378	2.38E-01
gene29378	209027	NC_000077.6	Pycr1	2.38E-01
gene10516	110351	NC_000070.6	Rap1gap	2.38E-01
gene40773	225182	NC_000084.6	Rbbp8	2.37E-01
gene32705	76251	NC_000079.6	Ercc6l2	2.37E-01
gene33190	27015	NC_000079.6	Polk	2.37E-01
gene5801	14049	NC_000068.7	Eya2	2.37E-01
gene36060	170719	NC_000081.6	Oxr1	2.37E-01
gene14385	109648	NC_000072.6	Npy	2.37E-01
gene12394	72145	NC_000071.6	Wdfy3	2.37E-01
gene23584	14697	NC_000075.6	Gnb5	2.37E-01
gene3361	70730	NC_000068.7	6330409D20Rik	2.37E-01
gene32994	77041	NC_000079.6	Arsk	2.37E-01
gene40786	225187	NC_000084.6	Ankrd29	2.36E-01
gene19440	67417	NC_000073.6	Ears2	2.36E-01
gene31977	214579	NC_000079.6	Aldh5a1	2.36E-01
gene40161	66968	NC_000083.6	Plin5	2.36E-01
gene27283	237759	NC_000077.6	Col23a1	2.36E-01

gene5367	69277	NC_000068.7	3300002I08Rik	2.36E-01
gene13987	53621	NC_000072.6	Cnot4	2.36E-01
gene3520	241327	NC_000068.7	Olfml2a	2.36E-01
gene35110	58860	NC_000080.6	Adamdec1	2.35E-01
gene38754	12458	NC_000083.6	Ccr6	2.35E-01
gene17284	22717	NC_000073.6	Zfp59	2.35E-01
gene15612	53857	NC_000072.6	Tuba8	2.35E-01
gene838	111175	NC_000067.6	Pecr	2.35E-01
gene9029	1E+08	NC_000070.6	Gm32435	2.35E-01
gene13863	627049	NC_000072.6	Zfp800	2.35E-01
gene2134	18514	NC_000067.6	Pbx1	2.35E-01
gene31526	238505	NC_000079.6	Mtr	2.35E-01
gene11605	231128	NC_000071.6	Fam193a	2.34E-01
gene7800	14867	NC_000069.6	Gstm6	2.34E-01
gene19635	101490	NC_000073.6	Inpp5f	2.34E-01
gene2483	53791	NC_000067.6	Tlr5	2.34E-01
gene19100	=Gene;ger	NC_000073.6	LOC108167454	2.34E-01
gene3783	241452	NC_000068.7	Dhrs9	2.34E-01
gene5019	12235	NC_000068.7	Bub1	2.34E-01
gene26707	192950	NC_000077.6	Nacad	2.34E-01
gene12886	71985	NC_000071.6	Acad10	2.34E-01
gene19978	27027	NC_000073.6	Tspan32	2.34E-01
gene28687	217169	NC_000077.6	Tns4	2.33E-01
gene12925	109202	NC_000071.6	A930024E05Rik	2.33E-01
gene20560	75029	NC_000074.6	Purg	2.33E-01
gene37543	15160	NC_000082.6	Serpind1	2.33E-01
gene12409	75750	NC_000071.6	Slc10a6	2.33E-01
gene14425	18025	NC_000072.6	Nfe2l3	2.33E-01
gene22327	64931	NC_000075.6	Izumo1r	2.33E-01
gene35191	14586	NC_000080.6	Gfra2	2.33E-01
gene35904	69574	NC_000081.6	Cmbl	2.33E-01
gene27383	52570	NC_000077.6	Ccdc69	2.33E-01
gene35674	14600	NC_000081.6	Ghr	2.33E-01
gene4430	228361	NC_000068.7	Ambra1	2.33E-01
gene34304	67651	NC_000080.6	4930527F14Rik	2.33E-01
gene2165	320078	NC_000067.6	Olfml2b	2.32E-01
gene22159	244668	NC_000074.6	Sipa1l2	2.32E-01
gene8296	65086	NC_000069.6	Lpar3	2.32E-01
gene24028	654798	NC_000075.6	D030055H07Rik	2.32E-01
gene3971	228094	NC_000068.7	Cerkl	2.32E-01
gene29856	19088	NC_000078.6	Prkar2b	2.32E-01
gene24229	17350	NC_000075.6	Mlh1	2.32E-01
gene27442	103836	NC_000077.6	Zfp692	2.32E-01
gene31059	380787	NC_000078.6	A230065H16Rik	2.32E-01
gene5438	228778	NC_000068.7	6820408C15Rik	2.32E-01
gene41751	1E+08	NC_000084.6	Epg5	2.32E-01
gene40480	319625	NC_000083.6	Galm	2.31E-01
gene39044	319870	NC_000083.6	9330136K24Rik	2.31E-01

gene38750	66832	NC_000083.6	Rsph3a	2.31E-01
gene24098	19654	NC_000075.6	Rbm6	2.31E-01
gene35116	20855	NC_000080.6	Stc1	2.31E-01
gene2964	72668	NC_000068.7	Skida1	2.31E-01
gene2461	13800	NC_000067.6	Enah	2.31E-01
gene42865	71972	NC_000085.6	Dnmbp	2.31E-01
gene34092	26419	NC_000080.6	Mapk8	2.30E-01
gene32279	380842	NC_000079.6	Stmnd1	2.30E-01
gene9864	1E+08	NC_000070.6	Cyp4a32	2.30E-01
gene37789	239796	NC_000082.6	Mb21d2	2.30E-01
gene35476	1E+08	NC_000080.6	Gm35623	2.30E-01
gene26328	75030	NC_000076.6	4930503E24Rik	2.30E-01
gene30727	1E+08	NC_000078.6	3300002A11Rik	2.30E-01
gene33858	78787	NC_000080.6	Usp54	2.30E-01
gene5611	383766	NC_000068.7	Tldc2	2.30E-01
gene10411	70012	NC_000070.6	Cep85	2.30E-01
gene38142	208650	NC_000082.6	Cblb	2.30E-01
gene4904	241627	NC_000068.7	Wdr76	2.30E-01
gene10850	69743	NC_000070.6	Casz1	2.29E-01
gene1352	21934	NC_000067.6	Tnfrsf11a	2.29E-01
gene12357	76003	NC_000071.6	Tmem150cos	2.29E-01
gene35656	70551	NC_000080.6	Tmtc4	2.29E-01
gene25183	53374	NC_000076.6	Chst3	2.29E-01
gene33332	218544	NC_000079.6	Sgtb	2.29E-01
gene37827	11815	NC_000082.6	Apod	2.29E-01
gene9926	66743	NC_000070.6	Rnf220	2.29E-01
gene19310	18576	NC_000073.6	Pde3b	2.29E-01
gene32289	22017	NC_000079.6	Tpmt	2.29E-01
gene37366	239706	NC_000082.6	Mettl22	2.29E-01
gene26036	216274	NC_000076.6	Cep290	2.29E-01
gene39294	240055	NC_000083.6	Neurl1b	2.29E-01
gene18760	233552	NC_000073.6	Gdpd5	2.29E-01
gene19104	1E+08	NC_000073.6	Gm4070	2.28E-01
gene37009	19011	NC_000081.6	Endou	2.28E-01
gene33722	93732	NC_000080.6	Acox2	2.28E-01
gene8752	69329	NC_000070.6	Cfap206	2.28E-01
gene34743	74359	NC_000080.6	4931414P19Rik	2.28E-01
gene27233	14584	NC_000077.6	Gfpt2	2.28E-01
gene158	1E+08	NC_000067.6	Gm28154	2.28E-01
gene24570	78808	NC_000076.6	Stxbp5	2.28E-01
gene37984	85031	NC_000082.6	Pla1a	2.28E-01
gene11669	59056	NC_000071.6	Evc	2.28E-01
gene30564	68737	NC_000078.6	Angel1	2.28E-01
gene10383	69539	NC_000070.6	Trnp1	2.27E-01
gene38353	12228	NC_000082.6	Btg3	2.27E-01
gene4642	12064	NC_000068.7	Bdnf	2.27E-01
gene11341	78697	NC_000071.6	Pus7	2.27E-01
gene6560	229227	NC_000069.6	4932438A13Rik	2.27E-01

gene37314	268857	NC_000082.6	Nlrc3	2.27E-01
gene27603	212627	NC_000077.6	Prpsap2	2.27E-01
gene9643	11639	NC_000070.6	Ak4	2.27E-01
gene33356	76582	NC_000079.6	Ipo11	2.27E-01
gene13929	1.1E+08	NC_000072.6	Gm38781	2.26E-01
gene25221	1.1E+08	NC_000076.6	Gm40674	2.26E-01
gene24195	72341	NC_000075.6	Elp6	2.26E-01
gene12888	20239	NC_000071.6	Atxn2	2.26E-01
gene18700	244144	NC_000073.6	Usp35	2.26E-01
gene13993	1.1E+08	NC_000072.6	Gm38788	2.26E-01
gene38629	72058	NC_000082.6	Igsf5	2.26E-01
gene35452	1E+08	NC_000080.6	Gm34643	2.26E-01
gene6181	269400	NC_000068.7	Rtel1	2.26E-01
gene12879	77462	NC_000071.6	Tmem116	2.26E-01
gene6285	66371	NC_000069.6	Chmp4c	2.26E-01
gene19996	79202	NC_000073.6	Tnfrsf22	2.26E-01
gene11929	666938	NC_000071.6	Bend4	2.25E-01
gene11797	414111	NC_000071.6	C130083M11Rik	2.25E-01
gene35900	252967	NC_000081.6	Ropn1l	2.25E-01
gene2401	226751	NC_000067.6	Cdc42bpa	2.25E-01
gene6538	654424	NC_000069.6	Mccc1os	2.25E-01
gene8136	229841	NC_000069.6	Cenpe	2.25E-01
gene26883	73873	NC_000077.6	Fam161a	2.25E-01
gene24843	211329	NC_000076.6	Ncoa7	2.25E-01
gene28480	104601	NC_000077.6	Mycbpap	2.24E-01
gene15847	70809	NC_000072.6	Clec2g	2.24E-01
gene16175	1E+08	NC_000072.6	Gm35596	2.24E-01
gene33254	1.1E+08	NC_000079.6	Gm41037	2.24E-01
gene35080	12934	NC_000080.6	Dpysl2	2.24E-01
gene35371	219249	NC_000080.6	Tdrd3	2.24E-01
gene29454	104111	NC_000078.6	Adcy3	2.24E-01
gene39215	207209	NC_000083.6	Ccdc154	2.24E-01
gene30910	58208	NC_000078.6	Bcl11b	2.24E-01
gene20322	654455	NC_000074.6	Gm21944	2.24E-01
gene21619	12556	NC_000074.6	Cdh16	2.24E-01
gene26918	67030	NC_000077.6	Fancl	2.23E-01
gene24847	15214	NC_000076.6	Hey2	2.23E-01
gene30352	1E+08	NC_000078.6	4930426I24Rik	2.23E-01
gene32961	238722	NC_000079.6	Zfp72	2.23E-01
gene16218	320560	NC_000072.6	Dennd5b	2.23E-01
gene34336	218973	NC_000080.6	Wdhd1	2.23E-01
gene11319	26429	NC_000071.6	Orc5	2.23E-01
gene22870	72828	NC_000075.6	Ubash3b	2.23E-01
gene14023	209032	NC_000072.6	Zc3hav1l	2.22E-01
gene20874	=Gene;ger	NC_000074.6	LOC102638389	2.22E-01
gene41515	225608	NC_000084.6	Sh3tc2	2.22E-01
gene29329	319454	NC_000077.6	Rptoros	2.22E-01
gene33053	17260	NC_000079.6	Mef2c	2.22E-01

gene14339	243372	NC_000072.6	Zfp775	2.22E-01
gene7104	80877	NC_000069.6	Lrba	2.22E-01
gene9153	242474	NC_000070.6	Tmem245	2.22E-01
gene23005	70661	NC_000075.6	Sik3	2.22E-01
gene23149	235380	NC_000075.6	Dmxl2	2.22E-01
gene14036	1E+08	NC_000072.6	Gm38496	2.21E-01
gene41942	68423	NC_000085.6	Ankrd13d	2.21E-01
gene42539	226049	NC_000085.6	Dmrt2	2.21E-01
gene40088	224860	NC_000083.6	Plcl2	2.21E-01
gene41947	171180	NC_000085.6	Syt12	2.21E-01
gene2681	17221	NC_000067.6	Cd46	2.21E-01
gene21002	382056	NC_000074.6	Crtc1	2.21E-01
gene18285	72635	NC_000073.6	Lins1	2.21E-01
gene29081	217258	NC_000077.6	Abca8a	2.21E-01
gene2891	18761	NC_000068.7	Prkcq	2.21E-01
gene32645	1E+08	NC_000079.6	Gm20599	2.21E-01
gene16216	56306	NC_000072.6	Fam60a	2.20E-01
gene11494	16527	NC_000071.6	Kcnk3	2.20E-01
gene40377	320159	NC_000083.6	Fam179a	2.20E-01
gene27417	171212	NC_000077.6	Galnt10	2.20E-01
gene2051	16579	NC_000067.6	Kifap3	2.20E-01
gene14656	320172	NC_000072.6	E230016M11Rik	2.20E-01
gene25605	13637	NC_000076.6	Efna2	2.20E-01
gene23857	245000	NC_000075.6	Atr	2.20E-01
gene26764	1E+08	NC_000077.6	Gm30596	2.20E-01
gene16199	67015	NC_000072.6	Ccdc91	2.20E-01
gene42663	76630	NC_000085.6	Stambpl1	2.20E-01
gene13367	231863	NC_000071.6	Fbxl18	2.20E-01
gene21196	1E+08	NC_000074.6	Gm30389	2.20E-01
gene29157	14813	NC_000077.6	Grin2c	2.20E-01
gene23094	235344	NC_000075.6	Sik2	2.20E-01
gene39218	74154	NC_000083.6	Unkl	2.20E-01
gene32853	212569	NC_000079.6	Zfp273	2.20E-01
gene22318	1E+08	NC_000075.6	Gm10706	2.19E-01
gene14379	231946	NC_000072.6	Fam221a	2.19E-01
gene24665	17761	NC_000076.6	Map7	2.19E-01
gene3802	68986	NC_000068.7	Gad1os	2.19E-01
gene39242	76483	NC_000083.6	Lmf1	2.19E-01
gene16202	330450	NC_000072.6	Far2	2.19E-01
gene3787	320720	NC_000068.7	Fastkd1	2.19E-01
gene13856	101185	NC_000072.6	Pot1a	2.19E-01
gene16317	330460	NC_000073.6	Tmem150b	2.19E-01
gene24664	26408	NC_000076.6	Map3k5	2.19E-01
gene12035	381644	NC_000071.6	Cep135	2.18E-01
gene6627	214048	NC_000069.6	Larp1b	2.18E-01
gene22577	78658	NC_000075.6	Ncapd3	2.18E-01
gene486	403183	NC_000067.6	Mettl21e	2.18E-01
gene21191	1E+08	NC_000074.6	Gm30271	2.18E-01

gene9257	71354	NC_000070.6	Wdr31	2.18E-01
gene4533	72446	NC_000068.7	Prr5l	2.17E-01
gene27577	24082	NC_000077.6	Map2k3os	2.17E-01
gene7671	242126	NC_000069.6	Slc22a15	2.17E-01
gene37622	1E+08	NC_000082.6	Gm35545	2.17E-01
gene30400	77974	NC_000078.6	Rdh12	2.17E-01
gene18829	207728	NC_000073.6	Pde2a	2.17E-01
gene29283	69926	NC_000077.6	Dnah17	2.17E-01
gene7988	71647	NC_000069.6	4930447N08Rik	2.17E-01
gene10018	433752	NC_000070.6	AA415398	2.17E-01
gene29750	21340	NC_000078.6	Taf1b	2.17E-01
gene2727	227525	NC_000068.7	Dclre1c	2.17E-01
gene3955	80744	NC_000068.7	Cwc22	2.17E-01
gene28601	217143	NC_000077.6	Gpr179	2.17E-01
gene25790	237422	NC_000076.6	Ric8b	2.17E-01
gene471	17974	NC_000067.6	Nck2	2.17E-01
gene10222	269585	NC_000070.6	Zscan20	2.16E-01
gene13105	114674	NC_000071.6	Gtf2ird2	2.16E-01
gene29136	432611	NC_000077.6	Dnaic2	2.16E-01
gene27500	22416	NC_000077.6	Wnt3a	2.16E-01
gene4587	320554	NC_000068.7	Tcp11l1	2.16E-01
gene37086	72572	NC_000081.6	Spats2	2.16E-01
gene2613	=Gene;ger	NC_000067.6	LOC105244059	2.16E-01
gene21844	1E+08	NC_000074.6	Gm36531	2.16E-01
gene36524	239554	NC_000081.6	Foxred2	2.16E-01
gene33267	17951	NC_000079.6	Naip5	2.15E-01
gene29326	18164	NC_000077.6	Nptx1	2.15E-01
gene22115	73420	NC_000074.6	Ccsap	2.15E-01
gene23272	18007	NC_000075.6	Neo1	2.15E-01
gene9715	329910	NC_000070.6	Acot11	2.15E-01
gene21532	54672	NC_000074.6	Adgrg3	2.14E-01
gene16278	1E+08	NC_000073.6	Gm15925	2.14E-01
gene9841	1E+08	NC_000070.6	Foxd2os	2.14E-01
gene36135	14042	NC_000081.6	Ext1	2.14E-01
gene27049	70385	NC_000077.6	Spdl1	2.14E-01
gene41499	1E+08	NC_000084.6	Gm35607	2.14E-01
gene15832	232400	NC_000072.6	BC048546	2.14E-01
gene7119	1E+08	NC_000069.6	Gm31940	2.14E-01
gene38704	321007	NC_000083.6	Serac1	2.13E-01
gene28906	70218	NC_000077.6	Kif18b	2.13E-01
gene13877	101359	NC_000072.6	Prrt4	2.13E-01
gene42417	21888	NC_000085.6	Tle4	2.13E-01
gene15574	319618	NC_000072.6	Dcp1b	2.13E-01
gene12429	1E+08	NC_000071.6	BC005561	2.13E-01
gene33980	171463	NC_000080.6	Il17rd	2.12E-01
gene24100	19882	NC_000075.6	Mst1r	2.12E-01
gene12912	12589	NC_000071.6	Ift81	2.12E-01
gene34488	77945	NC_000080.6	Rpgrip1	2.12E-01

gene13084	100929	NC_000071.6	Tyw1	2.12E-01
gene17404	233064	NC_000073.6	Wdr62	2.12E-01
gene1629	240753	NC_000067.6	Plekha6	2.12E-01
gene29347	268515	NC_000077.6	Bahcc1	2.12E-01
gene41045	81500	NC_000084.6	Sil1	2.12E-01
gene7839	320181	NC_000069.6	Fndc7	2.12E-01
gene3734	319876	NC_000068.7	Cobll1	2.11E-01
gene5299	241694	NC_000068.7	Ralgapa2	2.11E-01
gene20384	11979	NC_000074.6	Atp7b	2.11E-01
gene33260	78038	NC_000079.6	Mccc2	2.11E-01
gene16626	619331	NC_000073.6	Zfp551	2.11E-01
gene25016	170728	NC_000076.6	Rtn4ip1	2.10E-01
gene23432	214897	NC_000075.6	Csnk1g1	2.10E-01
gene23191	1E+08	NC_000075.6	Peak1os	2.10E-01
gene15880	503550	NC_000072.6	Klri1	2.10E-01
gene24541	77777	NC_000076.6	Ulbp1	2.10E-01
gene9869	230649	NC_000070.6	Atpaf1	2.10E-01
gene20189	17207	NC_000074.6	Mcf2l	2.10E-01
gene24594	22634	NC_000076.6	Plagl1	2.10E-01
gene20029	211577	NC_000073.6	Mrgprf	2.10E-01
gene5653	71878	NC_000068.7	Fam83d	2.10E-01
gene35435	210789	NC_000080.6	Tbc1d4	2.09E-01
gene23536	235469	NC_000075.6	Zfp280d	2.09E-01
gene19417	13631	NC_000073.6	Eef2k	2.09E-01
gene36817	72026	NC_000081.6	Trmu	2.09E-01
gene35063	1.1E+08	NC_000080.6	Gm41182	2.09E-01
gene24923	19714	NC_000076.6	Rev3l	2.09E-01
gene38040	212514	NC_000082.6	Spice1	2.09E-01
gene30800	68149	NC_000078.6	Otub2	2.09E-01
gene1360	98432	NC_000067.6	Phlpp1	2.09E-01
gene40367	67052	NC_000083.6	Ndc80	2.08E-01
gene11863	57915	NC_000071.6	Tbc1d1	2.08E-01
gene42733	70567	NC_000085.6	Fra10ac1	2.08E-01
gene1748	329260	NC_000067.6	Dennd1b	2.08E-01
gene17953	74490	NC_000073.6	Mamstr	2.08E-01
gene5675	71389	NC_000068.7	Chd6	2.08E-01
gene23200	121021	NC_000075.6	Cspg4	2.08E-01
gene35937	223473	NC_000081.6	Nipal2	2.08E-01
gene18720	17921	NC_000073.6	Myo7a	2.08E-01
gene9177	1E+08	NC_000070.6	Gm35402	2.08E-01
gene8974	269529	NC_000070.6	Fbxo10	2.08E-01
gene38927	1E+08	NC_000083.6	Gm6712	2.08E-01
gene7627	70560	NC_000069.6	Wars2	2.08E-01
gene27912	327958	NC_000077.6	Pitpnm3	2.08E-01
gene42459	225997	NC_000085.6	Trpm6	2.08E-01
gene12816	76199	NC_000071.6	Med13l	2.08E-01
gene9105	381524	NC_000070.6	AI427809	2.08E-01
gene4826	72112	NC_000068.7	Ppp1r14d	2.07E-01

gene42527	226043	NC_000085.6	Cbwd1	2.07E-01
gene2952	67448	NC_000068.7	Plxdc2	2.07E-01
gene2147	19737	NC_000067.6	Rgs5	2.07E-01
gene10593	72754	NC_000070.6	Arhgef10l	2.07E-01
gene11102	12571	NC_000071.6	Cdk6	2.07E-01
gene25349	83675	NC_000076.6	Bicc1	2.07E-01
gene24141	74443	NC_000075.6	P4htm	2.07E-01
gene39043	22710	NC_000083.6	Zfp52	2.07E-01
gene5300	77806	NC_000068.7	A930019D19Rik	2.07E-01
gene20813	234267	NC_000074.6	Gpm6a	2.06E-01
gene20001	78914	NC_000073.6	Nadsyn1	2.06E-01
gene40594	74597	NC_000083.6	4833418N02Rik	2.06E-01
gene29202	192897	NC_000077.6	Itgb4	2.06E-01
gene11822	67249	NC_000071.6	Tbc1d19	2.06E-01
gene3334	13429	NC_000068.7	Dnm1	2.06E-01
gene10928	56226	NC_000070.6	Espn	2.06E-01
gene28148	790911	NC_000077.6	Slc13a2os	2.06E-01
gene12858	14357	NC_000071.6	Dtx1	2.06E-01
gene10977	1E+08	NC_000070.6	Gm13111	2.06E-01
gene30481	=Gene;ger	NC_000078.6	LOC108168018	2.06E-01
gene19021	209387	NC_000073.6	Trim30d	2.06E-01
gene2590	320119	NC_000067.6	Rps6kc1	2.06E-01
gene7225	13638	NC_000069.6	Efna3	2.06E-01
gene38541	16443	NC_000082.6	Itsn1	2.06E-01
gene32968	1E+08	NC_000079.6	Gm20590	2.06E-01
gene39531	1E+08	NC_000083.6	Kifc1	2.06E-01
gene1703	16763	NC_000067.6	Lad1	2.05E-01
gene28781	74407	NC_000077.6	Ttc25	2.05E-01
gene38043	117606	NC_000082.6	Boc	2.05E-01
gene22993	74602	NC_000075.6	4833428L15Rik	2.05E-01
gene2019	72753	NC_000067.6	2810442N19Rik	2.05E-01
gene38085	84544	NC_000082.6	Cd96	2.05E-01
gene15454	14311	NC_000072.6	Cidec	2.05E-01
gene15842	232406	NC_000072.6	BC035044	2.05E-01
gene3195	192166	NC_000068.7	Sardh	2.05E-01
gene42984	1E+08	NC_000085.6	Cfap43	2.05E-01
gene29334	11302	NC_000077.6	Aatk	2.04E-01
gene22570	244757	NC_000075.6	Glb1l2	2.04E-01
gene33252	218506	NC_000079.6	Mrps27	2.04E-01
gene25934	13998	NC_000076.6	Fgd6	2.04E-01
gene15420	30937	NC_000072.6	Lmcd1	2.04E-01
gene15406	75394	NC_000072.6	0610040F04Rik	2.04E-01
gene41954	20743	NC_000085.6	Sptbn2	2.04E-01
gene12374	191578	NC_000071.6	Helq	2.04E-01
gene15753	213522	NC_000072.6	Plekhg6	2.04E-01
gene9578	74519	NC_000070.6	Cyp2j9	2.04E-01
gene28856	50997	NC_000077.6	Mpp2	2.04E-01
gene593	241062	NC_000067.6	Pgap1	2.04E-01

gene25710	70615	NC_000076.6	Ankrd24	2.04E-01
gene8647	269514	NC_000070.6	Fbxl4	2.04E-01
gene2063	171567	NC_000067.6	Nme7	2.04E-01
gene2120	1E+08	NC_000067.6	Gm33610	2.03E-01
gene28169	16706	NC_000077.6	Ksr1	2.03E-01
gene41692	17919	NC_000084.6	Myo5b	2.03E-01
gene7006	76089	NC_000069.6	Rapgef2	2.03E-01
gene13843	93677	NC_000072.6	Lmod2	2.03E-01
gene18837	20480	NC_000073.6	Clpb	2.03E-01
gene13809	214642	NC_000072.6	Cped1	2.03E-01
gene15233	71699	NC_000072.6	Slc41a3	2.03E-01
gene2965	17354	NC_000068.7	Mllt10	2.03E-01
gene18010	54122	NC_000073.6	Uevld	2.03E-01
gene37325	83383	NC_000082.6	Tfap4	2.03E-01
gene19968	16002	NC_000073.6	Igf2	2.02E-01
gene23712	12040	NC_000075.6	Bckdhb	2.02E-01
gene19249	319934	NC_000073.6	Sbf2	2.02E-01
gene18421	208836	NC_000073.6	Fanci	2.02E-01
gene32273	66355	NC_000079.6	Gmpr	2.02E-01
gene36653	321003	NC_000081.6	Xpnpep3	2.02E-01
gene37706	ene=LOC1	NC_000082.6	LOC108168288	2.02E-01
gene17964	54200	NC_000073.6	Sult2b1	2.02E-01
gene13511	72587	NC_000071.6	Pan3	2.02E-01
gene39515	224694	NC_000083.6	Zfp81	2.02E-01
gene30340	319565	NC_000078.6	Syne2	2.01E-01
gene27953	74637	NC_000077.6	Shpk	2.01E-01
gene2754	70024	NC_000068.7	Mcm10	2.01E-01
gene8096	Gene;gene	NC_000069.6	LOC100503594	2.01E-01
gene32846	68271	NC_000079.6	Zfp85os	2.01E-01
gene25209	216011	NC_000076.6	Lrrc20	2.01E-01
gene1207	18627	NC_000067.6	Per2	2.01E-01
gene12541	231583	NC_000071.6	Slc26a1	2.01E-01
gene34774	1E+08	NC_000080.6	Gm20687	2.01E-01
gene17973	14814	NC_000073.6	Grin2d	2.01E-01
gene2237	18106	NC_000067.6	Cd244	2.01E-01
gene33906	71228	NC_000080.6	Dlg5	2.01E-01
gene8975	1E+08	NC_000070.6	Gm30939	2.01E-01
gene37664	12724	NC_000082.6	Clcn2	2.00E-01
gene14978	330361	NC_000072.6	Gcfc2	2.00E-01
gene30750	217830	NC_000078.6	9030617O03Rik	2.00E-01
gene17287	73430	NC_000073.6	1700049G17Rik	2.00E-01
gene29577	71169	NC_000078.6	Nbas	2.00E-01
gene38640	17858	NC_000082.6	Mx2	2.00E-01
gene33250	238803	NC_000079.6	Zfp366	2.00E-01
gene7606	242109	NC_000069.6	Zfp697	2.00E-01
gene30745	104718	NC_000078.6	Ttc7b	2.00E-01
gene19479	77035	NC_000073.6	Kdm8	2.00E-01
gene35264	68705	NC_000080.6	Gtf2f2	2.00E-01

gene81	1E+08	NC_000067.6	Tcf24	2.00E-01
gene30430	68354	NC_000078.6	Plekhd1os	1.99E-01
gene28520	18053	NC_000077.6	Ngfr	1.99E-01
gene37458	74108	NC_000082.6	Parn	1.99E-01
gene3943	228071	NC_000068.7	Sestd1	1.99E-01
gene1713	67313	NC_000067.6	5730559C18Rik	1.99E-01
gene20303	1.1E+08	NC_000074.6	Gm40466	1.99E-01
gene13534	231912	NC_000071.6	Katnal1	1.99E-01
gene7066	80890	NC_000069.6	Trim2	1.99E-01
gene35769	75259	NC_000081.6	4930556M19Rik	1.99E-01
gene38187	66497	NC_000082.6	Cmss1	1.99E-01
gene35772	1E+08	NC_000081.6	Gm34315	1.99E-01
gene20628	244418	NC_000074.6	D8Ertd82e	1.98E-01
gene28875	20533	NC_000077.6	Slc4a1	1.98E-01
gene1266	227377	NC_000067.6	Farp2	1.98E-01
gene33876	11534	NC_000080.6	Adk	1.98E-01
gene7899	72776	NC_000069.6	Sass6	1.98E-01
gene11265	1E+08	NC_000071.6	Gm31113	1.98E-01
gene17867	69578	NC_000073.6	2310016G11Rik	1.98E-01
gene42446	14537	NC_000085.6	Gcnt1	1.97E-01
gene21446	1E+08	NC_000074.6	Gm36243	1.97E-01
gene33382	71991	NC_000079.6	Ercc8	1.97E-01
gene26465	22781	NC_000076.6	Ikzf4	1.97E-01
gene17168	14809	NC_000073.6	Grik5	1.97E-01
gene40	1E+08	NC_000067.6	Gm19026	1.97E-01
gene41453	=Gene;ger	NC_000084.6	LOC108168394	1.97E-01
gene37602	12544	NC_000082.6	Cdc45	1.97E-01
gene3968	1.1E+08	NC_000068.7	Gm39866	1.97E-01
gene23870	114713	NC_000075.6	Rasa2	1.97E-01
gene7829	75504	NC_000069.6	1700013F07Rik	1.97E-01
gene2599	381318	NC_000067.6	Nsl1	1.97E-01
gene37819	268880	NC_000082.6	Xxylt1	1.97E-01
gene30658	71818	NC_000078.6	3200001D21Rik	1.97E-01
gene20232	234086	NC_000074.6	Erich1	1.96E-01
gene21464	71296	NC_000074.6	Crnde	1.96E-01
gene38926	ene;gene=l	NC_000083.6	LOC102640673	1.96E-01
gene23500	1.1E+08	NC_000075.6	Gm40531	1.96E-01
gene40482	67242	NC_000083.6	Gemin6	1.96E-01
gene22658	57810	NC_000075.6	Cdon	1.96E-01
gene39326	319720	NC_000083.6	9630028I04Rik	1.96E-01
gene20880	234311	NC_000074.6	Ddx60	1.96E-01
gene38256	106338	NC_000082.6	Nsun3	1.96E-01
gene10558	1E+08	NC_000070.6	Gm33214	1.95E-01
gene22855	235283	NC_000075.6	Gramd1b	1.95E-01
gene6934	16159	NC_000069.6	Il12a	1.95E-01
gene9290	21949	NC_000070.6	Tnfsf8	1.95E-01
gene22509	77609	NC_000075.6	Ccdc151	1.95E-01
gene9152	54366	NC_000070.6	Ctnnal1	1.95E-01

gene3838	65964	NC_000068.7	Zak	1.95E-01
gene33357	66254	NC_000079.6	Dimt1	1.95E-01
gene39409	74092	NC_000083.6	Tbc1d22bos	1.95E-01
gene21171	=Gene;ger	NC_000074.6	LOC105243223	1.95E-01
gene23764	1E+08	NC_000075.6	Mthfsl	1.95E-01
gene607	628004	NC_000067.6	Gm10561	1.94E-01
gene39639	114654	NC_000083.6	Ly6g6d	1.94E-01
gene9619	320438	NC_000070.6	Alg6	1.94E-01
gene17869	243963	NC_000073.6	Zfp473	1.94E-01
gene26002	382406	NC_000076.6	Poc1b	1.94E-01
gene20876	72333	NC_000074.6	Palld	1.94E-01
gene11966	70701	NC_000071.6	Nipal1	1.94E-01
gene9353	76804	NC_000070.6	Kdm4c	1.94E-01
gene30354	210762	NC_000078.6	Ppp1r36	1.93E-01
gene39055	70081	NC_000083.6	2210404O09Rik	1.93E-01
gene32079	282663	NC_000079.6	Serpinb1b	1.93E-01
gene10413	665186	NC_000070.6	Gm7534	1.93E-01
gene2775	227545	NC_000068.7	Proser2	1.93E-01
gene33041	72852	NC_000079.6	Mblac2	1.93E-01
gene32066	238564	NC_000079.6	Mylk4	1.93E-01
gene28135	216974	NC_000077.6	Proca1	1.93E-01
gene29218	1E+08	NC_000077.6	Gm33250	1.92E-01
gene35729	14573	NC_000081.6	Gdnf	1.92E-01
gene7936	66568	NC_000069.6	Rwdd3	1.92E-01
gene11490	75434	NC_000071.6	1700001C02Rik	1.92E-01
gene11568	277854	NC_000071.6	Depdc5	1.92E-01
gene18658	83671	NC_000073.6	Sytl2	1.92E-01
gene4401	19400	NC_000068.7	Rapsn	1.92E-01
gene157	94227	NC_000067.6	Pi15	1.92E-01
gene2903	16184	NC_000068.7	Il2ra	1.92E-01
gene3747	73668	NC_000068.7	Ttc21b	1.91E-01
gene20686	68797	NC_000074.6	Pdgfrl	1.91E-01
gene8278	52184	NC_000069.6	Odf2l	1.91E-01
gene17293	1E+08	NC_000073.6	Zfp850	1.91E-01
gene9292	21923	NC_000070.6	Tnc	1.91E-01
gene1587	212933	NC_000067.6	Pm20d1	1.91E-01
gene18363	244058	NC_000073.6	Rgma	1.91E-01
gene15087	75541	NC_000072.6	Nat8f4	1.91E-01
gene9023	230145	NC_000070.6	Galnt12	1.91E-01
gene6556	71492	NC_000069.6	Bbs7	1.91E-01
gene7879	69740	NC_000069.6	Dph5	1.91E-01
gene37157	668225	NC_000081.6	Fignl2	1.91E-01
gene42504	71738	NC_000085.6	Mamdc2	1.91E-01
gene28364	77864	NC_000077.6	Ypel2	1.90E-01
gene18082	71974	NC_000073.6	Prmt3	1.90E-01
gene30094	56784	NC_000078.6	Ralgapa1	1.90E-01
gene1208	1E+08	NC_000067.6	Gm35591	1.90E-01
gene189	71877	NC_000067.6	Efhc1	1.90E-01

gene1310	252876	NC_000067.6	Gin1	1.90E-01
gene7078	50754	NC_000069.6	Fbxw7	1.90E-01
gene22427	434377	NC_000075.6	Zfp560	1.90E-01
gene32864	69315	NC_000079.6	1700001L19Rik	1.90E-01
gene23351	75600	NC_000075.6	Calml4	1.90E-01
gene40789	1E+08	NC_000084.6	Gm36232	1.90E-01
gene18298	67009	NC_000073.6	Ttc23	1.90E-01
gene14668	243469	NC_000072.6	Igk	1.89E-01
gene28648	19049	NC_000077.6	Ppp1r1b	1.89E-01
gene1439	76707	NC_000067.6	Clasp1	1.89E-01
gene23221	66812	NC_000075.6	Ppcdc	1.89E-01
gene39346	20630	NC_000083.6	Snrpc	1.89E-01
gene11706	15476	NC_000071.6	Hs3st1	1.89E-01
gene26657	=Gene;ger	NC_000077.6	LOC108167879	1.89E-01
gene8531	76947	NC_000070.6	Ndufaf6	1.89E-01
gene4528	68170	NC_000068.7	B230118H07Rik	1.89E-01
gene15565	22750	NC_000072.6	Zfp9	1.88E-01
gene14581	58909	NC_000072.6	Fam13a	1.88E-01
gene6063	1E+08	NC_000068.7	Gm14305	1.88E-01
gene13403	18861	NC_000071.6	Pms2	1.88E-01
gene23791	21761	NC_000075.6	Morf4l1	1.88E-01
gene19390	73919	NC_000073.6	Lyrm1	1.88E-01
gene35920	54381	NC_000081.6	Cpq	1.88E-01
gene38042	1.1E+08	NC_000082.6	Gm41455	1.88E-01
gene38723	1E+08	NC_000083.6	Tmem181c-ps	1.88E-01
gene21931	68270	NC_000074.6	Dnaaf1	1.88E-01
gene32136	208366	NC_000079.6	Rpp40	1.88E-01
gene32238	68346	NC_000079.6	Sirt5	1.88E-01
gene20962	636741	NC_000074.6	Zfp964	1.87E-01
gene15169	56505	NC_000072.6	Ruvbl1	1.87E-01
gene19604	77613	NC_000073.6	Prss36	1.87E-01
gene31426	238455	NC_000078.6	Macc1	1.87E-01
gene24717	56554	NC_000076.6	Raet1d	1.87E-01
gene11796	414108	NC_000071.6	9230114K14Rik	1.87E-01
gene27767	216846	NC_000077.6	Cntrob	1.87E-01
gene37129	239667	NC_000081.6	Dip2b	1.87E-01
gene8509	65973	NC_000070.6	Asph	1.87E-01
gene39298	73365	NC_000083.6	1700049J03Rik	1.87E-01
gene12500	1E+08	NC_000071.6	Btbd8	1.87E-01
gene21440	109151	NC_000074.6	Chd9	1.87E-01
gene21327	56527	NC_000074.6	Mast1	1.87E-01
gene2669	18845	NC_000067.6	Plxna2	1.87E-01
gene35869	11732	NC_000081.6	Ank	1.87E-01
gene38670	224481	NC_000083.6	Tfb1m	1.86E-01
gene26919	69922	NC_000077.6	Vrk2	1.86E-01
gene1038	621875	NC_000067.6	A530040E14Rik	1.86E-01
gene34939	29820	NC_000080.6	Tnfrsf19	1.86E-01
gene12923	30841	NC_000071.6	Kdm2b	1.86E-01

gene42506	67483	NC_000085.6	1700028P14Rik	1.86E-01
gene25735	216177	NC_000076.6	AU041133	1.86E-01
gene14484	330319	NC_000072.6	Wipf3	1.86E-01
gene34884	219114	NC_000080.6	Ska3	1.86E-01
gene11807	78796	NC_000071.6	Zcchc4	1.86E-01
gene17772	243958	NC_000073.6	Siglecg	1.86E-01
gene23855	102502	NC_000075.6	Pls1	1.86E-01
gene9508	1E+08	NC_000070.6	Gm12610	1.86E-01
gene29807	380752	NC_000078.6	Tssc1	1.86E-01
gene12086	12617	NC_000071.6	Cenpc1	1.86E-01
gene32623	214639	NC_000079.6	4930486L24Rik	1.85E-01
gene1459	19258	NC_000067.6	Ptpn4	1.85E-01
gene1512	72585	NC_000067.6	Lypd1	1.85E-01
gene36261	13196	NC_000081.6	Asap1	1.85E-01
gene24355	11481	NC_000075.6	Acvr2b	1.85E-01
gene35322	211253	NC_000080.6	Mtrf1	1.85E-01
gene37448	239719	NC_000082.6	Mkl2	1.85E-01
gene38651	245305	NC_000082.6	B230307C23Rik	1.85E-01
gene38568	12394	NC_000082.6	Runx1	1.85E-01
gene6198	21400	NC_000068.7	Tcea2	1.85E-01
gene11984	100737	NC_000071.6	Dcun1d4	1.85E-01
gene7604	236539	NC_000069.6	Phgdh	1.85E-01
gene4972	241633	NC_000068.7	Atp8b4	1.85E-01
gene36825	223754	NC_000081.6	Tbc1d22a	1.85E-01
gene6267	170753	NC_000069.6	Zfp704	1.84E-01
gene27896	637515	NC_000077.6	Nlrp1b	1.84E-01
gene10183	269582	NC_000070.6	Clspn	1.84E-01
gene19451	18751	NC_000073.6	Prkcb	1.84E-01
gene34061	78754	NC_000080.6	Galnt15	1.84E-01
gene17381	233057	NC_000073.6	Zfp940	1.84E-01
gene7019	1E+08	NC_000069.6	Gm36569	1.84E-01
gene40468	381110	NC_000083.6	Rmdn2	1.84E-01
gene3784	14725	NC_000068.7	Lrp2	1.84E-01
gene29036	78455	NC_000077.6	Helz	1.84E-01
gene2619	76843	NC_000067.6	Dtl	1.83E-01
gene7301	242100	NC_000069.6	Pglyrp3	1.83E-01
gene8291	1.1E+08	NC_000069.6	Gm40166	1.83E-01
gene28384	207742	NC_000077.6	Rnf43	1.83E-01
gene13520	77521	NC_000071.6	Mtus2	1.83E-01
gene19271	233733	NC_000073.6	Galnt18	1.83E-01
gene9178	64817	NC_000070.6	Svep1	1.83E-01
gene39156	224617	NC_000083.6	Tbc1d24	1.83E-01
gene36495	93686	NC_000081.6	Rbfox2	1.83E-01
gene18801	22229	NC_000073.6	Ucp3	1.83E-01
gene22213	1E+08	NC_000074.6	Gm32856	1.83E-01
gene12196	231440	NC_000071.6	Parm1	1.82E-01
gene37953	320299	NC_000082.6	Iqcb1	1.82E-01
gene29926	17286	NC_000078.6	Meox2	1.82E-01

gene43085	226255	NC_000085.6	Atrnl1	1.82E-01
gene7115	170643	NC_000069.6	Kirrel	1.82E-01
gene12803	57816	NC_000071.6	Tesc	1.82E-01
gene12366	1E+08	NC_000071.6	5430416N02Rik	1.82E-01
gene19523	68616	NC_000073.6	Gdpd3	1.82E-01
gene18640	72759	NC_000073.6	Tmem135	1.82E-01
gene20261	11601	NC_000074.6	Angpt2	1.82E-01
gene15314	232288	NC_000072.6	Frmd4b	1.82E-01
gene2770	57743	NC_000068.7	Sec61a2	1.82E-01
gene42016	18426	NC_000085.6	Ovol1	1.82E-01
gene21695	68051	NC_000074.6	Nutf2	1.82E-01
gene42270	666926	NC_000085.6	Gm8369	1.82E-01
gene35010	12143	NC_000080.6	Blk	1.81E-01
gene635	68549	NC_000067.6	Sgol2a	1.81E-01
gene30365	110606	NC_000078.6	Fntb	1.81E-01
gene22701	235184	NC_000075.6	Msantd2	1.81E-01
gene25002	331623	NC_000076.6	Bend3	1.81E-01
gene37831	71911	NC_000082.6	Bdh1	1.81E-01
gene1718	67997	NC_000067.6	Ddx59	1.81E-01
gene2900	1E+08	NC_000068.7	Gm10851	1.81E-01
gene39421	224671	NC_000083.6	Btbd9	1.81E-01
gene1356	635504	NC_000067.6	Gm7160	1.81E-01
gene34938	70478	NC_000080.6	Mipep	1.81E-01
gene951	13838	NC_000067.6	Epha4	1.81E-01
gene11573	20537	NC_000071.6	Slc5a1	1.81E-01
gene24006	56318	NC_000075.6	Acpp	1.81E-01
gene33715	66082	NC_000080.6	Abhd6	1.80E-01
gene40542	78148	NC_000083.6	8430430B14Rik	1.80E-01
gene4470	1E+08	NC_000068.7	Gm32824	1.80E-01
gene28317	66196	NC_000077.6	Myo19	1.80E-01
gene17393	72556	NC_000073.6	Zfp566	1.80E-01
gene36496	74303	NC_000081.6	1700109K24Rik	1.80E-01
gene28298	21410	NC_000077.6	Hnf1b	1.80E-01
gene16173	1E+08	NC_000072.6	Gm35768	1.80E-01
gene24201	16578	NC_000075.6	Kif9	1.80E-01
gene38686	239985	NC_000083.6	Arid1b	1.80E-01
gene7891	229776	NC_000069.6	Cdc14a	1.80E-01
gene34775	239102	NC_000080.6	Zfhx2	1.80E-01
gene21450	26383	NC_000074.6	Fto	1.80E-01
gene37910	70757	NC_000082.6	Hacd2	1.79E-01
gene4439	192285	NC_000068.7	Phf21a	1.79E-01
gene26656	140703	NC_000077.6	Emid1	1.79E-01
gene33384	74559	NC_000079.6	Elovl7	1.79E-01
gene36154	97998	NC_000081.6	Deptor	1.79E-01
gene39920	56050	NC_000083.6	Cyp39a1	1.79E-01
gene34050	1E+08	NC_000080.6	Gm35907	1.78E-01
gene7826	53883	NC_000069.6	Celsr2	1.78E-01
gene5516	12396	NC_000068.7	Cbfa2t2	1.78E-01

gene24505	70337	NC_000076.6	Iyd	1.78E-01
gene22269	234912	NC_000075.6	9230110C19Rik	1.78E-01
gene13992	208164	NC_000072.6	Fam180a	1.78E-01
gene28058	1E+08	NC_000077.6	Gm12339	1.78E-01
gene42428	208518	NC_000085.6	Cep78	1.78E-01
gene19506	=Gene;ger	NC_000073.6	LOC105243053	1.78E-01
gene19295	11865	NC_000073.6	Arntl	1.78E-01
gene26769	75280	NC_000077.6	4930554G24Rik	1.77E-01
gene36953	78895	NC_000081.6	Pus7l	1.77E-01
gene41291	240261	NC_000084.6	Ccdc112	1.77E-01
gene9156	54357	NC_000070.6	Epb41l4b	1.77E-01
gene2041	18933	NC_000067.6	Prrx1	1.77E-01
gene41619	52662	NC_000084.6	Ldlrad4	1.77E-01
gene29452	668212	NC_000078.6	Efr3b	1.76E-01
gene30303	17420	NC_000078.6	Mnat1	1.76E-01
gene7661	1E+08	NC_000069.6	Gm32900	1.76E-01
gene9797	14084	NC_000070.6	Faf1	1.76E-01
gene36122	1E+08	NC_000081.6	Gm34678	1.76E-01
gene18630	50490	NC_000073.6	Nox4	1.76E-01
gene40303	320209	NC_000083.6	Ddx11	1.76E-01
gene224	68187	NC_000067.6	Fam135a	1.76E-01
gene37494	70620	NC_000082.6	Ube2v2	1.76E-01
gene12413	246293	NC_000071.6	Klhl8	1.76E-01
gene28306	56321	NC_000077.6	Aatf	1.76E-01
gene18651	319711	NC_000073.6	E230029C05Rik	1.76E-01
gene1067	15559	NC_000067.6	Htr2b	1.76E-01
gene29608	14245	NC_000078.6	Lpin1	1.75E-01
gene11105	77036	NC_000071.6	1700109H08Rik	1.75E-01
gene20991	73095	NC_000074.6	Slc25a42	1.75E-01
gene20027	233979	NC_000073.6	Tpcn2	1.75E-01
gene425	16178	NC_000067.6	Il1r2	1.75E-01
gene40551	213556	NC_000083.6	Plekhh2	1.75E-01
gene22270	234915	NC_000075.6	Cep126	1.75E-01
gene35702	ene=LOC1	NC_000081.6	LOC108168213	1.75E-01
gene30209	18974	NC_000078.6	Pole2	1.75E-01
gene40500	68027	NC_000083.6	Tmem178	1.75E-01
gene12834	21388	NC_000071.6	Tbx5	1.75E-01
gene31465	432720	NC_000079.6	Akr1c19	1.75E-01
gene15019	232156	NC_000072.6	Slc4a5	1.74E-01
gene24714	667281	NC_000076.6	H60b	1.74E-01
gene16766	243842	NC_000073.6	Gltscr1	1.74E-01
gene22256	1E+08	NC_000075.6	Gm31751	1.74E-01
gene15321	17342	NC_000072.6	Mitf	1.74E-01
gene8043	71481	NC_000069.6	Alpk1	1.74E-01
gene23084	330938	NC_000075.6	Dixdc1	1.74E-01
gene12052	69982	NC_000071.6	Spink2	1.74E-01
gene37486	66793	NC_000082.6	Efcab1	1.74E-01
gene11579	71101	NC_000071.6	Uvssa	1.74E-01

gene30881	78801	NC_000078.6	Ak7	1.74E-01
gene13715	1E+08	NC_000072.6	Umad1	1.74E-01
gene16119	665037	NC_000072.6	Gm7457	1.73E-01
gene38548	121022	NC_000082.6	Mrps6	1.73E-01
gene28352	56427	NC_000077.6	Tubd1	1.73E-01
gene17856	233199	NC_000073.6	Mybpc2	1.73E-01
gene23918	1E+08	NC_000075.6	Gm2773	1.73E-01
gene33040	67486	NC_000079.6	Polr3g	1.73E-01
gene41228	269016	NC_000084.6	Sh3rf2	1.73E-01
gene19150	19024	NC_000073.6	Ppfibp2	1.73E-01
gene29419	210004	NC_000077.6	B3gntl1	1.73E-01
gene23242	56380	NC_000075.6	Arid3b	1.72E-01
gene20478	76604	NC_000074.6	1700047A11Rik	1.72E-01
gene22916	58235	NC_000075.6	Nectin1	1.72E-01
gene29907	403205	NC_000078.6	Agr3	1.72E-01
gene12931	330189	NC_000071.6	Tmem120b	1.72E-01
gene8500	320790	NC_000070.6	Chd7	1.72E-01
gene29082	217262	NC_000077.6	Abca9	1.72E-01
gene40486	381112	NC_000083.6	Arhgef33	1.72E-01
gene11394	231051	NC_000071.6	Kmt2c	1.72E-01
gene379	72097	NC_000067.6	2010300C02Rik	1.72E-01
gene17429	333193	NC_000073.6	Proser3	1.72E-01
gene21831	319481	NC_000074.6	Wdr59	1.72E-01
gene39915	1E+08	NC_000083.6	Ankrd66	1.72E-01
gene1759	12316	NC_000067.6	Aspm	1.71E-01
gene25064	77987	NC_000076.6	Ascc3	1.71E-01
gene18841	14276	NC_000073.6	Folr2	1.71E-01
gene9437	1E+08	NC_000070.6	Gm33730	1.71E-01
gene38107	224170	NC_000082.6	Dzip3	1.71E-01
gene5096	140629	NC_000068.7	Ubox5	1.71E-01
gene24538	215748	NC_000076.6	Cnksr3	1.71E-01
gene27256	216725	NC_000077.6	Adamts2	1.71E-01
gene22467	18577	NC_000075.6	Pde4a	1.71E-01
gene39978	224814	NC_000083.6	Abcc10	1.70E-01
gene40312	19274	NC_000083.6	Ptprm	1.70E-01
gene26761	12808	NC_000077.6	Cobl	1.70E-01
gene18433	16576	NC_000073.6	Kif7	1.70E-01
gene3940	545428	NC_000068.7	Ccdc141	1.70E-01
gene25925	74454	NC_000076.6	4933408J17Rik	1.70E-01
gene26387	14632	NC_000076.6	Gli1	1.70E-01
gene2417	72568	NC_000067.6	Lin9	1.70E-01
gene5011	72180	NC_000068.7	Zfp661	1.70E-01
gene18266	272396	NC_000073.6	Tarsl2	1.70E-01
gene28080	237858	NC_000077.6	Tusc5	1.70E-01
gene38160	74201	NC_000082.6	Cep97	1.70E-01
gene33256	17755	NC_000079.6	Map1b	1.70E-01
gene13771	21426	NC_000072.6	Tfec	1.70E-01
gene34068	53600	NC_000080.6	Timm23	1.69E-01

gene19448	70261	NC_000073.6	Chp2	1.69E-01
gene15273	101401	NC_000072.6	Adamts9	1.69E-01
gene9685	108079	NC_000070.6	Prkaa2	1.69E-01
gene9549	67694	NC_000070.6	Ift74	1.69E-01
gene12762	75665	NC_000071.6	Ccdc64	1.69E-01
gene36870	69440	NC_000081.6	Dennd6b	1.69E-01
gene42682	240641	NC_000085.6	Kif20b	1.68E-01
gene3226	227674	NC_000068.7	Ddx31	1.68E-01
gene35064	219151	NC_000080.6	Scara3	1.68E-01
gene26977	20856	NC_000077.6	Stc2	1.68E-01
gene33444	268706	NC_000079.6	Slc38a9	1.68E-01
gene36794	73225	NC_000081.6	Fam118a	1.68E-01
gene30734	104884	NC_000078.6	Tdp1	1.68E-01
gene4402	Gene;gene	NC_000068.7	LOC102632821	1.68E-01
gene5815	319684	NC_000068.7	5031425F14Rik	1.68E-01
gene1796	64214	NC_000067.6	Rgs18	1.68E-01
gene40202	19725	NC_000083.6	Rfx2	1.68E-01
gene13471	245880	NC_000071.6	Wasf3	1.68E-01
gene41356	66758	NC_000084.6	Zfp474	1.67E-01
gene23612	214345	NC_000075.6	Lrrc1	1.67E-01
gene38508	207932	NC_000082.6	Urb1	1.67E-01
gene41546	104082	NC_000084.6	Wdr7	1.67E-01
gene3441	16874	NC_000068.7	Lhx6	1.67E-01
gene27914	74477	NC_000077.6	4933427D14Rik	1.67E-01
gene21897	72155	NC_000074.6	Cenpn	1.67E-01
gene633	67198	NC_000067.6	Spats2l	1.67E-01
gene37110	11419	NC_000081.6	Asic1	1.67E-01
gene43075	226250	NC_000085.6	Afap1l2	1.67E-01
gene40820	106931	NC_000084.6	Kctd1	1.67E-01
gene399	98388	NC_000067.6	Chst10	1.67E-01
gene28035	319822	NC_000077.6	Smyd4	1.67E-01
gene30718	319670	NC_000078.6	Eml5	1.67E-01
gene6816	70839	NC_000069.6	P2ry12	1.67E-01
gene34084	320736	NC_000080.6	Vstm4	1.67E-01
gene40113	72415	NC_000083.6	Sgol1	1.67E-01
gene1579	108900	NC_000067.6	Fam72a	1.66E-01
gene38636	56175	NC_000082.6	Bace2	1.66E-01
gene36849	223773	NC_000081.6	Zbed4	1.66E-01
gene35315	1E+08	NC_000080.6	Gm30970	1.66E-01
gene3933	99031	NC_000068.7	Osbpl6	1.66E-01
gene22684	12649	NC_000075.6	Chek1	1.66E-01
gene20670	70546	NC_000074.6	Zdhhc2	1.66E-01
gene4893	140476	NC_000068.7	Strc	1.66E-01
gene16394	213011	NC_000073.6	Zfp583	1.66E-01
gene27262	22678	NC_000077.6	Zfp2	1.66E-01
gene11909	245945	NC_000071.6	Rbm47	1.66E-01
gene29328	74370	NC_000077.6	Rptor	1.66E-01
gene18669	73845	NC_000073.6	Ankrd42	1.66E-01

gene41201	71302	NC_000084.6	Arhgap26	1.66E-01
gene16861	384569	NC_000073.6	Nova2	1.66E-01
gene37981	320214	NC_000082.6	Maats1	1.66E-01
gene15364	55983	NC_000072.6	Pdzrn3	1.66E-01
gene14175	76156	NC_000072.6	Fam131b	1.65E-01
gene11639	70292	NC_000071.6	Afap1	1.65E-01
gene7009	1E+08	NC_000069.6	Gm17359	1.65E-01
gene22983	399548	NC_000075.6	Scn4b	1.65E-01
gene23097	235345	NC_000075.6	4833427G06Rik	1.65E-01
gene16413	18616	NC_000073.6	Peg3	1.65E-01
gene39257	214931	NC_000083.6	Fbxl16	1.65E-01
gene13506	14255	NC_000071.6	Flt3	1.65E-01
gene21707	66369	NC_000074.6	Dus2	1.65E-01
gene23926	272636	NC_000075.6	Esyt3	1.65E-01
gene11968	21682	NC_000071.6	Tec	1.65E-01
gene3497	20744	NC_000068.7	Strbp	1.65E-01
gene5384	69270	NC_000068.7	Gins1	1.65E-01
gene36493	17189	NC_000081.6	Mb	1.65E-01
gene8198	56224	NC_000069.6	Tspan5	1.65E-01
gene392	56210	NC_000067.6	Rev1	1.65E-01
gene35241	271221	NC_000080.6	5031414D18Rik	1.64E-01
gene32412	1E+08	NC_000079.6	Gm32834	1.64E-01
gene28160	18099	NC_000077.6	Nlk	1.64E-01
gene11684	75079	NC_000071.6	Zbtb49	1.64E-01
gene7898	229780	NC_000069.6	Trmt13	1.64E-01
gene41683	66468	NC_000084.6	Ska1	1.64E-01
gene17320	80749	NC_000073.6	Lrfn1	1.64E-01
gene12449	675363	NC_000071.6	Gm9640	1.64E-01
gene9950	20441	NC_000070.6	St3gal3	1.64E-01
gene35308	380921	NC_000080.6	Dgkh	1.64E-01
gene27333	71774	NC_000077.6	Shroom1	1.64E-01
gene34076	319955	NC_000080.6	Ercc6	1.64E-01
gene22054	320869	NC_000074.6	Spata33	1.63E-01
gene6468	71862	NC_000069.6	Gpr160	1.63E-01
gene30507	217705	NC_000078.6	Fam161b	1.63E-01
gene5364	245174	NC_000068.7	Zfp937	1.63E-01
gene17978	211535	NC_000073.6	Ccdc114	1.63E-01
gene23105	1E+08	NC_000075.6	Gm32819	1.63E-01
gene33486	16398	NC_000079.6	Itga2	1.63E-01
gene14080	21577	NC_000072.6	Tcrb	1.63E-01
gene26963	75572	NC_000077.6	Acyp2	1.63E-01
gene14033	74253	NC_000072.6	Klrg2	1.63E-01
gene15484	68971	NC_000072.6	Tamm41	1.63E-01
gene22524	619310	NC_000075.6	Zfp872	1.63E-01
gene2361	76816	NC_000067.6	Sdccag8	1.62E-01
gene21314	23863	NC_000074.6	Dand5	1.62E-01
gene17886	78070	NC_000073.6	Cpt1c	1.62E-01
gene596	73980	NC_000067.6	4930444A19Rik	1.62E-01

gene39442	18585	NC_000083.6	Pde9a	1.62E-01
gene39633	17687	NC_000083.6	Msh5	1.62E-01
gene2097	18986	NC_000067.6	Pou2f1	1.62E-01
gene43046	53611	NC_000085.6	Vti1a	1.62E-01
gene10049	381549	NC_000070.6	Zfp69	1.62E-01
gene6165	73368	NC_000068.7	Col20a1	1.62E-01
gene34297	56291	NC_000080.6	Styx	1.62E-01
gene42218	269060	NC_000085.6	Dagla	1.61E-01
gene39309	16580	NC_000083.6	Kifc5b	1.61E-01
gene29835	1E+08	NC_000078.6	Gm32443	1.61E-01
gene10496	230848	NC_000070.6	Zbtb40	1.61E-01
gene10997	18762	NC_000070.6	Prkcz	1.61E-01
gene22228	69149	NC_000075.6	Kbtbd3	1.61E-01
gene15798	381812	NC_000072.6	Cracr2a	1.61E-01
gene39114	22654	NC_000083.6	Zfp13	1.60E-01
gene8231	229905	NC_000069.6	Kyat3	1.60E-01
gene14041	1.1E+08	NC_000072.6	Gm38688	1.60E-01
gene33373	67263	NC_000079.6	Zswim6	1.60E-01
gene37322	11515	NC_000082.6	Adcy9	1.60E-01
gene1023	98496	NC_000067.6	Pid1	1.60E-01
gene14055	209773	NC_000072.6	Dennd2a	1.60E-01
gene9770	230597	NC_000070.6	Zfyve9	1.60E-01
gene23051	235323	NC_000075.6	Usp28	1.60E-01
gene5780	629777	NC_000068.7	Zfp335os	1.60E-01
gene35589	58187	NC_000080.6	Cldn10	1.60E-01
gene32680	ene;gene=l	NC_000079.6	LOC108167344	1.60E-01
gene9545	67770	NC_000070.6	Caap1	1.60E-01
gene15049	26903	NC_000072.6	Dysf	1.60E-01
gene36112	83925	NC_000081.6	Trps1	1.60E-01
gene40460	70536	NC_000083.6	Qpct	1.59E-01
gene7699	54524	NC_000069.6	Syt6	1.59E-01
gene36178	387609	NC_000081.6	Zhx2	1.59E-01
gene29830	11431	NC_000078.6	Acp1	1.59E-01
gene30852	94040	NC_000078.6	Clmn	1.59E-01
gene9161	545622	NC_000070.6	Ptpn3	1.59E-01
gene24673	71804	NC_000076.6	Mtfr2	1.59E-01
gene23948	245020	NC_000075.6	Slc35g2	1.59E-01
gene18797	320452	NC_000073.6	P4ha3	1.59E-01
gene35592	66573	NC_000080.6	Dzip1	1.59E-01
gene19238	56788	NC_000073.6	Scube2	1.59E-01
gene911	241116	NC_000067.6	Ccdc108	1.59E-01
gene26790	380686	NC_000077.6	Cnrip1	1.59E-01
gene27501	216795	NC_000077.6	Wnt9a	1.58E-01
gene24797	237339	NC_000076.6	L3mbtl3	1.58E-01
gene15099	21802	NC_000072.6	Tgfa	1.58E-01
gene10249	320896	NC_000070.6	C330020E22Rik	1.58E-01
gene3320	108897	NC_000068.7	Aif1l	1.58E-01
gene27710	216835	NC_000077.6	Usp43	1.58E-01

gene33498	52552	NC_000079.6	Parp8	1.58E-01
gene2941	74455	NC_000068.7	Nsun6	1.57E-01
gene7094	229488	NC_000069.6	Fam160a1	1.57E-01
gene25386	71236	NC_000076.6	Rsph14	1.57E-01
gene24778	ene;gene=L	NC_000076.6	LOC108167801	1.57E-01
gene33121	73942	NC_000079.6	Fam151b	1.57E-01
gene1994	240869	NC_000067.6	Zbtb37	1.56E-01
gene32850	72958	NC_000079.6	Zfp493	1.56E-01
gene1363	12043	NC_000067.6	Bcl2	1.56E-01
gene11596	269642	NC_000071.6	Nat8l	1.56E-01
gene40035	17240	NC_000083.6	Mdfi	1.56E-01
gene34942	50720	NC_000080.6	Sacs	1.56E-01
gene24886	14302	NC_000076.6	Frk	1.56E-01
gene28199	237877	NC_000077.6	Atad5	1.56E-01
gene25758	58250	NC_000076.6	Chst11	1.56E-01
gene9913	108067	NC_000070.6	Eif2b3	1.56E-01
gene39486	106648	NC_000083.6	Cyp4f15	1.55E-01
gene23949	20842	NC_000075.6	Stag1	1.55E-01
gene29879	380753	NC_000078.6	Atxn7l1	1.55E-01
gene2726	104362	NC_000068.7	Meig1	1.55E-01
gene13243	381678	NC_000071.6	Zcwpw1	1.55E-01
gene867	=Gene;ger	NC_000067.6	LOC102633705	1.55E-01
gene9025	12819	NC_000070.6	Col15a1	1.55E-01
gene19646	1E+08	NC_000073.6	Gm33122	1.55E-01
gene36799	14114	NC_000081.6	Fbln1	1.55E-01
gene2248	93840	NC_000067.6	Vangl2	1.55E-01
gene41501	328968	NC_000084.6	Carmn	1.55E-01
gene28023	103677	NC_000077.6	Smg6	1.55E-01
gene23998	74025	NC_000075.6	Nphp3	1.55E-01
gene18151	11982	NC_000073.6	Atp10a	1.55E-01
gene25233	216019	NC_000076.6	Hkdc1	1.54E-01
gene25791	1E+08	NC_000076.6	Gm34633	1.54E-01
gene22180	212728	NC_000074.6	Gm17296	1.54E-01
gene12972	269702	NC_000071.6	Mphosph9	1.54E-01
gene34876	21821	NC_000080.6	Ift88	1.54E-01
gene5165	73747	NC_000068.7	1110034G24Rik	1.54E-01
gene43066	1E+08	NC_000085.6	Gm32441	1.54E-01
gene15857	1.1E+08	NC_000072.6	Gm38906	1.54E-01
gene31961	68732	NC_000079.6	Lrrc16a	1.54E-01
gene19595	56216	NC_000073.6	Stx1b	1.53E-01
gene40505	20541	NC_000083.6	Slc8a1	1.53E-01
gene30853	212073	NC_000078.6	Syne3	1.53E-01
gene16172	16439	NC_000072.6	Itpr2	1.53E-01
gene4590	211896	NC_000068.7	Depdc7	1.53E-01
gene9612	242557	NC_000070.6	Atg4c	1.53E-01
gene21491	68219	NC_000074.6	Nudt21	1.53E-01
gene12493	21814	NC_000071.6	Tgfbr3	1.53E-01
gene3397	1E+08	NC_000068.7	Gm34178	1.53E-01

gene3670	11477	NC_000068.7	Acvr1	1.53E-01
gene41277	240255	NC_000084.6	Ythdc2	1.53E-01
gene35723	268780	NC_000081.6	Egflam	1.52E-01
gene15031	1E+08	NC_000072.6	Gm21284	1.52E-01
gene26585	1E+08	NC_000077.6	Gm11944	1.52E-01
gene30284	67217	NC_000078.6	L3hypdh	1.52E-01
gene40561	20532	NC_000083.6	Slc3a1	1.52E-01
gene35313	219189	NC_000080.6	Vwa8	1.52E-01
gene35321	66897	NC_000080.6	Naa16	1.52E-01
gene42533	107351	NC_000085.6	Kank1	1.52E-01
gene36779	109270	NC_000081.6	Prr5	1.52E-01
gene2492	21339	NC_000067.6	Taf1a	1.52E-01
gene38488	21844	NC_000082.6	Tiam1	1.51E-01
gene37773	16180	NC_000082.6	Il1rap	1.51E-01
gene18675	74041	NC_000073.6	Ddias	1.51E-01
gene43063	226245	NC_000085.6	Plekhs1	1.51E-01
gene40026	224836	NC_000083.6	Usp49	1.51E-01
gene40469	1E+08	NC_000083.6	Gm33006	1.51E-01
gene35584	239273	NC_000080.6	Abcc4	1.51E-01
gene17392	330502	NC_000073.6	Zfp82	1.51E-01
gene19956	320802	NC_000073.6	Ifitm10	1.51E-01
gene39479	208285	NC_000083.6	Cyp4f17	1.51E-01
gene18513	101592	NC_000073.6	Efl1	1.51E-01
gene9604	242553	NC_000070.6	Kank4	1.51E-01
gene27281	193286	NC_000077.6	BC049762	1.51E-01
gene8994	74152	NC_000070.6	Stra6l	1.50E-01
gene38168	66315	NC_000082.6	Senp7	1.50E-01
gene32039	13557	NC_000079.6	E2f3	1.50E-01
gene29685	382686	NC_000078.6	3110053B16Rik	1.50E-01
gene7897	109245	NC_000069.6	Lrrc39	1.50E-01
gene5368	1E+08	NC_000068.7	Gm10130	1.50E-01
gene3773	1E+08	NC_000068.7	Gm36231	1.50E-01
gene13565	381694	NC_000071.6	B3glct	1.50E-01
gene31966	12763	NC_000079.6	Cmah	1.50E-01
gene10611	230872	NC_000070.6	Crocc	1.50E-01
gene6908	72789	NC_000069.6	Veph1	1.50E-01
gene37519	50525	NC_000082.6	Spag6l	1.50E-01
gene35783	18162	NC_000081.6	Npr3	1.49E-01
gene30720	76260	NC_000078.6	Ttc8	1.49E-01
gene25083	66686	NC_000076.6	Dcbld1	1.49E-01
gene6256	66230	NC_000069.6	Mrps28	1.49E-01
gene25732	408062	NC_000076.6	Zfp873	1.49E-01
gene1275	98314	NC_000067.6	D2hgdh	1.49E-01
gene36893	=Gene;ger	NC_000081.6	LOC105246018	1.49E-01
gene8729	24018	NC_000070.6	Rngtt	1.49E-01
gene42748	209478	NC_000085.6	Tbc1d12	1.49E-01
gene31045	104859	NC_000078.6	Tecpr2	1.48E-01
gene10042	18046	NC_000070.6	Nfyc	1.48E-01

gene15403	1E+08	NC_000072.6	Gm35165	1.48E-01
gene7092	229487	NC_000069.6	Gatb	1.48E-01
gene11731	231214	NC_000071.6	Cc2d2a	1.48E-01
gene7415	66353	NC_000069.6	Riiad1	1.48E-01
gene29320	268512	NC_000077.6	Slc26a11	1.48E-01
gene12039	231326	NC_000071.6	Aasdh	1.48E-01
gene555	20849	NC_000067.6	Stat4	1.48E-01
gene36640	213988	NC_000081.6	Tnrc6b	1.48E-01
gene34053	382864	NC_000080.6	Colq	1.48E-01
gene15495	381802	NC_000072.6	Tsen2	1.48E-01
gene43082	72133	NC_000085.6	Trub1	1.48E-01
gene32233	218194	NC_000079.6	Phactr1	1.48E-01
gene6639	73852	NC_000069.6	D3Ertd751e	1.48E-01
gene23988	1E+08	NC_000075.6	Gm16252	1.48E-01
gene26998	16199	NC_000077.6	Il9r	1.47E-01
gene41363	271521	NC_000084.6	Gm1859	1.47E-01
gene41496	328967	NC_000084.6	Arhgef37	1.47E-01
gene29689	211914	NC_000078.6	Asap2	1.47E-01
gene37685	209018	NC_000082.6	Vps8	1.47E-01
gene22869	54698	NC_000075.6	Crtam	1.47E-01
gene38128	70508	NC_000082.6	Bbx	1.47E-01
gene5886	18019	NC_000068.7	Nfatc2	1.47E-01
gene37318	12914	NC_000082.6	Crebbp	1.47E-01
gene12489	12545	NC_000071.6	Cdc7	1.47E-01
gene12874	269700	NC_000071.6	Gm15800	1.47E-01
gene42913	12234	NC_000085.6	Btrc	1.46E-01
gene11485	381628	NC_000071.6	Adgrf3	1.46E-01
gene24908	66337	NC_000076.6	Fam229b	1.46E-01
gene42680	75735	NC_000085.6	Pank1	1.46E-01
gene35995	52589	NC_000081.6	Ncald	1.46E-01
gene23510	15450	NC_000075.6	Lipc	1.46E-01
gene7995	59006	NC_000069.6	Myoz2	1.46E-01
gene42774	20411	NC_000085.6	Sorbs1	1.46E-01
gene33138	26556	NC_000079.6	Homer1	1.46E-01
gene5053	1.1E+08	NC_000068.7	Gm39932	1.46E-01
gene13112	57080	NC_000071.6	Gtf2ird1	1.46E-01
gene37150	109901	NC_000081.6	Cela1	1.46E-01
gene1037	381287	NC_000067.6	A530032D15Rik	1.45E-01
gene30657	72113	NC_000078.6	Adck1	1.45E-01
gene37698	319765	NC_000082.6	Igf2bp2	1.45E-01
gene24854	268291	NC_000076.6	Rnf217	1.45E-01
gene15725	1.1E+08	NC_000072.6	Gm40398	1.45E-01
gene25389	76877	NC_000076.6	Rab36	1.45E-01
gene22048	57429	NC_000074.6	Sult5a1	1.45E-01
gene2024	1E+08	NC_000067.6	Gm31985	1.45E-01
gene11630	242939	NC_000071.6	Cpz	1.45E-01
gene41601	1E+08	NC_000084.6	Gm30321	1.45E-01
gene27056	=Gene;ger	NC_000077.6	LOC108167896	1.44E-01

gene22578	83964	NC_000075.6	Jam3	1.44E-01
gene37485	1E+08	NC_000082.6	Gm33696	1.44E-01
gene37802	71528	NC_000082.6	9030404E10Rik	1.44E-01
gene14026	264134	NC_000072.6	Ttc26	1.44E-01
gene7942	1E+08	NC_000069.6	Gm33363	1.44E-01
gene28937	399609	NC_000077.6	C130046K22Rik	1.44E-01
gene8284	69219	NC_000069.6	Ddah1	1.44E-01
gene23554	72135	NC_000075.6	Pygo1	1.43E-01
gene9598	12695	NC_000070.6	Inadl	1.43E-01
gene4545	210622	NC_000068.7	Pamr1	1.43E-01
gene29180	170930	NC_000077.6	Sumo2	1.43E-01
gene15177	319655	NC_000072.6	Podxl2	1.43E-01
gene32814	78251	NC_000079.6	Zfp712	1.43E-01
gene23128	77591	NC_000075.6	Ddx10	1.43E-01
gene27712	55943	NC_000077.6	Stx8	1.43E-01
gene18219	1E+08	NC_000073.6	Gm32061	1.43E-01
gene25217	404634	NC_000076.6	H2afy2	1.43E-01
gene18474	1E+08	NC_000073.6	Gm32178	1.43E-01
gene38654	320586	NC_000082.6	A630089N07Rik	1.43E-01
gene21616	12354	NC_000074.6	Car7	1.43E-01
gene32676	211378	NC_000079.6	6720489N17Rik	1.43E-01
gene9043	16348	NC_000070.6	Invs	1.42E-01
gene34088	67580	NC_000080.6	Lrrc18	1.42E-01
gene5524	228807	NC_000068.7	Zfp341	1.42E-01
gene12505	170823	NC_000071.6	Glmn	1.41E-01
gene29235	1E+08	NC_000077.6	Gm11744	1.41E-01
gene37016	1E+08	NC_000081.6	Gm33636	1.41E-01
gene8910	22249	NC_000070.6	Unc13b	1.41E-01
gene35339	380924	NC_000080.6	Olfm4	1.41E-01
gene7730	22414	NC_000069.6	Wnt2b	1.41E-01
gene30659	74926	NC_000078.6	4930473H19Rik	1.41E-01
gene35734	73692	NC_000081.6	2410089E03Rik	1.40E-01
gene8121	214133	NC_000069.6	Tet2	1.40E-01
gene32684	380850	NC_000079.6	Gm5141	1.40E-01
gene23577	235493	NC_000075.6	Fam214a	1.40E-01
gene29159	328035	NC_000077.6	Fads6	1.40E-01
gene23478	1E+08	NC_000075.6	Gm15511	1.40E-01
gene18514	77800	NC_000073.6	4933406J10Rik	1.40E-01
gene41648	80718	NC_000084.6	Rab27b	1.40E-01
gene6920	109222	NC_000069.6	Rarres1	1.40E-01
gene12597	18973	NC_000071.6	Pole	1.39E-01
gene34982	319433	NC_000080.6	Serpine3	1.39E-01
gene35875	223435	NC_000081.6	Trio	1.39E-01
gene30224	217666	NC_000078.6	L2hgdh	1.39E-01
gene2557	67769	NC_000067.6	Gpatch2	1.39E-01
gene23342	235431	NC_000075.6	Coro2b	1.39E-01
gene35415	77744	NC_000080.6	Bora	1.39E-01
gene931	11790	NC_000067.6	Speg	1.39E-01

gene372	269180	NC_000067.6	Inpp4a	1.38E-01
gene11819	19664	NC_000071.6	Rbpj	1.38E-01
gene35994	252973	NC_000081.6	Grhl2	1.38E-01
gene22528	235048	NC_000075.6	Zfp599	1.38E-01
gene13557	75860	NC_000071.6	Tex26	1.38E-01
gene23138	270160	NC_000075.6	Rab39	1.38E-01
gene22698	72927	NC_000075.6	Hepacam	1.38E-01
gene43105	226265	NC_000085.6	Eno4	1.38E-01
gene41559	54670	NC_000084.6	Atp8b1	1.38E-01
gene7662	78908	NC_000069.6	Igsf3	1.38E-01
gene926	19275	NC_000067.6	Ptprn	1.38E-01
gene36481	223666	NC_000081.6	Arhgap39	1.38E-01
gene38041	212517	NC_000082.6	Cfap44	1.38E-01
gene23608	26944	NC_000075.6	Tinag	1.38E-01
gene9914	19207	NC_000070.6	Ptch2	1.38E-01
gene38748	1E+08	NC_000083.6	Gm1604a	1.38E-01
gene35142	94352	NC_000080.6	Loxl2	1.37E-01
gene11837	319216	NC_000071.6	4932441J04Rik	1.37E-01
gene31644	27052	NC_000079.6	Aoah	1.37E-01
gene14579	73998	NC_000072.6	Herc3	1.37E-01
gene23876	235534	NC_000075.6	Pxylp1	1.37E-01
gene33284	26886	NC_000079.6	Cenph	1.37E-01
gene35465	1E+08	NC_000080.6	Gm35197	1.37E-01
gene40075	76441	NC_000083.6	Daam2	1.36E-01
gene25408	1.1E+08	NC_000076.6	Gm40696	1.36E-01
gene6744	12427	NC_000069.6	Ccna1	1.36E-01
gene29954	246196	NC_000078.6	Zfp277	1.36E-01
gene19485	233865	NC_000073.6	D430042O09Rik	1.36E-01
gene4818	76464	NC_000068.7	Casc5	1.36E-01
gene4970	14178	NC_000068.7	Fgf7	1.36E-01
gene15590	67200	NC_000072.6	Ccdc77	1.36E-01
gene33007	105377	NC_000079.6	Slf1	1.36E-01
gene39921	1.1E+08	NC_000083.6	Gm41581	1.36E-01
gene41313	20358	NC_000084.6	Sema6a	1.36E-01
gene2802	209361	NC_000068.7	Taf3	1.36E-01
gene37143	21422	NC_000081.6	Tfcp2	1.36E-01
gene1015	80721	NC_000067.6	Slc19a3	1.36E-01
gene25116	544696	NC_000076.6	Tbc1d32	1.36E-01
gene21119	=Gene;ger	NC_000074.6	LOC108167537	1.36E-01
gene31968	193385	NC_000079.6	Fam65b	1.36E-01
gene1163	347722	NC_000067.6	Agap1	1.36E-01
gene10165	1E+08	NC_000070.6	Gm32908	1.36E-01
gene1695	215690	NC_000067.6	Nav1	1.36E-01
gene14577	26357	NC_000072.6	Abcg2	1.36E-01
gene41608	68166	NC_000084.6	Spire1	1.35E-01
gene15582	111173	NC_000072.6	Erc1	1.35E-01
gene28182	268451	NC_000077.6	Rab11fip4	1.35E-01
gene3028	67121	NC_000068.7	Mastl	1.35E-01

gene2055	20339	NC_000067.6	Sele	1.35E-01
gene22061	234857	NC_000074.6	Spire2	1.35E-01
gene36161	105837	NC_000081.6	Mtbp	1.35E-01
gene30387	27260	NC_000078.6	Plek2	1.35E-01
gene9927	=Gene;ger	NC_000070.6	LOC102637763	1.35E-01
gene30085	66132	NC_000078.6	1110008L16Rik	1.35E-01
gene37335	78809	NC_000082.6	4930562C15Rik	1.34E-01
gene9664	242584	NC_000070.6	Wdr78	1.34E-01
gene26317	319468	NC_000076.6	Ppm1h	1.34E-01
gene13735	75725	NC_000072.6	Phf14	1.34E-01
gene32041	218121	NC_000079.6	Mboat1	1.34E-01
gene30700	399558	NC_000078.6	Flrt2	1.34E-01
gene1518	107895	NC_000067.6	Mgat5	1.34E-01
gene35022	72400	NC_000080.6	Pinx1	1.34E-01
gene30367	=Gene;ger	NC_000078.6	LOC108168009	1.34E-01
gene5238	16558	NC_000068.7	Kif16b	1.34E-01
gene42840	67573	NC_000085.6	Loxl4	1.34E-01
gene32823	238690	NC_000079.6	Zfp458	1.34E-01
gene33757	238939	NC_000080.6	Gm281	1.34E-01
gene6241	16196	NC_000069.6	Il7	1.34E-01
gene18636	72433	NC_000073.6	Rab38	1.34E-01
gene42263	666907	NC_000085.6	Ms4a4a	1.34E-01
gene22317	330890	NC_000075.6	Piwil4	1.33E-01
gene30675	108800	NC_000078.6	Ston2	1.33E-01
gene24789	73803	NC_000076.6	4930401C15Rik	1.33E-01
gene5669	320799	NC_000068.7	Zhx3	1.33E-01
gene23301	102747	NC_000075.6	Lrrc49	1.33E-01
gene10758	70005	NC_000070.6	Znf41-ps	1.33E-01
gene7411	72634	NC_000069.6	Tdrkh	1.33E-01
gene26471	20431	NC_000076.6	Pmel	1.33E-01
gene9287	1.1E+08	NC_000070.6	Gm42295	1.33E-01
gene2269	93842	NC_000067.6	Igsf9	1.33E-01
gene8714	100019	NC_000070.6	Mdn1	1.33E-01
gene32678	1E+08	NC_000079.6	Gm36298	1.33E-01
gene13650	21789	NC_000072.6	Tfpi2	1.33E-01
gene43068	1E+08	NC_000085.6	Gm32575	1.33E-01
gene28063	=Gene;ger	NC_000077.6	LOC108167849	1.32E-01
gene33114	19418	NC_000079.6	Rasgrf2	1.32E-01
gene25137	18768	NC_000076.6	Pkib	1.32E-01
gene8097	74442	NC_000069.6	Sgms2	1.32E-01
gene39434	LOC10263	NC_000083.6	LOC102631757	1.32E-01
gene6156	228993	NC_000068.7	Slc17a9	1.32E-01
gene20899	=Gene;ger	NC_000074.6	LOC108167532	1.32E-01
gene17688	70227	NC_000073.6	Zfp619	1.32E-01
gene10316	19273	NC_000070.6	Ptpru	1.32E-01
gene28302	217031	NC_000077.6	Tada2a	1.32E-01
gene68	72481	NC_000067.6	2610203C22Rik	1.32E-01
gene27348	622459	NC_000077.6	Gm12216	1.31E-01

gene15141	72103	NC_000072.6	Aplf	1.31E-01
gene13237	1E+08	NC_000071.6	Gm36403	1.31E-01
gene9710	230576	NC_000070.6	Ttc22	1.31E-01
gene28090	319634	NC_000077.6	Efcab5	1.31E-01
gene42745	74055	NC_000085.6	Plce1	1.31E-01
gene24770	18605	NC_000076.6	Enpp1	1.31E-01
gene19793	76612	NC_000073.6	Lrrc27	1.31E-01
gene28149	20500	NC_000077.6	Slc13a2	1.31E-01
gene21665	277973	NC_000074.6	Slc9a5	1.31E-01
gene40138	72836	NC_000083.6	Pot1b	1.31E-01
gene36606	1E+08	NC_000081.6	Gm30977	1.31E-01
gene10000	27028	NC_000070.6	Ermap	1.31E-01
gene16393	384763	NC_000073.6	Zfp667	1.31E-01
gene4462	1E+08	NC_000068.7	Prdm11	1.31E-01
gene8345	19220	NC_000069.6	Ptgfr	1.30E-01
gene12835	433945	NC_000071.6	Gm5563	1.30E-01
gene27274	21408	NC_000077.6	Zfp354a	1.30E-01
gene2684	12902	NC_000067.6	Cr2	1.30E-01
gene27012	69306	NC_000077.6	Efcab9	1.30E-01
gene16262	245126	NC_000073.6	Tarm1	1.30E-01
gene41705	69190	NC_000084.6	Dym	1.30E-01
gene24949	70503	NC_000076.6	Ddo	1.30E-01
gene35094	108912	NC_000080.6	Cdca2	1.30E-01
gene18296	1E+08	NC_000073.6	Gm34079	1.30E-01
gene10031	329934	NC_000070.6	Foxo6	1.30E-01
gene25168	215999	NC_000076.6	Mcu	1.30E-01
gene42223	67752	NC_000085.6	Ppp1r32	1.30E-01
gene40777	63955	NC_000084.6	Cables1	1.30E-01
gene20868	18004	NC_000074.6	Nek1	1.30E-01
gene38177	56277	NC_000082.6	Tmem45a	1.30E-01
gene28833	12189	NC_000077.6	Brca1	1.30E-01
gene20640	414128	NC_000074.6	G630064G18Rik	1.30E-01
gene15567	232339	NC_000072.6	Ankrd26	1.30E-01
gene28059	380714	NC_000077.6	Rph3al	1.29E-01
gene37842	239827	NC_000082.6	Pigz	1.29E-01
gene33169	218461	NC_000079.6	Pde8b	1.29E-01
gene32423	68203	NC_000079.6	Diras2	1.29E-01
gene23555	67685	NC_000075.6	Dyx1c1	1.29E-01
gene23853	22063	NC_000075.6	Trpc1	1.29E-01
gene32714	218294	NC_000079.6	Cdc14b	1.29E-01
gene23379	68014	NC_000075.6	Zwilch	1.29E-01
gene20563	104271	NC_000074.6	Tex15	1.29E-01
gene430	17082	NC_000067.6	Il1rl1	1.29E-01
gene8961	17279	NC_000070.6	Melk	1.29E-01
gene23283	73893	NC_000075.6	Tmem202	1.29E-01
gene39314	240057	NC_000083.6	Syngap1	1.29E-01
gene8397	209584	NC_000069.6	Tyw3	1.29E-01
gene29956	238130	NC_000078.6	Dock4	1.29E-01

gene18462	12144	NC_000073.6	Blm	1.28E-01
gene41563	83814	NC_000084.6	Nedd4l	1.28E-01
gene41577	328977	NC_000084.6	Zfp532	1.28E-01
gene19832	244233	NC_000073.6	Cd163l1	1.28E-01
gene34902	628693	NC_000080.6	Gm6904	1.28E-01
gene38855	21646	NC_000083.6	Tcte2	1.28E-01
gene33179	544963	NC_000079.6	Iqgap2	1.28E-01
gene5511	228802	NC_000068.7	Bpifb5	1.28E-01
gene18772	101488	NC_000073.6	Slco2b1	1.28E-01
gene7779	229699	NC_000069.6	Slc16a4	1.28E-01
gene32466	319719	NC_000079.6	Simc1	1.28E-01
gene36167	15117	NC_000081.6	Has2	1.28E-01
gene5045	78751	NC_000068.7	Zc3h6	1.27E-01
gene32460	15466	NC_000079.6	Hrh2	1.27E-01
gene23045	18113	NC_000075.6	Nnmt	1.27E-01
gene13887	68794	NC_000072.6	Flnc	1.27E-01
gene37875	1E+08	NC_000082.6	Gm34825	1.27E-01
gene11314	80979	NC_000071.6	Slc26a5	1.27E-01
gene5525	=Gene;ger	NC_000068.7	LOC102635682	1.26E-01
gene21447	244585	NC_000074.6	Rpgrip1l	1.26E-01
gene29542	209334	NC_000078.6	Gen1	1.26E-01
gene13182	1.1E+08	NC_000071.6	Gm16599	1.26E-01
gene29080	27404	NC_000077.6	Abca8b	1.26E-01
gene23163	235386	NC_000075.6	Hykk	1.26E-01
gene35303	21943	NC_000080.6	Tnfsf11	1.26E-01
gene23781	107885	NC_000075.6	Mthfs	1.26E-01
gene10980	242800	NC_000070.6	Ttc34	1.26E-01
gene12928	74890	NC_000071.6	Morn3	1.26E-01
gene33461	1.1E+08	NC_000079.6	Gm41069	1.25E-01
gene10603	18600	NC_000070.6	Padi2	1.25E-01
gene24451	209737	NC_000075.6	Kif15	1.25E-01
gene21478	13897	NC_000074.6	Ces1e	1.25E-01
gene13319	243312	NC_000071.6	Elfn1	1.25E-01
gene4437	1E+08	NC_000068.7	Gm31473	1.25E-01
gene5109	1E+08	NC_000068.7	Gm30286	1.25E-01
gene40759	106861	NC_000084.6	Abhd3	1.25E-01
gene40985	402767	NC_000084.6	A830052D11Rik	1.25E-01
gene11602	1E+08	NC_000071.6	Cfap99	1.25E-01
gene37952	106389	NC_000082.6	Eaf2	1.25E-01
gene15268	243548	NC_000072.6	Prickle2	1.25E-01
gene2637	1E+08	NC_000067.6	Gm10516	1.25E-01
gene36066	11600	NC_000081.6	Angpt1	1.25E-01
gene13886	232664	NC_000072.6	Ccdc136	1.25E-01
gene38645	114604	NC_000082.6	Prdm15	1.25E-01
gene24875	692132	NC_000076.6	Trappc3l	1.25E-01
gene12613	50883	NC_000071.6	Chek2	1.25E-01
gene21846	30947	NC_000074.6	Adat1	1.24E-01
gene24208	19228	NC_000075.6	Pth1r	1.24E-01

gene40868	1E+08	NC_000084.6	Gm16090	1.24E-01
gene38817	104103	NC_000083.6	Airn	1.24E-01
gene39343	30051	NC_000083.6	Spdef	1.24E-01
gene35936	67724	NC_000081.6	Pop1	1.24E-01
gene23709	22137	NC_000075.6	Ttk	1.24E-01
gene11493	1E+08	NC_000071.6	Gm9899	1.24E-01
gene34114	75698	NC_000080.6	Fam35a	1.24E-01
gene27711	71860	NC_000077.6	Cfap52	1.24E-01
gene10845	17175	NC_000070.6	Masp2	1.24E-01
gene15563	72720	NC_000072.6	Zfp248	1.23E-01
gene43016	381232	NC_000085.6	Mirt1	1.23E-01
gene42958	94219	NC_000085.6	Cnnm2	1.23E-01
gene38736	20112	NC_000083.6	Rps6ka2	1.23E-01
gene12506	231571	NC_000071.6	Rpap2	1.23E-01
gene2761	227541	NC_000068.7	Camk1d	1.23E-01
gene20296	624198	NC_000074.6	Gm6483	1.23E-01
gene11761	209707	NC_000071.6	Lcorl	1.23E-01
gene16081	109135	NC_000072.6	Plekha5	1.23E-01
gene36487	239546	NC_000081.6	Zfp647	1.23E-01
gene15332	108655	NC_000072.6	Foxp1	1.23E-01
gene16305	21955	NC_000073.6	Tnnt1	1.23E-01
gene16787	1E+08	NC_000073.6	Gm29443	1.23E-01
gene24966	73075	NC_000076.6	Ppil6	1.23E-01
gene31932	319179	NC_000079.6	Hist1h2be	1.22E-01
gene31394	217935	NC_000078.6	Wdr60	1.22E-01
gene3735	=Gene;ger	NC_000068.7	LOC108168763	1.22E-01
gene6649	1E+08	NC_000069.6	Gm32340	1.22E-01
gene1692	93689	NC_000067.6	Lmod1	1.22E-01
gene26973	65257	NC_000077.6	Asb3	1.22E-01
gene18706	233529	NC_000073.6	Kctd14	1.22E-01
gene30715	104871	NC_000078.6	Spata7	1.22E-01
gene23956	235542	NC_000075.6	Ppp2r3a	1.22E-01
gene37003	239647	NC_000081.6	Pced1b	1.22E-01
gene26339	320398	NC_000076.6	Lrig3	1.22E-01
gene8116	67553	NC_000069.6	Gstcd	1.22E-01
gene3993	69482	NC_000068.7	Nup35	1.22E-01
gene32334	1.1E+08	NC_000079.6	Gm40943	1.22E-01
gene32831	328274	NC_000079.6	Zfp459	1.21E-01
gene17114	57745	NC_000073.6	Zfp112	1.21E-01
gene29831	24057	NC_000078.6	Sh3yl1	1.21E-01
gene42689	15566	NC_000085.6	Htr7	1.21E-01
gene15241	14115	NC_000072.6	Fbln2	1.21E-01
gene42660	70166	NC_000085.6	Lipn	1.21E-01
gene7996	118449	NC_000069.6	Synpo2	1.21E-01
gene7162	76927	NC_000069.6	Tsacc	1.21E-01
gene6330	ein beta 1-l	NC_000069.6	LOC100038947	1.21E-01
gene22243	71785	NC_000075.6	Pdgfd	1.21E-01
gene11359	16511	NC_000071.6	Kcnh2	1.21E-01

gene33218	110596	NC_000079.6	Arhgef28	1.21E-01
gene1248	16560	NC_000067.6	Kif1a	1.21E-01
gene36	620393	NC_000067.6	Fam150a	1.21E-01
gene25096	1E+08	NC_000076.6	Cep85l	1.20E-01
gene26735	626870	NC_000077.6	Gm11992	1.20E-01
gene40656	1E+08	NC_000083.6	Gm1976	1.20E-01
gene28097	268445	NC_000077.6	Ankrd13b	1.20E-01
gene24345	320256	NC_000075.6	Dlec1	1.20E-01
gene21120	73316	NC_000074.6	Calr3	1.20E-01
gene9660	1E+08	NC_000070.6	Gm12709	1.20E-01
gene13590	243362	NC_000071.6	Stard13	1.20E-01
gene19712	76429	NC_000073.6	Lhpp	1.20E-01
gene17391	243906	NC_000073.6	Zfp14	1.20E-01
gene38017	14432	NC_000082.6	Gap43	1.20E-01
gene6383	12873	NC_000069.6	Cpa3	1.20E-01
gene24814	210757	NC_000076.6	Themis	1.20E-01
gene29878	320046	NC_000078.6	F730043M19Rik	1.19E-01
gene11559	1E+08	NC_000071.6	Gm36840	1.19E-01
gene9110	319636	NC_000070.6	Fsd1l	1.19E-01
gene15123	269774	NC_000072.6	Aak1	1.19E-01
gene23769	319673	NC_000075.6	9330159M07Rik	1.19E-01
gene34354	93834	NC_000080.6	Peli2	1.19E-01
gene11729	109323	NC_000071.6	C1qtnf7	1.19E-01
gene19740	330662	NC_000073.6	Dock1	1.19E-01
gene32827	629016	NC_000079.6	Zfp953	1.19E-01
gene896	269209	NC_000067.6	Stk36	1.19E-01
gene40270	14158	NC_000083.6	Fer	1.19E-01
gene11641	81840	NC_000071.6	Sorcs2	1.19E-01
gene28031	237847	NC_000077.6	Rtn4rl1	1.18E-01
gene13130	71304	NC_000071.6	Wbscr25	1.18E-01
gene11287	68770	NC_000071.6	Phtf2	1.18E-01
gene15521	11689	NC_000072.6	Alox5	1.18E-01
gene36804	22422	NC_000081.6	Wnt7b	1.18E-01
gene25043	209462	NC_000076.6	Hace1	1.18E-01
gene9743	16975	NC_000070.6	Lrp8	1.18E-01
gene24813	19272	NC_000076.6	Ptprk	1.18E-01
gene25759	338365	NC_000076.6	Slc41a2	1.18E-01
gene21183	76775	NC_000074.6	Slc10a7	1.18E-01
gene30142	338320	NC_000078.6	Mia2	1.18E-01
gene84	211660	NC_000067.6	Cspp1	1.18E-01
gene41540	1E+08	NC_000084.6	Gm36651	1.18E-01
gene37547	72168	NC_000082.6	Aifm3	1.17E-01
gene3859	66935	NC_000068.7	Cir1	1.17E-01
gene2538	226791	NC_000067.6	Lyplal1	1.17E-01
gene33336	60411	NC_000079.6	Cenpk	1.17E-01
gene22853	80902	NC_000075.6	Zfp202	1.17E-01
gene29978	319504	NC_000078.6	Nrcam	1.17E-01
gene27479	ene;gene=L	NC_000077.6	LOC108167848	1.17E-01

gene34090	239027	NC_000080.6	Arhgap22	1.17E-01
gene3142	329366	NC_000068.7	Ccdc187	1.17E-01
gene19019	434219	NC_000073.6	Trim30c	1.17E-01
gene8111	271981	NC_000069.6	Tbck	1.17E-01
gene6949	242083	NC_000069.6	Ppm1l	1.17E-01
gene42677	240638	NC_000085.6	Slc16a12	1.16E-01
gene10180	214150	NC_000070.6	ago-03	1.16E-01
gene20896	12876	NC_000074.6	Cpe	1.16E-01
gene20947	211134	NC_000074.6	Lzts1	1.16E-01
gene28086	15567	NC_000077.6	Slc6a4	1.16E-01
gene36352	1E+08	NC_000081.6	Gm32994	1.16E-01
gene13668	243725	NC_000072.6	Ppp1r9a	1.16E-01
gene1673	71001	NC_000067.6	Mgat4e	1.16E-01
gene45467	22290	NC_000087.7	Uty	1.16E-01
gene29755	1E+08	NC_000078.6	Gm36129	1.16E-01
gene36912	16564	NC_000081.6	Kif21a	1.16E-01
gene31685	319207	NC_000079.6	Pgbd1	1.16E-01
gene28010	380711	NC_000077.6	Rap1gap2	1.16E-01
gene38593	110948	NC_000082.6	Hlcs	1.16E-01
gene23867	1E+08	NC_000075.6	Gm28729	1.15E-01
gene21950	382034	NC_000074.6	Gse1	1.15E-01
gene40261	50758	NC_000083.6	Fbxl17	1.15E-01
gene1029	66153	NC_000067.6	Fbxo36	1.15E-01
gene40491	381113	NC_000083.6	Cdkl4	1.15E-01
gene13286	243308	NC_000071.6	A430033K04Rik	1.15E-01
gene10658	71529	NC_000070.6	Kazn	1.15E-01
gene19665	57752	NC_000073.6	Tacc2	1.15E-01
gene2304	1E+08	NC_000067.6	Pydc3	1.14E-01
gene24840	73681	NC_000076.6	Trmt11	1.14E-01
gene24279	12773	NC_000075.6	Ccr4	1.14E-01
gene302	320705	NC_000067.6	Bend6	1.14E-01
gene38873	21826	NC_000083.6	Thbs2	1.14E-01
gene24537	320495	NC_000076.6	Ipcef1	1.14E-01
gene31054	78062	NC_000078.6	4930595D18Rik	1.14E-01
gene1973	26374	NC_000067.6	Rfwd2	1.14E-01
gene35730	545085	NC_000081.6	Wdr70	1.14E-01
gene16316	381979	NC_000073.6	Brsk1	1.14E-01
gene36222	1.1E+08	NC_000081.6	Gm41331	1.14E-01
gene11461	56873	NC_000071.6	Lmbr1	1.13E-01
gene35438	50933	NC_000080.6	Uchl3	1.13E-01
gene4558	99382	NC_000068.7	Abtb2	1.13E-01
gene2786	14007	NC_000068.7	Celf2	1.13E-01
gene15127	52372	NC_000072.6	D6Ertd527e	1.13E-01
gene17449	14447	NC_000073.6	Gapdhs	1.13E-01
gene26348	68876	NC_000076.6	Xrcc6bp1	1.13E-01
gene34981	629059	NC_000080.6	Fam124a	1.13E-01
gene9454	70122	NC_000070.6	Mllt3	1.13E-01
gene3375	241308	NC_000068.7	Ralgps1	1.13E-01

gene8174	1E+08	NC_000069.6	Gm19708	1.13E-01
gene23601	208982	NC_000075.6	Hmgcll1	1.13E-01
gene3500	241324	NC_000068.7	Crb2	1.13E-01
gene11799	1E+08	NC_000071.6	Ccdc149	1.13E-01
gene36016	1E+08	NC_000081.6	G930009F23Rik	1.13E-01
gene36180	210544	NC_000081.6	Tbc1d31	1.12E-01
gene36440	74281	NC_000081.6	Spatc1	1.12E-01
gene1918	207792	NC_000067.6	BC034090	1.12E-01
gene11887	243025	NC_000071.6	Tmem156	1.12E-01
gene34331	74480	NC_000080.6	Samd4	1.12E-01
gene18506	12173	NC_000073.6	Bnc1	1.12E-01
gene4745	66632	NC_000068.7	Dph6	1.12E-01
gene33463	408066	NC_000079.6	BC067074	1.12E-01
gene25322	69288	NC_000076.6	Rhobtb1	1.12E-01
gene38039	320007	NC_000082.6	Sidt1	1.12E-01
gene1946	78255	NC_000067.6	Ralgps2	1.12E-01
gene29073	208659	NC_000077.6	Fam20a	1.12E-01
gene32691	72061	NC_000079.6	2010111I01Rik	1.11E-01
gene30183	104806	NC_000078.6	Fancm	1.11E-01
gene5487	329540	NC_000068.7	Nol4l	1.11E-01
gene21359	74513	NC_000074.6	Neto2	1.11E-01
gene19814	=Gene;ger	NC_000073.6	LOC102635948	1.11E-01
gene2345	13603	NC_000067.6	Opn3	1.11E-01
gene11158	18671	NC_000071.6	Abcb1a	1.11E-01
gene37920	66667	NC_000082.6	Hspbap1	1.11E-01
gene11692	117591	NC_000071.6	Slc2a9	1.11E-01
gene2482	96935	NC_000067.6	Susd4	1.11E-01
gene33744	19270	NC_000080.6	Ptprg	1.11E-01
gene25854	1.1E+08	NC_000076.6	Gm40734	1.11E-01
gene36319	76510	NC_000081.6	Trappc9	1.10E-01
gene11123	18647	NC_000071.6	Cdk14	1.10E-01
gene9807	1E+08	NC_000070.6	Gm33192	1.10E-01
gene38031	212153	NC_000082.6	Ccdc191	1.10E-01
gene13924	83922	NC_000072.6	Cep41	1.10E-01
gene26100	52679	NC_000076.6	E2f7	1.10E-01
gene9317	214444	NC_000070.6	Cdk5rap2	1.10E-01
gene11131	207686	NC_000071.6	Cfap69	1.10E-01
gene13681	50799	NC_000072.6	Slc25a13	1.10E-01
gene25220	12817	NC_000076.6	Col13a1	1.10E-01
gene21488	14681	NC_000074.6	Gnao1	1.09E-01
gene30026	12810	NC_000078.6	Coch	1.09E-01
gene21877	1E+08	NC_000074.6	Gm16118	1.09E-01
gene7117	80891	NC_000069.6	Fcrls	1.09E-01
gene15817	21679	NC_000072.6	Tead4	1.09E-01
gene27957	11484	NC_000077.6	Aspa	1.09E-01
gene13722	15893	NC_000072.6	Ica1	1.09E-01
gene4653	1E+08	NC_000068.7	Gm30319	1.09E-01
gene1550	1E+08	NC_000067.6	Gm15674	1.09E-01

gene29443	13528	NC_000078.6	Dtnb	1.08E-01
gene12946	381741	NC_000071.6	Lrrc43	1.08E-01
gene24412	22354	NC_000075.6	Vipr1	1.08E-01
gene5170	66634	NC_000068.7	Mcm8	1.08E-01
gene5836	16500	NC_000068.7	Kcnb1	1.08E-01
gene14358	=Gene;ger	NC_000072.6	LOC108169140	1.08E-01
gene10461	230828	NC_000070.6	Il22ra1	1.07E-01
gene3789	75051	NC_000068.7	Ccdc173	1.07E-01
gene40795	64291	NC_000084.6	Osbpl1a	1.07E-01
gene14929	330355	NC_000072.6	Dnah6	1.07E-01
gene19812	76484	NC_000073.6	Kndc1	1.07E-01
gene12414	1.1E+08	NC_000071.6	Gm42139	1.07E-01
gene3685	407823	NC_000068.7	Baz2b	1.07E-01
gene24354	102448	NC_000075.6	Xylb	1.07E-01
gene33517	1E+08	NC_000079.6	Gm16263	1.06E-01
gene4600	22431	NC_000068.7	Wt1	1.06E-01
gene1626	240752	NC_000067.6	Pik3c2b	1.06E-01
gene17333	50760	NC_000073.6	Fbxo17	1.06E-01
gene31645	1E+08	NC_000079.6	Gm32242	1.06E-01
gene38666	1E+08	NC_000083.6	Gm34035	1.06E-01
gene30381	268566	NC_000078.6	Gphn	1.06E-01
gene33878	54169	NC_000080.6	Kat6b	1.06E-01
gene1648	1E+08	NC_000067.6	Gm31629	1.06E-01
gene8761	214944	NC_000070.6	Mob3b	1.06E-01
gene11551	107976	NC_000071.6	Bre	1.06E-01
gene10650	1E+08	NC_000070.6	Fhad1os2	1.06E-01
gene15684	93671	NC_000072.6	Cd163	1.06E-01
gene21080	1E+08	NC_000074.6	Fam129c	1.06E-01
gene27655	70383	NC_000077.6	Cox10	1.06E-01
gene573	227095	NC_000067.6	Hibch	1.06E-01
gene20916	72925	NC_000074.6	March1	1.06E-01
gene31391	22355	NC_000078.6	Vipr2	1.06E-01
gene35048	14365	NC_000080.6	Fzd3	1.05E-01
gene20462	270035	NC_000074.6	Letm2	1.05E-01
gene21661	234684	NC_000074.6	Lrrc29	1.05E-01
gene35941	56274	NC_000081.6	Stk3	1.05E-01
gene6575	57815	NC_000069.6	Spata5	1.05E-01
gene27601	109342	NC_000077.6	Slc5a10	1.05E-01
gene5279	66044	NC_000068.7	Dtd1	1.05E-01
gene39923	53901	NC_000083.6	Rcan2	1.05E-01
gene41364	69226	NC_000084.6	Snx24	1.05E-01
gene26332	20503	NC_000076.6	Slc16a7	1.05E-01
gene8736	12801	NC_000070.6	Cnr1	1.04E-01
gene12708	63873	NC_000071.6	Trpv4	1.04E-01
gene16397	330463	NC_000073.6	Zfp78	1.04E-01
gene38938	75202	NC_000083.6	Spaca6	1.04E-01
gene24726	353169	NC_000076.6	Slc2a12	1.04E-01
gene15989	67774	NC_000072.6	Borcs5	1.04E-01

gene24508	213783	NC_000076.6	Plekhg1	1.04E-01
gene20953	211135	NC_000074.6	D130040H23Rik	1.04E-01
gene14928	330354	NC_000072.6	Gm20560	1.04E-01
gene7106	1E+08	NC_000069.6	Gm31677	1.04E-01
gene13756	76311	NC_000072.6	1110019D14Rik	1.04E-01
gene23358	23938	NC_000075.6	Map2k5	1.03E-01
gene35005	382913	NC_000080.6	Neil2	1.03E-01
gene1526	22625	NC_000067.6	Map3k19	1.03E-01
gene26813	=Gene;ger	NC_000077.6	LOC101055656	1.03E-01
gene19501	11937	NC_000073.6	Atp2a1	1.03E-01
gene31642	140580	NC_000079.6	Elmo1	1.03E-01
gene23112	408028	NC_000075.6	Arhgap20os	1.03E-01
gene6775	1E+08	NC_000069.6	Gm26671	1.03E-01
gene14438	15400	NC_000072.6	Hoxa3	1.03E-01
gene1258	269224	NC_000067.6	Pask	1.03E-01
gene19443	233826	NC_000073.6	Palb2	1.03E-01
gene23496	76886	NC_000075.6	Fam81a	1.03E-01
gene41006	13824	NC_000084.6	Epb41l4a	1.03E-01
gene6333	381484	NC_000069.6	Gm5150	1.03E-01
gene25387	14687	NC_000076.6	Gnaz	1.03E-01
gene38732	1E+08	NC_000083.6	Rsph3b	1.03E-01
gene1852	69399	NC_000067.6	1700025G04Rik	1.03E-01
gene11558	665270	NC_000071.6	Plb1	1.03E-01
gene30724	71375	NC_000078.6	Foxn3	1.03E-01
gene24385	215446	NC_000075.6	Entpd3	1.02E-01
gene25754	1E+08	NC_000076.6	Gm38560	1.02E-01
gene33518	14165	NC_000079.6	Fgf10	1.02E-01
gene5208	74243	NC_000068.7	Slx4ip	1.02E-01
gene21892	75796	NC_000074.6	Cdyl2	1.02E-01
gene42445	353211	NC_000085.6	Prune2	1.02E-01
gene21238	330812	NC_000074.6	Rnf150	1.02E-01
gene38757	55951	NC_000083.6	Mpc1	1.02E-01
gene25567	15166	NC_000076.6	Hcn2	1.02E-01
gene39162	381072	NC_000083.6	Abca17	1.02E-01
gene20386	330721	NC_000074.6	Nek5	1.02E-01
gene37153	59033	NC_000081.6	Slc4a8	1.02E-01
gene15228	243538	NC_000072.6	Cfap100	1.02E-01
gene33723	268709	NC_000080.6	Fam107a	1.02E-01
gene2630	74023	NC_000067.6	Rd3	1.02E-01
gene10598	18602	NC_000070.6	Padi4	1.02E-01
gene5694	17865	NC_000068.7	Mybl2	1.02E-01
gene7931	56195	NC_000069.6	Ptbp2	1.01E-01
gene19281	1E+08	NC_000073.6	Micalcl	1.01E-01
gene32818	432770	NC_000079.6	Rslcan18	1.01E-01
gene3327	227721	NC_000068.7	Plpp7	1.01E-01
gene15826	791299	NC_000072.6	Gm10069	1.01E-01
gene5698	269389	NC_000068.7	Tox2	1.01E-01
gene4925	435684	NC_000068.7	Shf	1.01E-01

gene837	320933	NC_000067.6	D230017M19Rik	1.01E-01
gene32315	67911	NC_000079.6	Zfp169	1.01E-01
gene350	1E+08	NC_000067.6	Fer1l5	1.01E-01
gene25841	627270	NC_000076.6	Igf1os	1.01E-01
gene33063	72745	NC_000079.6	Tmem161b	1.01E-01
gene27610	1.1E+08	NC_000077.6	Gm39988	1.01E-01
gene20869	59009	NC_000074.6	Sh3rf1	1.01E-01
gene30526	73422	NC_000078.6	Prox2	1.01E-01
gene37214	223920	NC_000081.6	Soat2	1.00E-01
gene18552	72495	NC_000073.6	2610206C17Rik	1.00E-01
gene37102	72393	NC_000081.6	Faim2	1.00E-01
gene32119	1E+08	NC_000079.6	Gm15908	1.00E-01
gene20306	1E+08	NC_000074.6	Gm21811	1.00E-01
gene33089	12950	NC_000079.6	Hapln1	1.00E-01
gene26566	67596	NC_000076.6	Tespa1	1.00E-01
gene14499	330323	NC_000072.6	Fam188b	1.00E-01
gene4837	241624	NC_000068.7	Exd1	1.00E-01
gene21905	209239	NC_000074.6	Gan	9.99E-02
gene5025	1E+08	NC_000068.7	Gm14005	9.99E-02
gene33724	18300	NC_000080.6	Oit1	9.99E-02
gene24523	64009	NC_000076.6	Syne1	9.97E-02
gene25676	103406	NC_000076.6	Zfr2	9.95E-02
gene738	23792	NC_000067.6	Adam23	9.95E-02
gene10651	329977	NC_000070.6	Fhad1	9.94E-02
gene35946	666173	NC_000081.6	Vps13b	9.94E-02
gene35625	56643	NC_000080.6	Slc15a1	9.92E-02
gene42551	1E+08	NC_000085.6	Gm35438	9.91E-02
gene26067	216292	NC_000076.6	Mettl25	9.91E-02
gene10992	76866	NC_000070.6	Morn1	9.90E-02
gene17954	14344	NC_000073.6	Fut2	9.90E-02
gene19792	57740	NC_000073.6	Stk32c	9.89E-02
gene15432	58186	NC_000072.6	Rad18	9.89E-02
gene42926	71617	NC_000085.6	9130011E15Rik	9.89E-02
gene18295	67867	NC_000073.6	Lrrc28	9.88E-02
gene10449	230822	NC_000070.6	Ncmap	9.87E-02
gene3024	56075	NC_000068.7	Pdss1	9.86E-02
gene6837	1.1E+08	NC_000069.6	Gm37035	9.86E-02
gene18244	1E+08	NC_000073.6	Gm32633	9.84E-02
gene21233	16168	NC_000074.6	Il15	9.84E-02
gene11233	20346	NC_000071.6	Sema3a	9.83E-02
gene32952	74470	NC_000079.6	Cep72	9.83E-02
gene17123	22756	NC_000073.6	Zfp94	9.83E-02
gene13991	243755	NC_000072.6	Slc13a4	9.83E-02
gene28566	78473	NC_000077.6	Skap1	9.82E-02
gene9507	12578	NC_000070.6	Cdkn2a	9.81E-02
gene11564	1E+08	NC_000071.6	Gm2420	9.81E-02
gene30184	217653	NC_000078.6	Mis18bp1	9.80E-02
gene26812	17268	NC_000077.6	Meis1	9.80E-02

gene1857	269132	NC_000067.6	Colgalt2	9.80E-02
gene43038	1E+08	NC_000085.6	Gm31356	9.80E-02
gene10198	100177	NC_000070.6	Zmym6	9.79E-02
gene21691	72236	NC_000074.6	Tsnaxip1	9.79E-02
gene24796	268288	NC_000076.6	Samd3	9.78E-02
gene24909	71924	NC_000076.6	Tube1	9.78E-02
gene9387	17475	NC_000070.6	Mpdz	9.78E-02
gene6459	71827	NC_000069.6	Lrrc34	9.77E-02
gene16053	19277	NC_000072.6	Ptpro	9.77E-02
gene39173	268932	NC_000083.6	Caskin1	9.75E-02
gene39323	271424	NC_000083.6	Ip6k3	9.74E-02
gene6145	108115	NC_000068.7	Slco4a1	9.74E-02
gene4594	228413	NC_000068.7	Prrg4	9.74E-02
gene28558	15410	NC_000077.6	Hoxb3	9.73E-02
gene27240	68067	NC_000077.6	3010026O09Rik	9.73E-02
gene17686	233147	NC_000073.6	Zfp939	9.73E-02
gene42518	18719	NC_000085.6	Pip5k1b	9.72E-02
gene4456	1.1E+08	NC_000068.7	Gm13816	9.72E-02
gene568	227094	NC_000067.6	Tmem194b	9.72E-02
gene18492	244091	NC_000073.6	Fsd2	9.71E-02
gene30513	217708	NC_000078.6	Lin52	9.69E-02
gene2572	16526	NC_000067.6	Kcnk2	9.66E-02
gene27355	30805	NC_000077.6	Slc22a4	9.64E-02
gene28204	246104	NC_000077.6	Rhbdl3	9.63E-02
gene24224	791282	NC_000075.6	Gm10030	9.63E-02
gene14582	71484	NC_000072.6	8430415O14Rik	9.62E-02
gene42456	225994	NC_000085.6	Nmrk1	9.60E-02
gene40727	1E+08	NC_000084.6	Gm35180	9.58E-02
gene8938	242409	NC_000070.6	Tmem8b	9.58E-02
gene15168	65967	NC_000072.6	Eefsec	9.57E-02
gene38885	381062	NC_000083.6	Ermard	9.56E-02
gene40501	72167	NC_000083.6	Thumpd2	9.55E-02
gene5173	241639	NC_000068.7	Fermt1	9.55E-02
gene8063	170439	NC_000069.6	Elovl6	9.50E-02
gene6749	13175	NC_000069.6	Dclk1	9.50E-02
gene25329	11735	NC_000076.6	Ank3	9.49E-02
gene26985	414116	NC_000077.6	D630024D03Rik	9.49E-02
gene3319	23928	NC_000068.7	Lamc3	9.49E-02
gene12188	665563	NC_000071.6	Mthfd2l	9.45E-02
gene14504	11517	NC_000072.6	Adcyap1r1	9.44E-02
gene190	170829	NC_000067.6	Tram2	9.43E-02
gene22915	=Gene;ger	NC_000075.6	LOC102631930	9.43E-02
gene4723	74268	NC_000068.7	Aven	9.43E-02
gene37758	210126	NC_000082.6	Lpp	9.42E-02
gene17943	69217	NC_000073.6	Plekha4	9.41E-02
gene12939	269701	NC_000071.6	Wdr66	9.41E-02
gene7043	633285	NC_000069.6	Rbm46	9.39E-02
gene24039	70235	NC_000075.6	Poc1a	9.39E-02

gene41435	225579	NC_000084.6	Slc27a6	9.38E-02
gene15544	72263	NC_000072.6	1700030F04Rik	9.37E-02
gene25340	71721	NC_000076.6	Fam13c	9.36E-02
gene2808	209378	NC_000068.7	Itih5	9.36E-02
gene38509	70967	NC_000082.6	Eva1c	9.36E-02
gene39942	21645	NC_000083.6	Tcte1	9.35E-02
gene42457	67383	NC_000085.6	Carnmt1	9.35E-02
gene38711	77106	NC_000083.6	Tmem181a	9.31E-02
gene42109	107227	NC_000085.6	Macrod1	9.30E-02
gene36166	1.1E+08	NC_000081.6	Gm41326	9.29E-02
gene30736	217826	NC_000078.6	Kcnk13	9.28E-02
gene33091	13003	NC_000079.6	Vcan	9.27E-02
gene4535	241576	NC_000068.7	Ldlrad3	9.27E-02
gene28446	73922	NC_000077.6	4930405D11Rik	9.27E-02
gene39053	75424	NC_000083.6	Zfp820	9.26E-02
gene24950	327747	NC_000076.6	Mettl24	9.26E-02
gene32716	66503	NC_000079.6	1810034E14Rik	9.25E-02
gene1253	208777	NC_000067.6	Sned1	9.23E-02
gene22688	1E+08	NC_000075.6	Gm27219	9.23E-02
gene13822	30956	NC_000072.6	Aass	9.22E-02
gene11668	12933	NC_000071.6	Crmp1	9.21E-02
gene40677	73915	NC_000084.6	4833419F23Rik	9.20E-02
gene15055	75914	NC_000072.6	Exoc6b	9.20E-02
gene27616	327942	NC_000077.6	Pigl	9.20E-02
gene40655	77022	NC_000083.6	2700099C18Rik	9.19E-02
gene383	211484	NC_000067.6	Tsga10	9.19E-02
gene40717	75739	NC_000084.6	Mpp7	9.18E-02
gene23406	1E+08	NC_000075.6	Gm36538	9.18E-02
gene34171	71200	NC_000080.6	Dydc2	9.18E-02
gene20673	54384	NC_000074.6	Mtmr7	9.16E-02
gene19362	74424	NC_000073.6	Tmc5	9.16E-02
gene11967	22165	NC_000071.6	Txk	9.16E-02
gene999	16367	NC_000067.6	Irs1	9.15E-02
gene40320	268970	NC_000083.6	Arhgap28	9.15E-02
gene21163	78514	NC_000074.6	Arhgap10	9.14E-02
gene29981	217517	NC_000078.6	Stxbp6	9.14E-02
gene14594	20617	NC_000072.6	Snca	9.13E-02
gene35648	110821	NC_000080.6	Pcca	9.12E-02
gene30210	271005	NC_000078.6	Klhdc1	9.12E-02
gene1521	72160	NC_000067.6	Tmem163	9.11E-02
gene11488	381738	NC_000071.6	Drc1	9.09E-02
gene10046	242662	NC_000070.6	Rims3	9.09E-02
gene15597	330409	NC_000072.6	Cecr2	9.09E-02
gene4822	69408	NC_000068.7	Dnajc17	9.06E-02
gene1953	226525	NC_000067.6	Rasal2	9.06E-02
gene17749	434179	NC_000073.6	Gm5595	9.06E-02
gene6407	635702	NC_000069.6	Naaladl2	9.05E-02
gene14919	21415	NC_000072.6	Tcf7l1	9.05E-02

gene13581	12190	NC_000071.6	Brca2	9.04E-02
gene29110	69806	NC_000077.6	Slc39a11	9.04E-02
gene12706	433940	NC_000071.6	Fam222a	9.04E-02
gene1480	17167	NC_000067.6	Marco	9.02E-02
gene36156	12818	NC_000081.6	Col14a1	9.02E-02
gene9456	230393	NC_000070.6	Focad	9.01E-02
gene10547	18784	NC_000070.6	Pla2g5	9.01E-02
gene11513	69852	NC_000071.6	Tcf23	9.00E-02
gene33127	21828	NC_000079.6	Thbs4	8.99E-02
gene42866	93721	NC_000085.6	Cpn1	8.98E-02
gene1385	241197	NC_000067.6	Serpinb10	8.97E-02
gene33475	218639	NC_000079.6	Arl15	8.96E-02
gene6351	13123	NC_000069.6	Cyp7b1	8.96E-02
gene37540	58180	NC_000082.6	Hic2	8.96E-02
gene7033	56720	NC_000069.6	Tdo2	8.95E-02
gene784	17756	NC_000067.6	Map2	8.94E-02
gene36905	66871	NC_000081.6	Cpne8	8.94E-02
gene25951	12905	NC_000076.6	Cradd	8.93E-02
gene33012	68675	NC_000079.6	Fam172a	8.89E-02
gene42971	18011	NC_000085.6	Neurl1a	8.89E-02
gene24518	66084	NC_000076.6	Rmnd1	8.88E-02
gene20541	171167	NC_000074.6	Fut10	8.87E-02
gene434	110895	NC_000067.6	Slc9a4	8.86E-02
gene39791	258470	NC_000083.6	Olfr91	8.86E-02
gene8628	50500	NC_000070.6	Ttpa	8.86E-02
gene37513	224014	NC_000082.6	Fgd4	8.86E-02
gene3394	18516	NC_000068.7	Pbx3	8.84E-02
gene11748	16826	NC_000071.6	Ldb2	8.83E-02
gene40352	1E+08	NC_000083.6	Gm26510	8.82E-02
gene41597	14680	NC_000084.6	Gnal	8.81E-02
gene25342	70911	NC_000076.6	Phyhipl	8.81E-02
gene26723	1E+08	NC_000077.6	Gm36653	8.80E-02
gene1217	208727	NC_000067.6	Hdac4	8.80E-02
gene14481	1E+08	NC_000072.6	9130019P16Rik	8.80E-02
gene29876	1.1E+08	NC_000078.6	Gm40874	8.79E-02
gene21949	434353	NC_000074.6	A330074K22Rik	8.79E-02
gene25734	666584	NC_000076.6	BC024063	8.77E-02
gene40420	74196	NC_000083.6	Ttc27	8.77E-02
gene18294	=Gene;ger	NC_000073.6	LOC108167436	8.77E-02
gene33110	373852	NC_000079.6	4833422C13Rik	8.76E-02
gene11475	97209	NC_000071.6	A230098N10Rik	8.73E-02
gene26784	13649	NC_000077.6	Egfr	8.72E-02
gene37914	72599	NC_000082.6	Pdia5	8.72E-02
gene21204	68999	NC_000074.6	Anapc10	8.72E-02
gene16089	54611	NC_000072.6	Pde3a	8.70E-02
gene38082	433022	NC_000082.6	Plcxd2	8.70E-02
gene7017	70652	NC_000069.6	Tmem144	8.70E-02
gene32135	12593	NC_000079.6	Cdyl	8.69E-02

gene5387	78177	NC_000068.7	Ninl	8.66E-02
gene11384	108099	NC_000071.6	Prkag2	8.64E-02
gene30375	1E+08	NC_000078.6	Gm35240	8.64E-02
gene14889	52250	NC_000072.6	Reep1	8.63E-02
gene4788	381413	NC_000068.7	Gpr176	8.62E-02
gene13330	1.1E+08	NC_000071.6	Gm38728	8.61E-02
gene11549	71336	NC_000071.6	Rbks	8.61E-02
gene13900	320609	NC_000072.6	Strip2	8.59E-02
gene1061	227326	NC_000067.6	Gpr55	8.59E-02
gene20460	1E+08	NC_000074.6	Gm31288	8.58E-02
gene19413	233813	NC_000073.6	Vwa3a	8.58E-02
gene22271	1E+08	NC_000075.6	Gm19324	8.57E-02
gene2350	26909	NC_000067.6	Exo1	8.57E-02
gene9022	75691	NC_000070.6	Anks6	8.55E-02
gene15507	212516	NC_000072.6	Efcab12	8.54E-02
gene2485	68897	NC_000067.6	Disp1	8.51E-02
gene8967	319885	NC_000070.6	Zcchc7	8.51E-02
gene11770	20563	NC_000071.6	Slit2	8.50E-02
gene23153	94180	NC_000075.6	Acsbg1	8.50E-02
gene41289	28105	NC_000084.6	Trim36	8.49E-02
gene37497	19090	NC_000082.6	Prkdc	8.49E-02
gene28303	107476	NC_000077.6	Acaca	8.48E-02
gene38745	68655	NC_000083.6	Fndc1	8.48E-02
gene43137	1.1E+08	NC_000085.6	Gm41883	8.47E-02
gene40575	78586	NC_000083.6	Srbd1	8.47E-02
gene32219	1E+08	NC_000079.6	Gm32401	8.47E-02
gene41368	225518	NC_000084.6	Prdm6	8.46E-02
gene20736	69479	NC_000074.6	1700029J07Rik	8.46E-02
gene11662	269643	NC_000071.6	Ppp2r2c	8.46E-02
gene1520	1.1E+08	NC_000067.6	Gm41947	8.46E-02
gene24380	1E+08	NC_000075.6	Gm34159	8.46E-02
gene42438	14675	NC_000085.6	Gna14	8.45E-02
gene20149	619321	NC_000074.6	9530052E02Rik	8.45E-02
gene23794	108153	NC_000075.6	Adamts7	8.45E-02
gene38924	22759	NC_000083.6	Zfp97	8.43E-02
gene32102	68808	NC_000079.6	1110046J04Rik	8.41E-02
gene21940	69047	NC_000074.6	Atp2c2	8.41E-02
gene9902	230661	NC_000070.6	Tesk2	8.40E-02
gene10008	66101	NC_000070.6	Ppih	8.38E-02
gene8181	68369	NC_000069.6	0610031O16Rik	8.38E-02
gene23518	64008	NC_000075.6	Aqp9	8.37E-02
gene33346	71816	NC_000079.6	Rnf180	8.35E-02
gene38578	224440	NC_000082.6	Setd4	8.35E-02
gene23361	1E+08	NC_000075.6	Gm16759	8.33E-02
gene33339	67285	NC_000079.6	Cwc27	8.31E-02
gene24674	29863	NC_000076.6	Pde7b	8.29E-02
gene28439	217082	NC_000077.6	Hlf	8.29E-02
gene18304	16001	NC_000073.6	Igf1r	8.28E-02

gene22986	408059	NC_000075.6	BC049352	8.28E-02
gene30106	1E+08	NC_000078.6	Gm26973	8.28E-02
gene13952	243743	NC_000072.6	Plxna4	8.27E-02
gene21229	234515	NC_000074.6	Inpp4b	8.26E-02
gene12955	208628	NC_000071.6	Kntc1	8.26E-02
gene12179	73246	NC_000071.6	Rassf6	8.24E-02
gene8293	242253	NC_000069.6	Wdr63	8.22E-02
gene35417	52023	NC_000080.6	Pibf1	8.21E-02
gene37502	224008	NC_000082.6	Spidr	8.21E-02
gene6147	1E+08	NC_000068.7	Gm30144	8.21E-02
gene27232	1E+08	NC_000077.6	Gm36507	8.20E-02
gene18484	12877	NC_000073.6	Cpeb1	8.20E-02
gene3869	108699	NC_000068.7	Chn1	8.20E-02
gene15487	20965	NC_000072.6	Syn2	8.19E-02
gene3766	53416	NC_000068.7	Stk39	8.19E-02
gene8417	19218	NC_000069.6	Ptger3	8.19E-02
gene37089	16512	NC_000081.6	Kcnh3	8.18E-02
gene28914	72469	NC_000077.6	Plcd3	8.18E-02
gene32045	11607	NC_000079.6	Agtr1a	8.17E-02
gene43146	94281	NC_000085.6	Sfxn4	8.17E-02
gene9109	1E+08	NC_000070.6	Gm33729	8.17E-02
gene22610	382066	NC_000075.6	Prdm10	8.15E-02
gene315	75883	NC_000067.6	4930568A12Rik	8.15E-02
gene17747	208111	NC_000073.6	9830147E19Rik	8.14E-02
gene22368	72230	NC_000075.6	Zfp558	8.13E-02
gene18542	1E+08	NC_000073.6	Gm26708	8.12E-02
gene43103	73442	NC_000085.6	Hspa12a	8.11E-02
gene5798	114644	NC_000068.7	Slc13a3	8.11E-02
gene25741	237412	NC_000076.6	Gm4924	8.09E-02
gene29058	19739	NC_000077.6	Rgs9	8.08E-02
gene11916	11787	NC_000071.6	Apbb2	8.08E-02
gene986	69565	NC_000067.6	2310015K22Rik	8.08E-02
gene23085	1.1E+08	NC_000075.6	Gm39337	8.07E-02
gene406	18143	NC_000067.6	Npas2	8.06E-02
gene2736	209630	NC_000068.7	Frmd4a	8.06E-02
gene37592	13972	NC_000082.6	Gnb1l	8.05E-02
gene32321	218232	NC_000079.6	Ptpdc1	8.05E-02
gene24998	71365	NC_000076.6	Pdss2	8.05E-02
gene22441	53867	NC_000075.6	Col5a3	8.03E-02
gene38102	77647	NC_000082.6	Trat1	8.02E-02
gene4975	26458	NC_000068.7	Slc27a2	8.01E-02
gene24984	215951	NC_000076.6	Lace1	8.00E-02
gene34144	170677	NC_000080.6	Cdhr1	8.00E-02
gene38034	207798	NC_000082.6	Gramd1c	7.99E-02
gene40090	72238	NC_000083.6	Tbc1d5	7.99E-02
gene8420	107869	NC_000069.6	Cth	7.98E-02
gene20259	244329	NC_000074.6	Mcph1	7.98E-02
gene7570	54215	NC_000069.6	Cd160	7.97E-02

gene11547	1E+08	NC_000071.6	Gm38424	7.97E-02
gene12444	27405	NC_000071.6	Abcg3	7.97E-02
gene22623	330914	NC_000075.6	Arhgap32	7.97E-02
gene8893	ene;gene=l	NC_000070.6	LOC108168952	7.96E-02
gene22565	67484	NC_000075.6	Eepd1	7.95E-02
gene23664	70598	NC_000075.6	Filip1	7.95E-02
gene24511	270685	NC_000076.6	Mthfd1l	7.94E-02
gene4391	271813	NC_000068.7	Agbl2	7.94E-02
gene20169	=Gene;ger	NC_000074.6	LOC108167521	7.94E-02
gene9634	320508	NC_000070.6	Cachd1	7.94E-02
gene31613	1E+08	NC_000079.6	Gm31887	7.93E-02
gene1068	78795	NC_000067.6	Armc9	7.92E-02
gene5990	1E+08	NC_000068.7	Ppp4r1l-ps	7.91E-02
gene41539	667742	NC_000084.6	Piezo2	7.90E-02
gene23921	68121	NC_000075.6	Cep70	7.89E-02
gene19983	16535	NC_000073.6	Kcnq1	7.87E-02
gene3985	20378	NC_000068.7	Frzb	7.86E-02
gene17846	20981	NC_000073.6	Syt3	7.85E-02
gene35595	66435	NC_000080.6	Uggt2	7.84E-02
gene5370	668923	NC_000068.7	Zfp442	7.81E-02
gene31039	26448	NC_000078.6	Mok	7.80E-02
gene8265	12722	NC_000069.6	Clca3a1	7.79E-02
gene9968	68625	NC_000070.6	Cfap57	7.78E-02
gene19386	20216	NC_000073.6	Acsm3	7.77E-02
gene6739	55994	NC_000069.6	Smad9	7.77E-02
gene40576	18754	NC_000083.6	Prkce	7.75E-02
gene35914	20356	NC_000081.6	Sema5a	7.73E-02
gene20806	22341	NC_000074.6	Vegfc	7.71E-02
gene19222	320360	NC_000073.6	Ric3	7.69E-02
gene609	75388	NC_000067.6	Boll	7.68E-02
gene18432	77011	NC_000073.6	Ticrr	7.66E-02
gene32569	75050	NC_000079.6	Kif27	7.65E-02
gene20042	319493	NC_000074.6	A430078G23Rik	7.65E-02
gene39390	442827	NC_000083.6	Rab44	7.64E-02
gene1323	241175	NC_000067.6	Cntnap5b	7.64E-02
gene5637	228846	NC_000068.7	D630003M21Rik	7.64E-02
gene3305	14299	NC_000068.7	Ncs1	7.63E-02
gene15994	14760	NC_000072.6	Gpr19	7.61E-02
gene6745	70970	NC_000069.6	4931419H13Rik	7.61E-02
gene12376	70681	NC_000071.6	Fam175a	7.61E-02
gene3813	228019	NC_000068.7	Mettl8	7.59E-02
gene614	227120	NC_000067.6	Plcl1	7.59E-02
gene15194	66098	NC_000072.6	Chchd6	7.59E-02
gene33813	218793	NC_000080.6	Ube2e2	7.58E-02
gene25930	327799	NC_000076.6	Usp44	7.58E-02
gene6638	67161	NC_000069.6	Sclt1	7.57E-02
gene3551	1.1E+08	NC_000068.7	Gm39804	7.54E-02
gene36751	64706	NC_000081.6	Scube1	7.53E-02

gene3414	98870	NC_000068.7	AI182371	7.52E-02
gene26763	1E+08	NC_000077.6	Gm12002	7.52E-02
gene292	19076	NC_000067.6	Prim2	7.48E-02
gene40498	320784	NC_000083.6	C230072F16Rik	7.47E-02
gene35291	108811	NC_000080.6	Ccdc122	7.45E-02
gene5194	18798	NC_000068.7	Plcb4	7.45E-02
gene3235	68975	NC_000068.7	Med27	7.43E-02
gene11302	330050	NC_000071.6	Fam185a	7.41E-02
gene1513	210356	NC_000067.6	Nckap5	7.40E-02
gene16101	108096	NC_000072.6	Slco1a5	7.40E-02
gene18307	434197	NC_000073.6	Fam169b	7.40E-02
gene4611	66541	NC_000068.7	Immp1l	7.39E-02
gene15358	72171	NC_000072.6	Shq1	7.39E-02
gene37324	106393	NC_000082.6	Srl	7.39E-02
gene37905	239839	NC_000082.6	Ccdc14	7.39E-02
gene15277	1.1E+08	NC_000072.6	Gm38863	7.38E-02
gene17830	638411	NC_000073.6	Gm10109	7.37E-02
gene22544	73230	NC_000075.6	Bmper	7.37E-02
gene37885	239833	NC_000082.6	Lmln	7.37E-02
gene15288	67582	NC_000072.6	Slc25a26	7.36E-02
gene17170	18987	NC_000073.6	Pou2f2	7.35E-02
gene12324	77630	NC_000071.6	Prdm8	7.34E-02
gene33269	17952	NC_000079.6	Naip6	7.32E-02
gene1111	53972	NC_000067.6	Ngef	7.31E-02
gene40423	268977	NC_000083.6	Ltbp1	7.31E-02
gene10738	21941	NC_000070.6	Tnfrsf8	7.30E-02
gene13958	20336	NC_000072.6	Exoc4	7.30E-02
gene6454	14013	NC_000069.6	Mecom	7.29E-02
gene21479	234564	NC_000074.6	Ces1f	7.28E-02
gene6242	=Gene;ger	NC_000069.6	LOC102634900	7.27E-02
gene29751	195733	NC_000078.6	Grhl1	7.25E-02
gene4912	78655	NC_000068.7	Eif3j1	7.25E-02
gene24679	52906	NC_000076.6	Ahi1	7.24E-02
gene15605	194401	NC_000072.6	Mical3	7.24E-02
gene35225	214254	NC_000080.6	Nudt15	7.24E-02
gene38246	75145	NC_000082.6	4930547E14Rik	7.23E-02
gene2119	226610	NC_000067.6	Fam78b	7.23E-02
gene4722	213788	NC_000068.7	Chrm5	7.21E-02
gene662	1E+08	NC_000067.6	Als2cr11b	7.20E-02
gene29147	1E+08	NC_000077.6	Cd300ld4	7.20E-02
gene40549	240174	NC_000083.6	Thada	7.20E-02
gene21132	319555	NC_000074.6	Nwd1	7.20E-02
gene40018	75462	NC_000083.6	1700001C19Rik	7.17E-02
gene13783	64213	NC_000072.6	St7	7.14E-02
gene11814	231238	NC_000071.6	Sel1l3	7.14E-02
gene40606	210741	NC_000083.6	Kcnk12	7.14E-02
gene41225	77619	NC_000084.6	Prelid2	7.14E-02
gene3501	227801	NC_000068.7	Dennd1a	7.14E-02

gene28977	77097	NC_000077.6	Tanc2	7.13E-02
gene8400	209601	NC_000069.6	Erich3	7.13E-02
gene24536	18390	NC_000076.6	Oprm1	7.11E-02
gene11459	56874	NC_000071.6	Rnf32	7.10E-02
gene34985	71000	NC_000080.6	4931440J10Rik	7.10E-02
gene43111	214084	NC_000085.6	Slc18a2	7.09E-02
gene22193	93742	NC_000074.6	Pard3	7.05E-02
gene31513	208440	NC_000079.6	Dip2c	7.03E-02
gene29964	16981	NC_000078.6	Lrrn3	6.99E-02
gene5284	94249	NC_000068.7	Slc24a3	6.98E-02
gene13123	1E+08	NC_000071.6	Gm30003	6.97E-02
gene22275	22068	NC_000075.6	Trpc6	6.95E-02
gene36994	69354	NC_000081.6	Slc38a4	6.95E-02
gene7648	242122	NC_000069.6	Vtcn1	6.93E-02
gene3821	66559	NC_000068.7	Metap1d	6.93E-02
gene18698	244141	NC_000073.6	Nars2	6.93E-02
gene1709	12292	NC_000067.6	Cacna1s	6.92E-02
gene7694	329727	NC_000069.6	Dennd2c	6.92E-02
gene10747	433801	NC_000070.6	Gm13212	6.90E-02
gene17298	319482	NC_000073.6	9530053A07Rik	6.90E-02
gene22363	72560	NC_000075.6	Naalad2	6.87E-02
gene33116	17686	NC_000079.6	Msh3	6.85E-02
gene9201	230259	NC_000070.6	E130308A19Rik	6.84E-02
gene9429	338349	NC_000070.6	Cntln	6.84E-02
gene32568	1E+08	NC_000079.6	Gm26555	6.84E-02
gene29042	18750	NC_000077.6	Prkca	6.83E-02
gene2925	241230	NC_000068.7	St8sia6	6.82E-02
gene9845	20460	NC_000070.6	Stil	6.82E-02
gene10278	1E+08	NC_000070.6	Gm12963	6.81E-02
gene688	241066	NC_000067.6	Carf	6.80E-02
gene40891	13527	NC_000084.6	Dtna	6.79E-02
gene15324	330390	NC_000072.6	Gm765	6.79E-02
gene30549	217721	NC_000078.6	Mfsd7c	6.79E-02
gene8002	19142	NC_000069.6	Prss12	6.79E-02
gene25748	21665	NC_000076.6	Tdg	6.78E-02
gene2516	226778	NC_000067.6	Mark1	6.77E-02
gene9401	329872	NC_000070.6	Frem1	6.76E-02
gene38734	68195	NC_000083.6	Rnaset2b	6.75E-02
gene22897	1E+08	NC_000075.6	Gm16322	6.73E-02
gene13644	101202	NC_000072.6	Hepacam2	6.73E-02
gene16673	232889	NC_000073.6	Pla2g4c	6.70E-02
gene38769	23984	NC_000083.6	Pde10a	6.70E-02
gene9125	242466	NC_000070.6	Zfp462	6.69E-02
gene6018	1E+08	NC_000068.7	Zfp831	6.69E-02
gene4960	99100	NC_000068.7	Cep152	6.69E-02
gene33853	76670	NC_000080.6	Cfap70	6.68E-02
gene36162	20649	NC_000081.6	Sntb1	6.68E-02
gene41490	170826	NC_000084.6	Ppargc1b	6.67E-02

gene9924	381544	NC_000070.6	Gm1661	6.65E-02
gene22296	270118	NC_000075.6	Maml2	6.62E-02
gene741	76668	NC_000067.6	Mdh1b	6.61E-02
gene25451	59093	NC_000076.6	Pcbp3	6.60E-02
gene41408	320253	NC_000084.6	March3	6.60E-02
gene35450	71264	NC_000080.6	4933432I03Rik	6.60E-02
gene2926	30963	NC_000068.7	Hacd1	6.60E-02
gene16051	232441	NC_000072.6	Rerg	6.59E-02
gene12614	209683	NC_000071.6	Ttc28	6.57E-02
gene20909	407795	NC_000074.6	BC030870	6.57E-02
gene2384	68226	NC_000067.6	Efcab2	6.57E-02
gene3580	109241	NC_000068.7	Mbd5	6.56E-02
gene15998	381822	NC_000072.6	Lockd	6.56E-02
gene4101	258571	NC_000068.7	Olfr1033	6.56E-02
gene38296	74185	NC_000082.6	Gbe1	6.56E-02
gene9621	230500	NC_000070.6	Efcab7	6.54E-02
gene41753	108052	NC_000084.6	Slc14a1	6.53E-02
gene36013	1E+08	NC_000081.6	Gm35248	6.53E-02
gene42809	71085	NC_000085.6	Arhgap19	6.52E-02
gene20762	71069	NC_000074.6	Stox2	6.52E-02
gene30744	1E+08	NC_000078.6	Gm10432	6.52E-02
gene41713	269037	NC_000084.6	Ctif	6.52E-02
gene8177	17777	NC_000069.6	Mttp	6.50E-02
gene32987	73296	NC_000079.6	Rhobtb3	6.50E-02
gene27374	75530	NC_000077.6	Lyrm7	6.50E-02
gene13270	381680	NC_000071.6	Nxpe5	6.50E-02
gene39353	268935	NC_000083.6	Scube3	6.49E-02
gene21172	=Gene;ger	NC_000074.6	LOC108167319	6.48E-02
gene1510	71111	NC_000067.6	Gpr39	6.48E-02
gene2005	22164	NC_000067.6	Tnfsf4	6.47E-02
gene1878	74091	NC_000067.6	Npl	6.47E-02
gene19114	434223	NC_000073.6	Gm1966	6.46E-02
gene6958	66183	NC_000069.6	Sptssb	6.45E-02
gene12158	54403	NC_000071.6	Slc4a4	6.45E-02
gene21472	20538	NC_000074.6	Slc6a2	6.43E-02
gene15064	94282	NC_000072.6	Sfxn5	6.42E-02
gene3222	68870	NC_000068.7	Ak8	6.41E-02
gene26288	ene;gene=l	NC_000076.6	LOC102639054	6.40E-02
gene4610	77766	NC_000068.7	Elp4	6.40E-02
gene24645	215821	NC_000076.6	Arfgef3	6.40E-02
gene38021	56490	NC_000082.6	Zbtb20	6.39E-02
gene12666	52850	NC_000071.6	Sgsm1	6.38E-02
gene18075	=Gene;ger	NC_000073.6	LOC108167511	6.37E-02
gene33524	56838	NC_000079.6	Ccl28	6.36E-02
gene34081	69069	NC_000080.6	1810011H11Rik	6.36E-02
gene33381	75597	NC_000079.6	Ndufaf2	6.35E-02
gene36267	11514	NC_000081.6	Adcy8	6.35E-02
gene6916	66880	NC_000069.6	Rsrc1	6.35E-02

gene8995	381522	NC_000070.6	Ccdc180	6.34E-02
gene29948	20259	NC_000078.6	Scin	6.33E-02
gene7919	229791	NC_000069.6	Plppr4	6.33E-02
gene28410	76626	NC_000077.6	Msi2	6.33E-02
gene1725	1E+08	NC_000067.6	Platr22	6.32E-02
gene13363	243328	NC_000071.6	Slc29a4	6.31E-02
gene468	14200	NC_000067.6	Fhl2	6.30E-02
gene17277	112415	NC_000073.6	C030039L03Rik	6.29E-02
gene36213	68501	NC_000081.6	Nsmce2	6.29E-02
gene25253	67307	NC_000076.6	Pbld2	6.27E-02
gene12194	12223	NC_000071.6	Btc	6.24E-02
gene37091	1E+08	NC_000081.6	Gm34338	6.23E-02
gene32688	14121	NC_000079.6	Fbp1	6.23E-02
gene27104	380694	NC_000077.6	Ccnjl	6.23E-02
gene42707	319931	NC_000085.6	A330032B11Rik	6.23E-02
gene30289	104001	NC_000078.6	Rtn1	6.23E-02
gene29765	1E+08	NC_000078.6	Gm36287	6.22E-02
gene3373	99326	NC_000068.7	Garnl3	6.22E-02
gene13979	76223	NC_000072.6	Agbl3	6.22E-02
gene34006	218865	NC_000080.6	Chdh	6.21E-02
gene6623	269423	NC_000069.6	Abhd18	6.21E-02
gene28373	114714	NC_000077.6	Rad51c	6.21E-02
gene20937	234356	NC_000074.6	Csgalnact1	6.21E-02
gene36812	19013	NC_000081.6	Ppara	6.20E-02
gene32660	630579	NC_000079.6	Zfp808	6.19E-02
gene2651	1E+08	NC_000067.6	Gm31406	6.19E-02
gene24971	103268	NC_000076.6	Cep57l1	6.18E-02
gene25242	216021	NC_000076.6	Stox1	6.18E-02
gene42841	74580	NC_000085.6	Pyroxd2	6.17E-02
gene21715	58994	NC_000074.6	Smpd3	6.17E-02
gene25767	69784	NC_000076.6	1500009L16Rik	6.17E-02
gene39446	12411	NC_000083.6	Cbs	6.15E-02
gene38173	320712	NC_000082.6	Abi3bp	6.14E-02
gene40370	68789	NC_000083.6	Trmt61b	6.14E-02
gene20932	234353	NC_000074.6	Psd3	6.13E-02
gene15461	211651	NC_000072.6	Fancd2	6.12E-02
gene29539	238076	NC_000078.6	Kcns3	6.12E-02
gene27302	213084	NC_000077.6	Cdkl3	6.11E-02
gene8567	208820	NC_000070.6	Triqk	6.10E-02
gene5510	66971	NC_000068.7	Cdk5rap1	6.10E-02
gene15689	232370	NC_000072.6	Clstn3	6.10E-02
gene17585	68947	NC_000073.6	Chst8	6.09E-02
gene578	227099	NC_000067.6	Pms1	6.08E-02
gene42706	208922	NC_000085.6	Cpeb3	6.08E-02
gene26900	1.1E+08	NC_000077.6	Gm12061	6.08E-02
gene8661	212377	NC_000070.6	Mms22l	6.08E-02
gene2917	19212	NC_000068.7	Pter	6.07E-02
gene15617	232345	NC_000072.6	A2m	6.06E-02

gene40543	1E+08	NC_000083.6	Gm35551	6.05E-02
gene16299	243816	NC_000073.6	Gp6	6.04E-02
gene12356	231503	NC_000071.6	Tmem150c	6.04E-02
gene10463	242702	NC_000070.6	Myom3	6.04E-02
gene10024	16656	NC_000070.6	Hivep3	6.04E-02
gene19379	117147	NC_000073.6	Acsm1	6.03E-02
gene21764	11906	NC_000074.6	Zfhx3	6.03E-02
gene10277	230775	NC_000070.6	Adgrb2	6.03E-02
gene18378	20450	NC_000073.6	St8sia2	6.03E-02
gene587	329152	NC_000067.6	Hecw2	6.01E-02
gene6602	329628	NC_000069.6	Fat4	6.01E-02
gene8180	11529	NC_000069.6	Adh7	6.00E-02
gene30552	320244	NC_000078.6	Ttll5	6.00E-02
gene35956	26942	NC_000081.6	Spag1	5.99E-02
gene19834	244234	NC_000073.6	5830411N06Rik	5.99E-02
gene1021	77629	NC_000067.6	Sphkap	5.99E-02
gene30074	72658	NC_000078.6	2700097O09Rik	5.98E-02
gene15883	170733	NC_000072.6	Klra17	5.96E-02
gene11671	68525	NC_000071.6	Evc2	5.96E-02
gene18674	75985	NC_000073.6	Rab30	5.96E-02
gene18436	626359	NC_000073.6	Wdr93	5.95E-02
gene23979	270192	NC_000075.6	Rab6b	5.95E-02
gene33095	108138	NC_000079.6	Xrcc4	5.95E-02
gene19221	22141	NC_000073.6	Tub	5.95E-02
gene40012	224829	NC_000083.6	Trerf1	5.93E-02
gene13249	13113	NC_000071.6	Cyp3a13	5.93E-02
gene32210	17152	NC_000079.6	Mak	5.92E-02
gene15575	319734	NC_000072.6	Cacna2d4	5.92E-02
gene21668	270091	NC_000074.6	Lrrc36	5.92E-02
gene5273	241688	NC_000068.7	Dzank1	5.91E-02
gene39558	12815	NC_000083.6	Col11a2	5.91E-02
gene37775	239789	NC_000082.6	Gmnc	5.90E-02
gene4640	228421	NC_000068.7	Kif18a	5.88E-02
gene27777	327954	NC_000077.6	Dnah2	5.87E-02
gene40359	17929	NC_000083.6	Myom1	5.87E-02
gene25615	23805	NC_000076.6	Apc2	5.86E-02
gene15299	20917	NC_000072.6	Suclg2	5.84E-02
gene13404	=Gene;ger	NC_000071.6	LOC108169081	5.83E-02
gene33001	78771	NC_000079.6	Mctp1	5.83E-02
gene28605	56013	NC_000077.6	Srcin1	5.82E-02
gene27416	=Gene;ger	NC_000077.6	LOC102635527	5.81E-02
gene29934	14009	NC_000078.6	Etv1	5.81E-02
gene24993	268297	NC_000076.6	Scml4	5.81E-02
gene23860	235533	NC_000075.6	Gk5	5.80E-02
gene19328	233765	NC_000073.6	Plekha7	5.80E-02
gene17631	243931	NC_000073.6	Tshz3	5.80E-02
gene19391	381917	NC_000073.6	Dnah3	5.79E-02
gene37435	791359	NC_000082.6	Gm9961	5.78E-02

gene15502	94346	NC_000072.6	Tmem40	5.77E-02
gene1143	1E+08	NC_000067.6	Mroh2a	5.77E-02
gene15799	381813	NC_000072.6	Prmt8	5.76E-02
gene13034	243274	NC_000071.6	Tmem132d	5.75E-02
gene15519	67492	NC_000072.6	Zfand4	5.75E-02
gene11665	76071	NC_000071.6	Jakmip1	5.73E-02
gene4932	381417	NC_000068.7	Gm14085	5.72E-02
gene6616	380614	NC_000069.6	Intu	5.71E-02
gene26282	24117	NC_000076.6	Wif1	5.71E-02
gene28332	192197	NC_000077.6	Bcas3	5.71E-02
gene25201	237360	NC_000076.6	Adamts14	5.69E-02
gene13796	75196	NC_000072.6	Ankrd7	5.69E-02
gene3529	1E+08	NC_000068.7	Gm30067	5.68E-02
gene16148	320662	NC_000072.6	Casc1	5.68E-02
gene29683	625810	NC_000078.6	Gm6626	5.67E-02
gene23178	244891	NC_000075.6	Scaper	5.67E-02
gene22346	234964	NC_000075.6	Ccdc67	5.67E-02
gene10295	67149	NC_000070.6	Nkain1	5.66E-02
gene19670	330660	NC_000073.6	Btbd16	5.66E-02
gene3694	18779	NC_000068.7	Pla2r1	5.66E-02
gene10917	1.1E+08	NC_000070.6	Gm42357	5.64E-02
gene22278	71544	NC_000075.6	Arhgap42	5.62E-02
gene2082	240888	NC_000067.6	Gpr161	5.60E-02
gene10012	332934	NC_000070.6	Zmynd12	5.59E-02
gene900	22420	NC_000067.6	Wnt6	5.59E-02
gene18505	ene;gene=L	NC_000073.6	LOC108167484	5.59E-02
gene12620	433938	NC_000071.6	Mn1	5.59E-02
gene20312	381598	NC_000074.6	2610005L07Rik	5.58E-02
gene21136	16795	NC_000074.6	Large	5.57E-02
gene3622	12298	NC_000068.7	Cacnb4	5.56E-02
gene28383	327992	NC_000077.6	Hsf5	5.56E-02
gene20387	23954	NC_000074.6	Nek3	5.55E-02
gene191	75712	NC_000067.6	Tmem14a	5.54E-02
gene7964	11304	NC_000069.6	Abca4	5.54E-02
gene21025	110385	NC_000074.6	Pde4c	5.54E-02
gene8839	68922	NC_000070.6	Dnaic1	5.54E-02
gene40780	338363	NC_000084.6	Tmem241	5.53E-02
gene16255	81904	NC_000073.6	Cacng7	5.53E-02
gene7848	57257	NC_000069.6	Vav3	5.53E-02
gene41871	1.1E+08	NC_000084.6	Gm41800	5.50E-02
gene12334	19092	NC_000071.6	Prkg2	5.50E-02
gene38869	64074	NC_000083.6	Smoc2	5.50E-02
gene28441	20913	NC_000077.6	Stxbp4	5.50E-02
gene35033	110265	NC_000080.6	Msra	5.49E-02
gene15074	236266	NC_000072.6	Alms1	5.48E-02
gene24959	633979	NC_000076.6	Ak9	5.47E-02
gene10905	18628	NC_000070.6	Per3	5.46E-02
gene42015	1E+08	NC_000085.6	Gm31166	5.45E-02

gene23144	270162	NC_000075.6	Elmod1	5.45E-02
gene8294	171166	NC_000069.6	Mcoln3	5.45E-02
gene41518	1.1E+08	NC_000084.6	Gm41750	5.44E-02
gene26663	407821	NC_000077.6	Znrf3	5.44E-02
gene13831	320405	NC_000072.6	Cadps2	5.43E-02
gene21190	622675	NC_000074.6	Zfp827	5.43E-02
gene38122	433024	NC_000082.6	Gm5486	5.43E-02
gene8092	16842	NC_000069.6	Lef1	5.42E-02
gene35881	110082	NC_000081.6	Dnah5	5.42E-02
gene11142	207618	NC_000071.6	Zfp804b	5.42E-02
gene4648	68201	NC_000068.7	Ccdc34	5.42E-02
gene137	29819	NC_000067.6	Stau2	5.41E-02
gene18073	78286	NC_000073.6	Nav2	5.41E-02
gene40330	13823	NC_000083.6	Epb41l3	5.40E-02
gene35407	13134	NC_000080.6	Dach1	5.39E-02
gene29067	74008	NC_000077.6	Arsg	5.39E-02
gene36305	13992	NC_000081.6	Khdrbs3	5.38E-02
gene2652	1.1E+08	NC_000067.6	Gm39747	5.34E-02
gene37393	70426	NC_000082.6	Tekt5	5.33E-02
gene25081	19886	NC_000076.6	Ros1	5.32E-02
gene40816	72504	NC_000084.6	Taf4b	5.30E-02
gene40466	1.1E+08	NC_000083.6	Gm41623	5.29E-02
gene29413	217371	NC_000077.6	Rab40b	5.28E-02
gene892	18802	NC_000067.6	Plcd4	5.28E-02
gene26063	278279	NC_000076.6	Tmtc2	5.28E-02
gene36423	432964	NC_000081.6	K230010J24Rik	5.27E-02
gene33527	245269	NC_000079.6	Nim1k	5.27E-02
gene10009	73332	NC_000070.6	Ccdc30	5.26E-02
gene33838	18074	NC_000080.6	Nid2	5.25E-02
gene42342	664779	NC_000085.6	Gm44505	5.25E-02
gene7634	74362	NC_000069.6	Spag17	5.25E-02
gene20089	20300	NC_000074.6	Ccl25	5.24E-02
gene5329	1E+08	NC_000068.7	9030622O22Rik	5.23E-02
gene11303	320118	NC_000071.6	Fbxl13	5.22E-02
gene42510	319924	NC_000085.6	Apba1	5.20E-02
gene23658	12816	NC_000075.6	Col12a1	5.20E-02
gene2807	16425	NC_000068.7	Itih2	5.19E-02
gene4969	75823	NC_000068.7	Fam227b	5.19E-02
gene42237	403202	NC_000085.6	A430093F15Rik	5.18E-02
gene11304	74511	NC_000071.6	Lrrc17	5.18E-02
gene842	381270	NC_000067.6	March4	5.15E-02
gene31527	11472	NC_000079.6	Actn2	5.15E-02
gene3001	208618	NC_000068.7	Etl4	5.15E-02
gene18527	233424	NC_000073.6	Tmc3	5.14E-02
gene37690	71751	NC_000082.6	Map3k13	5.14E-02
gene6328	668101	NC_000069.6	Sirpb1b	5.11E-02
gene15492	19016	NC_000072.6	Pparg	5.11E-02
gene14468	231991	NC_000072.6	Creb5	5.11E-02

gene22276	1E+08	NC_000075.6	Gm32014	5.09E-02
gene27352	56517	NC_000077.6	Slc22a21	5.09E-02
gene25828	103220	NC_000076.6	Ttc41	5.08E-02
gene33315	328329	NC_000079.6	Mast4	5.06E-02
gene18528	1E+08	NC_000073.6	Gm16638	5.06E-02
gene38626	50795	NC_000082.6	Sh3bgr	5.05E-02
gene19093	77505	NC_000073.6	Dnhd1	5.05E-02
gene18544	11864	NC_000073.6	Arnt2	5.04E-02
gene39986	106763	NC_000083.6	Ttbk1	5.04E-02
gene9293	1E+08	NC_000070.6	8030451A03Rik	5.02E-02
gene24713	=Gene;ger	NC_000076.6	LOC108167355	5.01E-02
gene8962	18507	NC_000070.6	Pax5	5.01E-02
gene35147	75016	NC_000080.6	4930480K23Rik	5.00E-02
gene18716	1.1E+08	NC_000073.6	Gm39050	5.00E-02
gene27158	171283	NC_000077.6	Havcr1	4.99E-02
gene39426	13417	NC_000083.6	Dnah8	4.99E-02
gene5491	13436	NC_000068.7	Dnmt3b	4.97E-02
gene24420	1E+08	NC_000075.6	Ccdc13	4.97E-02
gene6963	625901	NC_000069.6	Gm6634	4.96E-02
gene20803	234258	NC_000074.6	Neil3	4.95E-02
gene28297	1E+08	NC_000077.6	Gm12576	4.95E-02
gene16113	20928	NC_000072.6	Abcc9	4.94E-02
gene25658	14708	NC_000076.6	Gng7	4.93E-02
gene15164	1.1E+08	NC_000072.6	Gm38708	4.93E-02
gene71	17864	NC_000067.6	Mybl1	4.92E-02
gene15281	14924	NC_000072.6	Magi1	4.91E-02
gene25847	75317	NC_000076.6	Parpbp	4.91E-02
gene2059	14067	NC_000067.6	F5	4.91E-02
gene23474	19883	NC_000075.6	Rora	4.91E-02
gene41837	240476	NC_000084.6	Zfp407	4.90E-02
gene6370	381485	NC_000069.6	Trim55	4.89E-02
gene3661	241391	NC_000068.7	Galnt5	4.89E-02
gene32668	1E+08	NC_000079.6	Gm3604	4.88E-02
gene5615	629499	NC_000068.7	Mroh8	4.88E-02
gene32700	14088	NC_000079.6	Fancc	4.85E-02
gene34009	12289	NC_000080.6	Cacna1d	4.85E-02
gene36133	1.1E+08	NC_000081.6	Gm41323	4.85E-02
gene8155	242248	NC_000069.6	Bank1	4.84E-02
gene3528	320271	NC_000068.7	Scai	4.84E-02
gene3018	667663	NC_000068.7	Myo3a	4.84E-02
gene18712	75448	NC_000073.6	Rsf1os1	4.83E-02
gene19263	17540	NC_000073.6	Mrvi1	4.83E-02
gene10974	70673	NC_000070.6	Prdm16	4.82E-02
gene17993	27414	NC_000073.6	Sergef	4.82E-02
gene21726	272538	NC_000074.6	Tango6	4.82E-02
gene6756	26422	NC_000069.6	Nbea	4.81E-02
gene15375	12661	NC_000072.6	Chl1	4.80E-02
gene32142	69955	NC_000079.6	Fars2	4.80E-02

gene6786	1.1E+08	NC_000069.6	Gm40055	4.80E-02
gene26902	14025	NC_000077.6	Bcl11a	4.79E-02
gene9279	73750	NC_000070.6	Whrn	4.79E-02
gene24116	12217	NC_000075.6	Bsn	4.78E-02
gene1719	381293	NC_000067.6	Kif14	4.78E-02
gene20308	547150	NC_000074.6	6820431F20Rik	4.77E-02
gene40920	225289	NC_000084.6	AW554918	4.77E-02
gene14873	12180	NC_000072.6	Smyd1	4.76E-02
gene32139	380840	NC_000079.6	Lyrm4	4.76E-02
gene15113	72012	NC_000072.6	1600020E01Rik	4.76E-02
gene37741	17174	NC_000082.6	Masp1	4.74E-02
gene26168	58802	NC_000076.6	Kcnmb4	4.74E-02
gene30266	76967	NC_000078.6	2700049A03Rik	4.72E-02
gene28341	237911	NC_000077.6	Brip1	4.70E-02
gene30774	238384	NC_000078.6	Slc24a4	4.69E-02
gene24709	379043	NC_000076.6	Raet1e	4.69E-02
gene33108	66970	NC_000079.6	Ssbp2	4.69E-02
gene39929	12393	NC_000083.6	Runx2	4.69E-02
gene24641	215819	NC_000076.6	Nhsl1	4.68E-02
gene12496	1E+08	NC_000071.6	Gm17202	4.68E-02
gene25923	237465	NC_000076.6	Ccdc38	4.68E-02
gene18350	244049	NC_000073.6	Mctp2	4.67E-02
gene26670	76389	NC_000077.6	Ankrd36	4.66E-02
gene37768	210530	NC_000082.6	P3h2	4.64E-02
gene13030	208213	NC_000071.6	Tmem132c	4.64E-02
gene12440	626391	NC_000071.6	Zfp951	4.64E-02
gene24149	171429	NC_000075.6	Slc26a6	4.64E-02
gene35078	11549	NC_000080.6	Adra1a	4.64E-02
gene3552	76117	NC_000068.7	Arhgap15	4.63E-02
gene27051	20564	NC_000077.6	Slit3	4.63E-02
gene29550	1E+08	NC_000078.6	Gm34237	4.62E-02
gene8901	230085	NC_000070.6	Phf24	4.61E-02
gene22990	214531	NC_000075.6	Tmprss13	4.61E-02
gene20668	78506	NC_000074.6	Micu3	4.61E-02
gene24520	1E+08	NC_000076.6	Ccdc170	4.59E-02
gene28628	72973	NC_000077.6	Fbxo47	4.58E-02
gene38725	83672	NC_000083.6	Sytl3	4.57E-02
gene2132	20183	NC_000067.6	Rxrg	4.55E-02
gene12495	114642	NC_000071.6	Brdt	4.54E-02
gene25151	76138	NC_000076.6	Ccdc138	4.53E-02
gene40997	12326	NC_000084.6	Camk4	4.53E-02
gene34046	110084	NC_000080.6	Dnah1	4.52E-02
gene29545	26950	NC_000078.6	Vsnl1	4.52E-02
gene40140	52614	NC_000083.6	Adgre4	4.52E-02
gene30334	72338	NC_000078.6	Wdr89	4.52E-02
gene32998	238726	NC_000079.6	Fam81b	4.51E-02
gene32999	1E+08	NC_000079.6	Gm31219	4.50E-02
gene30747	73086	NC_000078.6	Rps6ka5	4.50E-02

gene33388	218581	NC_000079.6	Depdc1b	4.49E-02
gene6573	14173	NC_000069.6	Fgf2	4.49E-02
gene32173	71213	NC_000079.6	Cage1	4.49E-02
gene8023	271970	NC_000069.6	Arsj	4.48E-02
gene43050	21416	NC_000085.6	Tcf7l2	4.47E-02
gene3068	329358	NC_000068.7	Gm13387	4.47E-02
gene8040	71643	NC_000069.6	Zgrf1	4.46E-02
gene6576	1E+08	NC_000069.6	Gm36412	4.46E-02
gene41759	240427	NC_000084.6	Setbp1	4.45E-02
gene38693	224454	NC_000083.6	Zdhhc14	4.45E-02
gene21431	244579	NC_000074.6	Tox3	4.45E-02
gene13963	74354	NC_000072.6	Lrguk	4.43E-02
gene18718	233537	NC_000073.6	Gdpd4	4.42E-02
gene16247	378425	NC_000073.6	Nlrp12	4.42E-02
gene4460	80976	NC_000068.7	Syt13	4.42E-02
gene15443	269788	NC_000072.6	Lhfpl4	4.42E-02
gene23458	70549	NC_000075.6	Tln2	4.41E-02
gene37911	224129	NC_000082.6	Adcy5	4.41E-02
gene492	70676	NC_000067.6	Gulp1	4.39E-02
gene24583	13853	NC_000076.6	Epm2a	4.39E-02
gene39416	74762	NC_000083.6	Mdga1	4.38E-02
gene2929	625286	NC_000068.7	Tmem236	4.38E-02
gene24793	77220	NC_000076.6	Tmem200a	4.37E-02
gene1868	226518	NC_000067.6	Nmnat2	4.36E-02
gene30014	18760	NC_000078.6	Prkd1	4.35E-02
gene30935	212198	NC_000078.6	Wdr25	4.35E-02
gene8710	12014	NC_000070.6	Bach2	4.34E-02
gene37367	268860	NC_000082.6	Abat	4.34E-02
gene10243	1E+08	NC_000070.6	Gm12976	4.34E-02
gene37793	224079	NC_000082.6	Atp13a4	4.32E-02
gene43153	547091	NC_000085.6	Gm6020	4.31E-02
gene8832	109242	NC_000070.6	Kif24	4.30E-02
gene30667	75216	NC_000078.6	Cep128	4.30E-02
gene13320	17120	NC_000071.6	Mad1l1	4.29E-02
gene19226	117229	NC_000073.6	Stk33	4.29E-02
gene9620	67733	NC_000070.6	Itgb3bp	4.28E-02
gene32275	20238	NC_000079.6	Atxn1	4.28E-02
gene17991	16502	NC_000073.6	Kcnc1	4.27E-02
gene7674	12373	NC_000069.6	Casq2	4.24E-02
gene6973	12038	NC_000069.6	Bche	4.24E-02
gene12574	1E+08	NC_000071.6	4930522L14Rik	4.24E-02
gene22277	18667	NC_000075.6	Pgr	4.23E-02
gene41630	1E+08	NC_000084.6	Gm30821	4.23E-02
gene18230	11441	NC_000073.6	Chrna7	4.23E-02
gene5187	18795	NC_000068.7	Plcb1	4.22E-02
gene38608	1E+08	NC_000082.6	Gm31641	4.20E-02
gene8583	73314	NC_000070.6	Lrrc69	4.19E-02
gene33523	633640	NC_000079.6	Gm7120	4.18E-02

gene13576	320365	NC_000071.6	Fry	4.14E-02
gene22690	208076	NC_000075.6	Pknox2	4.14E-02
gene3835	56508	NC_000068.7	Rapgef4	4.13E-02
gene1172	74918	NC_000067.6	Iqca	4.13E-02
gene39342	23969	NC_000083.6	Pacsin1	4.12E-02
gene11227	108151	NC_000071.6	Sema3d	4.10E-02
gene34094	268729	NC_000080.6	Gm626	4.10E-02
gene25749	74782	NC_000076.6	Glt8d2	4.10E-02
gene3556	227835	NC_000068.7	Gtdc1	4.09E-02
gene38496	26559	NC_000082.6	Hunk	4.07E-02
gene22601	1E+08	NC_000075.6	Gm29724	4.07E-02
gene28371	320472	NC_000077.6	Ppm1e	4.06E-02
gene5782	57138	NC_000068.7	Slc12a5	4.06E-02
gene41586	320924	NC_000084.6	Ccbe1	4.06E-02
gene8119	77669	NC_000069.6	Arhgef38	4.04E-02
gene11998	212986	NC_000071.6	Scfd2	4.04E-02
gene22580	235086	NC_000075.6	Igsf9b	4.03E-02
gene14996	110958	NC_000072.6	M1ap	4.03E-02
gene14478	69993	NC_000072.6	Chn2	4.02E-02
gene22186	1E+08	NC_000074.6	Gm31718	4.02E-02
gene23111	244867	NC_000075.6	Arhgap20	4.02E-02
gene36136	320679	NC_000081.6	Samd12	4.00E-02
gene40712	=Gene;ger	NC_000084.6	LOC108168391	4.00E-02
gene37900	545156	NC_000082.6	Kalrn	4.00E-02
gene10454	78806	NC_000070.6	Stpg1	3.99E-02
gene19367	434232	NC_000073.6	Iqck	3.98E-02
gene35050	50759	NC_000080.6	Fbxo16	3.98E-02
gene14066	69923	NC_000072.6	Agk	3.97E-02
gene42064	622554	NC_000085.6	Majin	3.97E-02
gene13674	65255	NC_000072.6	Asb4	3.95E-02
gene41275	328949	NC_000084.6	Mcc	3.95E-02
gene8374	20447	NC_000069.6	St6galnac3	3.94E-02
gene29535	328061	NC_000078.6	Gm38407	3.94E-02
gene22696	245902	NC_000075.6	Ccdc15	3.94E-02
gene32649	-like;gbkey	NC_000079.6	LOC100861651	3.93E-02
gene10768	1.1E+08	NC_000070.6	Gm13165	3.92E-02
gene6696	433586	NC_000069.6	Maml3	3.92E-02
gene36297	380993	NC_000081.6	Zfat	3.92E-02
gene21179	73301	NC_000074.6	Ttc29	3.90E-02
gene26684	12323	NC_000077.6	Camk2b	3.89E-02
gene2582	19130	NC_000067.6	Prox1	3.89E-02
gene23821	78459	NC_000075.6	1700057G04Rik	3.88E-02
gene25109	75906	NC_000076.6	Fam184a	3.88E-02
gene42448	18552	NC_000085.6	Pcsk5	3.88E-02
gene5647	545481	NC_000068.7	Arhgap40	3.87E-02
gene8551	12557	NC_000070.6	Cdh17	3.87E-02
gene20934	1E+08	NC_000074.6	Gm15991	3.86E-02
gene20733	319940	NC_000074.6	Sorbs2os	3.85E-02

gene29927	319660	NC_000078.6	Agmo	3.84E-02
gene35058	71145	NC_000080.6	Scara5	3.84E-02
gene14373	140488	NC_000072.6	Igf2bp3	3.84E-02
gene12870	19894	NC_000071.6	Rph3a	3.84E-02
gene5471	74711	NC_000068.7	Ttll9	3.83E-02
gene19984	63830	NC_000073.6	Kcnq1ot1	3.83E-02
gene32673	77117	NC_000079.6	Zfp934	3.82E-02
gene41332	68857	NC_000084.6	Dtwd2	3.81E-02
gene41476	12322	NC_000084.6	Camk2a	3.81E-02
gene30806	74521	NC_000078.6	Ppp4r4	3.80E-02
gene29087	26399	NC_000077.6	Map2k6	3.79E-02
gene42612	19091	NC_000085.6	Prkg1	3.79E-02
gene11613	14772	NC_000071.6	Grk4	3.79E-02
gene37704	110197	NC_000082.6	Dgkg	3.79E-02
gene4730	20394	NC_000068.7	Scg5	3.78E-02
gene33272	17940	NC_000079.6	Naip1	3.77E-02
gene40691	240186	NC_000084.6	Zfp438	3.77E-02
gene22536	68283	NC_000075.6	9530077C05Rik	3.77E-02
gene10999	544678	NC_000070.6	Cfap74	3.76E-02
gene11726	665934	NC_000071.6	Gm7854	3.76E-02
gene674	381260	NC_000067.6	Gm973	3.74E-02
gene13538	1E+08	NC_000071.6	Gm15411	3.73E-02
gene26860	216560	NC_000077.6	Wdpcp	3.73E-02
gene6436	665113	NC_000069.6	Tnik	3.73E-02
gene13695	71238	NC_000072.6	Sdhaf3	3.73E-02
gene35757	320277	NC_000081.6	Spef2	3.71E-02
gene13938	73652	NC_000072.6	2210408F21Rik	3.70E-02
gene5226	75812	NC_000068.7	Tasp1	3.69E-02
gene25312	1.1E+08	NC_000076.6	Gm40683	3.67E-02
gene22157	244667	NC_000074.6	Disc1	3.67E-02
gene36282	54562	NC_000081.6	Lrrc6	3.65E-02
gene12764	12704	NC_000071.6	Cit	3.65E-02
gene31614	110067	NC_000079.6	Tcrg	3.64E-02
gene37429	74478	NC_000082.6	Snx29	3.63E-02
gene1830	545370	NC_000067.6	Hmcn1	3.63E-02
gene10041	60613	NC_000070.6	Kcnq4	3.63E-02
gene9586	18027	NC_000070.6	Nfia	3.63E-02
gene5846	1E+08	NC_000068.7	Gm11476	3.61E-02
gene14666	209590	NC_000072.6	Il23r	3.61E-02
gene8568	1.1E+08	NC_000070.6	Gm42256	3.59E-02
gene14021	330286	NC_000072.6	D630045J12Rik	3.59E-02
gene4726	14260	NC_000068.7	Fmn1	3.59E-02
gene34078	1E+08	NC_000080.6	3425401B19Rik	3.58E-02
gene42990	381229	NC_000085.6	Cfap58	3.58E-02
gene34047	1E+08	NC_000080.6	Gm35823	3.56E-02
gene4946	214968	NC_000068.7	Sema6d	3.56E-02
gene33441	218624	NC_000079.6	Il31ra	3.56E-02
gene2331	20739	NC_000067.6	Spta1	3.54E-02

gene6760	1E+08	NC_000069.6	Gm31914	3.54E-02
gene19803	212124	NC_000073.6	Cfap46	3.53E-02
gene35703	71432	NC_000081.6	5430437J10Rik	3.52E-02
gene37958	77782	NC_000082.6	Polq	3.50E-02
gene33973	110083	NC_000080.6	Dnah12	3.50E-02
gene34266	1E+08	NC_000080.6	Gm32857	3.48E-02
gene5	1.1E+08	NC_000067.6	Gm38717	3.48E-02
gene24968	75973	NC_000076.6	Ccdc162	3.48E-02
gene807	22779	NC_000067.6	Ikzf2	3.47E-02
gene38811	20519	NC_000083.6	Slc22a3	3.46E-02
gene41866	246102	NC_000084.6	Rttn	3.46E-02
gene9298	56079	NC_000070.6	Astn2	3.45E-02
gene7109	70762	NC_000069.6	Dclk2	3.44E-02
gene14511	232016	NC_000072.6	Ccdc129	3.44E-02
gene19977	17173	NC_000073.6	Ascl2	3.43E-02
gene18272	18553	NC_000073.6	Pcsk6	3.43E-02
gene25157	237353	NC_000076.6	Sh3rf3	3.43E-02
gene10942	260305	NC_000070.6	Nphp4	3.42E-02
gene27931	77577	NC_000077.6	Spns3	3.42E-02
gene2893	77543	NC_000068.7	8030442B05Rik	3.42E-02
gene18494	26557	NC_000073.6	Homer2	3.41E-02
gene5231	72899	NC_000068.7	Macrod2	3.40E-02
gene1252	381284	NC_000067.6	Crocc2	3.40E-02
gene26867	216565	NC_000077.6	Ehbp1	3.39E-02
gene8611	435772	NC_000070.6	Cnbd1	3.39E-02
gene22224	234889	NC_000075.6	Gucy1a2	3.37E-02
gene23249	382077	NC_000075.6	Ccdc33	3.37E-02
gene20637	319582	NC_000074.6	6430573F11Rik	3.36E-02
gene10967	22062	NC_000070.6	Trp73	3.34E-02
gene39079	1E+08	NC_000083.6	Gm16386	3.33E-02
gene9625	26563	NC_000070.6	Ror1	3.32E-02
gene4652	170442	NC_000068.7	Bbox1	3.32E-02
gene29760	67216	NC_000078.6	Mboat2	3.31E-02
gene21354	213435	NC_000074.6	Mylk3	3.31E-02
gene26303	117600	NC_000076.6	Srgap1	3.30E-02
gene10989	269615	NC_000070.6	Plch2	3.30E-02
gene23604	208994	NC_000075.6	Fam83b	3.30E-02
gene14222	29807	NC_000072.6	Tpk1	3.30E-02
gene22370	73732	NC_000075.6	Muc16	3.30E-02
gene8693	13841	NC_000070.6	Epha7	3.30E-02
gene16038	14917	NC_000072.6	Gucy2c	3.27E-02
gene1297	75159	NC_000067.6	4930533P14Rik	3.27E-02
gene23297	207596	NC_000075.6	Thsd4	3.26E-02
gene15568	12288	NC_000072.6	Cacna1c	3.24E-02
gene4760	17536	NC_000068.7	Meis2	3.23E-02
gene22538	319845	NC_000075.6	Bbs9	3.23E-02
gene1933	214575	NC_000067.6	Tdrd5	3.21E-02
gene3228	329375	NC_000068.7	Cfap77	3.20E-02

gene32956	11624	NC_000079.6	Ahrr	3.20E-02
gene30287	73936	NC_000078.6	Ccdc175	3.19E-02
gene5908	228911	NC_000068.7	Tshz2	3.19E-02
gene15275	330385	NC_000072.6	9530026P05Rik	3.18E-02
gene3742	20269	NC_000068.7	Scn3a	3.18E-02
gene24378	245049	NC_000075.6	Myrip	3.18E-02
gene32866	210044	NC_000079.6	Adcy2	3.17E-02
gene34658	1E+08	NC_000080.6	Gm30275	3.16E-02
gene1960	89867	NC_000067.6	Sec16b	3.16E-02
gene23046	235320	NC_000075.6	Zbtb16	3.16E-02
gene13999	243764	NC_000072.6	Chrm2	3.15E-02
gene41307	74574	NC_000084.6	Lvrn	3.15E-02
gene32880	78925	NC_000079.6	Srd5a1	3.15E-02
gene24952	83767	NC_000076.6	Wasf1	3.15E-02
gene814	12021	NC_000067.6	Bard1	3.14E-02
gene31611	218038	NC_000079.6	Amph	3.13E-02
gene5919	76960	NC_000068.7	Bcas1	3.13E-02
gene9834	666048	NC_000070.6	Trabd2b	3.13E-02
gene17352	20190	NC_000073.6	Ryr1	3.13E-02
gene29632	668525	NC_000078.6	Gm9222	3.12E-02
gene10779	1E+08	NC_000070.6	Gm13248	3.10E-02
gene40108	211482	NC_000083.6	Efhb	3.09E-02
gene394	16764	NC_000067.6	Aff3	3.08E-02
gene35834	215654	NC_000081.6	Cdh12	3.08E-02
gene27543	17910	NC_000077.6	Myo15	3.08E-02
gene16229	12121	NC_000072.6	Bicd1	3.06E-02
gene12672	320916	NC_000071.6	Wscd2	3.04E-02
gene23892	64085	NC_000075.6	Clstn2	3.03E-02
gene12828	1E+08	NC_000071.6	Tbx3os1	3.03E-02
gene22619	12023	NC_000075.6	Barx2	3.02E-02
gene35952	626596	NC_000081.6	Rgs22	3.01E-02
gene11601	231125	NC_000071.6	Zfyve28	3.01E-02
gene26993	18197	NC_000077.6	Nsg2	2.99E-02
gene19733	66930	NC_000073.6	Fank1	2.98E-02
gene14019	140494	NC_000072.6	Atp6v0a4	2.97E-02
gene7698	1E+08	NC_000069.6	Gm34049	2.95E-02
gene9908	74464	NC_000070.6	Zswim5	2.95E-02
gene35242	70859	NC_000080.6	Lrrc63	2.94E-02
gene14461	231986	NC_000072.6	Jazf1	2.93E-02
gene35662	223272	NC_000080.6	Itgbl1	2.93E-02
gene13840	214704	NC_000072.6	Iqub	2.92E-02
gene2779	320132	NC_000068.7	A230108P19Rik	2.92E-02
gene13792	30785	NC_000072.6	Cttnbp2	2.92E-02
gene22867	70989	NC_000075.6	Jhy	2.92E-02
gene18511	269959	NC_000073.6	Adamtsl3	2.91E-02
gene22246	110350	NC_000075.6	Dync2h1	2.90E-02
gene14232	66797	NC_000072.6	Cntnap2	2.89E-02
gene42081	18190	NC_000085.6	Nrxn2	2.89E-02

gene31975	210108	NC_000079.6	D130043K22Rik	2.88E-02
gene25247	52463	NC_000076.6	Tet1	2.88E-02
gene20634	1E+08	NC_000074.6	Gm35712	2.86E-02
gene1041	1E+08	NC_000067.6	Gm38510	2.85E-02
gene117	14048	NC_000067.6	Eya1	2.85E-02
gene11839	54216	NC_000071.6	Pcdh7	2.85E-02
gene35642	328479	NC_000080.6	Gm5089	2.82E-02
gene7123	329693	NC_000069.6	Fcrl5	2.82E-02
gene3671	76654	NC_000068.7	Upp2	2.79E-02
gene4647	107515	NC_000068.7	Lgr4	2.78E-02
gene6072	433520	NC_000068.7	Gm14403	2.78E-02
gene1476	73103	NC_000067.6	3110009E18Rik	2.78E-02
gene43089	14585	NC_000085.6	Gfra1	2.78E-02
gene36086	192190	NC_000081.6	Pkhd1l1	2.78E-02
gene34124	24131	NC_000080.6	Ldb3	2.78E-02
gene8601	1E+08	NC_000070.6	A530072M11Rik	2.77E-02
gene32722	1E+08	NC_000079.6	Gm31218	2.75E-02
gene9659	73094	NC_000070.6	Sgip1	2.75E-02
gene25313	73287	NC_000076.6	1700040L02Rik	2.73E-02
gene38668	24001	NC_000083.6	Tiam2	2.71E-02
gene42700	226098	NC_000085.6	Hectd2	2.70E-02
gene38913	1.1E+08	NC_000083.6	Gm41534	2.70E-02
gene33103	66795	NC_000079.6	Atg10	2.68E-02
gene41425	75444	NC_000084.6	1700011I03Rik	2.67E-02
gene26756	13195	NC_000077.6	Ddc	2.66E-02
gene2387	269152	NC_000067.6	Kif26b	2.63E-02
gene40925	1E+08	NC_000084.6	Gm33228	2.63E-02
gene4639	76894	NC_000068.7	Mettl15	2.62E-02
gene7028	54635	NC_000069.6	Pdgfc	2.62E-02
gene37473	67254	NC_000082.6	2900011O08Rik	2.60E-02
gene6497	72607	NC_000069.6	Usp13	2.60E-02
gene40809	225207	NC_000084.6	Zfp521	2.58E-02
gene8368	26938	NC_000069.6	St6galnac5	2.57E-02
gene5288	408057	NC_000068.7	BC039771	2.57E-02
gene9271	373864	NC_000070.6	Col27a1	2.57E-02
gene11157	242819	NC_000071.6	Rundc3b	2.56E-02
gene20244	17930	NC_000074.6	Myom2	2.55E-02
gene25153	13608	NC_000076.6	Edar	2.55E-02
gene41070	1E+08	NC_000084.6	Nrg2	2.53E-02
gene6985	20713	NC_000069.6	Serpini1	2.53E-02
gene16214	232560	NC_000072.6	Caprin2	2.53E-02
gene27676	237806	NC_000077.6	Dnah9	2.53E-02
gene23646	235505	NC_000075.6	Cd109	2.53E-02
gene35170	57746	NC_000080.6	Piwil2	2.51E-02
gene6026	665001	NC_000068.7	Gm14391	2.50E-02
gene3587	71897	NC_000068.7	Lypd6b	2.49E-02
gene32434	26564	NC_000079.6	Ror2	2.48E-02
gene33800	21834	NC_000080.6	Thrb	2.48E-02

gene34378	67419	NC_000080.6	3632451O06Rik	2.43E-02
gene2018	103967	NC_000067.6	Dnm3	2.42E-02
gene4595	211936	NC_000068.7	Ccdc73	2.42E-02
gene32038	68916	NC_000079.6	Cdkal1	2.42E-02
gene36406	223642	NC_000081.6	Zc3h3	2.42E-02
gene8977	666060	NC_000070.6	Frmpd1	2.42E-02
gene5020	74121	NC_000068.7	Acoxl	2.41E-02
gene21162	110784	NC_000074.6	Nr3c2	2.41E-02
gene2989	70882	NC_000068.7	Armc3	2.39E-02
gene42559	19726	NC_000085.6	Rfx3	2.39E-02
gene2162	70729	NC_000067.6	Nos1ap	2.38E-02
gene26746	53604	NC_000077.6	Zpbp	2.37E-02
gene40878	381126	NC_000084.6	Garem	2.37E-02
gene12770	269693	NC_000071.6	Ccdc60	2.36E-02
gene5083	228598	NC_000068.7	Ebf4	2.36E-02
gene24561	338362	NC_000076.6	Ust	2.35E-02
gene16120	20449	NC_000072.6	St8sia1	2.35E-02
gene33131	76469	NC_000079.6	Cmya5	2.35E-02
gene31410	13411	NC_000078.6	Dnah11	2.35E-02
gene6079	1E+08	NC_000068.7	Gm14412	2.34E-02
gene12233	320696	NC_000071.6	Ccdc158	2.34E-02
gene712	72823	NC_000067.6	Pard3b	2.32E-02
gene12080	13839	NC_000071.6	Epha5	2.32E-02
gene11173	231014	NC_000071.6	9330182L06Rik	2.32E-02
gene31982	195208	NC_000079.6	Dcdc2a	2.32E-02
gene27392	245827	NC_000077.6	Fat2	2.31E-02
gene35459	76619	NC_000080.6	1700087I21Rik	2.31E-02
gene19344	233781	NC_000073.6	Xylt1	2.28E-02
gene18105	14453	NC_000073.6	Gas2	2.28E-02
gene11262	12293	NC_000071.6	Cacna2d1	2.28E-02
gene12400	231532	NC_000071.6	Arhgap24	2.27E-02
gene34294	70713	NC_000080.6	Gpr137c	2.26E-02
gene27358	1E+08	NC_000077.6	Gm12223	2.26E-02
gene42641	67795	NC_000085.6	Rnls	2.24E-02
gene24575	215772	NC_000076.6	Adgb	2.24E-02
gene4010	241520	NC_000068.7	Fam171b	2.24E-02
gene41568	225638	NC_000084.6	Alpk2	2.23E-02
gene12988	56087	NC_000071.6	Dnah10	2.22E-02
gene13646	12311	NC_000072.6	Calcr	2.22E-02
gene8662	212390	NC_000070.6	Klhl32	2.22E-02
gene39931	109115	NC_000083.6	Supt3	2.22E-02
gene41347	71373	NC_000084.6	Prr16	2.21E-02
gene8643	76132	NC_000070.6	Faxc	2.21E-02
gene24829	72780	NC_000076.6	Rspo3	2.20E-02
gene11638	231148	NC_000071.6	Ablim2	2.20E-02
gene4756	399568	NC_000068.7	BC052040	2.19E-02
gene3585	16574	NC_000068.7	Kif5c	2.19E-02
gene21154	71831	NC_000074.6	1700007B14Rik	2.19E-02

gene36238	19296	NC_000081.6	Pvt1	2.19E-02
gene23840	331004	NC_000075.6	Slc9a9	2.18E-02
gene23894	74080	NC_000075.6	Nmnat3	2.17E-02
gene24402	209012	NC_000075.6	Ulk4	2.16E-02
gene12603	231605	NC_000071.6	Galnt9	2.15E-02
gene8577	12395	NC_000070.6	Runx1t1	2.14E-02
gene1680	329252	NC_000067.6	Lgr6	2.14E-02
gene28435	1E+08	NC_000077.6	Gm34726	2.14E-02
gene2757	74186	NC_000068.7	Ccdc3	2.14E-02
gene30360	20741	NC_000078.6	Sptb	2.14E-02
gene25029	1E+08	NC_000076.6	Gm35154	2.13E-02
gene38192	12837	NC_000082.6	Col8a1	2.13E-02
gene20729	234214	NC_000074.6	Sorbs2	2.11E-02
gene6483	72413	NC_000069.6	Kcnmb2	2.11E-02
gene27528	18618	NC_000077.6	Pemt	2.10E-02
gene971	1E+08	NC_000067.6	Gm29536	2.10E-02
gene29393	71276	NC_000077.6	Ccdc57	2.08E-02
gene3515	14536	NC_000068.7	Nr6a1	2.08E-02
gene8474	242297	NC_000070.6	Fam110b	2.08E-02
gene26048	74978	NC_000076.6	Lrriq1	2.07E-02
gene4787	71313	NC_000068.7	Fsip1	2.06E-02
gene24803	16773	NC_000076.6	Lama2	2.06E-02
gene27111	11548	NC_000077.6	Adra1b	2.05E-02
gene40563	73582	NC_000083.6	Camkmt	2.05E-02
gene6902	624866	NC_000069.6	Lekr1	2.04E-02
gene32061	218138	NC_000079.6	Gmds	2.03E-02
gene7	19888	NC_000067.6	Rp1	2.02E-02
gene40693	791318	NC_000084.6	Gm10125	2.01E-02
gene15360	232313	NC_000072.6	Gxylt2	2.01E-02
gene16415	57775	NC_000073.6	Usp29	1.96E-02
gene13558	381693	NC_000071.6	Wdr95	1.96E-02
gene1343	320311	NC_000067.6	Rnf152	1.96E-02
gene21799	244653	NC_000074.6	Hydin	1.94E-02
gene33392	238871	NC_000079.6	Pde4d	1.94E-02
gene16099	28254	NC_000072.6	Slco1a6	1.93E-02
gene2391	69726	NC_000067.6	Smyd3	1.93E-02
gene30438	64075	NC_000078.6	Smoc1	1.92E-02
gene6413	192167	NC_000069.6	Nlgn1	1.92E-02
gene23268	73673	NC_000075.6	Rec114	1.92E-02
gene40345	=Gene;ger	NC_000083.6	LOC105246307	1.91E-02
gene29990	664883	NC_000078.6	Nova1	1.89E-02
gene33084	13612	NC_000079.6	Edil3	1.88E-02
gene3729	60344	NC_000068.7	Fign	1.88E-02
gene41251	269019	NC_000084.6	Stk32a	1.88E-02
gene37608	111519	NC_000082.6	Igl	1.87E-02
gene36771	72301	NC_000081.6	1810041L15Rik	1.86E-02
gene41733	71206	NC_000084.6	Katnal2	1.85E-02
gene9307	56710	NC_000070.6	Brinp1	1.83E-02

gene23251	20897	NC_000075.6	Stra6	1.81E-02
gene27121	13591	NC_000077.6	Ebf1	1.81E-02
gene23989	81907	NC_000075.6	Tmem108	1.81E-02
gene8216	22253	NC_000069.6	Unc5c	1.80E-02
gene33781	218763	NC_000080.6	Lrrc3b	1.80E-02
gene368	70853	NC_000067.6	Vwa3b	1.80E-02
gene12071	319387	NC_000071.6	Adgrl3	1.80E-02
gene42845	545291	NC_000085.6	Hpse2	1.80E-02
gene6400	1E+08	NC_000069.6	Gm31813	1.79E-02
gene9197	634731	NC_000070.6	Susd1	1.79E-02
gene36041	64705	NC_000081.6	Dpys	1.79E-02
gene35425	16597	NC_000080.6	Klf12	1.79E-02
gene29920	75847	NC_000078.6	Ispd	1.78E-02
gene33888	76633	NC_000080.6	1700112E06Rik	1.77E-02
gene5867	69553	NC_000068.7	Fam65c	1.76E-02
gene36309	1E+08	NC_000081.6	Gm19782	1.75E-02
gene31140	111507	NC_000078.6	Igh	1.73E-02
gene19767	17314	NC_000073.6	Mgmt	1.73E-02
gene25906	380654	NC_000076.6	Cfap54	1.72E-02
gene36918	239606	NC_000081.6	Slc2a13	1.71E-02
gene35367	56419	NC_000080.6	Diaph3	1.70E-02
gene6860	269437	NC_000069.6	Plch1	1.70E-02
gene33344	52882	NC_000079.6	Rgs7bp	1.70E-02
gene18646	109264	NC_000073.6	Me3	1.69E-02
gene40714	74934	NC_000084.6	Armc4	1.69E-02
gene24735	14051	NC_000076.6	Eya4	1.68E-02
gene32332	75607	NC_000079.6	Wnk2	1.68E-02
gene7240	140493	NC_000069.6	Kcnn3	1.67E-02
gene33791	218772	NC_000080.6	Rarb	1.67E-02
gene40286	381107	NC_000083.6	Tmem232	1.67E-02
gene35931	17181	NC_000081.6	Matn2	1.67E-02
gene35640	69634	NC_000080.6	Clybl	1.66E-02
gene9414	320226	NC_000070.6	Ccdc171	1.63E-02
gene13819	19283	NC_000072.6	Ptprz1	1.60E-02
gene21874	80707	NC_000074.6	Wwox	1.59E-02
gene33009	72371	NC_000079.6	2210408I21Rik	1.59E-02
gene10909	100072	NC_000070.6	Camta1	1.57E-02
gene19699	55987	NC_000073.6	Cpxm2	1.56E-02
gene1442	14633	NC_000067.6	Gli2	1.56E-02
gene11499	65254	NC_000071.6	Dpysl5	1.55E-02
gene30117	73490	NC_000078.6	Mipol1	1.54E-02
gene29889	79221	NC_000078.6	Hdac9	1.54E-02
gene33775	674895	NC_000080.6	Nek10	1.53E-02
gene2365	1E+08	NC_000067.6	Gm36904	1.53E-02
gene33930	414118	NC_000080.6	Zmiz1os1	1.53E-02
gene9644	72685	NC_000070.6	Dnajc6	1.53E-02
gene26953	237711	NC_000077.6	Eml6	1.52E-02
gene5224	228677	NC_000068.7	Sptlc3	1.52E-02

gene42563	226075	NC_000085.6	Glis3	1.51E-02
gene809	66722	NC_000067.6	Spag16	1.50E-02
gene2376	545391	NC_000067.6	Gm16432	1.49E-02
gene24017	208583	NC_000075.6	Nek11	1.49E-02
gene32306	320614	NC_000079.6	A330033J07Rik	1.47E-02
gene24566	320825	NC_000076.6	Samd5	1.44E-02
gene2640	16510	NC_000067.6	Kcnh1	1.44E-02
gene27492	380698	NC_000077.6	Obscn	1.44E-02
gene12327	75784	NC_000071.6	1700007G11Rik	1.43E-02
gene101	240726	NC_000067.6	Slco5a1	1.41E-02
gene8218	12167	NC_000069.6	Bmpr1b	1.40E-02
gene41292	1E+08	NC_000084.6	Gm32288	1.40E-02
gene3759	241431	NC_000068.7	Xirp2	1.39E-02
gene24522	13982	NC_000076.6	Esr1	1.39E-02
gene40628	14309	NC_000083.6	Fshr	1.38E-02
gene2648	329324	NC_000067.6	Syt14	1.37E-02
gene38793	69310	NC_000083.6	Pacrg	1.37E-02
gene8507	74438	NC_000070.6	Clvs1	1.36E-02
gene27033	70357	NC_000077.6	Kcnip1	1.34E-02
gene4621	77015	NC_000068.7	Mpped2	1.34E-02
gene24996	109205	NC_000076.6	Sobp	1.33E-02
gene1046	ene;gene=L	NC_000067.6	LOC101055758	1.32E-02
gene35862	448987	NC_000081.6	Fbxl7	1.32E-02
gene9559	230451	NC_000070.6	Junos	1.32E-02
gene32282	67252	NC_000079.6	Cap2	1.32E-02
gene12573	636173	NC_000071.6	Vmn2r-ps23	1.32E-02
gene41440	78923	NC_000084.6	Chsy3	1.32E-02
gene36630	239556	NC_000081.6	Cacna1i	1.31E-02
gene30466	50779	NC_000078.6	Rgs6	1.31E-02
gene14627	70779	NC_000072.6	Prdm5	1.31E-02
gene24318	207181	NC_000075.6	Rbms3	1.29E-02
gene23568	546144	NC_000075.6	Wdr72	1.28E-02
gene15435	259302	NC_000072.6	Srgap3	1.28E-02
gene20892	21892	NC_000074.6	Tll1	1.28E-02
gene3978	18573	NC_000068.7	Pde1a	1.27E-02
gene30732	78767	NC_000078.6	Efcab11	1.26E-02
gene23387	214058	NC_000075.6	Megf11	1.25E-02
gene36977	1E+08	NC_000081.6	Gm38469	1.25E-02
gene20415	11733	NC_000074.6	Ank1	1.25E-02
gene25799	74007	NC_000076.6	Btbd11	1.21E-02
gene9267	72701	NC_000070.6	Zfp618	1.19E-02
gene913	75570	NC_000067.6	Nhej1	1.19E-02
gene10793	ene=LOC1	NC_000070.6	LOC108168963	1.18E-02
gene26075	380660	NC_000076.6	Acss3	1.18E-02
gene27412	14799	NC_000077.6	Gria1	1.17E-02
gene10490	13844	NC_000070.6	Ephb2	1.16E-02
gene9426	242509	NC_000070.6	Bnc2	1.16E-02
gene26252	74053	NC_000076.6	Grip1	1.15E-02

gene21396	94187	NC_000074.6	Zfp423	1.14E-02
gene11152	11496	NC_000071.6	Adam22	1.14E-02
gene18288	233332	NC_000073.6	Adamts17	1.13E-02
gene33402	67295	NC_000079.6	Rab3c	1.12E-02
gene9368	19266	NC_000070.6	Ptprd	1.11E-02
gene41360	67847	NC_000084.6	Sncaip	1.10E-02
gene12165	330119	NC_000071.6	Adamts3	1.09E-02
gene41718	207259	NC_000084.6	Zbtb7c	1.09E-02
gene4615	329482	NC_000068.7	Dcdc5	1.08E-02
gene25880	77531	NC_000076.6	Anks1b	1.05E-02
gene4577	241589	NC_000068.7	D430041D05Rik	1.04E-02
gene31584	14634	NC_000079.6	Gli3	1.04E-02
gene8717	140577	NC_000070.6	Ankrd6	1.03E-02
gene38313	268902	NC_000082.6	Robo2	1.01E-02
gene33728	74430	NC_000080.6	4930452B06Rik	1.00E-02
gene3307	665700	NC_000068.7	Hmcn2	9.95E-03
gene38875	71682	NC_000083.6	Wdr27	9.90E-03
gene27161	24052	NC_000077.6	Sgcd	9.90E-03
gene3939	22138	NC_000068.7	Ttn	9.86E-03
gene2812	353282	NC_000068.7	Sfmbt2	9.85E-03
gene29480	208439	NC_000078.6	Klhl29	9.82E-03
gene1968	23850	NC_000067.6	Pappa2	9.72E-03
gene30037	76826	NC_000078.6	Nubpl	9.61E-03
gene1543	210417	NC_000067.6	Thsd7b	9.50E-03
gene12894	13048	NC_000071.6	Cux2	9.44E-03
gene40381	11682	NC_000083.6	Alk	9.44E-03
gene35292	239188	NC_000080.6	Enox1	9.38E-03
gene14599	232035	NC_000072.6	Ccser1	9.34E-03
gene21304	12286	NC_000074.6	Cacna1a	9.17E-03
gene2935	12296	NC_000068.7	Cacnb2	9.08E-03
gene3950	241494	NC_000068.7	Zfp385b	9.07E-03
gene22874	73144	NC_000075.6	3110039I08Rik	9.05E-03
gene22166	270109	NC_000074.6	Pcnx2	9.04E-03
gene3434	74410	NC_000068.7	Ttll11	8.91E-03
gene36756	77627	NC_000081.6	Efcab6	8.88E-03
gene9297	18491	NC_000070.6	Pappa	8.60E-03
gene11454	654796	NC_000071.6	9530036O11Rik	8.46E-03
gene944	1E+08	NC_000067.6	Gm32622	8.42E-03
gene20006	210274	NC_000073.6	Shank2	8.34E-03
gene12037	320827	NC_000071.6	C530008M17Rik	8.25E-03
gene6888	16497	NC_000069.6	Kcnab1	8.20E-03
gene40840	12558	NC_000084.6	Cdh2	8.12E-03
gene30402	19363	NC_000078.6	Rad51b	8.08E-03
gene23034	244853	NC_000075.6	Nxpe4	8.06E-03
gene40256	13640	NC_000083.6	Efna5	7.99E-03
gene6448	75740	NC_000069.6	Egfem1	7.91E-03
gene28430	382543	NC_000077.6	Ankfn1	7.89E-03
gene22354	270120	NC_000075.6	Fat3	7.87E-03

gene19734	11489	NC_000073.6	Adam12	7.86E-03
gene11315	19699	NC_000071.6	Reln	7.83E-03
gene29047	76380	NC_000077.6	Cep112	7.78E-03
gene13763	114142	NC_000072.6	Foxp2	7.56E-03
gene35597	50787	NC_000080.6	Hs6st3	7.48E-03
gene20548	211323	NC_000074.6	Nrg1	7.48E-03
gene621	212712	NC_000067.6	Satb2	7.45E-03
gene8485	252838	NC_000070.6	Tox	7.42E-03
gene4905	228564	NC_000068.7	Frmd5	7.31E-03
gene22994	114873	NC_000075.6	Dscaml1	7.31E-03
gene35887	18163	NC_000081.6	Ctnnd2	7.17E-03
gene40076	319991	NC_000083.6	Kif6	7.16E-03
gene22176	210027	NC_000074.6	Slc35f3	7.08E-03
gene18257	70638	NC_000073.6	Fam189a1	6.98E-03
gene36052	22762	NC_000081.6	Zfpm2	6.91E-03
gene41869	623279	NC_000084.6	Dok6	6.89E-03
gene9435	77739	NC_000070.6	Adamtsl1	6.87E-03
gene2641	226861	NC_000067.6	Hhat	6.84E-03
gene2339	54418	NC_000067.6	Fmn2	6.82E-03
gene19323	20679	NC_000073.6	Sox6	6.80E-03
gene40922	108013	NC_000084.6	Celf4	6.76E-03
gene31518	12671	NC_000079.6	Chrm3	6.76E-03
gene38306	19876	NC_000082.6	Robo1	6.72E-03
gene9167	677884	NC_000070.6	Pakap	6.72E-03
gene38262	13837	NC_000082.6	Epha3	6.58E-03
gene36927	12805	NC_000081.6	Cntn1	6.53E-03
gene3798	329421	NC_000068.7	Myo3b	6.42E-03
gene36833	106014	NC_000081.6	Fam19a5	6.33E-03
gene7926	99586	NC_000069.6	Dpyd	6.33E-03
gene13678	13426	NC_000072.6	Dync1i1	6.25E-03
gene41245	72930	NC_000084.6	Ppp2r2b	6.18E-03
gene544	56363	NC_000067.6	Tmeff2	6.17E-03
gene9566	75578	NC_000070.6	Fggy	6.16E-03
gene11943	243043	NC_000071.6	Kctd8	6.14E-03
gene37767	22061	NC_000082.6	Trp63	6.13E-03
gene8030	109676	NC_000069.6	Ank2	6.10E-03
gene204	226922	NC_000067.6	Kcnq5	6.06E-03
gene14003	320127	NC_000072.6	Dgki	5.86E-03
gene33753	72003	NC_000080.6	Synpr	5.86E-03
gene517	227058	NC_000067.6	Dnah7b	5.81E-03
gene3930	241489	NC_000068.7	Pde11a	5.72E-03
gene16072	18705	NC_000072.6	Pik3c2g	5.65E-03
gene12297	231470	NC_000071.6	Fras1	5.59E-03
gene31523	20191	NC_000079.6	Ryr2	5.48E-03
gene6119	12561	NC_000068.7	Cdh4	5.48E-03
gene16135	20678	NC_000072.6	Sox5	5.06E-03
gene29962	93757	NC_000078.6	Immp2l	4.80E-03
gene56	71096	NC_000067.6	Sntg1	4.78E-03

gene40918	225288	NC_000084.6	Fhod3	4.78E-03
gene27025	66011	NC_000077.6	Ranbp17	4.49E-03
gene18663	23859	NC_000073.6	Dlg2	4.44E-03
gene21919	12554	NC_000074.6	Cdh13	4.37E-03
gene13739	330267	NC_000072.6	Thsd7a	4.13E-03
gene36957	320709	NC_000081.6	Tmem117	4.05E-03
gene11284	50791	NC_000071.6	Magi2	4.05E-03
gene30045	238161	NC_000078.6	Akap6	3.99E-03
gene13348	330222	NC_000071.6	Sdk1	3.61E-03
gene11792	19017	NC_000071.6	Ppargc1a	3.60E-03
gene6299	76897	NC_000069.6	Ralyl	3.57E-03
gene25379	11994	NC_000076.6	Pcdh15	3.52E-03
gene0	497097	NC_000067.6	Xkr4	3.38E-03
gene5680	19281	NC_000068.7	Ptprt	3.21E-03
gene3761	26877	NC_000068.7	B3galt1	3.02E-03
gene31593	192136	NC_000079.6	Sugct	3.01E-03
gene15305	320265	NC_000072.6	Fam19a1	2.94E-03
gene8412	320840	NC_000069.6	Negr1	2.89E-03
gene40341	224997	NC_000083.6	Dlgap1	2.69E-03
gene13092	319974	NC_000071.6	Auts2	2.60E-03
gene35664	14169	NC_000080.6	Fgf14	2.52E-03
gene33989	238988	NC_000080.6	Erc2	2.50E-03
gene38797	50873	NC_000083.6	Park2	2.46E-03
gene18394	244071	NC_000073.6	Agbl1	2.39E-03
gene18690	23966	NC_000073.6	Tenm4	2.14E-03
gene11773	80334	NC_000071.6	Kcnip4	2.11E-03
gene34503	21473	NC_000080.6	Tcra	2.10E-03
gene20248	94109	NC_000074.6	Csmd1	1.83E-03
gene33735	14198	NC_000080.6	Fhit	1.50E-03
gene9802	78933	NC_000070.6	Agbl4	1.36E-03
gene3541	94217	NC_000068.7	Lrp1b	1.27E-03
gene14606	14804	NC_000072.6	Grid2	1.19E-03
