# Supplementary material for: Targeted RNA-Seq Reveals the M. tuberculosis Transcriptome from an In Vivo Infection Model
Source: Biology (Basel). 2021 Aug 31;10(9):848. doi: 10.3390/biology10090848 (PMC8467220; doi:10.3390/biology10090848)
Supplement: Supplementary file 1 [file biology-10-00848-s001.zip › TableS8_r1.pdf]

Tabla S8. Gene Ontology terms significantly enriched by category related to the most expressed mouse genes

GO ID	GO name	P-Value
**A. Biological Process**		
GO:0043903	regulation of symbiosis, encompassing mutualism through parasitism	3.23E-06
GO:0042981	regulation of apoptotic process	5.37E-06
GO:0002683	negative regulation of immune system process	1.05E-05
GO:0002478	antigen processing and presentation of exogenous peptide antigen	1.68E-04
GO:0043065	positive regulation of apoptotic process	2.71E-04
GO:0002831	regulation of response to biotic stimulus	3.67E-04
GO:0045597	positive regulation of cell differentiation	5.05E-04
GO:0070374	positive regulation of ERK1 and ERK2 cascade	5.33E-04
GO:0071356	cellular response to tumor necrosis factor	6.52E-04
GO:0019730	antimicrobial humoral response	8.82E-04
GO:0006915	apoptotic process	1.27E-03
GO:0071222	cellular response to lipopolysaccharide	2.15E-03
GO:0051851	modulation by host of symbiont process	2.57E-03
GO:1902105	regulation of leukocyte differentiation	3.73E-03
GO:0097237	cellular response to toxic substance	5.09E-03
GO:0030097	hemopoiesis	5.40E-03
GO:0001817	regulation of cytokine production	5.60E-03
GO:0045765	regulation of angiogenesis	6.22E-03
GO:0035458	cellular response to interferon-beta	6.48E-03
GO:0034341	response to interferon-gamma	7.20E-03
**B. Cellular Component**		
GO:0005615	extracellular space	1.11E-12
GO:0043209	myelin sheath	1.60E-08
GO:0042824	MHC class I peptide loading complex	2.65E-08
GO:0042612	MHC class I protein complex	1.80E-05
GO:0030670	phagocytic vesicle membrane	3.45E-05
GO:0062023	collagen-containing extracellular matrix	5.45E-05
GO:0045121	membrane raft	7.45E-05
GO:0005794	Golgi apparatus	2.92E-03
GO:0005839	proteasome core complex	5.17E-03
GO:0005764	lysosome	5.21E-03
GO:0022626	cytosolic ribosome	5.36E-03
GO:0031966	mitochondrial membrane	6.05E-03
GO:0009897	external side of plasma membrane	1.04E-02
GO:0036464	cytoplasmic ribonucleoprotein granule	1.26E-02
GO:0048471	perinuclear region of cytoplasm	1.41E-02
GO:0120025	plasma membrane bounded cell projection	2.02E-02
GO:0005634	nucleus	3.21E-02
**C. Molecular Function**		
GO:0044877	protein-containing complex binding	1.08E-09
GO:0042802	identical protein binding	4.48E-08
GO:0005198	structural molecule activity	2.29E-04
GO:0042605	peptide antigen binding	8.60E-04
GO:0030881	beta-2-microglobulin binding	8.66E-04
GO:0003924	GTPase activity	9.41E-04
GO:0008009	chemokine activity	1.33E-03
GO:0030234	enzyme regulator activity	1.69E-03
GO:0045236	CXCR chemokine receptor binding	2.98E-03
GO:0042608	T cell receptor binding	3.88E-03
GO:0004888	transmembrane signaling receptor activity	5.05E-03
GO:0016209	antioxidant activity	6.95E-03
GO:0004298	threonine-type endopeptidase activity	1.21E-02
GO:0050998	nitric-oxide synthase binding	1.47E-02
GO:0019843	rRNA binding	2.58E-02
GO:0042610	CD8 receptor binding	2.88E-02
GO:0005525	GTP binding	4.02E-02
GO:0046979	TAP2 binding	4.73E-02
GO:0046978	TAP1 binding	4.73E-02
