# Supplementary material for: Targeted RNA-Seq Reveals the M. tuberculosis Transcriptome from an In Vivo Infection Model
Source: Biology (Basel). 2021 Aug 31;10(9):848. doi: 10.3390/biology10090848 (PMC8467220; doi:10.3390/biology10090848)
Supplement: Supplementary file 1 [file biology-10-00848-s001.zip › TableS9_r1.pdf]

Table S9. KEGG pathways overrepresented by the most expressed mouse genes.

KEGG ID	Pathway	%
03010	Ribosome	37.71
03050	Proteasome	27.66
00190	Oxidative phosphorylation	26.32
03060	Protein export	17.86
04612	Antigen processing and presentati	17.78
04714	Thermogenesis	14.71
04145	Phagosome	14.36
04142	Lysosome	12.21
04216	Ferroptosis	12.20
04657	IL-17 signaling pathway	12.09
04668	TNF signaling pathway	11.50
05152	Tuberculosis	10.44
04620	Toll-like receptor signaling pathway	10.10
04062	Chemokine signaling pathway	9.69
04380	Osteoclast differentiation	9.56
04670	Leukocyte transendothelial migrati	9.48
04623	Cytosolic DNA-sensing pathway	9.38
00010	Glycolysis / Gluconeogenesis	9.09
04625	C-type lectin receptor signaling pat	8.93
04210	Apoptosis	8.82
